# A unified approach for divergent synthesis of contiguous stereodiads employing a small boronyl group

**DOI:** 10.1038/s41467-020-14592-7

**Published:** 2020-02-07

**Authors:** Miao Zhan, Zhengwei Ding, Shaozhi Du, Haohua Chen, Chao Feng, Ming Xu, Zhi Liu, Mengxi Zhang, Chao Wu, Yu Lan, Pengfei Li

**Affiliations:** 1 Frontier Institute of Science and Technology, Xi’an Jiaotong University, 99 Yanxiang Road, Xi’an, 710054 China; 2 Institute of Medical Research, Northwestern Polytechnical University, Xi’an, 710072 China; 3 College of Chemistry & Pharmacy, Northwest A&F University, 22 Xinong Road, Yangling, 712100 China; 4 School of Chemistry and Chemical Engineering, and Chongqing Key Laboratory of Theoretical and Computational Chemistry, Chongqing University, Chongqing, 400030 China; 5 College of Chemistry, Zhengzhou University, Zhengzhou, 450001 China; 6 State Key Laboratory of Elemento-Organic Chemistry, Nankai University, Tianjin, 300071 China; 7 Xi’an Key Laboratory of Sustainable Energy Materials Chemistry, Xi’an Jiaotong University, Xi’an, 710049 China

**Keywords:** Reaction mechanisms, Stereochemistry, Synthetic chemistry methodology

## Abstract

Acyclic contiguous stereocenters are frequently seen in biologically active natural and synthetic molecules. Although various synthetic methods have been reported, predictable and unified approaches to all possible stereoisomers are rare, particularly for those containing non-reactive hydrocarbon substituents. Herein, a *β*-boronyl group is employed as a readily accessible handle for predictable *α*-functionalization of enolates with either *syn* or *anti* selectivity depending on reaction conditions. Contiguous tertiary-tertiary and tertiary-quaternary stereocenters are thus accessed in generally good yields and diastereoselectivity. Based on experimental and computational studies, mechanism for *syn* selective alkylation is proposed, and Bpin (pinacolatoboronyl) behaves as a smaller group than most carbon-centered groups. The synthetic utility of this methodology is demonstrated by preparation of several key intermediates for bioactive molecules.

## Abstract (web summary)

Predictable and unified approaches to all possible stereoisomers of acyclic compounds with contiguous stereocentres are rare. Here, the authors disclose a divergent *α*-functionalization of enolates with either *syn* or *anti* selectivity employing a *β*-boronyl group as a small, directing handle.

## Introduction

**Fig. 1:**
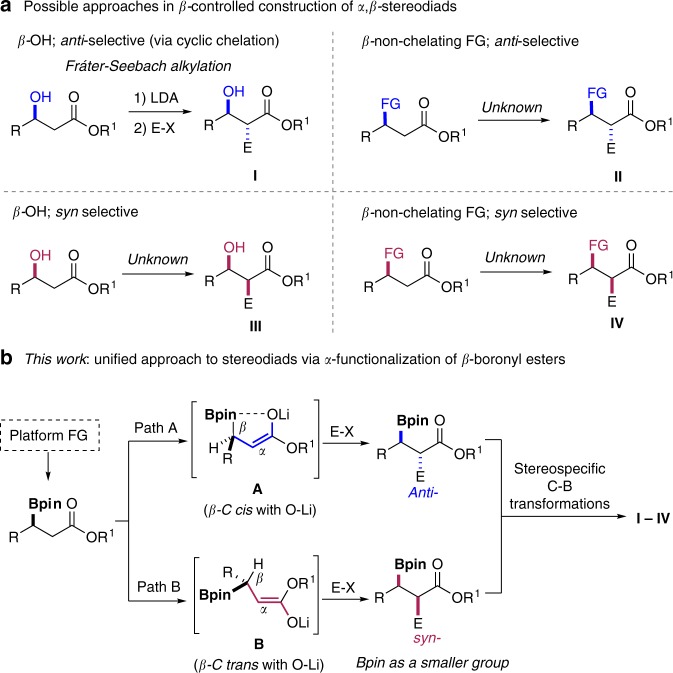
*β*-Controlled *α*-alkylation of carbonyl compounds. **a** Possible strategies in *β*-controlled construction of contiguous *α*,*β*-stereodiads. **b** Boronyl group-controlled unified approach to stereodiads via *α*-functionalization of *β*-boronyl esters.

Introduction of substituents with predictable stereoselectivity is crucial for rational synthetic design of functional molecules. Despite the already advanced toolbox for practitioners of synthetic chemistry, it is still challenging to access any and every stereoisomer of contiguous acyclic stereodiads in a predictable fashion, particularly for substrates that contain only hydrocarbon groups^1–5^. For example, electrophilic *α* alkylation of an enolate is one of the most fundamental textbook transformation.^6^ A venerable variant, Fráter–Seebach alkylation, can reliably produce the desired product with *anti*-selectivity (**I**) starting from *β*-hydroxyl carboxylate (Fig. 1a)^7–9^. A cyclic lithium enolate intermediate involving chelation of the *β*-alkoxide anion is the key stereo-controlling factor. This method has been extensively employed in syntheses of polyketides and other natural products^10,11^. However, the *syn*-product **III** could not be obtained in similar vein (Fig. 1a). Furthermore, when the *β*-substituent is a non-chelating group, usually no stereocontrol could be achieved (Fig. 1a, for **II** and **IV**). Even in the extreme cases where R^1^ could be a chiral auxiliary, it is still challenging to access both diastereomers (**II** and **IV**). Therefore, a unified approach for predictable construction of *syn*- and/or *anti*-stereodiads would be highly desirable yet, to the best of our knowledge, has remained elusive.

Considering that a boronyl group may be readily introduced by recent catalytic asymmetric borylation reactions^12–17^ and could serve as a platform to access other functional groups in a stereospecific fashion^18–26^, we hope to develop a stereodivergent *α*-functionalizations of *β*-boronyl carbonyls with predictable diastereoselectivity employing the unique role of boronyl group (Fig. 1b). The resulting acyclic compounds bearing contiguous tertiary–tertiary or even tertiary–quaternary stereogenic centers should have great value in syntheses of natural products and pharmaceuticals^27–30^.

In the past few years, a number of catalytic approaches to boronyl-containing 1,2-stereodiads have been reported by Miura, Ito, Liao, Brown, and other groups^31–44^. However, usually only one diastereomer rather than the other is preferred, and development of general and reliable methodologies for obtaining all possible stereoisomers of *β*-boron carbonyls remains highly challenging^45–50^. The Ito group^51^ and the Zhong group^52^ have independently reported diastereoselective borylative alkylation of *α*,*β*-unsaturated esters. However, the enantioselective variant has not been accomplished.

Herein, we wish to disclose our results in developing a practical and general, *β*-boronyl and conditions-controlled approach for divergent synthesis of both *syn* and *anti*-products containing contiguous tertiary–tertiary and tertiary–quaternary stereodiads (Fig. 1b). Upon deprotonation, a *cis*-enolate (path A) or a *trans* enolate (path B) may be selectively generated. In the *cis* case, due to the Lewis acidic property of sp^2^ boron atom, by invoking a five-membered chelation mode **A**, the *anti*-selectivity could be realized because the electrophile should attack from the less-hindered face of **A** (Fig. 1b). On the other hand, if the deprotonation follows an Ireland model^53,54^, an open-chain *trans* intermediate **B** could be formed. 1,3-Allylic strain would induce a relatively fixed conformation and the facial selectivity of alkylation should be dictated by the relative size difference of the Bpin and R group (Fig. 1b, path B). Pleasingly, we discover that in all cases, the Bpin group behaves uniquely as a smaller group than R, and therefore the *syn*-product is generally favored.

## Results

### Reaction optimization

We commenced the study by investigating the model reaction between *β*-boronyl esters **1a** and allyl bromide **2**. Simple treatment of a solution of **1a** in THF with LDA (lithium diisopropylamide) followed by addition of **2** at −78 ^°^C led to a mixture of diastereomers favoring *syn*-**4a** in 65% overall yield (*anti/syn* = 1:2.4, Table 1, entry 1). The relative configurations could be confirmed by transformation of the boronates to the corresponding alcohols. Although toluene was not an effective solvent for this reaction, a mixed solvents (THF/toluene = 1:1 v/v) could slightly improve the diastereoselectivity (*anti/syn* = 1:3.9, Table 1, entry 3). In contrast, when hexamethylphosphoramide (HMPA) was used as an additive, the *anti*-product **3a** was obtained with excellent diastereoselectivity (*anti*/*syn* > 20:1, Table 1, entry 4), albeit in low yield (33%). Further, a modification of the ethyl ester **1a** to 3-pentyl ester **1b** markedly enhanced the *syn*-selectivity (Table 1, entry 5, 77% yield, *anti*/*syn* = 1:10). A slightly higher *syn*-selectivity was achieved when some more toluene cosolvent was used (THF/toluene 1:1.5 v/v, Table 1, entry 6). The best conditions for *anti*-product **3** was obtained when *tert*-butyl ester **1c** was employed (Table 1, entry 9, 75% yield, *anti*/*syn* > 20:1).

**Table 1:** Condition optimization for the allylation of *β-*boronyl esters^a^.


Entry	1	Solvent	Additive	Yield (3 + 4) (%)	d.r. (3/4)
1	**1a**	THF	none	65	1:2.4
2	**1a**	Toluene	none	0	–
3	**1a**	THF/Toluene (1:1)	none	69	1:3.9
4	**1a**	THF	HMPA	33	> 20:1
5	**1b**	THF/Toluene (1:1)	none	77	1:10
6	**1b**	THF/Toluene (1:1.5)	none	77	1:11.8
7	**1b**	THF	HMPA	50	> 20:1
8	**1c**	THF/Toluene (1:1)	none	75	1:3.5
9	**1c**	THF	HMPA	75	> 20:1

^a^Reaction conditions: **1** (0.25 mmol), **2** (1.5 equiv), LDA (1.1 equiv), HMPA (0.2 mL, if used) in 1 mL of solvent at −78 °C for 12 h. Yields and diastereoselectivities were determined by ^1^H NMR analysis of the crude reaction mixture with 1,3,5-trimethoxybenzene as an internal standard.

### Substrate scope

**Fig. 2:**
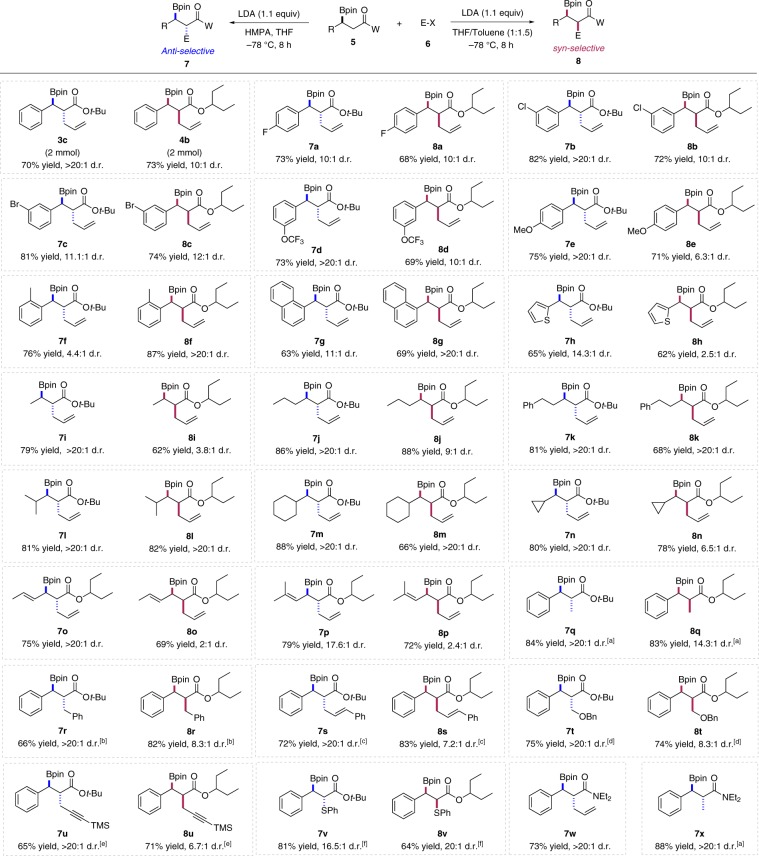
Substrate scope of the diastereodivergent *α*-functionalization of *β*-boronyl carbonyl compounds. Reaction conditions: unless otherwise noted, the reactions were performed at 0.25  mmol scale with 1.5 equiv allyl bromide, 1.1 equiv LDA, and 0.2 mL HMPA (if used). Isolated yields shown. D.r. values determined by ^1^H NMR analysis of crude poducts. ^a^Iodomethane as the electrophile. ^b^Benzyl bromide as the electrophile. ^c^Cinnamyl bromide as the electrophile. ^d^Benzylchloromethyl ether as the electrophile. ^e^3-Bromo-1-(trimethylsilyl)-1-propyne as the electrophile. ^f^Diphenyl disulfide as the electrophile.

**Fig. 3:**
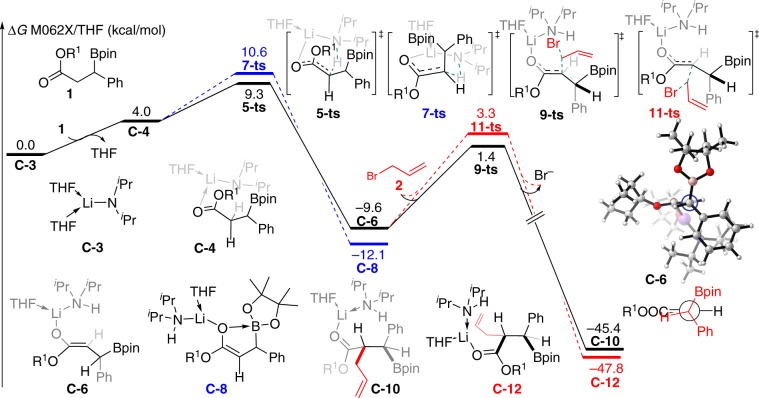
Calculated free energy profiles for the *α*-alkylation of carbonyl compounds (R^1^ = 3-pentyl). The favored pathway is labeled by solid lines. The values given in kcal/mol are the relative free energies calculated by the M06-2X/6-311 + G(d,p)//B3LYP/6-31 G(d) method in THF solvent.

**Fig. 4:**
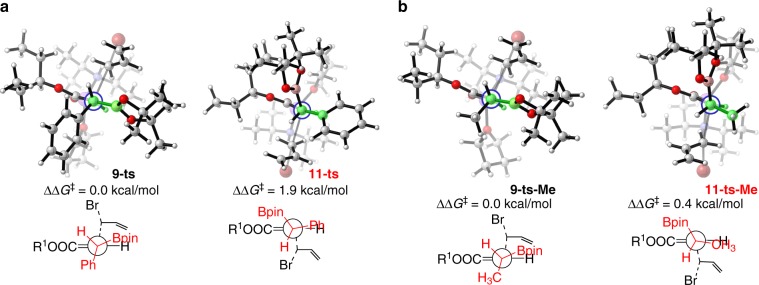
Optimized structures at nucleophilic substitution steps. **a** Transition state of **9-ts** and **11-ts**. **b** Transition state of **9-ts-Me** and **11-ts-Me**. (R^1^ = 3-pentyl).

With optimal reaction conditions established, we explored the scope of these reactions. We found that the diastereodivergent reaction was successfully performed on a 2.0 mmol scale with little effects on the yield and selectivity (Figs. 2, 3c, 4b). As shown in Fig. 2, a variety of *β*-boronyl esters and electrophiles participated in these transformations in good to excellent yields with consistent diastereoselectivity for both *syn* and *anti*-products. For example, halide substituents on the aryl ring were well compatible in the reaction conditions (**7a**–**7c** for *anti* and **8a**–**8c** for *syn*-products). The aromatic ring containing methoxy or trifluoromethoxy group proved to be competent reaction partners (**7d**, **7e**, **8d**, **8e**). It is noteworthy that, substrates with sterically demanding groups furnished the *syn*-products with higher diastereoselectivity when compared with the phenyl group (**8f**, **8g** versus **4b**), which were consistent with our observation that Bpin served as the smaller group. The substrate bearing 2-thiophene furnished the *syn*-product with lower diastereoselectivity (**8h**, 62% yield, *anti*/*syn* = 1:2.5). This could be understood because the steric hindrance of 2-thiophenyl group should be smaller than phenyl group. Nevertheless, the *anti*-product **7** **h** was obtained with high diastereoselectivity. We next examined the viability of substrates with aliphatic substituents. To our surprise, the *syn*-product **8i** could still be obtained with moderate selectivity (*anti/syn* = 1:3.8), which means that Bpin behaves as a smaller group even than methyl group. As expected, the more bulkier alkyl group was employed, the higher *syn*-selectivity could be achieved (**8j**–**8n**). These results all pointed that Bpin acted as a smaller group in this transformation. In parallel, these *β*-boronyl esters containing aliphatic substituents successfully furnished the *anti*-products **7i**–**7n** with excellent diastereoselectivity. In addition, alkenyl-substituted *β*-boronyl esters also participated in this reaction and generated the corresponding products **7o**–**7p** and **8o**–**8p** with selectivity of same trend.

We also examined the scope with respect to other electrophiles. Beyond allyl substituent, methyl, benzyl, cinnamyl, benzyloxymethyl, and propargyl groups could also be installed to the *β*-boronyl esters with high selectivity for both *syn* and *anti*-products (**7q**–**7u** and **8q**–**8u**). Besides alkyls, the phenylthio group could also be introduced to the *α*-position of *β*-boronyl esters with excellent diastereoselectivity when diphenyl disulfide was employed as the electrophile (**7v**, **8v**). Finally, we tested similar alkylation of *β*-boronyl amides. However, only *anti*-products could be accessed under either set of conditions (**7w**–**7x**), probably because of the severe steric interaction between NEt_2_ and *β*-carbon in the Ireland model, leading to exclusive formation of *cis*-enolate^55^.

### Mechanistic origins of the *syn*-selectivity

Density functional M06-2X^56^ with a standard 6-311 + G(d,p) basis set was employed to gain further insight into the diastereodivergent alkylation reactions, paticularly the general *syn*-selective outcome. In our theoretical calculations, the *β*-boryl ester **1** and allyl bromide **2** were chosen as reactants in model reaction, which can give *syn*-product **4b** in experimental observations (Fig. 3). As depicted in Fig. 3, ligand exchange between solvent-coordinated LDA **C-3** and *β*-boryl ester **1** forms O-coordinated complex **C-4** in an endothermic process with 4.0 kcal/mol free energy, which can attribute to the weak coordination ability of ester. In complex **C-4**, the coordination onto lithium significantly increased the ester’s *α*-acidity. Subsequently, amino-assisted deprotonation of ester’s *α*-hydrogen can smoothly occur via either six-membered ring transition state **5-ts** or **7-ts**. The calculated relative free energy of **7-ts** is 1.3 kcal/mol higher than that of **5-ts**, indicating that the generation of open-chain enolate **C-6** is favorable with 13.6 kcal/mol exergonic. The energy difference between transition states **5-ts** and **7-ts** can be attributed to the position of bulky boronylbenzyl group, which is located at the axial position in **7-ts**. In this case, 1,3-allylic strain resulted in a lowest energy conformation, where the *β*-proton and the bulky ester group OR^1^ of enolate **C-6** are *cis* to each other and nearly coplanar^57^. The subsequent intermolecular nucleophilic substitution by enolate **C-6** with allyl bromide **2** could occur through two different transition states owing to the chiral environment of *β*-C in enolate **C-6**. The intermolecular nucleophilic substitution by the *si*-face of enolate **C-6** onto the allyl bromide **2** could occur via linear transition state **9-ts**, which leads to the generation of *syn*-product **4b** from intermediate **C-10**. On the contrary, the *anti*-isomer might be obtained via transition state **11-ts**, where the nucleophilic substitution could take place by the *re*-face of enolate **C-6**. The calculated energy barrier of transition state **11-ts** is 12.9 kcal/mol, which is 1.9 kcal/mol higher than that of **9-ts**. Therefore, the computational study depicted that the *syn*-**4b** should be the major product, which is consistent with experimental observations. As shown in Fig. 4a, optimized structure analysis shows that the main difference between **9-ts** and **11-ts** is the conformation of the *α*- and *β*-carbon atoms. To the satisfaction of the minimum steric repulsion of coming allyl bromide in nucleophilic substitution, the *β*-phenyl group and *α*-hydrogen adopt an eclipsed conformation in higher free energy transition state **11-ts**, while, the *β*-boryl and *α*-hydrogen are eclipsed in transition state **9-ts**. The phenyl group is larger than that of boryl group, which causes larger steric repulsion between eclipsed *β*-phenyl group and *α*-hydrogen in transition state **11-ts**. Based on the aforementioned theoretical results, we hypothesized when the bulky phenyl group is masked by a smaller methyl group, poor diastereoselectivity will be observed in the experiment. As shown in Fig. 4b, in the nucleophilic substitution step, the calculated relative free energy of the *re*-face attack transition state **11-ts-Me** is only 0.4 kcal/mol higher than that of *si*-face attack transition state **9-ts-Me**. The theoretically calculated diastereoselectivity value for the methyl masked reaction would decrease to 2.8:1, which is consistent with the experimental observation value (3.8:1). Although the Bpin group contains a five-membered heterocycle and a bulky pinacol moiety, its planar structure combined with substituent-free oxygen atoms might render it a less sterically demanding structural unit than common carbon-centered groups. In a recent report, Tillin et al. also noted smaller size effect of Bpin in comparison with groups such as cyclohexyl^58^.

### Construction of tertiary–quaternary stereodiads

**Fig. 5:**
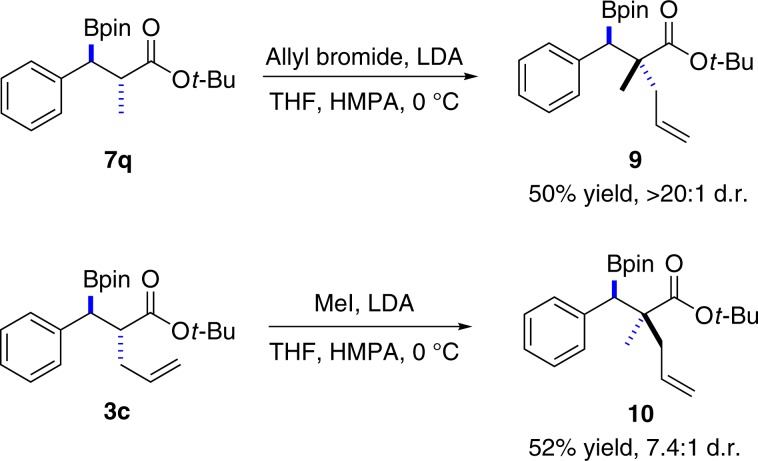
Construction of tertiary–quaternary stereocenters. Preparation of the diastereoisomers **9** and **10**.

The construction of quaternary carbon stereocenters, especially in an open-chain molecule, is usually a challenge in synthetic chemistry^59^. Encouraged by the above results, we proceeded with a second *α*-alkylation of the obtained products **7q** and **3c** under the standard *anti*-selective conditions. Pleasingly, the tertiary–quaternary diastereoisomers **9** and **10** were obtained with high stereoselectivity (Fig. 5). Therefore, all diastereoisomers of the tertiary–quaternary stereocenters could be obtained by simply changing the sequence of *α*-alkylation. In combination with the well-established enantioselective 1,4-borylation methods^12–17^, all possible stereoisomers could be accessed in such an approach.

### Stereospecific C-B transformations

**Fig. 6:**
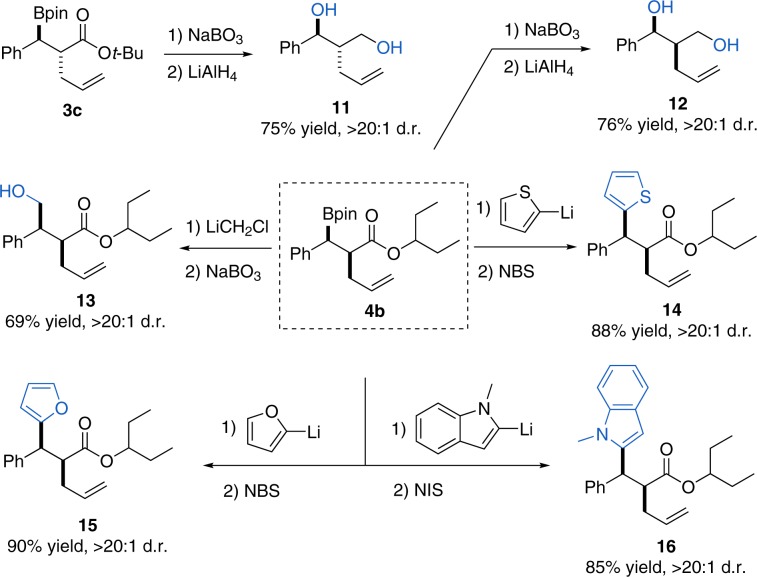
Products Transformations. Transformations of the boronic ester to alcohols (**11** and **12**), hydroxymethyl (**13**), and heterocycles (**14**, **15,** and **16**).

To exemplify the versatility of this methodology in building contiguous stereodiads, we converted the organoboronic acid derivatives into other classes of compounds by using established methods (Fig. 6). For example, the diastereoisomeric diols **11** and **12** could be accessed easily by an oxidation–reduction sequence from **3c** and **4b**, respectively. Moreover, the hydroxymethylated product **13** could be prepared by using the in situ formed LiCH_2_Cl and subsequent oxidation with NaBO_3_^60^. Furthermore, the coupling of 2-lithiofuran with boronic ester **4b** gave the arylated product **14** in high yield with complete stereoretention by using the protocol developed by the Aggarwal group^61^. Similarly, other heterocycles such as indole and furan groups could be installed without erosion in diastereomeric ratio. These 1,1-diaryl compounds are important pharmacophores, and their stereocontrolled preparation have been challenging through traditional protocols^62–65^.

**Fig. 7:**
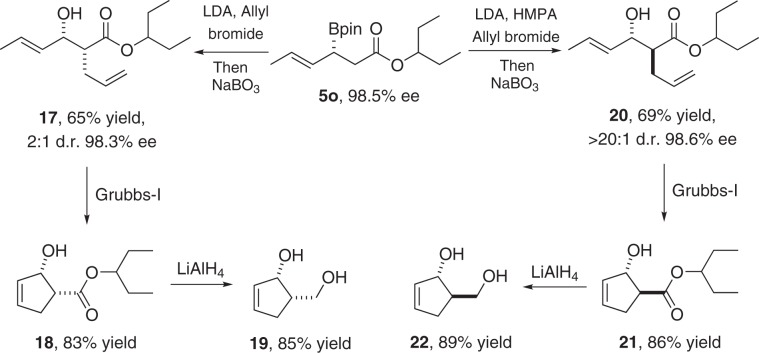
Synthetic utility of the divergent methodology. Unified enantioselective synthesis of *cis* and *trans* diols **19** and **22**.

In our previous work, the *cis*-diol **19**, a key intermediate of carbocyclic nucleosides^66^, was successfully employed for total synthesis of prostratin, a potent anti-HIV and antitumor natural product^67^. The *trans*-diol **22** is also an important synthetic intermediate of carbocyclic analogs of the antiviral ribavirin^68^. Evans aldol reaction was previously used for the synthesis of these diols. However, the use of stoichiometric amounts of chiral auxiliaries, excess expensive and sensitive Lewis acid *n*-Bu_2_BOTf limited the synthetic efficiency. Herein, based on the current approach, unified catalytic enantioselective syntheses of *cis*-diol **19** and *trans*-diol **22** can be achieved using inexpensive chmicals (Fig. 7). Optically pure **5o** was obtained through Cu-catalyzed enantioselective 1,4-borylation of the sorbic acid-derived ester^69,70^. Diastereoselective *α*-allylation of chiral **5o** with standard conditions and subsequent oxidation with NaBO_3_ furnished **17** and **20** in good yields. The *cis*-diol **19** and *trans*-diol **22** were then obtained by ring-closing olefin metathesis of **17** and **20** followed by reduction with LiAlH_4_, respectively.

In conclusion, we have developed a predictable and unified protocol for both *syn* and *anti* diastereoselective *α*-functionalization of readily available *β*-boronyl carbonyls. The key to success was the exploitation of the dual roles of Bpin: its apparently small size and its Lewis acidic character. The structural predictability, operational simplicity, and broad substrate scope in combination with the versatile role of Bpin in synthetic transformations should render the method useful in preparation of contiguous diads, which are present in many types of biologically active molecules yet difficult to access by conventional approaches. The surprising size effect of a boronyl group may well be used in other parts of organoboron chemistry.

## Methods

### General procedure for *anti α*-functionalized-*β*-boronyl esters

A solution of diisopropylamine (0.275 mmol, 38.5 µL) in THF (0.5 mL) was cooled to 0 °C and treated with *n*-BuLi (110 µL, 2.5 M in hexane) dropwise. The reaction mixture was stirred for 15 min and cooled to −78 °C. To the freshly prepared solution of LDA, a solution was added of *β*-boronyl ester (0.25 mmol) in THF/HMPA (0.5 ml/0.2 ml) dropwise over 2 min. After stirring at the same temperature for 3 h, the electrophile (0.375 mmol) was added to the reaction mixture dropwise over 1 min and stirred for 8 h. Then the reaction was quenched with saturated aq. NH_4_Cl solution (1.0 mL) and diluted with DCM (5.0 mL). The layers were separated, the aqueous phase was extracted with DCM (2 × 5.0 mL), the combined organic phases were washed with water and brine, dried (MgSO_4_) and concentrated in vacuo. The crude product was purified by flash column chromatography to yield the desired *anti*-product.

### General procedure for *syn α*-functionalized-*β*-boronyl esters

A solution of diisopropylamine (0.275 mmol, 38.5 µL) in toluene (0.6 mL) was cooled to 0 °C and treated with *n*-BuLi (110 µL, 2.5 M in hexane) dropwise. The reaction mixture was stirred for 15 min and cooled to −78 °C. To the freshly prepared solution of LDA, a solution was added of *β*-boronyl ester (0.25 mmol) in THF (0.4 mL) dropwise over 2 min. After stirring at the same temperature for 15 min, the electrophile (0.375 mmol) was added to the reaction mixture dropwise over 1 min and stirred for 8 h. Then the reaction was quenched with saturated aq. NH_4_Cl solution (1.0 mL) and diluted with DCM (5.0 mL). The layers were separated, the aqueous phase was extracted with DCM (2 × 5.0 mL), the combined organic phases were washed with water and brine, dried (MgSO_4_) and concentrated in vacuo. The crude product was purified by flash column chromatography to yield the desired *syn*-product.

### Spectroscopic methods

^1^H NMR and ^13^C NMR spectra were recorded using Bruker Avance 400 MHz spectrometers. High-resolution mass spectra (HRMS) were obtained on a WATERS I-Class VION IMS QTof spectrometer. Enantiomeric excesses (ee) were determined by chiral HPLC analysis using Waters 2489 Series chromatographs using a mixture of HPLC-grade hexane and isopropanol as eluent.

## Supplementary information

Supplementary Information

Description of Additional Supplementary Files

Supplementary Data 1

## Data Availability

The authors declare that the data supporting the findings of this study are available within the article and Supplementary Information file.

## References

[1] Krautwald S, Sarlah D, Schafroth MA, Carreira EM (2013). Enantio- and diastereodivergent dual catalysis: α-allylation of branched aldehydes. Science.

[2] Shi S-L, Wong ZL, Buchwald SL (2016). Copper-catalysed enantioselective stereodivergent synthesis of amino alcohols. Nature.

[3] Kaldre D, Klose I, Maulide N (2018). Stereodivergent synthesis of 1,4-dicarbonyls by traceless charge–accelerated sulfonium rearrangement. Science.

[4] Trost BM, Hung C-I, Saget T, Gnanamani E (2018). Branched aldehydes as linchpins for the enantioselective and stereodivergent synthesis of 1,3-aminoalcohols featuring a quaternary stereocentre. Nat. Catal..

[5] Bruffaerts J, Pierrot D, Marek I (2018). Efficient and stereodivergent synthesis of unsaturated acyclic fragments bearing contiguous stereogenic elements. Nat. Chem..

[6] Carey, F. A. & Sundberg, R. J. Alkylation of Enolates and Other Carbon Nucleophiles. In: Carey, F. A. & Sundberg, R. J. (eds) *Advanced organic chemistry, part B: reactions and synthesis*, 5th edn, 1–62 (Springer, New York, 2007).

[7] Fráter G (1979). Über die stereospezifität der α-alkylierung von β-hydroxycarbonsäureestern. Vorläufige Mitteilung. Helv. Chim. Acta.

[8] Seebach D, Wasmuth D (1980). Herstellung von erythro-2-hydroxybernsteinsäure-derivaten aus äpfelsäureester. vorläufige mitteilung. Helv. Chim. Acta.

[9] Li, J. J. Frater–Seebach alkylation. In: Li, J. J. (ed.) *Name reactions: a collection of detailed reaction mechanisms*, 2nd edn, 127 (Springer-Verlag, Berlin Heidelberg GmbH, New York, 2003).

[10] Barth R, Mulzer J (2007). Total synthesis of efomycine M. Angew. Chem., Int. Ed..

[11] Crimmins MT, O’Bryan EA (2010). Enantioselective total synthesis of spirofungins A and B. Org. Lett..

[12] Lee J-E, Yun J (2008). Catalytic asymmetric boration of acyclic α,β-unsaturated esters and nitriles. Angew. Chem., Int. Ed..

[13] Zheng K, Liu X, Feng X (2018). Recent advances in metal-catalyzed asymmetric 1,4-conjugate addition (ACA) of nonorganometallic nucleophiles. Chem. Rev..

[14] Hornillos V, Vila C, Otten E, Feringa BL (2015). Catalytic asymmetric synthesis of phosphine boronates. Angew. Chem., Int. Ed..

[15] O’Brien JM, Lee K-s, Hoveyda AH (2010). Enantioselective synthesis of boron-substituted quaternary carbons by NHC−Cu-catalyzed boronate conjugate additions to unsaturated carboxylic esters, ketones, or thioesters. J. Am. Chem. Soc..

[16] Zhu L, Kitanosono T, Xu P, Kobayashi S (2015). A Cu(II)-based strategy for catalytic enantioselective β-borylation of α,β-unsaturated acceptors. Chem. Commun..

[17] Wu H, Radomkit S, O’Brien JM, Hoveyda AH (2012). Metal-free catalytic enantioselective C–B bond formation: (pinacolato)boron conjugate additions to α,β-unsaturated ketones, esters, weinreb amides, and aldehydes promoted by chiral N-heterocyclic carbenes. J. Am. Chem. Soc..

[18] Sandford C, Aggarwal VK (2017). Stereospecific functionalizations and transformations of secondary and tertiary boronic esters. Chem. Commun..

[19] García-Ruiz C (2017). Stereospecific allylic functionalization: the reactions of allylboronate complexes with electrophiles. J. Am. Chem. Soc..

[20] Rygus JPG, Crudden CM (2017). Enantiospecific and iterative Suzuki–Miyaura cross-couplings. J. Am. Chem. Soc..

[21] Zhao S (2018). Enantiodivergent Pd-catalyzed C–C bond formation enabled through ligand parameterization. Science.

[22] Ding S, Xu L, Li P (2016). Copper-catalyzed boron-selective C(sp2)–C(sp3) oxidative cross-coupling of arylboronic acids and alkyltrifluoroborates involving a single-electron transmetalation process. ACS Catal..

[23] Wang G (2017). N,B-bidentate boryl ligand-supported iridium catalyst for efficient functional-group-directed C–H borylation. J. Am. Chem. Soc..

[24] Xu L, Zhang S, Li P (2015). Boron-selective reactions as powerful tools for modular synthesis of diverse complex molecules. Chem. Soc. Rev..

[25] Fyfe JWB, Watson AJB (2017). Recent developments in organoboron chemistry: old dogs, new tricks. Chem.

[26] Leonori D, Aggarwal VK (2014). Lithiation–borylation methodology and its application in synthesis. Acc. Chem. Res..

[27] Surleraux DLNG (2005). Discovery and selection of TMC114, a next generation HIV-1 protease inhibitor. J. Med. Chem..

[28] Kobayashi M (1980). Bioorganic synthesis and absolute configuration of faranal. J. Am. Chem. Soc..

[29] Fujisawa T (2002). Highly water-soluble matrix metalloproteinases inhibitors and their effects in a rat adjuvant-induced arthritis model. Bioorg. Med. Chem..

[30] Tzschentke TM (2007). (–)-(1*R*, 2*R*)-3-(3-Dimethylamino-1-ethyl-2-methyl-propyl)-phenol hydrochloride (tapentadol HCl): a novel μ-opioid receptor agonist/norepinephrine reuptake inhibitor with broad-spectrum analgesic properties. J. Pharmacol. Exp. Ther..

[31] Matsuda N, Hirano K, Satoh T, Miura M (2013). Regioselective and stereospecific copper-catalyzed aminoboration of styrenes with bis(pinacolato)diboron and O-benzoyl-N,N-dialkylhydroxylamines. J. Am. Chem. Soc..

[32] Kubota K, Watanabe Y, Hayama K, Ito H (2016). Enantioselective synthesis of chiral piperidines via the stepwise dearomatization/borylation of pyridines. J. Am. Chem. Soc..

[33] Jiang L (2016). Highly diastereo- and enantioselective Cu-catalyzed borylative coupling of 1,3-dienes and aldimines. Angew. Chem., Int. Ed..

[34] Logan KM, Sardini SR, White SD, Brown MK (2018). Nickel-catalyzed stereoselective arylboration of unactivated alkenes. J. Am. Chem. Soc..

[35] Itoh T, Kanzaki Y, Shimizu Y, Kanai M (2018). Copper(I)-catalyzed enantio- and diastereodivergent borylative coupling of styrenes and imines. Angew. Chem., Int. Ed..

[36] Morgan JB, Miller SP, Morken JP (2003). Rhodium-catalyzed enantioselective diboration of simple alkenes. J. Am. Chem. Soc..

[37] Meng F, Haeffner F, Hoveyda AH (2014). Diastereo- and enantioselective reactions of bis(pinacolato)diboron, 1,3-enynes, and aldehydes catalyzed by an easily accessible bisphosphine–Cu complex. J. Am. Chem. Soc..

[38] Logan KM, Smith KB, Brown MK (2015). Copper/palladium synergistic catalysis for the syn- and anti-selective carboboration of alkenes. Angew. Chem., Int. Ed..

[39] Kato K, Hirano K, Miura M (2016). Synthesis of β-boryl-α-aminosilanes by copper-catalyzed aminoboration of vinylsilanes. Angew. Chem., Int. Ed..

[40] Ito H, Horita Y, Yamamoto E (2012). Potassium tert-butoxide-mediated regioselective silaboration of aromatic alkenes. Chem. Commun..

[41] Liu Z, Li X, Zeng T, Engle KM (2019). Directed, palladium(II)-catalyzed enantioselective anti-carboboration of alkenyl carbonyl compounds. ACS Catal..

[42] Feng J-J, Oestreich M (2019). Tertiary α-silyl alcohols by diastereoselective coupling of 1,3-dienes and acylsilanes initiated by enantioselective copper-catalyzed borylation. Angew. Chem., Int. Ed..

[43] Chen B, Cao P, Liao Y, Wang M, Liao J (2018). Enantioselective copper-catalyzed methylboration of alkenes. Org. Lett..

[44] Wang H-M (2018). Copper-catalyzed borylative cyclization of substituted N-(2-Vinylaryl)benzaldimines. Org. Lett..

[45] He Z-T (2014). Copper-catalyzed asymmetric hydroboration of α-dehydroamino acid derivatives: facile synthesis of chiral β-hydroxy-α-amino acids. Org. Lett..

[46] Xie J-B, Lin S, Qiao S, Li G (2016). Asymmetric catalytic enantio- and diastereoselective boron conjugate addition reactions of α-functionalized α,β-unsaturated carbonyl substrates. Org. Lett..

[47] Lillo V (2009). Asymmetric β-boration of α,β-unsaturated esters with chiral (NHC)Cu catalysts. Organometallics.

[48] Kubota K, Hayama K, Iwamoto H, Ito H (2015). Enantioselective borylative dearomatization of indoles through copper(I) catalysis. Angew. Chem., Int. Ed..

[49] Chen L, Shen J-J, Gao Q, Xu S (2018). Synthesis of cyclic chiral α-amino boronates by copper-catalyzed asymmetric dearomative borylation of indoles. Chem. Sci..

[50] Bi Y-P (2019). Stereoselective synthesis of all-cis boryl tetrahydroquinolines via copper-catalyzed regioselective addition/cyclization of o-aldiminyl cinnamate with B_2_Pin_2_. Org. Biomol. Chem..

[51] Hayama K, Kubota K, Iwamoto H, Ito H (2017). Copper(I)-catalyzed diastereoselective dearomative carboborylation of indoles. Chem. Lett..

[52] Zuo Y-J (2018). Copper-catalyzed diastereoselective synthesis of β-boryl-α-quaternary carbon carboxylic esters. Org. Biomol. Chem..

[53] Ireland RE, Mueller RH, Willard AK (1976). The ester enolate Claisen rearrangement. Stereochemical control through stereoselective enolate formation. J. Am. Chem. Soc..

[54] Ireland RE, Wipf P, Armstrong JD (1991). Stereochemical control in the ester enolate Claisen rearrangement. 1. Stereoselectivity in silyl ketene acetal formation. J. Org. Chem..

[55] Xie L, Isenberger KM, Held G, Dahl LM (1997). Highly stereoselective kinetic enolate formation: steric vs electronic effects. J. Org. Chem..

[56] Zhao Y, Truhlar DG (2008). The M06 suite of density functionals for main group thermochemistry, thermochemical kinetics, noncovalent interactions, excited states, and transition elements: two new functionals and systematic testing of four M06-class functionals and 12 other functionals. Theor. Chem. Acc..

[57] Evans, D. A. Stereoselective alkylation reactions of chiral metal enolates. In *Asymmetric Synthesis* Vol. 3 (ed. Morrison, J. D.) 2–110 (Academic Press, Orlando, 1984).

[58] Tillin C (2019). Complex boron-containing molecules through a 1,2-metalate rearrangement/anti-sn2′ elimination/cycloaddition reaction sequence. Synlett.

[59] Feng J, Holmes M, Krische MJ (2017). Acyclic quaternary carbon stereocenters via enantioselective transition metal catalysis. Chem. Rev..

[60] Chen A, Ren L, Crudden CM (1999). Catalytic asymmetric hydrocarboxylation and hydrohydroxymethylation. A two-step approach to the enantioselective functionalization of vinylarenes. J. Org. Chem..

[61] Bonet A, Odachowski M, Leonori D, Essafi S, Aggarwal VK (2014). Enantiospecific sp^2^–sp^3^ coupling of secondary and tertiary boronic esters. Nat. Chem..

[62] Ameen D, Snape TJ (2013). Chiral 1,1-diaryl compounds as important pharmacophores. MedChemComm.

[63] Caruana L, Kniep F, Johansen TK, Poulsen PH, Jørgensen KA (2014). A new organocatalytic concept for asymmetric α-alkylation of aldehydes. J. Am. Chem. Soc..

[64] Stadler D, Bach T (2008). Highly diastereoselective Friedel–Crafts alkylation reactions via chiral α-functionalized benzylic carbocations. Chem. Asian J..

[65] Rubenbauer P, Bach T (2008). Gold(III) chloride-catalyzed diastereoselective alkylation reactions with chiral benzylic acetates. Adv. Synth. Catal..

[66] Crimmins MT, King BW, Zuercher WJ, Choy AL (2000). An efficient, general asymmetric synthesis of carbocyclic nucleosides: application of an asymmetric aldol/ring-closing metathesis strategy. J. Org. Chem..

[67] Tong G, Liu Z, Li P (2018). Total synthesis of (±)-prostratin. Chem.

[68] Kuang R (2000). Enantioselective syntheses of carbocyclic ribavirin and its analogs: linear versus convergent approaches. Tetrahedron Lett..

[69] Kitanosono T, Xu P, Kobayashi S (2013). Heterogeneous and homogeneous chiral Cu(II) catalysis in water: enantioselective boron conjugate additions to dienones and dienoesters. Chem. Commun..

[70] Kobayashi S, Xu P, Endo T, Ueno M, Kitanosono T (2012). Chiral copper(II)-catalyzed enantioselective boron conjugate additions to α,β-unsaturated carbonyl compounds in water. Angew. Chem., Int. Ed..

