# Supplementary material for: A unified approach for divergent synthesis of contiguous stereodiads employing a small boronyl group
Source: Nat Commun. 2020 Feb 7;11:792. doi: 10.1038/s41467-020-14592-7 (PMC7005891; doi:10.1038/s41467-020-14592-7)
Supplement: Supplementary file 1 — Supplementary Information [file 41467_2020_14592_MOESM1_ESM.pdf]

Supplementary Information for

A unified approach for divergent synthesis of contiguous stereodiads employing a small boronyl group

Zhan et al.

Supplementary Methods

General experimental methods

Commercial reagents were purchased from Adamas, J&K, Acros Organics, Energy, Sigma-Aldrich, Alfa Aesar, or TCI and used as received unless otherwise noted. THF and toluene were purified by distillation over sodium/benzophenone and stored under argon. n-BuLi was purchased from Acros Organics and used directly. HMPA and diisopropylamine were distilled over CaH_2. All air-sensitive manipulations were carried out under an argon atmosphere in a glovebox or by standard Schlenk techniques. Glassware was dried at 120 ^{o}C for at least 2 h prior to use.

NMR spectra were recorded using Bruker Avance 400 MHz spectrometers and were referenced to $CHCl_3$ (7.27 and 77.16 ppm for ^{1}H and ^{13}C respectively). The data are reported as follows: chemical shift in ppm on the δ scale, multiplicity (s = singlet, d = doublet, t = triplet, q = quartet, m = multiplet, dd = doublet of doublet, etc.), coupling constants (Hz), and integration. High resolution mass spectra (HRMS) were obtained on a WATERS I-Class VION IMS QTof spectrometer. Optical rotations were obtained on a Anton-paar polarimeter; concentration (c) is in g/100 mL. Melting points are reported with a Hanon MP300 or Shanghai YiCe WRX-4 apparatus. Flash column chromatography was undertaken on silica gel (200-300 mesh). Enantiomeric excesses (ee) were determined by chiral HPLC analysis using Waters 2489 Series chromatographs using a mixture of HPLC-grade hexane and isopropanol as eluent. All of *α,β*-unsaturated carbonyl substrates were prepared according to the literature procedure.[1,2]

Computational Methods

All the DFT calculations were carried out with the GAUSSIAN 09 series of programs[3]. DFT method B3-LYP[4,5] with a standard 6–31G(d) basis set was used for geometry optimizations. Harmonic vibrational frequency calculations were performed for all of the stationary points to confirm them as a local minima or transition structures, and to derive the thermochemical corrections for the enthalpies and free energies. The M06-2X functional in combination with the 6–311+G(d,p) basis set was used to calculate the solvation single point energies to give more accurate energy information. The solvent effects were considered by single point calculations on the gas-phase stationary points with an SMD[6] solvation model in the THF solvent.

Supplementary Table 1. Supplementary results for the condition optimization

Supplimentary Results for Condition Optimization for the Allylation of β-Boronyl Esters[a]

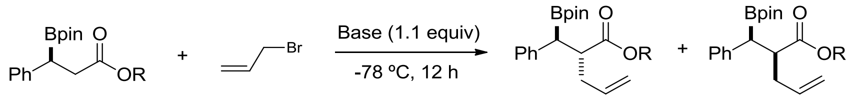

1 (±) **2** (1.5 equiv) **3** **4**

1a: R = Ethyl
1b: R = 3-pentyl

entry	1	Base	solvent	additive	Yield (**3**+**4**) (%)	d.r. (**3**/**4**)
1	1a	LiHMDS	THF	none	<10	–
2	1a	NaHMDS	THF	none	<10	–
3	1a	KHMDS	THF	none	<10	–
4	1a	LTMP	THF	none	71	1:2.5
5	1b	LTMP	THF/Toluene (1:1)	none	75	1:5.6
6	1b	LDA	THF	DMPU	25	1:1
7	1b	LDA	THF	NMP	0	–

[a]Reaction conditions: **1** (0.25 mmol), **2** (1.5 equiv), Base (1.1 equiv), additive (0.2 mL, if used) in 1 mL of solvent at -78 ℃ for 12 h. Yields and diastereoselectivities were determined by ^{1}H NMR analysis of the crude reaction mixture with 1,3,5-trimethoxybenzene as an internal standard.

Supplementary Table 2. Diasetreoselective allylation of substrate with a larger boronyl group

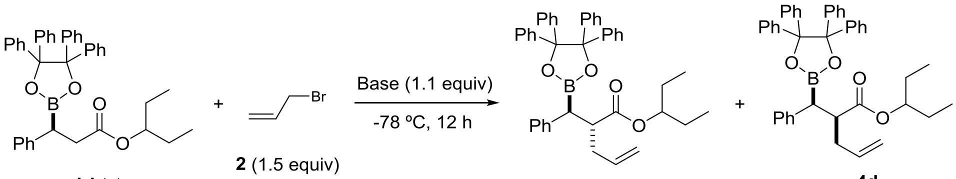

1d (±) **2** (1.5 equiv) **3d** **4d**

Base	Solvent	Additive	Yield (**3d**+**4d**) (%)	d.r. (**3d**/**4d**)
LDA	THF	none	92	1:4.3
LDA	THF	HMPA	57	7.1:1

Reaction conditions: **1d** (0.25 mmol), **2** (1.5 equiv), Base (1.1 equiv), additive (0.2 mL, if used) in 1 mL of solvent at -78 ℃ for 12 h. Yields and diastereoselectivities were determined by ^{1}H NMR analysis of the crude reaction mixture with 1,3,5-trimethoxybenzene as an internal standard.

Experimental and DFT calculation of the enolate intermediates

The lithium enolate intermediates were trapped by TMSCl to observe the *trans/cis* ratios for different substrates under the *syn*-selective deprotonation conditions. After deprotonation at -78 °C, TMSCl was added and the mixture was stirred for 30 min to 2 h at -78 °C. The solvents were removed under high vacuum and the resulting residue was directly analyzed by ^{1}H NMR. The ratios of trapped enolates were sensitive to the workup procedures and fluctuate to certain extents. When substrate **1b** (R = Ph) was used, the ratio of enolate isomers was >20:1~3.99:1 (*trans*:*cis*, Supplementary Figures 206-208). When substrate

5i (R = Me) was employed, the ratio of enolate isomers was 7.7:1~5.3:1 (*trans*:*cis*, Supplementary Figures 209 and 210). The deprotonation of substrate **5i** was also studied by DFT calculations (Supplementary Figure 1). The results showed that the calculated relative free energy of **7-ts-Me** is 2.0 kcal/mol higher than that of **5-ts-Me**, indicating that the generation of open-chain enolate **C-6-Me** is more favorable. By comparison, the calculated relative free energy of **7-ts** is 1.3 kcal/mol higher than **5-ts** (substrate **1b**).

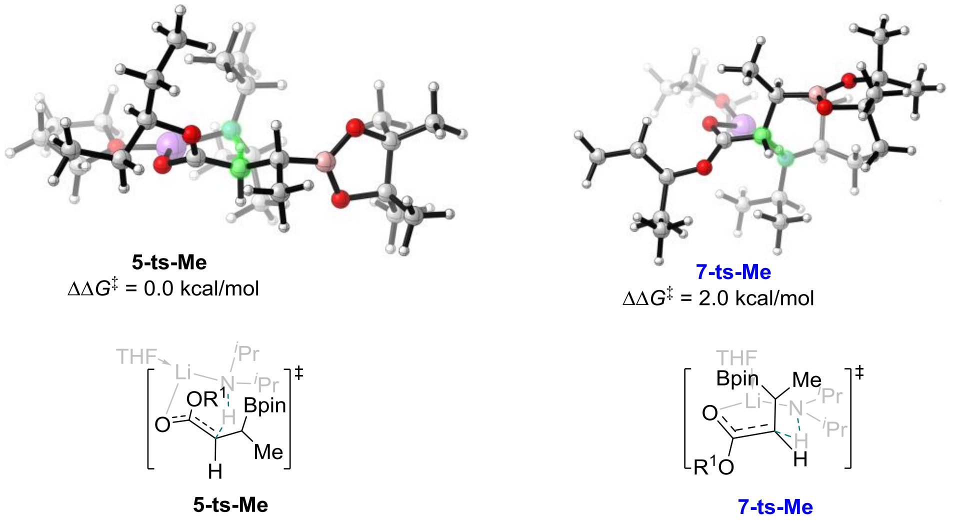

Supplementary Figure 1. Optimized structures at deprotonation steps (**5i**, R = Me, R[1] = 3-pentyl)

Optimized structures of more *trans*-enolate (R^1 = 3-pentyl)

Density functional M06-2X with a standard 6-311+G(d,p) basis set was employed to study the whole diastereodivergent alkylation process. The free energy profile of the *trans*-enolate was calculated and fully optimized. According to the calculation results, the *trans*-enolate with R = Ph, R = Me, R = Cyclohexyl and R = Thienyl gave similar conformation showing that *β*-H is *cis* and almost coplanar with the large -$OCHEt_2$ group.

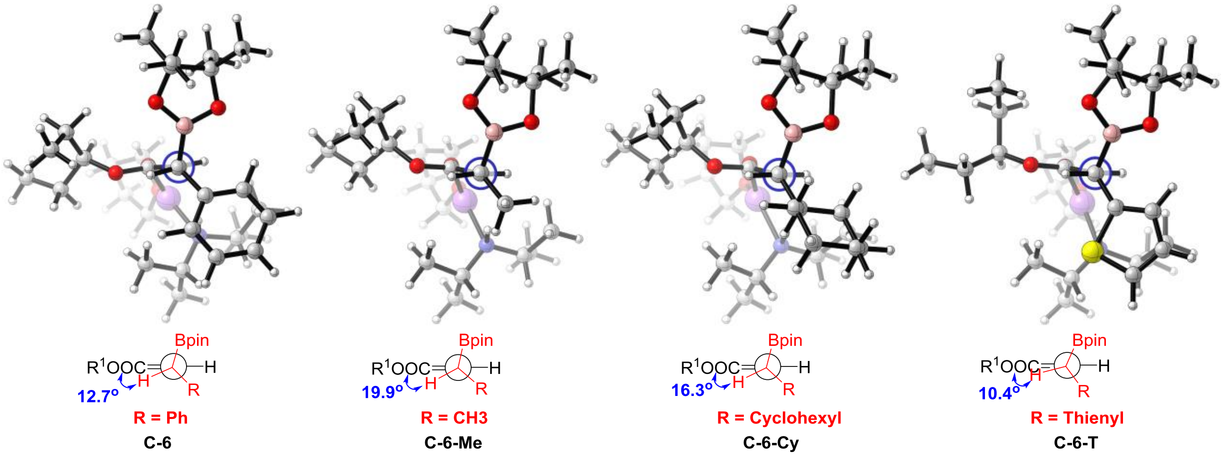

Supplementary Figure 2. Optimized structures of several *trans*-enolates (R[1] = 3-pentyl)

General Procedures for the borylation of *α*,*β*-unsaturated carbonyl compounds.

General Procedure A: Copper-catalyzed borylation of *α,β*-unsaturated esters.[7]

In a oven-dried Schlenk tube were placed CuCl (0.06 mmol, 6.0 mg), NaO*t*-Bu (0.18 mmol, 18.0 mg) and DPEphos ligand (0.06 mmol, 32.4 mg). THF (1.6 mL) was added under argon. The reaction mixture was stirred for 30 min at room temperature, and then bis(pinacolato)diboron (2.20 mmol, 564 mg) in THF (1.2 mL) was added. The reaction mixture was stirred for 30 min and then *a,ß*-unsaturated ester (2 mmol) in THF (1.2 mL) was added, followed by MeOH (4.0 mmol, 160 μL). The reaction tube was washed with THF (5 mL), sealed, and stirred until no starting material was detected by TLC. The reaction mixture was filtered through a pad of celite and concentrated. The product was purified by silica gel chromatography.

General Procedure B: Copper-catalyzed borylation of *α,β*-unsaturated amides[8]

In a oven-dried Schlenk tube were placed CuCl (0.06 mmol, 6.0 mg), NaO*t*-Bu (0.18 mmol, 18.0 mg) and DPPP ligand (0.06 mmol, 24.7 mg). THF (1.6 mL) was added under argon. The reaction mixture was stirred for 30 min at room temperature, and then bis(pinacolato)diboron (2.20 mmol, 564 mg) in THF (1.2 mL) was added. The reaction mixture was stirred for 30 min and then *a,ß*-unsaturated amide (2 mmol) in THF (1.2 mL) was added, followed by MeOH (4.0 mmol, 160 μL). The reaction tube was washed with THF (5 mL), sealed, and stirred until no starting material was detected by TLC. The reaction mixture was filtered through a pad of celite and concentrated. The product was purified by silica gel chromatography.

Pentan-3-yl-3-phenyl-3-(4,4,5,5-tetramethyl-1,3,2-dioxaborolan-2-yl)propanoate (1b)

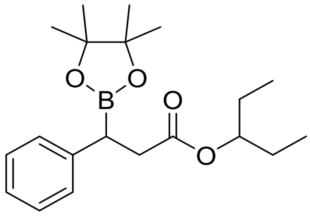

Following the General Procedure A, the title compound was isolated as a colorless oil in 90% yield.

^{1}H NMR (400 MHz, $CDCl_3$) δ 7.27 – 7.11 (m, 5H), 4.77 – 4.71 (m, 1H), 2.89 (dd, *J* = 16.0, 9.6 Hz, 1H), 2.78 – 2.74 (m, 1H), 2.66 (dd, *J* = 16.0, 6.2 Hz, 1H), 1.57 – 1.44 (m, 4H), 1.21 (s, 6H), 1.16 (s, 6H), 0.88 – 0.78 (m, 6H); **^{13}C NMR** (101 MHz, $CDCl_3$) δ 173.49, 141.65, 128.54, 128.33, 125.70, 83.64, 76.80, 37.47, 26.67, 26.65, 24.69, 9.77, 9.69.

***tert*-Butyl-3-phenyl-3-(4,4,5,5-tetramethyl-1,3,2-dioxaborolan-2-yl)propanoate (1c)**

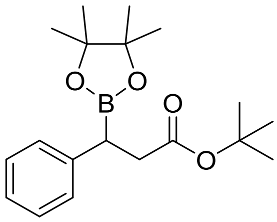

Following the General Procedure A, the title compound was isolated as a white solid in 92% yield. **m.p.** = 51 – 52 ℃.

^{1}H NMR (400 MHz, $CDCl_3$) δ 7.26 – 7.11 (m, 5H), 2.80 (dd, *J* = 15.9, 10.1 Hz, 1H), 2.70 (dd, *J* = 10.0, 6.0 Hz, 1H), 2.58 (dd, *J* = 15.9, 5.9 Hz, 1H), 1.40 (s, 9H), 1.22 (s, 6H), 1.16 (s, 6H); **^{13}C NMR** (101 MHz, $CDCl_3$) δ 172.95, 141.63, 128.51, 128.37, 125.63, 83.58, 80.29, 38.49, 28.20, 24.78, 24.61.

^{1}H NMR (400 MHz, $CDCl_3$) ^{13}C NMR (101 MHz, $CDCl_3$)

Pentan-3-yl-3-(4-fluorophenyl)-3-(4,4,5,5-tetramethyl-1,3,2-dioxaborolan-2-yl)propanoate (5a)

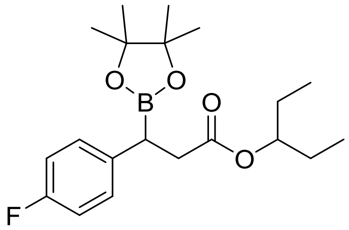

Following the General Procedure A, the title compound was isolated as a colorless oil in 88% yield.

^{1}H NMR (400 MHz, $CDCl_3$) δ 7.20 – 7.15 (m, 2H), 6.97 – 6.91 (m, 2H), 4.76 – 4.70 (m, 1H), 2.85 (dd, *J* = 15.8, 9.1 Hz, 1H), 2.74 (dd, *J* = 9.0, 6.5 Hz, 1H), 2.64 (dd, *J* = 15.8, 6.4 Hz, 1H), 1.59 – 1.43 (m, 4H), 1.21 (s, 6H), 1.17 (s, 6H), 0.87 – 0.77 (m, 6H); **^{13}C NMR** (101 MHz, $CDCl_3$) δ 173.33, 161.29 (d, *J* = 243.1 Hz), 137.22 (d, *J* = 3.1 Hz), 129.69 (d, *J* = 7.8 Hz), 115.30 (d, *J* = 21.1 Hz), 83.74, 76.89, 37.59, 26.65, 26.63, 24.69, 24.67, 9.78, 9.69.

***tert*-Butyl-3-(4-fluorophenyl)-3-(4,4,5,5-tetramethyl-1,3,2-dioxaborolan-2-yl)propanoate (5a′)**

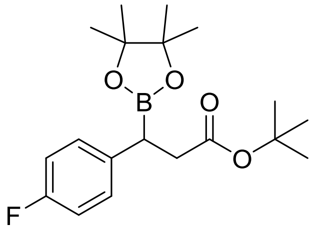

Following the General Procedure A, the title compound was isolated as a colorless oil in 85% yield.

^{1}H NMR (400 MHz, $CDCl_3$) δ 7.19 – 7.14 (m, 2H), 6.96 – 6.90 (m, 2H), 2.75 (dd, *J* = 15.5, 9.6 Hz, 1H), 2.69 – 2.65 (m, 1H), 2.56 (dd, *J* = 15.5, 5.9 Hz, 1H), 1.40 (s, 9H), 1.22 (s, 6H), 1.17 (s, 6H); **^{13}C NMR** (101 MHz, $CDCl_3$) δ 172.74, 161.24 (d, *J* = 242.9 Hz), 137.26 (d, *J* = 3.2 Hz), 129.70 (d, *J* = 7.8 Hz), 115.24 (d, *J* = 21.1 Hz), 83.67, 80.40, 38.62, 28.18, 24.77, 24.60.

Pentan-3-yl-3-(3-chlorophenyl)-3-(4,4,5,5-tetramethyl-1,3,2-dioxaborolan-2-yl)propanoate (5b)

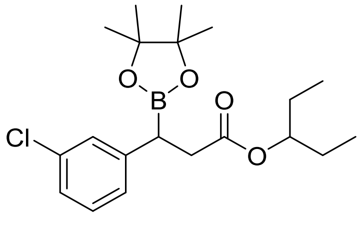

Following the General Procedure A, the title compound was isolated as a white solid in 85% yield. **m.p.** = 50 – 51 ℃.

^{1}H NMR (400 MHz, $CDCl_3$) δ 7.23 – 7.10 (m, 4H), 4.77 – 4.71 (m, 1H), 2.87 (dd, *J* = 15.8, 9.1 Hz, 1H), 2.76 – 2.72 (m, 1H), 2.66 (dd, *J* = 15.8, 6.4 Hz, 1H), 1.57 – 1.46 (m, 4H), 1.22 (s, 6H), 1.18 (s, 6H), 0.85 (t, *J* = 7.4 Hz,

3H), 0.80 (t, *J* = 7.4 Hz, 3H); **^{13}C NMR** (101 MHz, $CDCl_3$) δ 173.14, 143.73, 134.23, 129.71, 128.41, 126.54, 125.92, 83.81, 76.95, 37.15, 26.62, 26.61, 24.64, 9.74, 9.66.

***tert*-Butyl-3-(3-chlorophenyl)-3-(4,4,5,5-tetramethyl-1,3,2-dioxaborolan-2-yl)propanoate (5b′)**

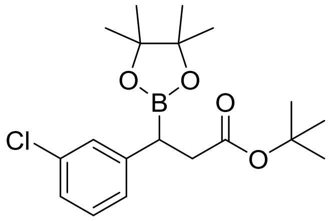

Following the General Procedure A, the title compound was isolated as a colorless oil in 86% yield.
^{1}H NMR (400 MHz, $CDCl_3$) δ 7.21 (s, 1H), 7.19 – 7.09 (m, 3H), 2.77 (dd, *J* = 15.8, 9.6 Hz, 1H), 2.68 (dd, *J* = 9.5, 6.1 Hz, 1H), 2.57 (dd, *J* = 15.8, 6.0 Hz, 1H), 1.40 (s, 9H), 1.22 (s, 6H), 1.17 (s, 6H); **^{13}C NMR** (101 MHz, $CDCl_3$) δ 172.51, 143.76, 134.14, 129.64, 128.41, 126.53, 125.80, 83.70, 80.42, 38.14, 28.11, 24.70, 24.55.

Pentan-3-yl-3-(3-bromophenyl)-3-(4,4,5,5-tetramethyl-1,3,2-dioxaborolan-2-yl)propanoate (5c)

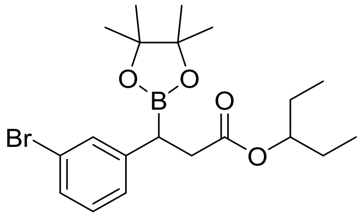

Following the General Procedure A, the title compound was isolated as a colorless oil in 87% yield.
^{1}H NMR (400 MHz, $CDCl_3$) δ 7.38 (s, 1H), 7.27 (d, *J* = 7.7 Hz, 1H), 7.17 – 7.09 (m, 2H), 4.77 – 4.71 (m, 1H), 2.86 (dd, *J* = 15.7, 8.9 Hz, 1H), 2.75 – 2.71 (m, 1H), 2.66 (dd, *J* = 15.7, 6.4 Hz, 1H), 1.61 – 1.43 (m, 4H), 1.22 (s, 6H), 1.18 (s, 6H), 0.85 (t, *J* = 7.4 Hz, 3H), 0.80 (t, *J* = 7.4 Hz, 3H); **^{13}C NMR** (101 MHz, $CDCl_3$) δ 173.10, 144.02, 131.30, 130.01, 128.82, 126.98, 122.57, 83.79, 76.94, 37.16, 26.61, 26.60, 24.62, 9.73, 9.66.

***tert*-Butyl-3-(3-bromophenyl)-3-(4,4,5,5-tetramethyl-1,3,2-dioxaborolan-2-yl)propanoate (5c′)**

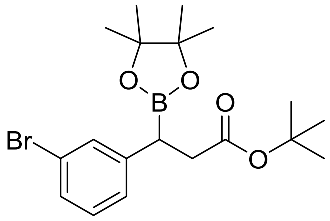

Following the General Procedure A, the title compound was isolated as a colorless oil in 85% yield.
^{1}H NMR (400 MHz, $CDCl_3$) δ 7.37 – 7.09 (m, 4H), 2.77 (dd, *J* = 15.5, 9.8 Hz, 1H), 2.69 – 2.65 (m, 1H), 2.57 (dd, *J* = 15.7, 6.0 Hz, 1H), 1.41 (s, 9H), 1.22 (s, 6H), 1.18 (s, 6H); **^{13}C NMR** (101 MHz, $CDCl_3$) δ 172.58, 144.13, 131.41, 130.02, 128.79, 127.08, 122.56, 83.80, 80.55, 38.23, 28.18, 24.76, 24.62.

Pentan-3-yl-3-(4,4,5,5-tetramethyl-1,3,2-dioxaborolan-2-yl)-3-(3-(trifluoromethoxy)phenyl)propa noate (5d)

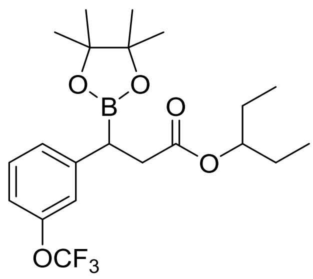

Following the General Procedure A, the title compound was isolated as a colorless oil in 80% yield.

^{1}H NMR (400 MHz, $CDCl_3$) δ 7.28 – 7.24 (m, 1H), 7.16 (d, *J* = 7.8 Hz, 1H), 7.12 (s, 1H), 7.00 (d, *J* = 8.1 Hz, 1H), 4.78 – 4.72 (m, 1H), 2.88 (dd, *J* = 15.7, 9.4 Hz, 1H), 2.79 (dd, *J* = 9.3, 6.1 Hz, 1H), 2.68 (dd, *J* = 15.8, 6.1 Hz, 1H), 1.61 – 1.42 (m, 4H), 1.21 (s, 6H), 1.17 (s, 6H), 0.85 (t, *J* = 7.4 Hz, 3H), 0.80 (t, *J* = 7.4 Hz, 3H); **^{13}C NMR** (101 MHz, $CDCl_3$) δ 173.13, 149.44 (q, *J* = 1.8 Hz), 144.03, 129.73, 126.86, 120.81, 120.61 (q, *J* = 256.6 Hz), 118.27, 83.85, 77.03, 37.14, 26.62, 26.60, 24.58, 24.56, 9.69, 9.60.

***tert*-Butyl-3-(4,4,5,5-tetramethyl-1,3,2-dioxaborolan-2-yl)-3-(3-(trifluoromethoxy)phenyl)propan oate (5d′)**

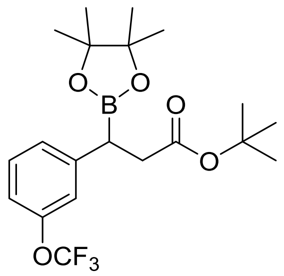

Following the General Procedure A, the title compound was isolated as a colorless oil in 82% yield.

^{1}H NMR (400 MHz, $CDCl_3$) δ 7.28 – 7.24 (m, 1H), 7.15 (d, *J* = 7.7 Hz, 1H), 7.11 (s, 1H), 7.00 (d, *J* = 8.1 Hz, 1H), 2.82 – 2.71 (m, 2H), 2.60 (dd, *J* = 14.8, 5.0 Hz, 1H), 1.40 (s, 9H), 1.22 (s, 6H), 1.17 (s, 6H); **^{13}C NMR** (101 MHz, $CDCl_3$) δ 172.53, 149.42 (q, *J* = 1.6 Hz), 144.11, 129.70, 126.92, 120.84, 120.63 (q, *J* = 256.6 Hz), 118.23, 83.80, 80.54, 38.20, 28.12, 24.70, 24.54.

Pentan-3-yl-3-(4-methoxyphenyl)-3-(4,4,5,5-tetramethyl-1,3,2-dioxaborolan-2-yl)propanoate (5e)

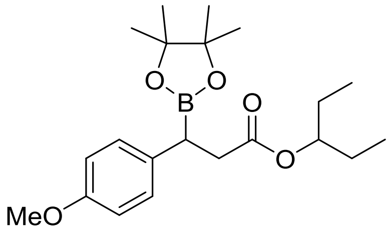

Following the General Procedure A, the title compound was isolated as a colorless oil in 87% yield.

^{1}H NMR (400 MHz, $CDCl_3$) δ 7.14 (d, *J* = 8.4 Hz, 2H), 6.80 (d, *J* = 8.4 Hz, 2H), 4.77 – 4.70 (m, 1H), 3.77 (s, 3H), 2.84 (dd, *J* = 15.6, 9.2 Hz, 1H), 2.71 – 2.67 (m, 1H), 2.62 (dd, *J* = 15.6, 6.3 Hz, 1H), 1.59 – 1.42 (m, 4H), 1.21 (s, 6H), 1.17 (s, 6H), 0.86 (t, *J* = 7.4 Hz, 3H), 0.80 (t, *J* = 7.4 Hz, 3H); **^{13}C NMR** (101 MHz, $CDCl_3$) δ 173.56, 157.72, 133.57, 129.25, 114.00, 83.58, 76.74, 55.32, 37.78, 26.66, 26.64, 24.70, 9.80, 9.71.

***tert*-Butyl-3-(4-methoxyphenyl)-3-(4,4,5,5-tetramethyl-1,3,2-dioxaborolan-2-yl)propanoate (5e′)**

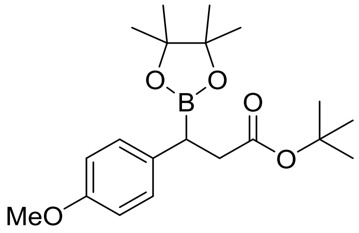

Following the General Procedure A, the title compound was isolated as a colorless oil in 84% yield.

^{1}H NMR (400 MHz, $CDCl_3$) δ 7.13 (d, *J* = 8.6 Hz, 2H), 6.80 (d, *J* = 8.6 Hz, 2H), 3.77 (s, 3H), 2.75 (dd, *J* = 15.8, 9.8 Hz, 1H), 2.63 (dd, *J* = 9.8, 6.1 Hz, 1H), 2.54 (dd, *J* = 15.8, 6.1 Hz, 1H), 1.40 (s, 9H), 1.22 (s, 6H), 1.17 (s, 6H); **^{13}C NMR** (101 MHz, $CDCl_3$) δ 172.99, 157.66, 133.62, 129.28, 113.95, 83.53, 80.22, 55.30, 38.81, 28.21, 24.79, 24.63.

Pentan-3-yl-3-(4,4,5,5-tetramethyl-1,3,2-dioxaborolan-2-yl)-3-(o-tolyl)propanoate (5f)

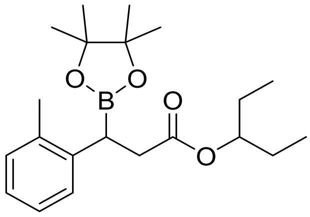

Following the General Procedure A, the title compound was isolated as as a white solid in 88% yield. **m.p.** = 51 – 52 °

^{1}H NMR (400 MHz, $CDCl_3$) δ 7.19 – 7.01 (m, 4H), 4.77 – 4.70 (m, 1H), 2.97 (dd, *J* = 9.8, 6.1 Hz, 1H), 2.88 (dd, *J* = 16.3, 9.8 Hz, 1H), 2.62 (dd, *J* = 16.3, 6.1 Hz, 1H), 2.36 (s, 3H), 1.58 – 1.44 (m, 4H), 1.21 (s, 6H), 1.16 (s, 6H), 0.86 – 0.78 (m, 6H); **^{13}C NMR** (101 MHz, $CDCl_3$) δ 173.63, 140.06, 136.29, 130.45, 127.63, 126.09, 125.57, 83.55, 76.74, 36.90, 26.64, 24.71, 24.69, 20.19, 9.76, 9.70.

***tert*-Butyl-3-(4,4,5,5-tetramethyl-1,3,2-dioxaborolan-2-yl)-3-(o-tolyl)propanoate (5f′)**

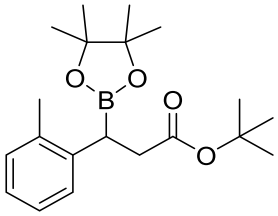

Following the General Procedure A, the title compound was isolated as a colorless oil in 86% yield.

^{1}H NMR (400 MHz, $CDCl_3$) δ 7.17 (d, *J* = 7.2 Hz, 1H), 7.11 – 7.01 (m, 3H), 2.90 (dd, *J* = 10.0, 6.4 Hz, 1H), 2.77 (dd, *J* = 16.5, 10.1 Hz, 1H), 2.54 (dd, *J* = 16.5, 6.3 Hz, 1H), 2.35 (s, 3H), 1.39 (s, 9H), 1.21 (s, 6H), 1.16 (s, 6H); **^{13}C NMR** (101 MHz, $CDCl_3$) δ 173.06, 140.07, 136.29, 130.38, 127.71, 126.02, 125.47, 83.47, 80.16, 37.89, 28.17, 24.78, 24.63, 20.16.

Pentan-3-yl-3-(naphthalen-1-yl)-3-(4,4,5,5-tetramethyl-1,3,2-dioxaborolan-2-yl)propanoate (5g)

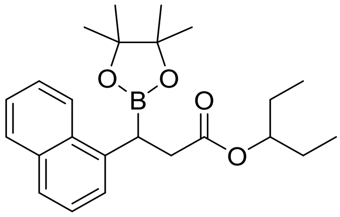

Following the General Procedure A, the title compound was isolated as a colorless oil in 83% yield.

^{1}H NMR (400 MHz, $CDCl_3$) δ 8.18 (d, *J* = 8.3 Hz, 1H), 7.83 – 7.81 (m, 1H), 7.67 (dd, *J* = 7.5, 1.5 Hz, 1H), 7.51 – 7.35 (m, 4H), 4.78 – 4.72 (m, 1H), 3.52 (dd, *J* = 9.7, 6.2 Hz, 1H), 3.04 (dd, *J* = 16.7, 9.8 Hz, 1H), 2.78 (dd, *J* = 16.7, 6.2 Hz, 1H), 1.58 – 1.42 (m, 4H), 1.23 (s, 6H), 1.15 (s, 6H), 0.86 – 0.82 (m, 3H), 0.79 – 0.75 (m, 3H); **^{13}C NMR** (101 MHz, $CDCl_3$) δ 173.60, 138.34, 134.23, 132.11, 128.85, 126.55, 125.74, 125.67, 125.48, 124.29, 83.82, 76.84, 37.38, 26.61, 26.60, 24.77, 24.70, 9.77, 9.69.

***tert*-Butyl-3-(naphthalen-1-yl)-3-(4,4,5,5-tetramethyl-1,3,2-dioxaborolan-2-yl)propanoat (5g′)**

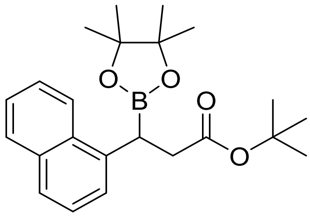

Following the General Procedure A, the title compound was isolated as a colorless oil in 84% yield.

^{1}H NMR (400 MHz, $CDCl_3$) δ 8.18 (d, *J* = 8.3 Hz, 1H), 7.81 (d, *J* = 7.9 Hz, 1H), 7.66 (d, *J* = 7.3 Hz, 1H), 7.50 – 7.35 (m, 4H), 3.46 (dd, *J* = 9.8, 6.3 Hz, 1H), 2.94 (dd, *J* = 16.7, 10.1 Hz, 1H), 2.70 (dd, *J* = 16.6, 6.2 Hz, 1H), 1.39 (s, 9H), 1.23 (s, 6H), 1.15 (s, 6H); **^{13}C NMR** (101 MHz, $CDCl_3$) δ 172.98, 138.35, 134.18, 132.15, 128.81, 126.45, 125.71, 125.69, 125.62, 125.44, 124.32, 83.73, 80.28, 38.38, 28.17, 24.83, 24.64.

Pentan-3-yl-3-(4,4,5,5-tetramethyl-1,3,2-dioxaborolan-2-yl)-3-(thiophen-2-yl)propanoate (5h)

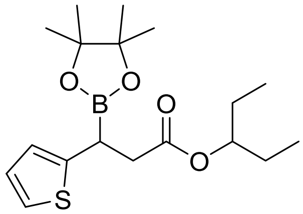

Following the General Procedure A, the title compound was isolated as a colorless oil in 83% yield.

^{1}H NMR (400 MHz, $CDCl_3$) δ 7.08 (dd, *J* = 5.1, 1.1 Hz, 1H), 6.89 (dd, *J* = 5.1, 3.5 Hz, 1H), 6.83 (d, *J* = 3.4 Hz, 1H), 4.79 – 4.73 (m, 1H), 3.04 (dd, *J* = 9.8, 6.2 Hz, 1H), 2.92 – 2.85 (m, 1H), 2.75 (dd, *J* = 16.8, 6.1 Hz, 1H), 1.60 – 1.46 (m, 4H), 1.24 (s, 6H), 1.21 (s, 6H), 0.88 – 0.82 (m, 6H); **^{13}C NMR** (101 MHz, $CDCl_3$) δ 173.05, 144.40, 126.91, 124.22, 123.08, 83.94, 77.04, 38.35, 26.67, 24.75, 24.70, 9.77, 9.74.

***tert*-Butyl-3-(4,4,5,5-tetramethyl-1,3,2-dioxaborolan-2-yl)-3-(thiophen-2-yl)propanoate (5h′)**

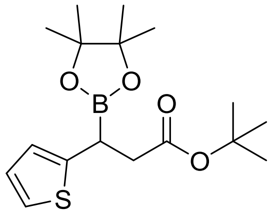

Following the General Procedure A, the title compound was isolated as a colorless oil in 82% yield.

^{1}H NMR (400 MHz, $CDCl_3$) δ 7.00 (d, *J* = 5.1 Hz, 1H), 6.83 – 6.81 (m, 1H), 6.74 (d, *J* = 3.0 Hz, 1H), 2.90 (dd, *J* = 9.9, 6.2 Hz, 1H), 2.72 (dd, *J* = 16.9, 10.1 Hz, 1H), 2.60 (dd, *J* = 16.9, 6.2 Hz, 1H), 1.36 (s, 9H), 1.18 (s, 6H), 1.14 (s, 6H); **^{13}C NMR** (101 MHz, $CDCl_3$) δ 172.42, 144.42, 126.86, 124.10, 122.99, 83.82, 80.49, 39.33, 28.17, 24.80, 24.60.

Pentan-3-yl-3-(4,4,5,5-tetramethyl-1,3,2-dioxaborolan-2-yl)butanoate (5i)

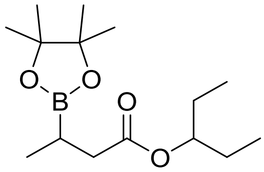

Following the General Procedure A, the title compound was isolated as a colorless oil in 86% yield.

^{1}H NMR (400 MHz, $CDCl_3$) δ 4.79 – 4.73 (m, 1H), 2.45 (dd, *J* = 16.3, 7.6 Hz, 1H), 2.35 (dd, *J* = 16.4, 6.9 Hz, 1H), 1.60 – 1.47 (m, 4H), 1.44 – 1.35 (m, 1H), 1.24 (m, 12H), 1.01 (d, *J* = 7.5 Hz, 3H), 0.92 – 0.81 (m, 6H); **^{13}C NMR** (101 MHz, $CDCl_3$) δ 173.98, 83.25, 76.52, 37.95, 26.71, 26.69, 24.85, 24.82, 15.16, 9.83, 9.80.

***tert*-Butyl-3-(4,4,5,5-tetramethyl-1,3,2-dioxaborolan-2-yl)butanoate (5i′)**

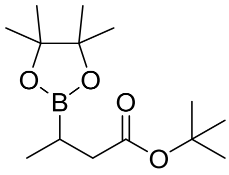

Following the General Procedure A, the title compound was isolated as a colorless oil in 88% yield.

^{1}H NMR (400 MHz, $CDCl_3$) δ 2.35 (dd, *J* = 16.5, 7.8 Hz, 1H), 2.26 (dd, *J* = 16.5, 6.9 Hz, 1H), 1.43 (s, 9H), 1.39 – 1.29 (m, 1H), 1.24 (s, 6H), 1.23 (s, 6H), 0.99 (d, *J* = 7.5 Hz, 3H); **^{13}C NMR** (101 MHz, $CDCl_3$) δ 173.43, 83.18, 79.97, 38.96, 28.27, 24.88, 24.80, 15.08.

Pentan-3-yl-3-(4,4,5,5-tetramethyl-1,3,2-dioxaborolan-2-yl)hexanoate (5j)

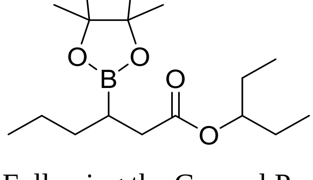

Following the General Procedure A, the title compound was isolated as a colorless oil in 85% yield.

^{1}H NMR (400 MHz, $CDCl_3$) δ 4.78 – 4.72 (m, 1H), 2.44 (dd, *J* = 16.5, 8.2 Hz, 1H), 2.38 (dd, *J* = 16.4, 5.9 Hz, 1H), 1.62 – 1.28 (m, 9H), 1.24 (s, 6H), 1.23 (s, 6H), 0.90 – 0.85 (m, 9H); **^{13}C NMR** (101 MHz, $CDCl_3$) δ 174.10, 83.15, 76.47, 35.92, 32.83, 26.68, 26.66, 24.89, 24.82, 22.00, 14.41, 9.80, 9.77.

***tert*-Butyl-3-(4,4,5,5-tetramethyl-1,3,2-dioxaborolan-2-yl)hexanoate (5j′)**

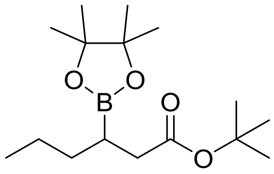

Following the General Procedure A, the title compound was isolated as a colorless oil in 86% yield.
^{1}H NMR (400 MHz, $CDCl_3$) δ 2.34 (dd, *J* = 14.8, 6.4 Hz, 1H), 2.29 (dd, *J* = 14.8, 4.6 Hz, 1H), 1.43 – 1.23 (m, 26H), 0.88 (t, *J* = 6.9 Hz, 3H); **^{13}C NMR** (101 MHz, $CDCl_3$) δ 173.62, 83.12, 79.95, 37.01, 32.83, 28.26, 24.97, 24.80, 22.07, 14.45.

Pentan-3-yl-5-phenyl-3-(4,4,5,5-tetramethyl-1,3,2-dioxaborolan-2-yl)pentanoate (5k)

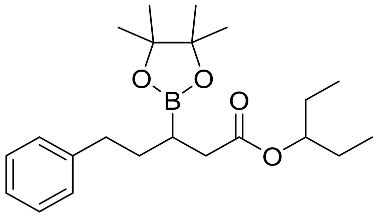

Following the General Procedure A, the title compound was was isolated as a colorless oil in 89% yield.
^{1}H NMR (400 MHz, $CDCl_3$) δ 7.28 – 7.14 (m, 5H), 4.79 – 4.73 (m, 1H), 2.70 – 2.58 (m, 2H), 2.54 – 2.41 (m, 2H), 1.84 – 1.75 (m, 1H), 1.70 – 1.63 (m, 1H), 1.61 – 1.47 (m, 4H), 1.46 – 1.38 (m, 1H), 1.26 (s, 6H), 1.25 (s, 6H), 0.89 – 0.85 (m, 6H); **^{13}C NMR** (101 MHz, $CDCl_3$) δ 173.90, 142.76, 128.53, 128.39, 125.77, 83.31, 76.61, 35.92, 35.29, 32.76, 26.68, 26.66, 24.95, 24.89, 9.83, 9.81.

***tert*-Butyl-5-phenyl-3-(4,4,5,5-tetramethyl-1,3,2-dioxaborolan-2-yl)pentanoate (5k′)**

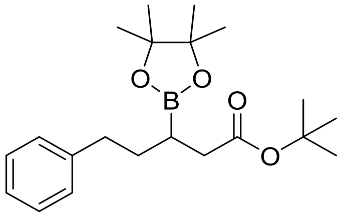

Following the General Procedure A, the title compound was was isolated as a colorless oil in 87% yield.
^{1}H NMR (400 MHz, $CDCl_3$) δ 7.28 – 7.14 (m, 5H), 2.69 – 2.57 (m, 2H), 2.44 – 2.33 (m, 2H), 1.82 – 1.73 (m, 1H), 1.67 – 1.58 (m, 1H), 1.43 – 1.33 (m, 10H), 1.26 (s, 6H), 1.25 (s, 6H); **^{13}C NMR** (101 MHz, $CDCl_3$) δ 173.41, 142.85, 128.55, 128.40, 125.77, 83.27, 80.10, 37.00, 35.35, 32.75, 28.26, 25.01, 24.86.

Pentan-3-yl 4-methyl-3-(4,4,5,5-tetramethyl-1,3,2-dioxaborolan-2-yl)pentanoate (5l)

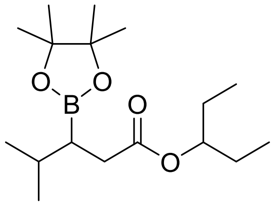

Following the General Procedure A, the title compound was isolated as a colorless oil in 84% yield.

^{1}H NMR (400 MHz, $CDCl_3$) δ 4.77 – 4.71 (m, 1H), 2.48 (dd, *J* = 16.6, 10.6 Hz, 1H), 2.36 (dd, *J* = 16.6, 5.6 Hz, 1H), 1.81 – 1.70 (m, 1H), 1.59 – 1.49 (m, 4H), 1.33 – 1.27 (m, 1H), 1.25 (s, 6H), 1.23 (s, 6H), 0.96 – 0.85 (m, 12H); **^{13}C NMR** (101 MHz, $CDCl_3$) δ 174.43, 83.16, 76.44, 33.98, 29.23, 26.68, 26.66, 25.04, 24.90, 22.18, 21.50, 9.79.

***tert*-Butyl-4-methyl-3-(4,4,5,5-tetramethyl-1,3,2-dioxaborolan-2-yl)pentanoate (5l′)**

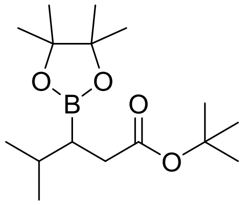

Following the General Procedure A, the title compound was isolated as a colorless oil in 83% yield.

^{1}H NMR (400 MHz, $CDCl_3$) δ 2.38 (dd, *J* = 16.7, 10.8 Hz, 1H), 2.29 (dd, *J* = 16.8, 5.7 Hz, 1H), 1.77 – 1.69 (m, 1H), 1.43 (s, 9H), 1.25 (s, 6H), 1.23 (s, 6H), 0.94 – 0.92 (m, 6H); **^{13}C NMR** (101 MHz, $CDCl_3$) δ 173.93, 83.12, 79.86, 34.96, 29.13, 28.25, 25.14, 24.84, 22.20, 21.68.

Pentan-3-yl-3-cyclohexyl-3-(4,4,5,5-tetramethyl-1,3,2-dioxaborolan-2-yl)propanoate (5m)

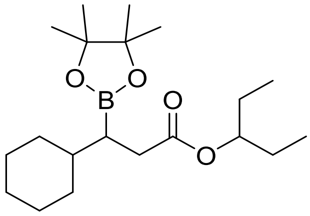

Following the General Procedure A, the title compound was isolated as a colorless oil in 88% yield.

^{1}H NMR (400 MHz, $CDCl_3$) δ 4.77 – 4.71 (m, 1H), 2.48 (dd, *J* = 16.6, 10.5 Hz, 1H), 2.38 (dd, *J* = 16.6, 5.5 Hz, 1H), 1.71 – 1.28 (m, 11H), 1.25 (s, 6H), 1.23 (s, 6H), 1.19 – 0.97 (m, 5H), 0.91 – 0.83 (m, 6H); **^{13}C NMR** (101 MHz, $CDCl_3$) δ 174.52, 83.14, 76.39, 39.32, 33.80, 32.69, 32.17, 26.87, 26.80, 26.69, 26.67, 26.65, 25.05, 24.88, 9.80

***tert*-Butyl-3-cyclohexyl-3-(4,4,5,5-tetramethyl-1,3,2-dioxaborolan-2-yl)propanoate (5m′)**

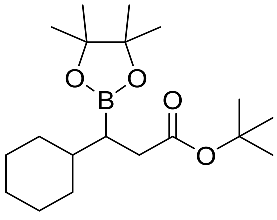

Following the General Procedure A, the title compound was isolated as a colorless oil in 87% yield.
^{1}H NMR (400 MHz, $CDCl_3$) δ 2.38 (dd, *J* = 16.7, 10.7 Hz, 1H), 2.30 (dd, *J* = 16.8, 5.7 Hz, 1H), 1.70 – 1.58 (m, 5H), 1.43 – 1.33 (m, 10H), 1.26 – 0.96 (m, 18H); **^{13}C NMR** (101 MHz, $CDCl_3$) δ 174.01, 83.11, 79.83, 39.21, 34.81, 32.69, 32.35, 28.25, 26.89, 26.82, 26.72, 25.16, 24.84.

Pentan-3-yl 3-cyclopropyl-3-(4,4,5,5-tetramethyl-1,3,2-dioxaborolan-2-yl)propanoate (5n)

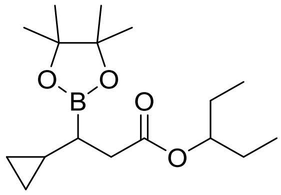

Following the General Procedure A, the title compound was isolated as a colorless oil in 86% yield.
^{1}H NMR (400 MHz, $CDCl_3$) δ 4.78 – 4.72 (m, 1H), 2.58 – 2.46 (m, 2H), 1.62 – 1.47 (m, 4H), 1.25 (s, 6H), 1.24 (s, 6H), 0.89 – 0.85 (m, 6H), 0.77 – 0.65 (m, 2H), 0.47 – 0.37 (m, 2H), 0.21 – 0.18 (m, 1H), 0.09 – 0.05 (m, 1H); **^{13}C NMR** (101 MHz, $CDCl_3$) δ 174.05, 83.21, 76.52, 36.51, 26.65, 24.89, 24.74, 12.02, 9.80, 4.77, 4.08.

***tert*-Butyl-3-cyclopropyl-3-(4,4,5,5-tetramethyl-1,3,2-dioxaborolan-2-yl)propanoate (5n′)**

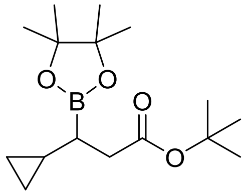

Following the General Procedure A, the title compound was isolated as a colorless oil in 87% yield.
^{1}H NMR (400 MHz, $CDCl_3$) δ 2.49 – 2.38 (m, 2H), 1.43 (s, 9H), 1.25 (s, 6H), 1.24 (s, 6H), 0.73 – 0.63 (m, 2H), 0.45 – 0.36 (m, 2H), 0.18 – 0.04 (m, 2H); **^{13}C NMR** (101 MHz, $CDCl_3$) δ 173.51, 83.17, 79.96, 37.56, 28.24, 24.95, 24.69, 11.92, 4.80, 3.99.

Pentan-3-yl-5-methyl-3-(4,4,5,5-tetramethyl-1,3,2-dioxaborolan-2-yl)hex-4-enoate (5p)[9]

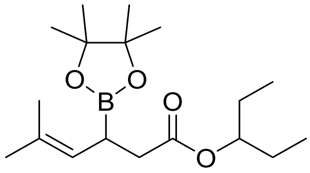

To an aqueous solution (5 mL) of $Cu(OH)_2$ (10.0 mg, 5 mol%) and *race* 6,6’-bis(1-hydroxy-2,2-dimethylpropyl)-2,2’-bipyridine (39.5 mg, 6 mol%) was added an aqueous acetic acid (24 mM, 5 mL). After stirred vigorously for 1 h at room temperature, the resultant mixture was allowed to cool to 5 °C. Dienoesters (2.0 mmol) and $B_2(pin)_2$ (609 mg, 2.4 mmol) were then added successively at the same temperature. The mixture was stirred for 5 h and diluted with DCM (10 mL). The layers were separated and the aqueous layer was extracted with DCM (2 × 10 mL). The combined organic layers were washed with water (5 mL) and brine (5 mL), dried ($MgSO_4$), filtered and concentrated in vacuo. The crude product was purified by flash column chromatography. **^{1}H NMR** (400 MHz, $CDCl_3$) δ 5.04 – 5.01 (m, 1H), 4.77 – 4.71 (m, 1H), 2.51 (dd, *J* = 16.1, 8.4 Hz, 1H), 2.40 (dd, *J* = 16.1, 6.8 Hz, 1H),

2.32 (dd, J = 16.0, 8.6 Hz, 1H), 1.68 (d, J = 1.1 Hz, 3H), 1.63 (d, J = 1.3 Hz, 3H), 1.58 – 1.48 (m, 4H), 1.23 (s, 6H), 1.21 (s, 6H), 0.88 – 0.84 (m, 6H); **^{13}C NMR** (101 MHz, $CDCl_3$) δ 173.79, 132.28, 123.26, 83.25, 76.60, 36.56, 26.69, 25.98, 24.81, 24.66, 18.26, 9.76; **HRMS** (ESI) *m/z* calcd for $C_{18}H_{34}BO_4$ $(M+H)^+$ 325.2545, found 325.2546.

Pentan-3-yl (*R*,*E*)-3-(4,4,5,5-tetramethyl-1,3,2-dioxaborolan-2-yl)hex-4-enoate (5o)[9]

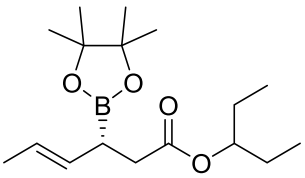

To an aqueous solution (5.0 mL) of $Cu(OH)_2$ (10.0 mg, 5 mol%) and (*S*,*S*)-6,6'–bis(1-hydroxy-2,2-dimethylpropyl)-2,2'-bipyridine (39.5 mg, 6 mol%) was added an aqueous acetic acid (24 mM, 5.0 mL). After stirred vigorously for 1 h at room temperature, the resultant mixture was allowed to cool to 5 °C. Dienoesters (2.0 mmol) and $B_2(pin)_2$ (609 mg, 2.4 mmol) were then added successively at the same temperature. The mixture was stirred for 5 h and diluted with DCM (10 mL). The layers were separated and the aqueous layer was extracted with DCM (2 × 10.0 mL). The combined organic layers were washed with water (5.0 mL) and brine (5.0 mL), dried ($MgSO_4$), filtered and concentrated in vacuo. The crude products were purified by flash column chromatography. **^{1}H NMR** (400 MHz, $CDCl_3$) δ 5.49 – 5.38 (m, 2H), 4.78 – 4.72 (m, 1H), 2.55 (dd, J = 16.4, 8.5 Hz, 1H), 2.45 (dd, J = 16.4, 6.8 Hz, 1H), 2.17 – 2.12 (m, 1H), 1.63 (d, J = 4.3 Hz, 3H), 1.58 – 1.48 (m, 4H), 1.24 (s, 6H), 1.22 (s, 6H), 0.89 – 0.84 (m, 6H); **^{13}C NMR** (101 MHz, $CDCl_3$) δ 173.67, 129.91, 125.06, 83.43, 76.64, 35.92, 26.69, 24.82, 24.73, 18.31, 9.78; **HRMS** (ESI) *m/z* calcd for $C_{17}H_{32}BO_4$ $(M+H)^+$ 311.2388, found 311.2381. HPLC analysis of the product after being oxidized by $NaBO_3$•$4H_2O$ and then esterified with benzoyl chloride (IA, Hexanes: *i*-PrOH = 90:10, 1.0 mL/min, 25 ℃, λ = 225 nm) indicated 98.5% ee: t_R = 8.767 min (major), t_R = 13.628 min (minor); $[\alpha]_D^{25}$ = -7.7 (c = 1, CH_2Cl_2).

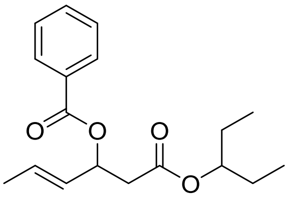
race

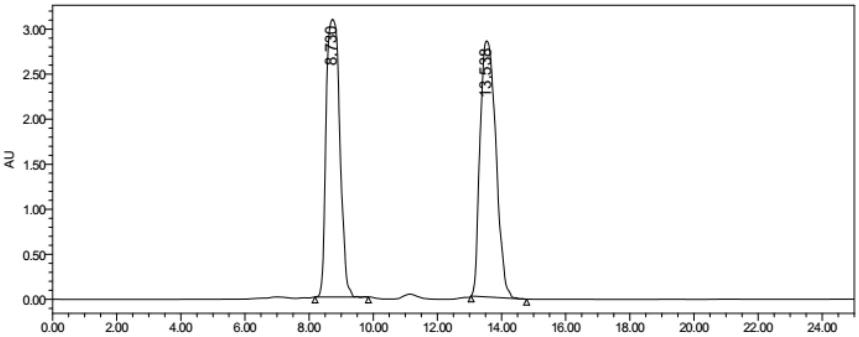

	Channel Description	RT (min)	Area	% Area	Height
1	W2489 ChB 225nm	8.730	91263737	48.36	3081931
2	W2489 ChB 225nm	13.538	97451629	51.64	2840859

Supplementary Figure 3. HPLC analysis of the *race*-**5o** after oxidization and esterification

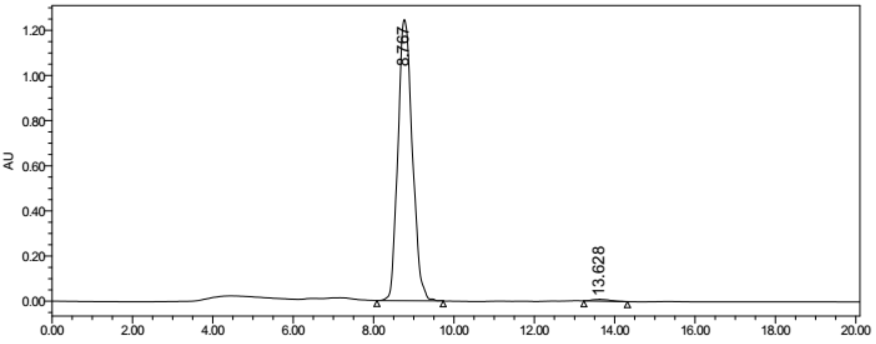

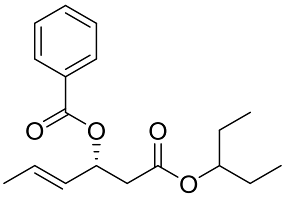

	Channel Description	RT (min)	Area	% Area	Height
1	W2489 ChB 225nm	8.767	31007445	99.25	1244525
2	W2489 ChB 225nm	13.628	234317	0.75	7421

Supplementary Figure 4. HPLC analysis of the chiral-**5o** after oxidization and esterification

***N,N*-Diethyl-3-phenyl-3-(4,4,5,5-tetramethyl-1,3,2-dioxaborolan-2-yl)propanamide (5q)**

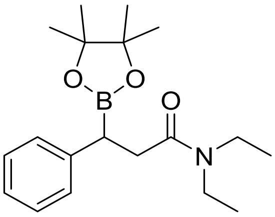

Following the General Procedure B, the title compound was was isolated as a white solid in 85% yield.
^{1}H NMR (400 MHz, $CDCl_3$) δ 7.28 – 7.09 (m, 5H), 3.56 – 3.22 (m, 4H), 2.90 – 2.79 (m, 2H), 2.59 – 2.55 (m, 1H), 1.19 – 1.14 (m, 6H), 1.11 (s, 6H), 1.11 (s, 6H); **^{13}C NMR** (101 MHz, $CDCl_3$) δ 174.91, 143.66, 128.37, 128.15, 124.95, 81.74, 42.45, 41.31, 38.05, 24.91, 24.84, 13.95, 13.00.

Procedures for synthesis of *α*-functionalized-*β*-boronyl carbonyls.

General Procedure C: synthesis of *syn α*-functionalized-*β*-boronyl esters (4b, 8a-8v).

A solution of diisopropylamine (0.275 mmol, 38.5 μL) in toluene (0.6 mL) was cooled to 0 ℃ and treated with *n*-BuLi (110 μL, 2.5 M in hexane) dropwise. The reaction mixture was stirred for 15 min and cooled to -78 ℃. To the freshly prepared solution of LDA was added a solution of *β*-boronyl ester (0.25 mmol) in THF (0.4 mL) dropwise over 2 minutes. After stirring at the same temperature for 15 min, the electrophile (0.375 mmol) was added to the reaction mixture dropwise over one minute and stirred for 8 h. Then the reaction was quenched with saturated aq. NH_4Cl solution (1.0 mL) and diluted with DCM (5.0 mL). The layers were separated, the aqueous phase was extracted with DCM (2 × 5.0 mL), the combined organic phases were washed with water and brine, dried ($MgSO_4$) and concentrated in vacuo. The crude product was purified by flash column chromatography to yield the desired *syn*-product.

General Procedure D: synthesis of *anti* α-functionalized-β-boronyl esters (3c, 7a-7v).

A solution of diisopropylamine (0.275 mmol, 38.5 μL) in THF (0.5 mL) was cooled to 0 ℃ and treated with *n*-BuLi (110 μL, 2.5 M in hexane) dropwise. The reaction mixture was stirred for 15 minutes and cooled to -78 ℃. To the freshly prepared solution of LDA was added a solution of β-boronyl ester (0.25 mmol) in THF/HMPA (0.5 ml/0.2 ml) dropwise over 2 minutes. After stirring at the same temperature for 3 h, the electrophile (0.375 mmol) was added to the reaction mixture dropwise over one minute and stirred for 8 h. Then the reaction was quenched with saturated aq. NH_4Cl solution (1.0 mL) and diluted with DCM (5.0 mL). The layers were separated, the aqueous phase was extracted with DCM (2 × 5.0 mL), the combined organic phases were washed with water and brine, dried ($MgSO_4$) and concentrated in vacuo. The crude product was purified by flash column chromatography to yield the desired *anti*-product.

General Procedure E: synthesis of *anti* α-functionalized-β-boronyl Amides (7w-7x).

A solution of diisopropylamine (0.275 mmol, 38.5 μL) in THF (0.5 mL) was cooled to 0 ℃ and treated with *n*-BuLi (110 μL, 2.5 M in hexane) dropwise. The reaction mixture was stirred for 15 minutes and cooled to -78 ℃. To the freshly prepared solution of LDA was added a solution of β-boronyl amide (0.25 mmol) in THF (0.5 ml) dropwise over 2 minutes. After stirring at the same temperature for 15 minutes, the electrophile (0.375 mmol) was added to the reaction mixture dropwise over one minute and stirred for 8 h. Then the reaction was quenched with saturated aq. NH_4Cl solution (1.0 mL) and diluted with DCM (5.0 mL). The layers were separated, the aqueous phase was extracted with DCM (2 × 5.0 mL), the combined organic phases were washed with water and brine, dried ($MgSO_4$) and concentrated in vacuo. The crude product was purified by flash column chromatography to yield the desired *anti*-product.

***syn*-Pentan-3-yl-2-(phenyl(4,4,5,5-tetramethyl-1,3,2-dioxaborolan-2-yl)methyl)pent-4-enoate (4b)**

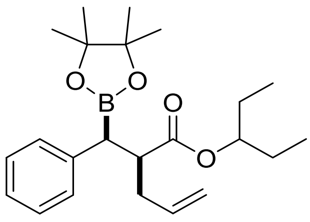

Following the General Procedure C using **1b** (0.25 mmol, 86.6 mg) and allyl bromide (0.375 mmol, 32.5 μL). Analysis of the crude ^{1}H NMR indicated 10:1 diastereoselectivity. Purification by silica gel chromatography gave 70.5 mg (73% yield) of title compound as a colorless oil.

^{1}H NMR (400 MHz, $CDCl_3$) δ 7.25 – 7.07 (m, 5H), 5.87 – 5.77 (m, 1H), 5.09 (dd, *J* = 17.1, 1.6 Hz, 1H), 5.01 (dd, *J* = 10.1, 0.8 Hz, 1H), 4.48 – 4.42 (m, 1H), 3.11 – 3.04 (m, 1H), 2.64 (d, *J* = 11.8 Hz, 1H), 2.46 – 2.35 (m, 2H), 1.38 – 1.30 (m, 2H), 1.20 – 1.12 (m, 14H), 0.73 – 0.69 (m, 3H), 0.50 – 0.46 (m, 3H); **^{13}C NMR** (101 MHz, $CDCl_3$) δ 174.73, 139.96, 135.64, 129.07, 128.34, 125.89, 117.01, 83.69, 76.44, 48.71, 37.75, 26.01, 25.91, 24.71, 24.62, 9.61, 9.12; **HRMS** (ESI) *m/z* calcd for $C_{23}H_{36}BO_4$ $(M+H)^+$ 387.2701, found 387.2704.

***syn*-Pentan-3-yl-2-((4-fluorophenyl)(4,4,5,5-tetramethyl-1,3,2-dioxaborolan-2-yl)methyl)pent-4-enoate (8a)**

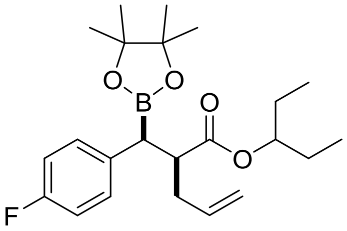

Following the General Procedure C using **5a** (0.25 mmol, 91.1 mg) and allyl bromide (0.375 mmol, 32.5 μL). Analysis of the crude ^{1}H NMR indicated 10:1 diastereoselectivity. Purification by silica gel chromatography gave 68.7 mg (68% yield) of title compound as a colorless oil.

^{1}H NMR (400 MHz, $CDCl_3$) δ 7.22 – 7.15 (m, 2H), 6.92 – 6.86 (m, 2H), 5.86 – 5.75 (m, 1H), 5.08 (dd, *J* = 17.1, 1.3 Hz, 1H), 5.01 (dd, *J* = 10.1, 0.8 Hz, 1H), 4.50 – 4.44 (m, 1H), 3.06 – 2.99 (m, 1H), 2.62 (d, *J* = 11.8 Hz, 1H), 2.45 – 2.31 (m, 2H), 1.39 – 1.31 (m, 2H), 1.21 – 1.14 (m, 14H), 0.74 – 0.71 (m, 3H), 0.52 – 0.48 (m, 3H); **^{13}C NMR** (101 MHz, $CDCl_3$) δ 174.63, 161.49 (d, *J* = 243.2 Hz), 135.70 (d, *J* = 3.1 Hz), 135.46, 130.47 (d, *J* = 7.8 Hz), 117.15, 115.11 (d, *J* = 21.1 Hz), 83.80, 76.58, 48.84, 37.66, 26.05, 25.96, 24.72, 24.62, 9.62, 9.09; **HRMS** (ESI) *m/z* calcd for $C_{23}H_{35}BFO_4$ $(M+H)^+$ 405.2607, found 405.2610.

***syn*-Pentan-3-yl-2-((3-chlorophenyl)(4,4,5,5-tetramethyl-1,3,2-dioxaborolan-2-yl)methyl)pent-4-enoate (8b)**

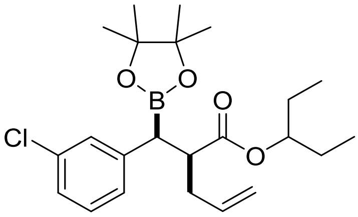

Following the General Procedure C using **5b** (0.25 mmol, 95.2 mg) and allyl bromide (0.375 mmol, 32.5 μL). Analysis of the crude ^{1}H NMR indicated 10:1 diastereoselectivity. Purification by silica gel chromatography gave 75.7 mg (72% yield) of title compound as a colorless oil.

^{1}H NMR (400 MHz, $CDCl_3$) δ 7.24 (s, 1H), 7.16 – 7.08 (m, 3H), 5.85 – 5.75 (m, 1H), 5.09 (d, *J* = 17.1 Hz, 1H), 5.02 (d, *J* = 10.1 Hz, 1H), 4.52 – 4.46 (m, 1H), 3.07 – 3.01 (m, 1H), 2.62 (d, *J* = 11.8 Hz, 1H), 2.45 – 2.34 (m, 2H), 1.43 – 1.31 (m, 2H), 1.23 – 1.17 (m, 14H), 0.75 – 0.71 (m, 3H), 0.54 – 0.51 (m, 3H); **^{13}C NMR** (101 MHz, $CDCl_3$) δ 174.46, 142.24, 135.35, 134.11, 129.58, 129.15, 127.32, 126.14, 117.27, 83.93, 76.72, 48.53, 37.61, 26.05, 25.97, 24.69, 24.65, 9.61, 9.12; **HRMS** (ESI) *m/z* calcd for $C_{23}H_{35}BClO_4$ $(M+H)^+$ 421.2311, found 421.2317.

***syn*-Pentan-3-yl-2-((3-bromophenyl)(4,4,5,5-tetramethyl-1,3,2-dioxaborolan-2-yl)methyl)pent-4-enoate (8c)**

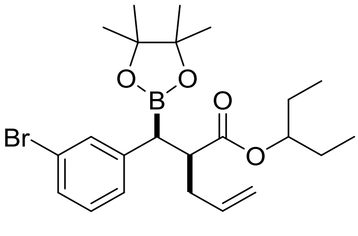

Following the General Procedure C using **5c** (0.25 mmol, 106.3 mg) and allyl bromide (0.375 mmol, 32.5 μL). Analysis of the crude ^{1}H NMR indicated 12:1 diastereoselectivity. Purification by silica gel chromatography gave 86.1 mg (74% yield) of title compound as a colorless oil.

^{1}H NMR (400 MHz, $CDCl_3$) δ 7.39 – 7.39 (m, 1H), 7.24 (d, *J* = 7.9 Hz, 1H), 7.17 (d, *J* = 7.8 Hz, 1H), 7.09 – 7.05 (m, 1H), 5.85 – 5.75 (m, 1H), 5.09 (dd, *J* = 17.1, 1.2 Hz, 1H), 5.02 (d, *J* = 10.1 Hz, 1H), 4.52 – 4.46 (m, 1H), 3.07 – 3.00 (m, 1H), 2.60 (d, *J* = 11.8 Hz, 1H), 2.46 – 2.32 (m, 2H), 1.43 – 1.30 (m, 2H), 1.24 – 1.15 (m, 14H), 0.75 – 0.72 (m, 3H), 0.55 – 0.51 (m, 3H); **^{13}C NMR** (101 MHz, $CDCl_3$) δ 174.41, 142.60, 135.34, 132.07, 129.88, 129.06, 127.79, 122.45, 117.24, 83.93, 76.74, 48.57, 37.57, 26.07, 25.99, 24.68, 24.67, 9.60, 9.15; **HRMS** (ESI) *m/z* calcd for $C_{23}H_{35}BBrO_4$ $(M+H)^+$ 465.1806, found 465.1815.

***syn*-Pentan-3-yl-2-((4,4,5,5-tetramethyl-1,3,2-dioxaborolan-2-yl)(3-(trifluoromethoxy)phenyl)met hyl)pent-4-enoate (8d)**

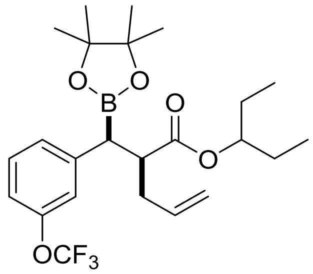

Following the General Procedure C using **5d** (0.25 mmol, 107.6 mg) and allyl bromide (0.375 mmol, 32.5 μL). Analysis of the crude ^{1}H NMR indicated 10:1 diastereoselectivity. Purification by silica gel chromatography gave 81.1 mg (69% yield) of title compound as a colorless oil.

^{1}H NMR (400 MHz, $CDCl_3$) δ 7.24 – 7.15 (m, 3H), 6.97 (d, *J* = 8.0 Hz, 1H), 5.86 – 5.76 (m, 1H), 5.09 (d, *J* = 17.0 Hz, 1H), 5.03 (d, *J* = 10.1 Hz, 1H), 4.50 – 4.44 (m, 1H), 3.09 – 3.02 (m, 1H), 2.67 (d, *J* = 11.8 Hz, 1H), 2.46 – 2.34 (m, 2H), 1.41 – 1.29 (m, 2H), 1.21 – 1.08 (m, 14H), 0.73 – 0.70 (m, 3H), 0.49 – 0.45 (m, 3H); **^{13}C NMR** (101 MHz, $CDCl_3$) δ 174.41, 149.33 (q, *J* = 1.8 Hz), 142.59, 135.29, 129.59, 127.82, 121.57, 120.63 (q, *J* = 256.6 Hz), 118.53, 117.32, 83.98, 76.78, 48.55, 37.62, 26.06, 25.95, 24.67, 24.61, 9.56, 9.05; **HRMS** (ESI) *m/z* calcd for $C_{24}H_{35}BF_3O_5$ $(M+H)^+$ 471.2524, found 471.2530.

***syn*-Pentan-3-yl-2-((4-methoxyphenyl)(4,4,5,5-tetramethyl-1,3,2-dioxaborolan-2-yl)methyl)pent-4-enoate (8e)**

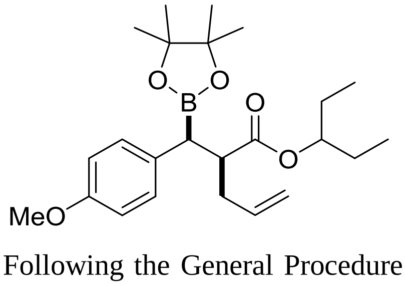

Following the General Procedure C using **5e** (0.25 mmol, 94.1 mg) and allyl bromide (0.375 mmol, 32.5 μL). Analysis of the crude ^{1}H NMR indicated 6.3:1 diastereoselectivity. Purification by silica gel chromatography gave 73.9 mg (71% yield) of title compound as a colorless oil.

^{1}H NMR (400 MHz, $CDCl_3$) δ 7.14 (d, *J* = 8.7 Hz, 2H), 6.75 (d, *J* = 8.7 Hz, 2H), 5.86 – 5.76 (m, 1H), 5.08 (dd, *J* = 17.0, 1.4 Hz, 1H), 5.00 (d, *J* = 10.1 Hz, 1H), 4.50 – 4.44 (m, 1H), 3.74 (s, 3H), 3.04 – 2.98 (m, 1H), 2.58 (d, *J* = 11.8 Hz, 1H), 2.44 – 2.29 (m, 2H), 1.42 – 1.30 (m, 2H), 1.21 – 1.09 (m, 14H), 0.75 – 0.71 (m, 3H), 0.52 – 0.48 (m, 3H); **^{13}C NMR** (101 MHz, $CDCl_3$) δ 174.82, 157.94, 135.71, 131.97, 130.03, 116.89, 113.83, 83.63, 76.37, 55.31,

49.00, 37.64, 26.04, 25.94, 24.73, 24.64, 9.61, 9.09; **HRMS** (ESI) *m/z* calcd for $C_{24}H_{38}BO_5$ $(M+H)^+$ 417.2807, found 417.2808.

***syn*-Pentan-3-yl-2-((4,4,5,5-tetramethyl-1,3,2-dioxaborolan-2-yl)(o-tolyl)methyl)pent-4-enoate (8f)**

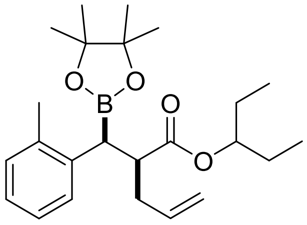

Following the General Procedure C using **5f** (0.25 mmol, 90.1 mg) and allyl bromide (0.375 mmol, 32.5 μL). Analysis of the crude ^{1}H NMR indicated >20:1 diastereoselectivity. Purification by silica gel chromatography gave 87.1 mg (87% yield) of title compound as a colorless oil.

^{1}H NMR (400 MHz, $CDCl_3$) δ 7.26 – 7.24 (m, 1H), 7.06 – 6.95 (m, 3H), 5.89 – 5.79 (m, 1H), 5.09 (dd, *J* = 17.0, 1.3 Hz, 1H), 5.01 (d, *J* = 10.1 Hz, 1H), 4.47 – 4.41 (m, 1H), 3.20 – 3.13 (m, 1H), 2.91 (d, *J* = 11.8 Hz, 1H), 2.45 – 2.41 (m, 2H), 2.36 (s, 3H), 1.35 – 1.23 (m, 2H), 1.19 – 1.08 (m, 14H), 0.68 – 0.64 (m, 3H), 0.53 – 0.50 (m, 3H); **^{13}C NMR** (101 MHz, $CDCl_3$) δ 175.02, 138.51, 136.61, 135.72, 130.28, 128.08, 125.82, 125.47, 83.50, 76.45, 48.09, 38.05, 26.04, 25.98, 24.71, 24.51, 20.42, 9.60, 9.20; **HRMS** (ESI) *m/z* calcd for $C_{24}H_{38}BO_4$ $(M+H)^+$ 401.2858, found 401.2862.

***syn*-Pentan-3-yl-2-(naphthalen-1-yl(4,4,5,5-tetramethyl-1,3,2-dioxaborolan-2-yl)methyl)pent-4-enoate (8g)**

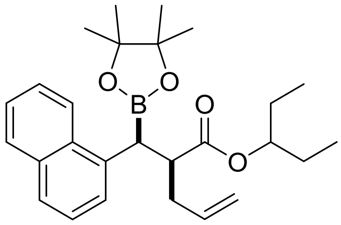

Following the General Procedure C using **5g** (0.25 mmol, 99.1 mg) and allyl bromide (0.375 mmol, 32.5 μL). Analysis of the crude ^{1}H NMR indicated >20:1 diastereoselectivity. Purification by silica gel chromatography gave 75.3 mg (69% yield) of title compound as a colorless oil.

^{1}H NMR (400 MHz, $CDCl_3$) δ 8.27 (d, *J* = 8.5 Hz, 1H), 7.77 (d, *J* = 8.0 Hz, 1H), 7.62 (d, *J* = 8.1 Hz, 1H), 7.50 – 7.33 (m, 4H), 5.92 – 5.82 (m, 1H), 5.12 (dd, *J* = 17.0, 1.4 Hz, 1H), 5.03 (d, *J* = 10.6 Hz, 1H), 4.34 – 4.28 (m, 1H), 3.54 – 3.46 (m, 1H), 3.35 – 3.29 (m, 1H), 2.61 – 2.50 (m, 2H), 1.17 (s, 6H), 1.10 (s, 6H), 1.02 – 0.82 (m, 4H), 0.53 – 0.49 (m, 3H), 0.36 – 0.32 (m, 3H); **^{13}C NMR** (101 MHz, $CDCl_3$) δ 174.76, 136.88, 135.75, 134.21, 132.37, 128.56, 126.48, 125.60, 125.48, 125.34, 124.66, 117.05, 83.77, 76.41, 48.47, 37.82, 25.77, 25.73, 24.75, 24.58, 9.40, 8.95; **HRMS** (ESI) *m/z* calcd for $C_{27}H_{38}BO_4$ $(M+H)^+$ 437.2858, found 437.2865.

***syn*-Pentan-3-yl-2-((4,4,5,5-tetramethyl-1,3,2-dioxaborolan-2-yl)(thiophen-2-yl)methyl)pent-4-enoate (8h)**

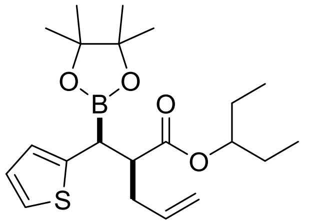

Following the General Procedure C using **5h** (0.25 mmol, 88.1 mg) and allyl bromide (0.375 mmol, 32.5 μL). Analysis of the crude ^{1}H NMR indicated 2.5:1 diastereoselectivity. Purification bysilica gel chromatography gave 60.8 mg (62% yield) of title compound as a colorless oil.

^{1}H NMR (400 MHz, $CDCl_3$) δ 7.05 (d, *J* = 5.0 Hz, 1H), 6.86 – 6.83 (m, 2H), 5.85 – 5.74 (m, 1H), 5.07 (d, *J* = 17.2 Hz, 1H), 5.01 (d, *J* = 10.1 Hz, 1H), 4.61 – 4.55 (m, 1H), 3.04 – 2.97 (m, 2H), 2.47 – 2.32 (m, 2H), 1.46 – 1.39 (m, 2H), 1.35 – 1.29 (m, 2H), 1.24 (s, 6H), 1.21 (s, 6H), 0.78 – 0.75 (m, 3H), 0.64 – 0.61 (m, 3H); **^{13}C NMR** (101 MHz, $CDCl_3$) δ 174.50, 142.26, 135.49, 126.73, 125.66, 123.54, 117.14, 84.00, 76.76, 49.68, 36.93, 26.10, 26.06, 24.77, 24.70, 9.63, 9.26; **HRMS** (ESI) *m/z* calcd for $C_{21}H_{34}BO_4S$ $(M+H)^+$ 393.2265, found 393.2273.

***syn*-Pentan-3-yl-2-(1-(4,4,5,5-tetramethyl-1,3,2-dioxaborolan-2-yl)ethyl)pent-4-enoate (8i)**

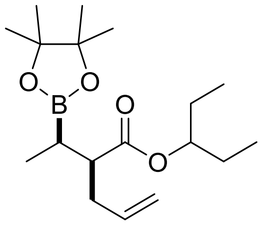

Following the General Procedure C using **5i** (0.25 mmol, 71.1 mg) and allyl bromide (0.375 mmol, 32.5 μL). Analysis of the crude ^{1}H NMR indicated 3.8:1 diastereoselectivity. Purification by silica gel chromatography gave 50.3 mg (62% yield) of title compound as a colorless oil.

^{1}H NMR (400 MHz, $CDCl_3$) δ 5.82 – 5.72 (m, 1H), 5.08 – 4.96 (m, 2H), 4.77 – 4.71 (m, 1H), 2.60 – 2.54 (m, 1H), 2.44 – 2.22 (m, 2H), 1.59 – 1.51 (m, 4H), 1.37 – 1.29 (m, 1H), 1.26 – 1.23 (m, 12H), 0.96 (d, *J* = 7.4 Hz, 2H), 0.90 – 0.85 (m, 6H); **^{13}C NMR** (101 MHz, $CDCl_3$) δ 175.36, 136.31, 116.50, 83.30, 76.67, 48.24, 36.38, 26.42, 26.36, 24.90, 24.83, 13.03, 9.79, 9.70; **HRMS** (ESI) *m/z* calcd for $C_{18}H_{34}BO_4$ $(M+H)^+$ 325.2545, found 325.2547.

***syn*-Pentan-3-yl-2-allyl-3-(4,4,5,5-tetramethyl-1,3,2-dioxaborolan-2-yl)hexanoate (8j)**

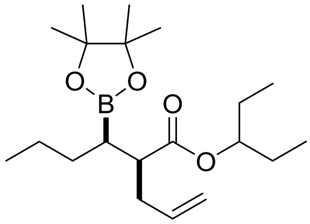

Following the General Procedure C using **5j** (0.25 mmol, 78.1 mg) and allyl bromide (0.375 mmol, 32.5 μL). Analysis of the crude ^{1}H NMR indicated 9:1 diastereoselectivity. Purification by silica gel chromatography gave 77.5 mg (88% yield) of title compound as a colorless oil.

^{1}H NMR (400 MHz, $CDCl_3$) δ 5.81 – 5.71 (m, 1H), 5.03 (d, *J* = 17.1 Hz, 1H), 4.97 (d, *J* = 10.1 Hz, 1H), 4.76 – 4.70 (m, 1H), 2.59 – 2.53 (m, 1H), 2.43 – 2.32 (m, 1H), 2.26 – 2.20 (m, 1H), 1.59 – 1.50 (m, 4H), 1.46 – 1.17 (m,

17H), 0.90 – 0.85 (m, 9H); **^{13}C NMR** (101 MHz, $CDCl_3$) δ 175.55, 136.17, 116.46, 83.35, 76.63, 47.84, 36.80, 32.01, 26.33, 26.26, 25.07, 24.95, 22.41, 14.50, 9.73, 9.66; **HRMS** (ESI) *m/z* calcd for $C_{20}H_{38}BO_4$ $(M+H)^+$ 353.2858, found 353.2859.

***syn*-Pentan-3-yl-2-(3-phenyl-1-(4,4,5,5-tetramethyl-1,3,2-dioxaborolan-2-yl)propyl)pent-4-enoate (8k)**

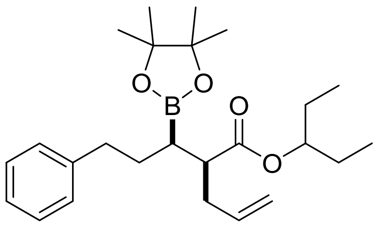

Following the General Procedure C using **5k** (0.25 mmol, 93.6 mg) and allyl bromide (0.375 mmol, 32.5 µL). Analysis of the crude ^{1}H NMR indicated >20:1 diastereoselectivity. Purification by silica gel chromatography gave 70.4 mg (68% yield) of title compound as a colorless oil.

^{1}H NMR (400 MHz, $CDCl_3$) δ 7.26 – 7.13 (m, 5H), 5.80 – 5.70 (m, 1H), 5.02 (d, J = 17.1 Hz, 1H), 4.97 (d, J = 10.1 Hz, 1H), 4.74 – 4.68 (m, 1H), 2.72 – 2.22 (m, 5H), 1.80 – 1.60 (m, 2H), 1.56 – 1.39 (m, 5H), 1.31 – 1.28 (m, 12H), 0.87 – 0.83 (m, 6H); **^{13}C NMR** (101 MHz, $CDCl_3$) δ 175.26, 142.63, 136.08, 128.43, 128.36, 125.79, 116.59, 83.50, 76.74, 47.60, 36.48, 35.54, 31.48, 26.32, 26.22, 25.07, 25.06, 9.74, 9.70; **HRMS** (ESI) *m/z* calcd for $C_{25}H_{40}BO_4$ $(M+H)^+$ 415.3014, found 415.3016.

***syn*-Pentan-3-yl-2-(2-methyl-1-(4,4,5,5-tetramethyl-1,3,2-dioxaborolan-2-yl)propyl)pent-4-enoate (8l)**

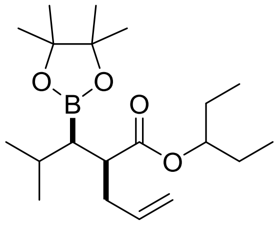

Following the General Procedure C using **5l** (0.25 mmol, 78.1 mg) and allyl bromide (0.375 mmol, 32.5 µL). Analysis of the crude ^{1}H NMR indicated >20:1 diastereoselectivity. Purification by silica gel chromatography gave 72.2 mg (82% yield) of title compound as a colorless oil.

^{1}H NMR (400 MHz, $CDCl_3$) δ 5.81 – 5.71 (m, 1H), 5.03 (ddd, J = 17.1, 3.1, 1.6 Hz, 1H), 4.98 – 4.94 (m, 1H), 4.74 – 4.68 (m, 1H), 2.73 – 2.67 (m, 1H), 2.35 – 2.18 (m, 2H), 1.72 – 1.65 (m, 1H), 1.59 – 1.50 (m, 1H), 1.37 (dd, J = 11.1, 4.8 Hz, 1H), 1.29 – 1.25 (m, 12H), 0.96 – 0.95 (m, 6H), 0.89 – 0.84 (m, 6H); **^{13}C NMR** (101 MHz, $CDCl_3$) δ 175.79, 136.11, 116.44, 83.40, 76.54, 46.37, 37.20, 28.32, 26.26, 26.15, 25.33, 24.98, 23.49, 19.95, 9.64, 9.62; **HRMS** (ESI) *m/z* calcd for $C_{20}H_{38}BO_4$ $(M+H)^+$ 353.2858, found 353.2862.

***syn*-Pentan-3-yl-2-(cyclohexyl(4,4,5,5-tetramethyl-1,3,2-dioxaborolan-2-yl)methyl)pent-4-enoate (8m)**

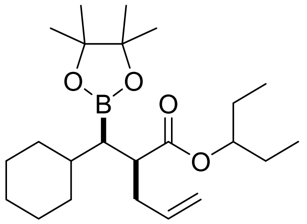

Following the General Procedure C using **5m** (0.25 mmol, 88.1 mg) and allyl bromide (0.375 mmol, 32.5 μL). Analysis of the crude ^{1}H NMR indicated >20:1 diastereoselectivity. Purification by silica gel chromatography gave 64.7 mg (66% yield) of title compound as a colorless oil.

^{1}H NMR (400 MHz, $CDCl_3$) δ 5.81 – 5.70 (m, 1H), 5.02 (d, *J* = 17.1 Hz, 1H), 4.95 (d, *J* = 10.1 Hz, 1H), 4.77 – 4.68 (m, 1H), 2.78 – 2.71 (m, 1H), 2.33 – 2.17 (m, 2H), 1.70 – 1.48 (m, 10H), 1.30 – 1.24 (m, 12H), 1.19 – 0.81 (m, 12H); **^{13}C NMR** (101 MHz, $CDCl_3$) δ 175.86, 136.13, 116.41, 83.39, 76.45, 45.58, 38.42, 37.32, 33.95, 30.72, 27.01, 26.87, 26.71, 26.33, 26.08, 25.34, 24.97, 9.69, 9.63; **HRMS** (ESI) *m/z* calcd for $C_{23}H_{42}BO_4$ $(M+H)^+$ 393.3171, found 393.3172.

***syn*-Pentan-3-yl-2-(cyclopropyl(4,4,5,5-tetramethyl-1,3,2-dioxaborolan-2-yl)methyl)pent-4-enoate (8n)**

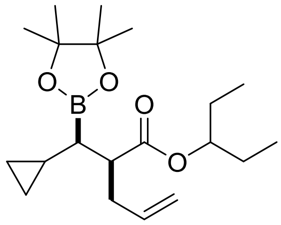

Following the General Procedure C using **5n** (0.25 mmol, 77.6 mg) and allyl bromide (0.375 mmol, 32.5 μL). Analysis of the crude ^{1}H NMR indicated 6.5:1 diastereoselectivity. Purification by silica gel chromatography gave 68.3 mg (78% yield) of title compound as a colorless oil.

^{1}H NMR (400 MHz, $CDCl_3$) δ 5.82 – 5.71 (m, 1H), 5.04 (d, *J* = 17.1 Hz, 1H), 4.97 (d, *J* = 10.0 Hz, 1H), 4.75 – 4.69 (m, 1H), 2.71 – 2.65 (m, 1H), 2.40 – 2.32 (m, 1H), 2.24 – 2.18 (m, 1H), 1.61 – 1.52 (m, 4H), 1.32 – 1.19 (m, 12H), 0.90 – 0.86 (m, 6H), 0.76 – 0.64 (m, 2H), 0.45 – 0.34 (m, 2H), 0.17 – 0.07 (m, 2H); **^{13}C NMR** (101 MHz, $CDCl_3$) δ 175.68, 136.12, 116.52, 83.37, 76.58, 48.09, 37.06, 26.04, 25.01, 24.71, 10.86, 9.67, 9.60, 6.34, 3.02; **HRMS** (ESI) *m/z* calcd for $C_{20}H_{36}BO_4$ $(M+H)^+$ 351.2701, found 351.2707.

***syn*-Pentan-3-yl 2-allyl-5-methyl-3-(4,4,5,5-tetramethyl-1,3,2-dioxaborolan-2-yl)hex-4-enoate (8p)**

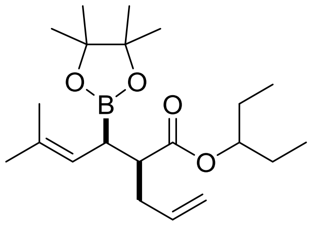

Following the General Procedure C using **5p** (0.25 mmol, 81.1 mg) and allyl bromide (0.375 mmol, 32.5 μL). Analysis of the crude 1H NMR indicated 2.4:1 diastereoselectivity. Purification by silica gel chromatography gave 65.6 mg (72% yield) of title compound as a colorless oil.

^{1}H NMR (400 MHz, $CDCl_3$) δ 5.83 – 5.70 (m, 1H), 5.07 – 4.97 (m, 3H), 4.73 – 4.63 (m, 1H), 2.72 – 2.66 (m, 1H), 2.41 – 2.16 (m, 3H), 1.64 (d, *J* = 0.7 Hz, 3H), 1.60 (d, *J* = 1.0 Hz, 3H), 1.54 – 1.46 (m, 4H), 1.23 (s, 6H), 1.21 (s, 6H), 0.87 – 0.82 (m, 6H); **^{13}C NMR** (101 MHz, $CDCl_3$) δ 175.33, 136.07, 132.74, 121.91, 116.64, 83.31, 76.51, 47.64, 36.89, 26.44, 26.38, 26.02, 24.89, 24.55, 18.34, 9.66, 9.61. **HRMS** (ESI) *m/z* calcd for $C_{21}H_{38}BO_4$ $(M+H)^+$ 365.2858, found 365.2852.

***syn*-Pentan-3-yl (*E*)-2-allyl-3-(4,4,5,5-tetramethyl-1,3,2-dioxaborolan-2-yl)hex-4-enoate (8o)**

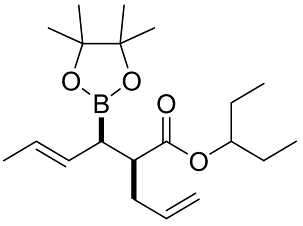

Following the General Procedure C using *race* **5o** (2.0 mmol, 620.4 mg) and allyl bromide (3.0 mmol, 260 μL). Analysis of the crude ^{1}H NMR indicated 2:1 diastereoselectivity. Purification bysilica gel chromatography gave 483.4 mg (69% yield) of title compound as a colorless oil.

^{1}H NMR (400 MHz, $CDCl_3$) δ 5.81 – 5.71 (m, 1H), 5.49 – 5.31 (m, 2H), 5.07 – 4.96 (m, 2H), 4.73 – 4.67 (m, 1H), 2.72 – 2.66 (m, 1H), 2.39 – 2.32 (m, 1H), 2.23 – 2.17 (m, 1H), 2.08 – 2.04 (m, 1H), 1.61 – 1.59 (m, 3H), 1.55 – 1.48 (m, 4H), 1.24 (s, 6H), 1.23 (s, 6H), 0.88 – 0.84 (m, 6H); **^{13}C NMR** (101 MHz, $CDCl_3$) δ 175.05, 136.02, 128.41, 126.96, 116.70, 83.49, 76.58, 47.41, 36.65, 26.38, 26.35, 24.90, 24.69, 18.27, 9.71, 9.6. **HRMS** (ESI) *m/z* calcd for $C_{20}H_{35}BKO_4$ $(M+K)^+$ 389.2260, found 423.2261.

***anti-tert*-Butyl-2-(phenyl(4,4,5,5-tetramethyl-1,3,2-dioxaborolan-2-yl)methyl)pent-4-enoate (3c)**

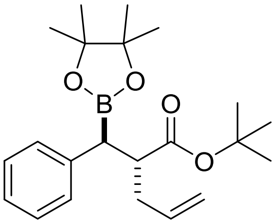

Following the General Procedure D using **1c** (0.25 mmol, 83.1 mg) and allyl bromide (0.375 mmol, 32.5 μL). Analysis of the crude ^{1}H NMR indicated >20:1 diastereoselectivity. Purification by silica gel chromatography gave 65.1 mg (70% yield) of title compound as a white solid. **m.p.** = 46 – 47 ℃.

^{1}H NMR (400 MHz, $CDCl_3$) δ 7.26 – 7.12 (m, 5H), 5.72 – 5.61 (m, 1H), 4.98 (dd, *J* = 10.1, 0.8 Hz, 1H), 4.92 (dd, *J* = 17.1, 1.5 Hz, 1H), 2.97 – 2.92 (m, 1H), 2.54 (d, *J* = 12.0 Hz, 1H), 2.33 – 2.27 (m, 1H), 2.04 – 1.97 (m, 1H), 1.47 (s, 9H), 1.18 (s, 6H), 1.12 (s, 6H); **^{13}C NMR** (101 MHz, $CDCl_3$) δ 175.13, 140.26, 134.75, 129.24, 128.53, 125.72, 117.26, 83.39, 80.47, 47.86, 34.53, 28.32, 24.70, 24.54; **HRMS** (ESI) *m/z* calcd for $C_{22}H_{34}BO_4$ $(M+H)^+$ 373.2545, found 373.2553.

***anti-tert*-Butyl-2-((4-fluorophenyl)(4,4,5,5-tetramethyl-1,3,2-dioxaborolan-2-yl)methyl)pent-4-eno ate (7a)**

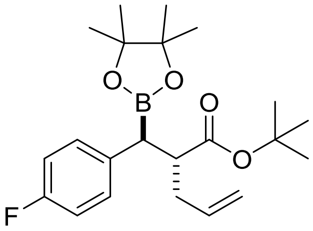

Following the General Procedure D using **5a′** (0.25 mmol, 87.6 mg) and allyl bromide (0.375 mmol, 32.5 μL). Analysis of the crude ^{1}H NMR indicated 10:1 diastereoselectivity. Purification by silica gel chromatography gave 71.2 mg (73% yield) of title compound as a white solid. **m.p.** = 62 – 64 ℃.

^{1}H NMR (400 MHz, $CDCl_3$) δ 7.18 – 7.13 (m, 2H), 6.97 – 6.91 (m, 2H), 5.70 – 5.59 (m, 1H), 4.99 (d, *J* = 10.0 Hz, 1H), 4.92 (d, *J* = 17.0 Hz, 1H), 2.92 – 2.86 (m, 1H), 2.52 (d, *J* = 12.0 Hz, 1H), 2.33 – 2.27 (m, 1H), 2.02 – 1.95 (m, 1H), 1.46 (s, 9H), 1.18 (s, 6H), 1.12 (s, 6H); **^{13}C NMR** (101 MHz, $CDCl_3$) δ 174.99, 161.30 (d, *J* = 243.2 Hz), 135.84 (d, *J* = 3.1 Hz), 134.53, 130.49 (d, *J* = 7.7 Hz), 117.46, 115.33 (d, *J* = 21.0 Hz), 83.47, 80.62, 47.94, 34.36, 28.29, 24.68, 24.55; **HRMS** (ESI) *m/z* calcd for $C_{22}H_{33}BFO_4$ $(M+H)^+$ 391.2450, found 391.2459.

***anti*-*tert*-Butyl-2-((3-chlorophenyl)(4,4,5,5-tetramethyl-1,3,2-dioxaborolan-2-yl)methyl)pent-4-eno ate (7b)**

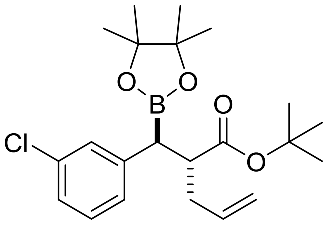

Following the General Procedure D using **5b′** (0.25 mmol, 91.7 mg) and allyl bromide (0.375 mmol, 32.5 μL). Analysis of the crude ^{1}H NMR indicated >20:1 diastereoselectivity. Purification by silica gel chromatography gave 83.4 mg (82% yield) of title compound as a colorless oil.

^{1}H NMR (400 MHz, $CDCl_3$) δ 7.22 – 7.08 (m, 4H), 5.70 – 5.59 (m, 1H), 5.00 (d, *J* = 9.5 Hz, 1H), 4.93 (dd, *J* = 17.0, 1.3 Hz, 1H), 2.95 – 2.89 (m, 1H), 2.52 (d, *J* = 11.9 Hz, 1H), 2.35 – 2.29 (m, 1H), 2.03 – 1.96 (m, 1H), 1.46 (s, 9H), 1.19 (s, 6H), 1.13 (s, 6H); **^{13}C NMR** (101 MHz, $CDCl_3$) δ 174.85, 142.54, 134.37, 134.29, 129.75, 129.21, 127.45, 126.01, 117.62, 83.58, 80.72, 47.79, 34.43, 28.27, 24.70, 24.50; **HRMS** (ESI) *m/z* calcd for $C_{22}H_{32}BClNaO_4$ $(M+Na)^+$ 429.1974, found 429.1975.

***anti*-*tert*-Butyl-2-((3-bromophenyl)(4,4,5,5-tetramethyl-1,3,2-dioxaborolan-2-yl)methyl)pent-4-enoate (7c)**

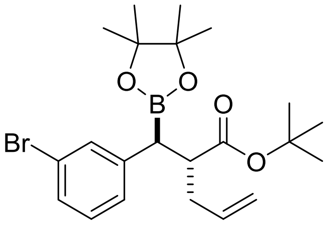

Following the General Procedure D using **5c′** (0.25 mmol, 102.8 mg) and allyl bromide (0.375 mmol, 32.5 μL). Analysis of the crude ^{1}H NMR indicated 11.1:1 diastereoselectivity. Purification by silica gel chromatography gave 91.4 mg (81% yield) of title compound as a colorless oil.

^{1}H NMR (400 MHz, $CDCl_3$) δ 7.38 – 7.10 (m, 4H), 5.69 – 5.59 (m, 1H), 5.01 (d, *J* = 10.0 Hz, 1H), 4.94 (d, *J* = 17.0 Hz, 1H), 2.94 – 2.88 (m, 1H), 2.51 (d, *J* = 11.9 Hz, 1H), 2.35 – 2.29 (m, 1H), 2.04 – 1.96 (m, 1H), 1.46 (s, 9H), 1.19 (s, 6H), 1.13 (s, 6H); **^{13}C NMR** (101 MHz, $CDCl_3$) δ 174.82, 142.90, 134.41, 132.14, 130.06, 128.93, 127.93, 122.65, 117.59, 83.59, 80.72, 47.84, 34.46, 28.28, 24.72, 24.50; **HRMS** (ESI) *m/z* calcd for $C_{22}H_{33}BBrO_4$ $(M+H)^+$ 451.1650, found 451.1653.

***anti*-*tert*-Butyl-2-((4,4,5,5-tetramethyl-1,3,2-dioxaborolan-2-yl)(3-(trifluoromethoxy)phenyl)meth yl)pent-4-enoate (7d)**

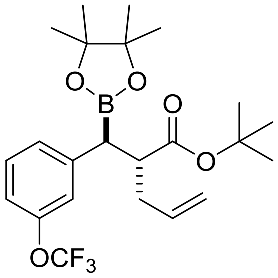

Following the General Procedure D using **5d′** (0.25 mmol, 104.1 mg) and allyl bromide (0.375 mmol, 32.5 μL). Analysis of the crude ^{1}H NMR indicated >20:1 diastereoselectivity. Purification by silica gel chromatography gave 83.3 mg (73% yield) of title compound as a colorless oil.

^{1}H NMR (400 MHz, $CDCl_3$) δ 7.29 – 7.10 (m, 3H), 7.01 (d, *J* = 8.2 Hz, 1H), 5.69 – 5.58 (m, 1H), 5.00 (d, *J* = 10.1 Hz, 1H), 4.92 (dd, *J* = 17.0, 1.3 Hz, 1H), 2.96 – 2.91 (m, 1H), 2.58 (d, *J* = 11.9 Hz, 1H), 2.37 – 2.31 (m, 1H), 2.03 – 1.95 (m, 1H), 1.47 (s, 9H), 1.19 (s, 6H), 1.12 (s, 6H); **^{13}C NMR** (101 MHz, $CDCl_3$) δ 174.82, 149.51 (q, *J* = 1.9 Hz), 142.87, 134.27, 129.75, 128.00, 121.47, 120.63 (q, *J* = 256.6 Hz) 118.41, 117.70, 83.60, 80.80, 47.82, 34.34, 28.26, 24.63, 24.50; **HRMS** (ESI) *m/z* calcd for $C_{23}H_{33}BF_3O_5$ $(M+H)^+$ 457.2368, found 457.2370.

***anti*-*tert*-Butyl-2-((4-methoxyphenyl)(4,4,5,5-tetramethyl-1,3,2-dioxaborolan-2-yl)methyl)pent-4-enoate (7e)**

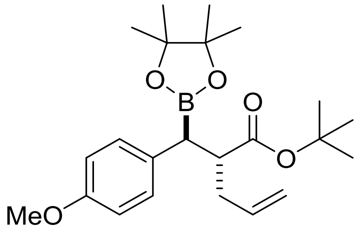

Following the General Procedure D using **5e′** (0.25 mmol, 90.7 mg) and allyl bromide (0.375 mmol, 32.5 μL). Analysis of the crude ^{1}H NMR indicated >20:1 diastereoselectivity. Purification by silica gel chromatography gave 75.4 mg (75% yield) of title compound as a white solid. **m.p.** = 73 – 74 ℃.

^{1}H NMR (400 MHz, $CDCl_3$) δ 7.11 (d, 2H), 6.80 (d, *J* = 8.4 Hz, 2H), 5.71 – 5.61(m, 1H), 4.97 (d, *J* = 10.1 Hz, 1H), 4.93 (d, *J* = 17.1 Hz, 1H), 3.78 (s, 3H), 2.90 – 2.84 (m, 1H),2.47 (d, *J* = 12.0 Hz, 1H), 2.32 – 2.26 (m, 1H), 2.04 – 1.97 (m, 1H), 1.46 (s, 9H), 1.19 (s, 6H),1.12 (s, 6H); **^{13}C NMR** (101 MHz, $CDCl_3$) δ 175.18, 157.72, 134.82, 132.06, 130.11, 117.21, 113.95, 83.32, 80.40, 55.26, 48.01, 34.44, 28.30, 24.72, 24.55; **HRMS** (ESI) *m/z* calcd for $C_{23}H_{36}BO_5$ $(M+H)^+$ 403.2650, found 403.2652.

***anti-tert*-Butyl-2-((4,4,5,5-tetramethyl-1,3,2-dioxaborolan-2-yl)(o-tolyl)methyl)pent-4-enoate (7f)**

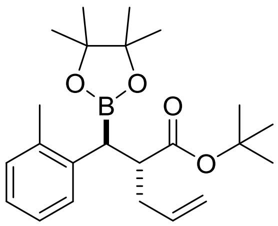

Following the General Procedure D using **5f′** (0.25 mmol, 86.6 mg) and allyl bromide (0.375 mmol, 32.5 μL). Analysis of the crude ^{1}H NMR indicated 4.4:1 diastereoselectivity. Purification by silica gel chromatography gave 73.4 mg (76% yield) of title compound as a colorless oil.

^{1}H NMR (400 MHz, $CDCl_3$) δ 7.20 – 7.01 (m, 4H), 5.70 – 5.60 (m, 1H), 4.93 (d, *J* = 10.0 Hz, 1H), 4.85 (d, *J* = 17.0 Hz, 1H), 3.10 – 3.04 (m, 1H), 2.80 (d, *J* = 12.0 Hz, 1H), 2.35 (s, 3H), 2.31 – 2.25 (m, 1H), 2.06 – 1.99 (m, 1H), 1.47 (s, 9H), 1.14 (s, 6H), 1.08 (s, 6H); **^{13}C NMR** (101 MHz, $CDCl_3$) δ 175.43, 138.48, 137.27, 135.31, 130.42, 127.74, 125.96, 125.25, 116.94, 83.21, 80.46, 47.68, 34.82, 28.34, 24.60, 24.54, 20.65; **HRMS** (ESI) *m/z* calcd for $C_{23}H_{36}BO_4$ $(M+H)^+$ 387.2701, found 423.2702.

***anti-tert*-Butyl-2-(naphthalen-1-yl(4,4,5,5-tetramethyl-1,3,2-dioxaborolan-2-yl)methyl)pent-4-enoate (7g)**

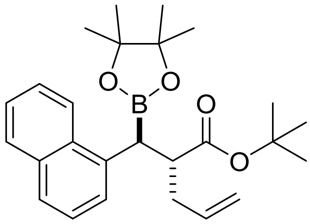

Following the General Procedure D using **5g′** (0.25 mmol, 95.6 mg) and allyl bromide (0.375 mmol, 32.5 μL). Analysis of the crude ^{1}H NMR indicated 11:1 diastereoselectivity. Purification by silica gel chromatography gave 66.5 mg (63% yield) of title compound as a colorless oil.

^{1}H NMR (400 MHz, $CDCl_3$) δ 8.27 (d, *J* = 8.2 Hz, 1H), 7.83 – 7.81 (m, 1H), 7.68 (d, *J* = 7.9 Hz, 1H), 7.50 – 7.37 (m, 4H), 5.70 – 5.59 (m, 1H), 4.89 (d, *J* = 10.1 Hz, 1H), 4.77 (d, *J* = 17.0 Hz, 1H), 3.35 – 3.17 (m, 2H), 2.18 – 1.92 (m, 2H), 1.50 (s, 9H), 1.17 (s, 6H), 1.07 (s, 6H); **^{13}C NMR** (101 MHz, $CDCl_3$) δ 175.28, 136.94, 135.12, 134.25, 132.90, 128.77, 126.40, 125.63, 125.47, 124.77, 117.12, 83.54, 80.53, 28.38, 24.74, 24.50; **HRMS** (ESI) *m/z* calcd for $C_{26}H_{36}BO_4$ $(M+H)^+$ 423.2701, found 423.2707.

***anti-tert*-Butyl-2-((4,4,5,5-tetramethyl-1,3,2-dioxaborolan-2-yl)(thiophen-2-yl)methyl)pent-4-enoate (7h)**

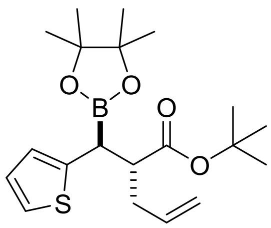

Following the General Procedure D using **5h′** (0.25 mmol, 84.6 mg) and allyl bromide (0.375 mmol, 32.5 μL). Analysis of the crude ^{1}H NMR indicated 14.3:1 diastereoselectivity. Purification by silica gel chromatography gave 61.5 mg (65% yield) of title compound as a colorless oil.

^{1}H NMR (400 MHz, $CDCl_3$) δ 7.10 (d, *J* = 5.0 Hz, 1H), 6.92 – 6.81 (m, 2H), 5.72 – 5.62 (m, 1H), 5.03 – 4.98 (m, 2H), 2.92 – 2.85 (m, 2H), 2.42 – 2.38 (m, 1H), 2.18 – 2.11 (m, 1H), 1.46 (s, 9H), 1.22 (s, 6H), 1.16 (s, 6H); **^{13}C NMR** (101 MHz, $CDCl_3$) δ 174.69, 142.93, 134.42, 126.96, 125.50, 123.39, 117.70, 83.68, 80.71, 48.84, 34.32, 28.28, 24.72, 24.58; **HRMS** (ESI) *m/z* calcd for $C_{20}H_{32}BO_4S$ $(M+H)^+$ 379.2109, found 379.2111.

***anti-tert*-Butyl-2-(1-(4,4,5,5-tetramethyl-1,3,2-dioxaborolan-2-yl)ethyl)pent-4-enoate (7i)**

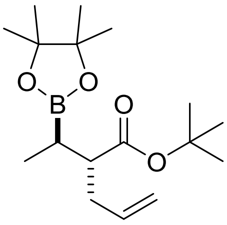

Following the General Procedure D using **5i′** (0.25 mmol, 67.6 mg) and allyl bromide (0.375 mmol, 32.5 μL). Analysis of the crude ^{1}H NMR indicated >20:1 diastereoselectivity. Purification by silica gel chromatography gave 61.3 mg (79% yield) of title compound as a colorless oil.

^{1}H NMR (400 MHz, $CDCl_3$) δ 5.79 – 5.68 (m, 1H), 5.07 – 4.97 (m, 3H), 2.45 – 2.30 (m, 3H), 1.43 (s, 9H), 1.33 – 1.23 (m, 13H), 0.95 (d, *J* = 7.5 Hz, 3H); **^{13}C NMR** (101 MHz, $CDCl_3$) δ 175.25, 136.00, 116.51, 83.07, 80.10, 48.08, 34.22, 28.28, 24.90, 24.81, 12.61; **HRMS** (ESI) *m/z* calcd for $C_{17}H_{32}BO_4$ $(M+H)^+$ 311.2388, found 311.2396.

***anti-tert*-Butyl-2-allyl-3-(4,4,5,5-tetramethyl-1,3,2-dioxaborolan-2-yl)hexanoate (7j)**

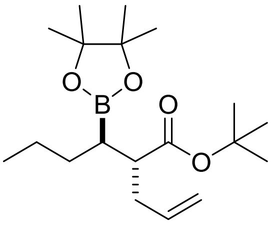

Following the General Procedure D using **5j′** (0.25 mmol, 74.6 mg) and allyl bromide (0.375 mmol, 32.5 μL). Analysis of the crude ^{1}H NMR indicated >20:1 diastereoselectivity. Purification by silica gel chromatography gave 72.7 mg (86% yield) of title compound as a colorless oil.

^{1}H NMR (400 MHz, $CDCl_3$) δ 5.79 – 5.69 (m, 1H), 5.04 (ddd, *J* = 17.1, 3.3, 1.5 Hz, 1H), 5.00 – 4.97 (m, 1H), 2.49 – 2.31 (m, 3H), 1.43 – 1.20 (m, 26H), 0.90 – 0.86 (m, 3H); **^{13}C NMR** (101 MHz, $CDCl_3$) δ 175.26, 136.10, 116.46, 83.06, 80.10, 46.87, 34.77, 30.74, 28.30, 25.11, 24.84, 22.20, 14.59; **HRMS** (ESI) *m/z* calcd for $C_{19}H_{36}BO_4$ $(M+H)^+$ 339.2701, found 339.2704.

***anti-tert*-Butyl-2-(3-phenyl-1-(4,4,5,5-tetramethyl-1,3,2-dioxaborolan-2-yl)propyl)pent-4-enoate (7k)**

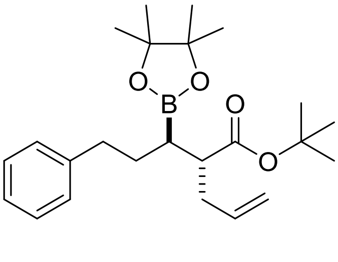

Following the General Procedure D using **5k′** (0.25 mmol, 90.1 mg) and allyl bromide (0.375 mmol, 32.5 μL). Analysis of the crude ^{1}H NMR indicated >20:1 diastereoselectivity. Purification by silica gel chromatography gave 81.1 mg (81% yield) of title compound as a colorless oil.

^{1}H NMR (400 MHz, $CDCl_3$) δ 7.28 – 7.14 (m, 5H), 5.75 – 5.65 (m, 1H), 5.03 – 4.95 (m, 2H), 2.72 – 2.30 (m, 5H), 1.79 – 1.66 (m, 2H), 1.42 (s, 9H), 1.31 – 1.27 (m, 13H); **^{13}C NMR** (101 MHz, $CDCl_3$) δ 175.11, 142.95, 135.92, 128.55, 128.38, 125.74, 116.60, 83.21, 80.25, 46.83, 35.36, 34.76, 30.66, 28.29, 25.19, 24.89; **HRMS** (ESI) *m/z* calcd for $C_{24}H_{38}BO_4$ $(M+H)^+$ 401.2858, found 401.2864.

***anti-tert*-Butyl-2-(2-methyl-1-(4,4,5,5-tetramethyl-1,3,2-dioxaborolan-2-yl)propyl)pent-4-enoate (7l)**

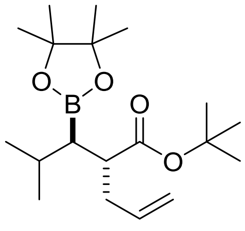

Following the General Procedure D using **5l′** (0.25 mmol, 74.6 mg) and allyl bromide (0.375 mmol, 32.5 μL). Analysis of the crude ^{1}H NMR indicated >20:1 diastereoselectivity. Purification by silica gel chromatography gave 68.5 mg (81% yield) of title compound as a colorless oil.

^{1}H NMR (400 MHz, $CDCl_3$) δ 5.76 – 5.65 (m, 1H), 5.06 – 4.97 (m, 2H), 2.63 – 2.58 (m, 1H), 2.44 – 2.31 (m, 2H), 1.91 – 1.79 (m, 1H), 1.43 (s, 9H), 1.25 (s, 6H), 1.23 (s, 6H), 1.16 (dd, *J* = 4.2 Hz, 1H), 1.00 (d, *J* = 6.8 Hz, 3H), 0.88 (d, *J* = 6.8 Hz, 3H); **^{13}C NMR** (101 MHz, $CDCl_3$) δ 175.49, 135.49, 116.72, 83.06, 80.05, 44.71, 34.70, 28.31, 26.17, 25.25, 24.97, 23.24, 20.37; **HRMS** (ESI) *m/z* calcd for $C_{19}H_{36}BO_4$ $(M+H)^+$ 339.2701, found 339.2706.

***anti-tert*-Butyl-2-(cyclohexyl(4,4,5,5-tetramethyl-1,3,2-dioxaborolan-2-yl)methyl)pent-4-enoate (7m)**

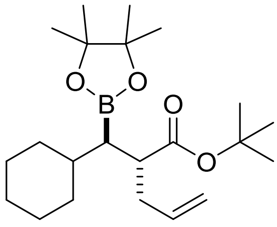

Following the General Procedure D using **5m′** (0.25 mmol, 84.6 mg) and allyl bromide (0.375 mmol, 32.5 μL). Analysis of the crude ^{1}H NMR indicated >20:1 diastereoselectivity. Purification by silica gel chromatography gave 83.2 mg (88% yield) of title compound as a colorless oil.

^{1}H NMR (400 MHz, $CDCl_3$) δ 5.76 – 5.65 (m, 1H), 5.06 – 4.97 (m, 2H), 2.68 – 2.63 (m, 1H), 2.43 – 2.28 (m, 2H), 1.73 – 1.63 (m, 5H), 1.52 – 1.43 (m, 11H), 1.25 (s, 6H), 1.23 (s, 6H), 1.21 – 1.06 (m, 5H); **^{13}C NMR** (101 MHz, $CDCl_3$) δ 175.55, 135.51, 116.70, 83.06, 80.01, 43.74, 36.30, 34.68, 33.71, 31.04, 28.32, 27.17, 26.98, 26.91, 25.24, 24.97; **HRMS** (ESI) *m/z* calcd for $C_{22}H_{40}BO_4$ $(M+H)^+$ 379.3014, found 379.3018.

***anti-tert*-Butyl-2-(cyclopropyl(4,4,5,5-tetramethyl-1,3,2-dioxaborolan-2-yl)methyl)pent-4-enoate (7n)**

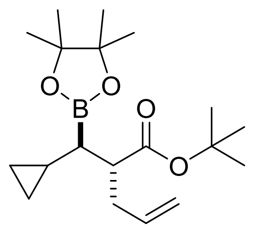

Following the General Procedure D using **5n′** (0.25 mmol, 74.1 mg) and allyl bromide (0.375 mmol, 32.5 μL). Analysis of the crude ^{1}H NMR indicated >20:1 diastereoselectivity. Purification by silica gel chromatography gave 67.3 mg (80% yield) of title compound as a colorless oil.

^{1}H NMR (400 MHz, $CDCl_3$) δ 5.77 – 5.67 (m, 1H), 5.05 (ddd, *J* = 17.1, 3.5, 1.4 Hz, 1H), 5.00 – 4.97 (m, 1H), 2.65 – 2.59 (m, 1H), 2.55 – 2.51 (m, 2H), 1.43 (s, 9H), 1.25 (s, 6H), 1.23 (s, 6H), 0.67 – 0.34 (m, 4H), 0.15 – 0.08 (m, 2H); **^{13}C NMR** (101 MHz, $CDCl_3$) δ 175.17, 135.96, 116.64, 83.07, 80.15, 48.19, 34.80, 28.28, 24.88, 24.85, 10.48, 6.47, 2.81; **HRMS** (ESI) *m/z* calcd for $C_{19}H_{33}BKO_4$ $(M+K)^+$ 375.2103, found 375.2117.

***anti*-Pentan-3-yl-2-allyl-5-methyl-3-(4,4,5,5-tetramethyl-1,3,2-dioxaborolan-2-yl)hex-4-enoate (7p)**

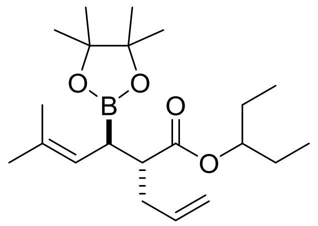

Following the General Procedure D using **5p** (0.25 mmol, 81.1 mg) and allyl bromide (0.375 mmol, 32.5 μL). Analysis of the crude ^{1}H NMR indicated 17.6:1 diastereoselectivity. Purification by silica gel chromatography gave 71.9 mg (79% yield) of title compound as a colorless oil.

^{1}H NMR (400 MHz, $CDCl_3$) δ 5.75 – 5.65 (m, 1H), 5.02 – 4.90 (m, 3H), 4.76 – 4.70 (m, 1H), 2.68 – 2.62 (m, 1H), 2.48 – 2.41 (m, 1H), 2.32 – 2.22 (m, 2H), 1.70 (d, *J* = 0.7 Hz, 3H), 1.62 (d, *J* = 0.9 Hz, 3H), 1.60 – 1.50 (m, 4H), 1.21 (s, 6H), 1.17 (s, 6H), 0.90 – 0.86 (m, 6H); **^{13}C NMR** (101 MHz, $CDCl_3$) δ 175.57, 135.52, 133.03, 122.34, 116.89, 83.03, 76.81, 46.81, 34.80, 26.49, 26.44, 26.11, 24.71, 24.64, 18.53, 9.82, 9.79. **HRMS** (ESI) *m/z* calcd for $C_{21}H_{37}BKO_4$ $(M+K)^+$ 403.2411, found 403.2412.

***anti*-Pentan-3-yl-(*E*)-2-allyl-3-(4,4,5,5-tetramethyl-1,3,2-dioxaborolan-2-yl)hex-4-enoate (7o)**

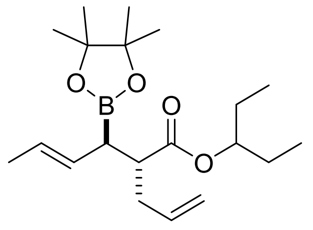

Following the General Procedure D using *race* **5o** (2.0 mmol, 620.4 mg) and allyl bromide (3.0 mmol, 260 μL). Analysis of the crude ^{1}H NMR indicated >20:1 diastereoselectivity. Purification by silica gel chromatography gave 525.5 mg (75% yield) of title compound as a colorless oil.

^{1}H NMR (400 MHz, $CDCl_3$) δ 5.75 – 5.65 (m, 1H), 5.52 – 5.44 (m, 1H), 5.29 – 5.23 (m, 1H), 5.04 – 4.97 (m, 2H), 4.76 – 4.69 (m, 1H), 2.65 (ddd, *J* = 10.1, 7.2, 4.5 Hz, 1H), 2.48 – 2.30 (m, 2H), 2.09 – 2.05 (m, 1H), 1.66 (dd, *J* =

6.4, 1.4 Hz, 3H), 1.58 – 1.51 (m, 4H), 1.22 (s, 6H), 1.20 (s, 6H), 0.91 – 0.86 (m, 6H); ^{13}**C NMR** (101 MHz, $CDCl_3$) δ 175.45, 135.32, 128.67, 127.24, 117.00, 83.25, 76.84, 46.10, 34.43, 26.49, 26.43, 24.78, 24.70, 18.37, 9.85, 9.80; **HRMS** (ESI) *m/z* calcd for $C_{20}H_{35}BKO_4$ $(M+K)^+$ 389.2260, found 389.2261.

***anti-N,N*-Diethyl-2-(phenyl(4,4,5,5-tetramethyl-1,3,2-dioxaborolan-2-yl)methyl)pentanamide (7w)**

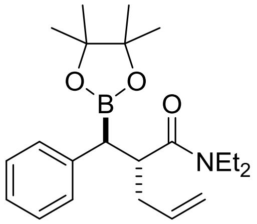

Following the General Procedure E using **5q** (0.25 mmol, 82.8 mg) and allyl bromide (0.375 mmol, 32.5 μL). Analysis of the crude ^{1}H NMR indicated >20:1 diastereoselectivity. Purification by silica gel chromatography gave 67.8 mg (73% yield) of title compound as a colorless oil.

^{1}**H NMR** (400 MHz, $CDCl_3$) δ 7.30 – 7.10 (m, 5H), 5.73 – 5.62 (m, 1H), 4.97 – 4.93 (m, 2H), 3.68 – 3.59 (m, 1H), 3.51 – 3.42 (m, 1H), 3.33 – 3.24 (m, 1H), 3.22 – 3.14 (m, 1H), 3.06 – 3.01 (m, 1H), 2.59 (d, *J* = 9.2 Hz, 1H), 2.28 – 2.15 (m, 2H), 1.28 – 1.24 (m, 3H), 1.16 – 1.13 (m, 9H), 1.09 (s, 6H); ^{13}**C NMR** (101 MHz, $CDCl_3$) δ 175.93, 142.42, 135.20, 129.11, 128.32, 125.27, 117.15, 45.89, 42.43, 40.67, 36.43, 24.78, 24.59, 14.17, 13.14; **HRMS** (ESI) *m/z* calcd for $C_{22}H_{35}BNO_3$ $(M+H)^+$ 372.2705, found 372.2709.

***syn*-Pentan-3-yl-2-methyl-3-phenyl-3-(4,4,5,5-tetramethyl-1,3,2-dioxaborolan-2-yl)propanoate (8q)**

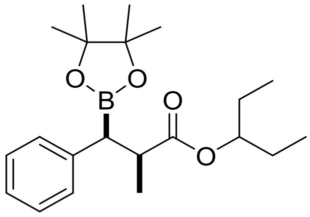

Following the General Procedure C using **1b** (0.25 mmol, 86.6 mg) and iodomethane (0.375 mmol, 23 μL). Analysis of the crude ^{1}H NMR indicated >14.3:1 diastereoselectivity. Purification by silica gel chromatography gave 74.8 mg (83% yield) of title compound as a colorless oil.

^{1}**H NMR** (400 MHz, $CDCl_3$) δ 7.25 – 7.07 (m, 5H), 4.55 – 4.49 (m, 1H), 3.07 – 2.99 (m, 1H), 2.64 (d, *J* = 11.2 Hz, 1H), 1.43 – 1.31 (m, 2H), 1.28 – 1.16 (m, 17H), 0.73 – 0.70 (m, 3H), 0.57 – 0.53 (m, 3H); ^{13}**C NMR** (101 MHz, $CDCl_3$) δ 176.17, 140.58, 129.01, 128.31, 125.77, 83.61, 76.22, 43.13, 26.39, 26.26, 24.73, 24.63, 18.24, 9.57, 9.24; **HRMS** (ESI) *m/z* calcd for $C_{21}H_{34}BO_4$ $(M+H)^+$ 361.2545, found 361.2545.

***syn*-Pentan-3-yl-2-benzyl-3-phenyl-3-(4,4,5,5-tetramethyl-1,3,2-dioxaborolan-2-yl)propanoate (8r)**

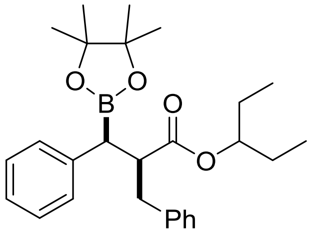

Following the General Procedure C using **1b** (0.25 mmol, 86.6 mg) and benzyl bromide (0.375 mmol, 45 μL). Analysis of the crude ^{1}H NMR indicated 8.3:1diastereoselectivity. Purification by silica gel chromatography gave 89.5 mg (82% yield) of title compound as a white solid. **m.p.** = 83 – 85 ℃.

^{1}H NMR (400 MHz, $CDCl_3$) δ 7.27 – 7.07 (m, 10H), 4.30 – 4.24 (m, 1H), 3.30 – 3.23 (m, 1H), 2.97 (dd, *J* = 13.2, 3.7 Hz, 1H), 2.87 (dd, *J* = 13.1, 11.3 Hz, 1H), 2.70 (d, *J* = 11.8 Hz, 1H), 1.24 (s, 6H), 1.20 (s, 6H), 1.09 – 0.87 (m, 4H), 0.32 – 0.27 (m, 6H); **^{13}C NMR** (101 MHz, $CDCl_3$) δ 174.75, 139.73, 139.66, 129.22, 129.15, 128.37, 126.38, 125.98, 83.80, 76.31, 51.51, 39.84, 25.60, 25.54, 24.84, 24.67, 8.93, 8.90; **HRMS** (ESI) *m/z* calcd for $C_{27}H_{38}BO_4$ $(M+H)^+$ 437.2858, found 437.2866.

***syn*-Pentan-3-yl (*E*)-5-phenyl-2-(phenyl(4,4,5,5-tetramethyl-1,3,2-dioxaborolan-2-yl)methyl)pent-4-enoate (8s)**

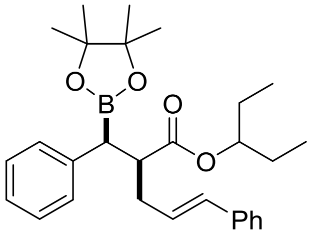

Following the General Procedure C using **1b** (0.25 mmol, 86.6 mg) and cinnamyl bromide (0.375 mmol, 55 μL). Analysis of the crude ^{1}H NMR indicated 7.2:1 diastereoselectivity. Purification by silica gel chromatography gave 95.9 mg (83% yield) of title compound as a white solid. **m.p.** = 75 – 77 ℃.

^{1}H NMR (400 MHz, $CDCl_3$) δ 7.32 – 7.08 (m, 10H), 6.42 (d, *J* = 15.8 Hz, 1H), 6.25 – 6.17 (m, 1H), 4.48 – 4.42 (m, 1H), 3.18 – 3.12 (m, 1H), 2.69 (d, *J* = 11.7 Hz, 1H), 2.62 – 2.52 (m, 2H), 1.32 – 1.24 (m, 2H), 1.22 – 1.07 (m, 14H), 0.61 – 0.58 (m, 3H), 0.47 – 0.43 (m, 3H); **^{13}C NMR** (101 MHz, $CDCl_3$) δ 174.80, 140.00, 137.58, 132.08, 129.14, 128.50, 128.38, 127.41, 127.16, 126.26, 125.93, 83.75, 76.49, 49.13, 36.95, 26.07, 25.91, 24.76, 24.68, 9.46, 9.12; **HRMS** (ESI) *m/z* calcd for $C_{29}H_{40}BO_4$ $(M+H)^+$ 463.3014, found 463.3020.

***syn*-Pentan-3-yl-2-((benzyloxy)methyl)-3-phenyl-3-(4,4,5,5-tetramethyl-1,3,2-dioxaborolan-2-yl)propanoate (8t)**

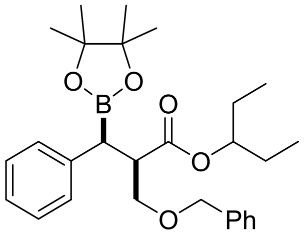

Following the General Procedure C using **1b** (0.25 mmol, 86.6 mg) and benzylchloromethyl ether (0.375 mmol, 52 μL). Analysis of the crude ^{1}H NMR indicated 8.3:1 diastereoselectivity. Purification by silica gel chromatography gave 86.3 mg (74% yield) of title compound as a colorless oil.

^{1}H NMR (400 MHz, $CDCl_3$) δ 7.34 – 7.07 (m, 10H), 4.55 – 4.48 (m, 3H), 3.73 – 3.68 (m, 1H), 3.61 (dd, *J* = 9.0, 5.5 Hz, 1H), 3.38 (ddd, *J* = 11.9, 8.6, 5.5 Hz, 1H), 2.59 (d, *J* = 11.8 Hz, 1H), 1.43 – 1.29 (m, 2H), 1.23 – 1.15 (m, 2H), 1.12 (s, 6H), 1.07 (s, 6H), 0.71 – 0.67 (m, 3H), 0.51 – 0.47 (m, 3H); **^{13}C NMR** (101 MHz, $CDCl_3$) δ 173.90,

139.46, 138.27, 129.15, 128.40, 128.34, 127.83, 127.63, 125.91, 83.65, 76.69, 73.04, 71.64, 49.21, 26.25, 26.10, 24.60, 24.55, 9.54, 9.17. **HRMS** (ESI) *m/z* calcd for $C_{28}H_{40}BO_5$ $(M+H)^+$ 467.2963, found 467.2959.

***syn*-Pentan-3-yl-2-(phenyl(4,4,5,5-tetramethyl-1,3,2-dioxaborolan-2-yl)methyl)-5-(trimethylsilyl)pent-4-ynoate (8u)**

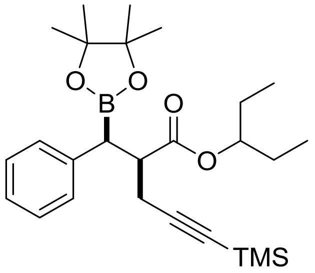

Following the General Procedure C using **1b** (0.25 mmol, 86.6 mg) and 3-bromo-1-(trimethylsilyl)-1-propyne (0.375 mmol, 61 μL). Analysis of the crude ^{1}H NMR indicated 6.7:1 diastereoselectivity. Purification by silica gel chromatography gave 81.0 mg (71% yield) of title compound as a colorless oil.

^{1}H NMR (400 MHz, $CDCl_3$) δ 7.27 – 7.10 (m, 5H), 4.56 – 4.50 (m, 1H), 3.16 – 3.10 (m, 1H), 2.68 – 2.51 (m, s3H), 1.45 – 1.38 (m, 2H), 1.20 – 1.17 (m, 14H), 0.79 – 0.52 (m, 6H), 0.12 (s, 9H); **^{13}C NMR** (101 MHz, $CDCl_3$) δ 173.92, 139.45, 129.31, 128.42, 126.12, 104.25, 86.20, 83.84, 76.91, 48.21, 26.31, 25.95, 24.68, 24.66, 23.30, 9.84, 9.26, 0.16. **HRMS** (ESI) *m/z* calcd for $C_{26}H_{41}BNaO_4Si$ $(M+Na)^+$ 479.2759, found 479.2758.

***syn*-Pentan-3-yl-3-phenyl-2-(phenylthio)-3-(4,4,5,5-tetramethyl-1,3,2-dioxaborolan-2-yl)propanoate (8v)**

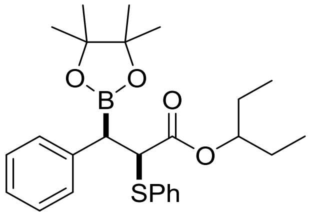

Following the General Procedure C using **1b** (0.25 mmol, 86.6 mg) and diphenyl disulfide (0.375 mmol, 81.9 mg). Analysis of the crude ^{1}H NMR indicated 20:1 diastereoselectivity. Purification by silica gel chromatography gave 72.7 mg (64% yield) of title compound as a white solid. **m.p.** = 90 – 91 ℃.

^{1}H NMR (400 MHz, $CDCl_3$) δ 7.58 (d, *J* = 7.2 Hz, 2H), 7.32 – 7.09 (m, 8H), 4.51 – 4.45 (m, 1H), 4.17 (d, *J* = 13.1 Hz, 2H), 2.94 (d, *J* = 13.1 Hz, 2H), 1.24 (s, 6H), 1.20 (s, 6H), 0.59 – 0.47 (m, 6H); **^{13}C NMR** (101 MHz, $CDCl_3$) δ 171.70, 138.77, 134.35, 132.82, 128.94, 128.47, 127.86, 126.32, 84.05, 77.34, 53.39, 26.22, 26.03, 24.81, 24.64, 9.39, 9.09. **HRMS** (ESI) *m/z* calcd for $C_{26}H_{36}BO_4S$ $(M+H)^+$ 455.2422, found 455.2429.

***anti*-*tert*-Butyl-2-methyl-3-phenyl-3-(4,4,5,5-tetramethyl-1,3,2-dioxaborolan-2-yl)propanoate (7q)**

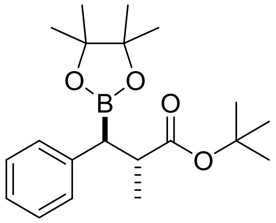

Following the General Procedure C using **1c** (0.25 mmol, 83.1 mg) and iodomethane (0.375 mmol, 23 μL). Analysis of the crude ^{1}H NMR indicated >20:1 diastereoselectivity. Purification by silica gel chromatography gave 72.7 mg (84% yield) of title compound as a colorless oil.

^{1}H NMR (400 MHz, $CDCl_3$) δ 7.26 – 7.12 (m, 5H), 2.86 – 2.78 (m, 1H), 2.34 (d, *J* = 11.5 Hz, 1H), 1.47 (s, 9H), 1.20 (s, 6H), 1.13 (s, 6H), 0.98 (d, *J* = 7.2 Hz, 3H); **^{13}C NMR** (101 MHz, $CDCl_3$) δ 176.77, 140.61, 129.17, 128.47, 125.67, 83.37, 80.09, 43.29, 28.23, 24.72, 24.57, 17.22; **HRMS** (ESI) *m/z* calcd for $C_{20}H_{32}BO_4$ $(M+H)^+$ 347.2388, found 347.2390.

***anti-tert*-Butyl-2-benzyl-3-phenyl-3-(4,4,5,5-tetramethyl-1,3,2-dioxaborolan-2-yl)propanoate (7r)**

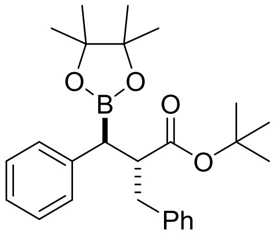

Following the General Procedure C using **1c** (0.25 mmol, 83.1 mg) and benzyl bromide (0.375 mmol, 45 μL). Analysis of the crude ^{1}H NMR indicated >20:1 diastereoselectivity. Purification by silica gel chromatography gave 69.7 mg (66% yield) of title compound as a white solid. **m.p.** = 127 – 128 ℃.

^{1}H NMR (400 MHz, $CDCl_3$) δ 7.31 – 7.01 (m, 10H), 3.19 – 3.13 (m, 1H), 2.86 (dd, *J* = 13.9, 4.0 Hz, 1H), 2.55 – 2.49 (m, 2H), 1.30 (s, 9H), 1.16 (s, 6H), 1.11 (s, 6H); **^{13}C NMR** (101 MHz, $CDCl_3$) δ 175.13, 140.27, 139.51, 129.47, 129.27, 128.64, 128.00, 126.08, 125.84, 83.48, 80.43, 49.82, 37.32, 28.08, 24.76, 24.47; **HRMS** (ESI) *m/z* calcd for $C_{26}H_{36}BO_4$ $(M+H)^+$ 423.2701, found 423.2707.

***anti-tert*-Butyl (*E*)-5-phenyl-2-(phenyl(4,4,5,5-tetramethyl-1,3,2-dioxaborolan-2-yl)methyl)pent-4-enoate (7s)**

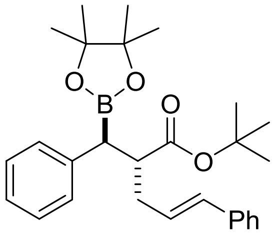

Following the General Procedure C using **1c** (0.25 mmol, 83.1 mg) and cinnamyl bromide (0.375 mmol, 55 μL). Analysis of the crude ^{1}H NMR indicated >20:1 diastereoselectivity . Purification by silica gel chromatography gave 80.7 mg (72% yield) of title compound as a white solid. **m.p.** = 105 – 106 ℃.

^{1}H NMR (400 MHz, $CDCl_3$) δ 7.29 – 7.12 (m, 10H), 6.25 (d, *J* = 15.7 Hz, 1H), 6.09 – 6.01 (m, 1H), 3.05 – 3.00 (m, 1H), 2.59 (d, *J* = 12.1 Hz, 1H), 2.48 – 2.42 (m, 1H), 2.20 – 2.12 (m, 1H), 1.45 (s, 9H), 1.19 (s, 6H), 1.12 (s, 6H); **^{13}C NMR** (101 MHz, $CDCl_3$) δ 175.15, 140.22, 137.67, 132.28, 129.25, 128.61, 128.53, 127.07, 126.85, 126.13, 125.80, 83.44, 80.56, 48.26, 33.97, 28.31, 24.70, 24.55; **HRMS** (ESI) *m/z* calcd for $C_{28}H_{38}BO_4$ $(M+H)^+$ 449.2858, found 449.2867.

***anti-tert*-Butyl-2-((benzyloxy)methyl)-3-phenyl-3-(4,4,5,5-tetramethyl-1,3,2-dioxaborolan-2-yl)propanoate (7t)**

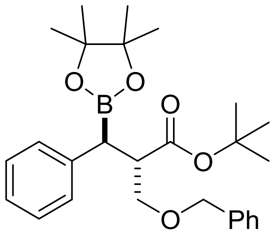

Following the General Procedure C using **1c** (0.25 mmol, 83.1 mg) and benzylchloromethyl ether (0.375 mmol, 52 μL). Analysis of the crude ^{1}H NMR indicated >20:1 diastereoselectivity. Purification by silica gel chromatography gave 84.8 mg (75% yield) of title compound as a white solid. **m.p.** = 51 – 52 ℃.

^{1}H NMR (400 MHz, $CDCl_3$) δ 7.30 – 7.11 (m, 10H), 4.61 – 4.02 (m, 2H), 3.59 (dd, *J* = 9.0, 3.1 Hz, 1H), 3.22 (dd, *J* = 9.0, 4.0 Hz, 1H), 2.98 – 2.93 (m, 1H), 2.83 (d, *J* = 12.0 Hz, 1H), 1.45 (s, 9H), 1.21 (s, 6H), 1.13 (s, 6H); **^{13}C NMR** (101 MHz, $CDCl_3$) δ 173.94, 140.29, 138.71, 129.34, 128.48, 128.25, 127.45, 127.35, 125.70, 83.37, 80.48, 72.90, 68.88, 49.73, 28.23, 24.68, 24.63. **HRMS** (ESI) *m/z* calcd for $C_{27}H_{38}BO_5$ $(M+H)^+$ 453.2807, found 453.2807.

***anti-tert*-Butyl-2-(phenyl(4,4,5,5-tetramethyl-1,3,2-dioxaborolan-2-yl)methyl)-5-(trimethylsilyl)pent-4-ynoate (7u)**

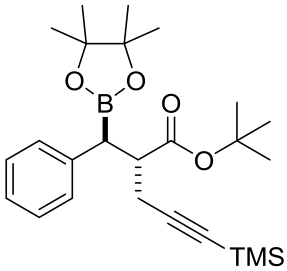

Following the General Procedure C using **1c** (0.25 mmol, 83.1 mg) and 3-bromo-1-(trimethylsilyl)-1-propyneb (0.375 mmol, 61 μL). Analysis of the crude ^{1}H NMR indicated >20:1 diastereoselectivity. Purification by silica gel chromatography gave 71.9 mg (65% yield) of title compound as a white solid. **m.p.** = 50 – 51 ℃.

^{1}H NMR (400 MHz, $CDCl_3$) δ 7.29 – 7.15 (m, 5H), 2.96 (ddd, *J* = 12.0, 5.5, 4.3 Hz, 1H), 2.72 (d, *J* = 12.1 Hz, 1H), 2.53 (dd, *J* = 18.5, 5.7 Hz, 1H), 2.09 (dd, *J* = 16.9, 5.7 Hz, 1H), 1.51 (s, 9H), 1.24 (s, 6H), 1.15 (s, 6H), 0.15 (s, 9H); **^{13}C NMR** (101 MHz, $CDCl_3$) δ 173.59, 139.91, 129.22, 128.63, 125.93, 103.47, 86.74, 83.43, 80.71, 47.36, 28.18, 24.72, 24.54, 21.38, 0.26; **HRMS** (ESI) *m/z* calcd for $C_{26}H_{36}BKO_4$ $(M+K)^+$ 481.2342, found 481.2340.

***anti-tert*-Butyl-3-phenyl-2-(phenylthio)-3-(4,4,5,5-tetramethyl-1,3,2-dioxaborolan-2-yl)propanoate (7v)**

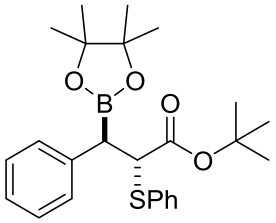

Following the General Procedure C using **1c** (0.25 mmol, 83.1 mg) and diphenyl disulfide (0.375 mmol, 81.9 mg). Analysis of the crude ^{1}H NMR indicated 16.5:1 diastereoselectivity. Purification by silica gel chromatography gave 89.2 mg (81% yield) of title compound as a white solid. **m.p.** = 113 – 115 ℃.

^{1}H NMR (400 MHz, $CDCl_3$) δ 7.31 – 7.20 (m, 10H), 3.97 (d, *J* = 12.6 Hz, 1H), 2.71 (d, *J* = 12.6 Hz, 1H), 1.38 (s, 9H), 1.17 (s, 6H), 1.10 (s, 6H); **^{13}C NMR** (101 MHz, $CDCl_3$) δ 172.29, 138.84, 134.22, 133.76, 129.20, 128.60, 128.57, 127.74, 126.25, 83.87, 81.23, 54.59, 28.00, 24.68, 24.40; **HRMS** (ESI) *m/z* calcd for $C_{25}H_{34}BO_4S$ $(M+H)^+$ 441.2265, found 441.2270.

***anti-N,N*-Diethyl-2-methyl-3-phenyl-3-(4,4,5,5-tetramethyl-1,3,2-dioxaborolan-2-yl)propanamide (7x)**

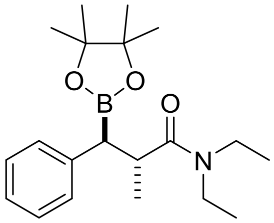

Following the General Procedure E using **5q** (0.25 mmol, 82.8 mg) and iodomethane (0.375 mmol, 23 μL). Analysis of the crude ^{1}H NMR indicated >20:1 diastereoselectivity. Purification by silica gel chromatography gave 76.0 mg (88% yield) of title compound as a colorless oil.

^{1}H NMR (400 MHz, $CDCl_3$) δ 7.26 – 7.10 (m, 5H), 3.57 – 3.25 (m, 4H), 2.97 (dq, *J* = 9.3, 7.0 Hz, 1H), 2.42 (d, *J* = 9.3 Hz, 1H), 1.30 – 1.09 (m, 18H), 1.06 (d, *J* = 7.0 Hz, 3H); **^{13}C NMR** (101 MHz, $CDCl_3$) δ 177.81, 142.49, 128.90, 128.19, 125.12, 82.29, 42.21, 41.33, 40.74, 24.71, 24.56, 18.08, 14.21, 13.00; **HRMS** (ESI) *m/z* calcd for $C_{20}H_{33}BNO_3$ $(M+H)^+$ 346.2548, found 346.2547.

***syn*-Pentan-3-yl 2-(hydroxy(phenyl)methyl)pent-4-enoate (4ba)[10]**

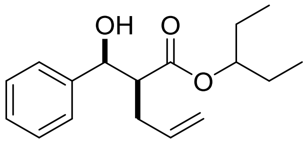

To a mixed solution of **4b** (0.25 mmol, 96.6 mg) in THF/H_2O (1.5 ml/1.5 ml) was added $NaBO_3 \cdot 4H_2O$ (1.5 mmol, 0.230 g) at room temperature. The reaction mixture was stirred for 8 h and quenched with saturated aq. $Na_2S_2O_3$ (5.0 mL). The mixture was extracted with DCM (3 × 10.0 mL). The combined organic phases were washed with water and brine, dried ($MgSO_4$) and concentrated in vacuo. Analysis of the crude ^{1}H NMR indicated >20:1 diastereoselectivity. Purification of the crude product by silica gel chromatography gave 60.8 mg (88% yield) of title compound as a colorless oil (ethyl acetate: petroleum ether = 1:10).

^{1}H NMR (400 MHz, $CDCl_3$) δ 7.38 – 7.24 (m, 5H), 5.80 – 5.70 (m, 1H), 5.06 (dd, *J* = 17.1, 1.6 Hz, 1H), 4.99 (d, *J* = 10.1 Hz, 1H), 4.95 (dd, *J* = 6.0, 2.9 Hz, 1H), 4.69 – 4.63 (m, 1H), 2.85 (d, *J* = 2.9 Hz, 1H), 2.84 – 2.80 (m, 1H), 2.54 – 2.40 (m, 2H), 1.51 – 1.34 (m, 4H), 0.81 (t, *J* = 7.5 Hz, 3H), 0.67 (t, *J* = 7.5 Hz, 3H); **^{13}C NMR** (101 MHz, $CDCl_3$) δ 174.28, 141.52, 135.55, 128.49, 127.96, 126.53, 117.02, 77.42, 74.26, 53.02, 31.94, 26.21, 26.14, 9.69, 9.38; **HRMS** (ESI) *m/z* calcd for $C_{17}H_{25}O_3$ $(M+H)^+$ 277.1798, found 277.1799.

***anti-tert*-Butyl-2-(hydroxy(phenyl)methyl)pent-4-enoate (3ca)**

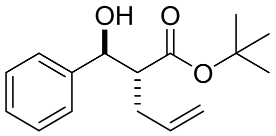

To a mixed solution of **3c** (0.25 mmol, 93.1 mg) in THF/H_2O (1.5 ml/1.5 ml) was added $NaBO_3 \cdot 4H_2O$ (1.5 mmol, 0.230 g) at room temperature. The reaction mixture was stirred for 8 h and quenched with saturated aq. $Na_2S_2O_3$ (5.0 mL). The mixture was extracted with DCM (3 × 10.0 mL). The combined organic phases were washed with water and brine, dried ($MgSO_4$) and concentrated in vacuo. Analysis of the crude ^{1}H NMR indicated >20:1 diastereoselectivity. Purification of the crude product by silica gel chromatography gave 57.1 mg (87% yield) of title compound as a colorless oil (ethyl acetate: petroleum ether =1:10).

^{1}H NMR (400 MHz, $CDCl_3$) δ 7.37 – 7.26 (m, 5H), 5.78 – 5.68 (m, 1H), 5.07 – 5.01 (m, 2H), 4.80 – 4.77 (m, 1H), 3.23 (d, J = 6.4 Hz, 1H), 2.76 – 2.71 (m, 1H), 2.35 – 2.18 (m, 1H), 1.38 (s, 9H); **^{13}C NMR** (101 MHz, $CDCl_3$) δ 174.16, 142.12, 134.71, 128.51, 127.91, 126.48, 117.34, 81.63, 74.72, 52.99, 34.18, 28.15; **HRMS** (ESI) *m/z* calcd for $C_{16}H_{23}O_3$ $(M+H)^+$ 263.1642, found 263.1645.

***syn*-2-allyl-1-phenylpropane-1,3-diol (12)**

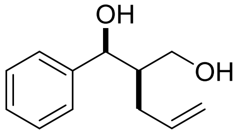

To a solution of **4ba** (0.25 mmol, 69.1 mg) in anhydrous THF (3.0 ml) was added $LiAlH_4$ (1.25 mmol, 47.4 mg) at 0 °C. The reaction mixture was stirred for 6 h and quenched with saturated aq. NH_4Cl solution (1 mL) and diluted with DCM (5.0 mL). The layers were separated, the aqueous phase was extracted with DCM (2 × 5 mL), the combined organic phases were washed with water and brine, dried ($MgSO_4$) and concentrated in vacuo. Analysis of the crude ^{1}H NMR indicated >20:1 diastereoselectivity. Purification of the crude product by silica gel chromatography gave 41.3 mg (86% yield) of title compound as a colorless oil (ethyl acetate: petroleum ether = 1:2).

^{1}H NMR (400 MHz, $CDCl_3$) δ 7.38 – 7.25 (m, 5H), 5.79 – 5.68 (m, 1H), 5.05 – 5.00 (m, 3H), 3.73 – 3.65 (m, 2H), 3.13 (s, 1H), 2.41 (s, 1H), 2.10 – 1.97 (m, 3H); **^{13}C NMR** (101 MHz, $CDCl_3$) δ 142.45, 137.04, 128.40, 127.54, 126.35, 116.70, 76.55, 63.89, 46.33, 30.11; **HRMS** (ESI) *m/z* calcd for $C_{12}H_{16}LiO_2$ $(M+Li)^+$ 199.1305, found 199.1313.

***anti*-2-allyl-1-phenylpropane-1,3-diol (11)**

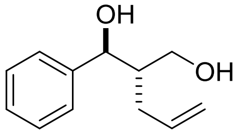

To a solution of **3ca** (0.25 mmol, 65.6 mg) in anhydrous THF (3.0 ml) was added $LiAlH_4$ (1.25 mmol, 47.4 mg) at 0 °C. The reaction mixture was stirred for 6 h and quenched with saturated aq. NH_4Cl solution (1 mL) and diluted

with DCM (5.0 mL). The layers were separated, the aqueous phase was extracted with DCM (2 × 5.0 mL), the combined organic phases were washed with water and brine, dried ($MgSO_4$) and concentrated in vacuo. Analysis of the crude ^{1}H NMR indicated >20:1 diastereoselectivity. Purification of the crude product by silica gel chromatography gave 41.2 mg (86% yield) of title compound as a colorless oil (ethyl acetate: petroleum ether = 1:2).

^{1}H NMR (400 MHz, $CDCl_3$) δ 7.38 – 7.26 (m, 5H), 5.77 – 5.67 (m, 1H), 5.05 – 5.00 (m, 2H), 4.72 (d, *J* = 6.7 Hz, 1H), 3.82 (d, *J* = 10.9 Hz, 1H), 3.67 (dd, *J* = 11.0, 6.0 Hz, 1H), 3.18 (s, 1H), 2.88 (s, 1H), 2.11 – 1.88 (m, 3H); **^{13}C NMR** (101 MHz, $CDCl_3$) δ 143.40, 136.29, 128.62, 127.88, 126.58, 116.91, 78.49, 64.27, 46.33, 33.13; **HRMS** (ESI) *m/z* calcd for $C_{12}H_{17}O_2$ (M+H)$^+$ 193.1223, found 193.1223.

syn-Pentan-3-yl-2-(2-hydroxy-1-phenylethyl)pent-4-enoate (13)[11]

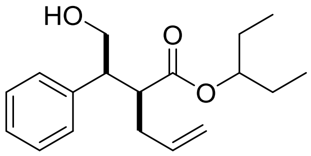

A solution of **4b** (0.25 mmol, 96.6 mg) and bromochloromethane (0.275 mmol, 32.5 μL) in Et_2O (5 mL) was cooled to -78 ℃ and treated with *n*-BuLi (110 μL, 2.5 M in hexane) dropwise over one minute. The reaction mixture was allowed to slowly warm to room temperature and then stirred for 2 h. Then the reaction was quenched with saturated aq. NH_4Cl solution (1.0 mL) and diluted with Et_2O (5.0 mL). The layers were separated, the aqueous phase was extracted with Et_2O (2 × 5.0 mL), the combined organic phases were washed with water and brine, dried ($MgSO_4$) and concentrated in vacuo. The residue was dissolved in THF/H_2O (1.5 ml/1.5 ml) and $NaBO_3$•$4H_2O$ (1.5 mmol, 0.230 g) was added at room temperature. The reaction mixture was stirred for 8 h and quenched with saturated aq. $Na_2S_2O_3$ (5.0 mL). The mixture was extracted with DCM (3 × 10.0 mL). The combined organic phases were washed with water and brine, dried ($MgSO_4$) and concentrated in vacuo. Analysis of the crude ^{1}H NMR indicated >20:1 diastereoselectivity. Purification of the crude product by silica gel chromatography gave 50.1 mg (69% yield) of title compound as a colorless oil (ethyl acetate: petroleum ether = 1:10).

^{1}H NMR (400 MHz, $CDCl_3$) δ 7.31 – 7.20 (m, 5H), 5.84 – 5.73 (m, 1H), 5.11 (d, *J* = 17.1 Hz, 1H), 5.04 (d, *J* = 10.2 Hz, 1H), 4.55 – 4.48 (m, 1H), 3.95 – 3.83 (m, 2H), 3.12 – 3.07 (m, 1H), 2.98 – 2.92 (m, 1H), 2.51 – 2.37 (m, 2H), 1.54 (t, *J* = 6.0 Hz, 1H), 1.43 – 1.17 (m, 4H), 0.75 – 0.71 (m, 3H), 0.61 – 0.57 (m, 3H); **^{13}C NMR** (101 MHz, $CDCl_3$) δ 173.92, 140.19, 135.19, 128.76, 128.72, 127.34, 117.35, 76.99, 64.44, 49.74, 47.83, 34.76, 25.89, 25.87, 9.58, 9.29; **HRMS** (ESI) *m/z* calcd for $C_{18}H_{27}O_3$ (M+H)$^+$ 291.1955, found 291.1955.

syn-Pentan-3-yl-2-((1-methyl-1H-indol-2-yl)(phenyl)methyl)pent-4-enoate (16)[12]

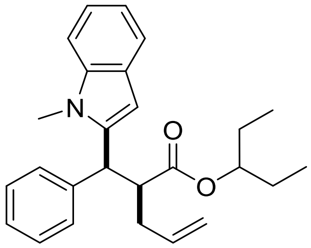

To a solution of *N*-methylindole (0.40 mmol, 50 µL) in THF (1.0 mL) was added *n*-BuLi (160 µL, 2.5 M in hexanes) dropwise at room temperature. The mixture was heated under reflux for 3 h. The reaction mixture was cooled to -78 ℃ and a solution of **4b** (0.25 mmol, 96.6 mg) in THF (1.0 mL) was added dropwise. The mixture was stirred at -78 ℃ for 1 h and a solution of NIS (0.30 mmol, 68 mg) in THF (1.0 mL) was added dropwise. After 1 h, saturated aq. $Na_2S_2O_3$ (2.0 mL) was added. And the reaction mixture was allowed to warm to room temperature. The reaction mixture was diluted with DCM (5.0 mL) and washed with water (10.0 mL). The aqueous layer was extracted with DCM (2 x 10.0 mL). The combined organic layers were dried ($MgSO_4$), filtered and concentrated under vacuum. Analysis of the crude ^{1}H NMR indicated >20:1 diastereoselectivity. Purification of the crude product by silica gel chromatography gave 82.8 mg (85% yield) of title compound as a colorless oil (ethyl acetate: petroleum ether = 1:40).

^{1}H NMR (400 MHz, $CDCl_3$) δ 7.61 (d, *J* = 7.7 Hz, 1H), 7.28 – 7.06 (m, 8H), 6.54 (s, 1H), 5.86 – 5.75 (m, 1H), 5.05 – 4.99 (m, 2H), 4.52 – 4.46 (m, 1H), 4.32 (d, *J* = 11.3 Hz, 1H), 3.54 (s, 3H), 3.36 – 3.30 (m, 1H), 2.70 – 2.42 (m, 2H), 1.45 – 1.38 (m, 3H), 1.10 – 1.02 (m, 2H), 0.81 – 0.77 (m, 3H), 0.40 – 0.37 (m, 3H); **^{13}C NMR** (101 MHz, $CDCl_3$) δ 174.00, 140.90, 140.15, 137.35, 135.03, 129.05, 128.55, 127.79, 127.08, 121.14, 120.26, 119.56, 117.17, 109.09, 98.88, 77.07, 51.88, 46.24, 36.62, 29.78, 25.86, 25.67, 9.71, 8.90; **HRMS** (ESI) *m/z* calcd for $C_{26}H_{32}NO_2$ $(M+H)^+$ 390.2428, found 390.2421.

***syn*-Pentan-3-yl-2-(furan-2-yl(phenyl)methyl)pent-4-enoate (15)**[12]

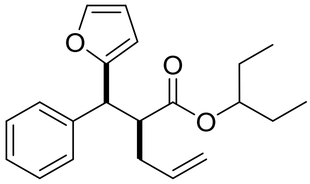

To a solution of furan (0.30 mmol, 21.8 µL) in THF (1.0 mL) was added *n*-BuLi (120 µL, 2.5 M in hexane) dropwise at room temperature. The cooling bath was removed and the mixture was stirred at room temperature for 1 h. The reaction mixture was cooled to -78 ℃ and a solution of **4b** (0.25 mmol, 96.6 mg) in THF (1.0 mL) was added dropwise. The mixture was stirred at -78 ℃ for 1 h and a solution of NBS (0.30 mmol, 53.4 mg) in THF (1.0 mL) was added dropwise. After 1 h, saturated aq. $Na_2S_2O_3$ (2.0 mL) was added. And the reaction mixture was allowed to warm to room temperature. The reaction mixture was diluted with DCM (5.0 mL) and washed with water (10.0 mL). The aqueous layer was extracted with DCM (2 x 10.0 mL). The combined organic layers were dried ($MgSO_4$), filtered and concentrated under vacuum. Analysis of the crude ^{1}H NMR indicated >20:1 diastereoselectivity. Purification of the crude product by silica gel chromatography gave 73.4 mg (90% yield) of title compound as a colorless oil (ethyl acetate: petroleum ether = 1:40).

^{1}H NMR (400 MHz, $CDCl_3$) δ 7.36 – 7.13 (m, 6H), 6.27 (dd, *J* = 3.0, 1.9 Hz, 1H), 6.15 (d, *J* = 3.0 Hz, 1H), 5.79 – 5.68 (m, 1H), 5.04 – 4.97 (m, 2H), 4.52 – 4.46 (m, 1H), 4.18 (d, *J* = 11.5 Hz, 1H), 3.33 – 3.27 (m, 1H), 2.35 – 2.18 (m, 2H), 1.40 – 1.33 (m, 2H), 1.20 – 1.09 (m, 2H), 0.75 – 0.72 (m, 3H), 0.49 – 0.45 (m, 3H); **^{13}C NMR** (101 MHz, $CDCl_3$) δ 173.71, 154.90, 141.86, 140.38, 134.99, 128.49, 128.45, 127.04, 117.16, 110.27, 106.95, 77.00, 50.65, 47.82, 36.19, 25.96, 25.83, 9.65, 9.07. **HRMS** (ESI) *m/z* calcd for $C_{21}H_{26}LiO_3$ $(M+Li)^+$ 333.2037, found 333.2026.

***syn*-Pentan-3-yl-2-(phenyl(thiophen-2-yl)methyl)pent-4-enoate (14)**[12]

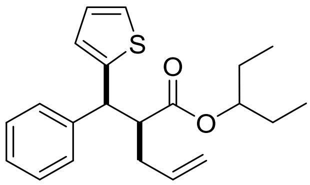

To a solution of thiophene (0.30 mmol, 24.0 μL) in THF (1.0 mL) was added *n*-BuLi (120 μL, 2.5 M in hexane) dropwise at room temperature. The mixture was warmed to 0 ℃ and stirred for 30 min. The reaction mixture was cooled to -78 ℃ and a solution of **4b** (0.25 mmol, 96.6 mg) in THF (1.0 mL) was added dropwise. The mixture was stirred at -78 ℃ for 1 h and a solution of NBS (0.30 mmol, 53.4 mg) in THF (1.0 mL) was added dropwise. After 1 h, saturated aq. $Na_2S_2O_3$ (2.0 mL) was added. And the reaction mixture was allowed to warm to room temperature. The reaction mixture was diluted with DCM (5 mL) and washed with water (10.0 mL). The aqueous layer was extracted with DCM (2 x 10.0 mL). The combined organic layers were dried ($MgSO_4$), filtered and concentrated under vacuum. Analysis of the crude ^{1}H NMR indicated >20:1 diastereoselectivity. Purification of the crude product by silica gel chromatography gave 75.4 mg (88% yield) of title compound as a colorless oil (ethyl acetate: petroleum ether = 1:40).

^{1}H NMR (400 MHz, $CDCl_3$) δ 7.36 – 7.12 (m, 6H), 6.95 (d, *J* = 3.3 Hz, 1H), 6.90 (dd, *J* = 4.9, 3.6 Hz, 1H), 5.80 – 5.70 (m, 1H), 5.05 – 4.98 (m, 2H), 4.52 – 4.46 (m, 1H), 4.39 (d, *J* = 11.6 Hz, 1H), 3.28 – 3.21 (m, 1H), 2.37 – 2.30 (m, 2H), 1.41 – 1.29 (m, 2H), 1.24 – 1.10 (m, 2H), 0.74 – 0.70 (m, 3H), 0.54 – 0.50 (m, 3H); **^{13}C NMR** (101 MHz, $CDCl_3$) δ 173.69, 146.19, 142.43, 134.93, 128.62, 127.99, 126.99, 126.79, 124.91, 124.38, 117.28, 77.07, 52.58, 49.68, 36.39, 25.92, 25.85, 9.63, 9.20; **HRMS** (ESI) *m/z* calcd for $C_{21}H_{27}O_2S$ $(M+H)^+$ 343.1726, found 343.1726.

***anti*-*tert*-Butyl-2-methyl-2-(phenyl(4,4,5,5-tetramethyl-1,3,2-dioxaborolan-2-yl)methyl)pent-4-enoate (9)**

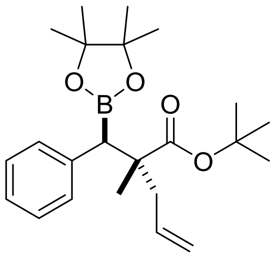

A solution of diisopropylamine (0.275 mmol, 38.5 μL) in THF (0.5 mL) was cooled to 0 ℃ and treated with *n*-BuLi (110 μL, 2.5 M in hexane) dropwise. The reaction mixture was stirred for 15 minutes and cooled to -78 ℃. To the freshly prepared solution of LDA was added a solution of **7q** (0.25 mmol, 86.6 mg) in THF/HMPA (0.5 ml/0.2 ml) dropwise over 2 minutes and then the mixture was warmed to 0 ℃. After stirring at the same temperature for 6 h, the allyl bromide (0.375 mmol, 32.5 μL) was added to the reaction mixture dropwise over one minute and stirred for 8 h. Then the reaction was quenched with saturated aq. NH_4Cl solution (1.0 mL) and diluted with DCM (5.0 mL). The layers were separated, the aqueous phase was extracted with DCM (2 × 5.0 mL), the combined organic phases were washed with water and brine, dried ($MgSO_4$) and concentrated in vacuo. Analysis of the crude

^{1}H NMR indicated >20:1 diastereoselectivity. Purification by silica gel chromatography gave 48.3 mg (50% yield) of title compound as a colorless oil.

^{1}H NMR (400 MHz, $CDCl_3$) δ 7.26 – 7.15 (m, 5H), 5.82 – 5.71 (m, 1H), 5.07 – 5.02 (m, 2H), 2.76 (s, 1H), 2.09 – 2.08 (m, 2H), 1.45 (s, 9H), 1.25 (s, 6H), 1.21 (s, 3H), 1.16 (s, 6H); ^{13}C NMR (101 MHz, $CDCl_3$) δ 177.09, 138.87, 134.99, 131.90, 127.83, 125.93, 117.95, 83.02, 80.47, 48.92, 42.53, 28.25, 24.90, 24.77, 21.41; **HRMS** (ESI) *m/z* calcd for $C_{23}H_{36}BO_4$ $(M+H)^+$ 387.2701, found 387.2707.

***anti-tert*-Butyl-2-methyl-2-(phenyl(4,4,5,5-tetramethyl-1,3,2-dioxaborolan-2-yl)methyl)pent-4-enoate (10)**

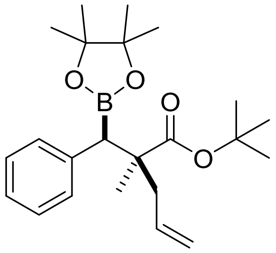

A solution of diisopropylamine (0.275 mmol, 38.5 μL) in THF (0.5 mL) was cooled to 0 ℃ and treated with *n*-BuLi (110 μL, 2.5 M in hexane) dropwise. The reaction mixture was stirred for 15 minutes and cooled to -78 ℃. To the freshly prepared solution of LDA was added a solution of **3c** (0.25 mmol, 93.1 mg) in THF/HMPA (0.5 ml/0.2 ml) dropwise over 2 minutes and then the mixture was warmed to 0 ℃. After stirring at the same temperature for 6 h, the iodomethane (0.375 mmol, 23 μL) was added to the reaction mixture dropwise over one minute and stirred for 8 h. Then the reaction was quenched with saturated aq. NH_4Cl solution (1.0 mL) and diluted with DCM (5.0 mL). The layers were separated, the aqueous phase was extracted with DCM (2 × 5.0 mL), the combined organic phases were washed with water and brine, dried ($MgSO_4$) and concentrated in vacuo. Analysis of the crude ^{1}H NMR indicated 7.4:1 diastereoselectivity. Purification by silica gel chromatography gave 50.2 mg (52% yield) of title compound as a colorless oil.

^{1}H NMR (400 MHz, $CDCl_3$) δ 7.29 – 7.13 (m, 5H), 5.81 – 5.71 (m, 1H), 5.06 – 5.02 (m, 2H), 2.87 (s, 1H), 2.62 (dd, J = 13.3, 7.0 Hz, 1H), 2.22 (dd, J = 13.5, 7.5 Hz, 1H), 1.35 (s, 9H), 1.25 (s, 6H), 1.21 (s, 6H), 1.19 (s, 3H); **^{13}C NMR** (101 MHz, $CDCl_3$) δ 175.41, 138.67, 134.79, 131.36, 127.84, 126.16, 117.74, 83.45, 80.37, 49.40, 44.46, 28.24, 24.88, 24.84, 19.11; **HRMS** (ESI) *m/z* calcd for $C_{23}H_{35}BKO_4$ $(M+K)^+$ 425.2260, found 425.2265.

Pentan-3-yl (2*R*,3*S*,*E*)-2-allyl-3-hydroxyhex-4-enoate (17)

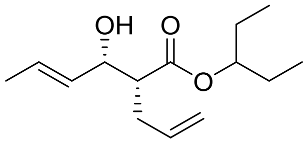

To a mixed solution of chiral **8o** (1.0 mmol, 350.3 mg) in THF/H_2O (6.0 ml/6.0 ml) was added $NaBO_3 \cdot 4H_2O$ (6.0 mmol, 0.920 g) at room temperature. The reaction mixture was stirred for 8 h and quenched with saturated aq. $Na_2S_2O_3$ (10.0 mL). The mixture was extracted with DCM (3 × 10.0 mL). The combined organic phases were washed with water and brine, dried ($MgSO_4$) and concentrated in vacuo. Purification by silica gel chromatography gave 225.9 mg (94% yield) of the title compound as a colorless oil.

^{1}H NMR (400 MHz, $CDCl_3$) δ 5.84 – 5.70 (m, 2H), 5.51 (ddq, *J* = 15.3, 7.0, 1.6 Hz, 1H), 5.11 – 5.00 (m, 2H), 4.80 – 4.74 (m, 1H), 4.28 – 4.23 (m, 1H), 2.63 – 2.58 (m, 1H), 2.49 – 2.35 (m, 3H), 1.71 – 1.68 (m, 3H), 1.60 – 1.52 (m, 4H), 0.90 – 0.86 (m, 6H); **^{13}C NMR** (101 MHz, $CDCl_3$) δ 173.95, 135.73, 130.55, 129.01, 116.90, 77.38, 73.20, 51.56, 32.24, 26.41, 26.39, 17.88, 9.74, 9.70. **HRMS** (ESI) *m/z* calcd for $C_{14}H_{24}LiO_3$ $(M+Li)^+$ 247.1873, found 247.1872. HPLC analysis of the product after being esterified with benzoyl chloride (IA, Hexanes: *i*-PrOH = 99:1, 1 mL/min, 25 ℃, λ = 225 nm) indicated 98.3% ee: t_R = 15.281 min (major), t_R = 17.209 min (minor). $[\alpha]_D^{25}$ = -2.5 (c = 1, CH_2Cl_2).

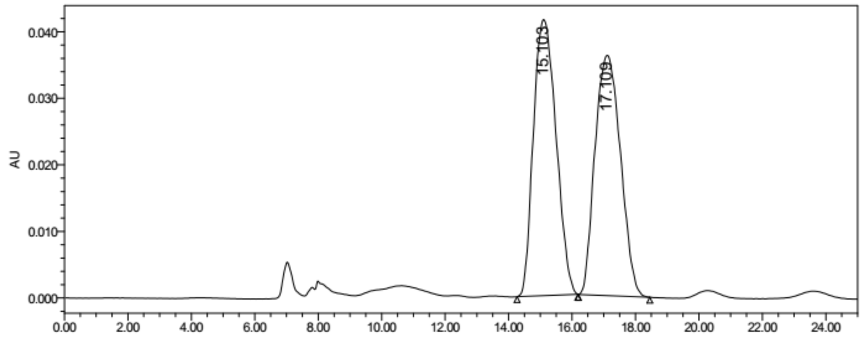

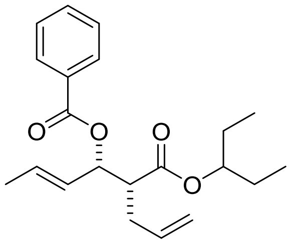

race

	Channel Description	RT (min)	Area	% Area	Height
1	W2489 ChA 254nm	15.103	2058316	50.49	41457
2	W2489 ChA 254nm	17.109	2018002	49.51	36086

Supplementary Figure 5. HPLC analysis of the *race*-**17** after esterification

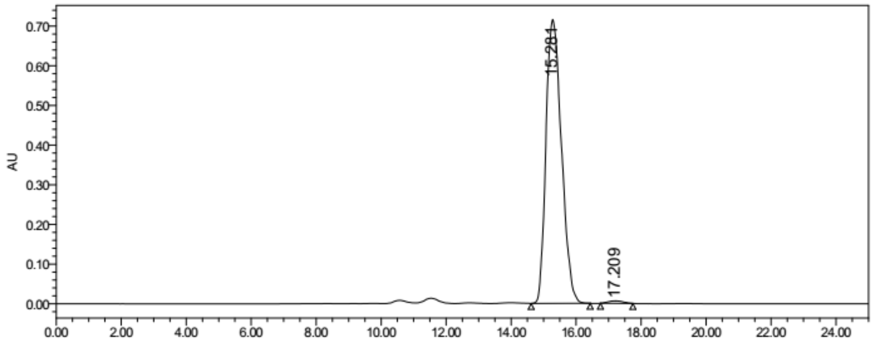

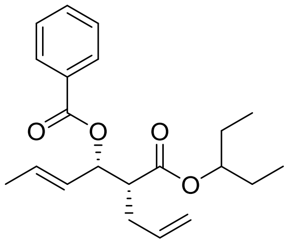

	Channel Description	RT (min)	Area	% Area	Height
1	W2489 ChA 254nm	15.281	23691658	99.17	714933
2	W2489 ChA 254nm	17.209	198229	0.83	6089

Supplementary Figure 6. HPLC analysis of the chiral-**17** after esterification

Pentan-3-yl (2*S*,3*S*,*E*)-2-allyl-3-hydroxyhex-4-enoate (20)

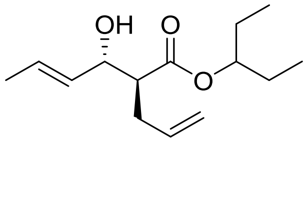

To a mixed solution of chiral **7o** (1.0 mmol, 350.3 mg) in THF/H_2O (6.0 ml/6.0 ml) was added $NaBO_3 \cdot 4H_2O$ (6.0 mmol, 0.920 g) at room temperature. The reaction mixture was stirred for 8 h and quenched with saturated aq. $Na_2S_2O_3$ (10.0 mL). The mixture was extracted with DCM (3 × 10.0 mL). The combined organic phases were washed with water and brine, dried ($MgSO_4$) and concentrated in vacuo. Purification by silica gel chromatography gave 221.1 mg (92% yield) of the title compound as a colorless oil.

^{1}H NMR (400 MHz, $CDCl_3$) δ 5.81 – 5.69 (m, 2H), 5.47 (ddq, *J* = 11.2, 6.6, 1.4 Hz, 1H), 5.14 – 5.06 (m, 1H), 5.05 – 5.02 (m, 1H), 4.82 – 4.76 (m, 1H), 4.21 – 4.16 (m, 1H), 2.67 (d, *J* = 6.9 Hz, 1H), 2.56 – 2.51 (m, 1H), 2.45 – 2.33 (m, 2H), 1.71 – 1.69 (m, 3H), 1.61 – 1.53 (m, 4H), 0.91 – 0.87 (m, 6H); **^{13}C NMR** (101 MHz, $CDCl_3$) δ 174.67, 134.98, 131.67, 128.52, 117.32, 77.48, 73.13, 51.33, 33.84, 26.51, 17.85, 9.78, 9.71. **HRMS** (ESI) *m/z* calcd for $C_{14}H_{24}NaO_3$ $(M+Na)^+$ 263.1618, found 263.1611. HPLC analysis of the product after being esterified with benzoyl chloride (IA, Hexanes: *i*-PrOH = 90:10, 1 mL/min, 25 ℃, λ = 225 nm) indicated 98.6% ee: t_R = 9.179 min (major), t_R = 11.627 min (minor). $[\alpha]_D^{25}$ = -11.4 (c = 1, CH_2Cl_2).

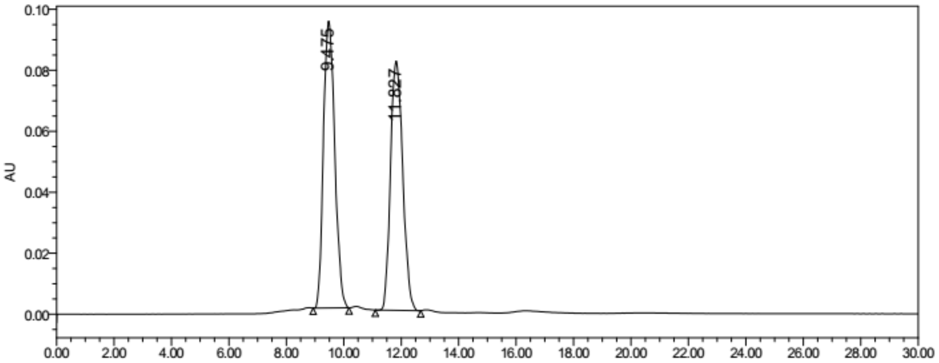

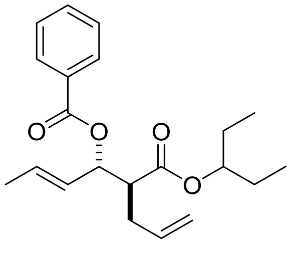

race

	Channel Description	RT (min)	Area	% Area	Height
1	W2489 ChA 254nm	9.475	2566695	51.50	94093
2	W2489 ChA 254nm	11.827	2417626	48.50	81753

Supplementary Figure 7. HPLC analysis of the *race*-**20** after esterification

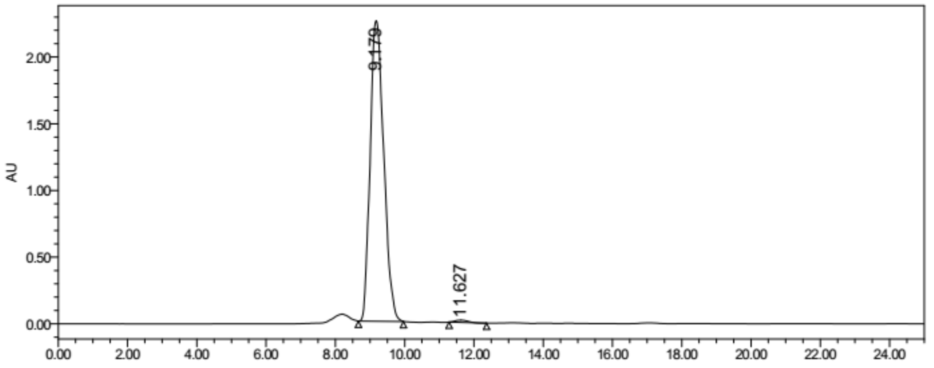

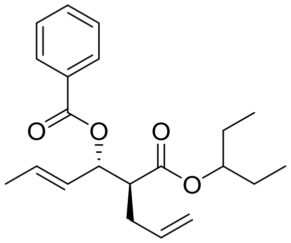

	Channel Description	RT (min)	Area	% Area	Height
1	W2489 ChA 254nm	9.179	61470574	99.28	2252973
2	W2489 ChA 254nm	11.627	446488	0.72	17205

Supplementary Figure 8. HPLC analysis of the chiral-**20** after esterification

Pentan-3-yl (1*R*,2*S*)-2-hydroxycyclopent-3-ene-1-carboxylate (18)

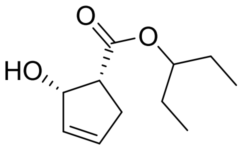

To a solution of **17** (0.5 mmol, 120.2 mg) in anhydrous DCM (5 ml) was added benzylidene-bis (tricyclohexylphosphine)dichlororuthenium (0.025 mmol, 21 mg) at room temperature. and The reaction mixture was stirred for 2 h. After that, the layers was separated and concentrated in vacuo. Purification of the crude product by silica gel chromatography gave 82.3 mg (83% yield) of title compound as a colorless oil (ethyl acetate: petroleum ether = 1:15).

^{1}H NMR (400 MHz, $CDCl_3$) δ 6.05 – 6.02 (m, 1H), 5.88 – 5.85 (m, 1H), 4.97 – 4.92 (m, 1H), 4.86 – 4.80 (m, 1H), 3.21 – 3.15 (m, 1H), 2.90 – 2.82 (m, 1H), 2.57 – 2.50 (m, 1H), 2.45 (d, *J* = 7.1 Hz, 1H), 1.68 – 1.52 (m, 4H), 0.94 – 0.88 (m, 6H); **^{13}C NMR** (101 MHz, CD_2Cl_2) δ 134.56, 131.76, 77.10, 76.53, 47.79, 33.50, 26.40, 26.38, 9.36, 9.33. **HRMS** (ESI) *m/z* calcd for $C_{11}H_{18}NaO_3$ $(M+Na)^+$ 221.1148, found 221.1141. $[\alpha]_D^{25}$ = 52 (c = 0.8, CH_2Cl_2).

Pentan-3-yl (1*S*,2*S*)-2-hydroxycyclopent-3-ene-1-carboxylate (21)

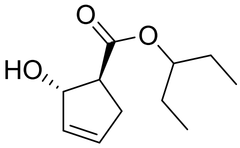

To a solution of **20** (0.5 mmol, 120.2 mg) in anhydrous DCM (5 ml) was added benzylidene-bis (tricycleohexylphosphine)dichlororuthenium (0.025 mmol, 21 mg) at room temperature. and The reaction mixture was stirred for 2 h. After that, the layers was separated and concentrated in vacuo. Purification of the crude product by silica gel chromatography gave 85.3 mg (86% yield) of title compound as a colorless oil (ethyl acetate: petroleum ether = 1:15).

^{1}H NMR (400 MHz, $CDCl_3$) δ 5.91 – 5.88 (m, 1H), 5.77 – 5.74 (m, 1H), 5.19 – 5.04 (m, 1H), 4.85 – 4.79 (m, 1H), 2.97 – 2.91 (m, 1H), 2.82 – 2.74 (m, 1H), 2.63 – 2.56 (m, 1H), 2.23 (d, *J* = 6.1 Hz, 1H), 1.66 – 1.52 (m, 4H), 0.92 – 0.87 (m, 6H); **^{13}C NMR** (101 MHz, $CDCl_3$) δ 174.42, 132.85, 132.34, 80.84, 77.26, 53.00, 34.86, 26.61, 26.57, 9.78, 9.76. **HRMS** (ESI) *m/z* calcd for $C_{11}H_{18}NaO_3$ $(M+Na)^+$ 221.1148, found 221.1143. $[\alpha]_D^{25}$ = 76.5 (c = 2, CH_2Cl_2).

(1*S*,5*S*)-5-(hydroxymethyl)cyclopent-2-en-1-ol (19)

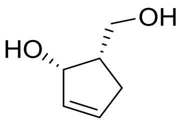

To a solution of **18** (0.15 mmol, 30.0 mg) in anhydrous THF (1.8 ml) was added $LiAlH_4$ (0.75 mmol, 28.2 mg) at 0 ℃. The reaction mixture was stirred for 6 h and quenched with saturated aq. NH_4Cl solution (1.0 mL) and diluted with DCM (3.0 mL). The layers were separated, the aqueous phase was extracted with DCM (2 × 3.0 mL), the combined organic phases were washed with water and brine, dried ($MgSO_4$) and concentrated in vacuo. Purification

of the crude product by silica gel chromatography gave 14.5 mg (85% yield) of title compound as a yellow oil (methanol: dichloromethane = 1:20).

^{1}H NMR (400 MHz, $CDCl_3$) δ 6.03 – 6.02 (m, 1H), 5.85 – 5.84 (m, 1H), 4.95 – 4.93 (m, 1H), 3.88 – 3.77 (m, 2H), 2.55 – 2.22 (m, 4H), 1.96 – 1.93 (m, 1H); **^{13}C NMR** (101 MHz, $CDCl_3$) δ 135.63, 132.58, 78.25, 63.08, 42.65, 33.77. **HRMS** (ESI) *m/z* calcd for $C_{26}H_{36}BO_4$ $(M+H)^+$ 423.2701, found 423.2707; **HRMS** (ESI) *m/z* calcd for $C_6H_{10}NaO_2$ $(M+Na)^+$ 137.0573, found 137.0580. $[\alpha]_D^{25}$ = 64.4 (c = 0.18, CH_2Cl_2).

(1*S*,5*R*)-5-(hydroxymethyl)cyclopent-2-en-1-ol (22)

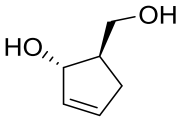

To a solution of **21** (0.15 mmol, 30.0 mg) in anhydrous THF (1.8 ml) was added $LiAlH_4$ (0.75 mmol, 28.2 mg) at 0 ℃. The reaction mixture was stirred for 6 h and quenched with saturated aq. NH_4Cl solution (1.0 mL) and diluted with DCM (3.0 mL). The layers were separated, the aqueous phase was extracted with DCM (2 × 3.0 mL), the combined organic phases were washed with water and brine, dried ($MgSO_4$) and concentrated in vacuo. Purification of the crude product by silica gel chromatography gave 15.2 mg (89% yield) of title compound as a yellow oil (methanol: dichloromethane = 1:20). **^{1}H NMR** (400 MHz, $CDCl_3$) δ 5.91 – 5.88 (m, 1H), 5.78 – 5.75 (m, 1H), 4.75 – 4.70 (m, 1H), 3.75 (dd, *J* = 10.5, 6.1 Hz, 1H), 3.62 (dd, *J* = 10.3, 8.6 Hz, 1H), 2.67 – 2.56 (m, 3H), 2.31 – 2.23 (m, 1H), 2.01 – 1.94 (m, 1H); **^{13}C NMR** (101 MHz, $CDCl_3$) δ 133.64, 132.92, 80.89, 65.39, 50.25, 34.35; **HRMS** (ESI) *m/z* calcd for $C_6H_{10}NaO_2$ $(M+Na)^+$ 137.0573, found 137.0578. $[\alpha]_D^{25}$ = 68.1 (c = 0.8, CH_2Cl_2).

Supplementary Figure 9. ^{1}H NMR spectrum for *tert*-Butyl-3-phenyl-3-(4,4,5,5-tetramethyl-1,3,2-dioxaborolan-2-yl)propanoate (1c)

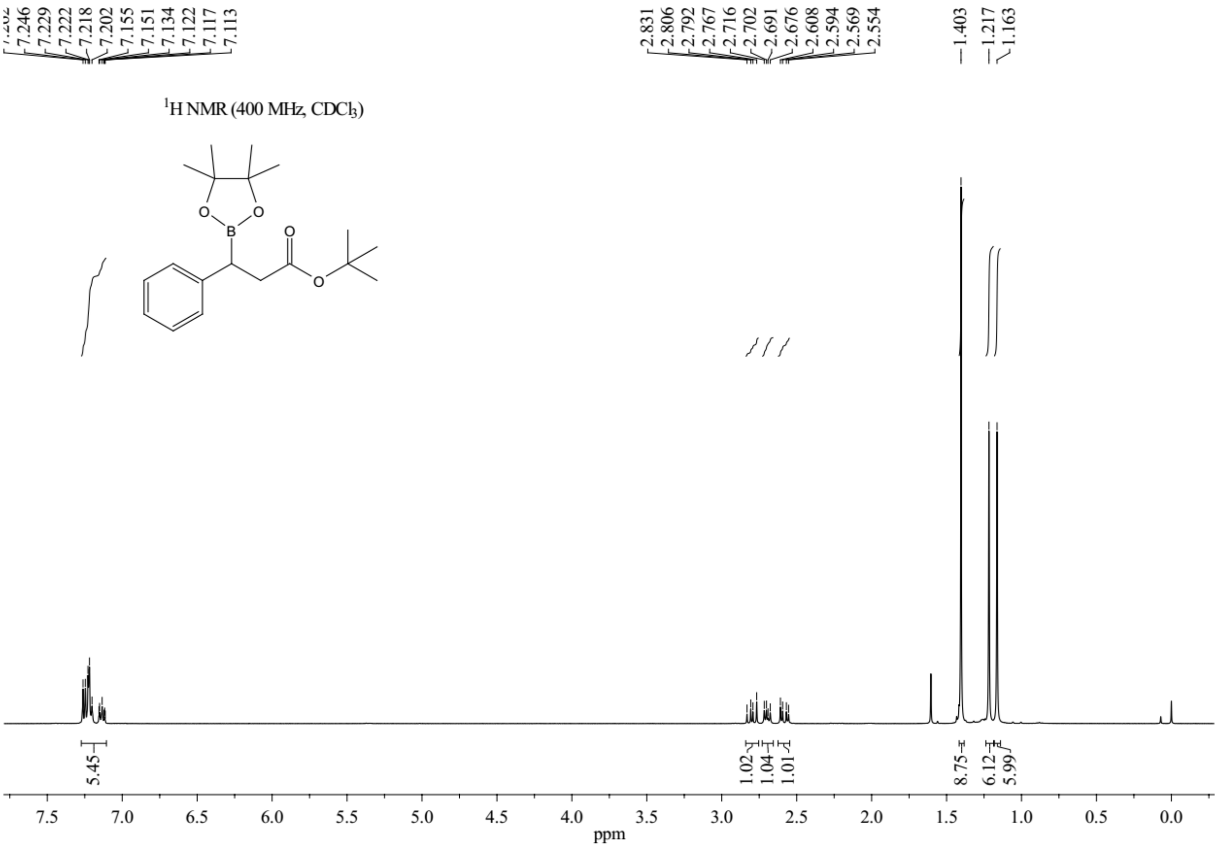

Supplementary Figure 10. ^{13}C NMR spectrum for 1c

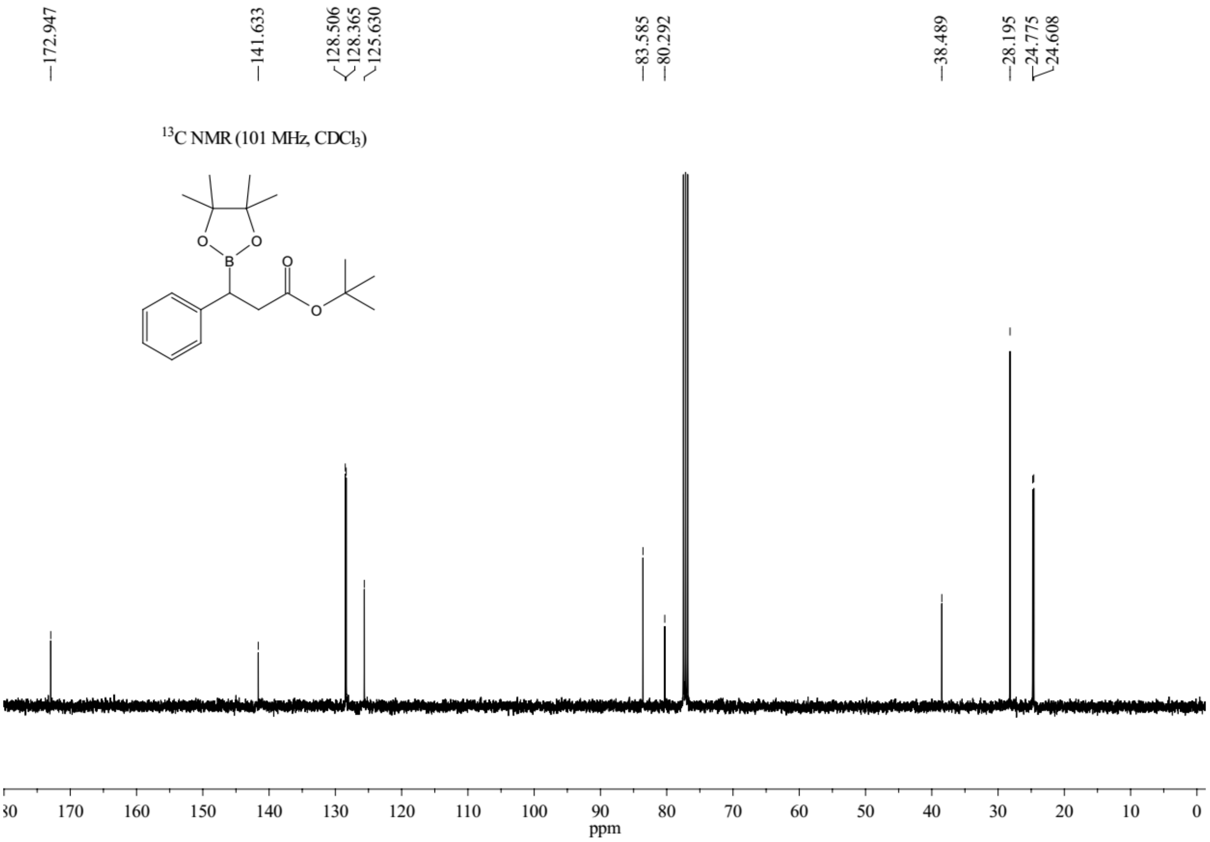

Supplementary Figure 11. ^{1}H NMR spectrum for Pentan-3-yl-3-phenyl-3-(4,4,5,5-tetramethyl-1,3,2-dioxaborolan-2-yl)propanoate (1b)

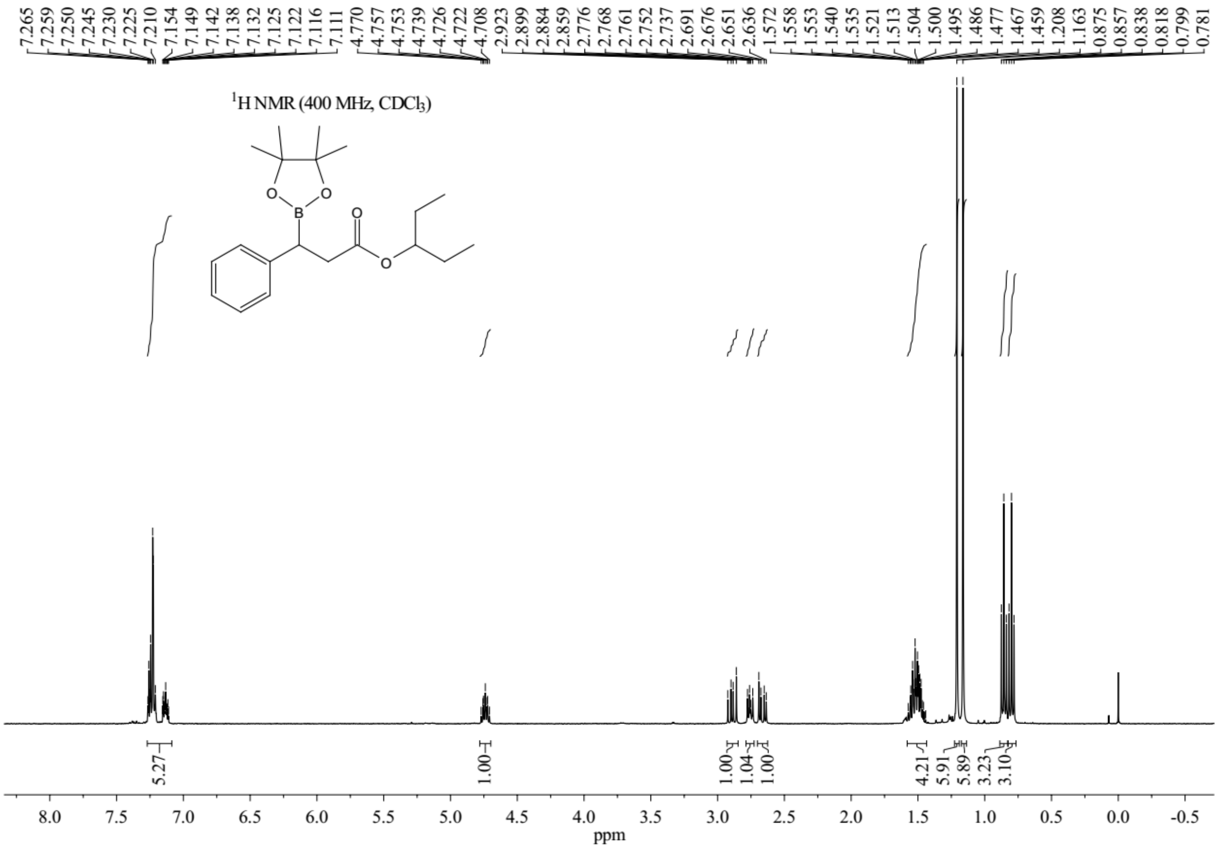

Supplementary Figure 12. ^{13}C NMR spectrum for 1b

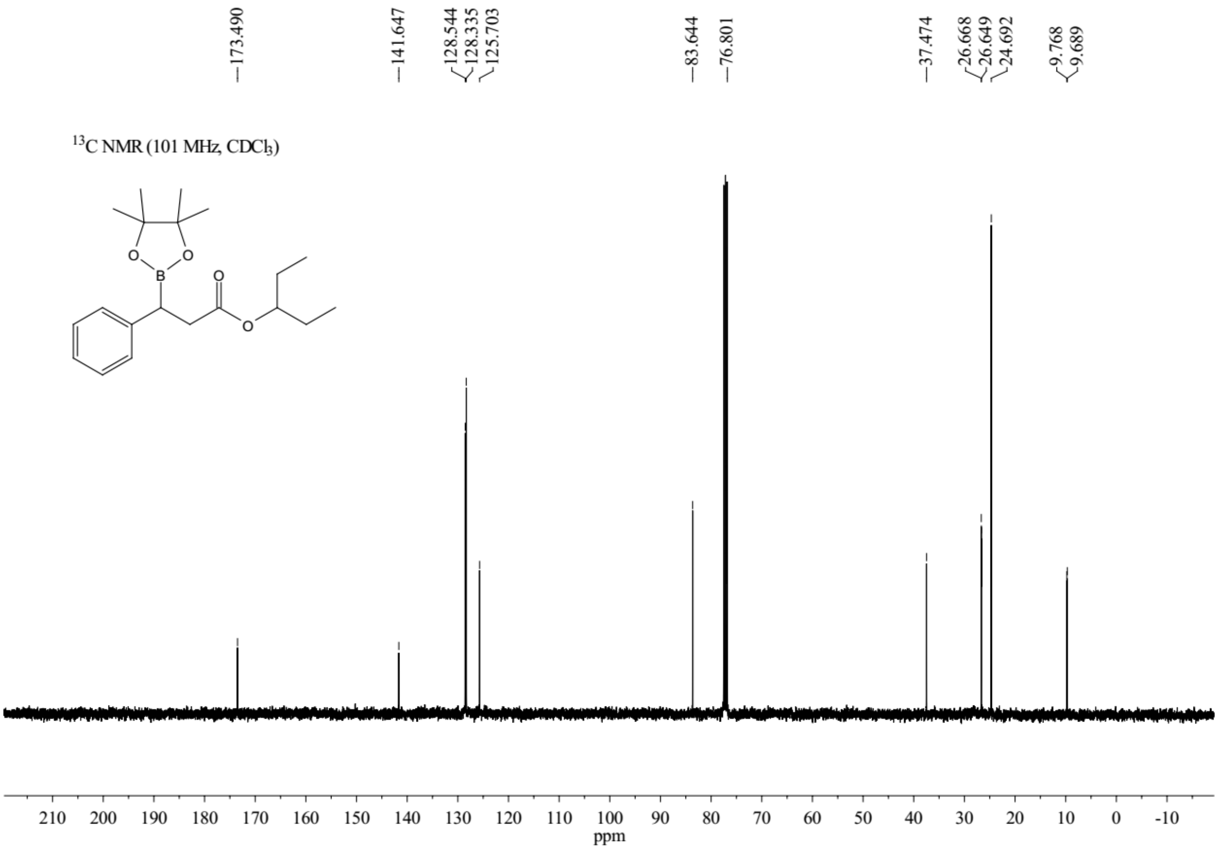

Supplementary Figure 13. ^{1}H NMR spectrum for *tert*-Butyl-3-(4-fluorophenyl)-3-(4,4,5,5-tetramethyl-1,3,2-dioxaborolan-2-yl)propanoate (5a′)

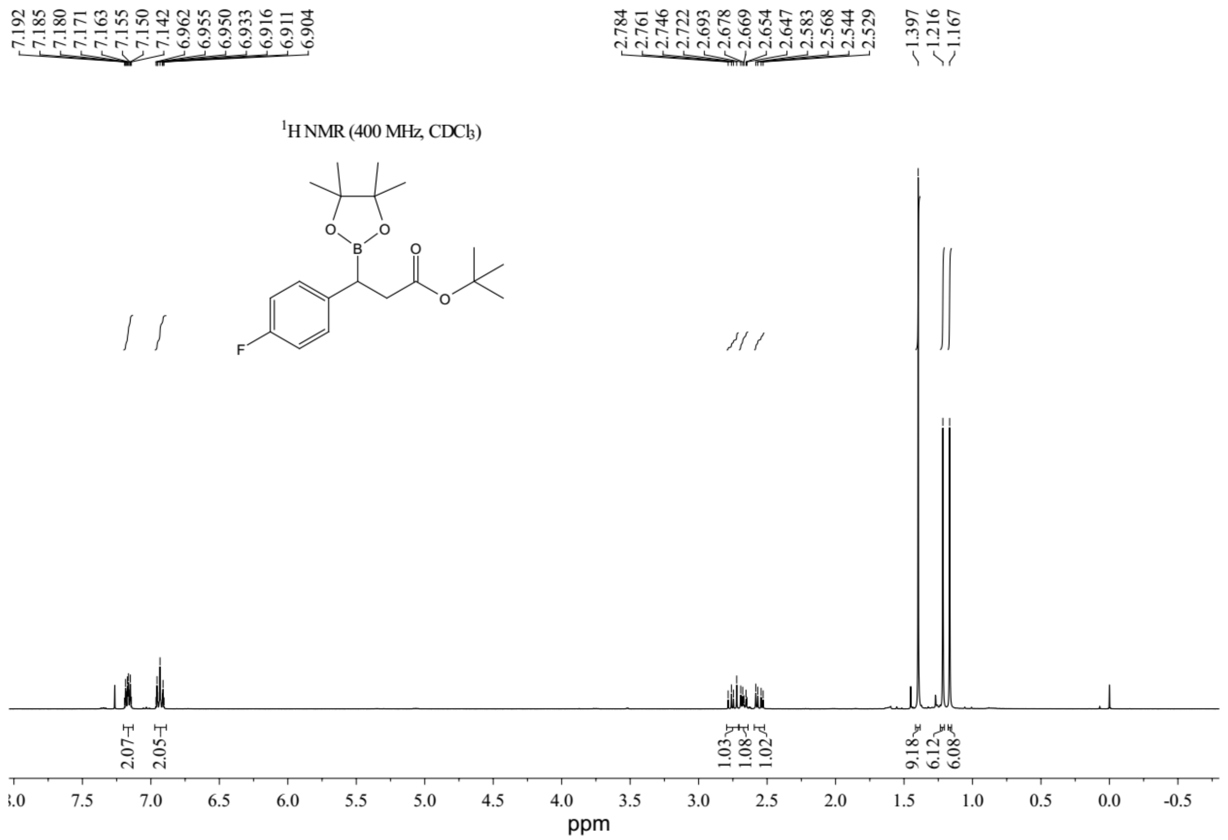

Supplementary Figure 14. ^{13}C NMR spectrum for 5a′

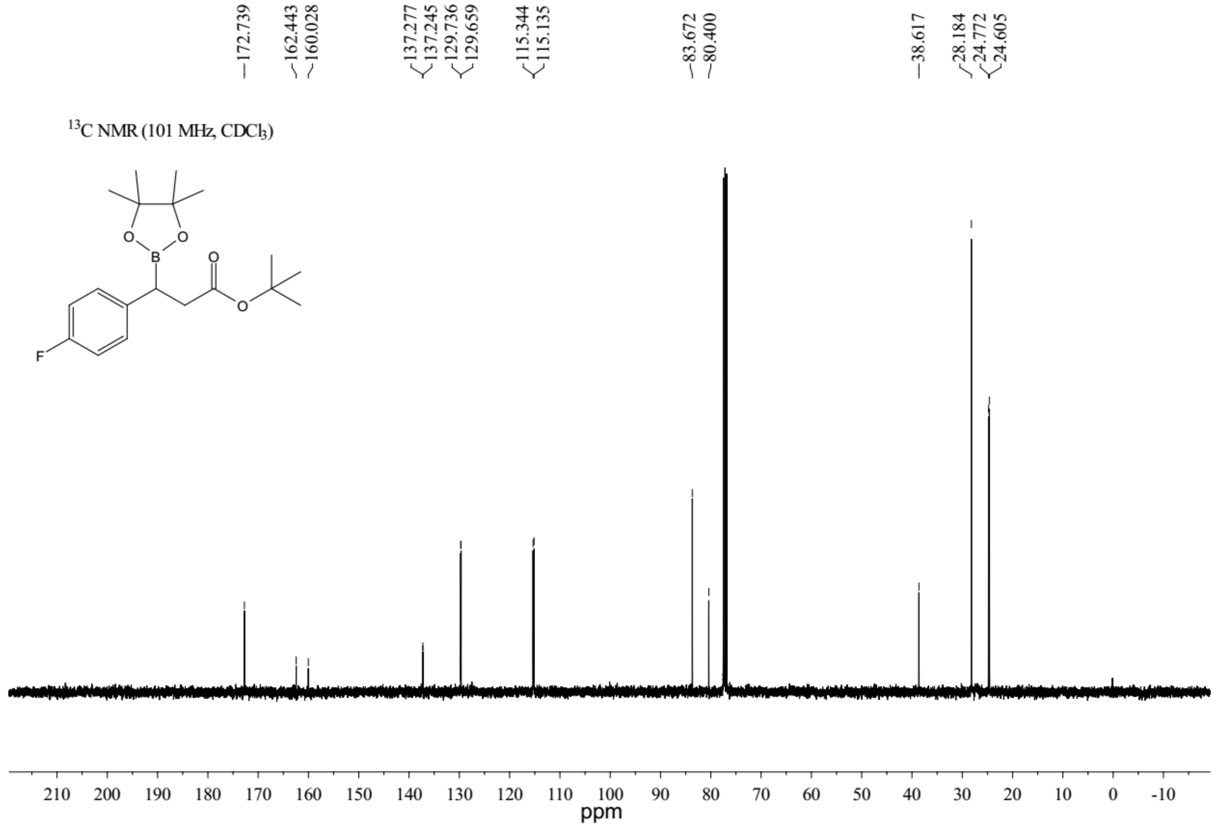

Supplementary Figure 15. ^{1}H NMR spectrum for Pentan-3-yl-3-(4-fluorophenyl)-3-(4,4,5,5-tetramethyl-1,3,2-dioxaborolan-2-yl)propanoate (5a)

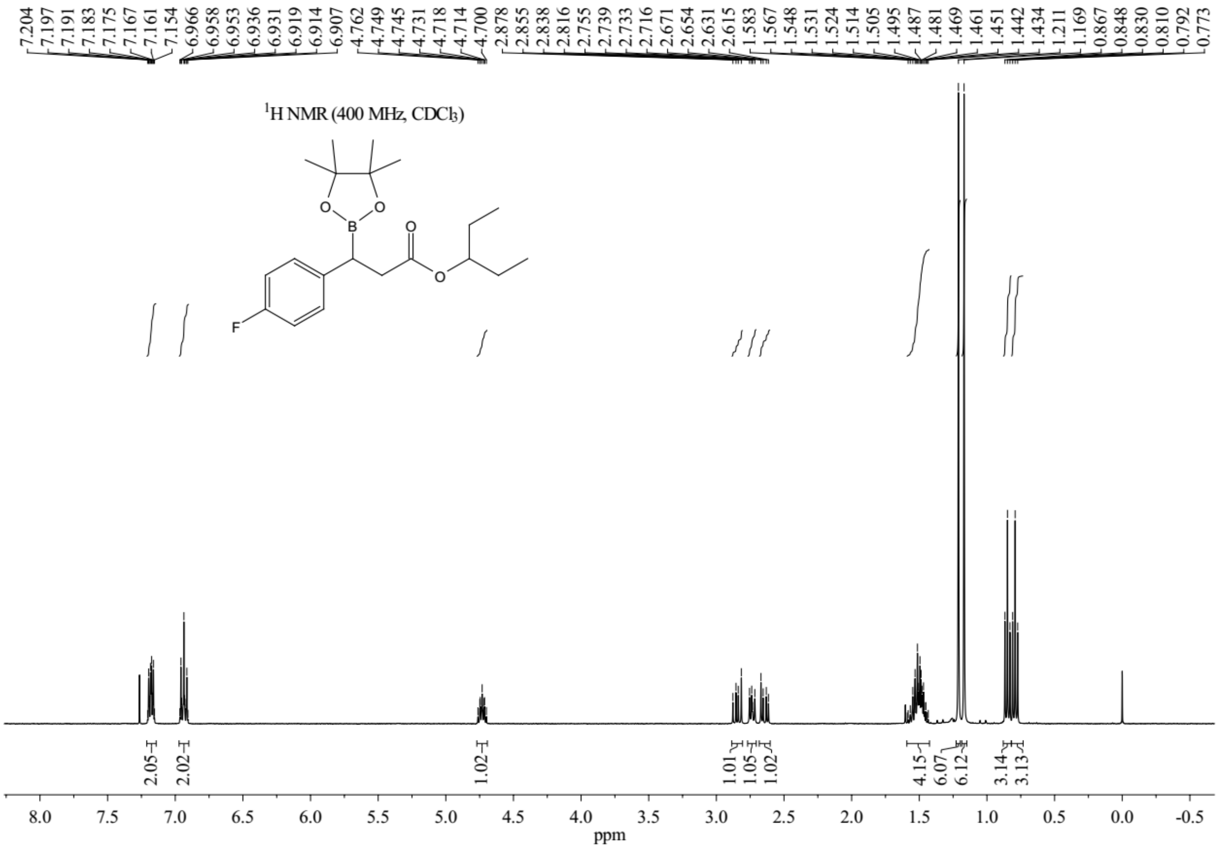

Supplementary Figure 16. ^{13}C NMR spectrum for 5a

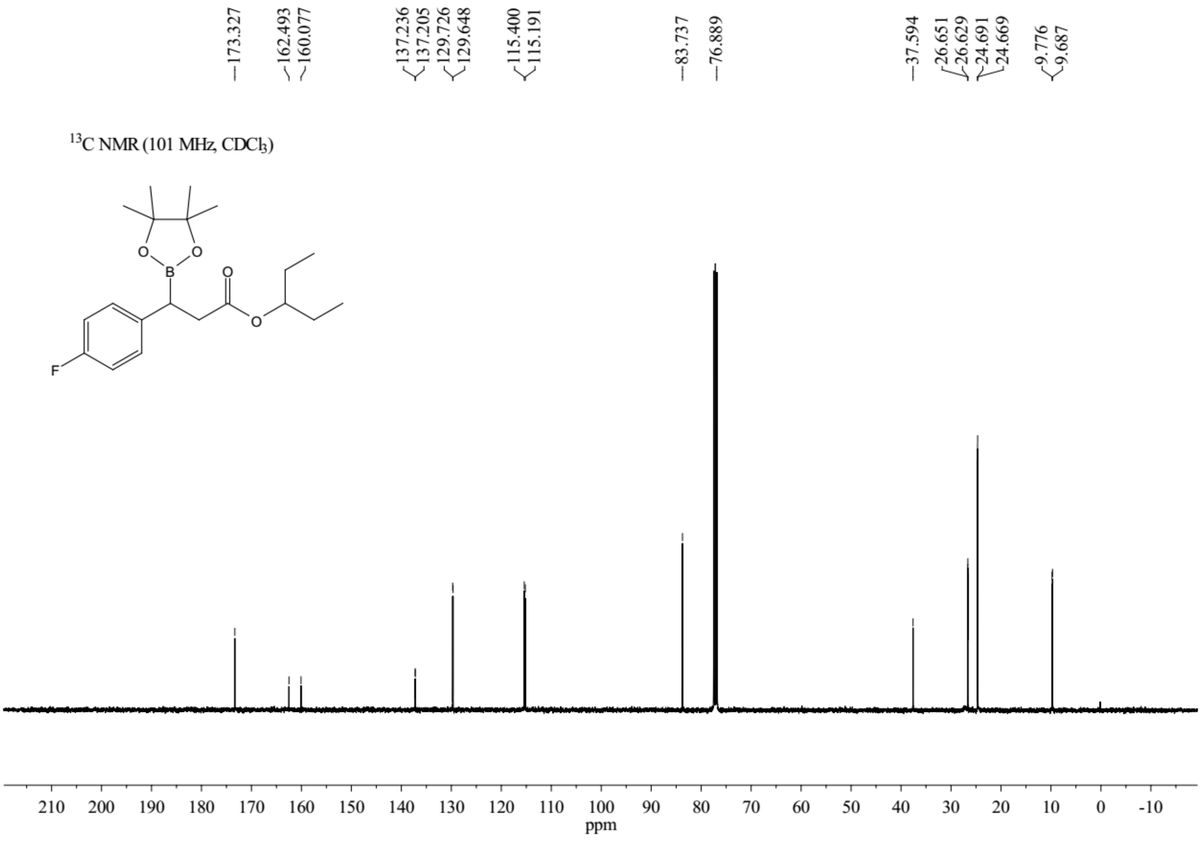

Supplementary Figure 17. ^{1}H NMR spectrum for *tert*-Butyl-3-(3-chlorophenyl)-3-(4,4,5,5-tetramethyl-1,3,2-dioxaborolan-2-yl)propanoate (5b′)

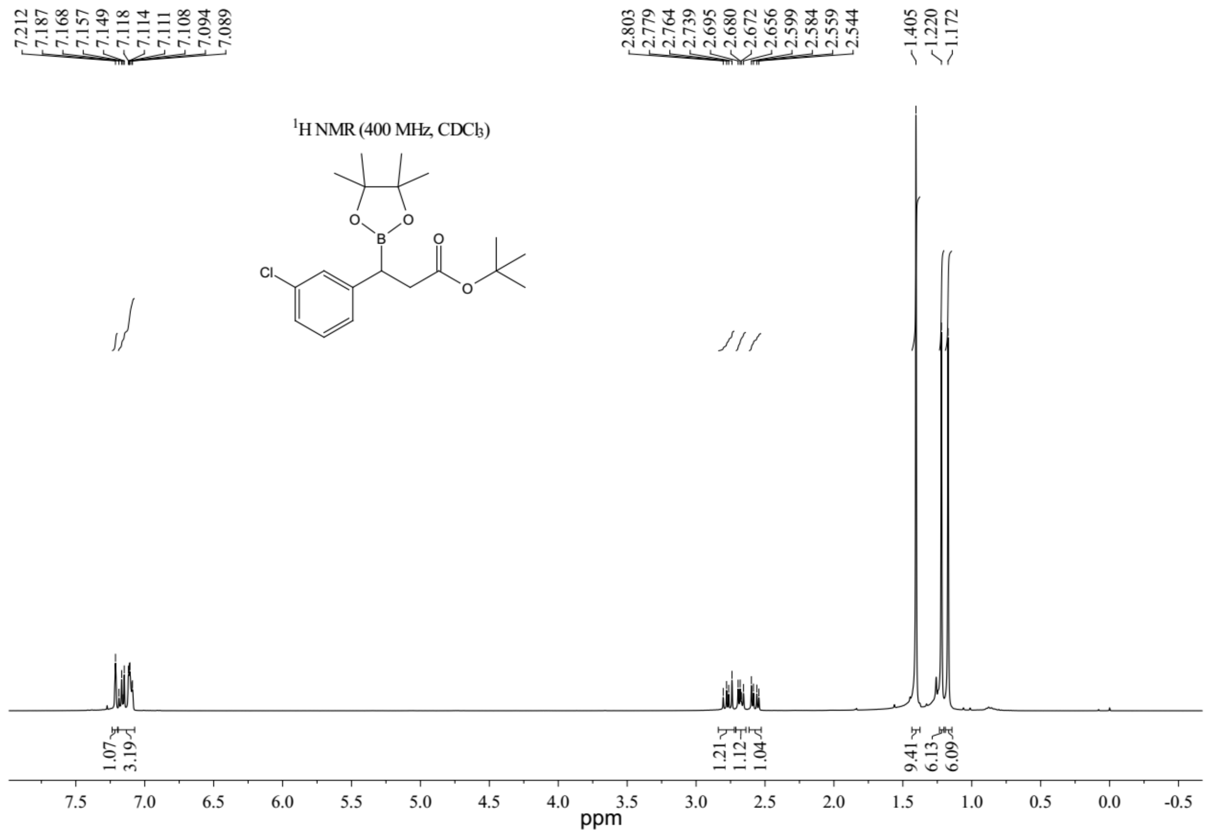

Supplementary Figure 18. ^{13}C NMR spectrum for 5b′

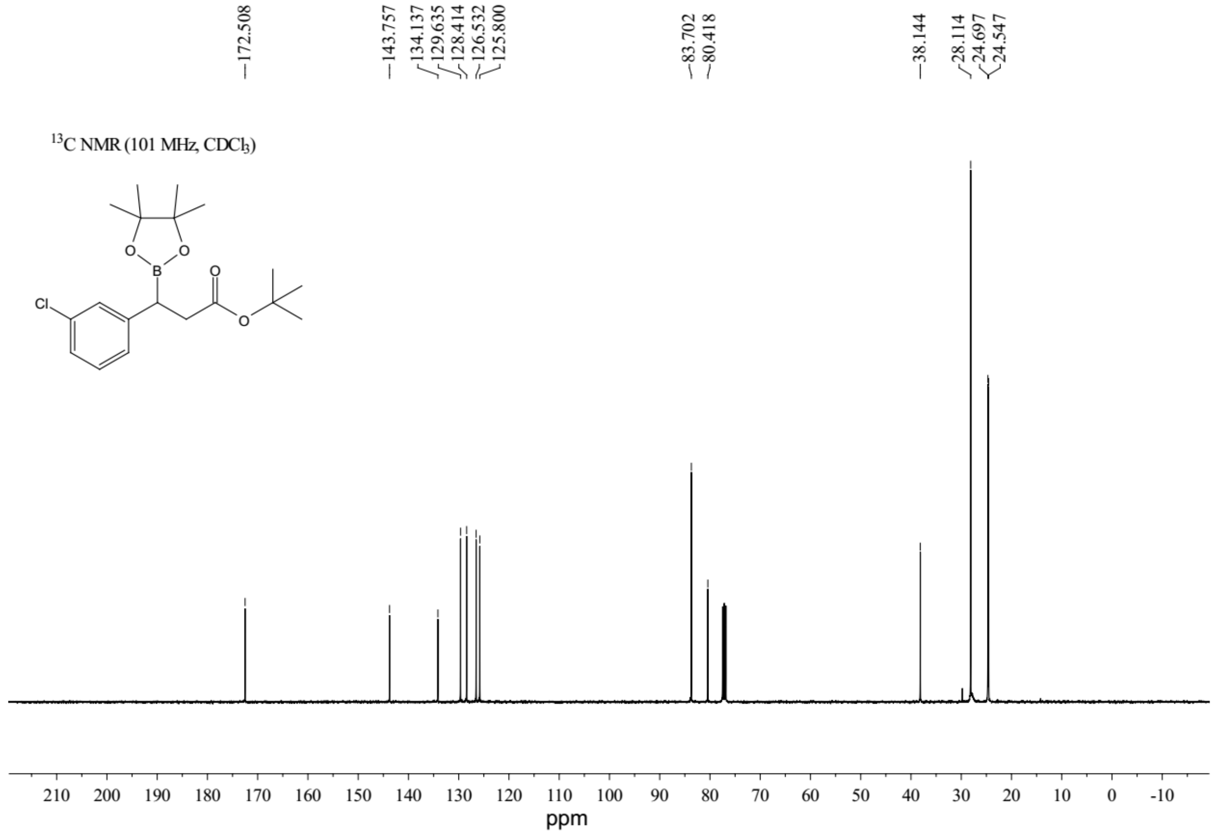

Supplementary Figure 19. ¹H NMR spectrum for Pentan-3-yl-3-(3-chlorophenyl)-3-(4,4,5,5-tetramethyl-1,3,2-dioxaborolan-2-yl)propanoate (5b)

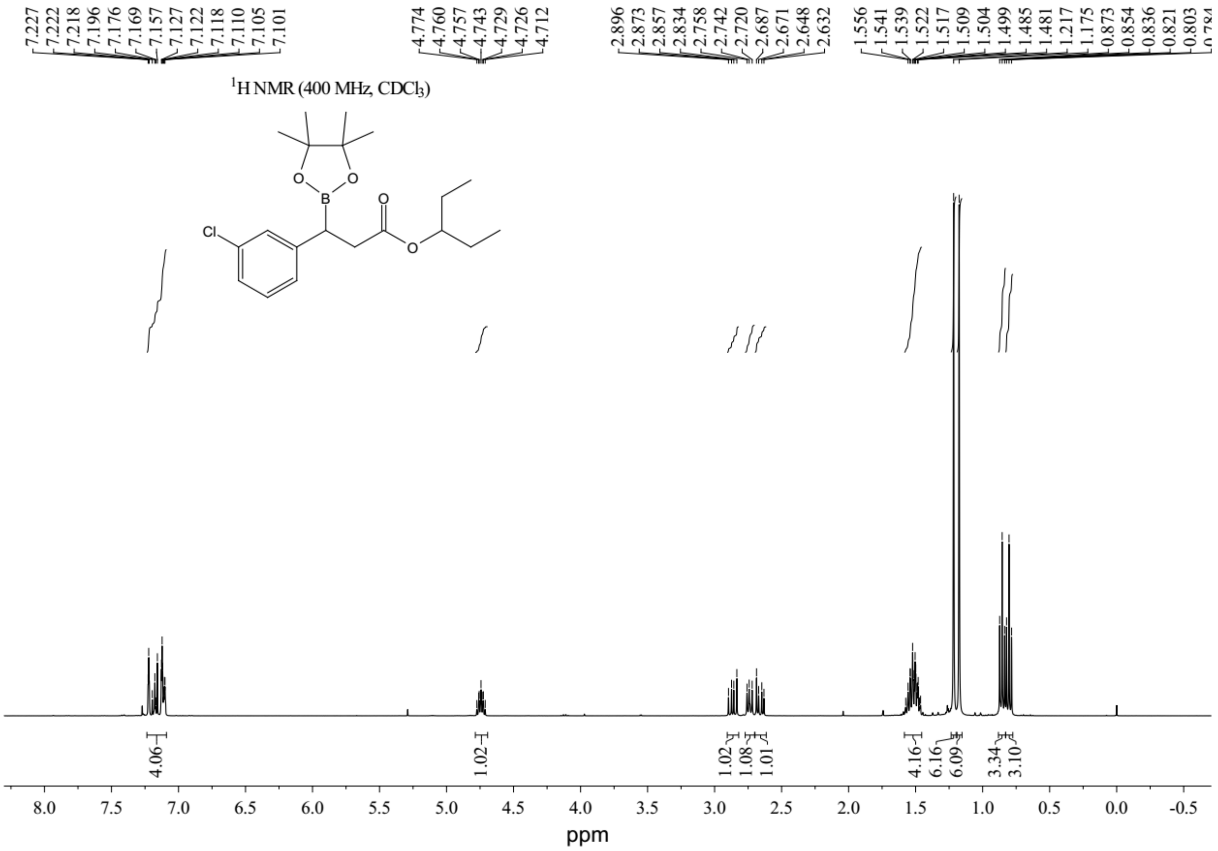

Supplementary Figure 20. ¹³C NMR spectrum for 5b

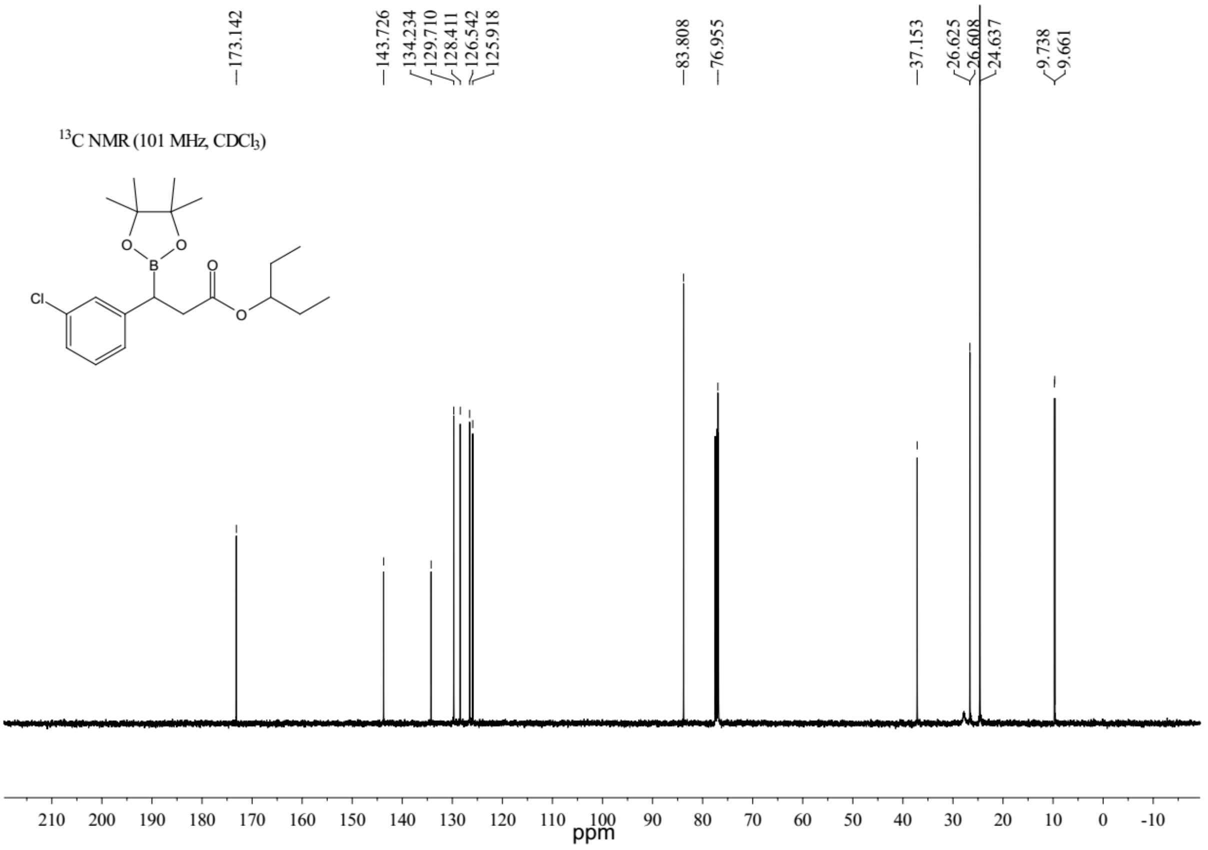

Supplementary Figure 21. ^{1}H NMR spectrum for *tert*-Butyl-3-(3-bromophenyl)-3-(4,4,5,5-tetramethyl-1,3,2-dioxaborolan-2-yl)propanoate (5c′)

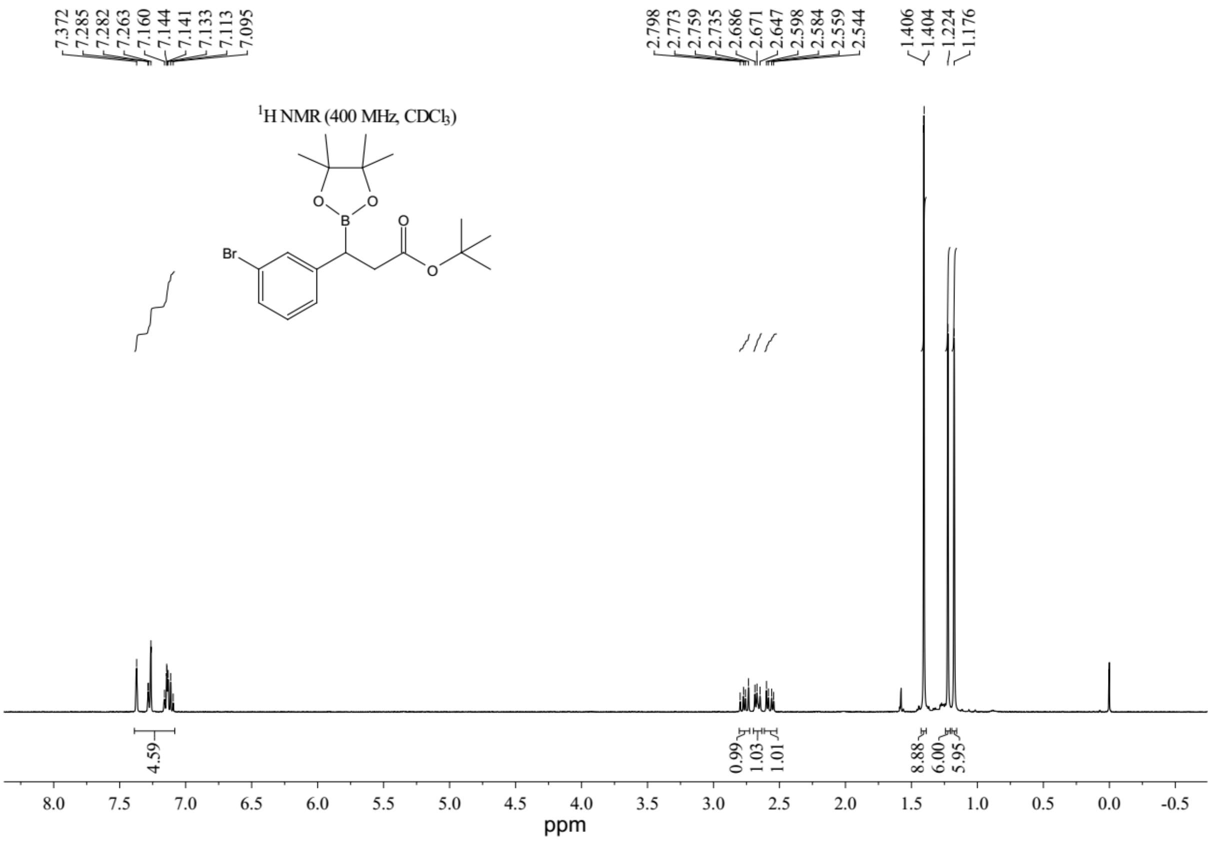

Supplementary Figure 22. ^{13}C NMR spectrum for 5c′

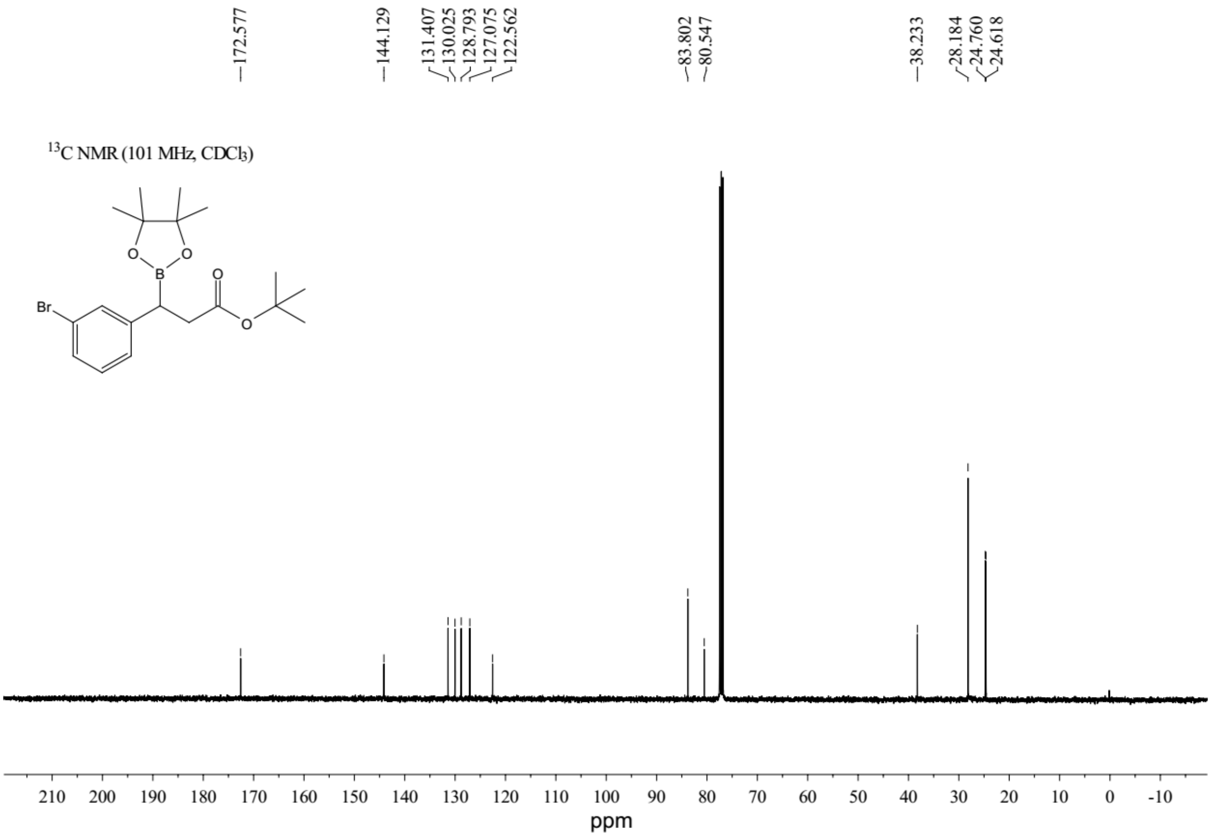

Supplementary Figure 23. ^{1}H NMR spectrum for Pentan-3-yl-3-(3-bromophenyl)-3-(4,4,5,5-tetramethyl-1,3,2-dioxaborolan-2-yl)propanoate (5c)

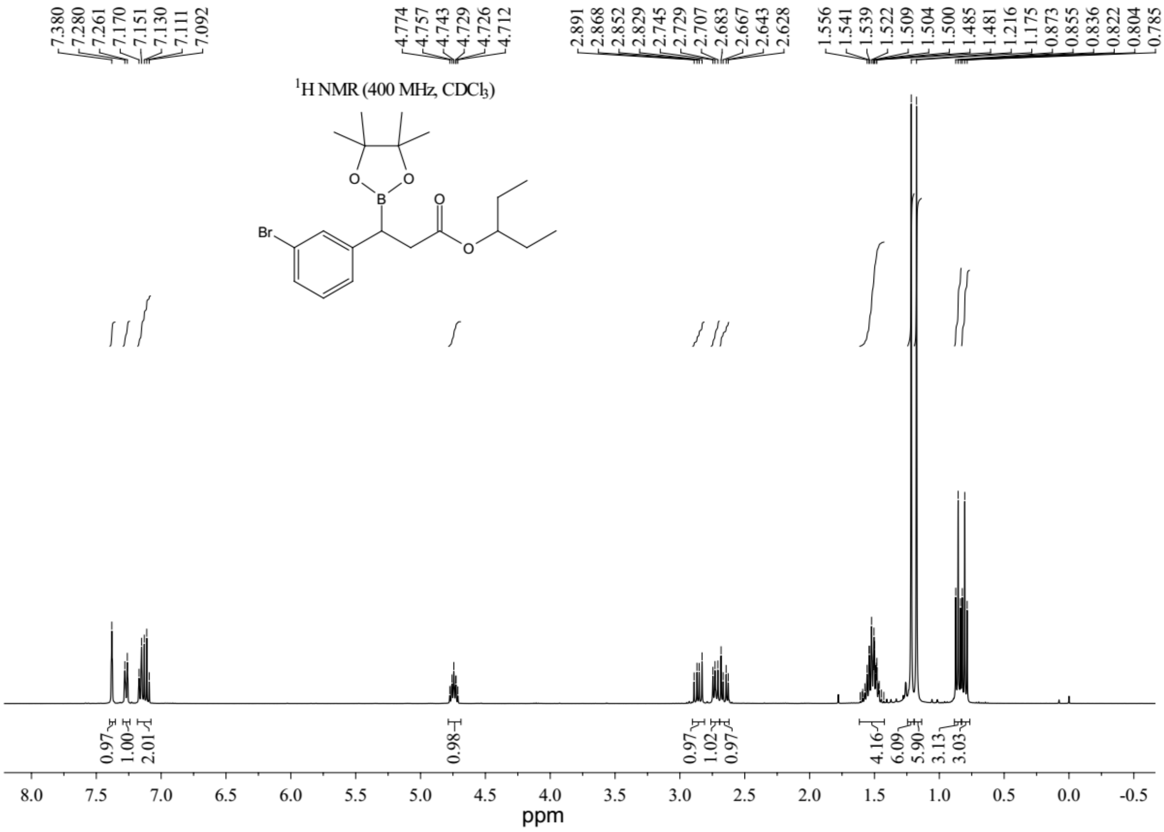

Supplementary Figure 24. ^{13}C NMR spectrum for 5c

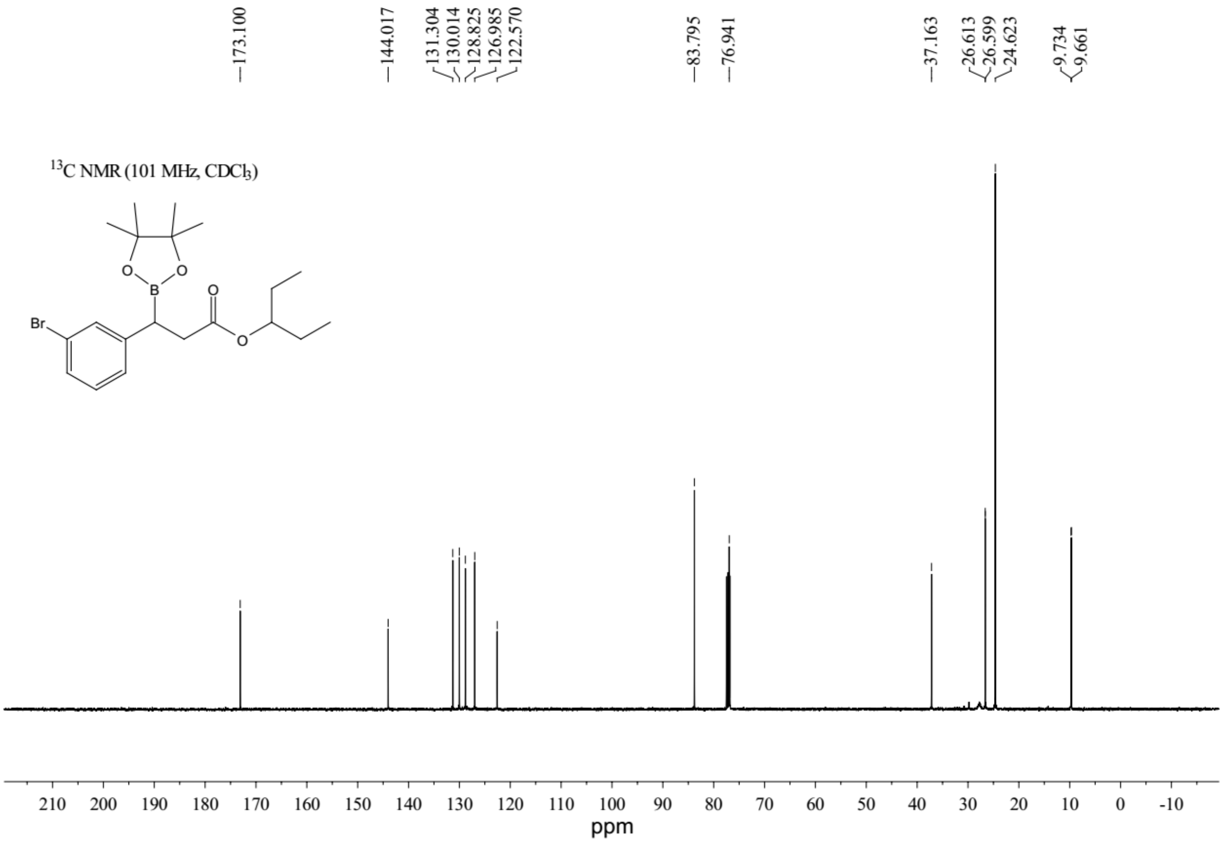

Supplementary Figure 25. ^{1}H NMR spectrum for *tert*-Butyl-3-(4,4,5,5-tetramethyl-1,3,2-dioxaborolan-2-yl)-3-(3-(trifluoromethoxy)phenyl)propano ate (5d′)

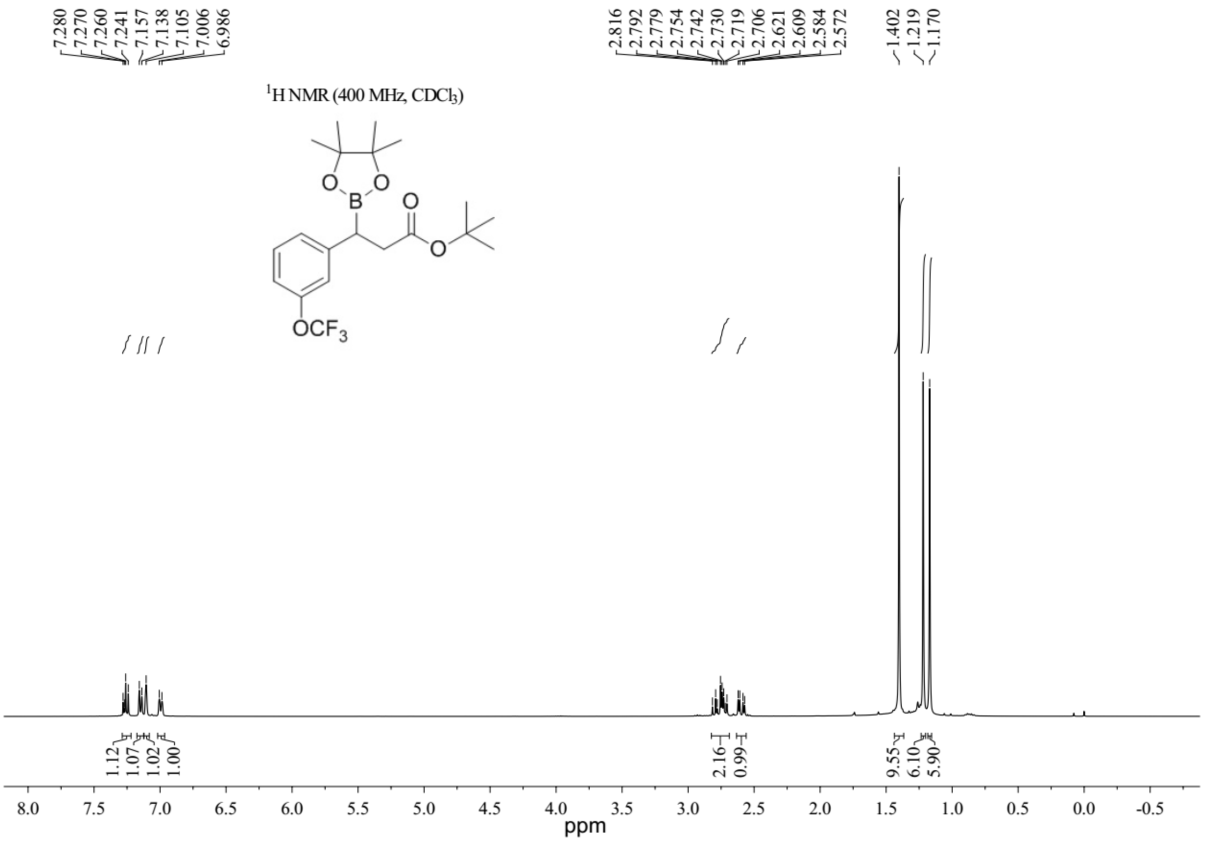

Supplementary Figure 26. ^{13}C NMR spectrum for 5d′

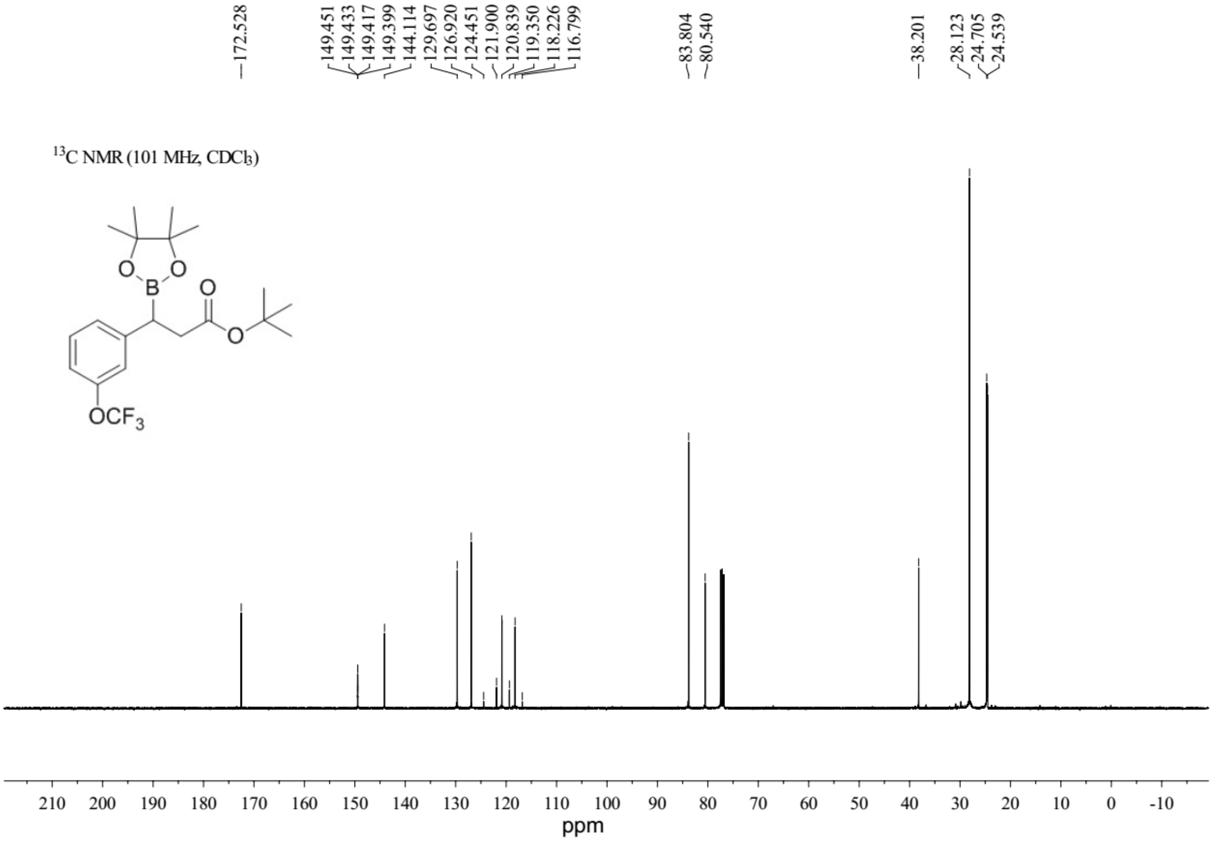

Supplementary Figure 27. ^{1}H NMR spectrum for Pentan-3-yl-3-(4,4,5,5-tetramethyl-1,3,2-dioxaborolan-2-yl)-3-(3-(trifluoromethoxy)phenyl)propa noate (5d)

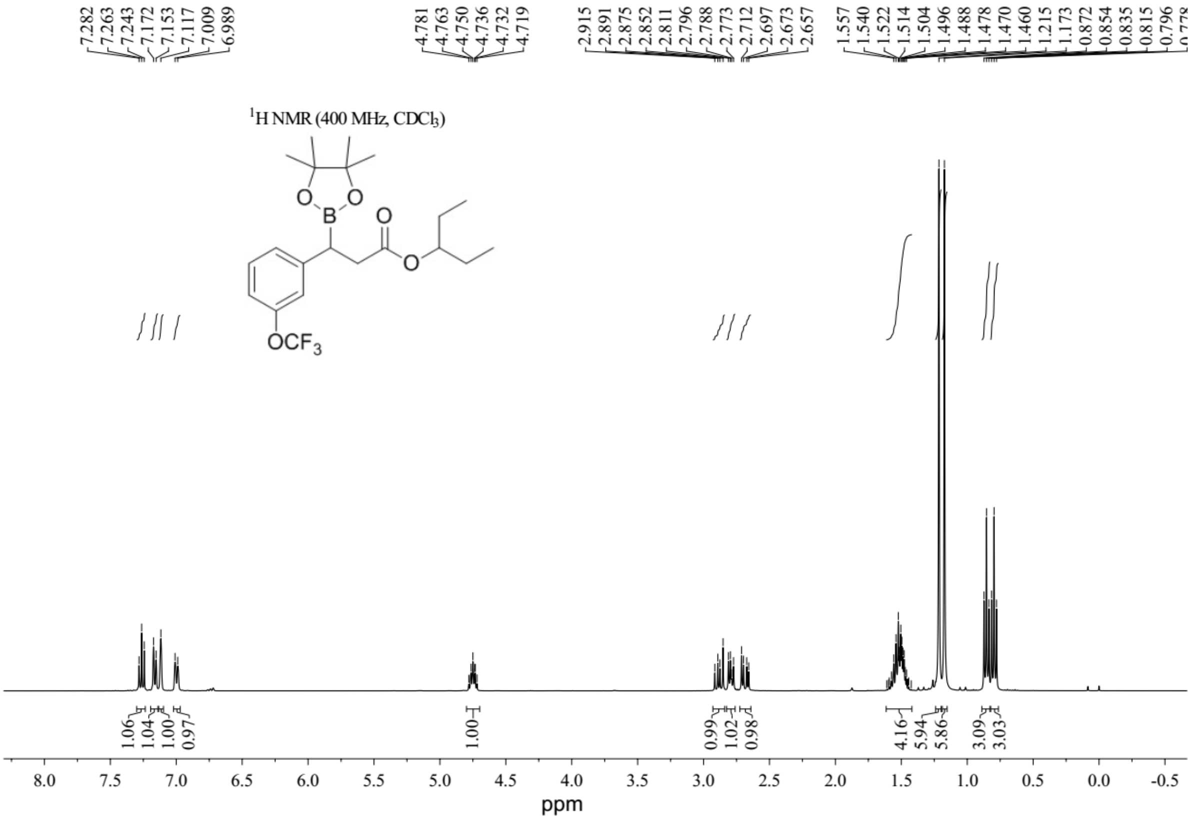

Supplementary Figure 28. ^{13}C NMR spectrum for 5d

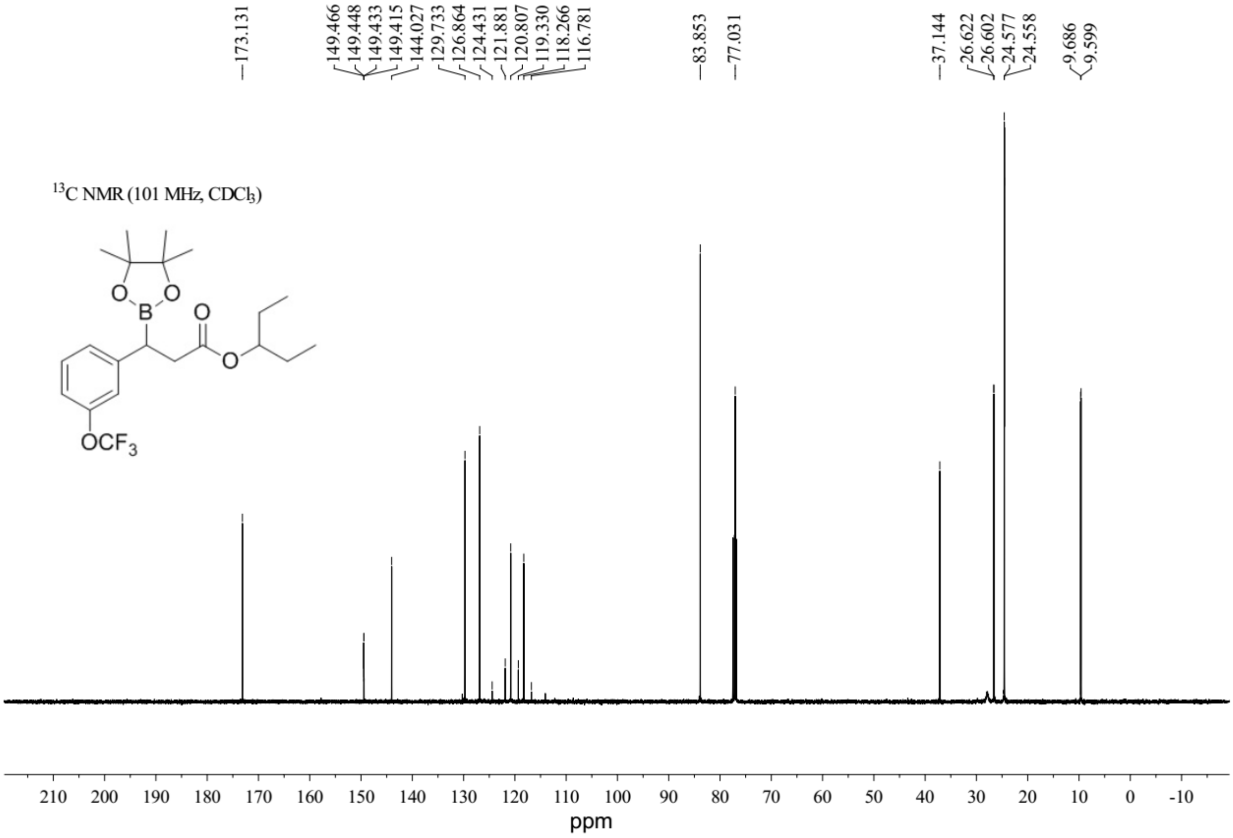

Supplementary Figure 29. ^{1}H NMR spectrum for *tert*-Butyl-3-(4-methoxyphenyl)-3-(4,4,5,5-tetramethyl-1,3,2-dioxaborolan-2-yl)propanoate (5e′)

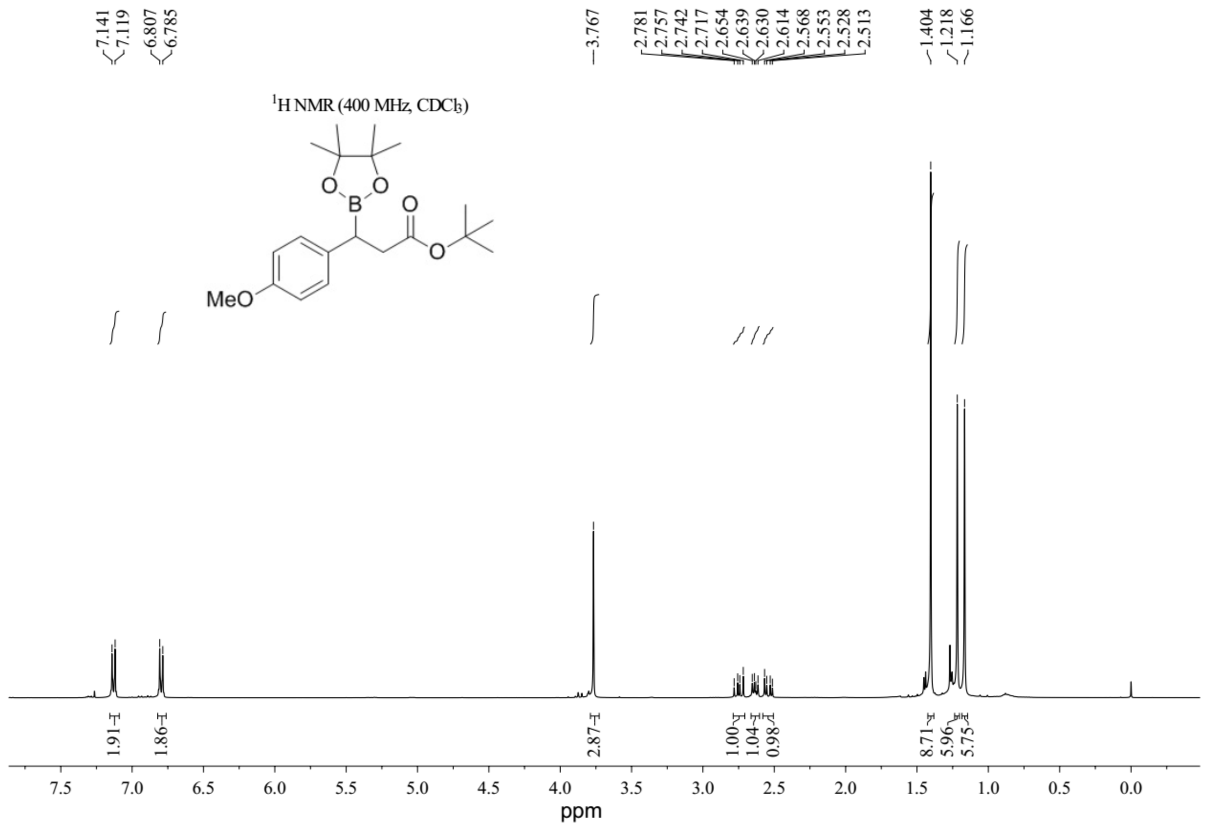

Supplementary Figure 30. ^{13}C NMR spectrum for 5e′

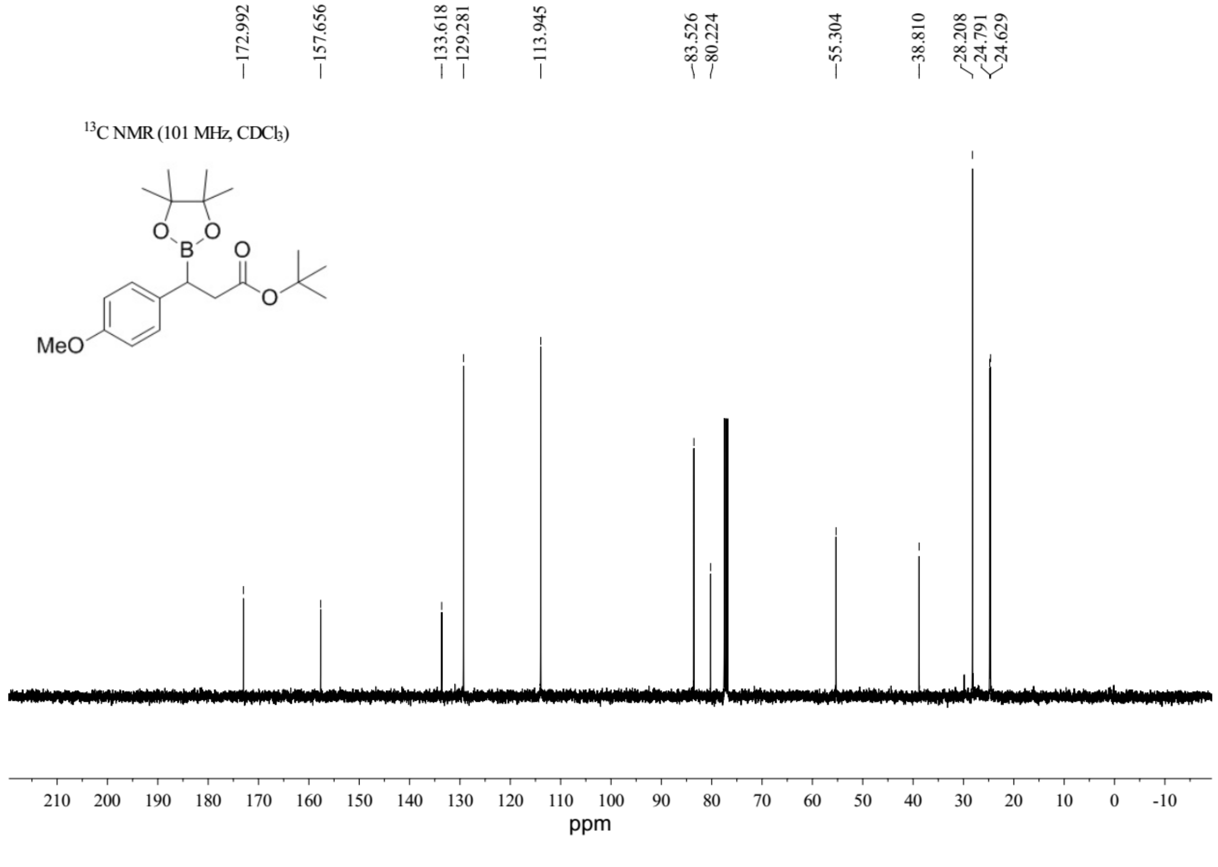

Supplementary Figure 31. ^{1}H NMR spectrum for Pentan-3-yl-3-(4-methoxyphenyl)-3-(4,4,5,5-tetramethyl-1,3,2-dioxaborolan-2-yl)propanoate (5e)

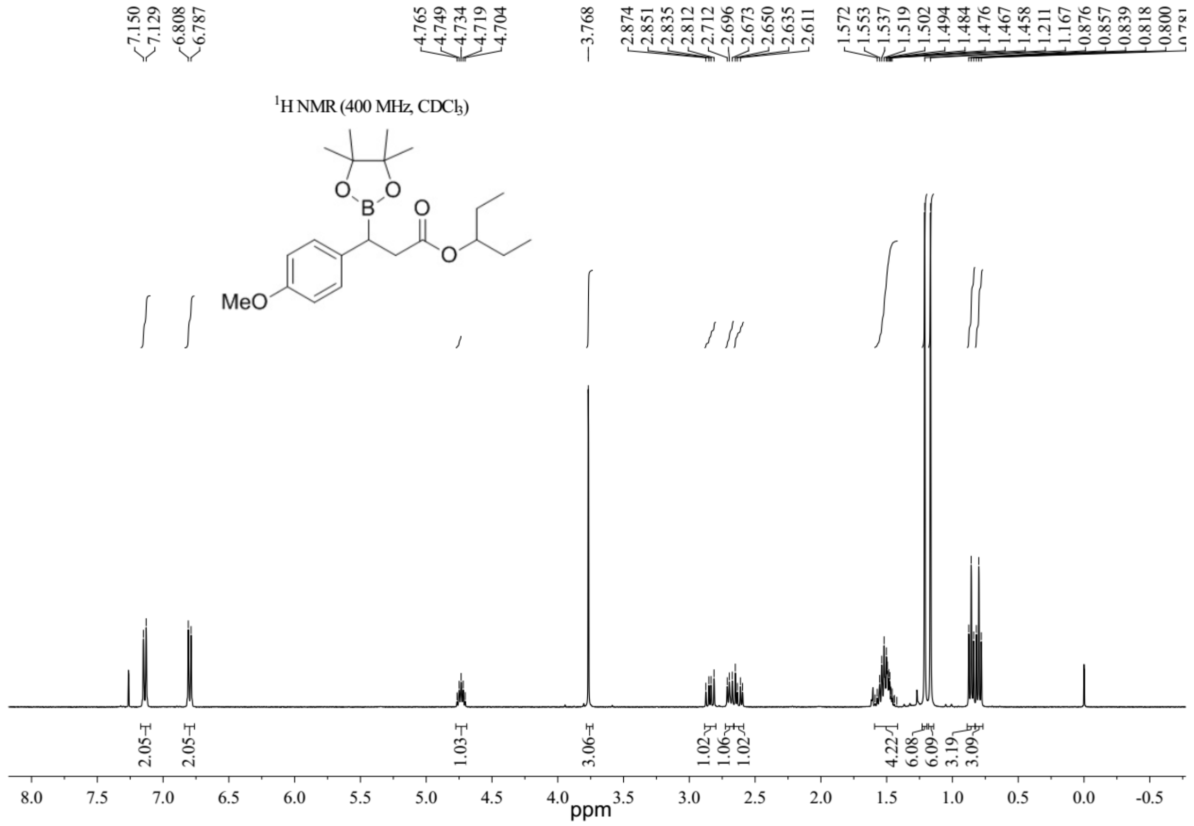

Supplementary Figure 32. ^{13}C NMR spectrum for 5e

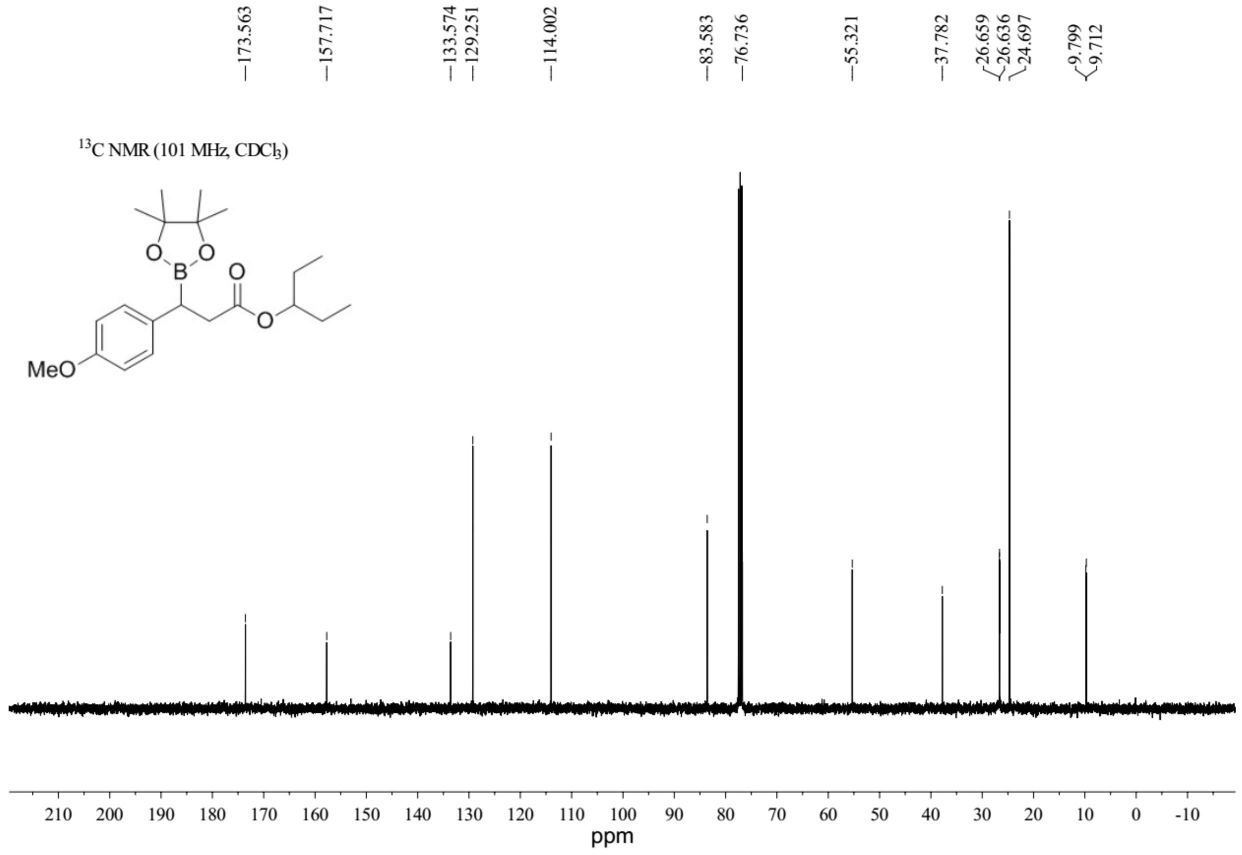

Supplementary Figure 33. ^{1}H NMR spectrum for *tert*-Butyl-3-(4,4,5,5-tetramethyl-1,3,2-dioxaborolan-2-yl)-3-(o-tolyl)propanoate (5f′)

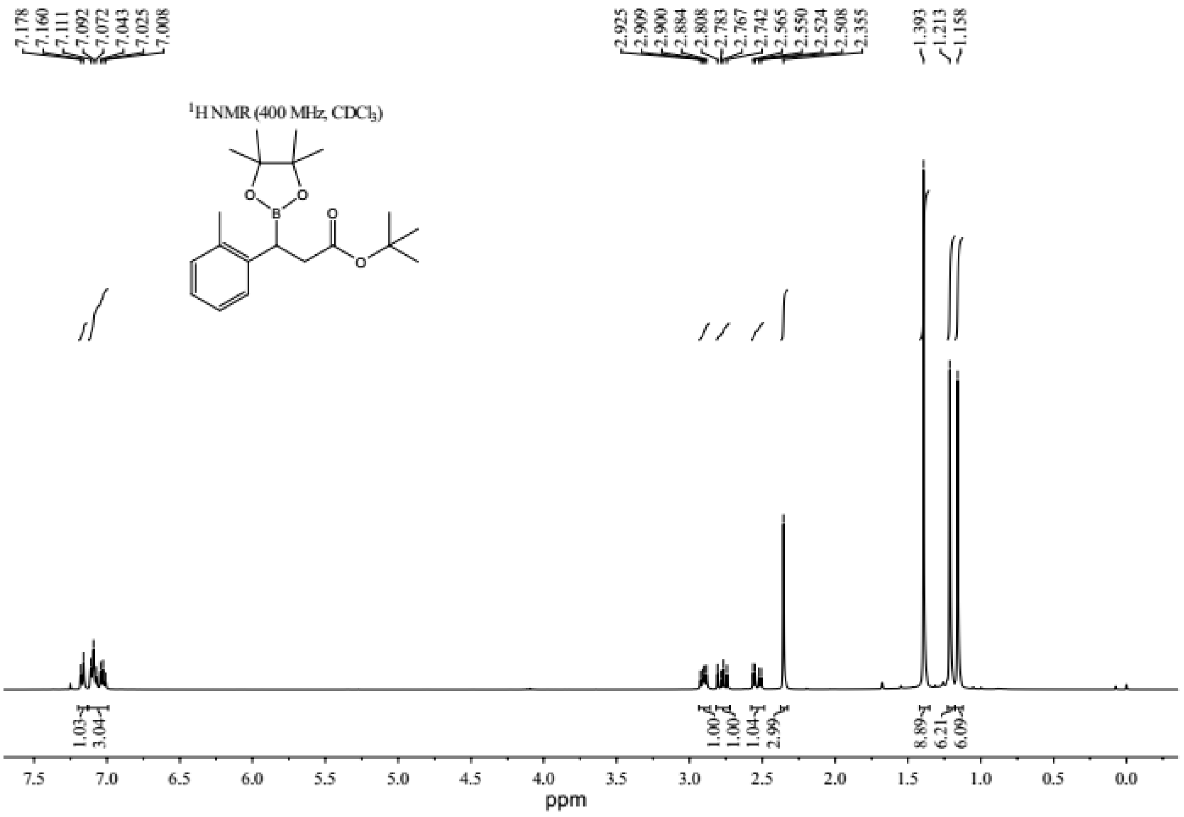

Supplementary Figure 34. ^{13}C NMR spectrum for 5f′

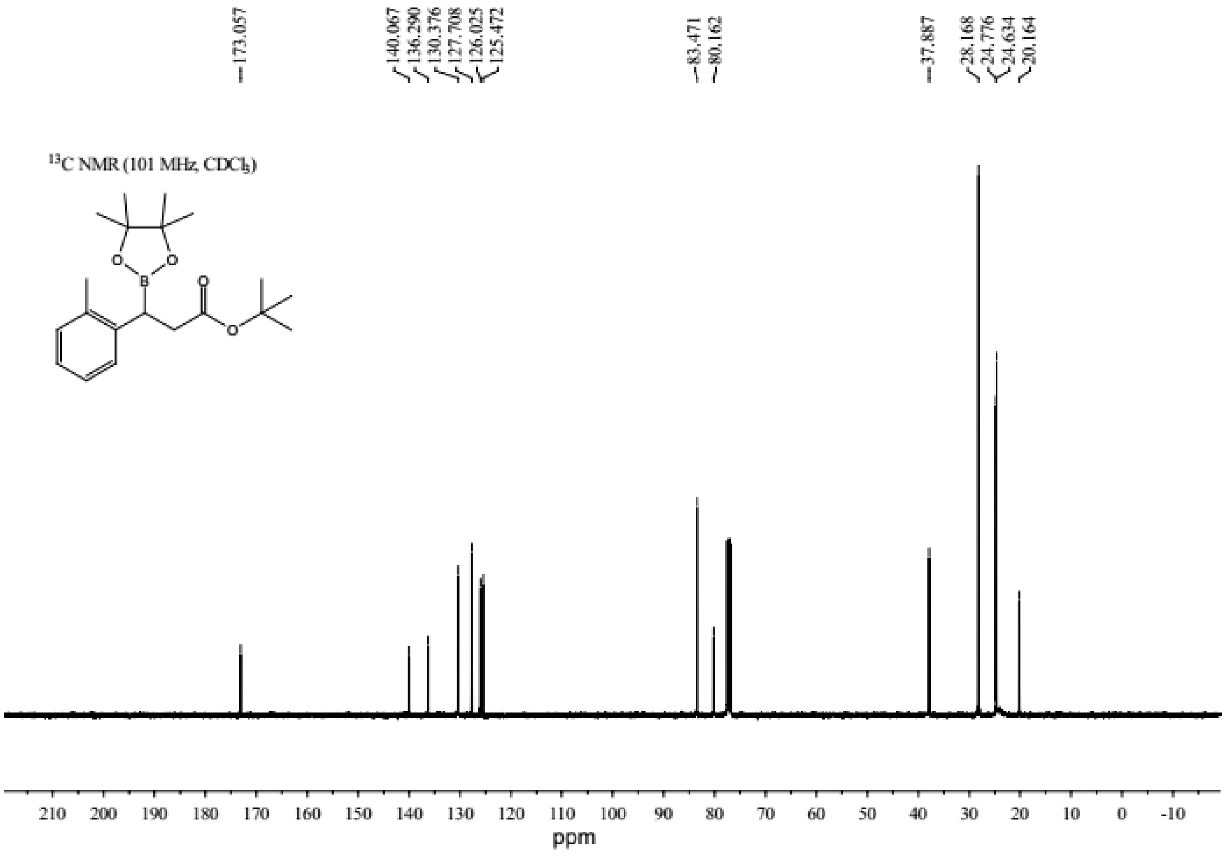

Supplementary Figure 35. ^{1}H NMR spectrum for Pentan-3-yl-3-(4,4,5,5-tetramethyl-1,3,2-dioxaborolan-2-yl)-3-(o-tolyl)propanoate (5f)

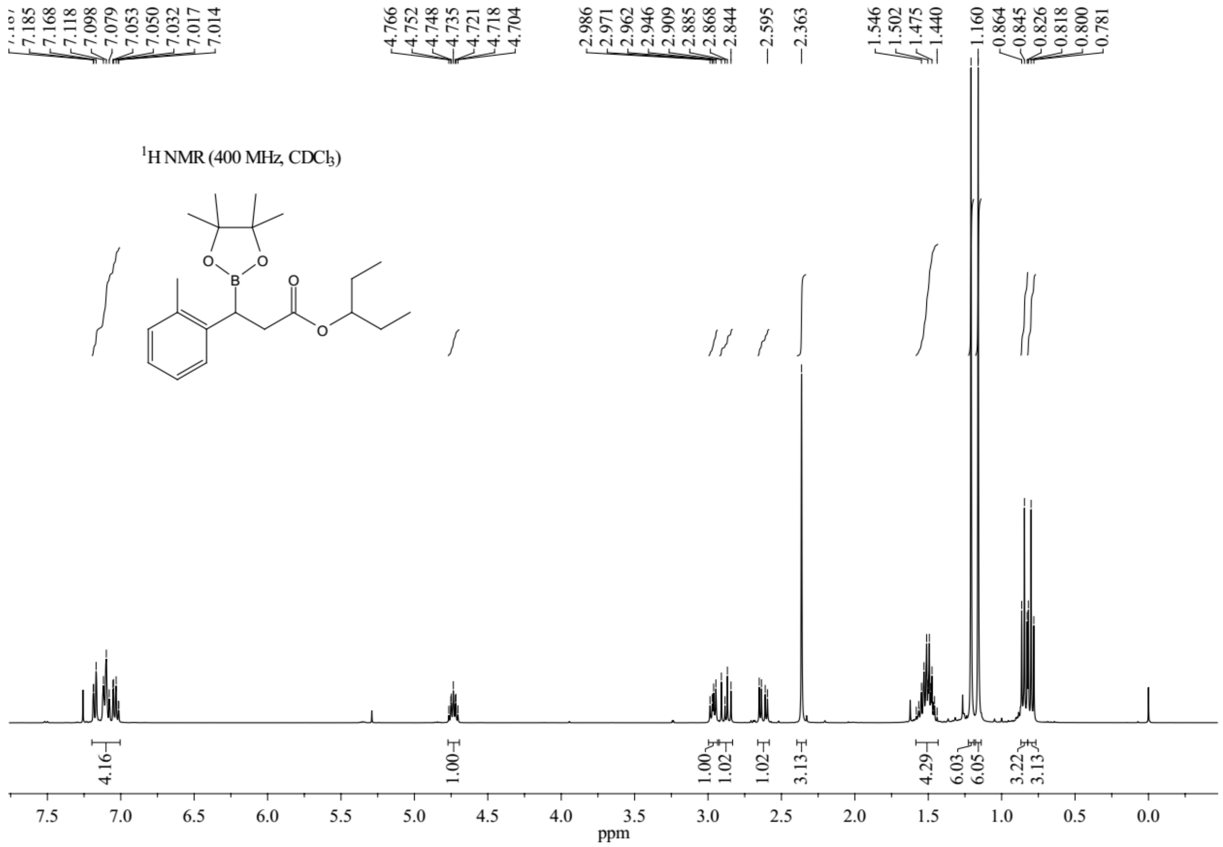

Supplementary Figure 36. ^{13}C NMR spectrum for 5f

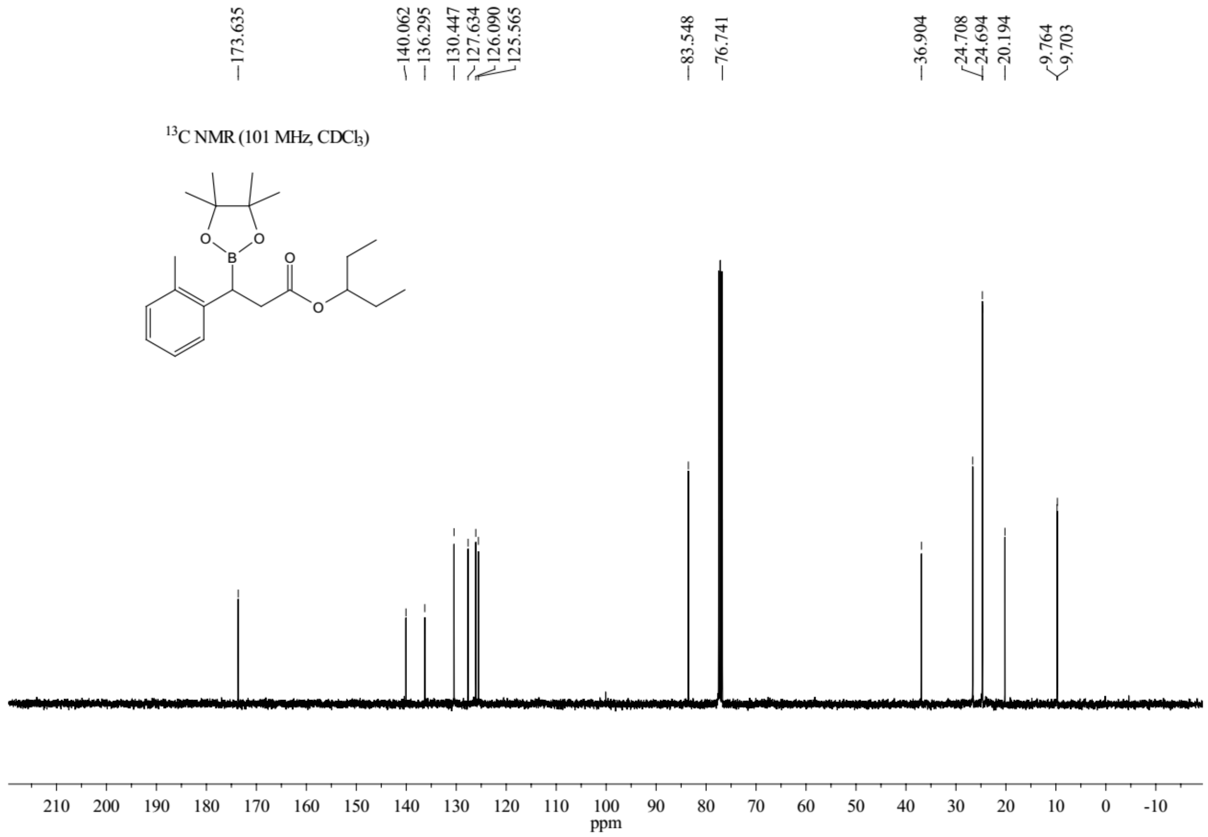

Supplementary Figure 37. ^{1}H NMR spectrum for *tert*-Butyl-3-(naphthalen-1-yl)-3-(4,4,5,5-tetramethyl-1,3,2-dioxaborolan-2-yl)propanoate (5g′)

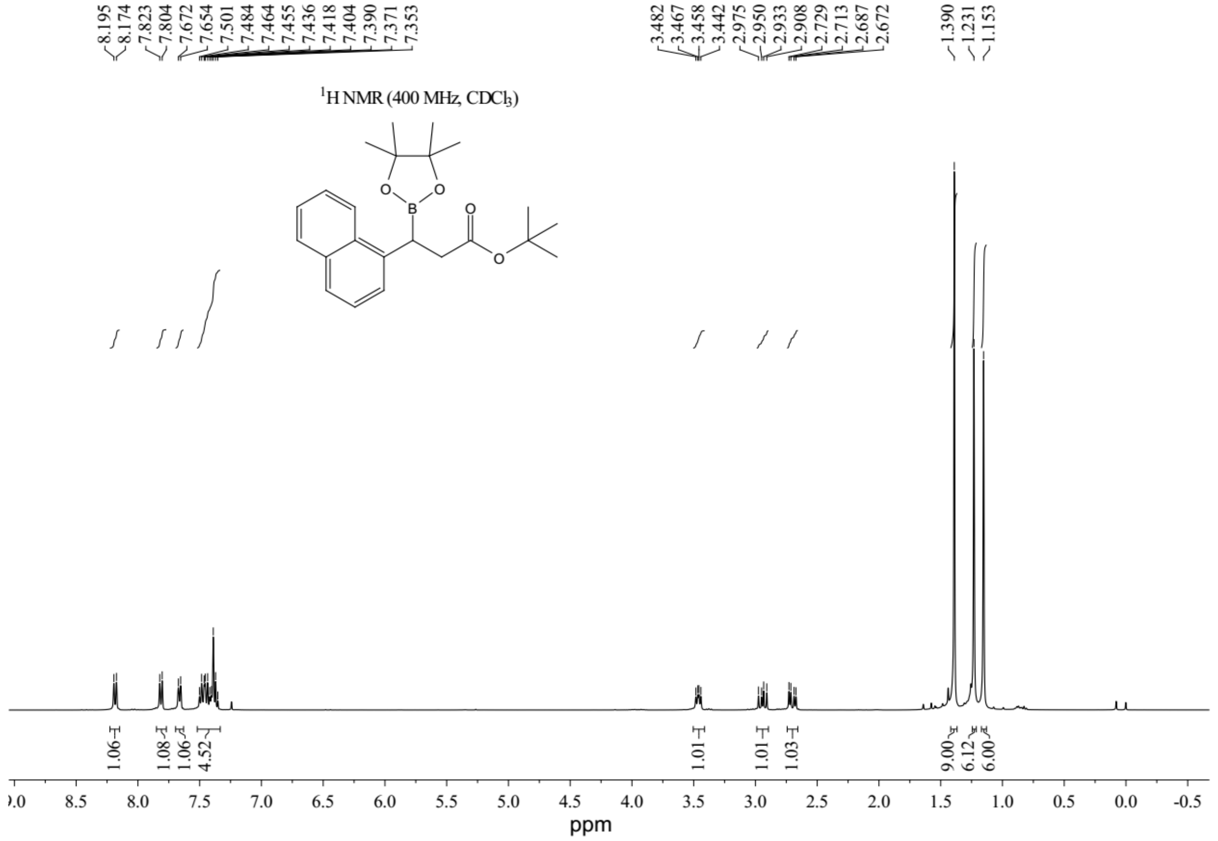

Supplementary Figure 38. ^{13}C NMR spectrum for 5g′

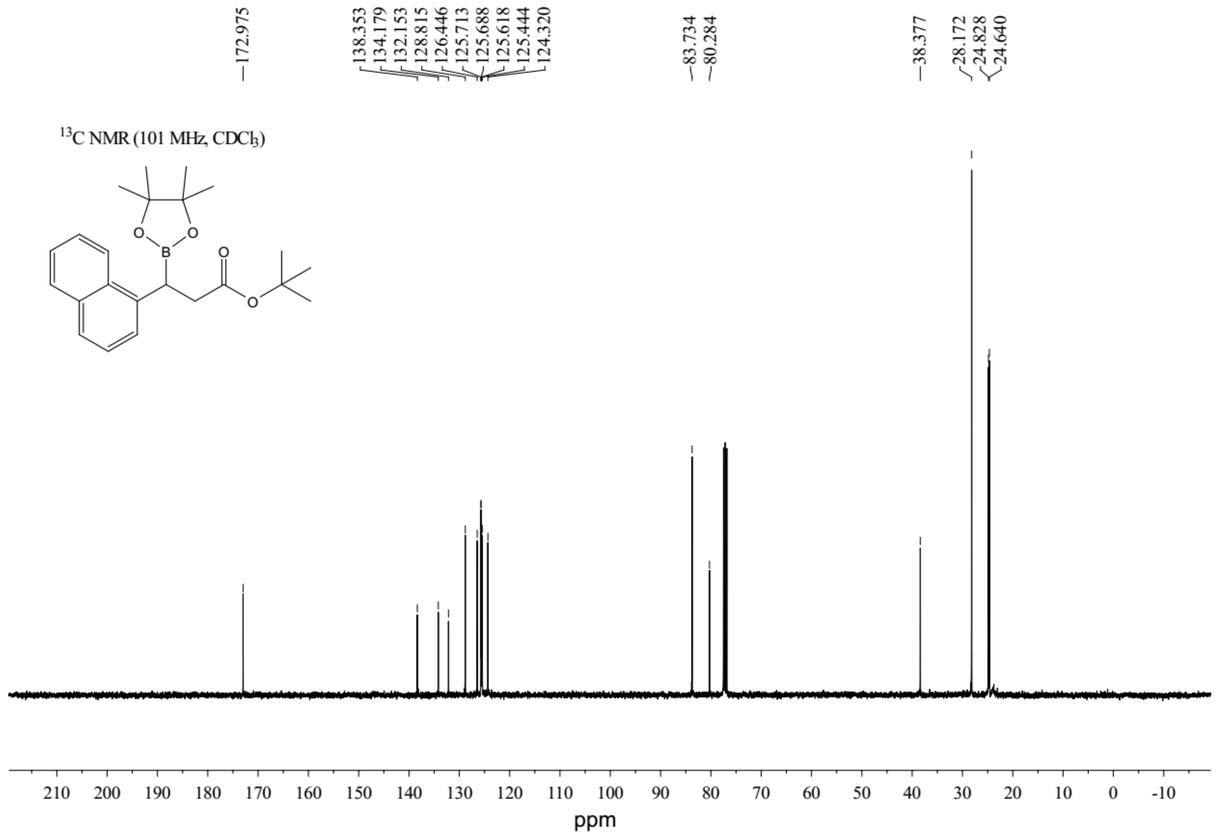

Supplementary Figure 39. ^{1}H NMR spectrum for Pentan-3-yl-3-(naphthalen-1-yl)-3-(4,4,5,5-tetramethyl-1,3,2-dioxaborolan-2-yl)propanoate (5g)

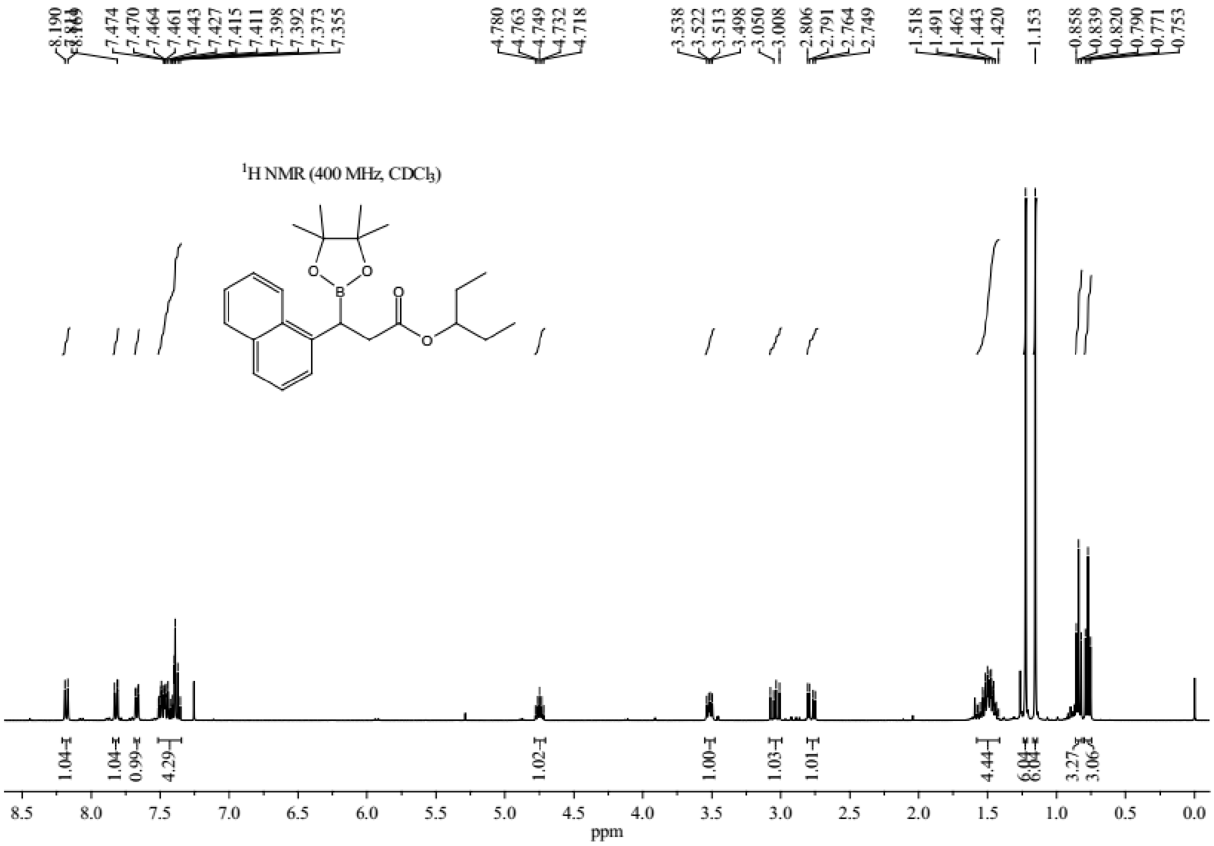

Supplementary Figure 40. ^{13}C NMR spectrum for 5g

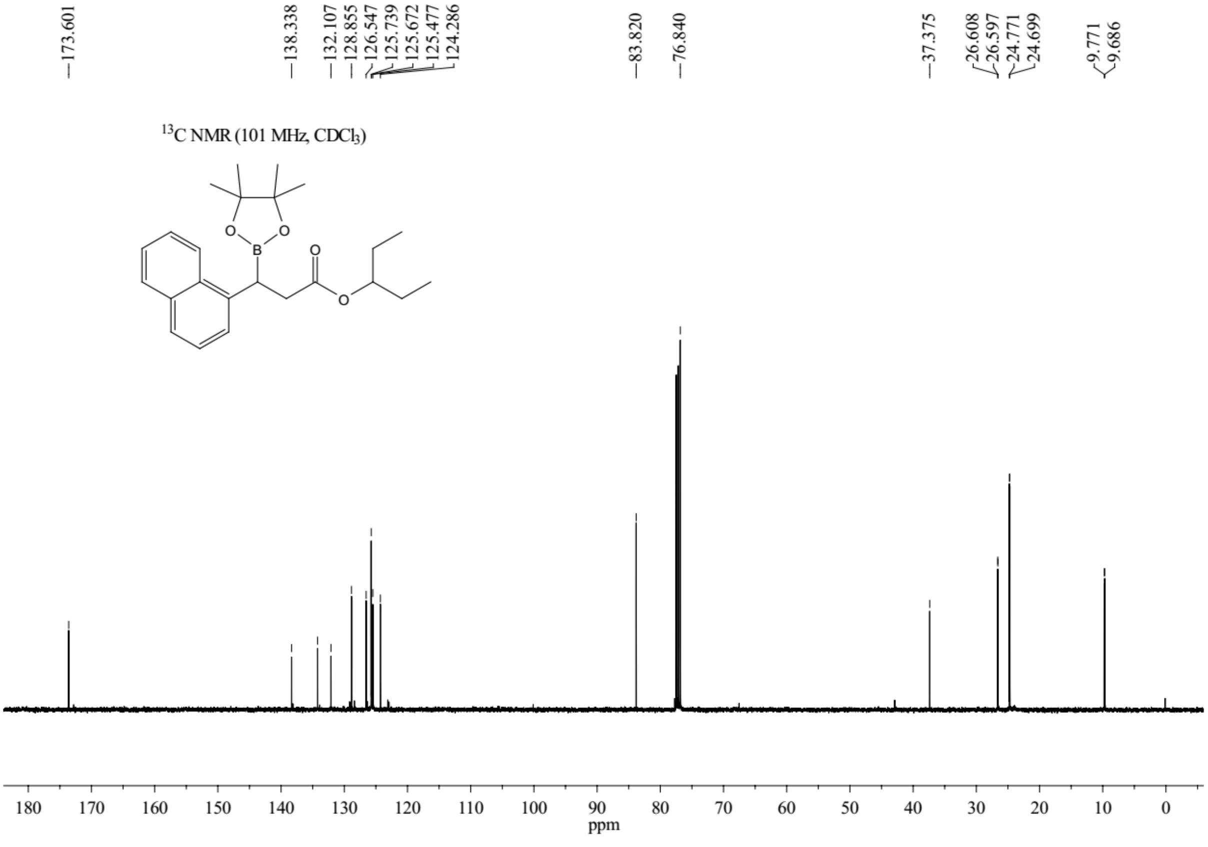

Supplementary Figure 41. ^{1}H NMR spectrum for *tert*-Butyl-3-(4,4,5,5-tetramethyl-1,3,2-dioxaborolan-2-yl)-3-(thiophen-2-yl)propanoate (5h′)

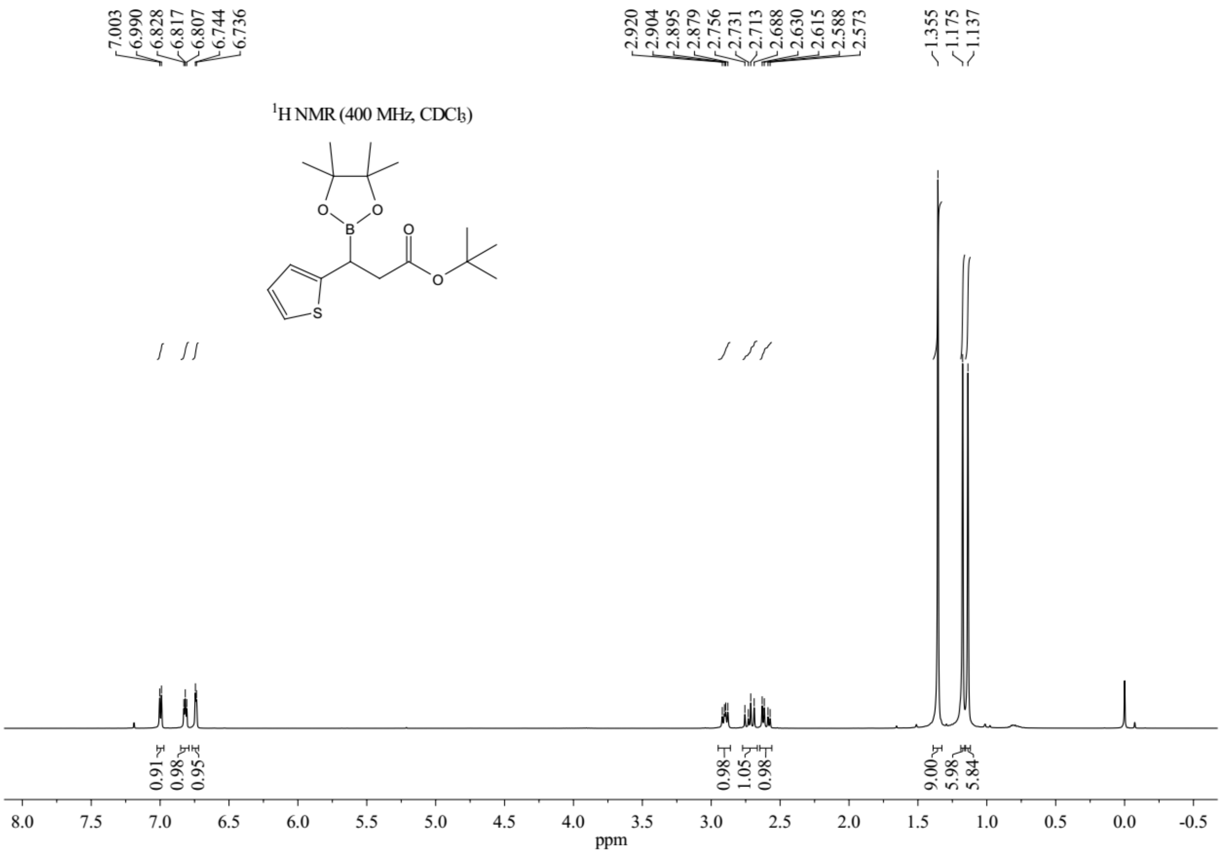

Supplementary Figure 42. ^{13}C NMR spectrum for 5h′

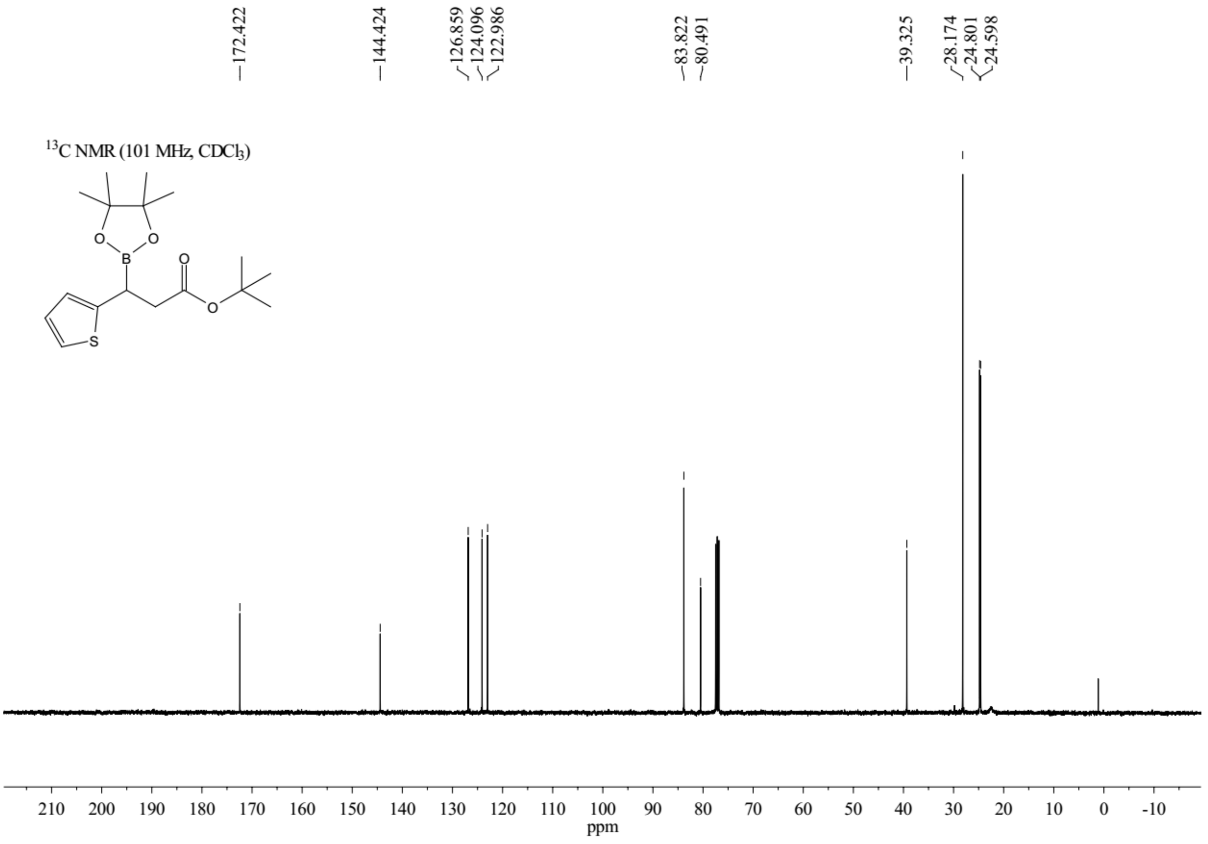

Supplementary Figure 43. ^{1}H NMR spectrum for Pentan-3-yl-3-(4,4,5,5-tetramethyl-1,3,2-dioxaborolan-2-yl)-3-(thiophen-2-yl)propanoate (5h)

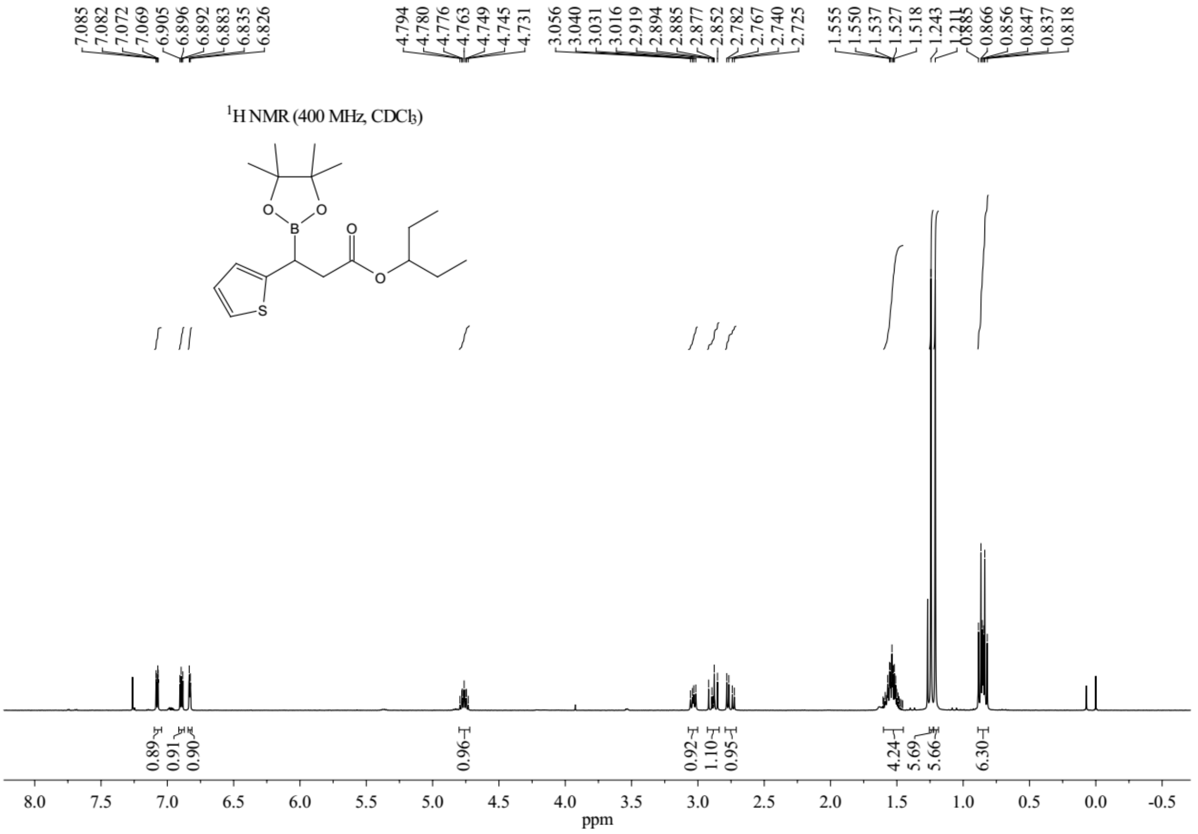

Supplementary Figure 44. ^{13}C NMR spectrum for 5h

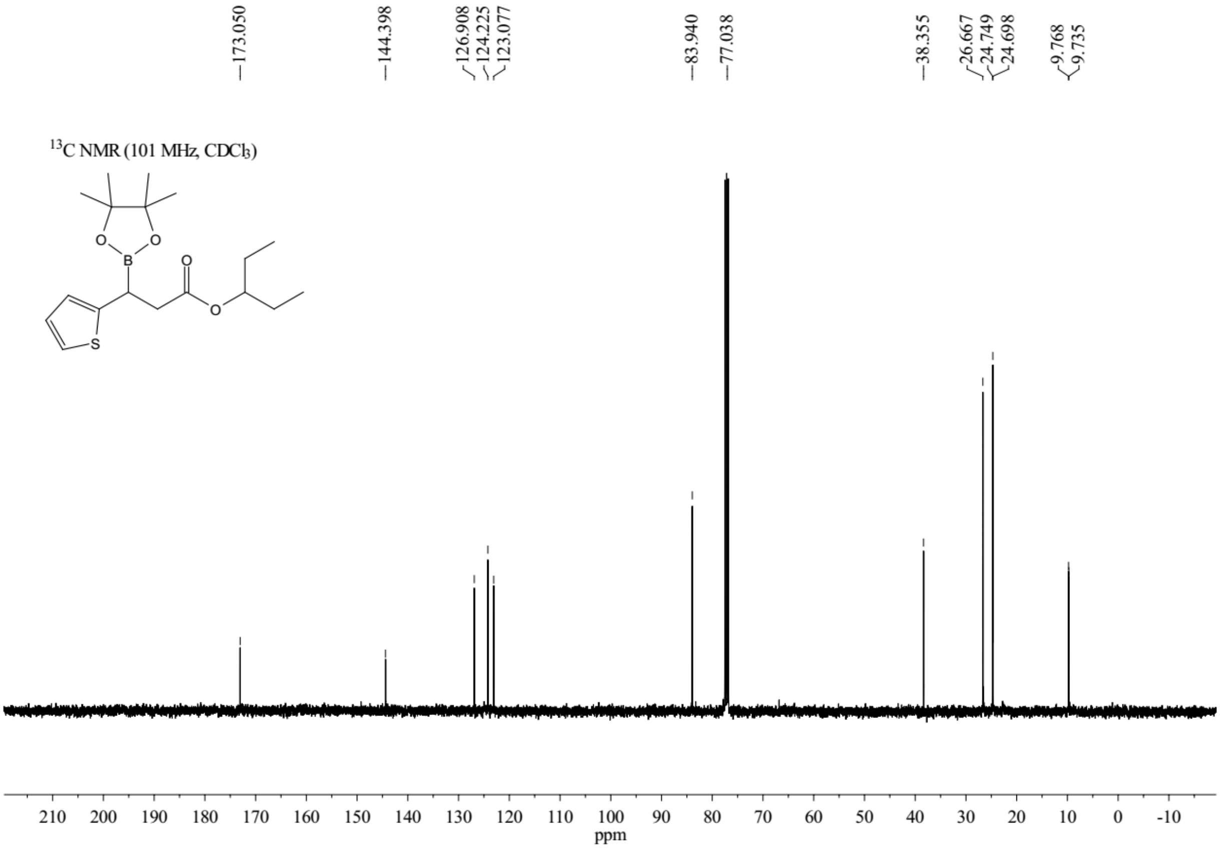

Supplementary Figure 45. ^{1}H NMR spectrum for *tert*-Butyl-3-(4,4,5,5-tetramethyl-1,3,2-dioxaborolan-2-yl)butanoate (5i′)

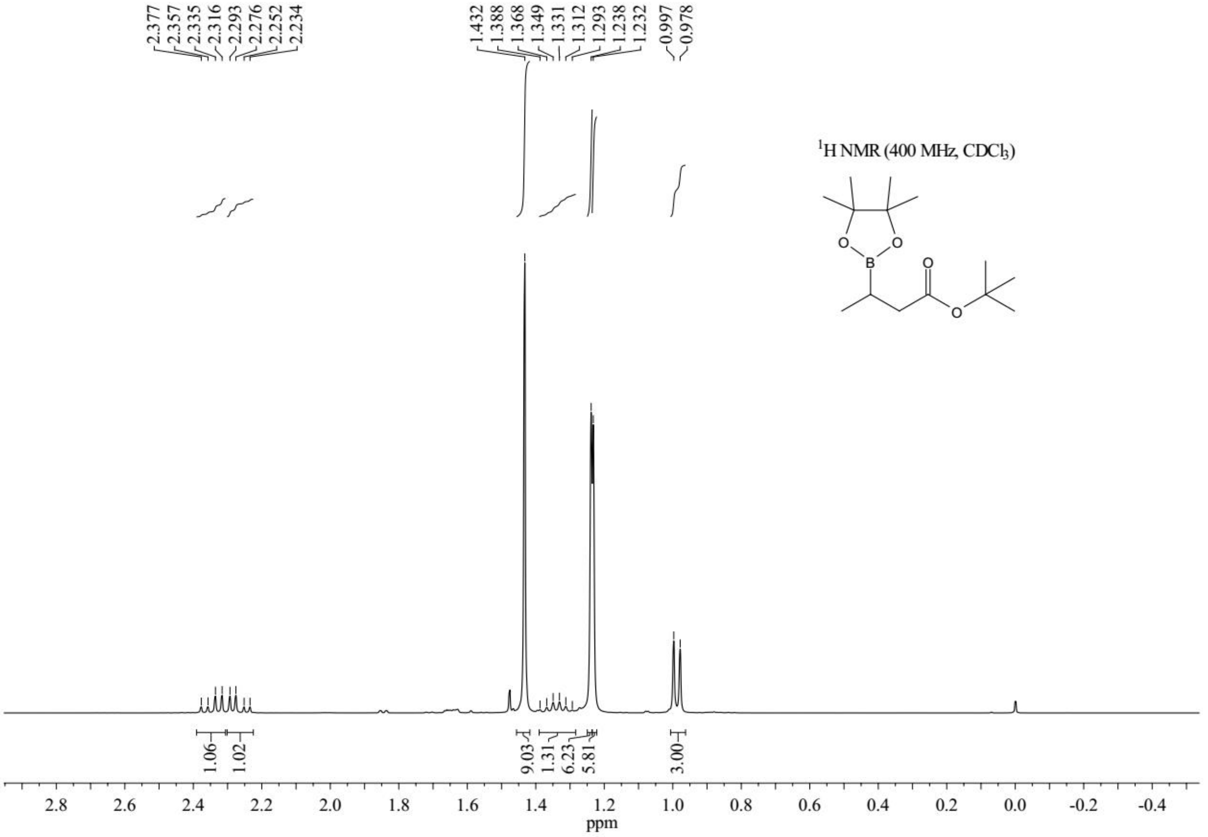

Supplementary Figure 46. ^{13}C NMR spectrum for 5i′

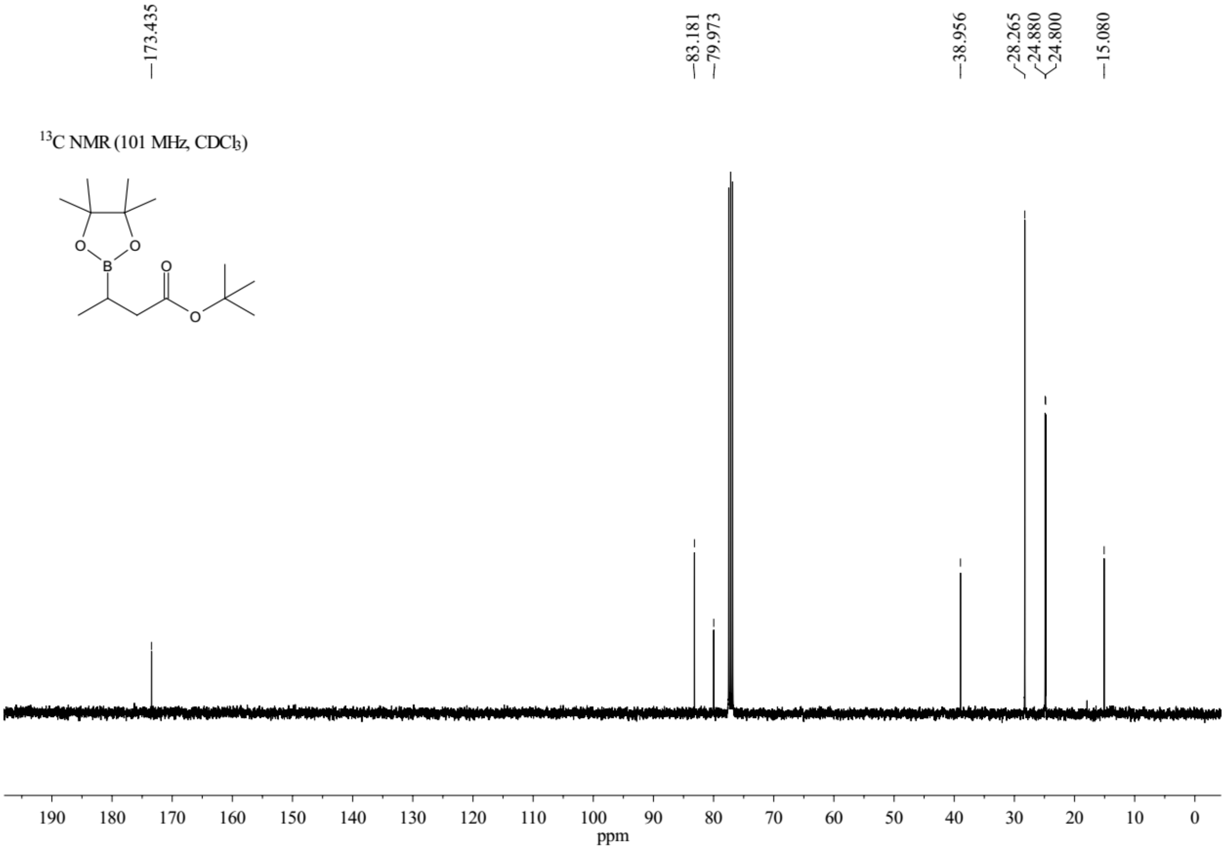

Supplementary Figure 47. ^{1}H NMR spectrum for Pentan-3-yl-3-(4,4,5,5-tetramethyl-1,3,2-dioxaborolan-2-yl)butanoate (5i)

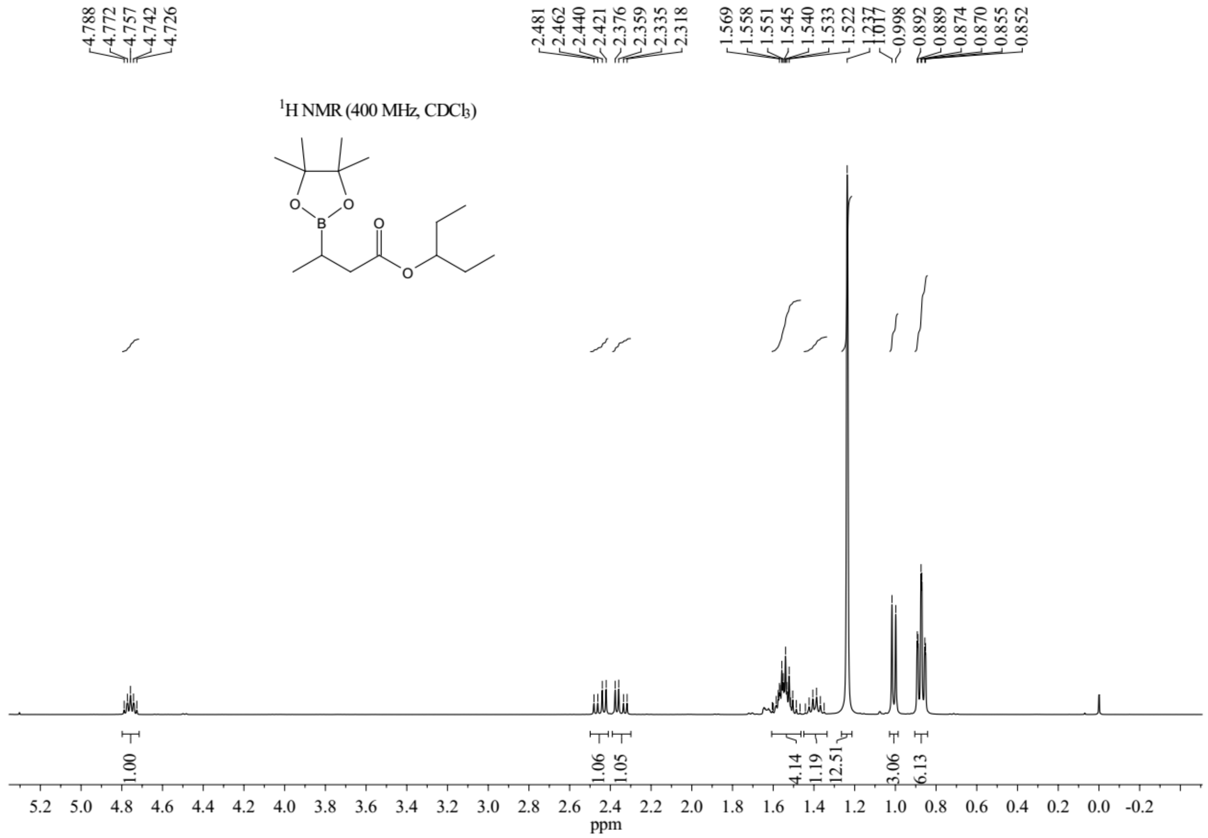

Supplementary Figure 48. ^{13}C NMR spectrum for 5i

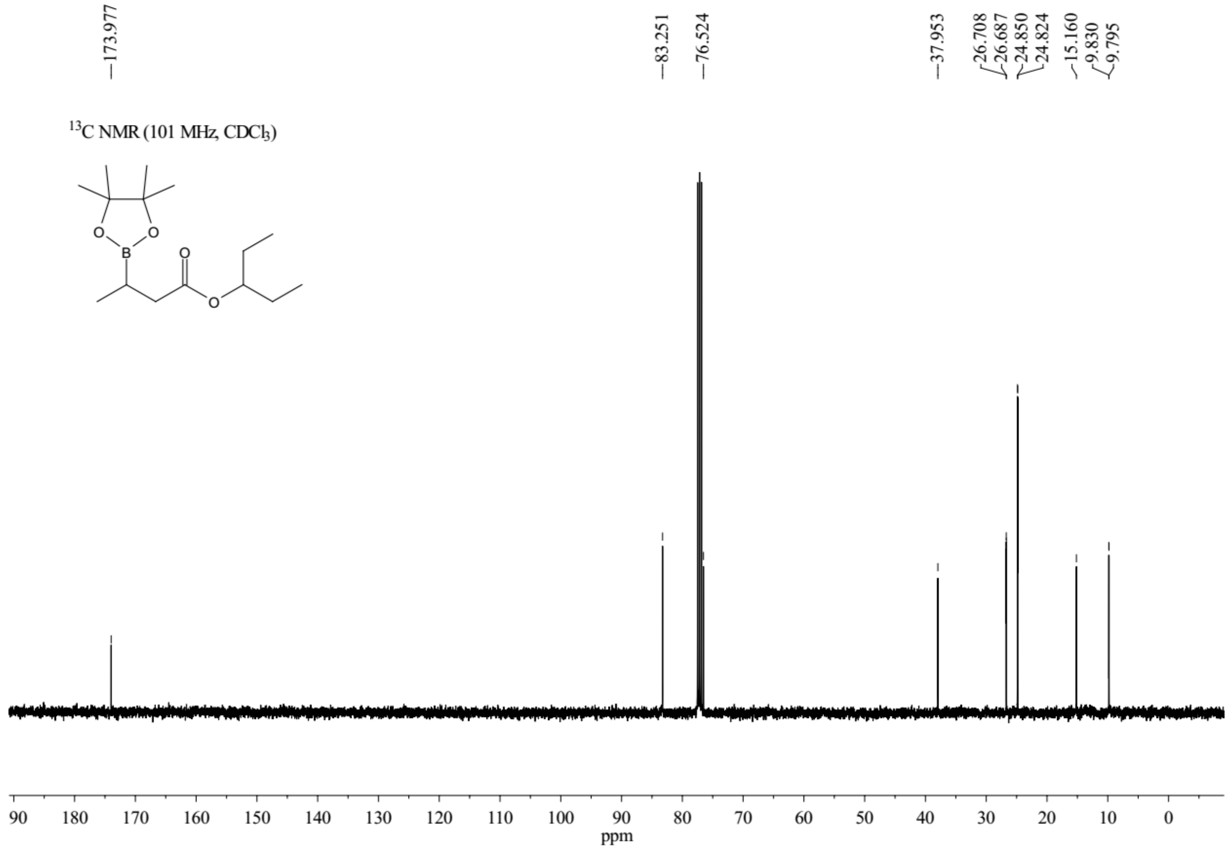

Supplementary Figure 49. ^{1}H NMR spectrum for *tert*-Butyl-3-(4,4,5,5-tetramethyl-1,3,2-dioxaborolan-2-yl)hexanoate (5j′)

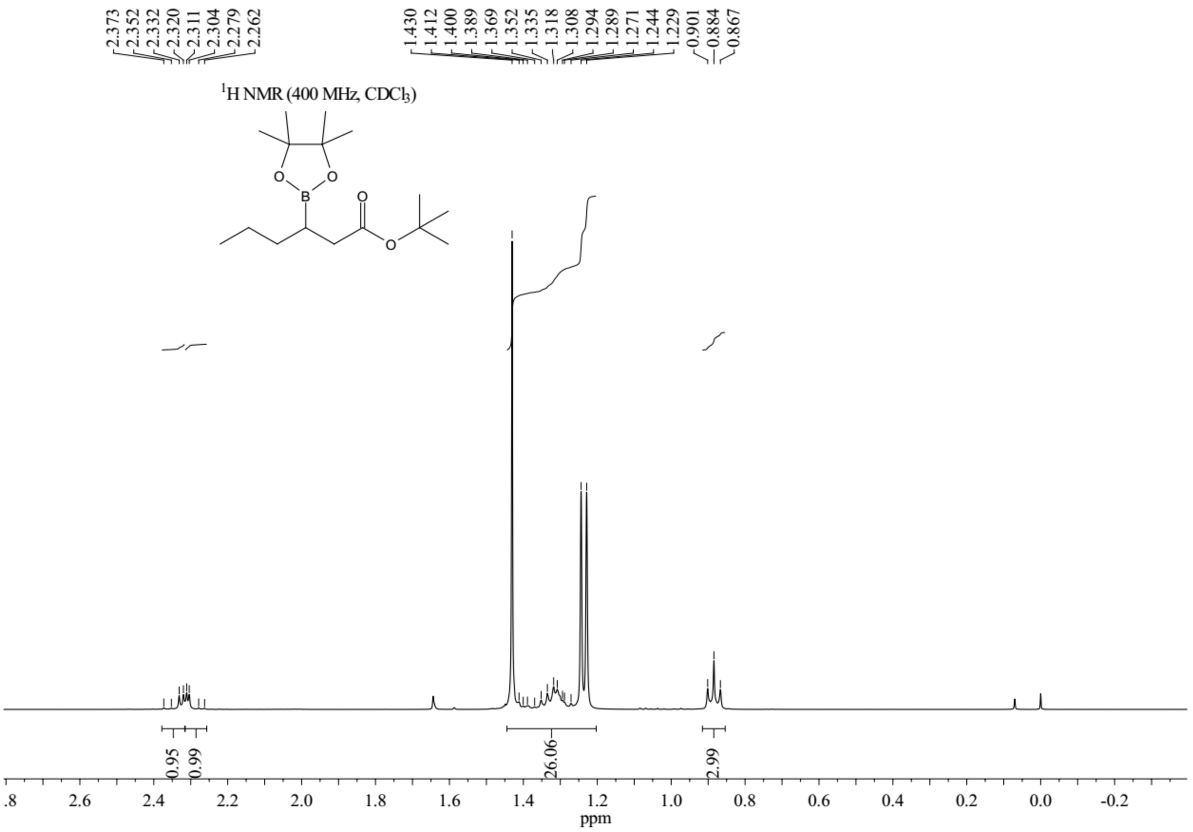

Supplementary Figure 50. ^{13}C NMR spectrum for 5j′

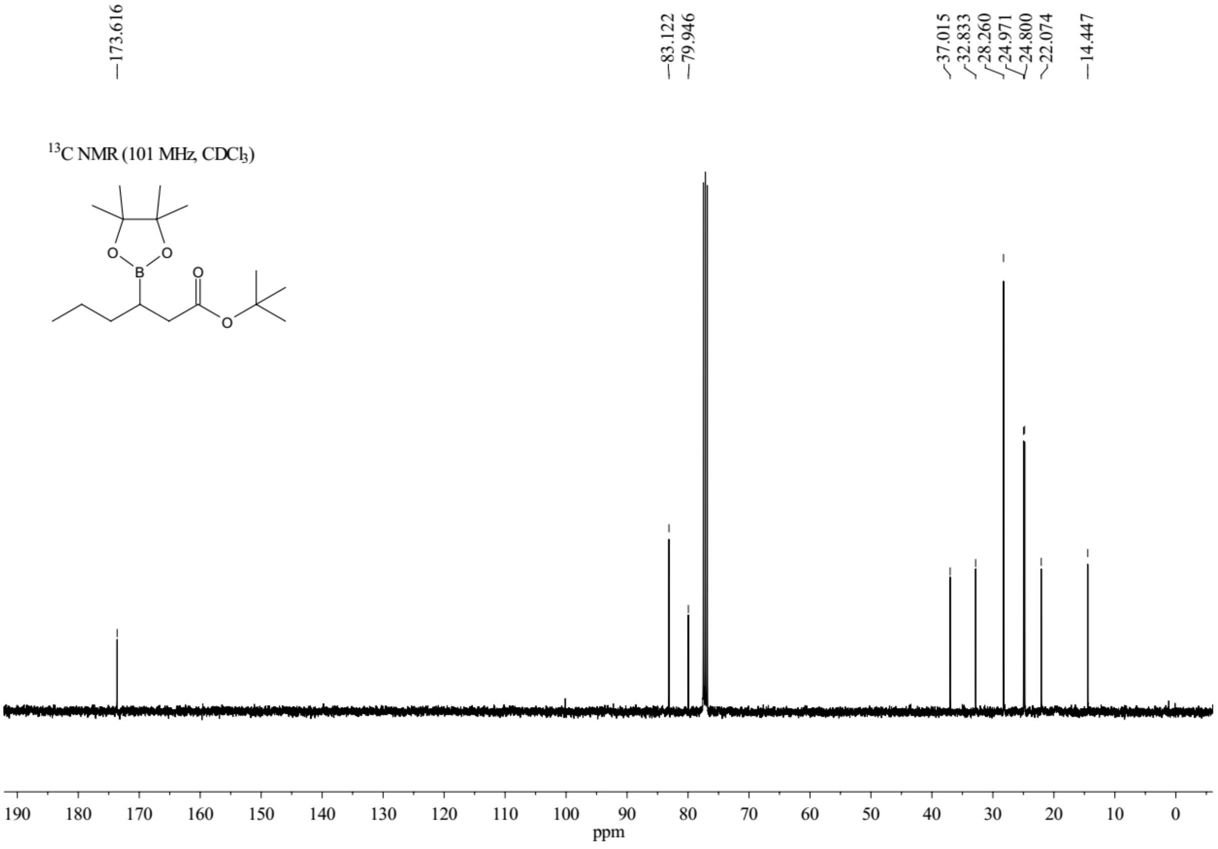

Supplementary Figure 51. ^{1}H NMR spectrum for Pentan-3-yl-3-(4,4,5,5-tetramethyl-1,3,2-dioxaborolan-2-yl)hexanoate (5j)

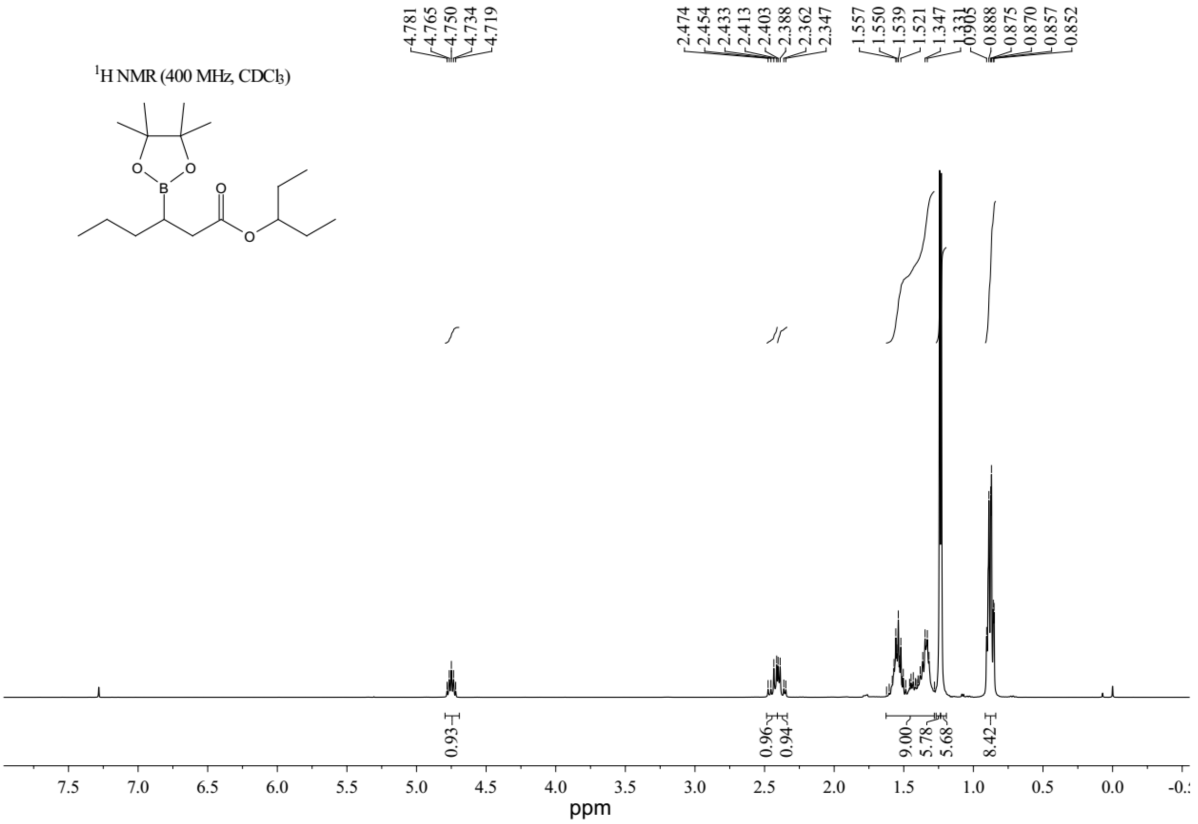

Supplementary Figure 52. ^{13}C NMR spectrum for 5j

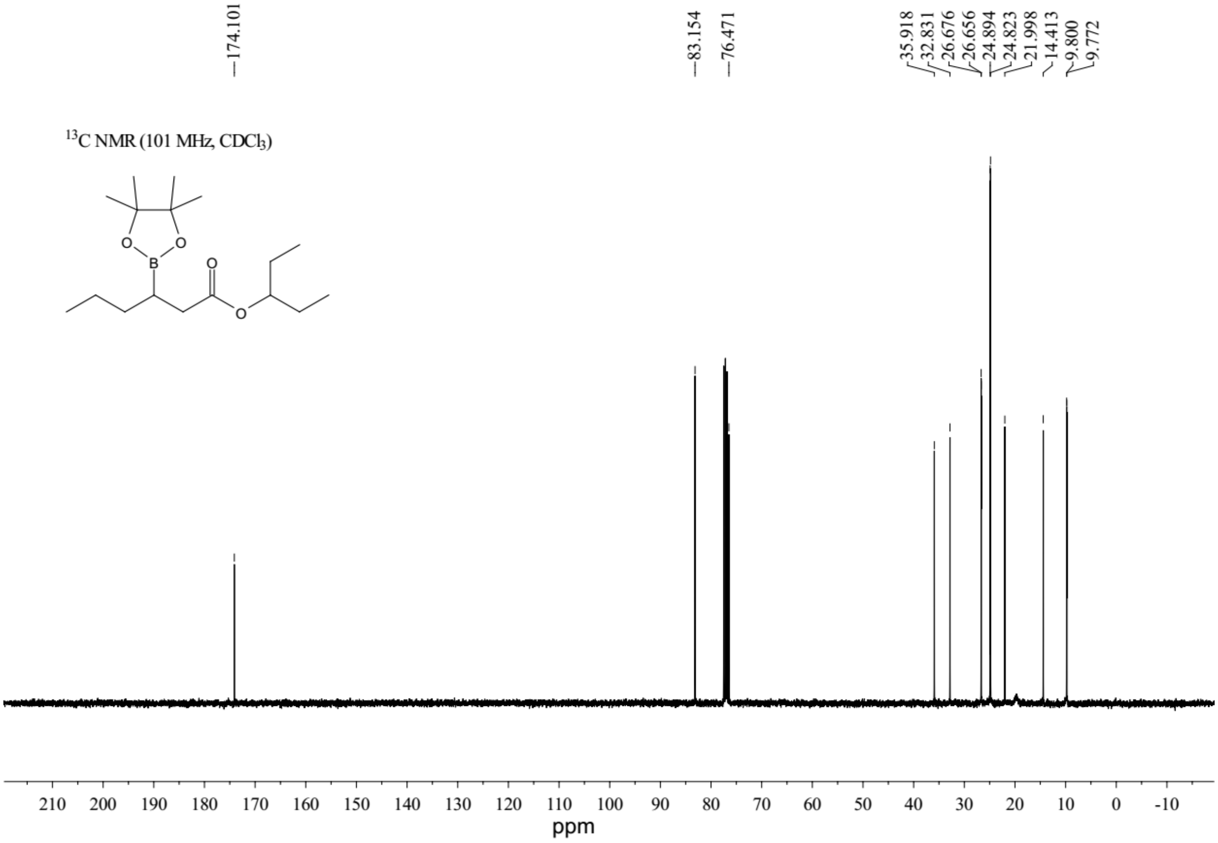

Supplementary Figure 53. ^{1}H NMR spectrum for *tert*-Butyl-5-phenyl-3-(4,4,5,5-tetramethyl-1,3,2-dioxaborolan-2-yl)pentanoate(5k′)

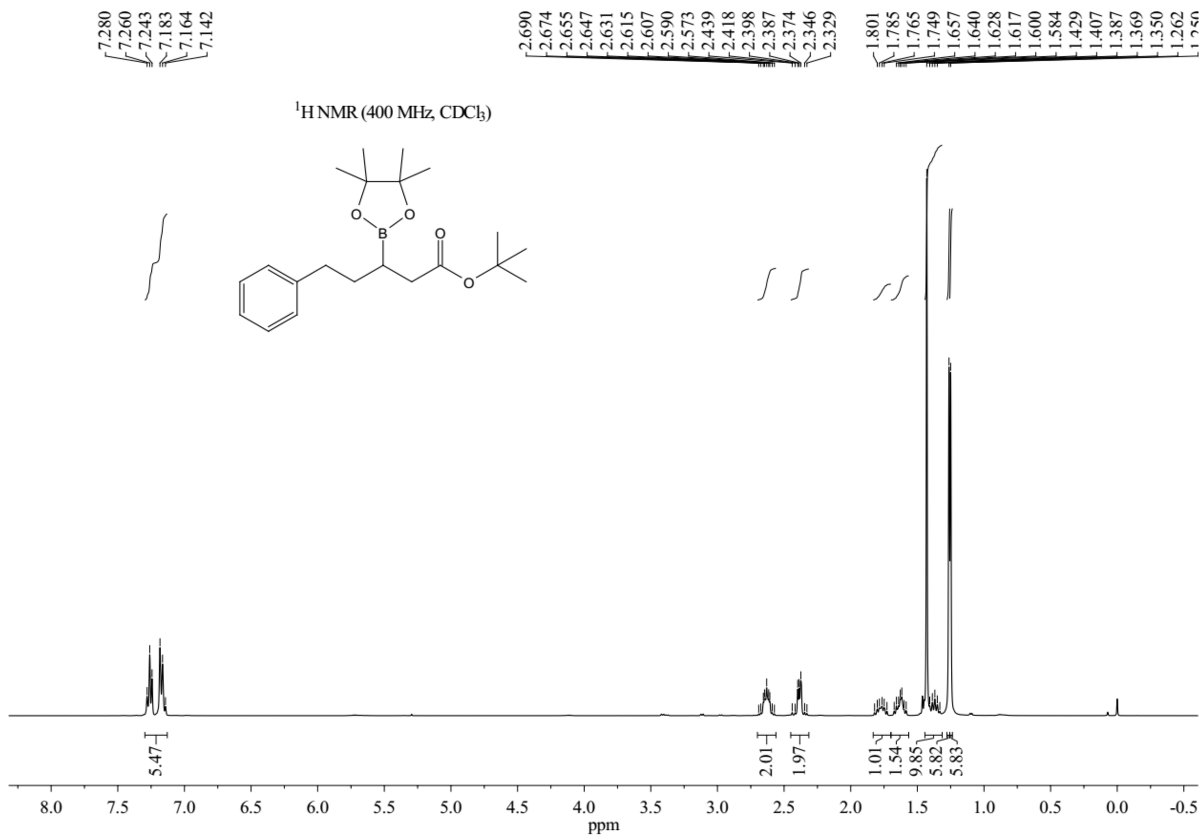

Supplementary Figure 54. ^{13}C NMR spectrum for 5k′

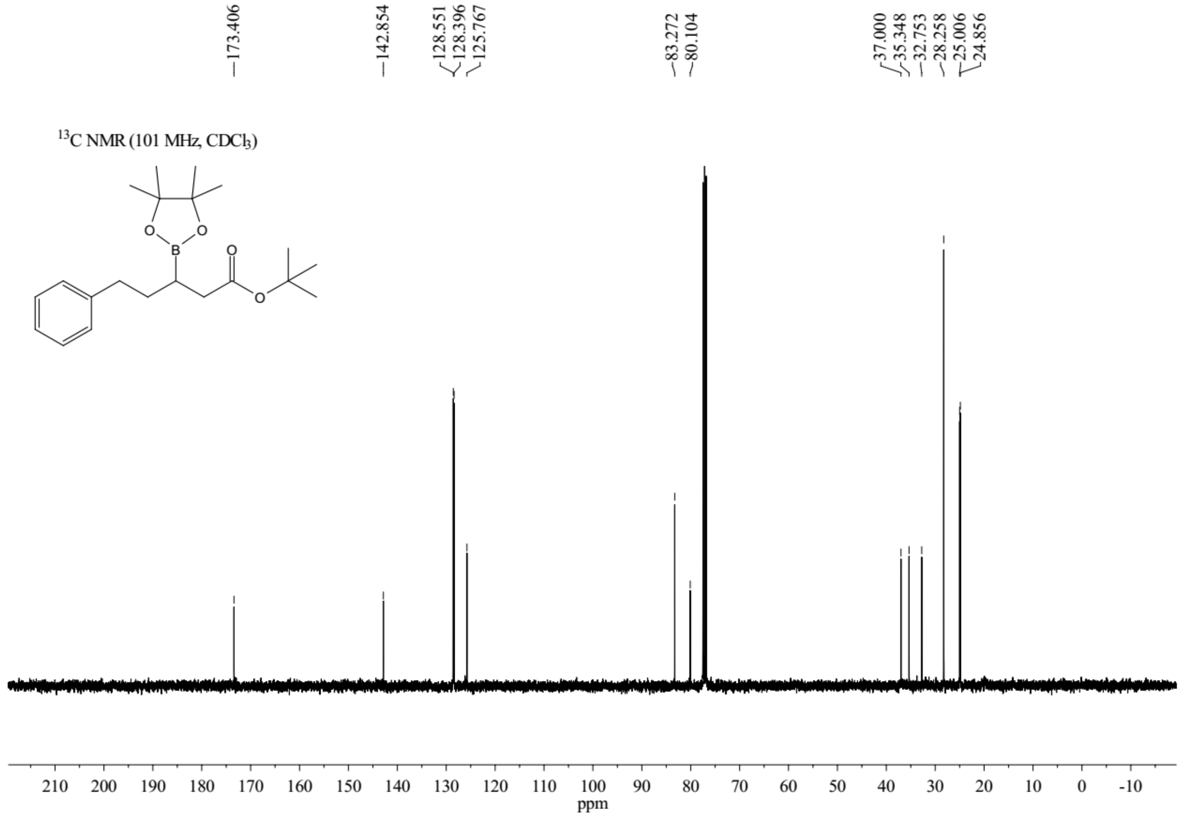

Supplementary Figure 55. ^{1}H NMR spectrum for Pentan-3-yl-5-phenyl-3-(4,4,5,5-tetramethyl-1,3,2-dioxaborolan-2-yl)pentanoate (5k)

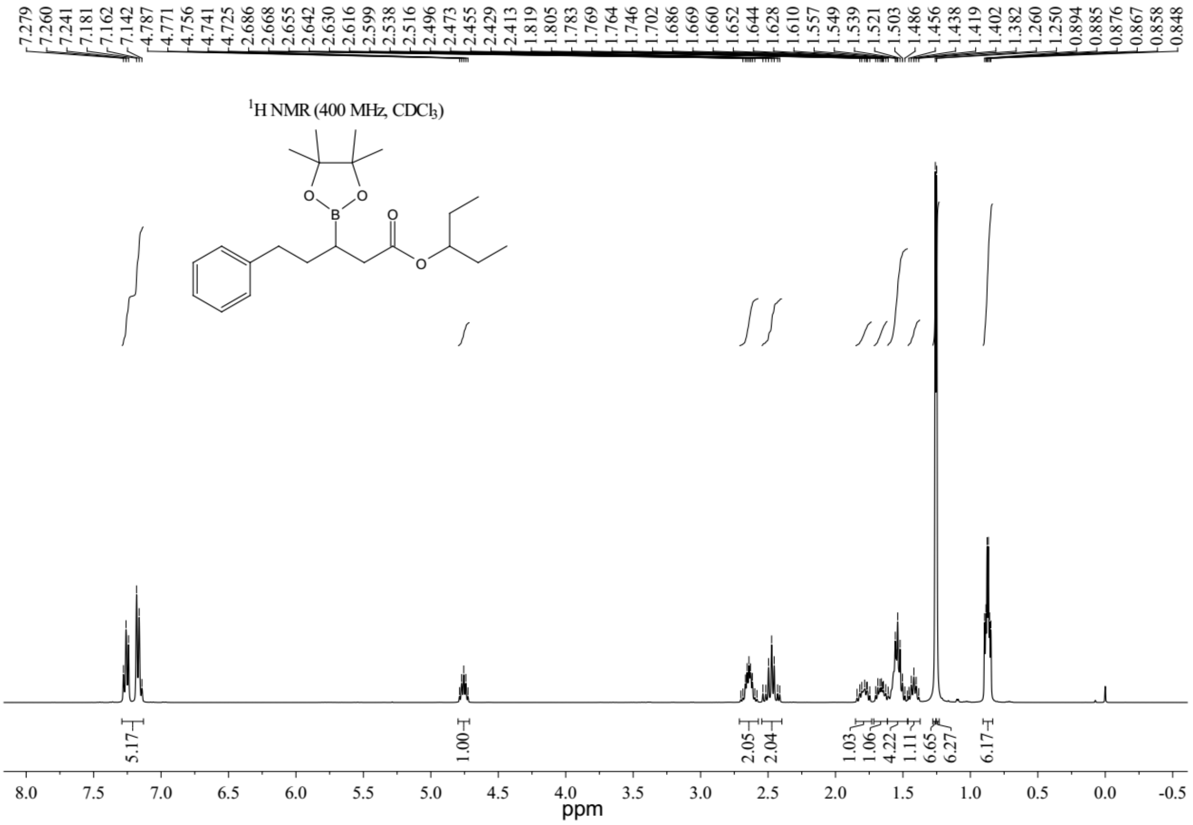

Supplementary Figure 56. ^{13}C NMR spectrum for 5k

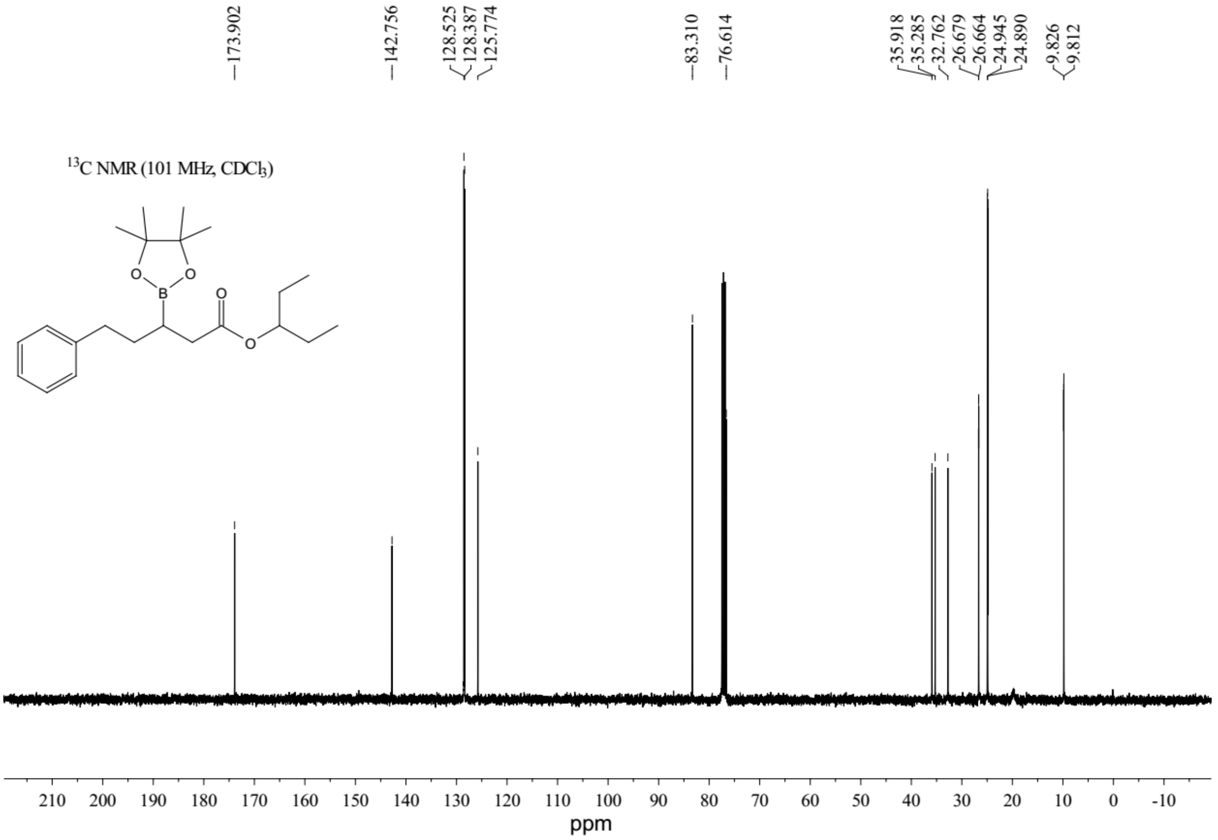

Supplementary Figure 57. ^{1}H NMR spectrum for *tert*-Butyl-4-methyl-3-(4,4,5,5-tetramethyl-1,3,2-dioxaborolan-2-yl)pentanoate (5l′)

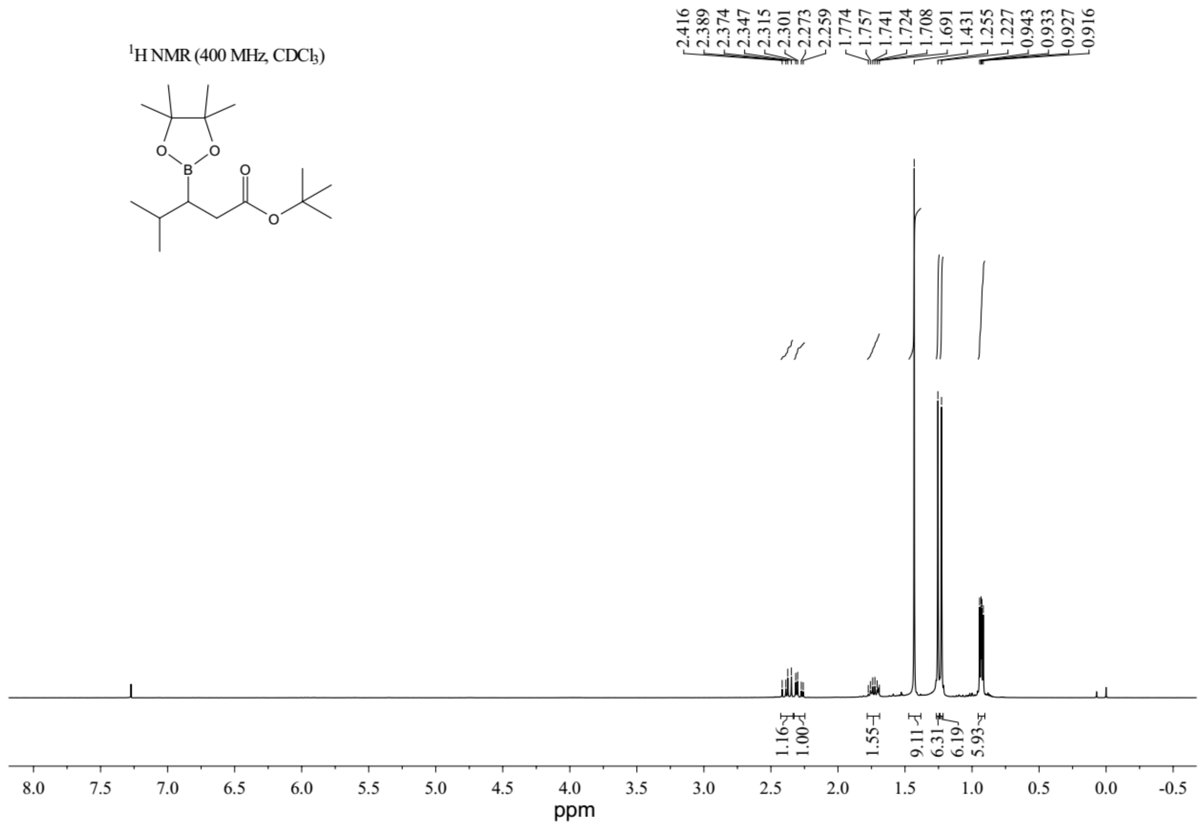

Supplementary Figure 58. ^{13}C NMR spectrum for 5l′

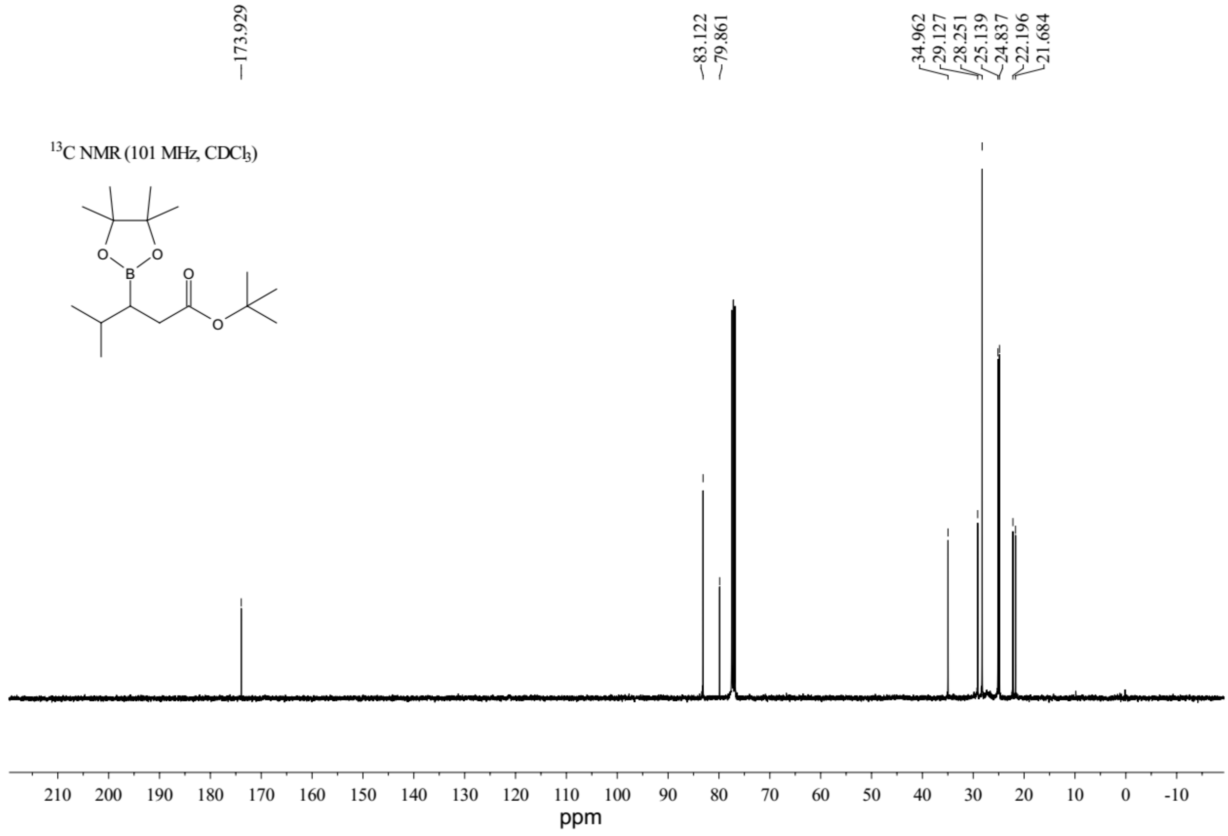

Supplementary Figure 59. ^{1}H NMR spectrum for Pentan-3-yl-4-methyl-3-(4,4,5,5-tetramethyl-1,3,2-dioxaborolan-2-yl)pentanoate (5l)

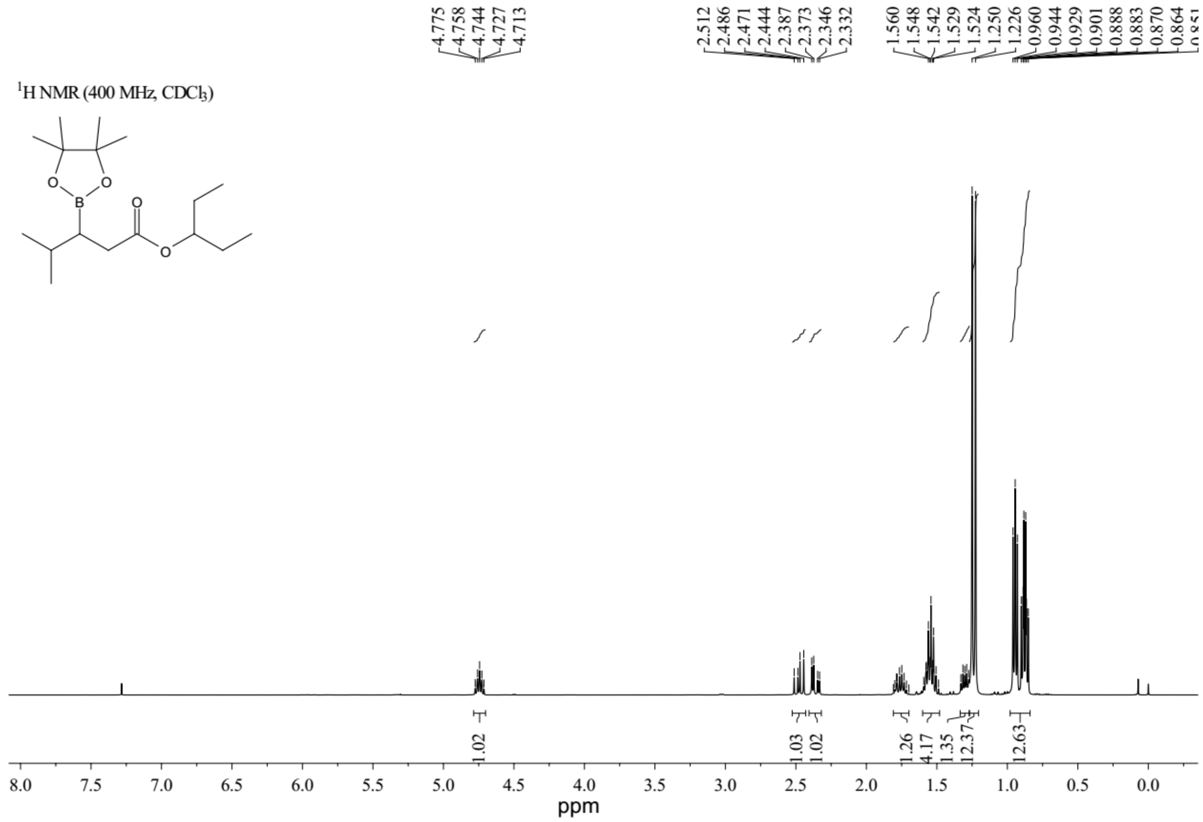

Supplementary Figure 60. ^{13}C NMR spectrum for 5l

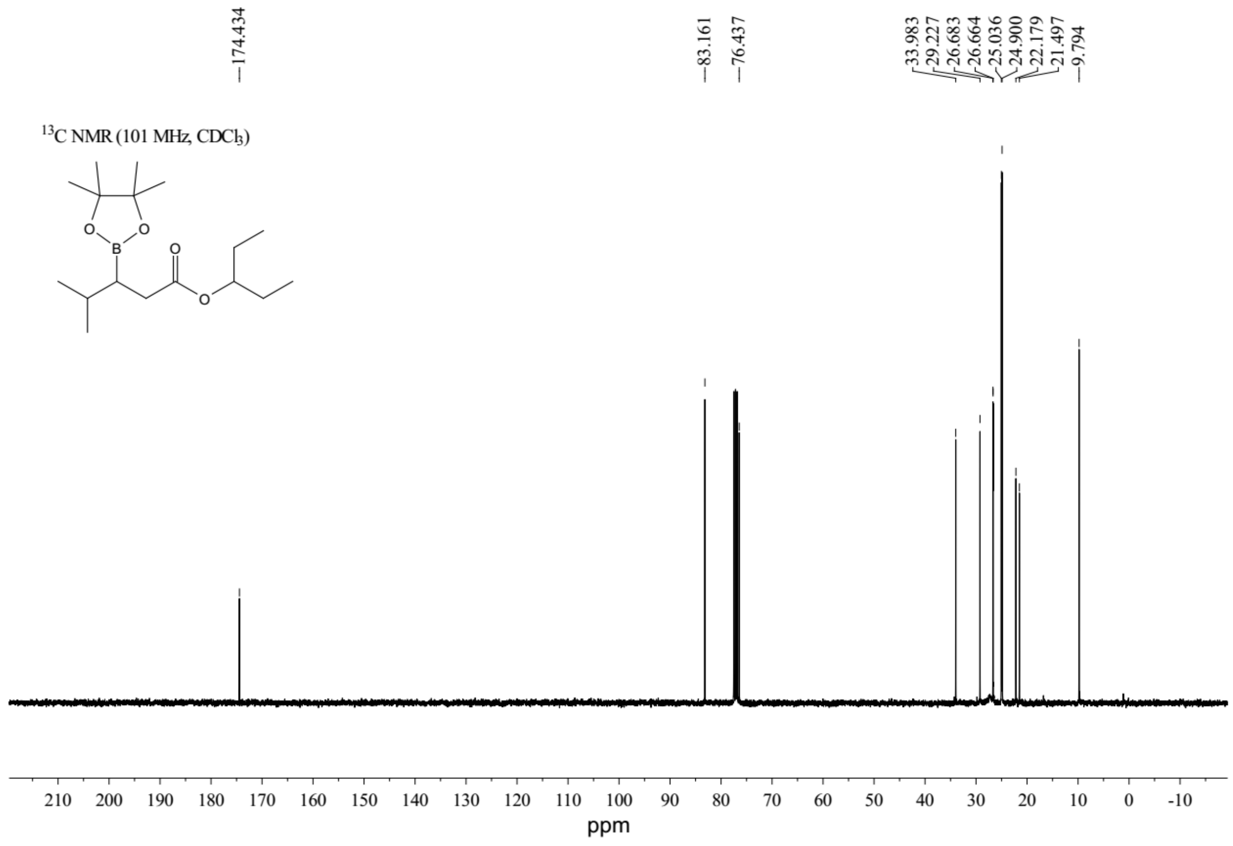

Supplementary Figure 61. ¹H NMR spectrum for *tert*-Butyl-3-cyclohexyl-3-(4,4,5,5-tetramethyl-1,3,2-dioxaborolan-2-yl)propanoate (5m′)

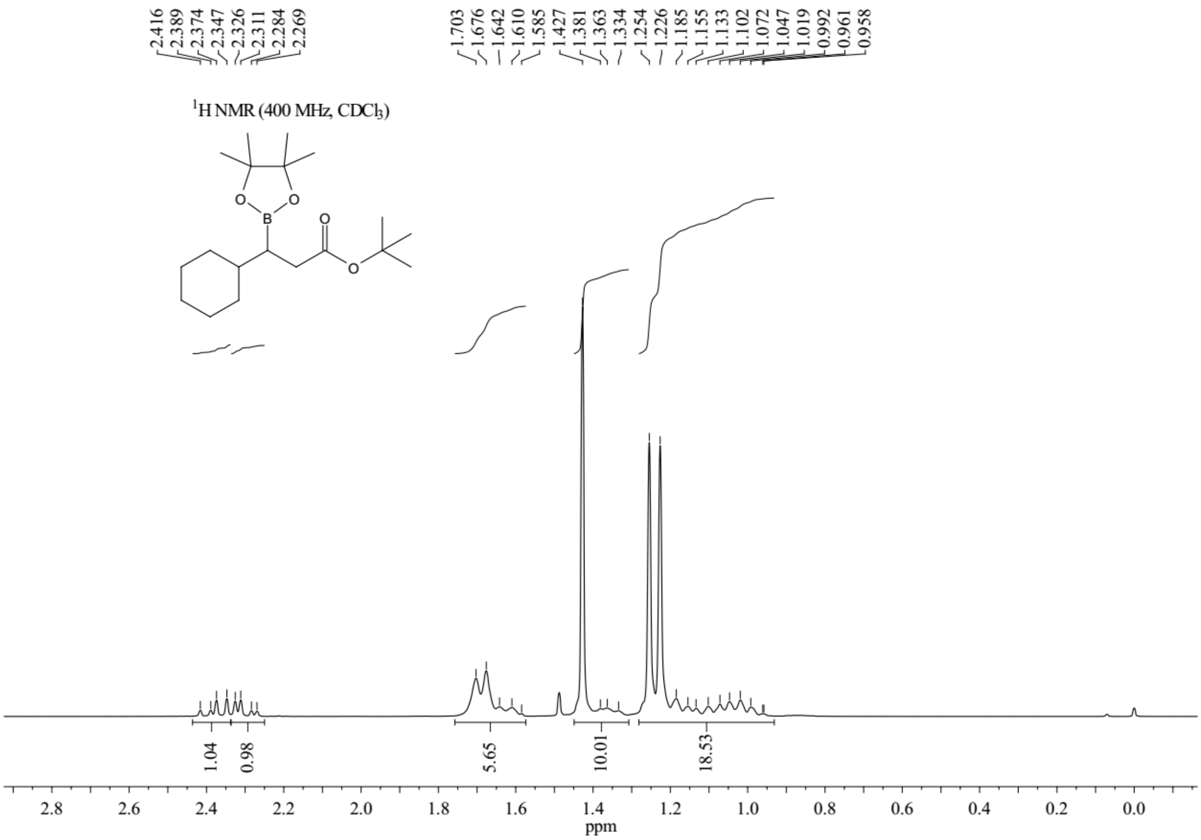

Supplementary Figure 62. ¹³C NMR spectrum for 5m′

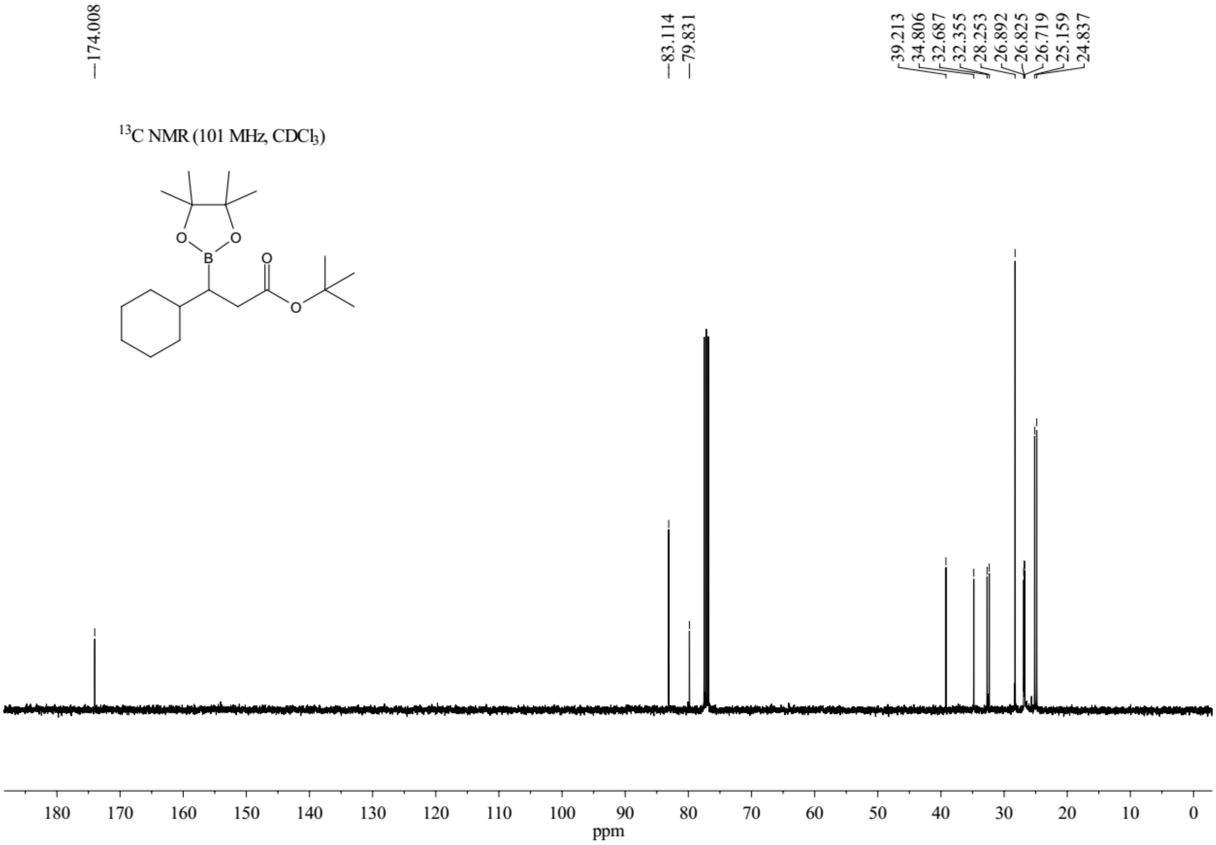

Supplementary Figure 63. ^{1}H NMR spectrum for Pentan-3-yl-3-cyclohexyl-3-(4,4,5,5-tetramethyl-1,3,2-dioxaborolan-2-yl)propanoate (5m)

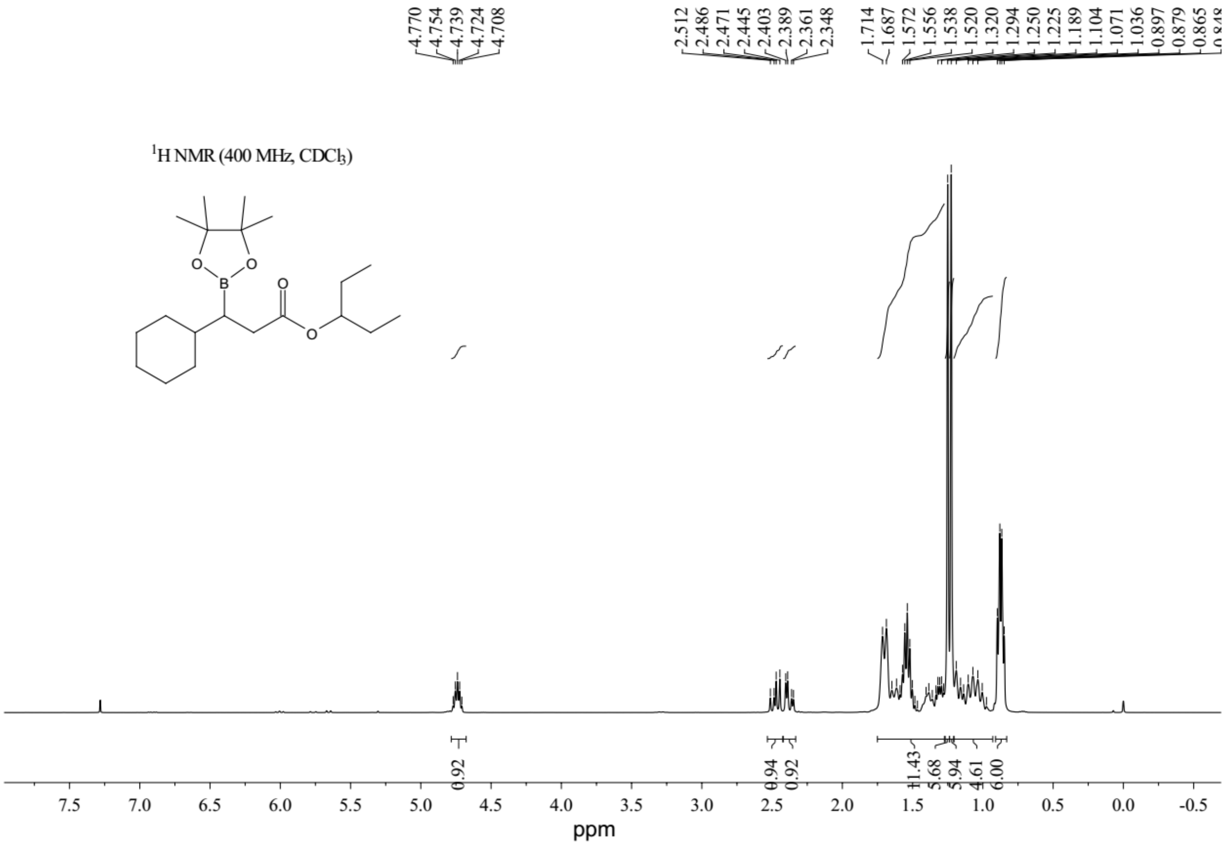

Supplementary Figure 64. ^{13}C NMR spectrum for 5m

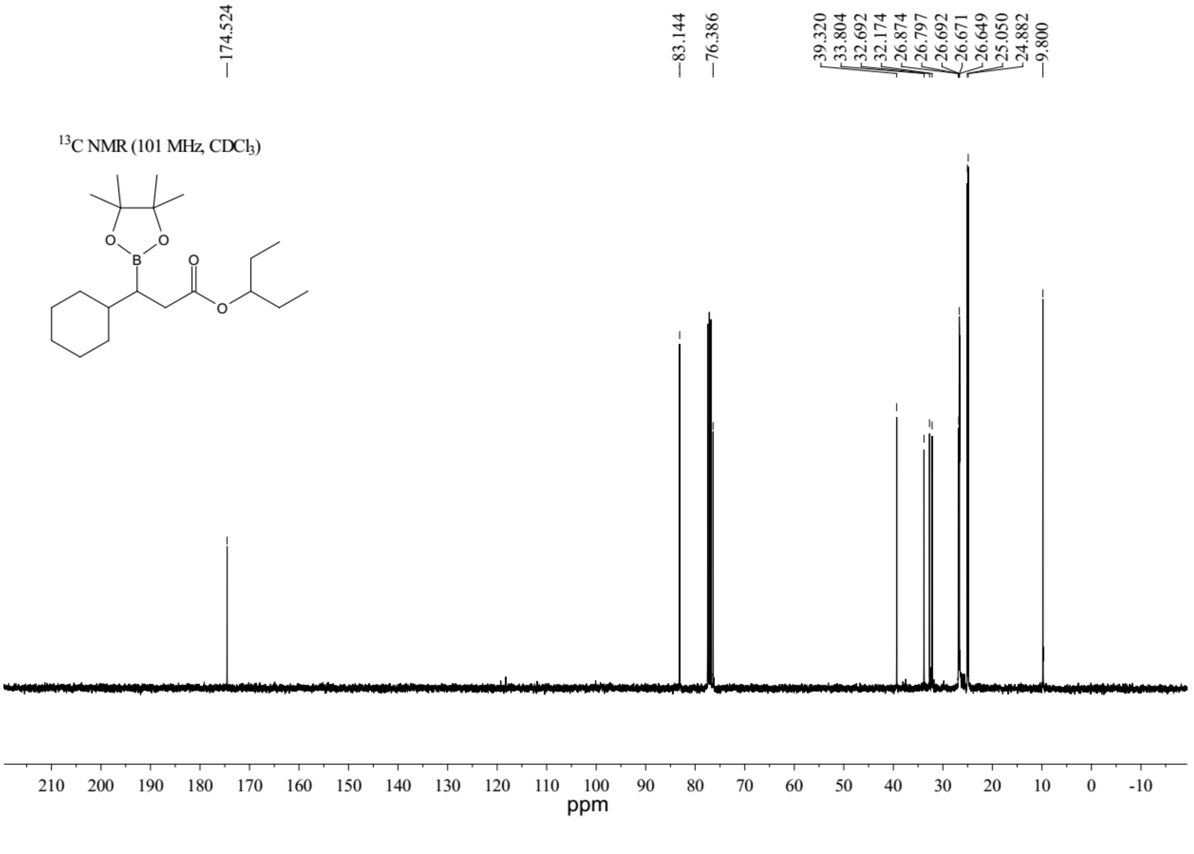

Supplementary Figure 65. ^{1}H NMR spectrum for *tert*-Butyl-3-cyclopropyl-3-(4,4,5,5-tetramethyl-1,3,2-dioxaborolan-2-yl)propanoate (5n′)

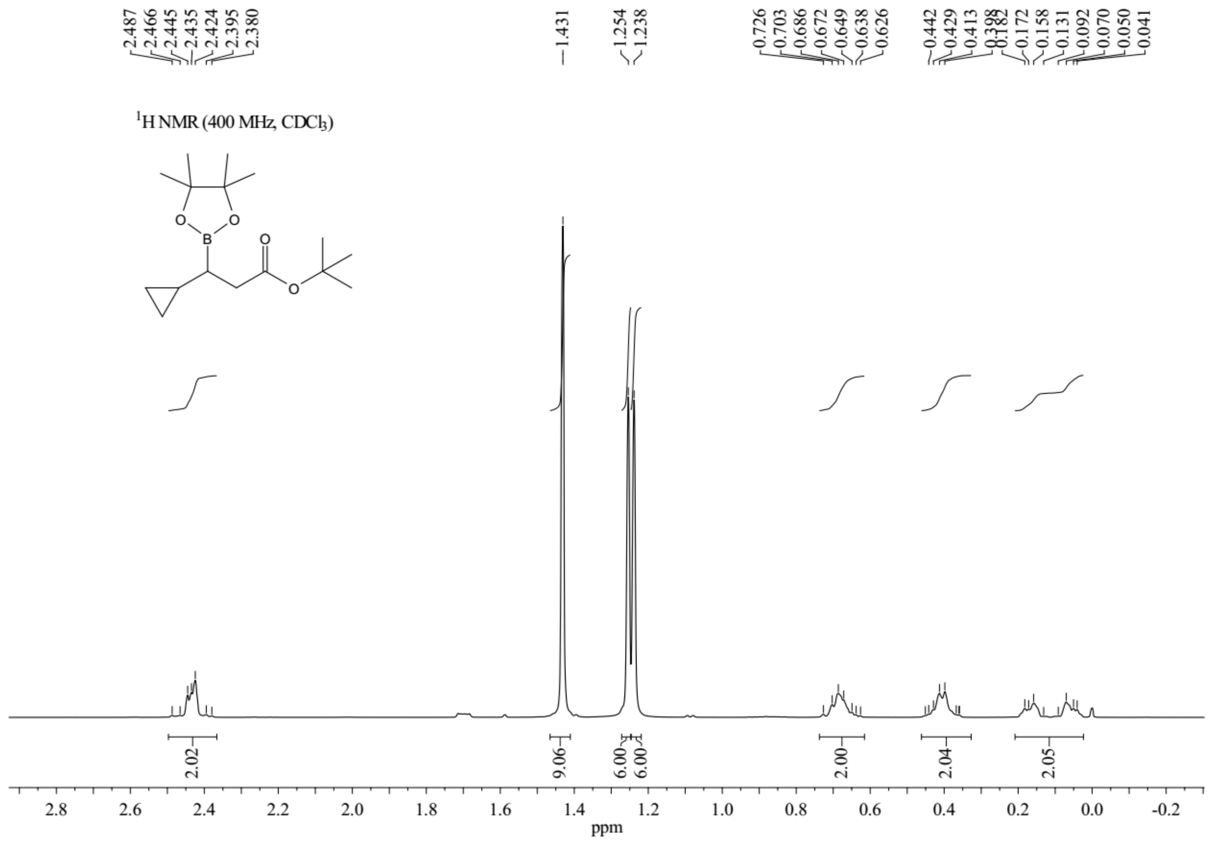

Supplementary Figure 66. ^{13}C NMR spectrum for 5n′

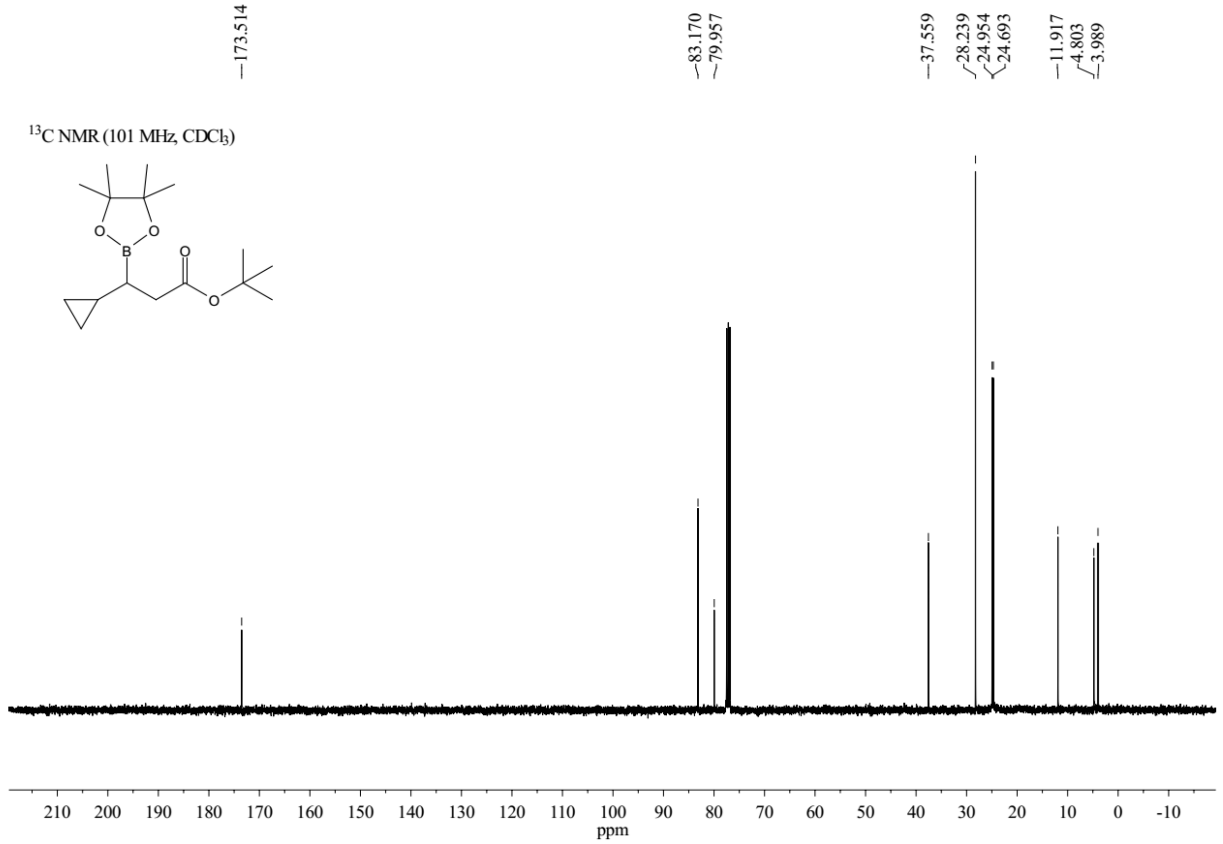

Supplementary Figure 67. ^{1}H NMR spectrum for Pentan-3-yl-3-cyclopropyl-3-(4,4,5,5-tetramethyl-1,3,2-dioxaborolan-2-yl)propanoate (5n)

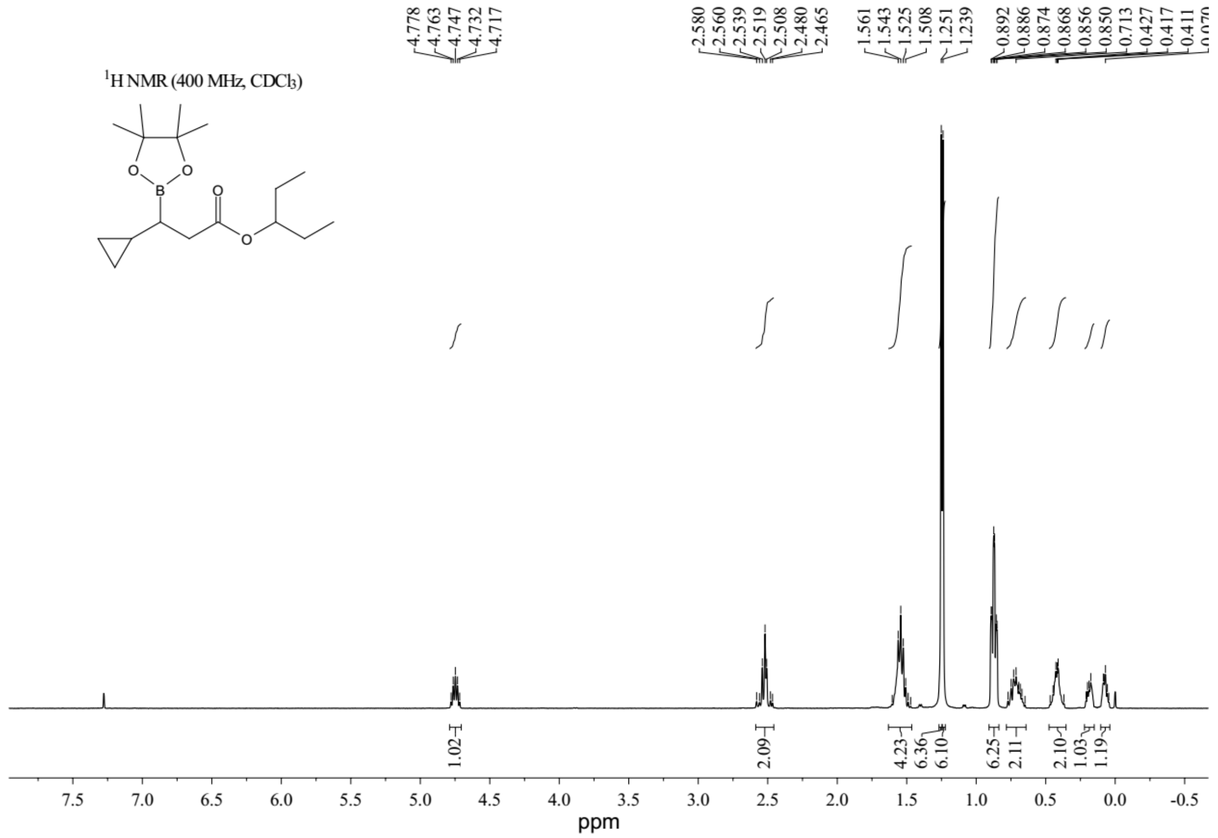

Supplementary Figure 68. ^{13}C NMR spectrum for 5n

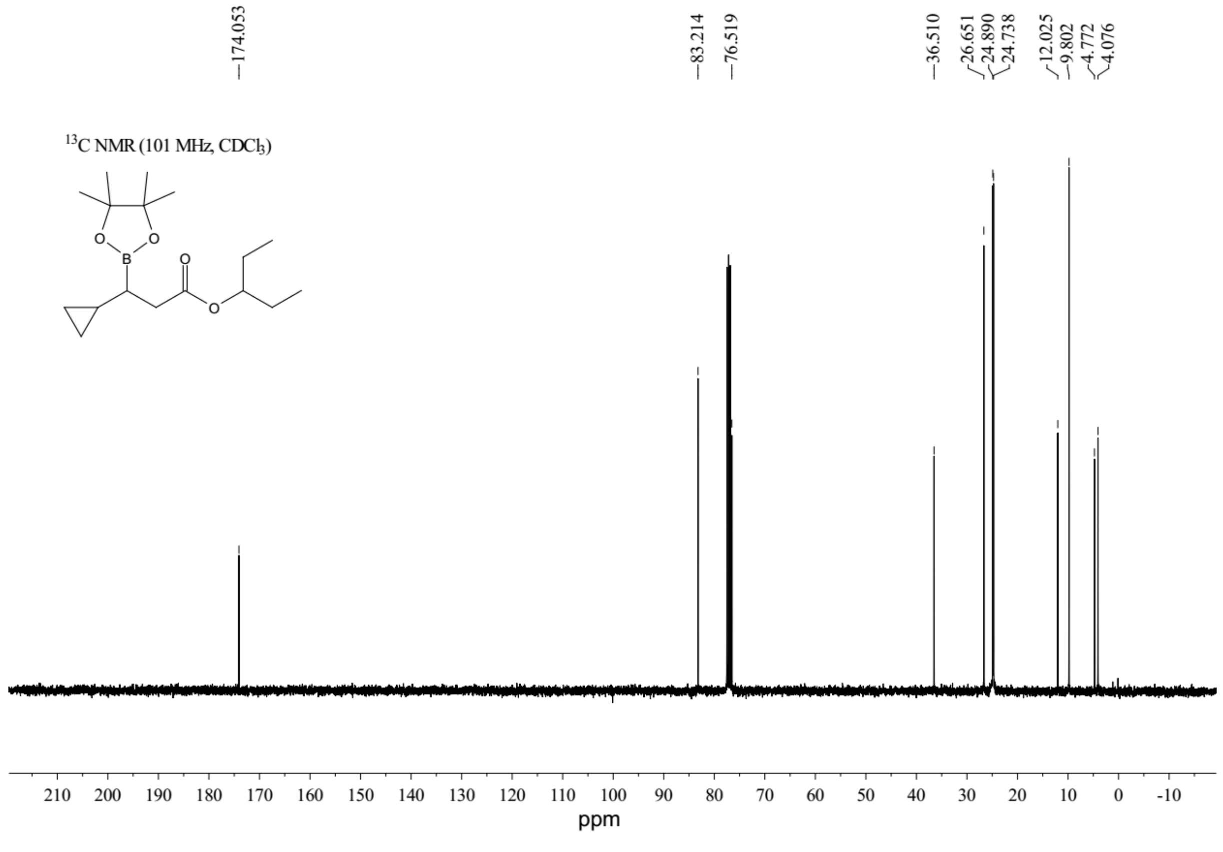

Supplementary Figure 69. ^{1}H NMR spectrum for Pentan-3-yl (*R*,*E*)-3-(4,4,5,5-tetramethyl-1,3,2-dioxaborolan-2-yl)hex-4-enoate (5o)

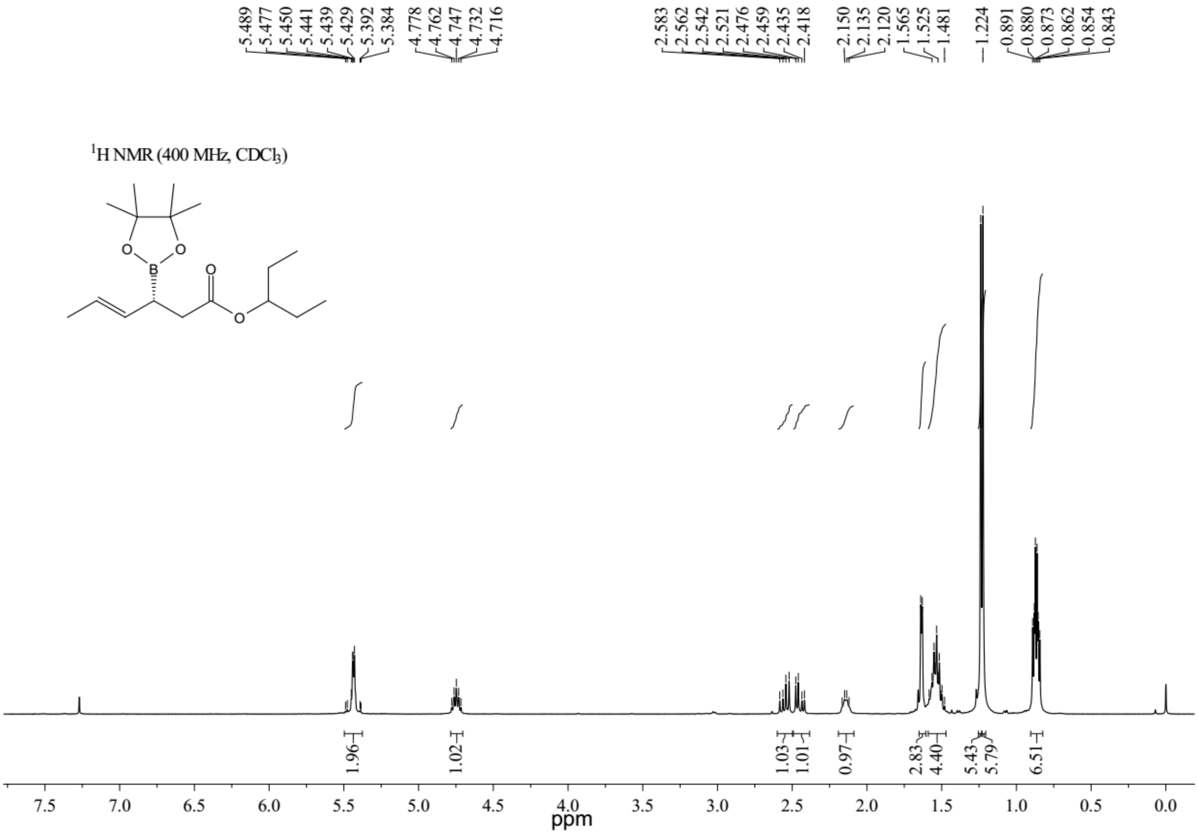

Supplementary Figure 70. ^{13}C NMR spectrum for 5o

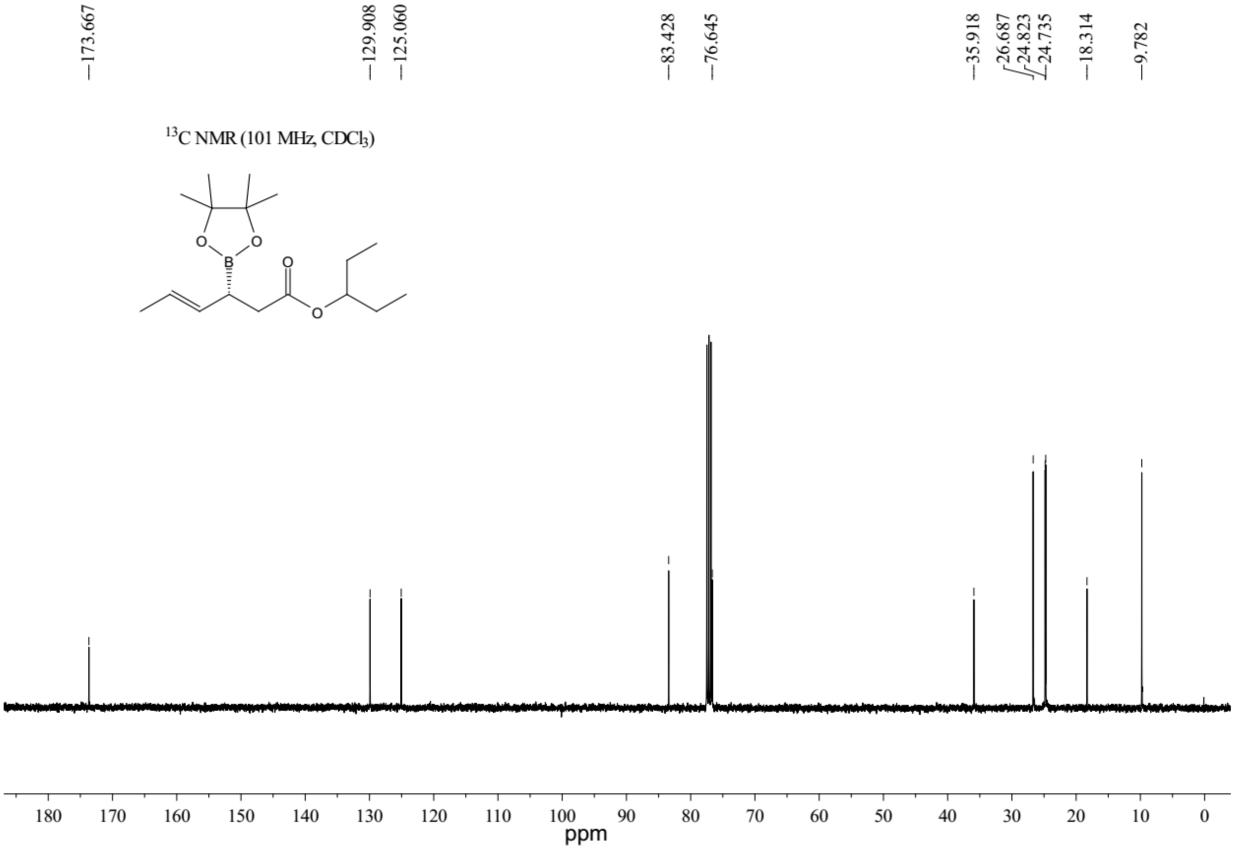

Supplementary Figure 71. ^{1}H NMR spectrum for Pentan-3-yl-5-methyl-3-(4,4,5,5-tetramethyl-1,3,2-dioxaborolan-2-yl)hex-4-enoate (5p)

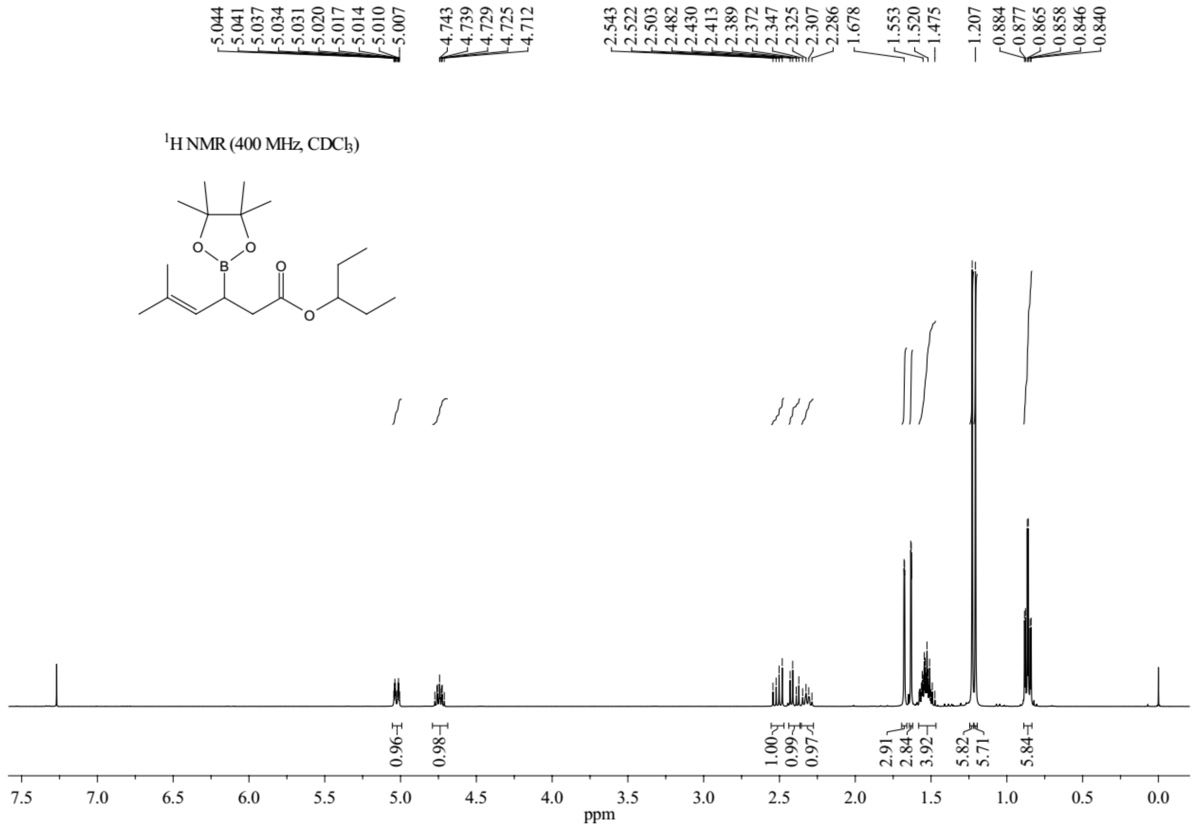

Supplementary Figure 72. ^{13}C NMR spectrum for 5p

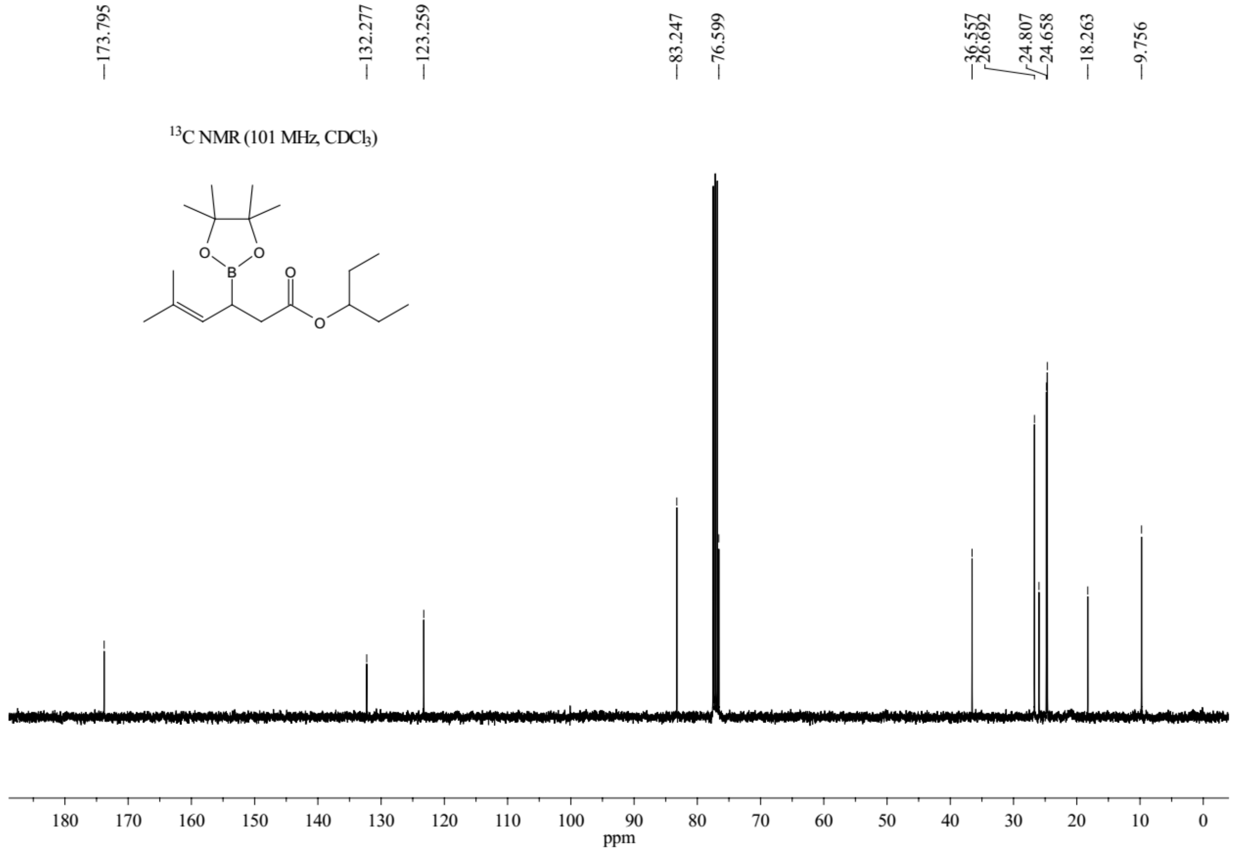

Supplementary Figure 73. ^{1}H NMR spectrum for *N,N*-Diethyl-3-phenyl-3-(4,4,5,5-tetramethyl-1,3,2-dioxaborolan-2-yl)propanamide (5q)

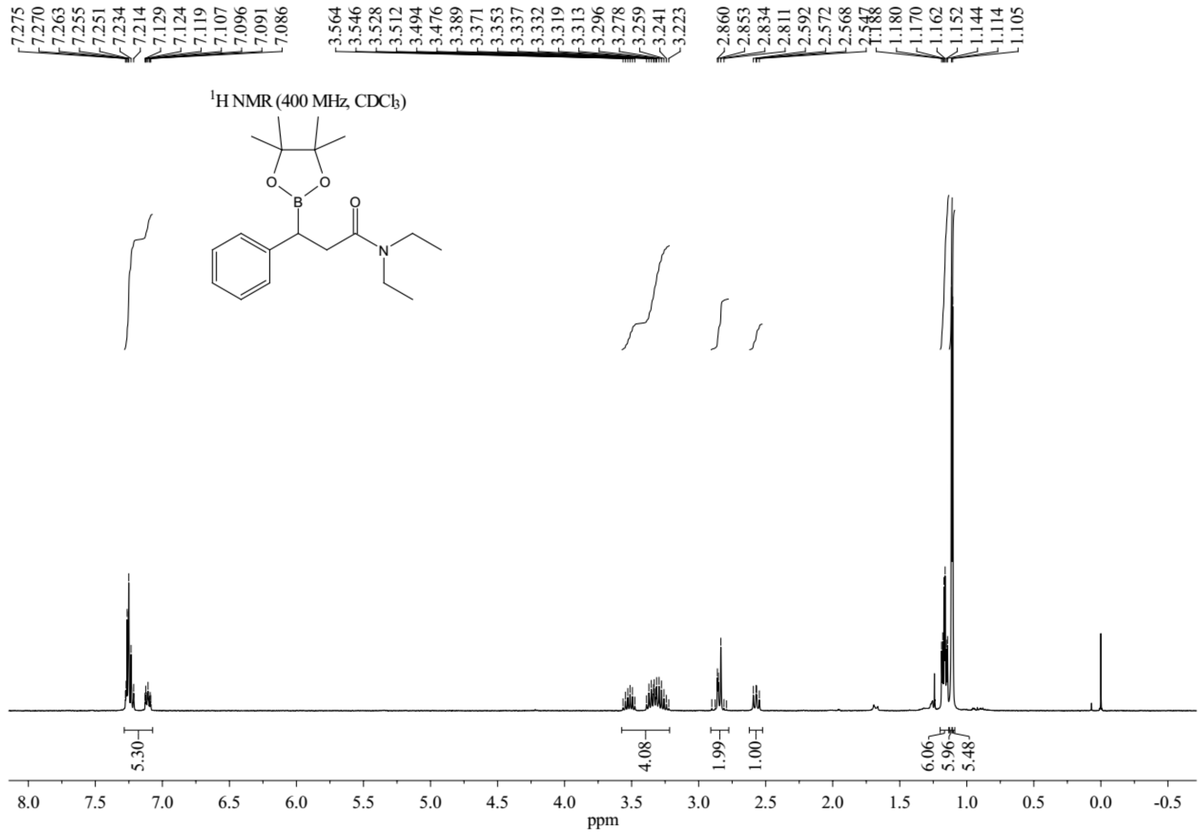

Supplementary Figure 74. ^{13}C NMR spectrum for 5q

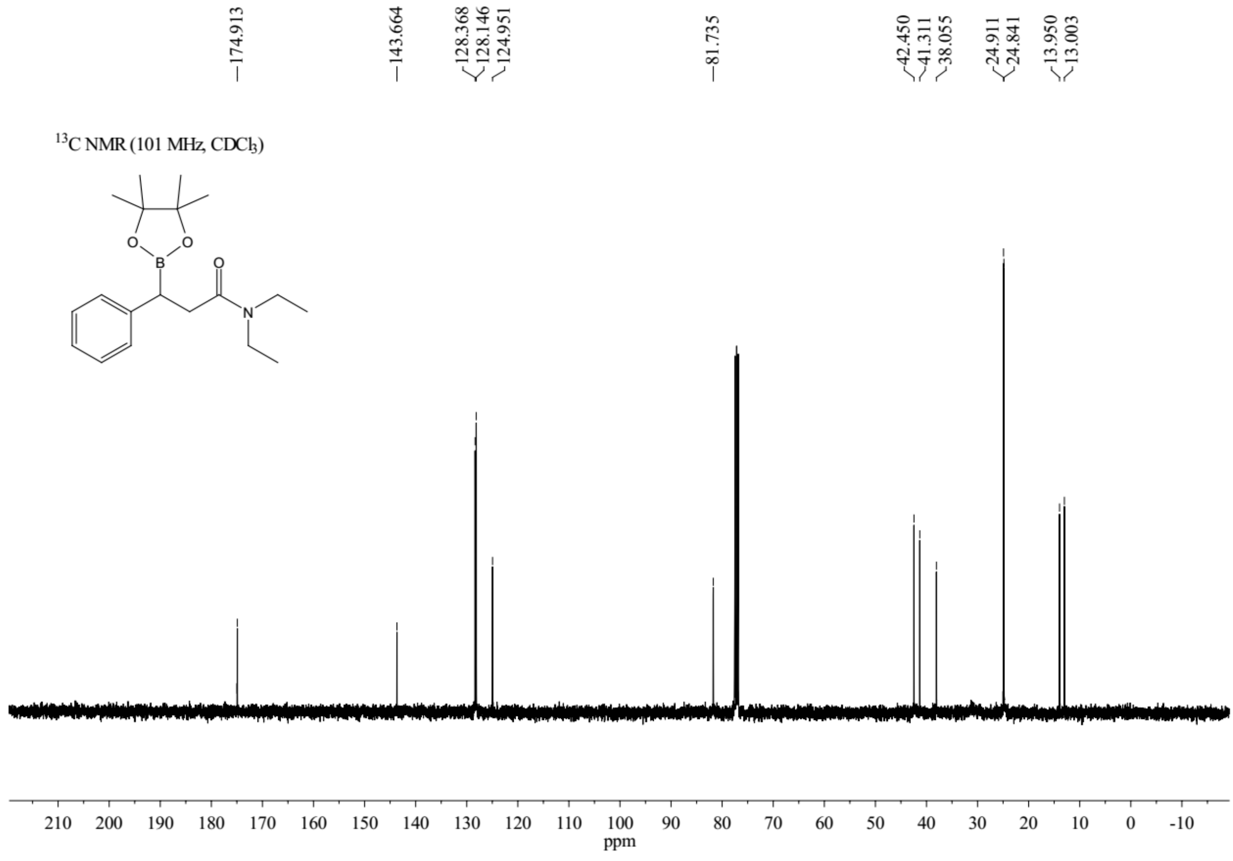

Supplementary Figure 75. ^{1}H NMR spectrum for *anti-tert*-Butyl-2-(phenyl(4,4,5,5-tetramethyl-1,3,2-dioxaborolan-2-yl)methyl)pent-4-enoate (3c)

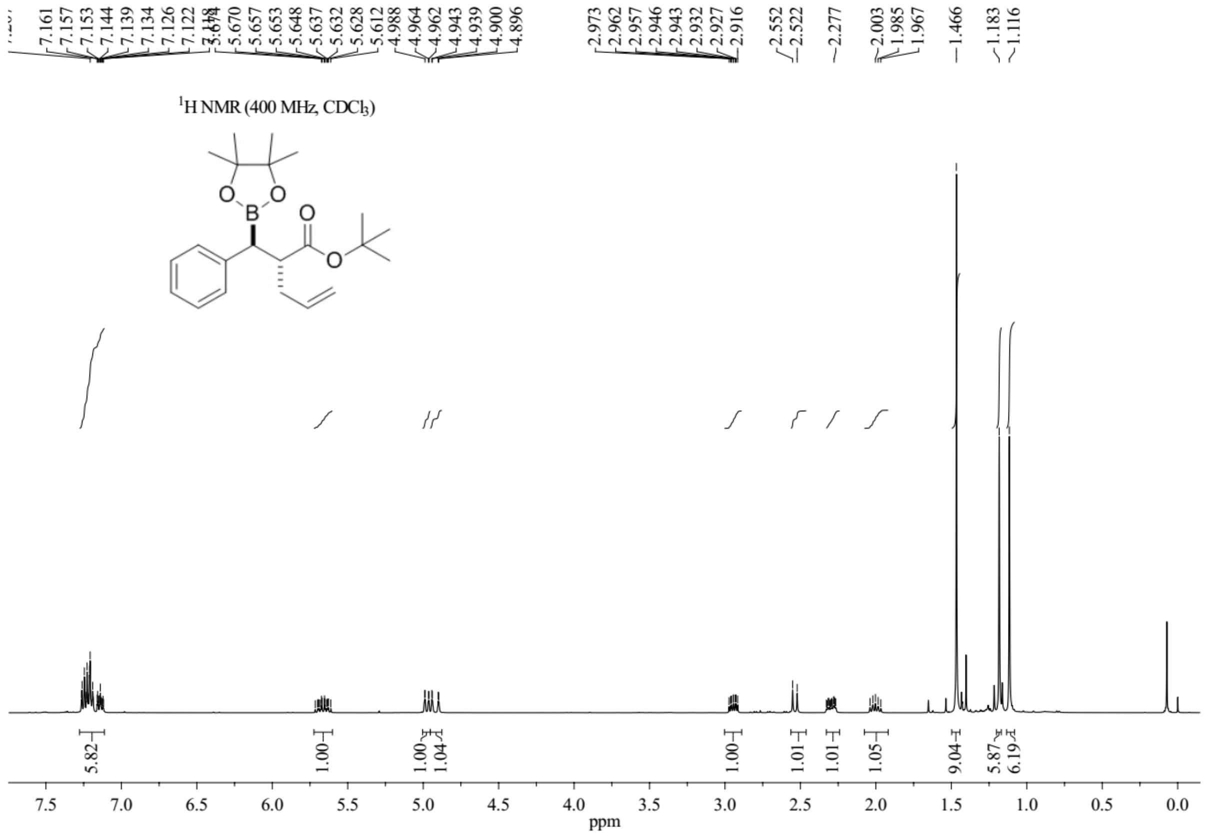

Supplementary Figure 76. ^{13}C NMR spectrum for 3c

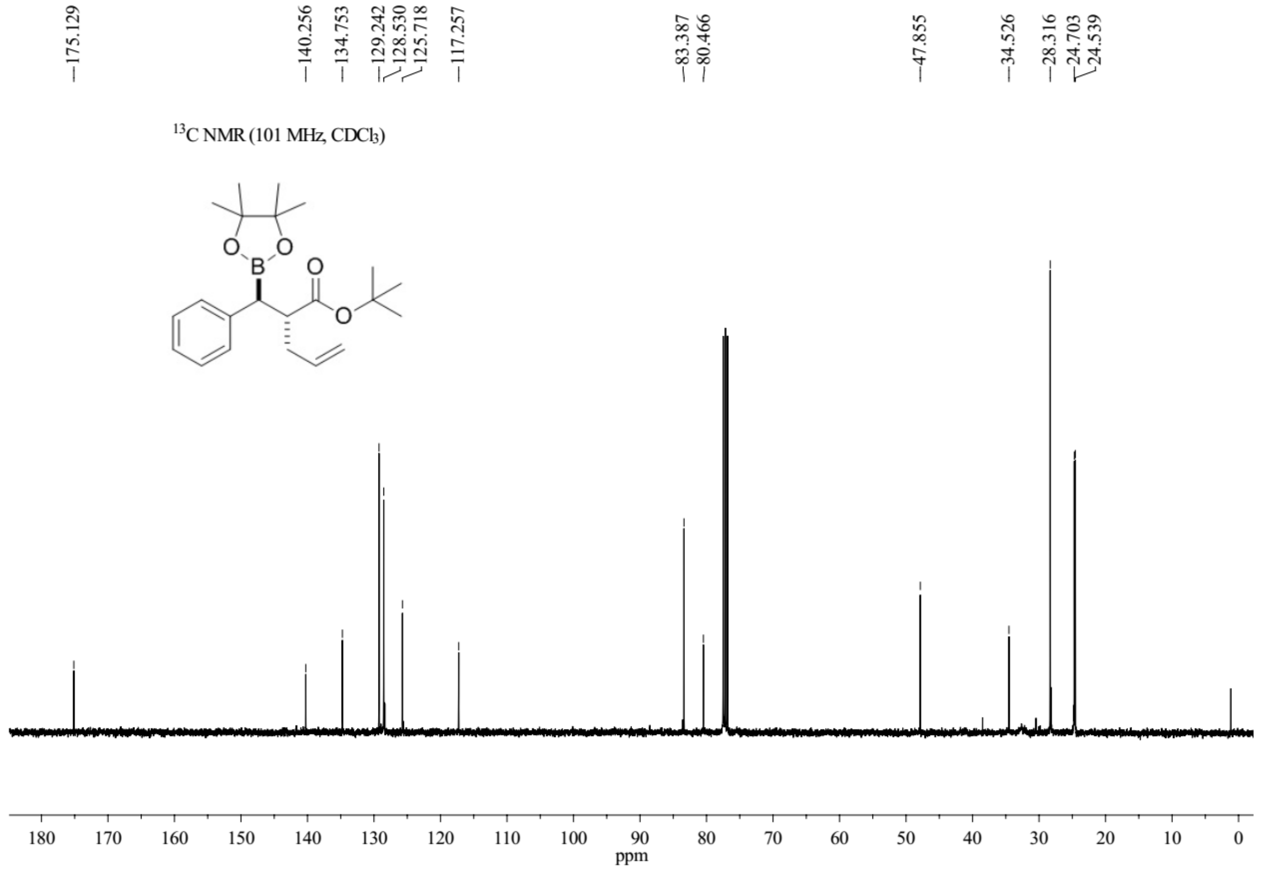

Supplementary Figure 77. ^{1}H NMR spectrum for *syn*-Pentan-3-yl 2-(phenyl(4,4,5,5-tetramethyl-1,3,2-dioxaborolan-2-yl)methyl)pent-4-enoate (4b)

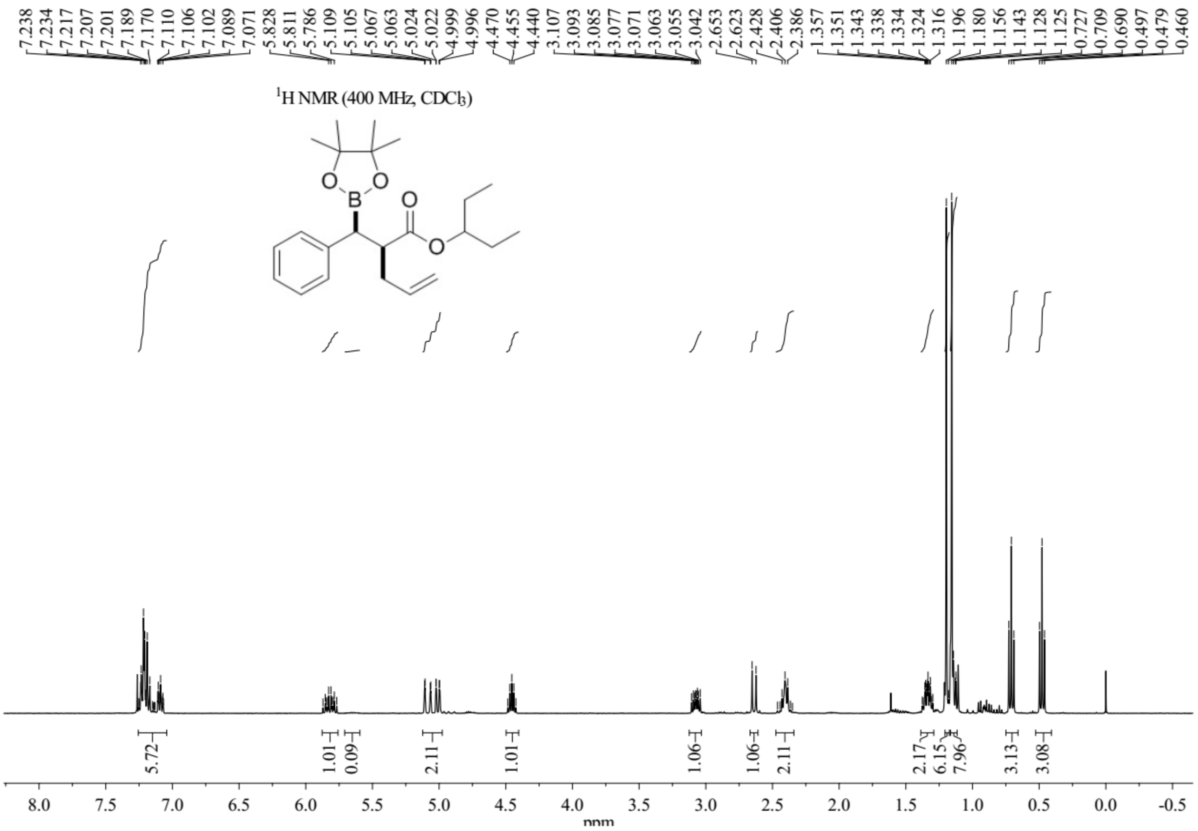

Supplementary Figure 78. ^{13}C NMR spectrum for 4b

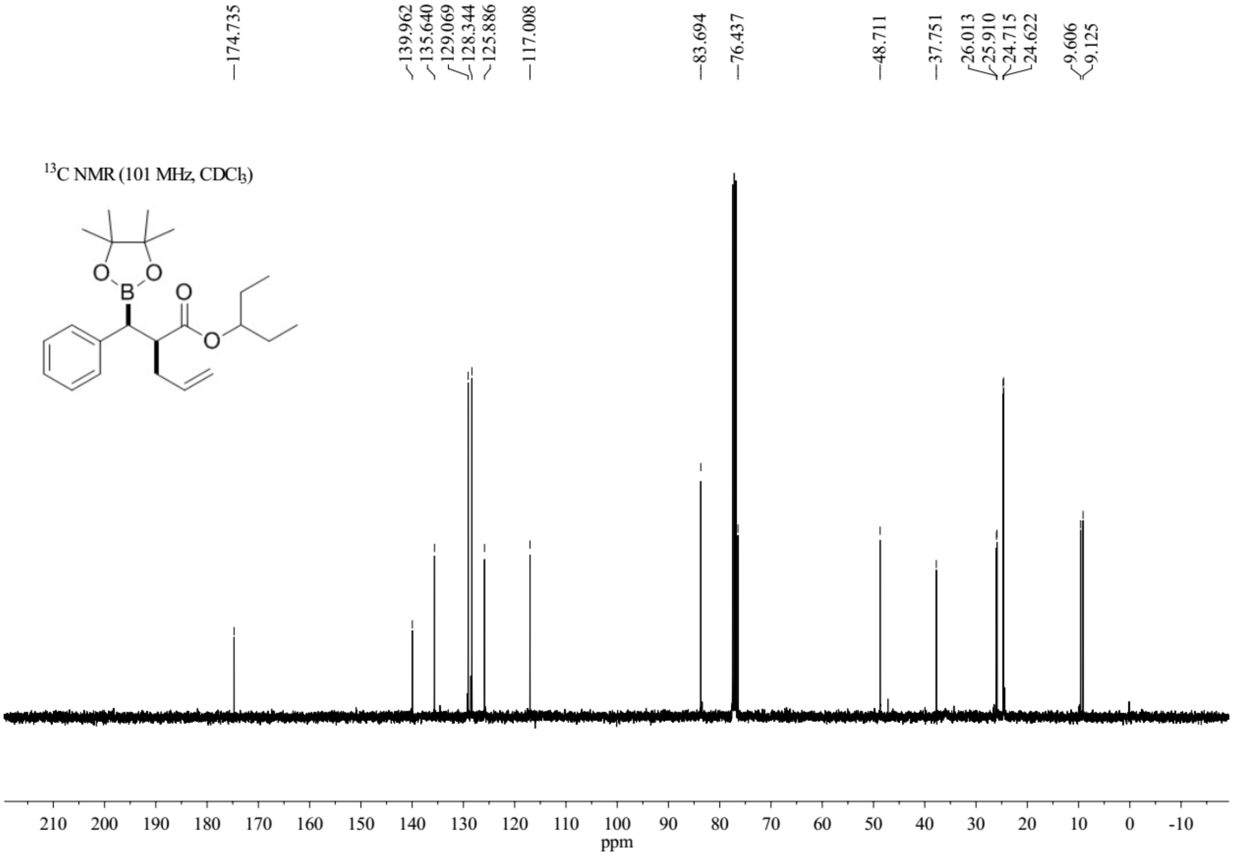

Supplementary Figure 79. ^{1}H NMR spectrum for *anti-tert*-Butyl-2-((4-fluorophenyl)(4,4,5,5-tetramethyl-1,3,2-dioxaborolan-2-yl)methyl)pent-4-enoate (7a)

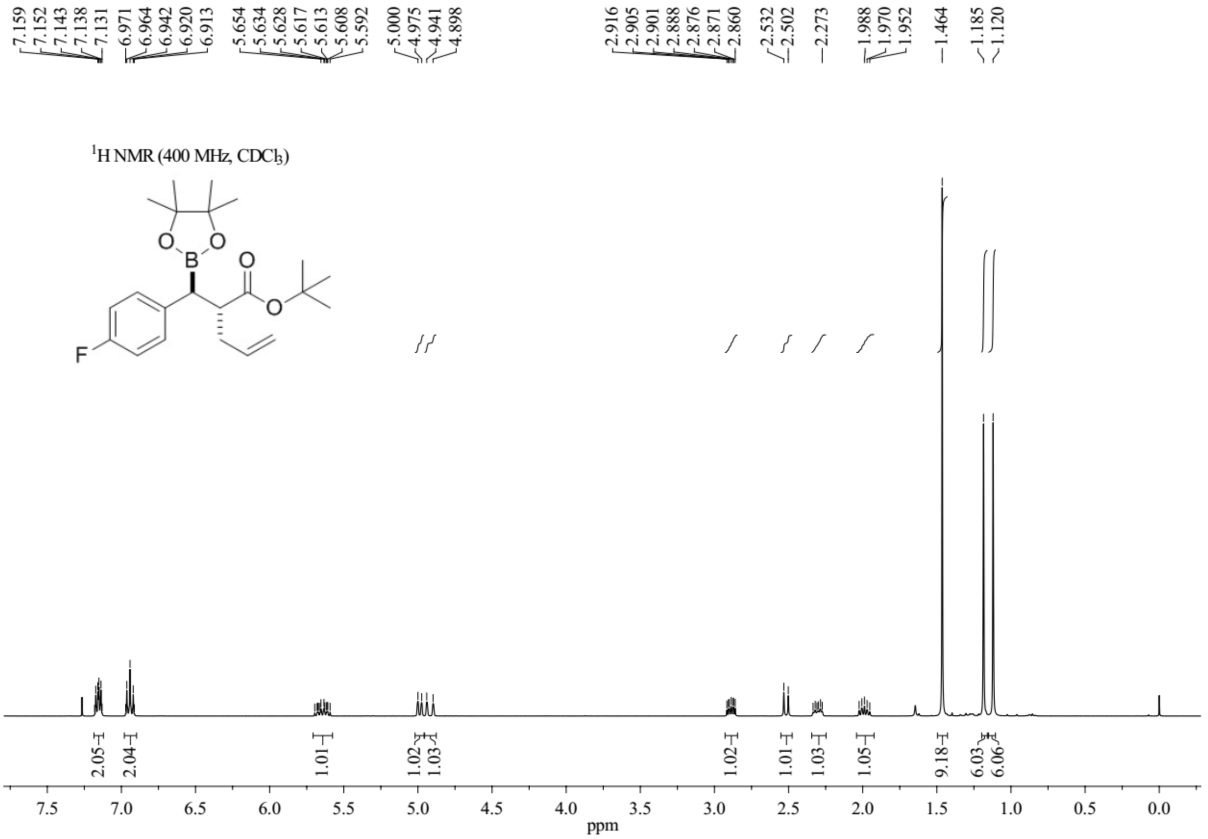

Supplementary Figure 80. ^{13}C NMR spectrum for 7a

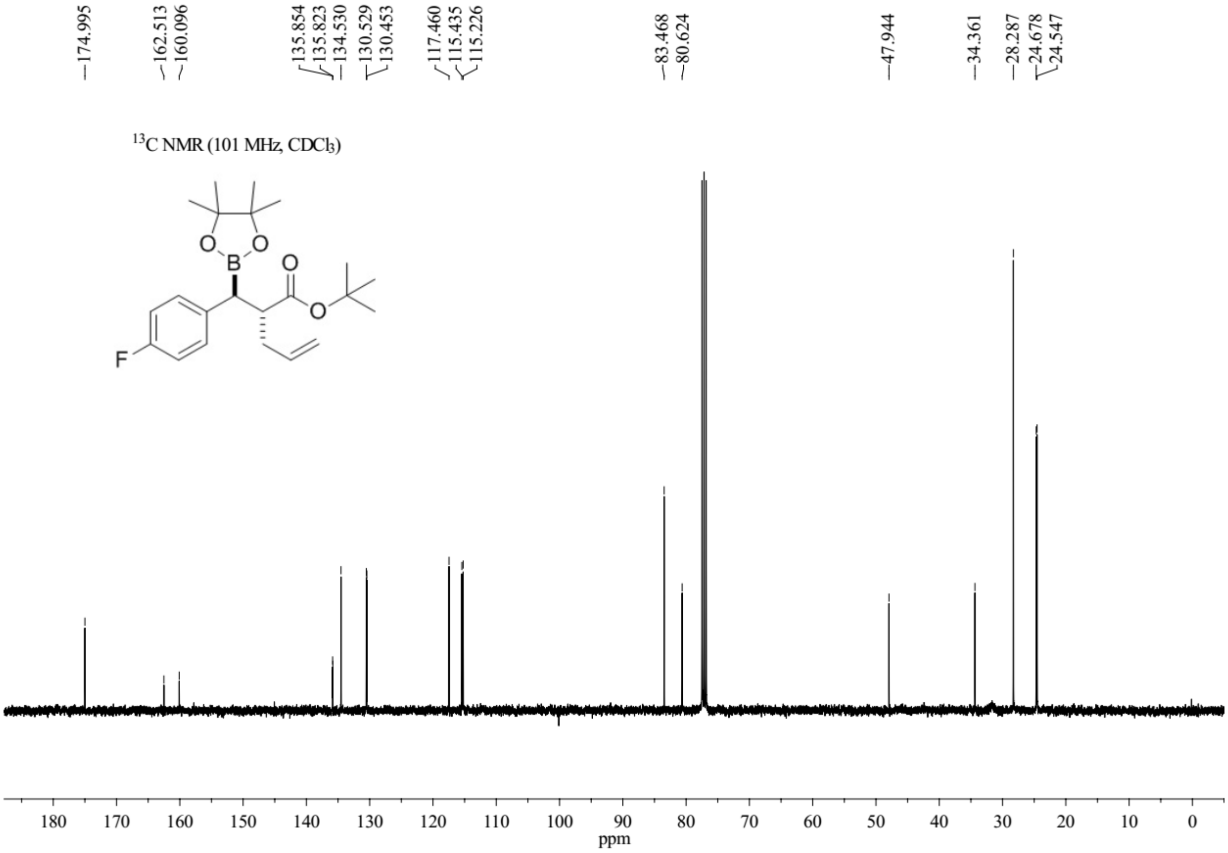

Supplementary Figure 81. ^{1}H NMR spectrum for*syn*-Pentan-3-yl-2-((4-fluorophenyl)(4,4,5,5-tetramethyl-1,3,2-dioxaborolan-2-yl)methyl)pent-4-enoate (8a)

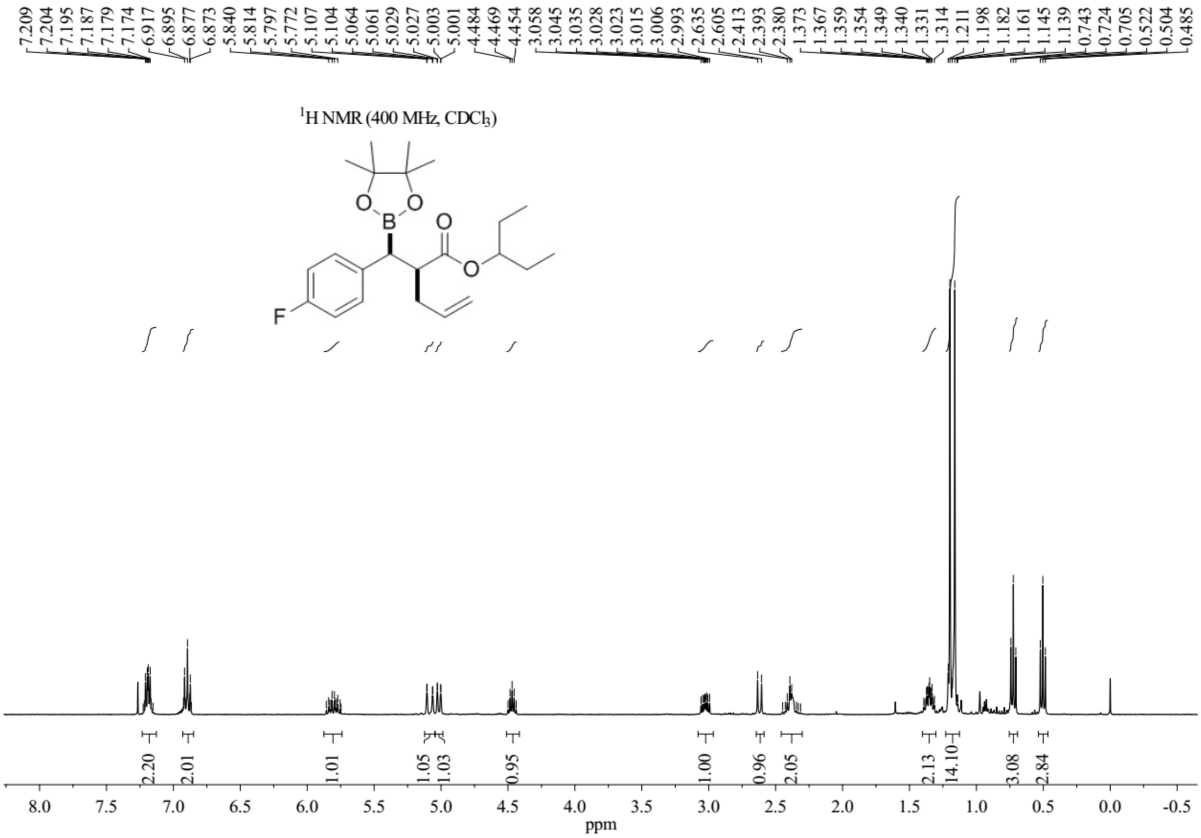

Supplementary Figure 82. ^{13}C NMR spectrum for 8a

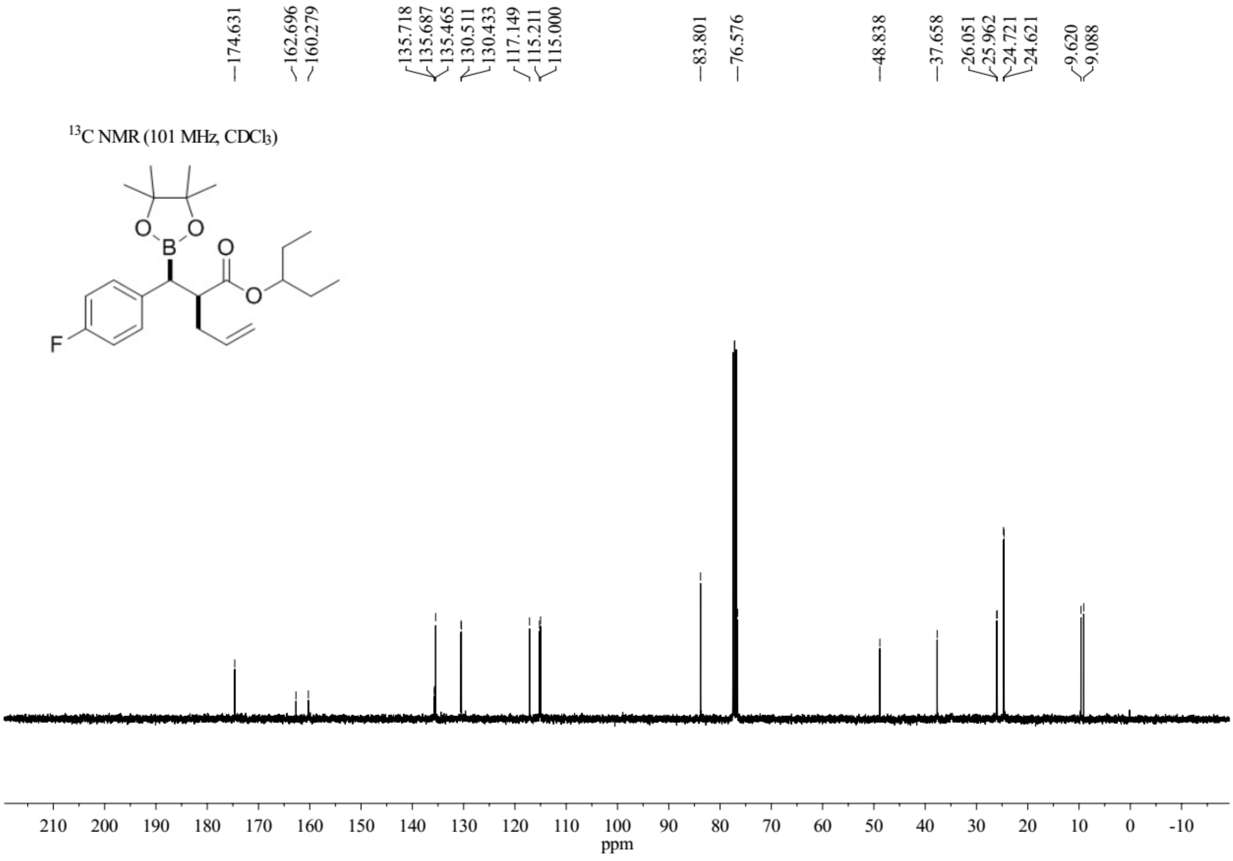

Supplementary Figure 83. **^{1}H NMR spectrum for *anti*-*tert*-Butyl-2-((3-chlorophenyl)(4,4,5,5-tetramethyl-1,3,2-dioxaborolan-2-yl)methyl)pent-4-enoate (7b)**

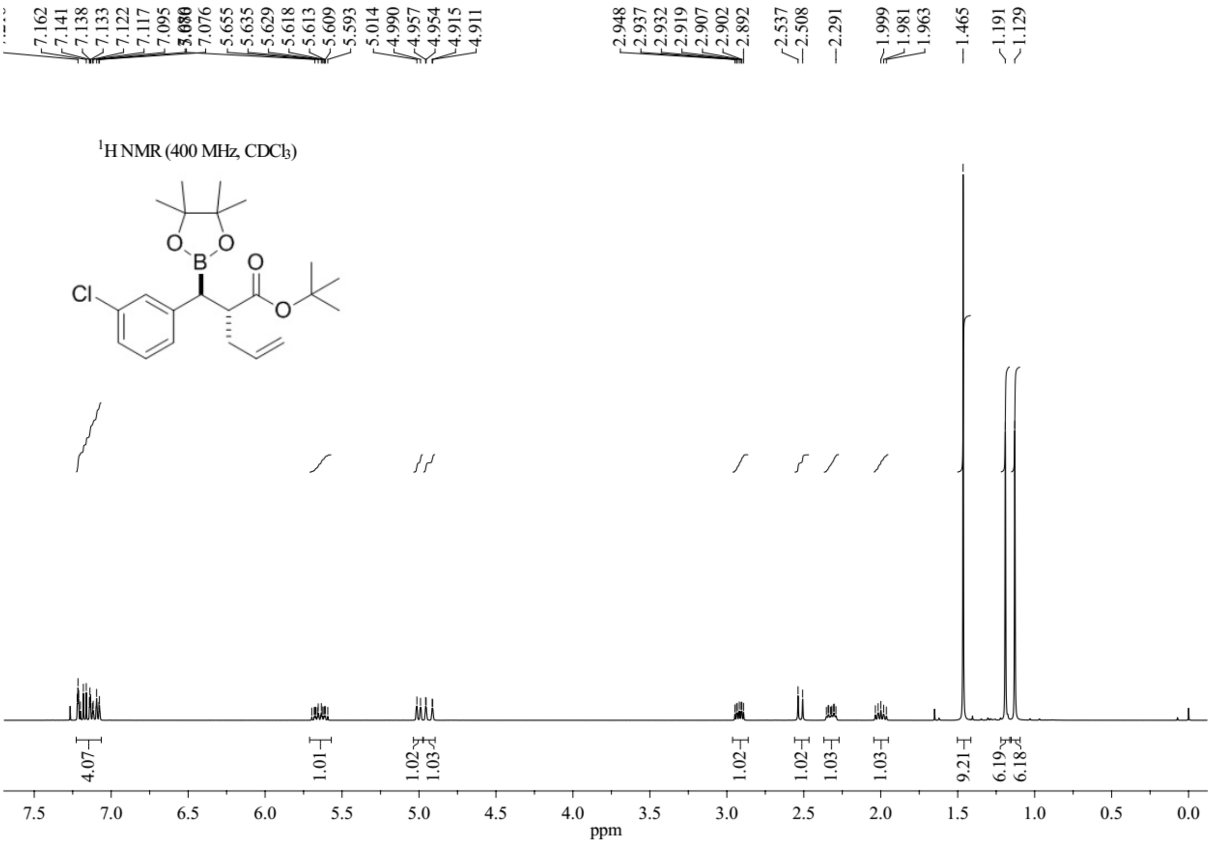

Supplementary Figure 84. **^{13}C NMR spectrum for 7b**

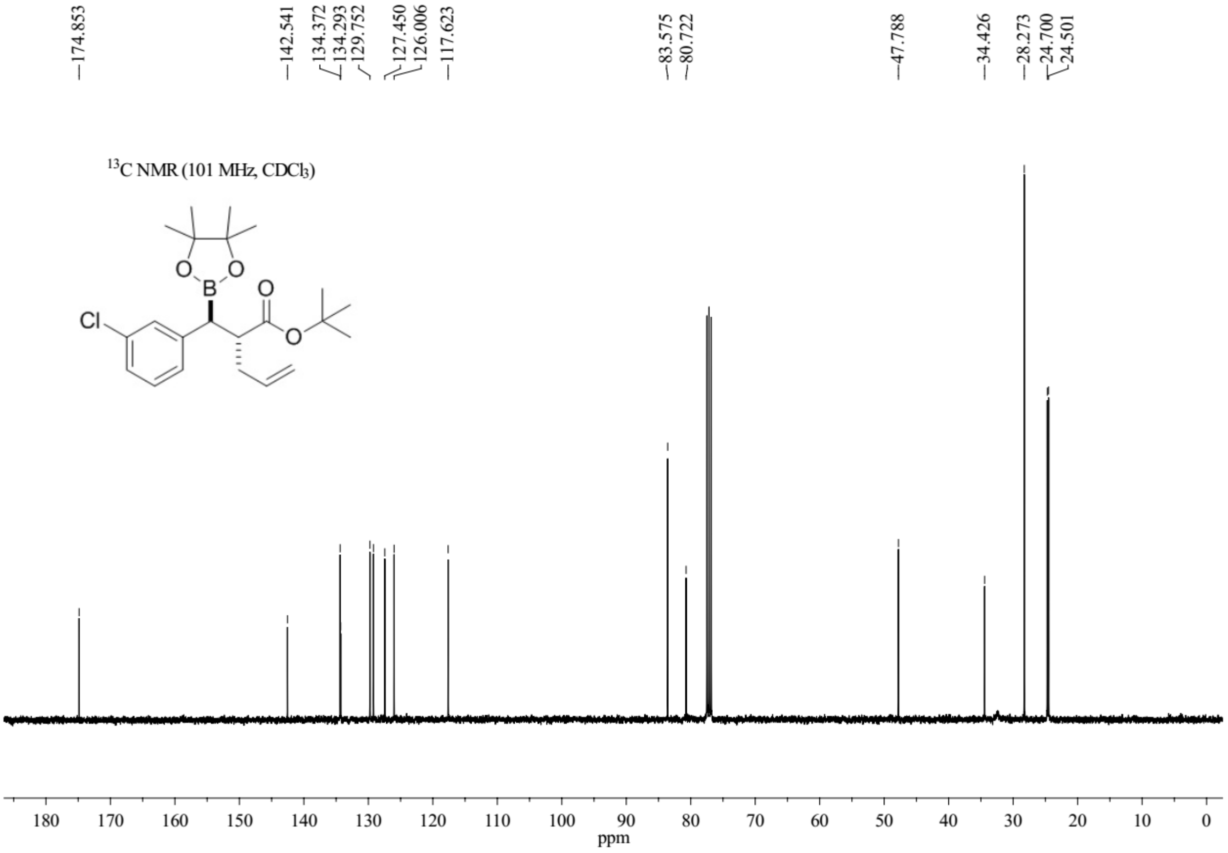

Supplementary Figure 85. ^{1}H NMR spectrum for *syn*-Pentan-3-yl-2-((3-chlorophenyl)(4,4,5,5-tetramethyl-1,3,2-dioxaborolan-2-yl)methyl)pent-4-enoate (8b)

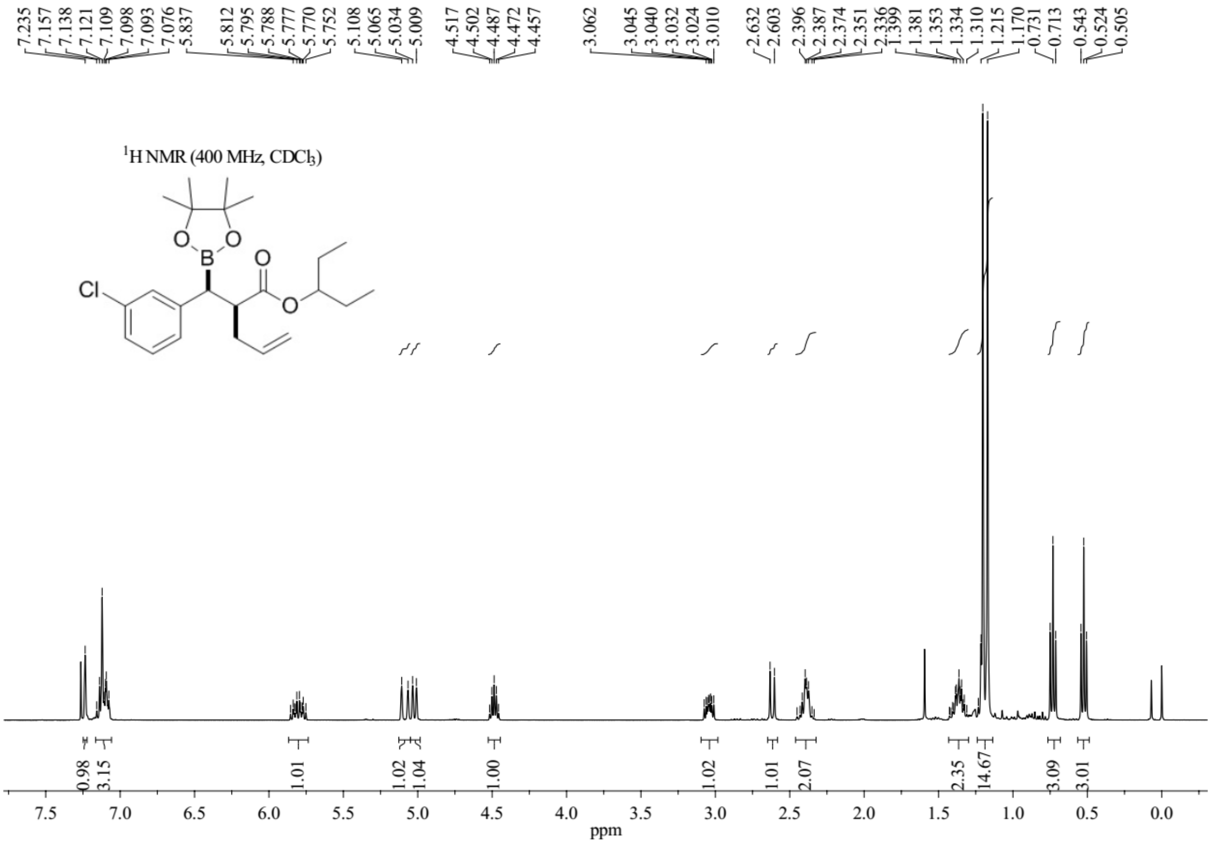

Supplementary Figure 86. ^{13}C NMR spectrum for 8b

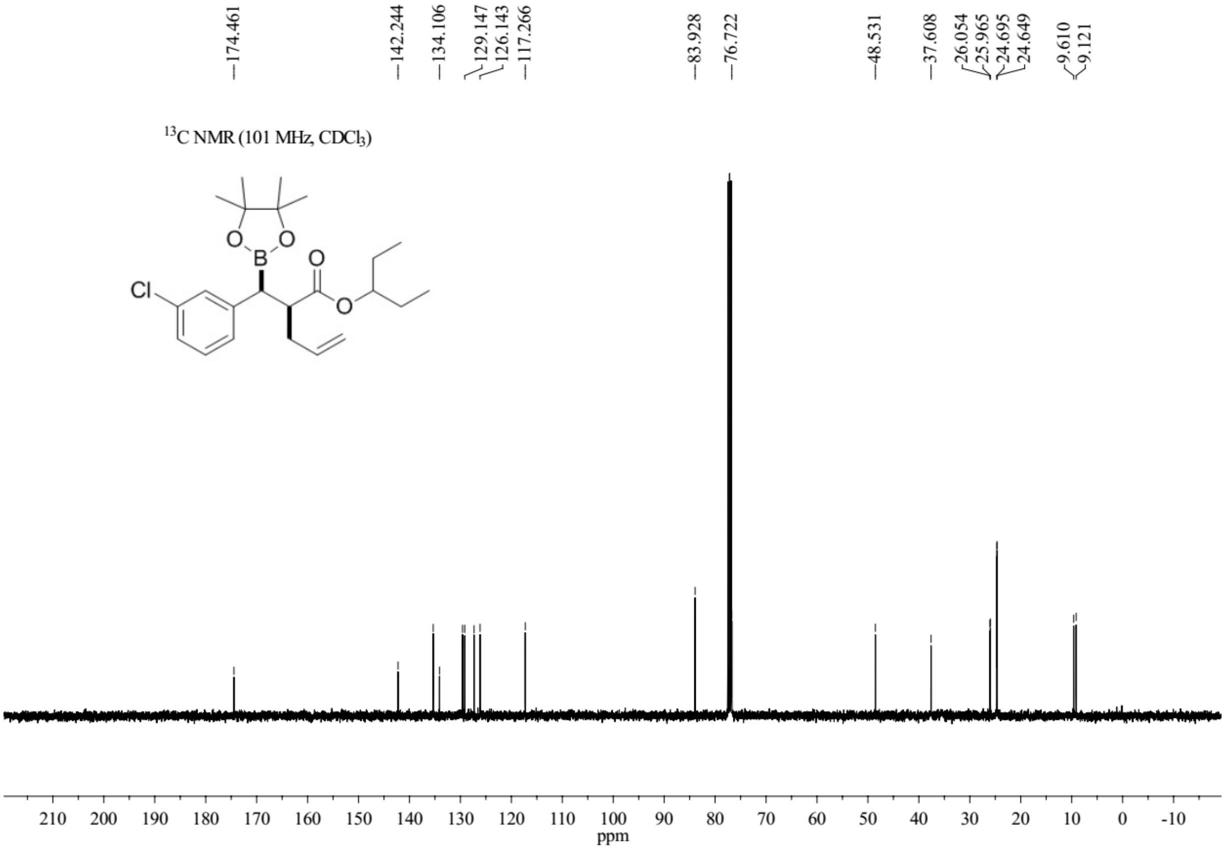

Supplementary Figure 87. ^{1}**H NMR spectrum for** ***anti-tert*****-Butyl-2-((3-bromophenyl)(4,4,5,5-tetramethyl-1,3,2-dioxaborolan-2-yl)methyl)pent-4-enoate (7c)**

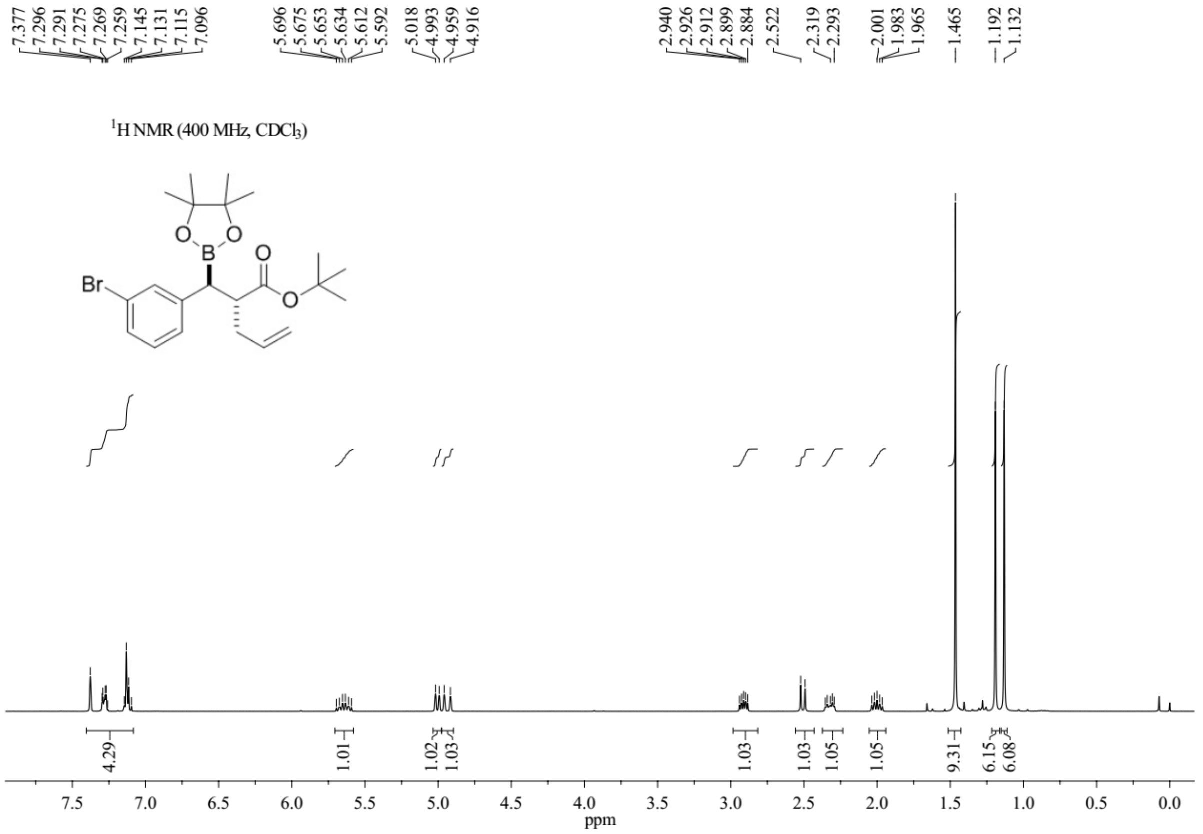

Supplementary Figure 88. ^{13}**C NMR spectrum for 7c**

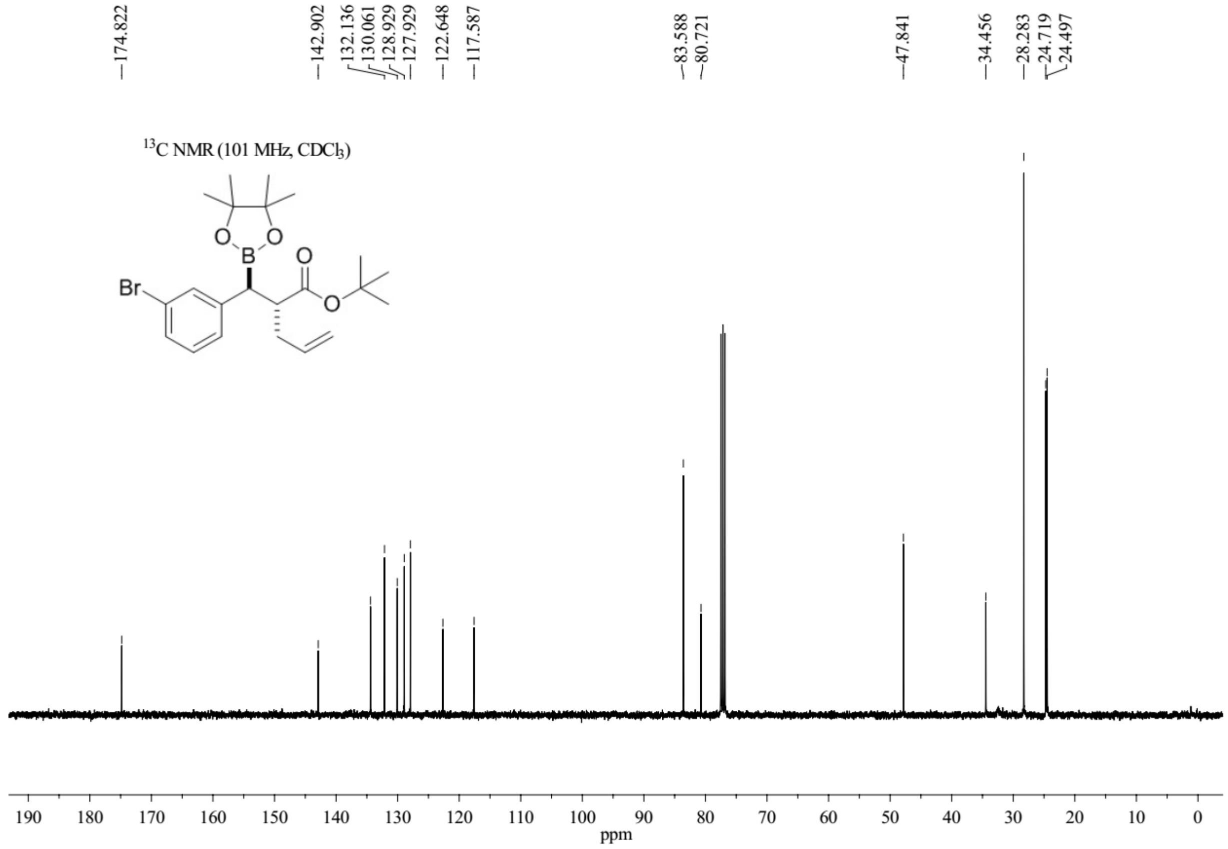

Supplementary Figure 89. ^{1}H NMR spectrum for *syn*-Pentan-3-yl-2-((3-bromophenyl)(4,4,5,5-tetramethyl-1,3,2-dioxaborolan-2-yl)methyl)pent-4-enoate (8c)

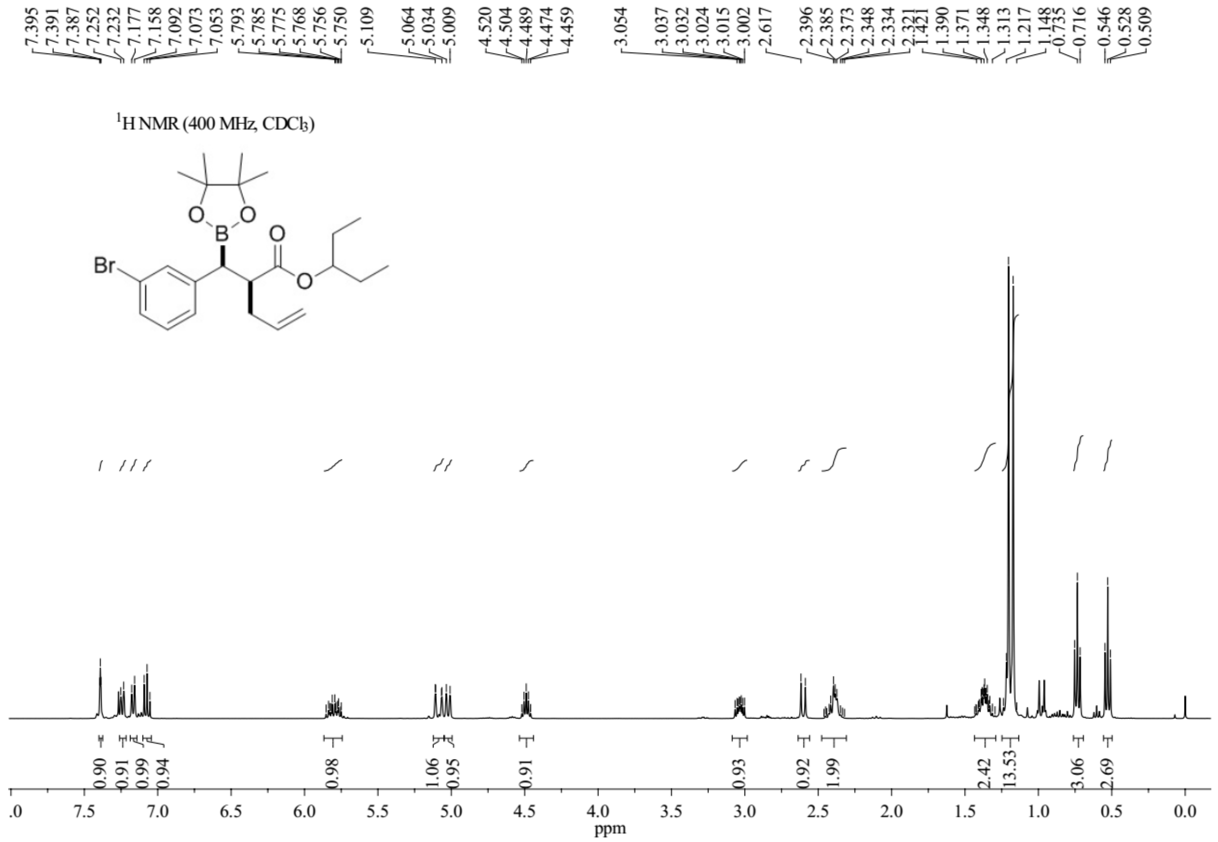

Supplementary Figure 90. ^{13}C NMR spectrum for 8c

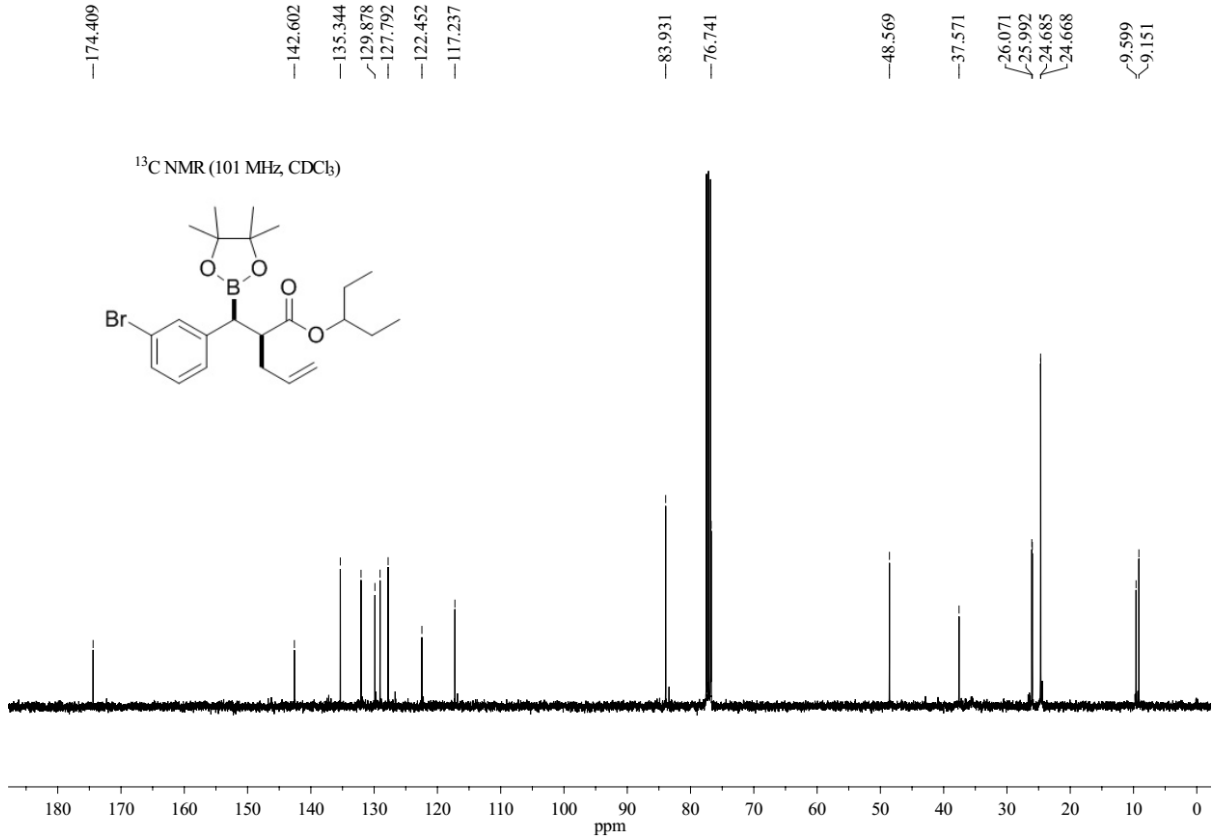

Supplementary Figure 91. 1H NMR spectrum for *anti-tert*-Butyl-2-((4,4,5,5-tetramethyl-1,3,2-dioxaborolan-2-yl)(3(trifluoromethoxy)phenyl)methy l)pent-4-enoate (7d)

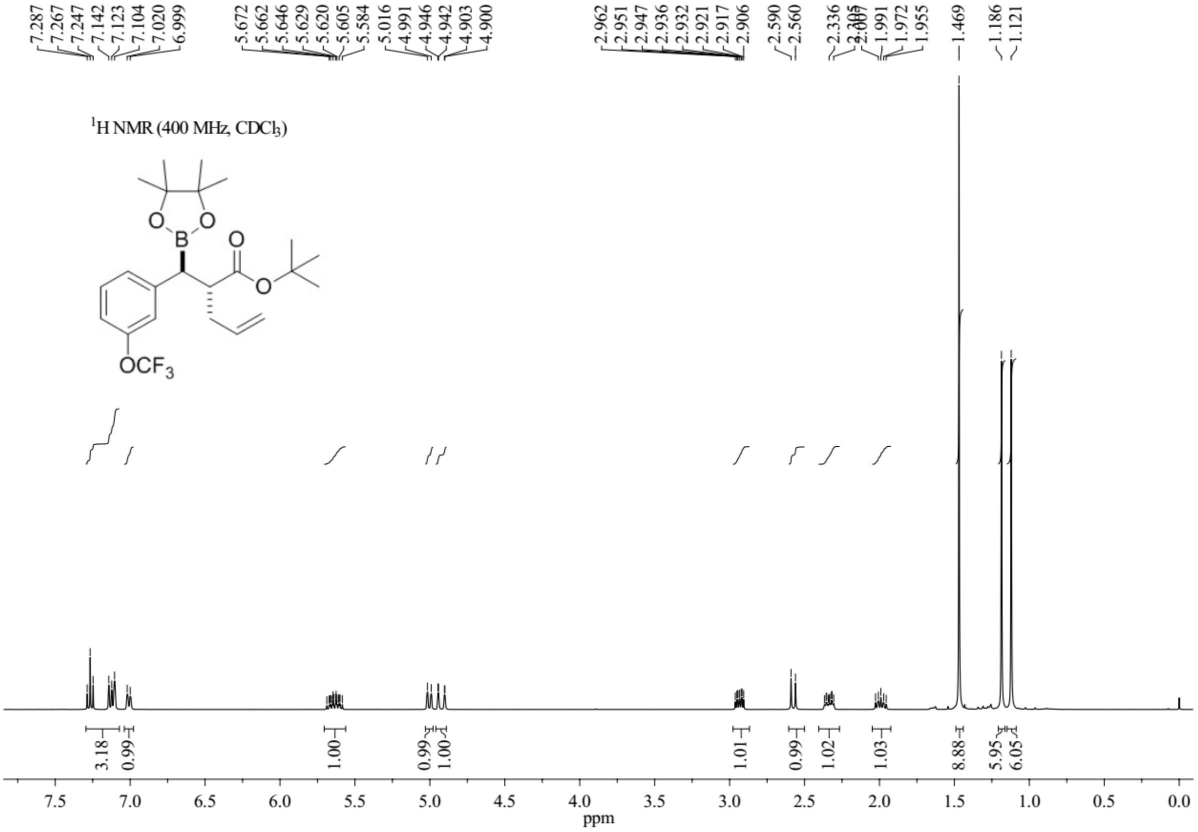

Supplementary Figure 92. 13C NMR spectrum for 7d

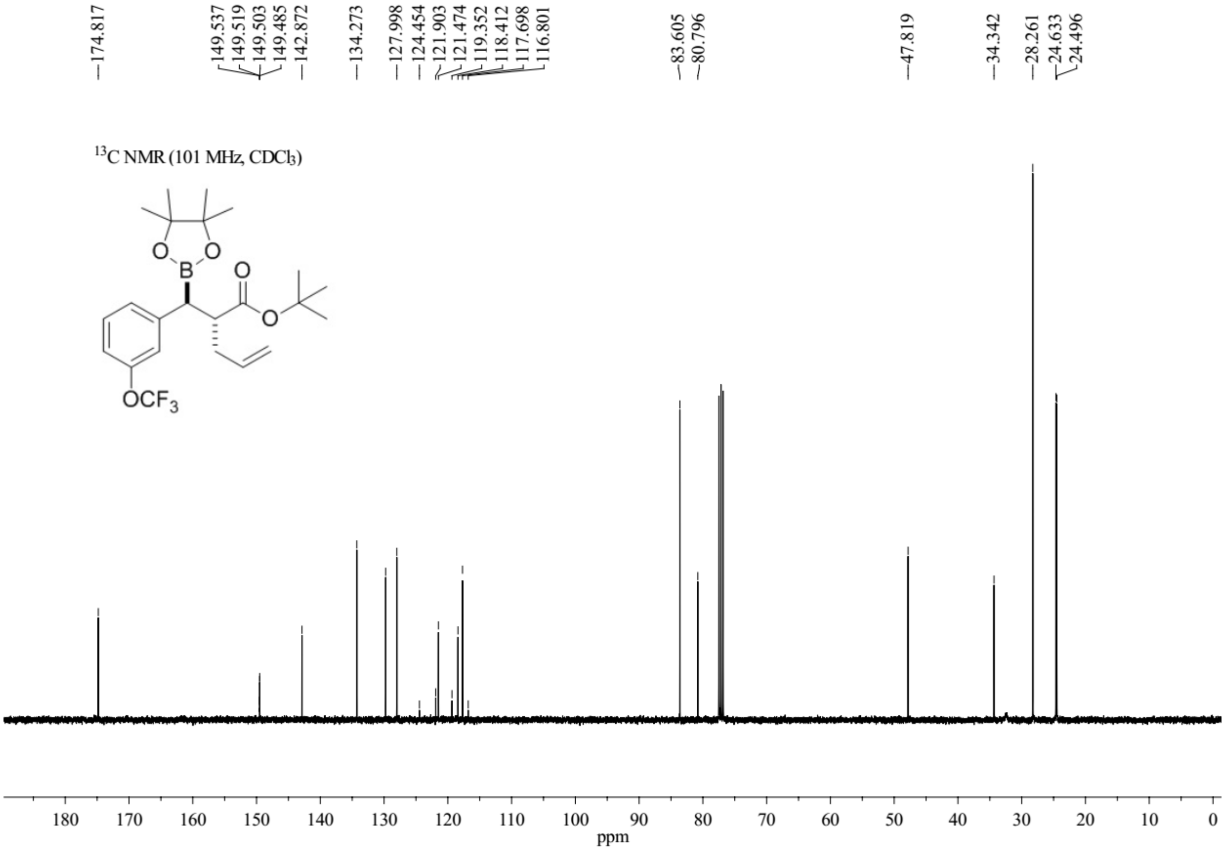

Supplementary Figure 93. ^{1}H NMR spectrum for *syn*-Pentan-3-yl-2-((4,4,5,5-tetramethyl-1,3,2-dioxaborolan-2-yl)(3-(trifluoromethoxy)phenyl)me thyl)pent-4-enoate (8d)

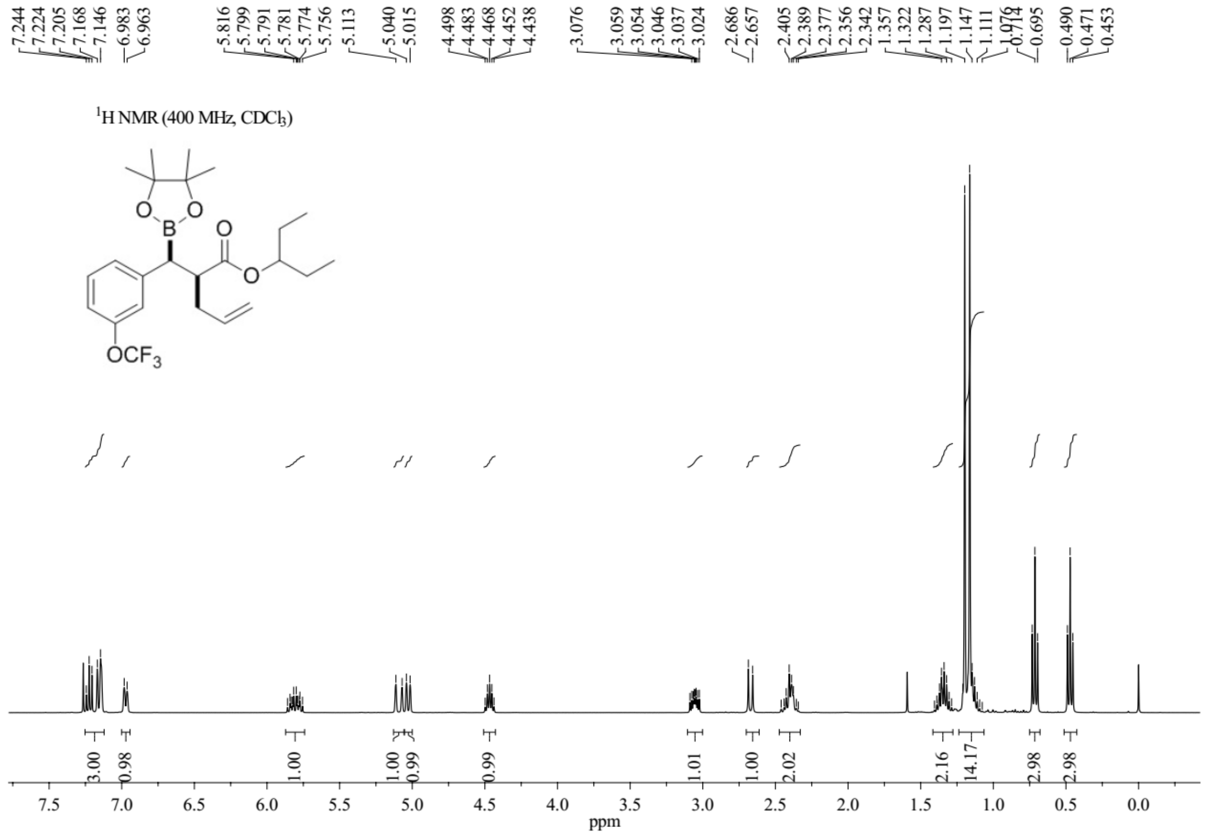

Supplementary Figure 94. ^{13}C NMR spectrum for 8d

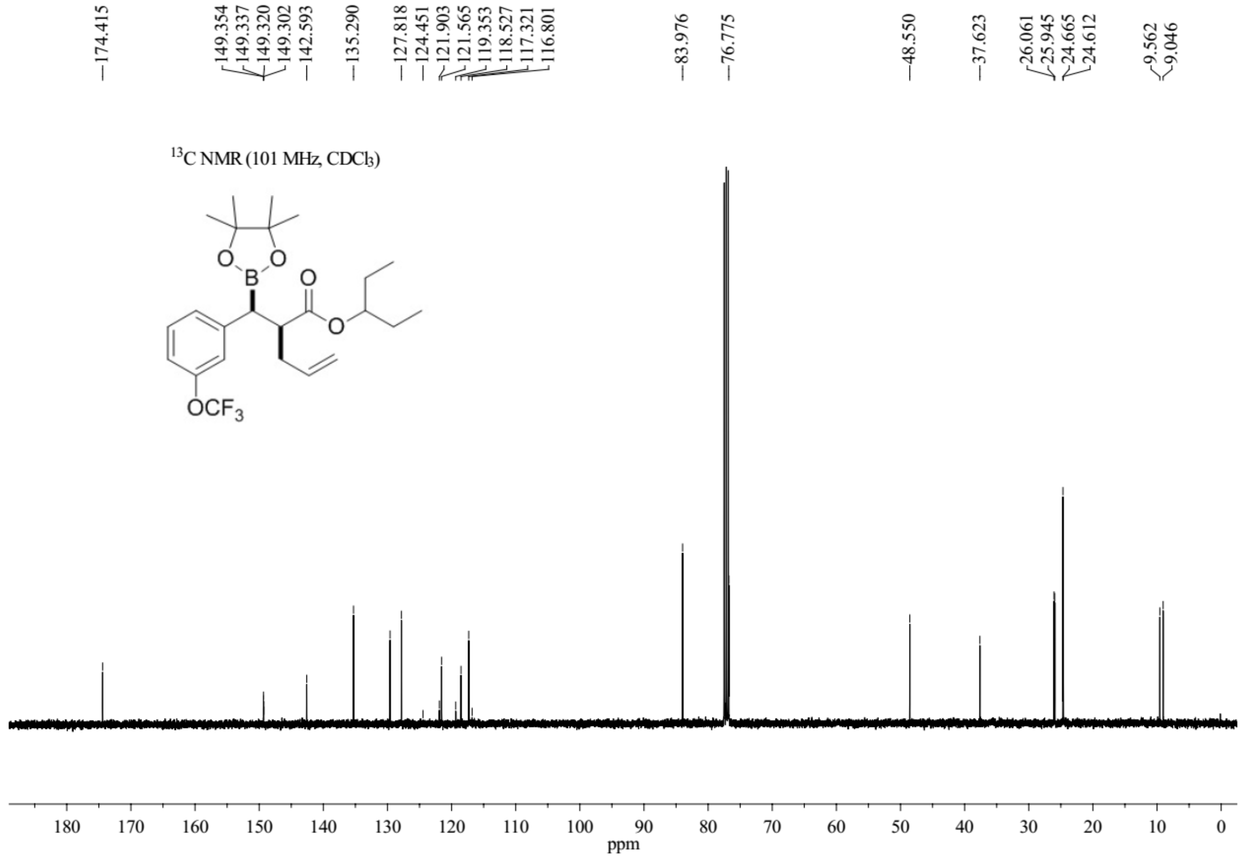

Supplementary Figure 95. ^{1}H NMR spectrum for *anti-tert*-Butyl-2-((4-methoxyphenyl)(4,4,5,5-tetramethyl-1,3,2-dioxaborolan-2-yl)methyl)pent-4-enoate (7e)

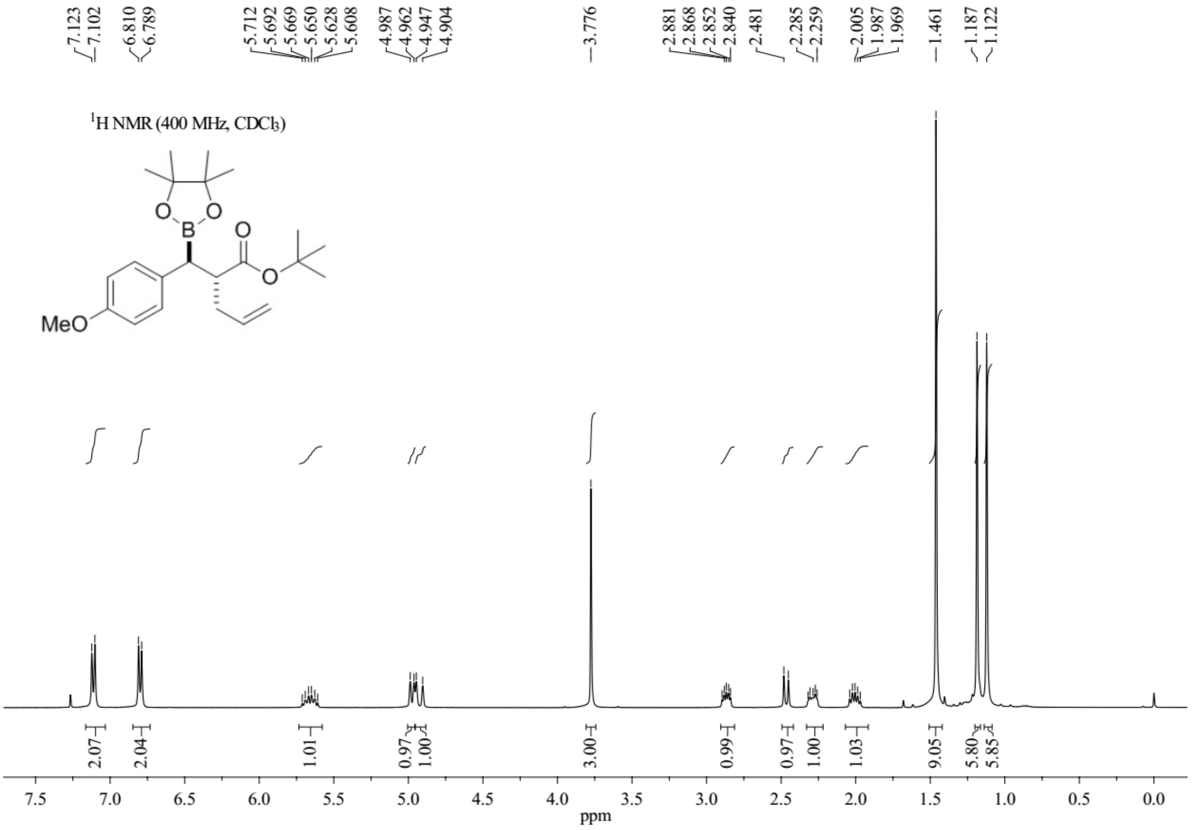

Supplementary Figure 96. ^{13}C NMR spectrum for 7e

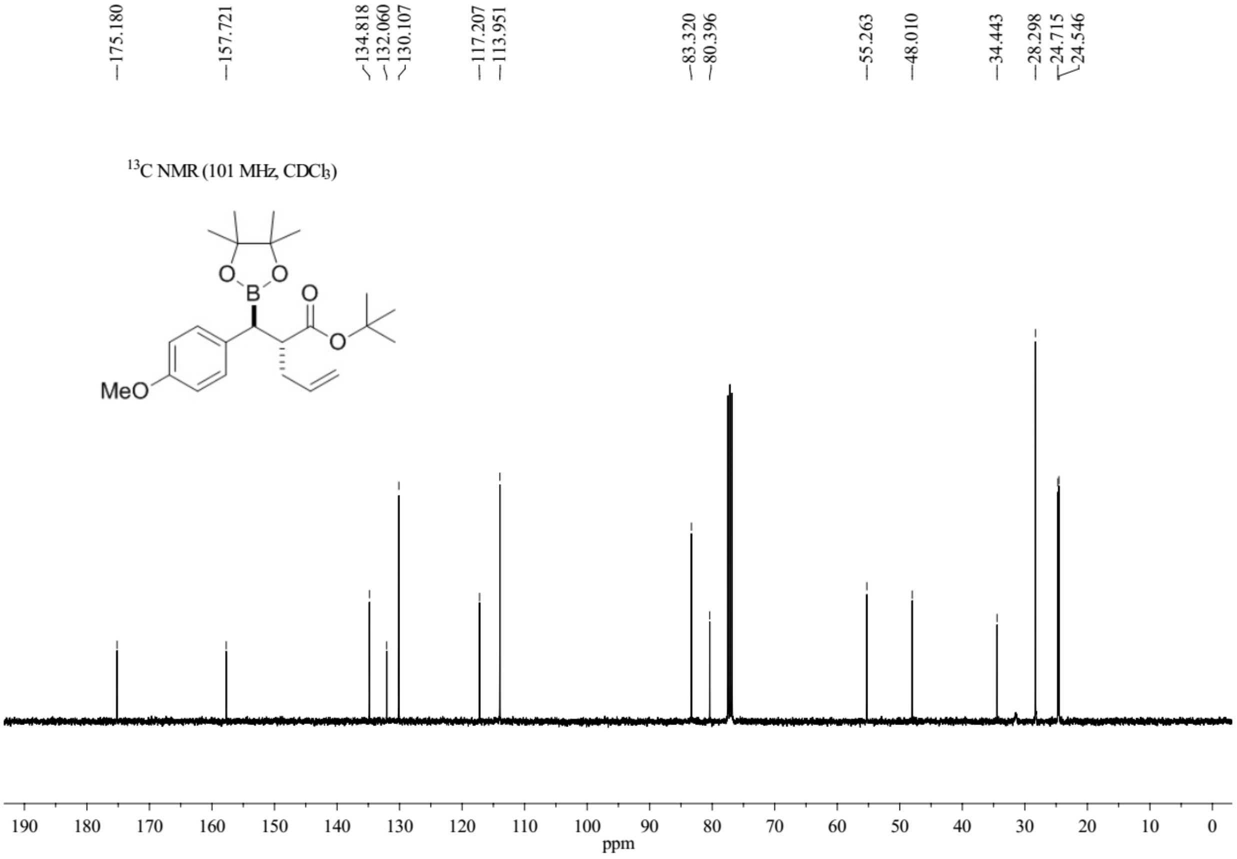

Supplementary Figure 97. ^{1}H NMR spectrum for *syn*-Pentan-3-yl-2-((4-methoxyphenyl)(4,4,5,5-tetramethyl-1,3,2-dioxaborolan-2-yl)methyl)pent-4-enoate (8e)

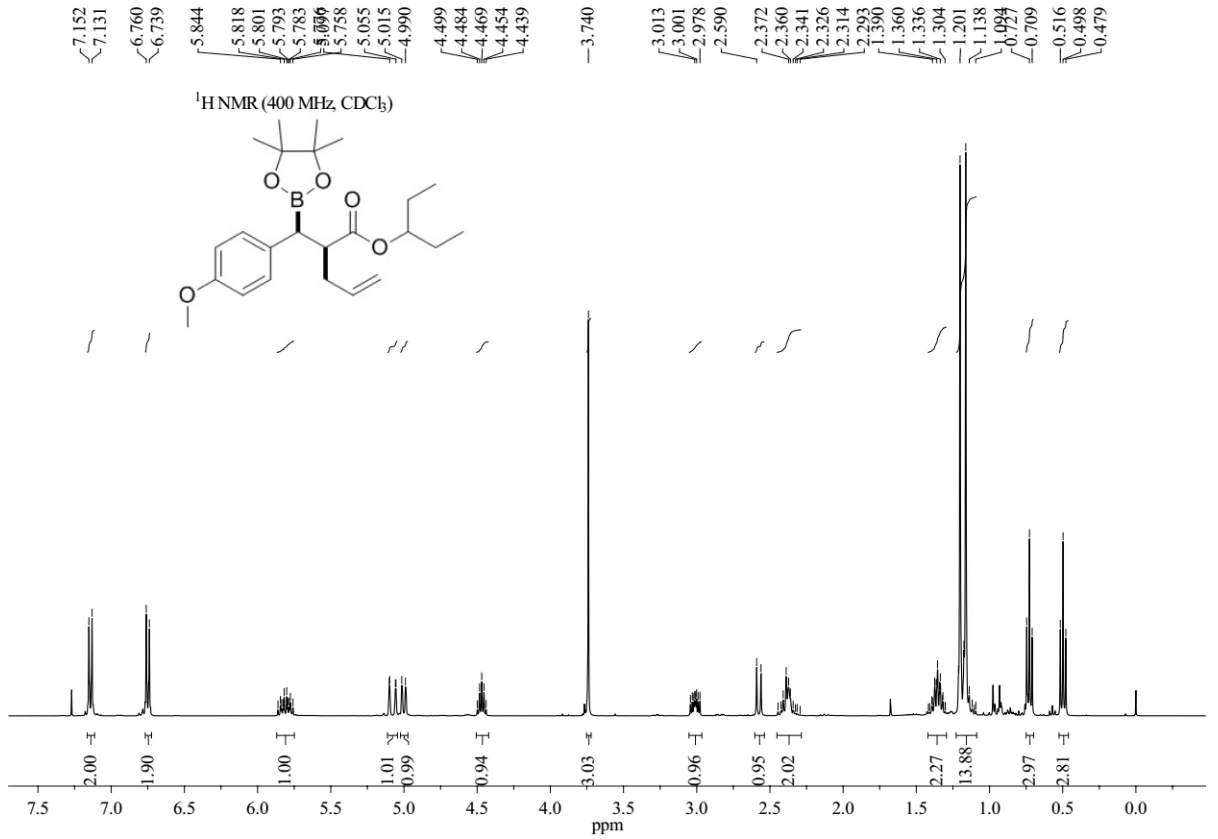

Supplementary Figure 98. ^{13}C NMR spectrum for 8e

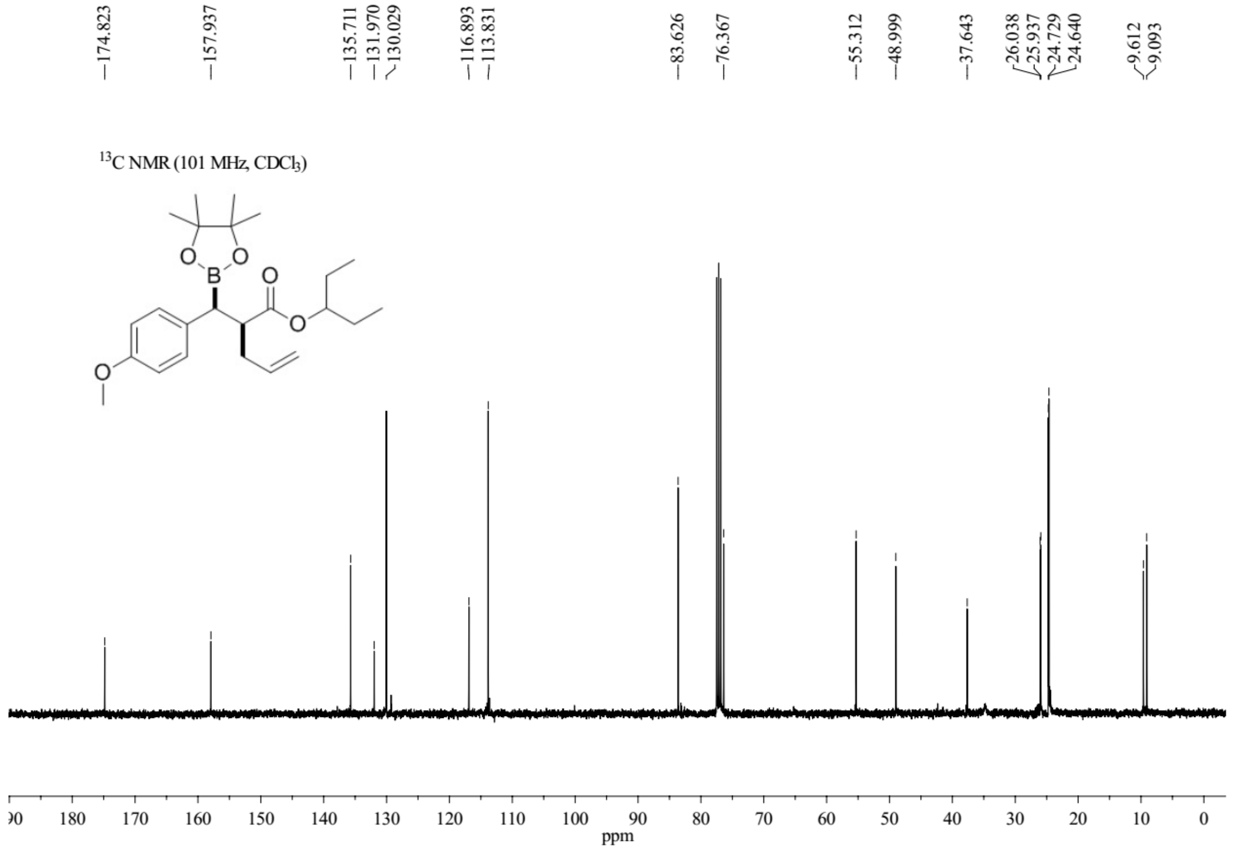

Supplementary Figure 99. ^{1}**H NMR spectrum for*anti-tert*-Butyl-2-((4,4,5,5-tetramethyl-1,3,2-dioxaborolan-2-yl)(o-tolyl)methyl)pent-4-enoate (7f)**

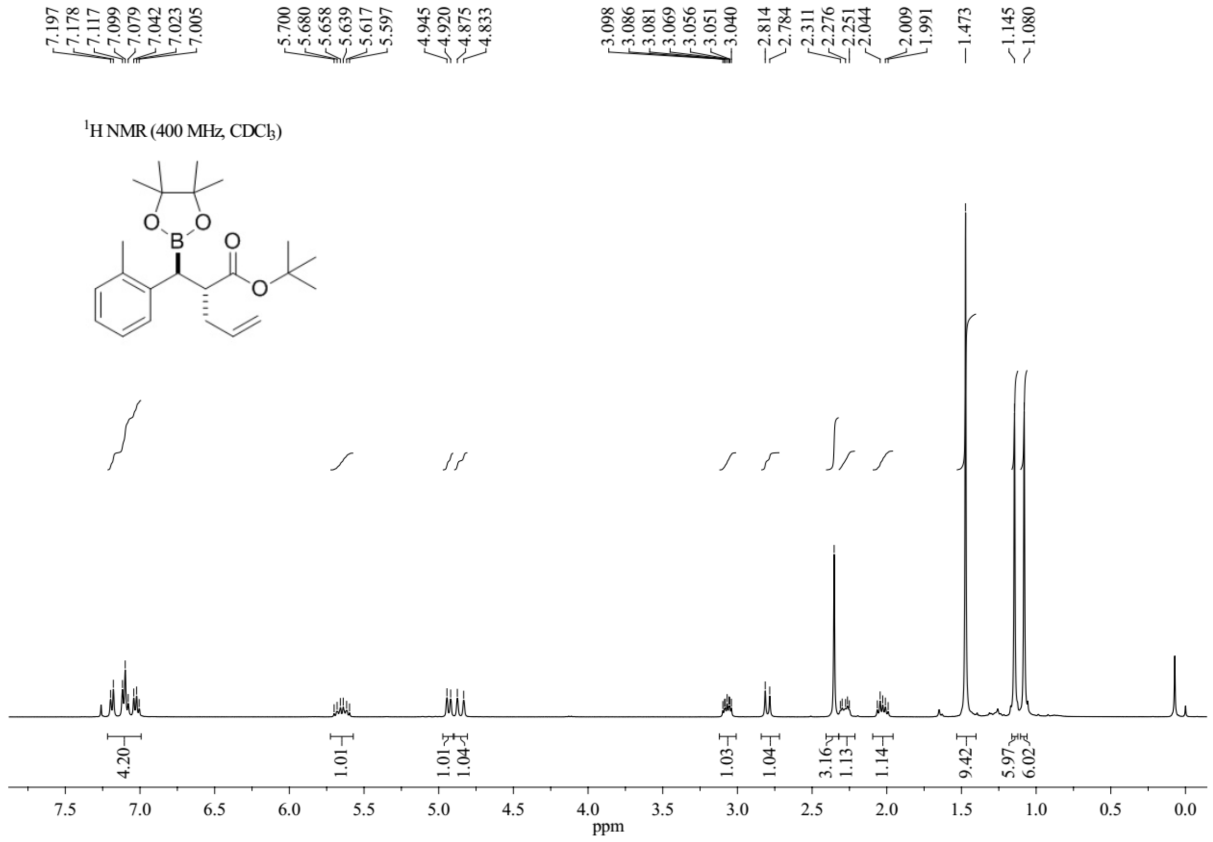

Supplementary Figure 100. ^{13}**C NMR spectrum for 7f**

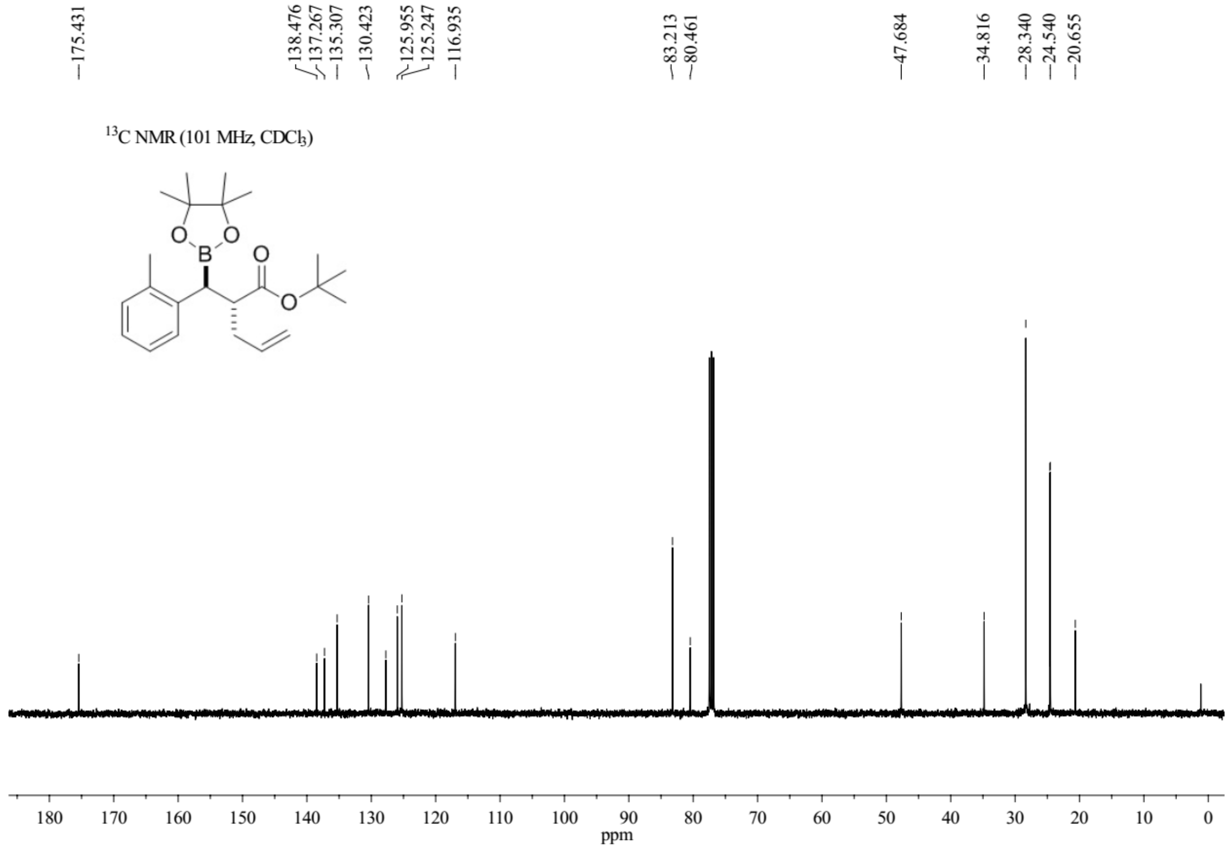

Supplementary Figure 101. **^{1}H NMR spectrum for *syn*-Pentan-3-yl-2-((4,4,5,5-tetramethyl-1,3,2-dioxaborolan-2-yl)(o-tolyl)methyl)pent-4-enoate (8f)**

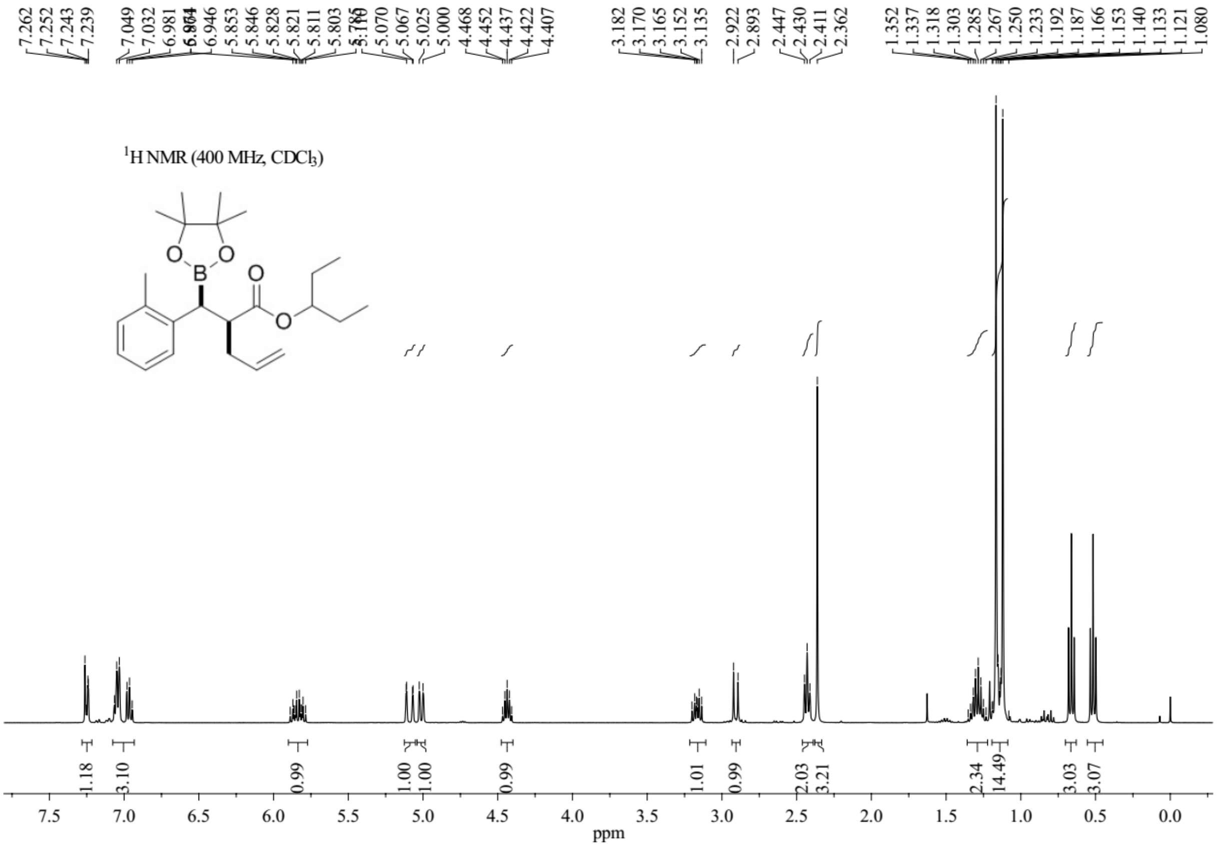

Supplementary Figure 102. **^{13}C NMR spectrum for 8f**

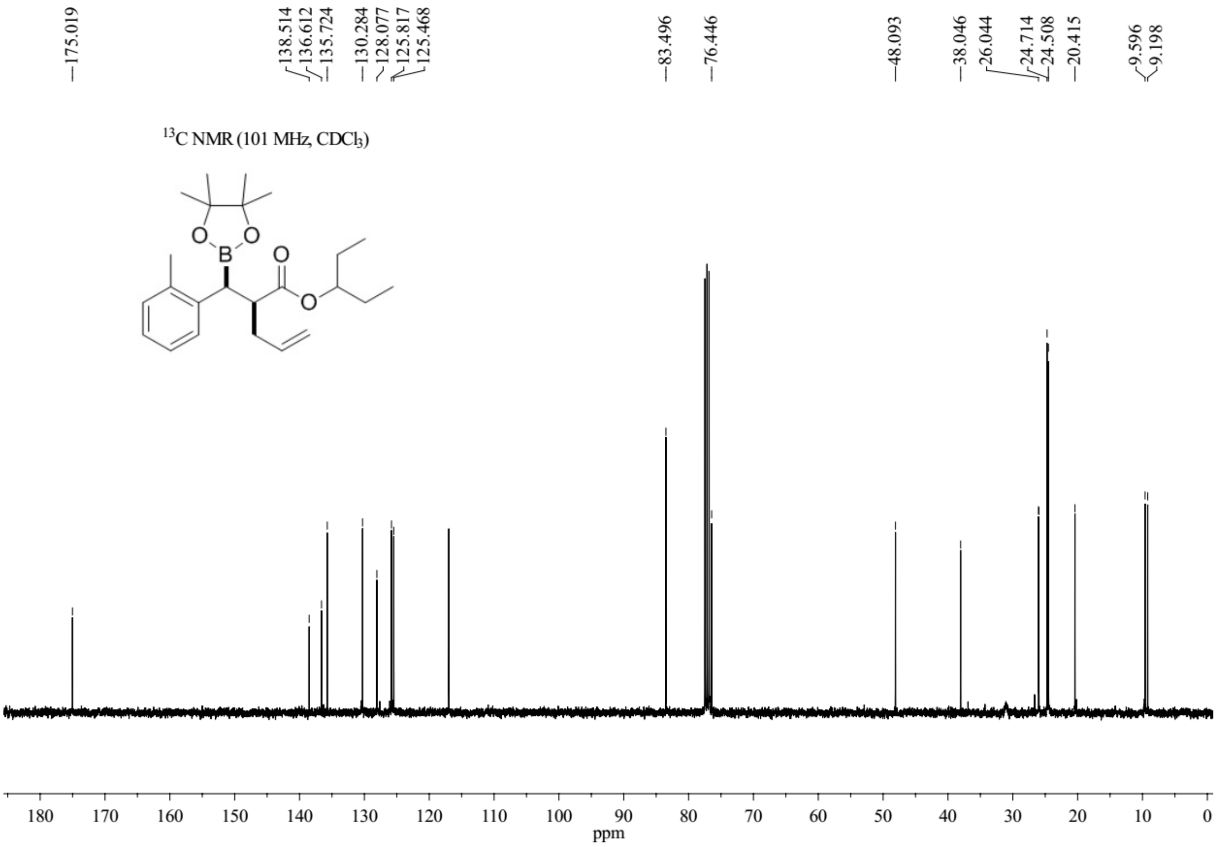

Supplementary Figure 103. ^{1}H NMR spectrum for *anti-tert*-Butyl-2-(naphthalen-1-yl(4,4,5,5-tetramethyl-1,3,2-dioxaborolan-2-yl)methyl)pent-4-enoate (7g)

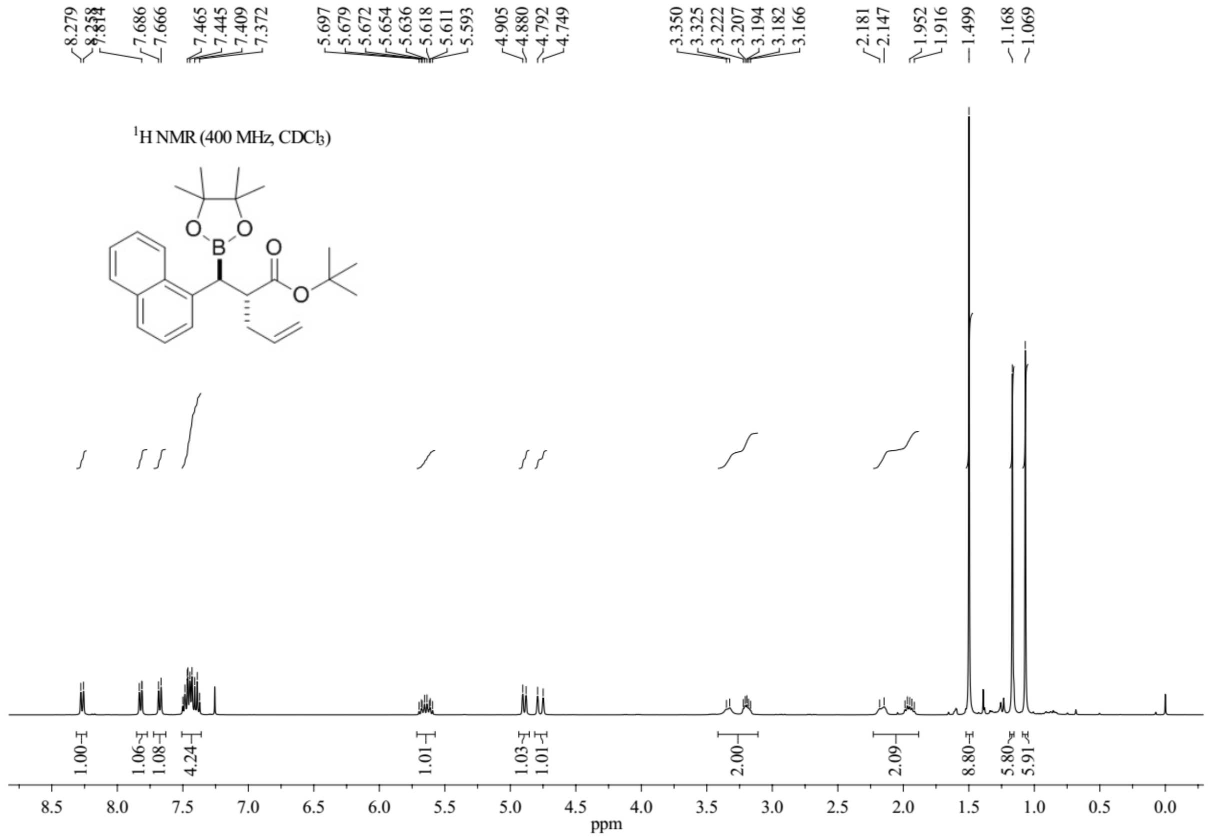

Supplementary Figure 104. ^{13}C NMR spectrum for 7g

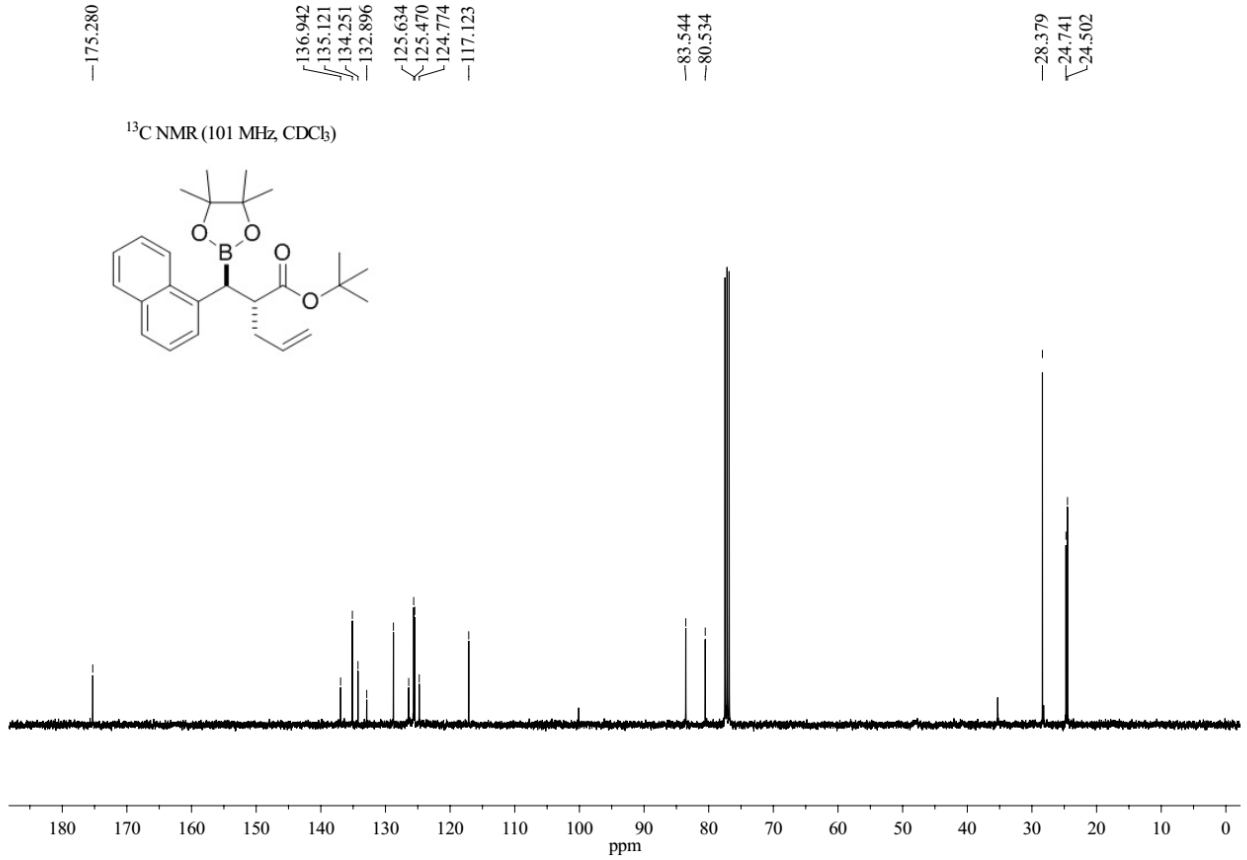

Supplementary Figure 105. ^{1}H NMR spectrum for *syn*-Pentan-3-yl-2-(naphthalen-1-yl(4,4,5,5-tetramethyl-1,3,2-dioxaborolan-2-yl)methyl)pent-4-enoate (8g)

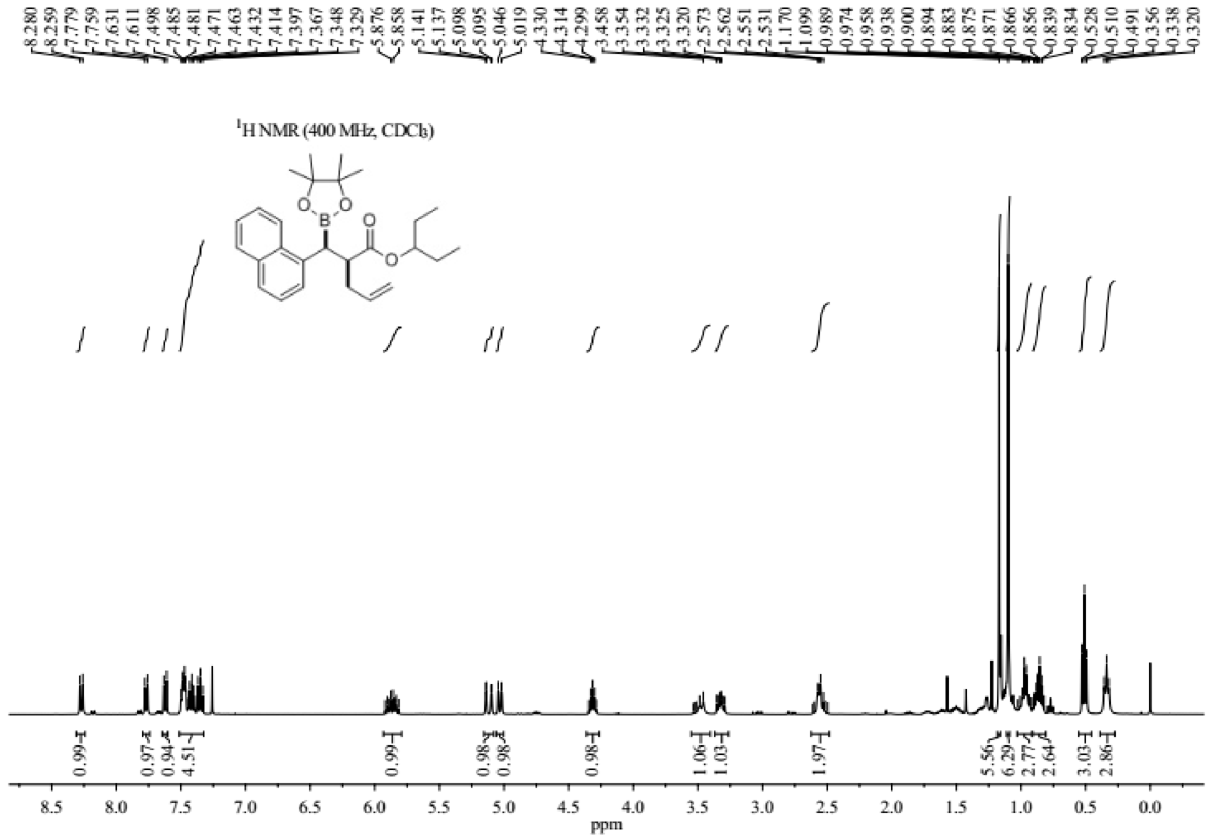

Supplementary Figure 106. ^{13}C NMR spectrum for 8g

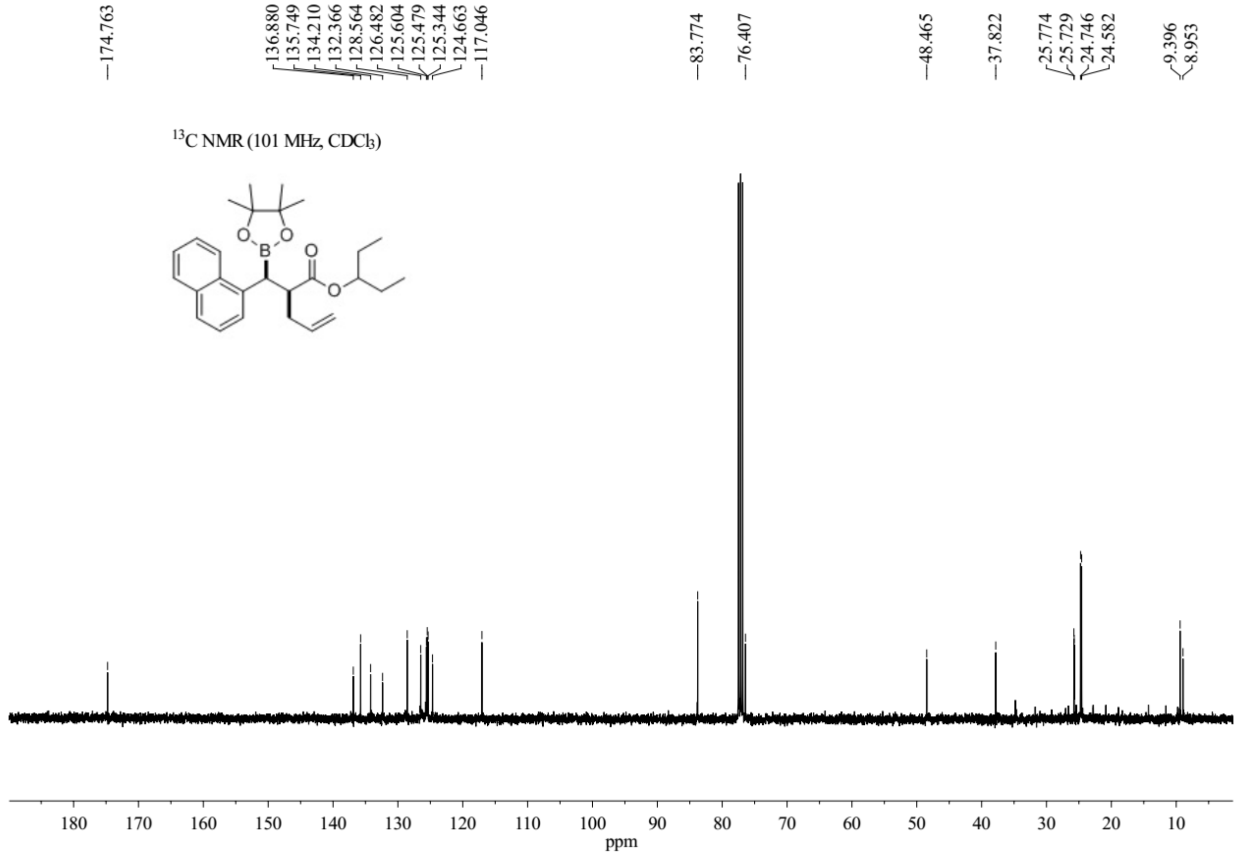

Supplementary Figure 107. ^{1}H NMR spectrum for *anti*-*tert*-Butyl-2-((4,4,5,5-tetramethyl-1,3,2-dioxaborolan-2-yl)(thiophen-2-yl)methyl)pent-4-enoate (7h)

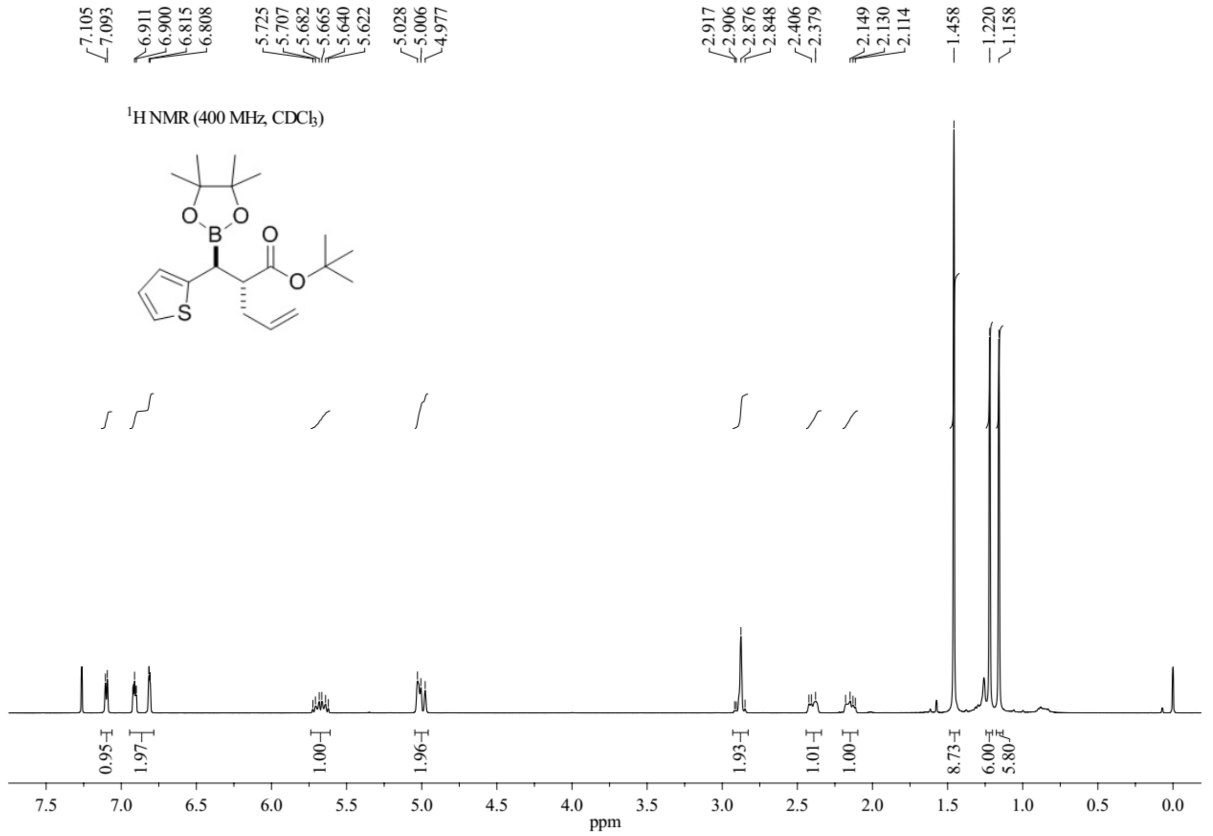

Supplementary Figure 108. ^{13}C NMR spectrum for 7h

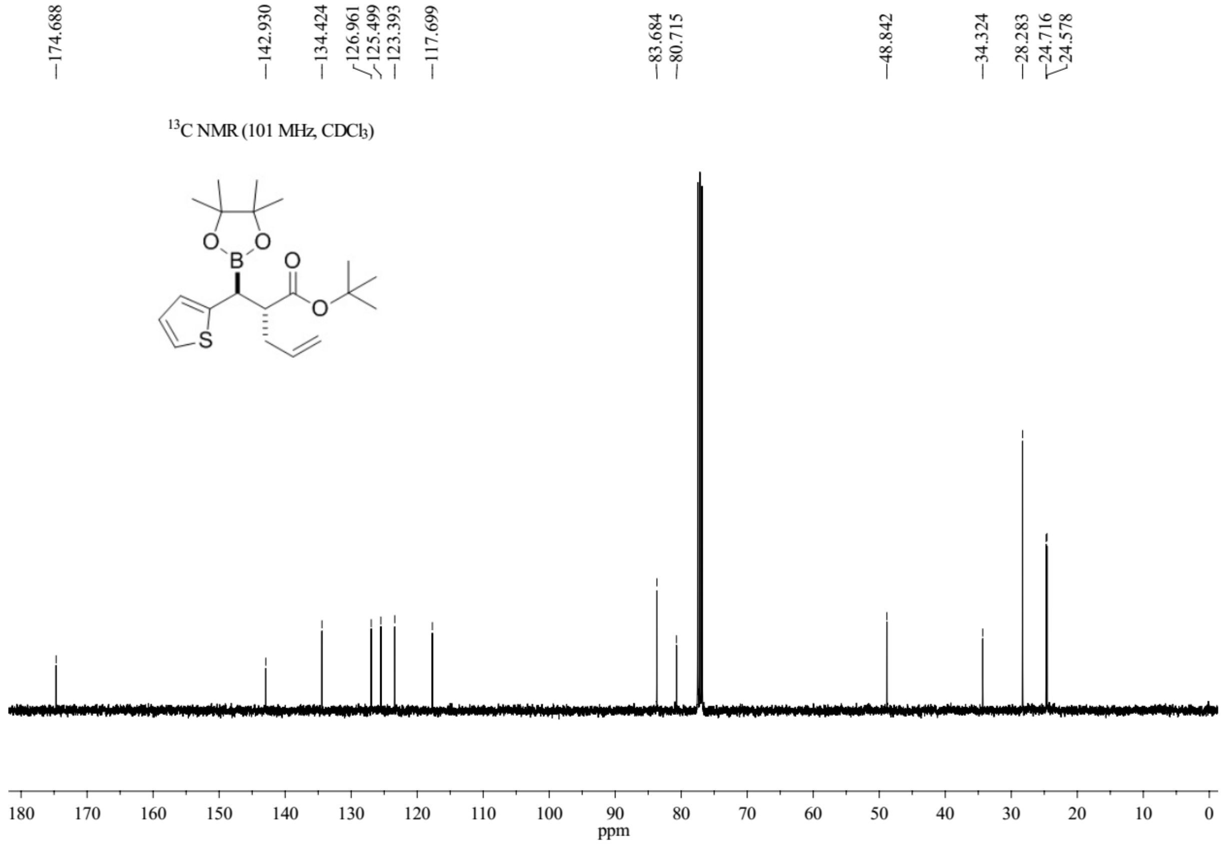

Supplementary Figure 109. ^{1}H NMR spectrum for *syn*-Pentan-3-yl-2-((4,4,5,5-tetramethyl-1,3,2-dioxaborolan-2-yl)(thiophen-2-yl)methyl)pent-4-enoate (8h)

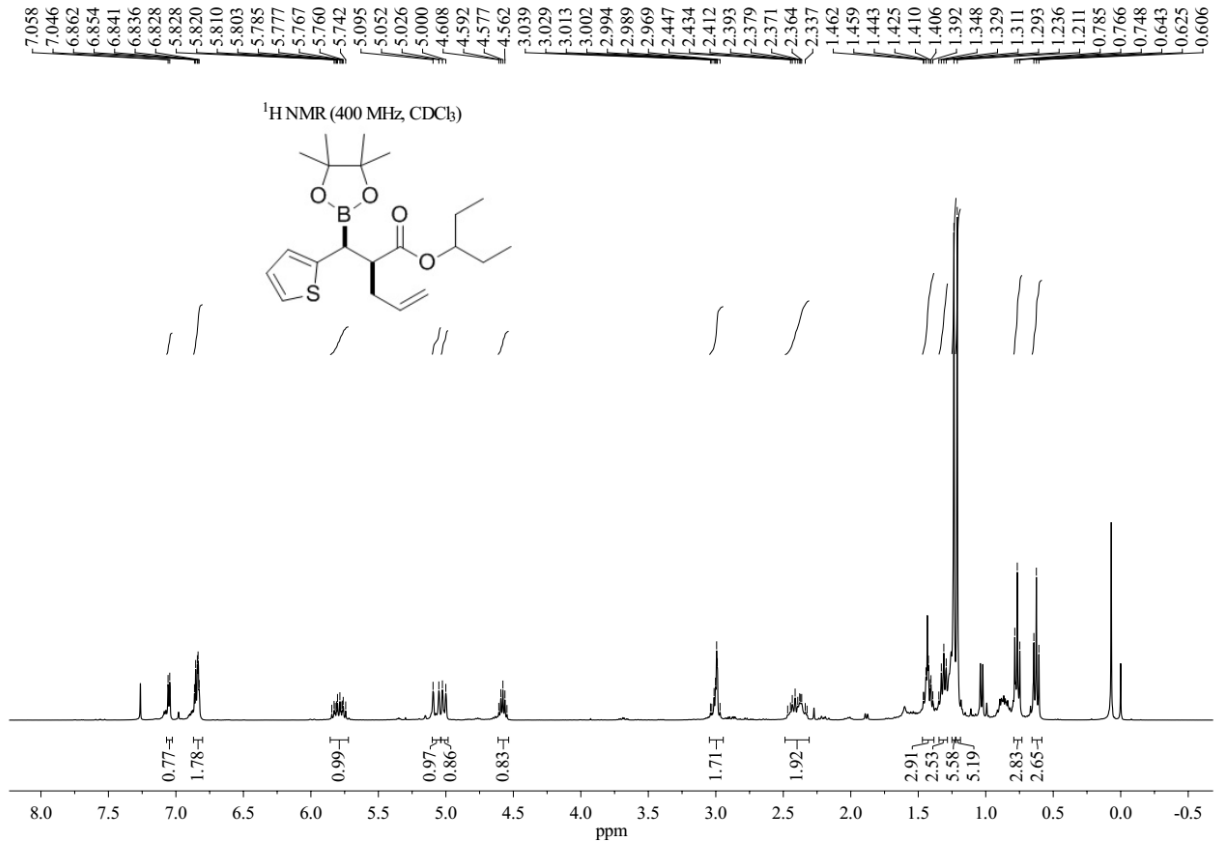

Supplementary Figure 110. ^{13}C NMR spectrum for 8h

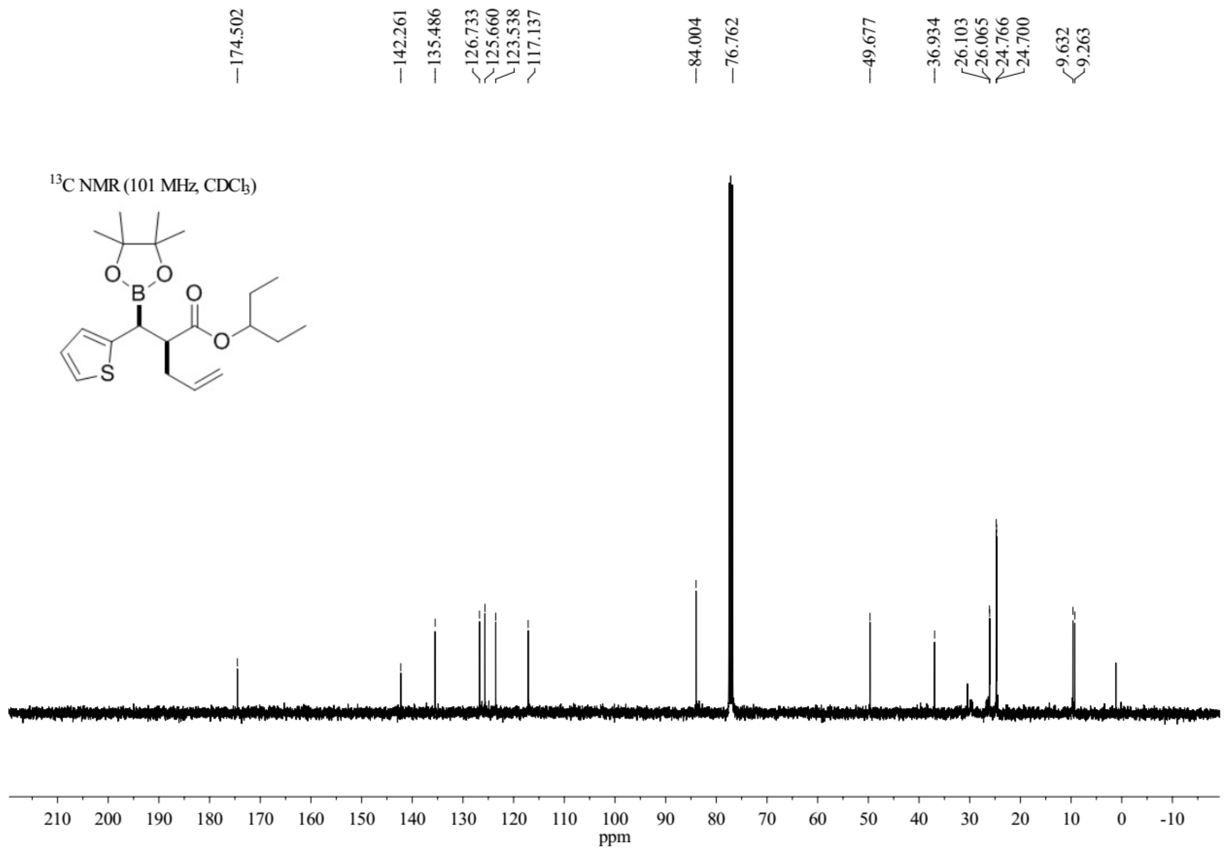

Supplementary Figure 111. ^{1}**H NMR spectrum for** ***anti-tert*****-Butyl-2-(1-(4,4,5,5-tetramethyl-1,3,2-dioxaborolan-2-yl)ethyl)pent-4-enoate (7i)**

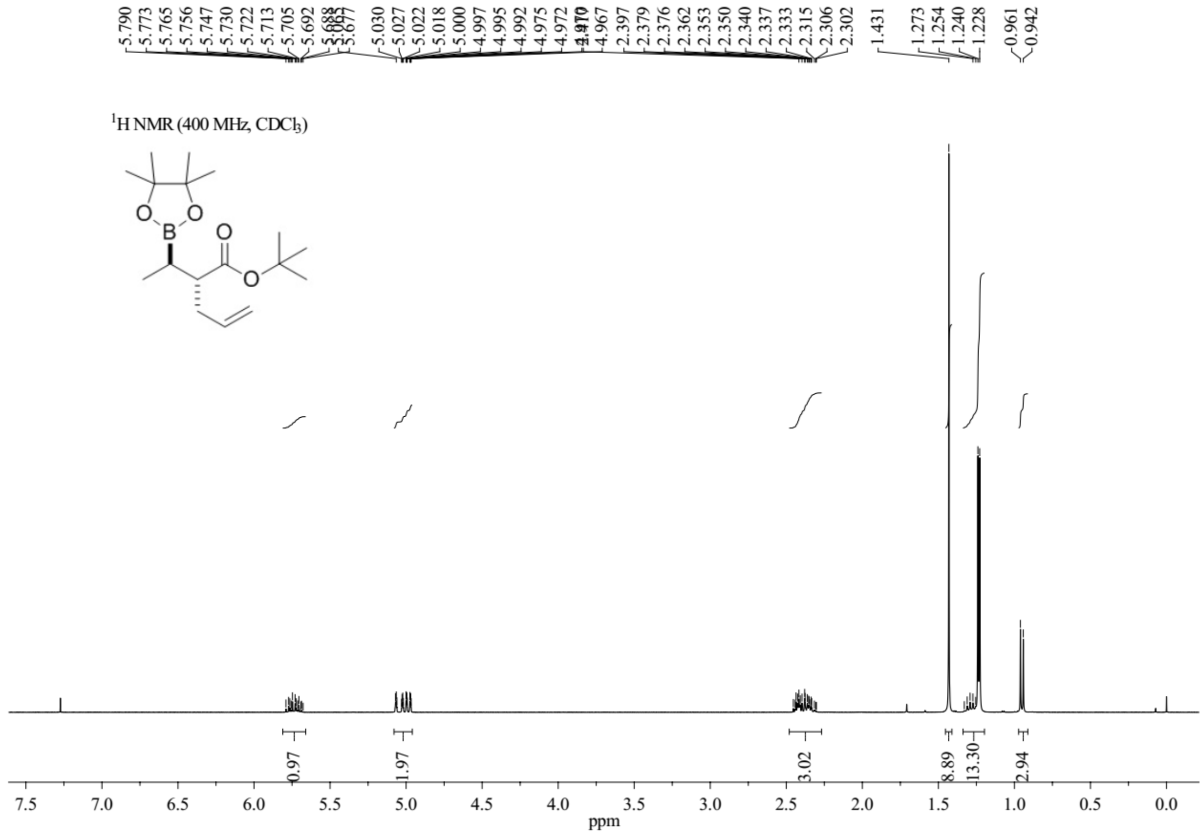

Supplementary Figure 112. ^{13}**C NMR spectrum for 7i**

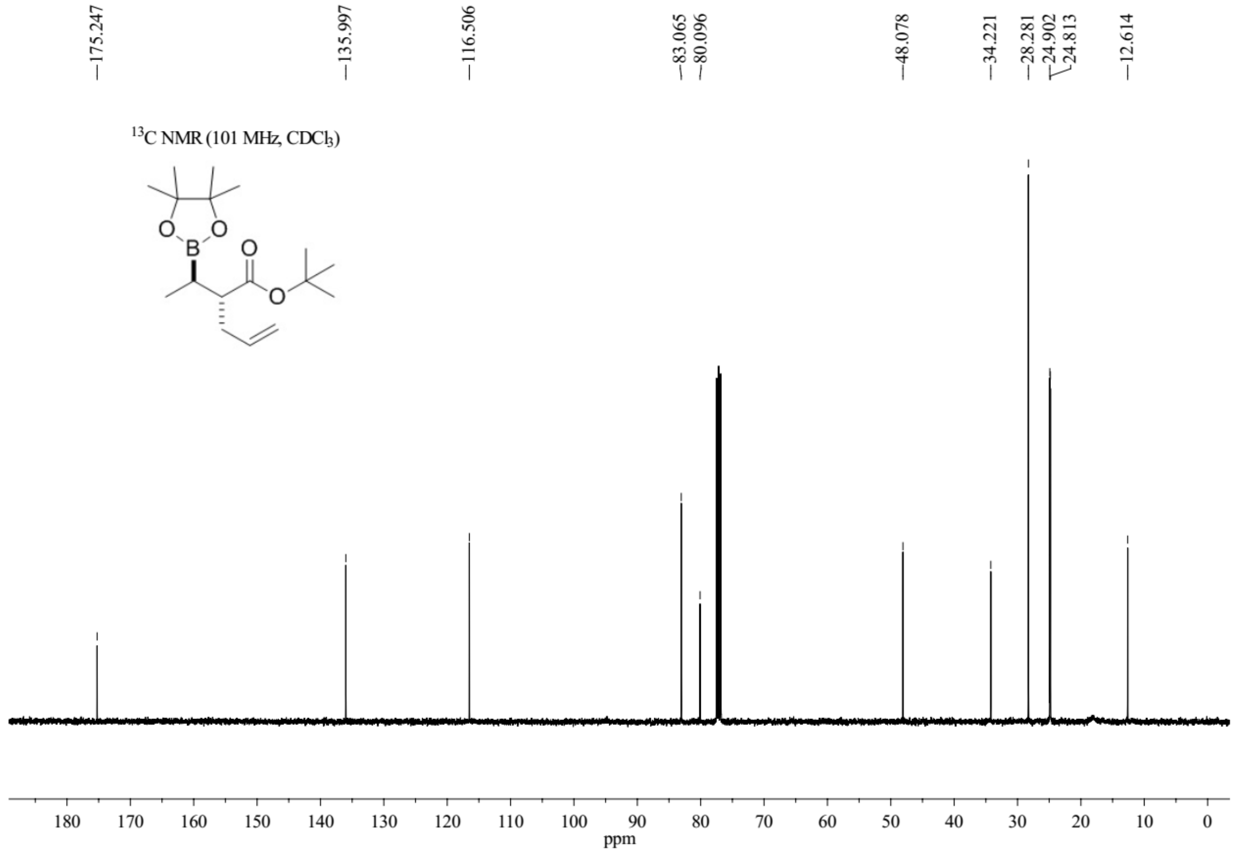

Supplementary Figure 113. ^{1}H NMR spectrum for *syn*-Pentan-3-yl-2-(1-(4,4,5,5-tetramethyl-1,3,2-dioxaborolan-2-yl)ethyl)pent-4-enoate(8i)

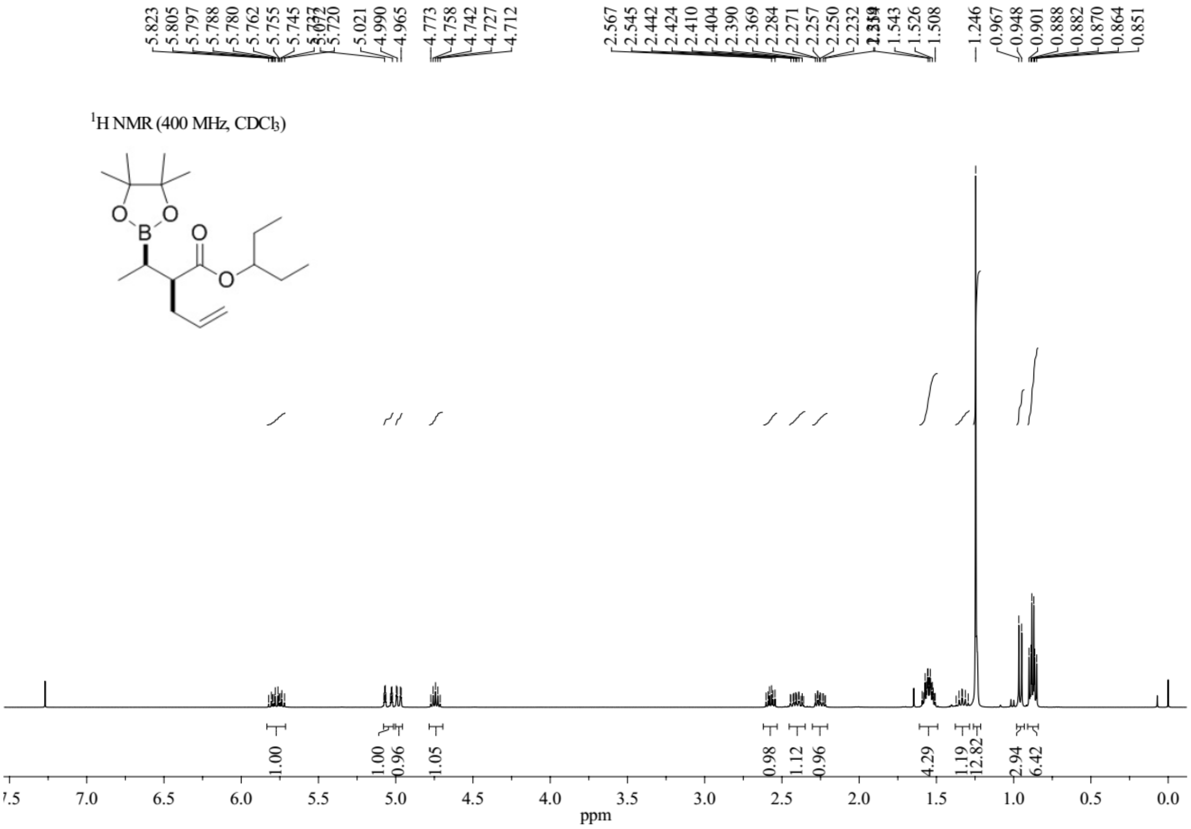

Supplementary Figure 114. ^{13}C NMR spectrum for 8i

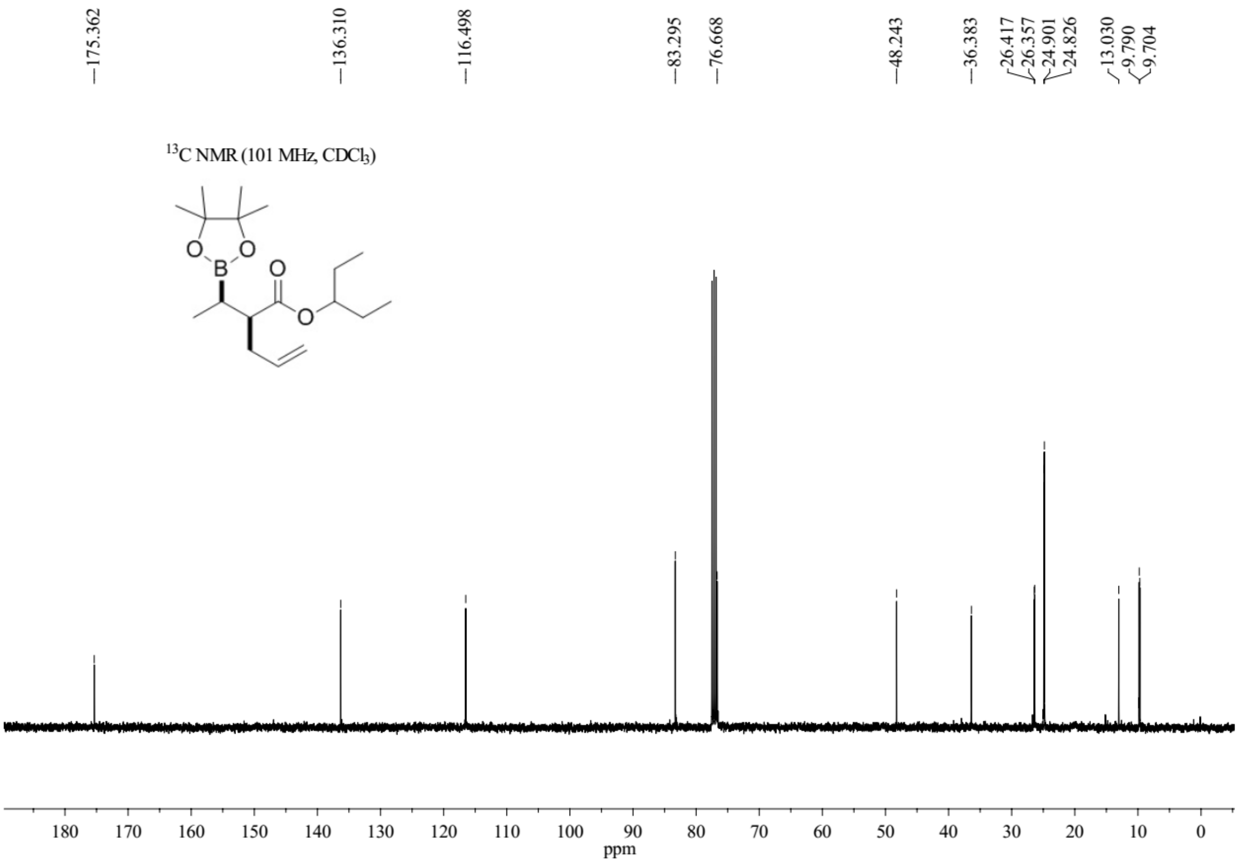

Supplementary Figure 115. ^{1}H NMR spectrum for *anti*-*tert*-Butyl-2-allyl-3-(4,4,5,5-tetramethyl-1,3,2-dioxaborolan-2-yl)hexanoate (7j)

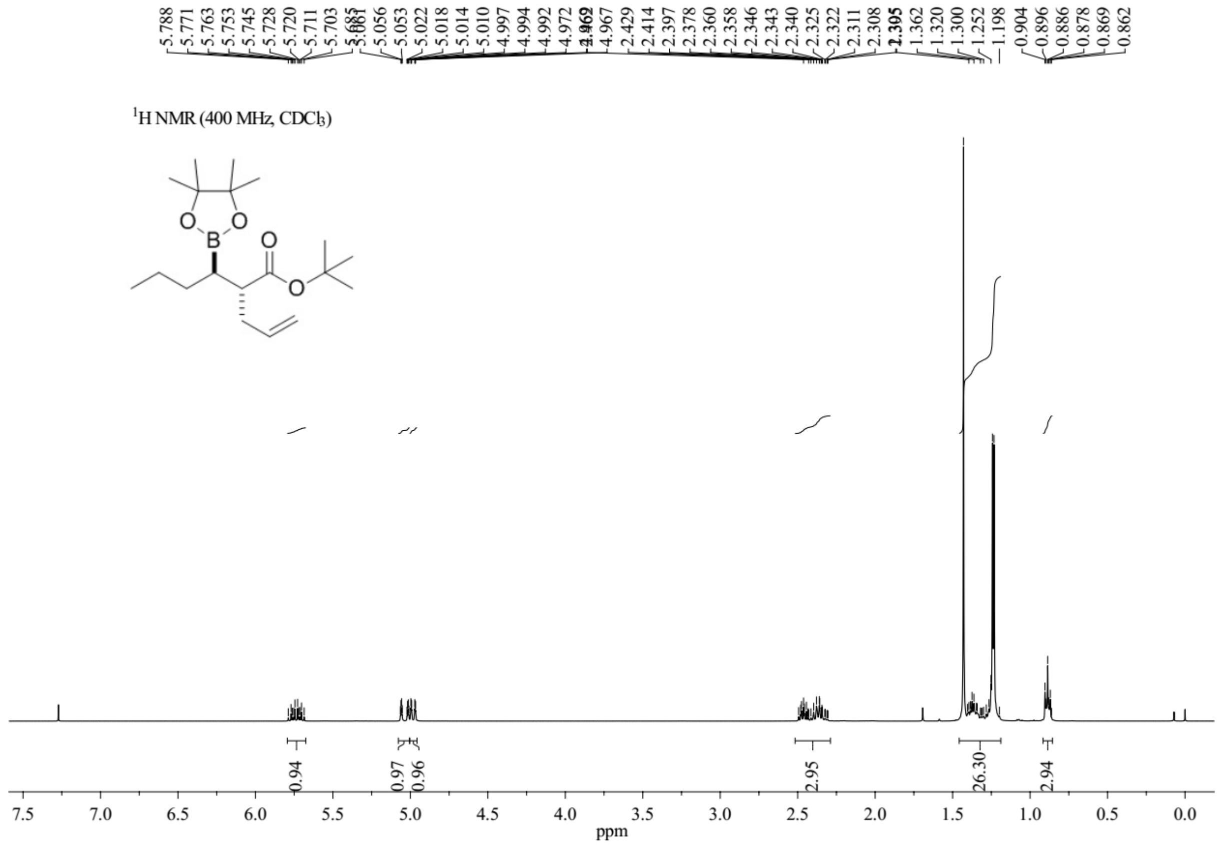

Supplementary Figure 116. ^{13}C NMR spectrum for 7j

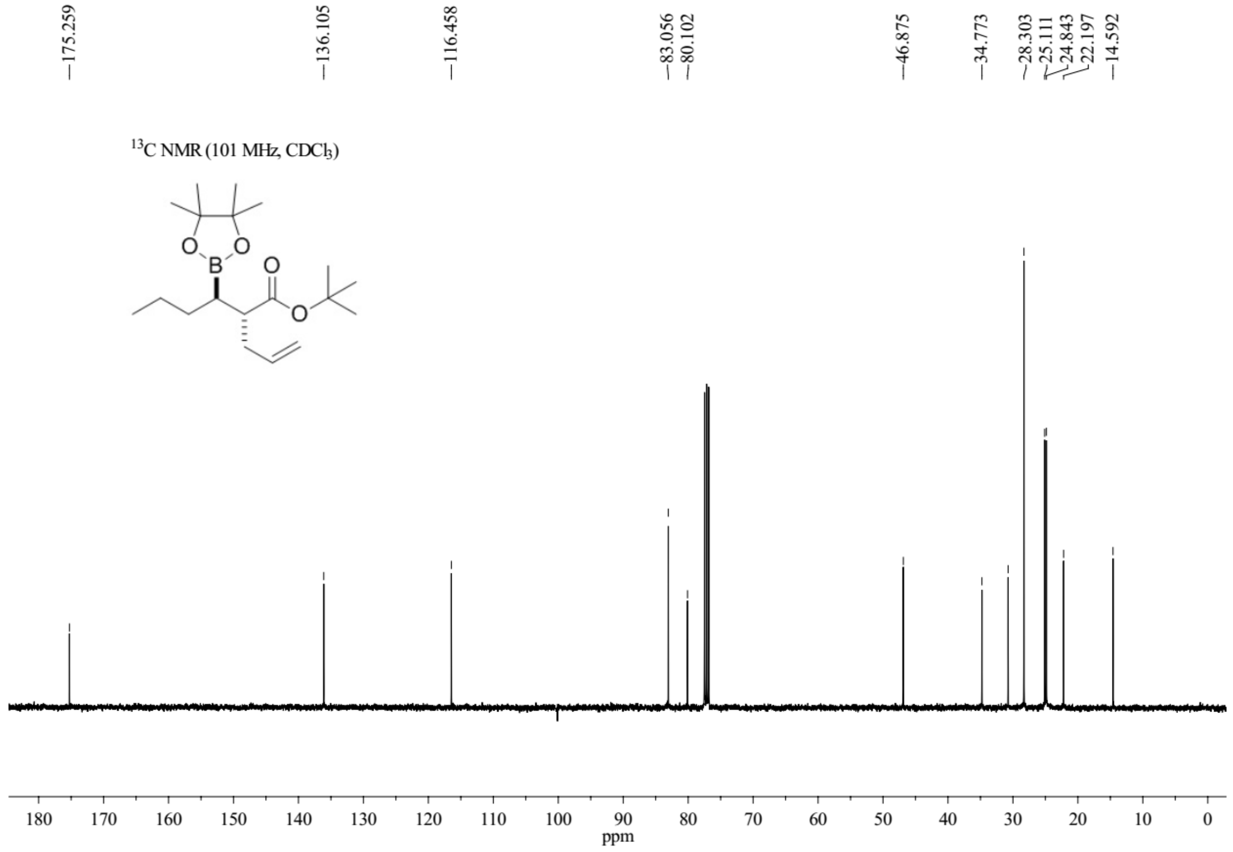

Supplementary Figure 117. ^{1}H NMR spectrum for *syn*-Pentan-3-yl 2-allyl-3-(4,4,5,5-tetramethyl-1,3,2-dioxaborolan-2-yl)hexanoate (8j)

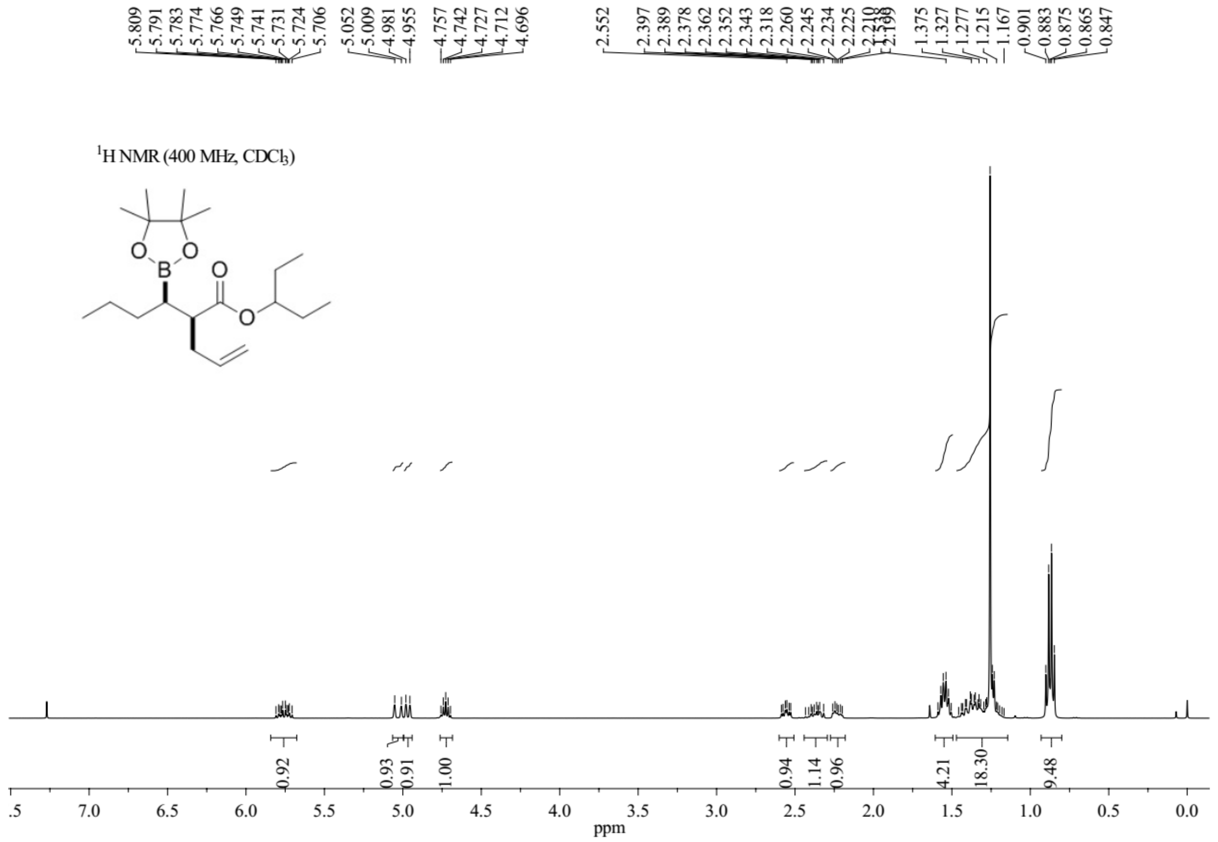

Supplementary Figure 118. ^{13}C NMR spectrum for 8j

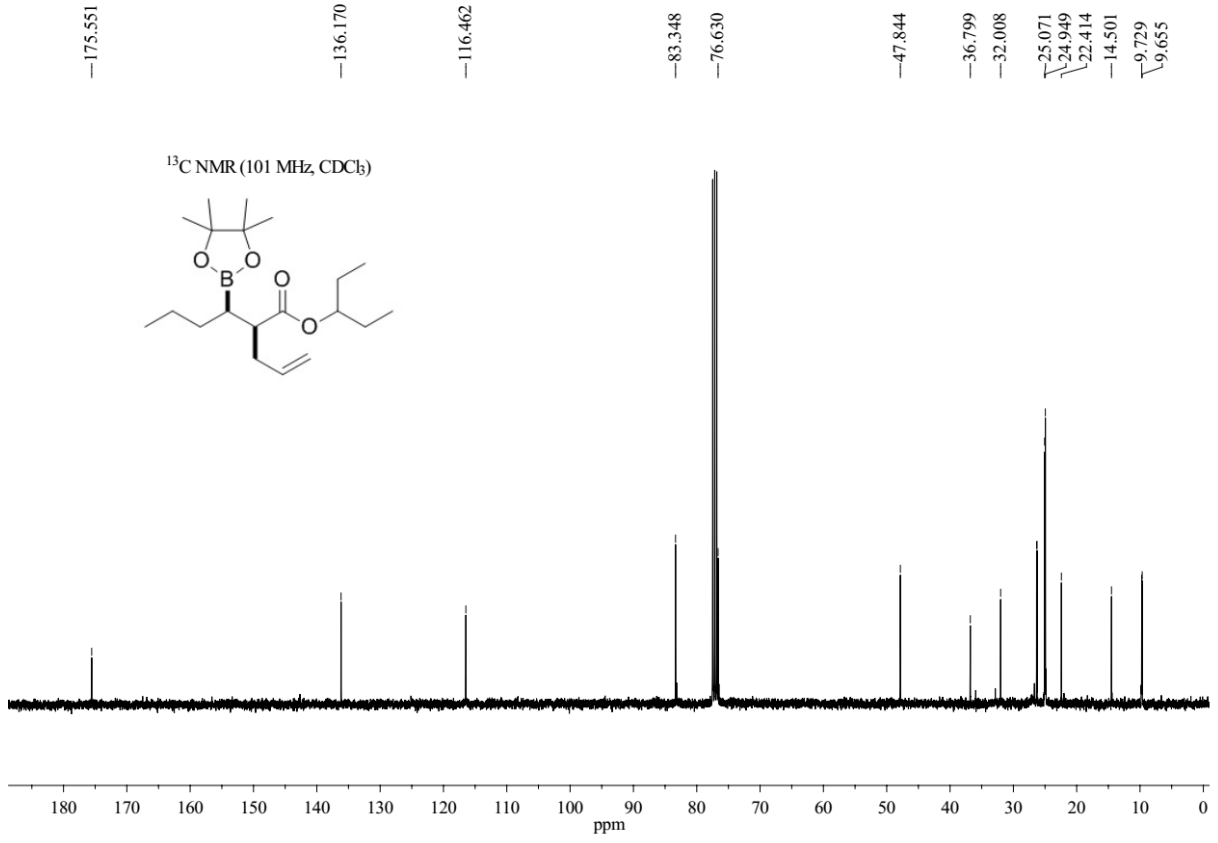

Supplementary Figure 119. ^{1}H NMR spectrum for *anti-tert*-Butyl-2-(3-phenyl-1-(4,4,5,5-tetramethyl-1,3,2-dioxaborolan-2-yl)propyl)pent-4-enoate (7k)

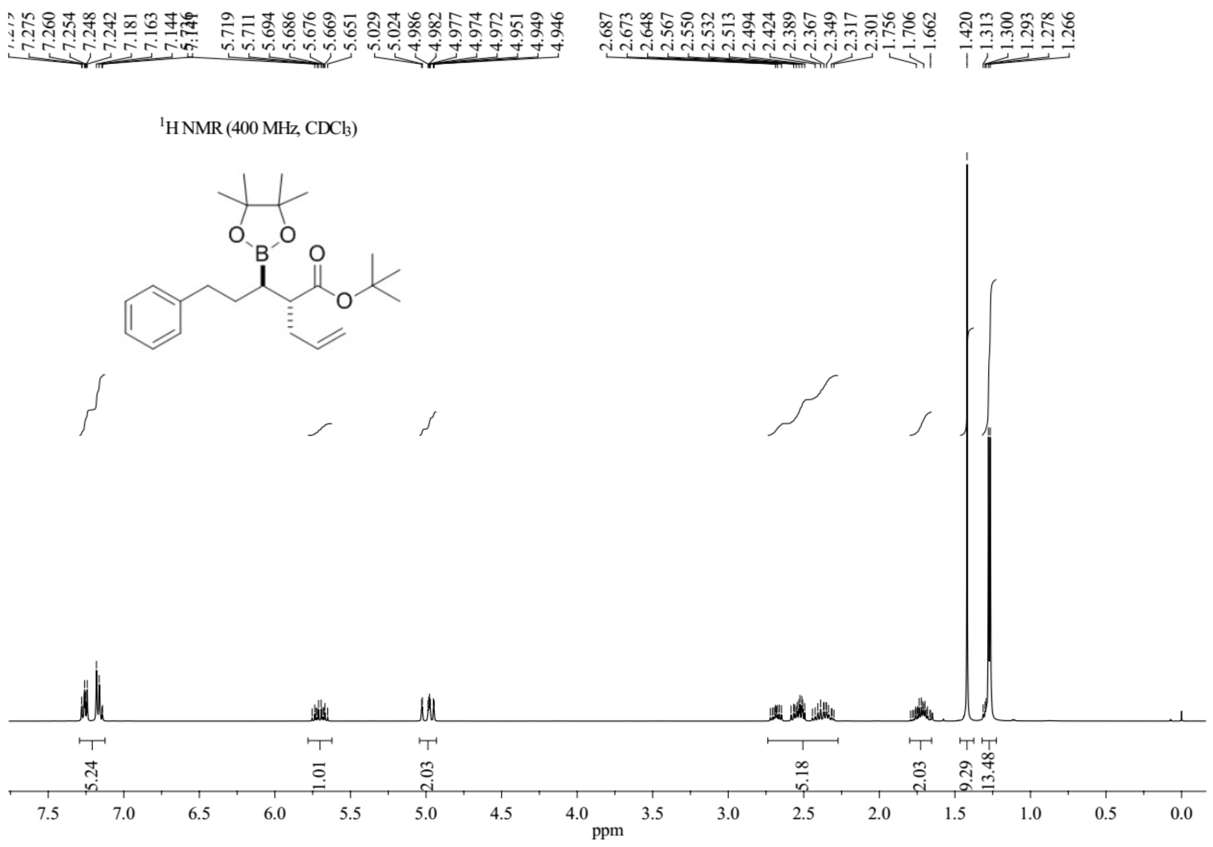

Supplementary Figure 120. ^{13}C NMR spectrum for 7k

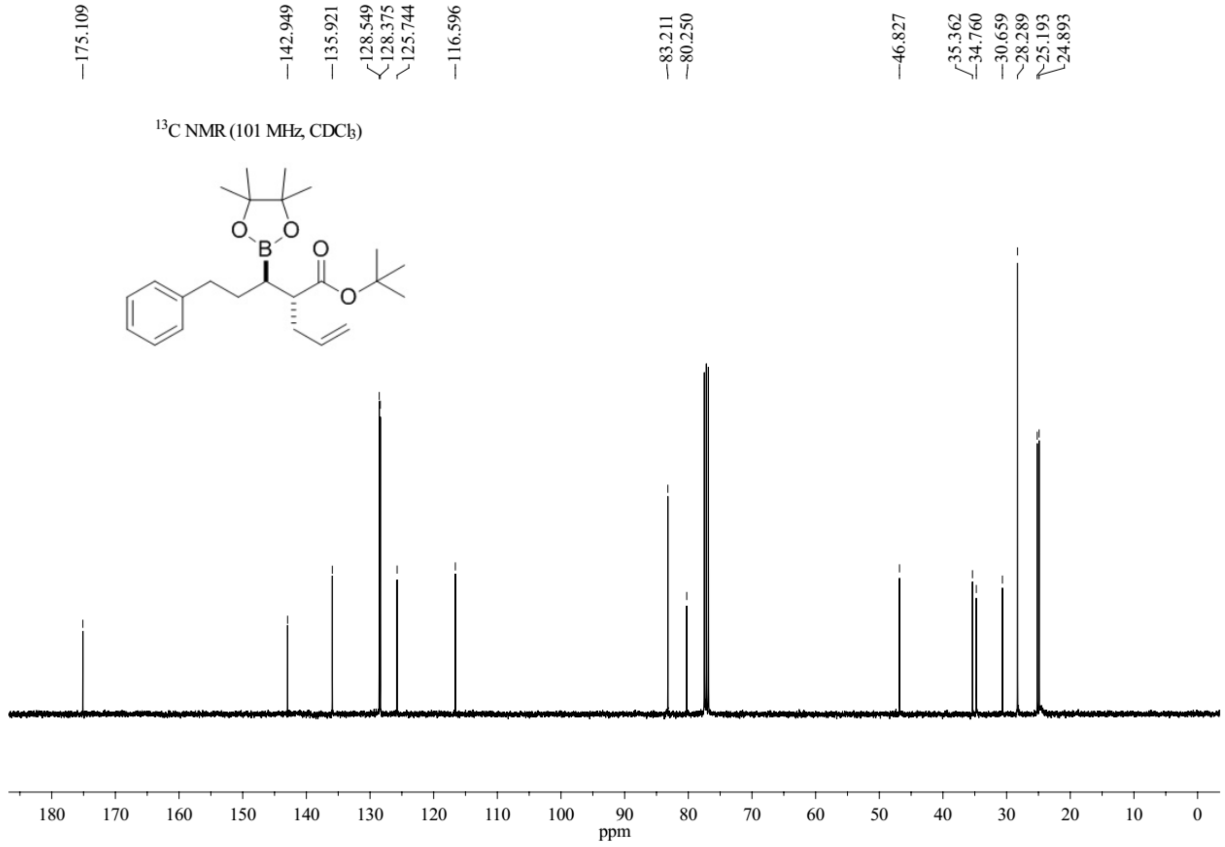

Supplementary Figure 121. ^{1}H NMR spectrum for *syn*-Pentan-3-yl-2-(3-phenyl-1-(4,4,5,5-tetramethyl-1,3,2-dioxaborolan-2-yl)propyl)pent-4-enoate (8k)

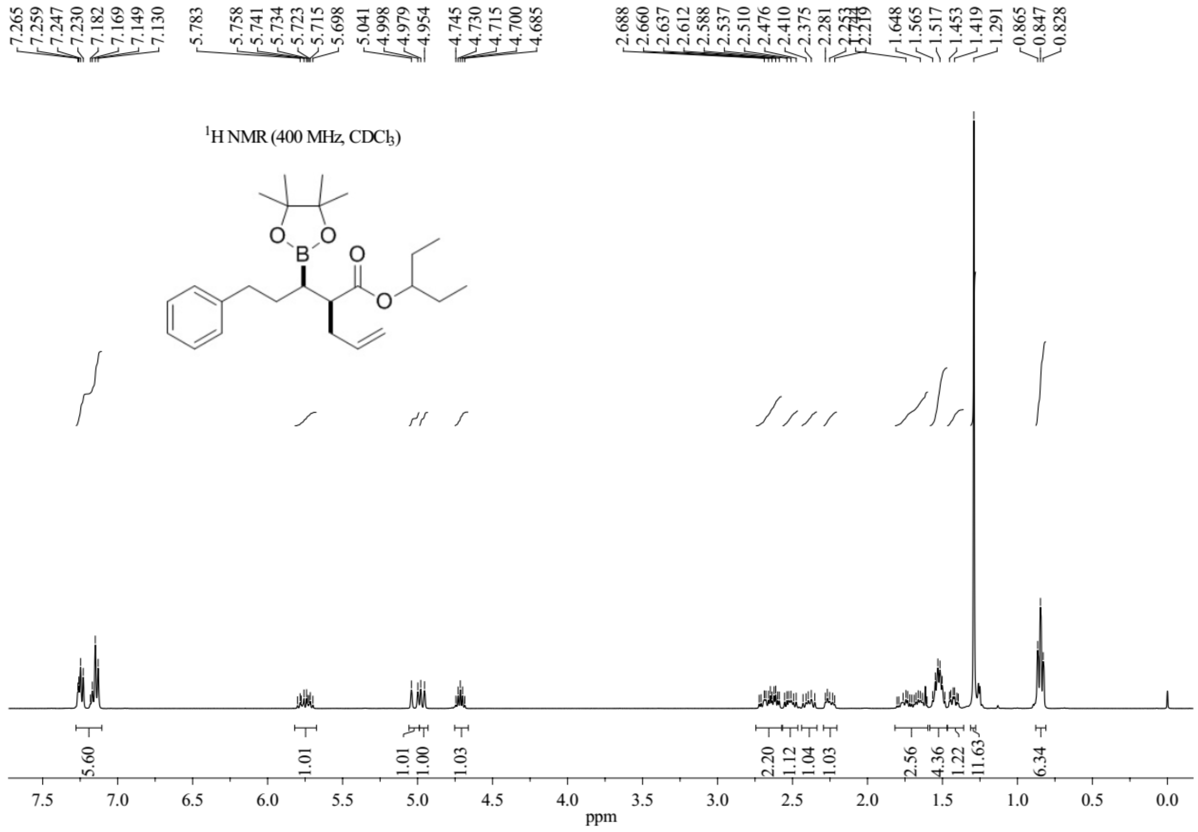

Supplementary Figure 122. ^{13}C NMR spectrum for 8k

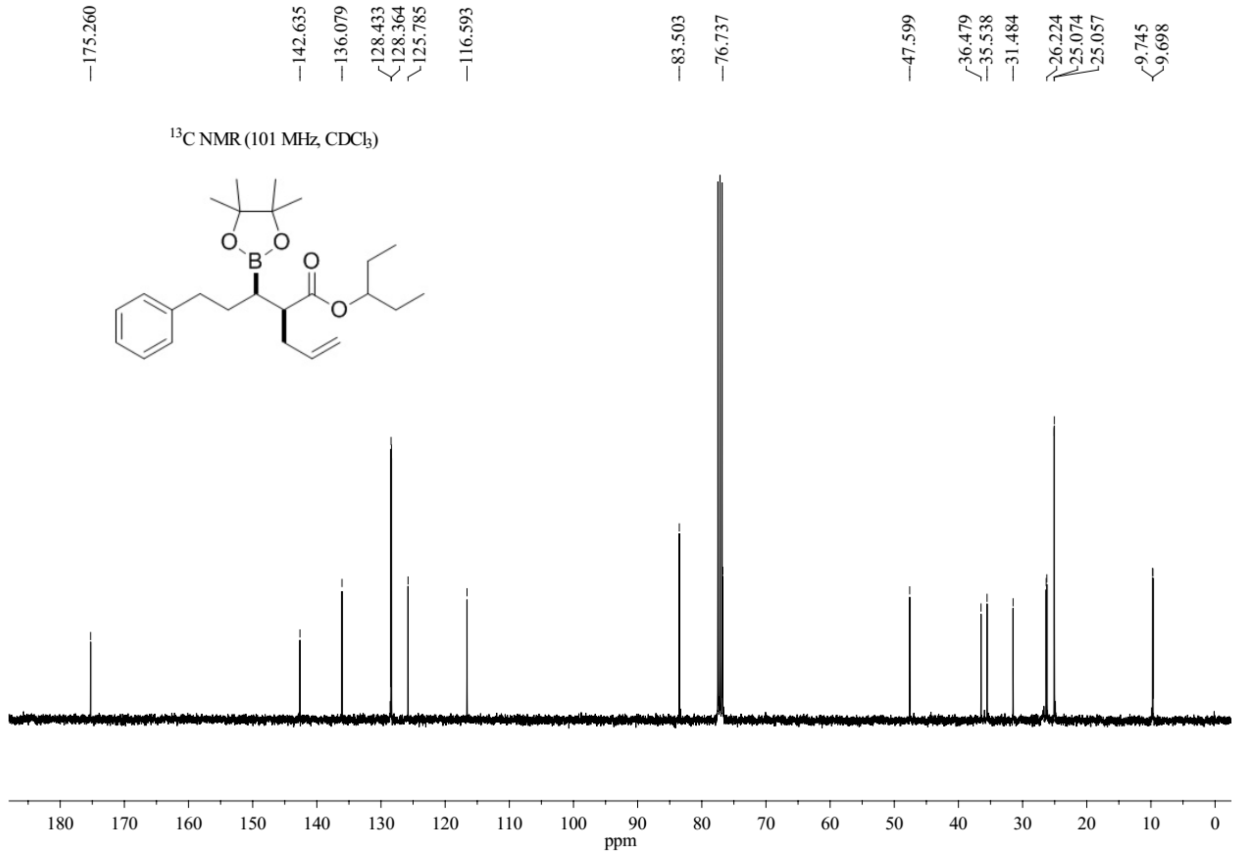

Supplementary Figure 123. ^{1}H NMR spectrum for *anti*-*tert*-Butyl-2-(2-methyl-1-(4,4,5,5-tetramethyl-1,3,2-dioxaborolan-2-yl)propyl)pent-4-enoate (7l)

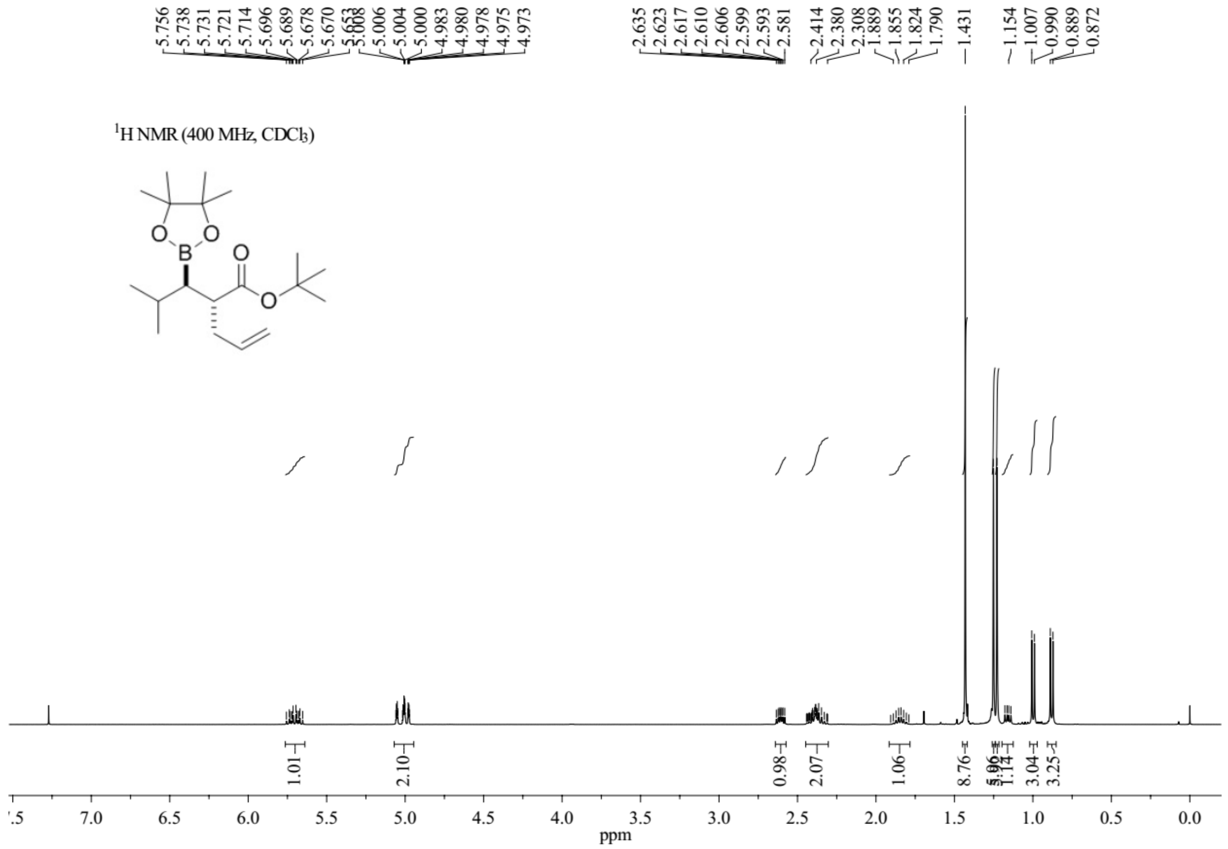

Supplementary Figure 124. ^{13}C NMR spectrum for 7l

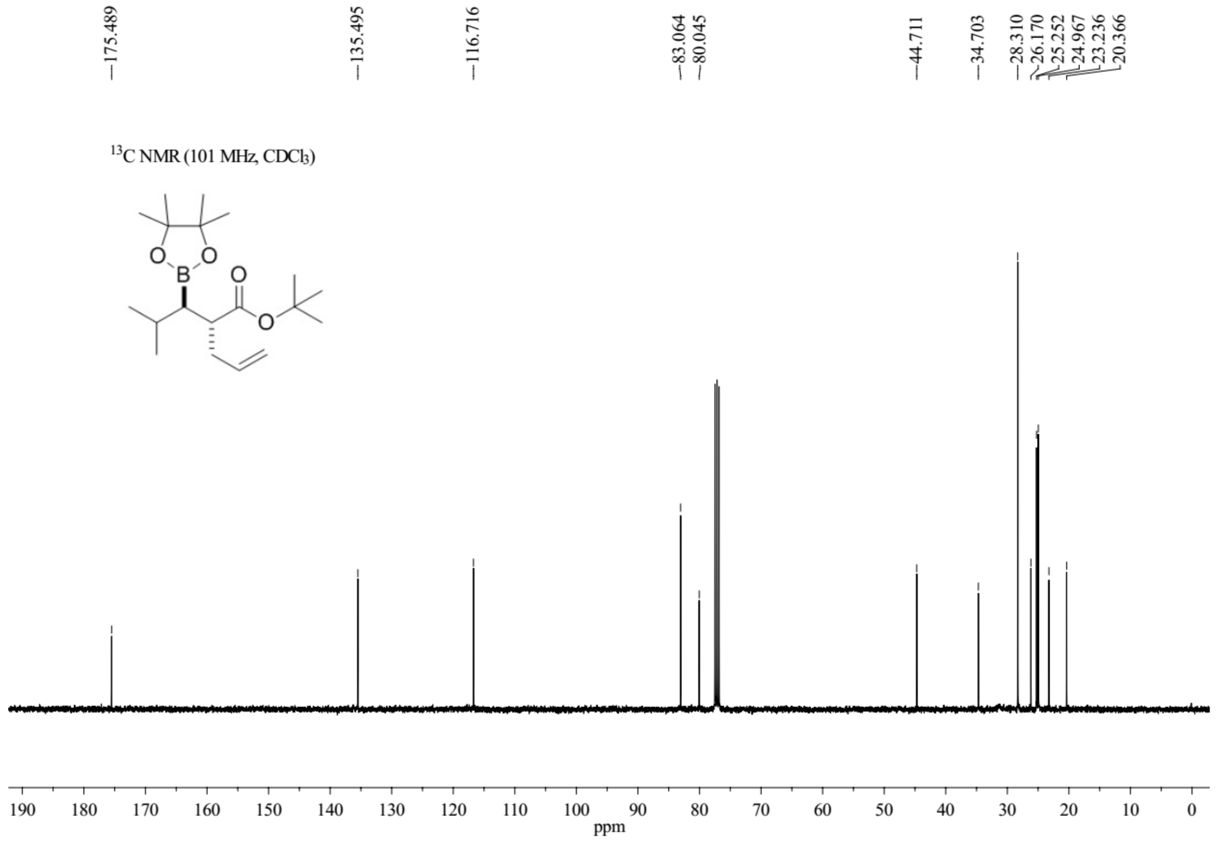

Supplementary Figure 125. ^{1}H NMR spectrum for *syn*-Pentan-3-yl-2-(2-methyl-1-(4,4,5,5-tetramethyl-1,3,2-dioxaborolan-2-yl)propyl)pent-4-enoate (8l)

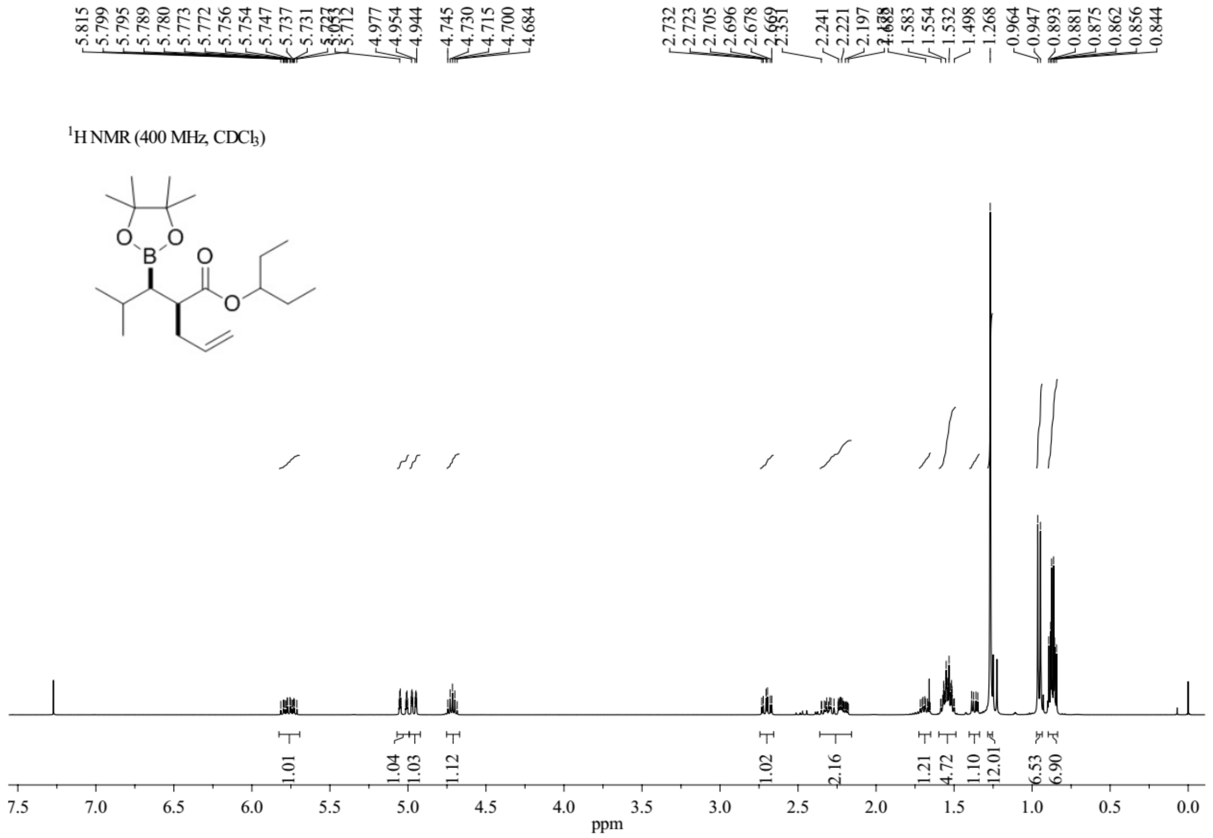

Supplementary Figure 126. ^{13}C NMR spectrum for 8l

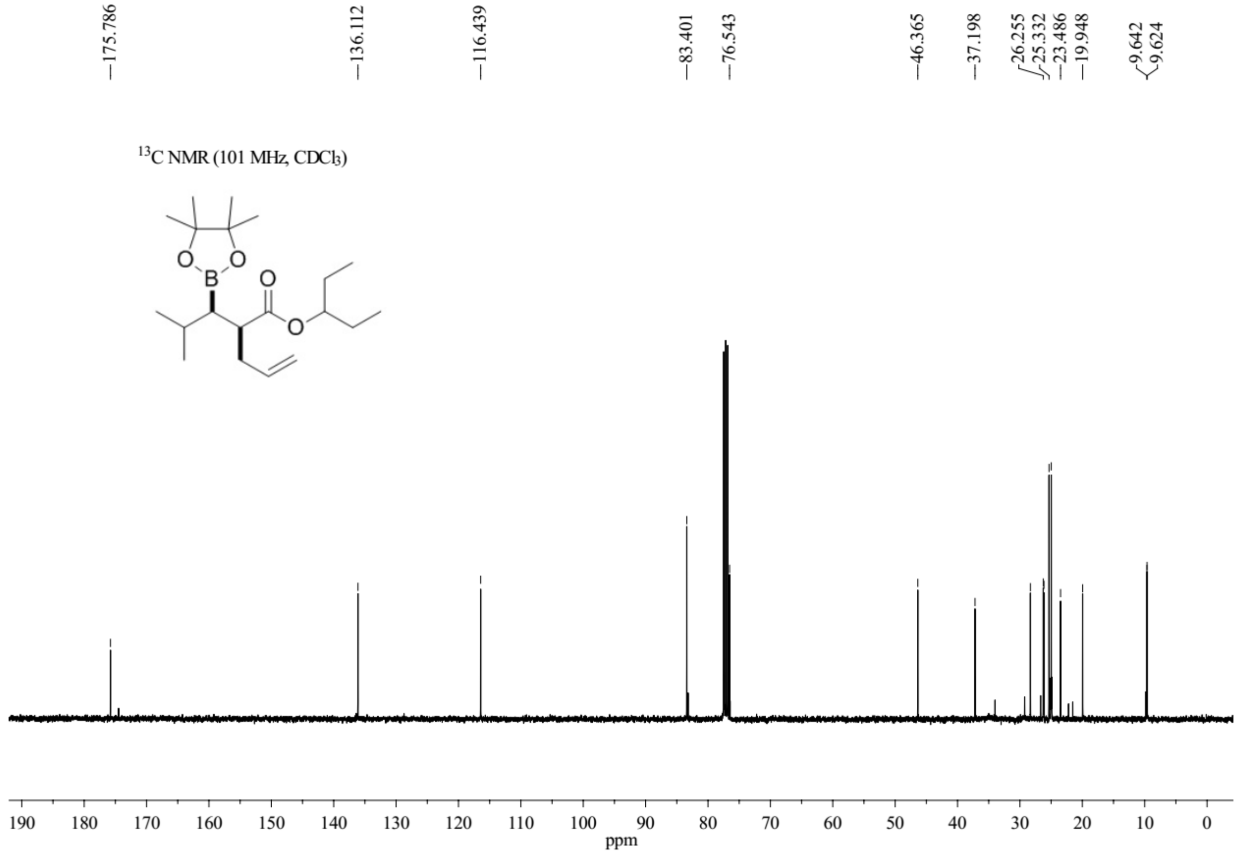

Supplementary Figure 127. ^{1}H NMR spectrum for *anti*-*tert*-Butyl-2-(cyclohexyl(4,4,5,5-tetramethyl-1,3,2-dioxaborolan-2-yl)methyl)pent-4-enoate (7m)

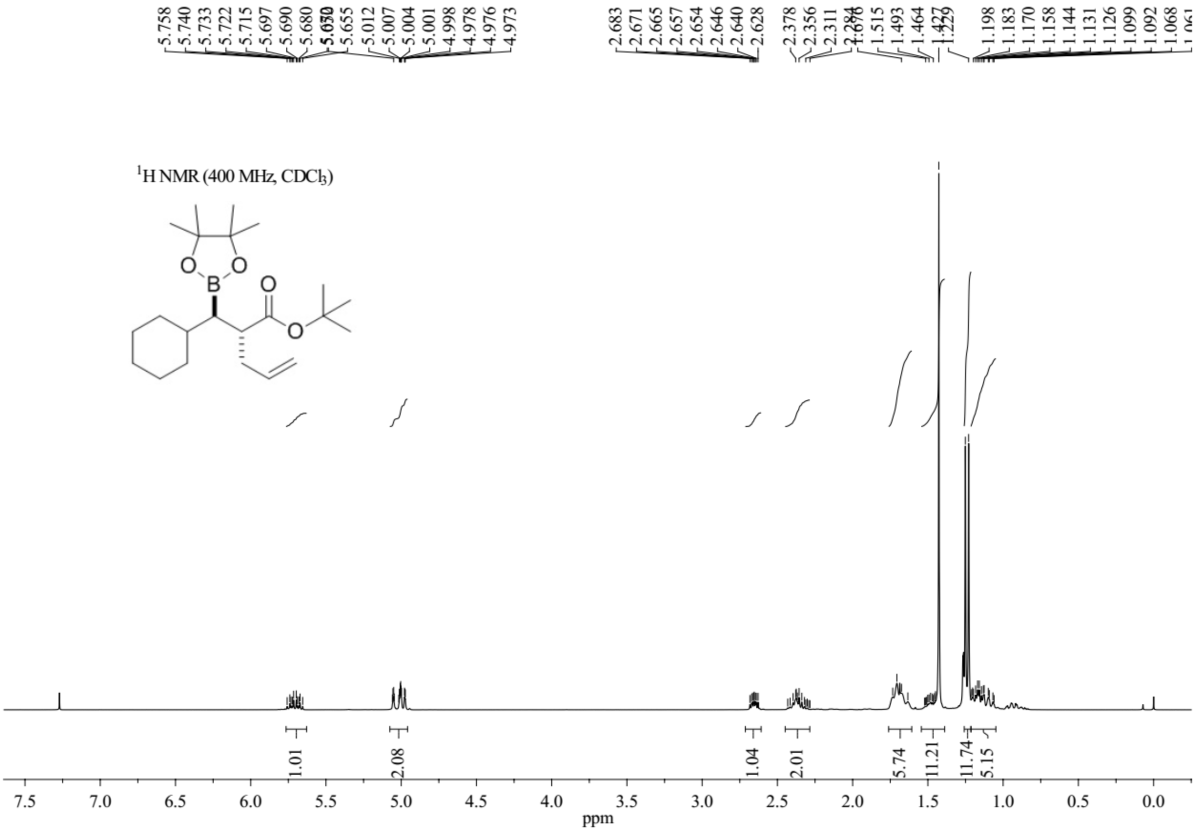

Supplementary Figure 128. ^{13}C NMR spectrum for 7m

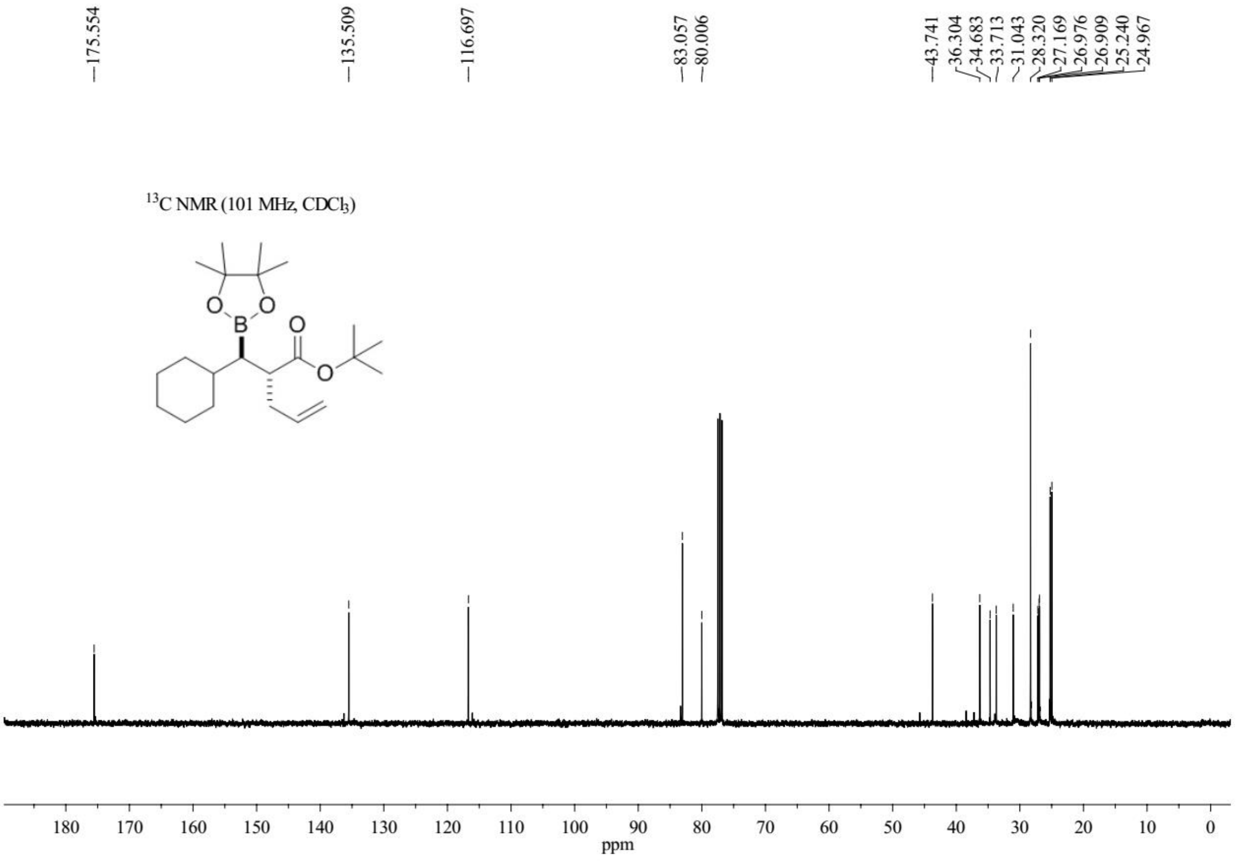

Supplementary Figure 129. ^{1}H NMR spectrum for *syn*-Pentan-3-yl-2-(cyclohexyl(4,4,5,5-tetramethyl-1,3,2-dioxaborolan-2-yl)methyl)pent-4-enoate (8m)

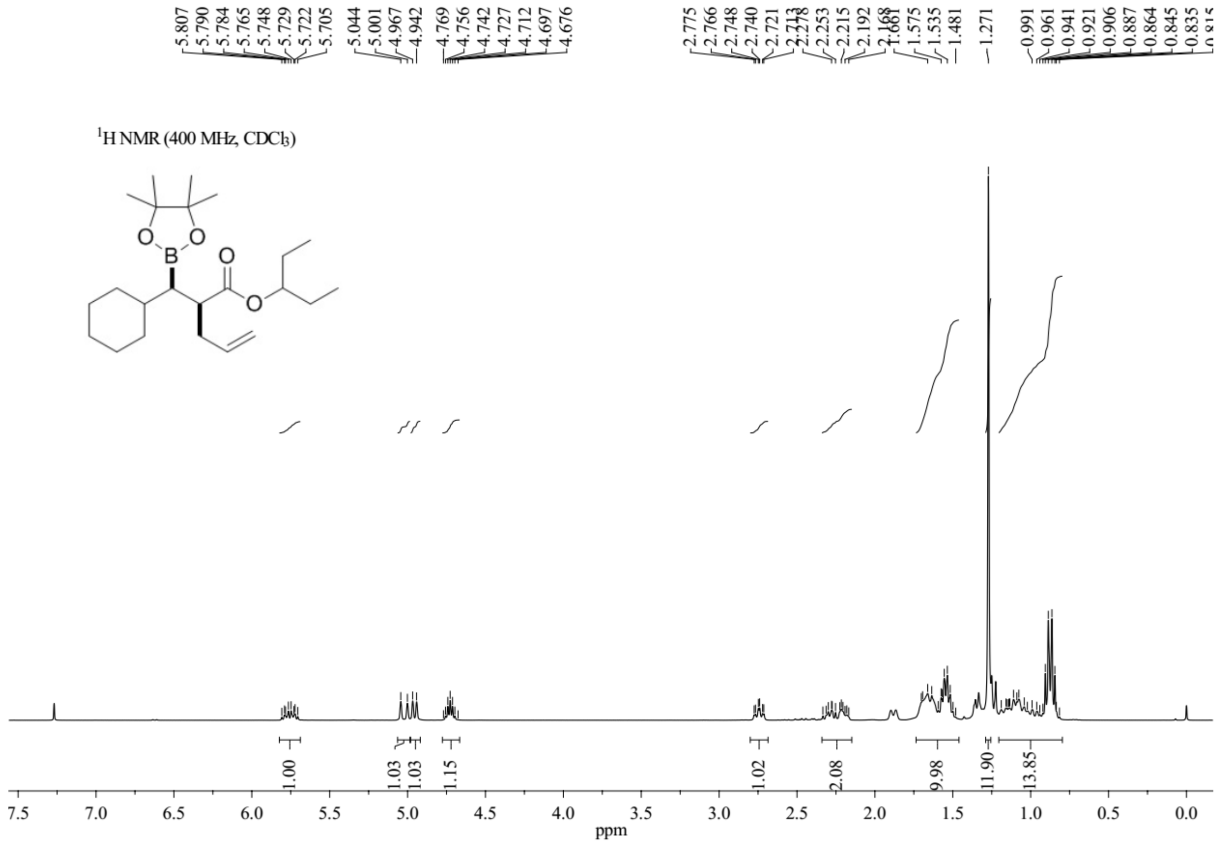

Supplementary Figure 130. ^{13}C NMR spectrum for 8m

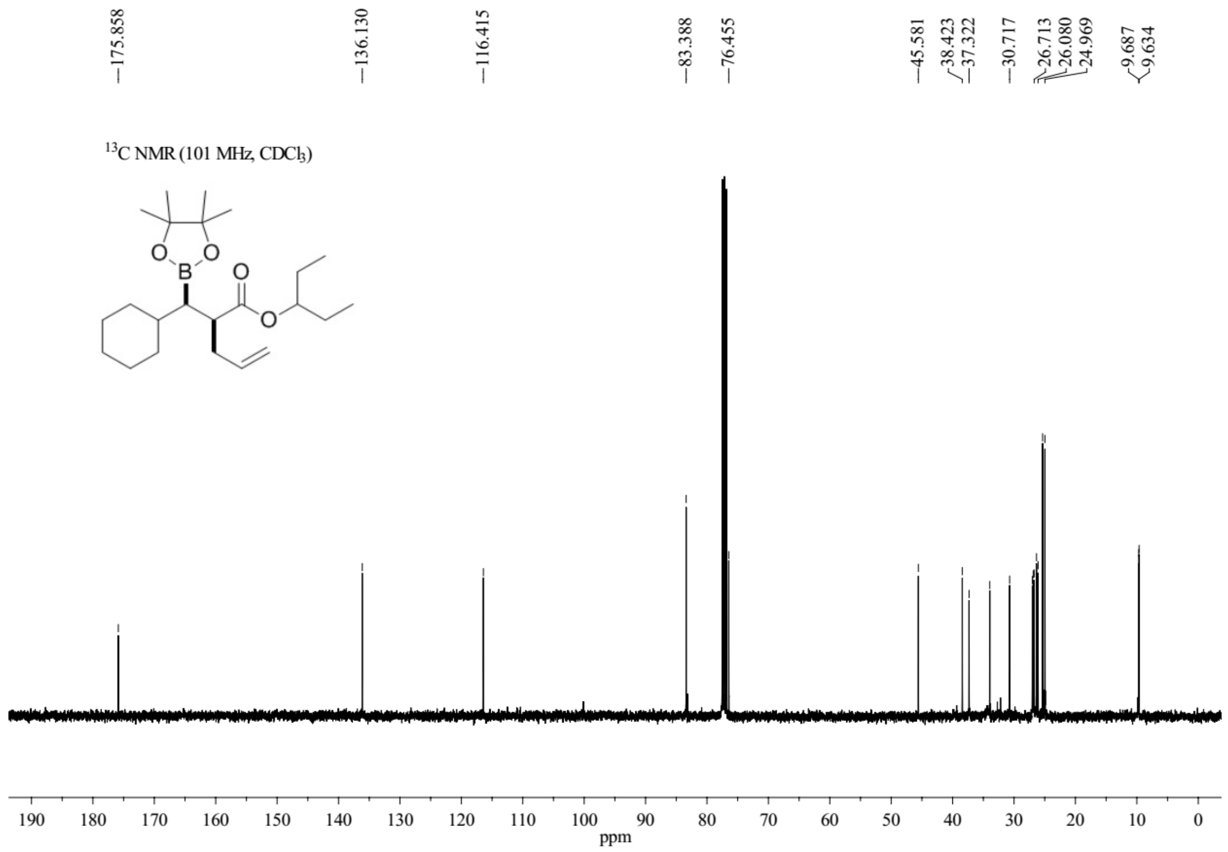

Supplementary Figure 131. ^{1}H NMR spectrum for *anti-tert*-Butyl-2-(cyclopropyl(4,4,5,5-tetramethyl-1,3,2-dioxaborolan-2-yl)methyl)pent-4-enoate (7n)

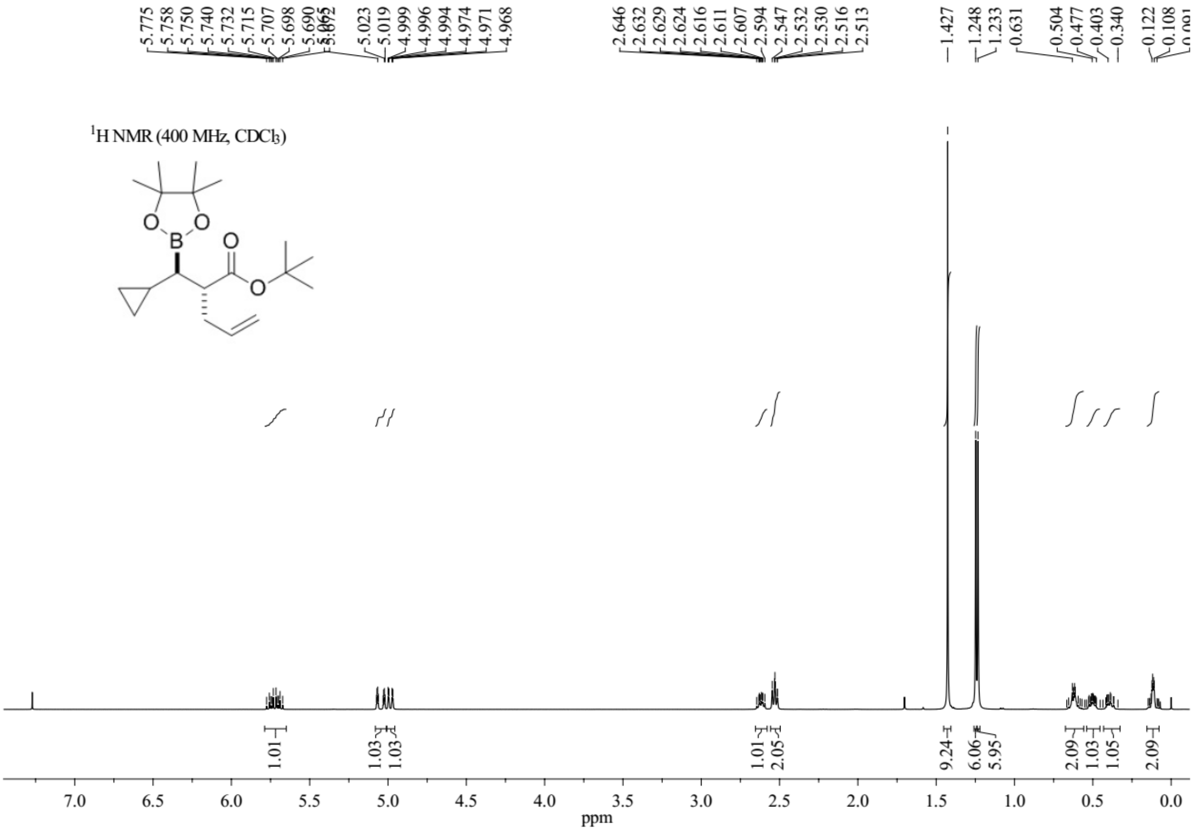

Supplementary Figure 132. ^{13}C NMR spectrum for 7n

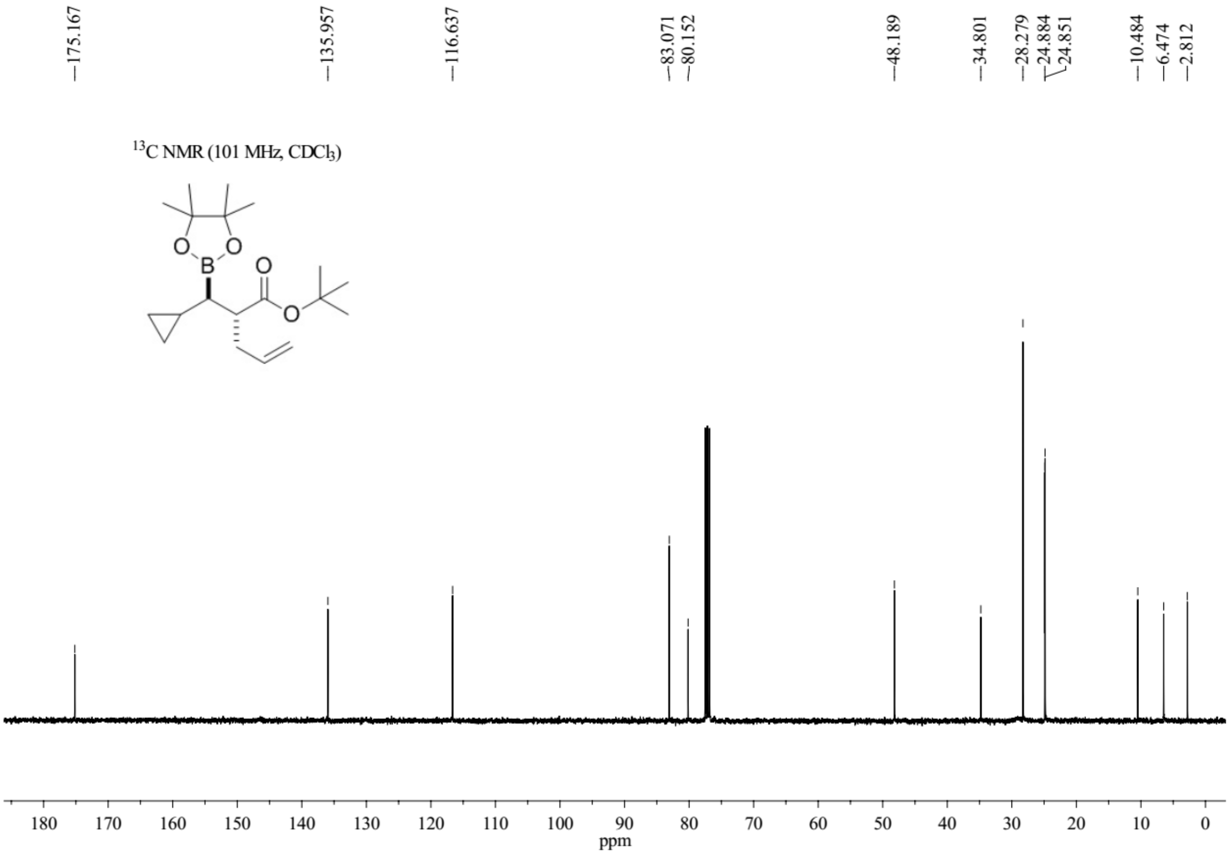

Supplementary Figure 133. ^{1}H NMR spectrum for *syn*-Pentan-3-yl-2-(cyclopropyl(4,4,5,5-tetramethyl-1,3,2-dioxaborolan-2-yl)methyl)pent-4-enoate (8n)

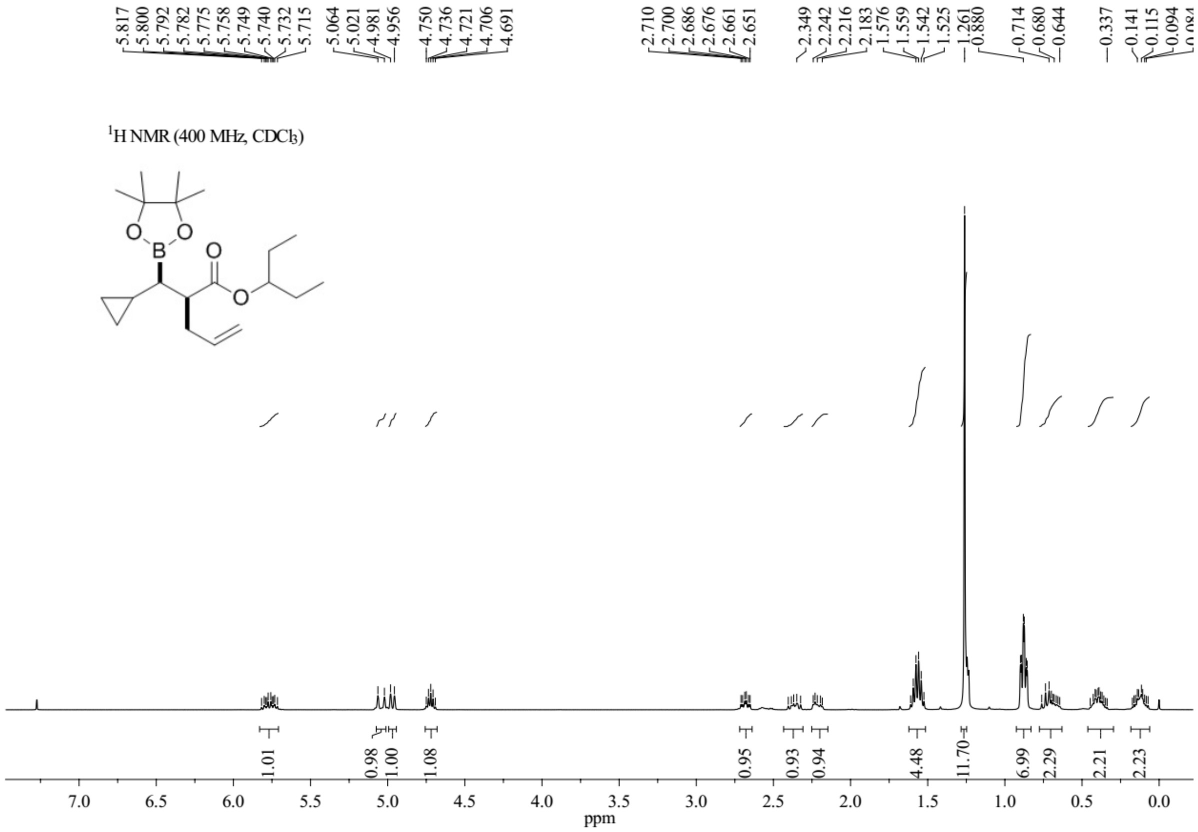

Supplementary Figure 134. ^{13}C NMR spectrum for 8n

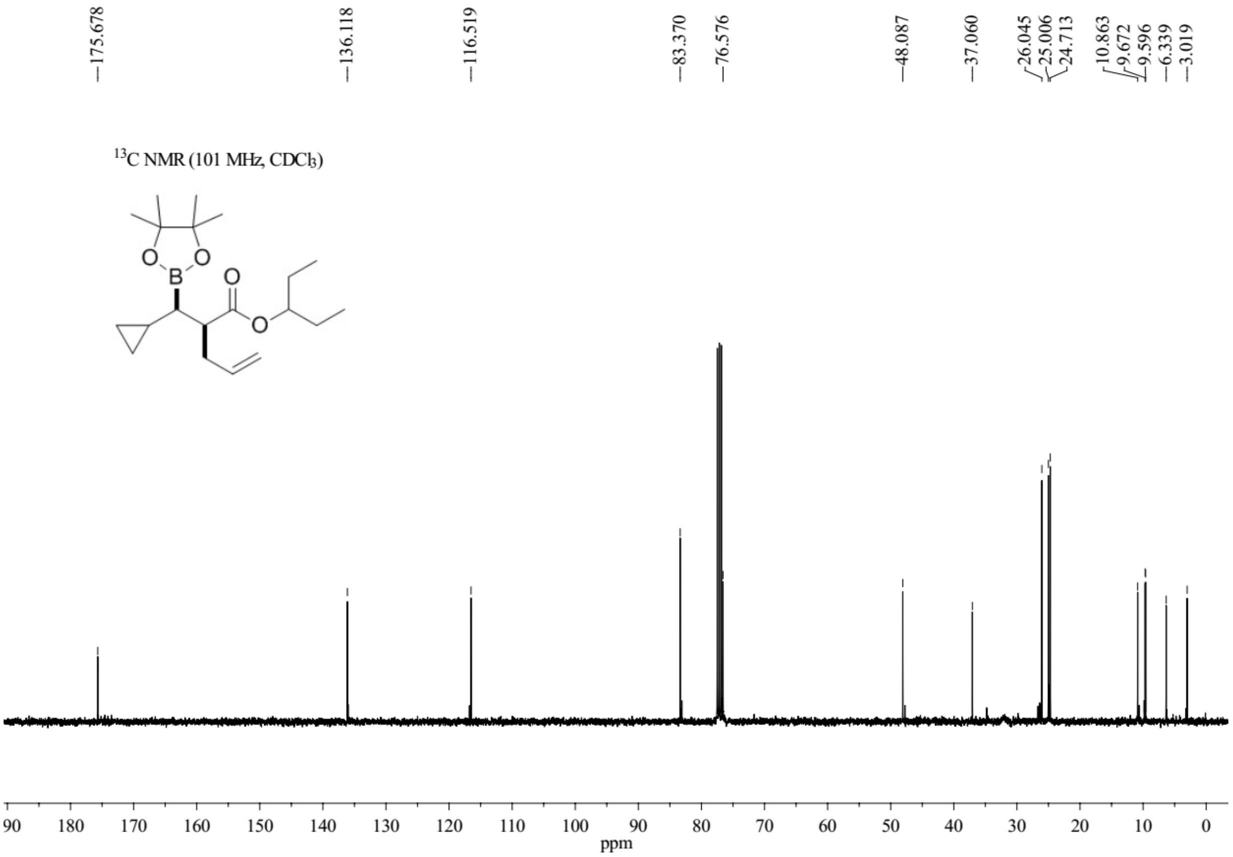

Supplementary Figure 135. ^{1}H NMR spectrum for *anti*-Pentan-3-yl (2*S*,3*S*,*E*)-2-allyl-3-(4,4,5,5-tetramethyl-1,3,2-dioxaborolan-2-yl)hex-4-enoate (7o)

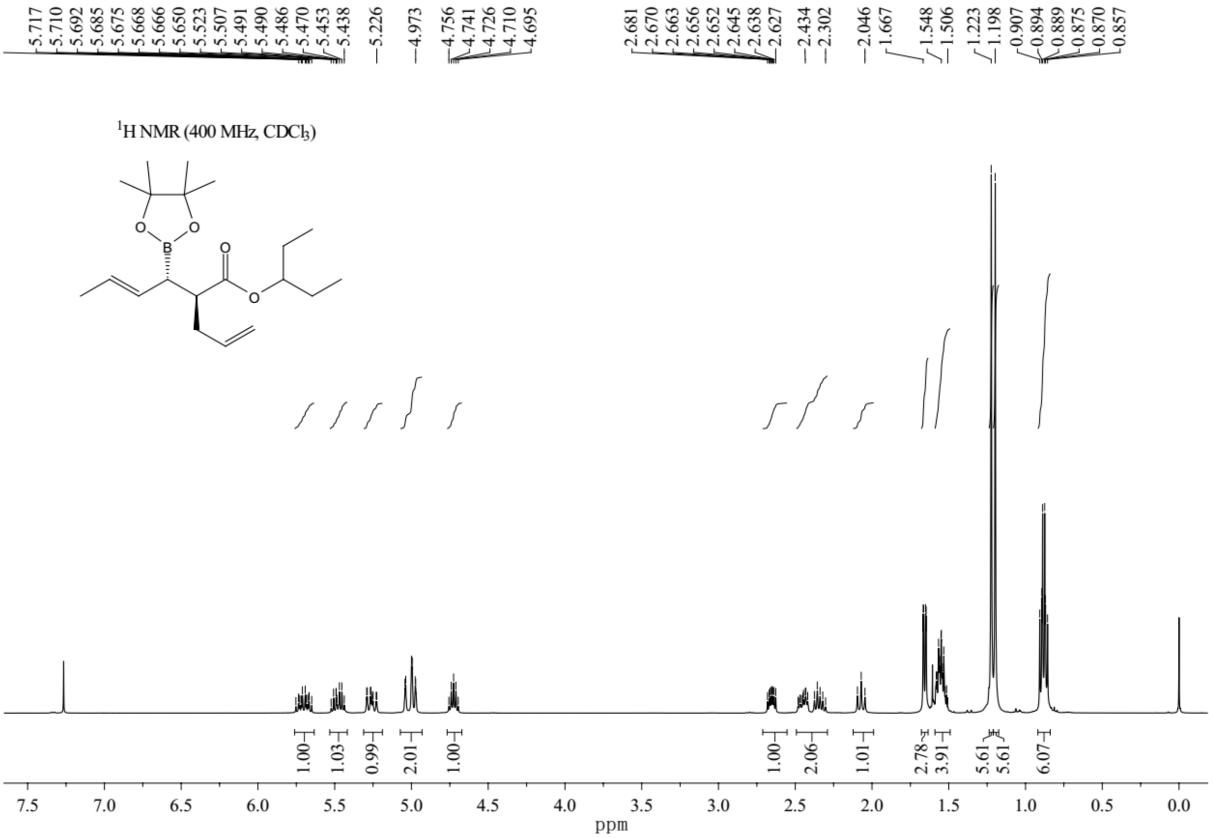

Supplementary Figure 136. ^{13}C NMR spectrum for 7o

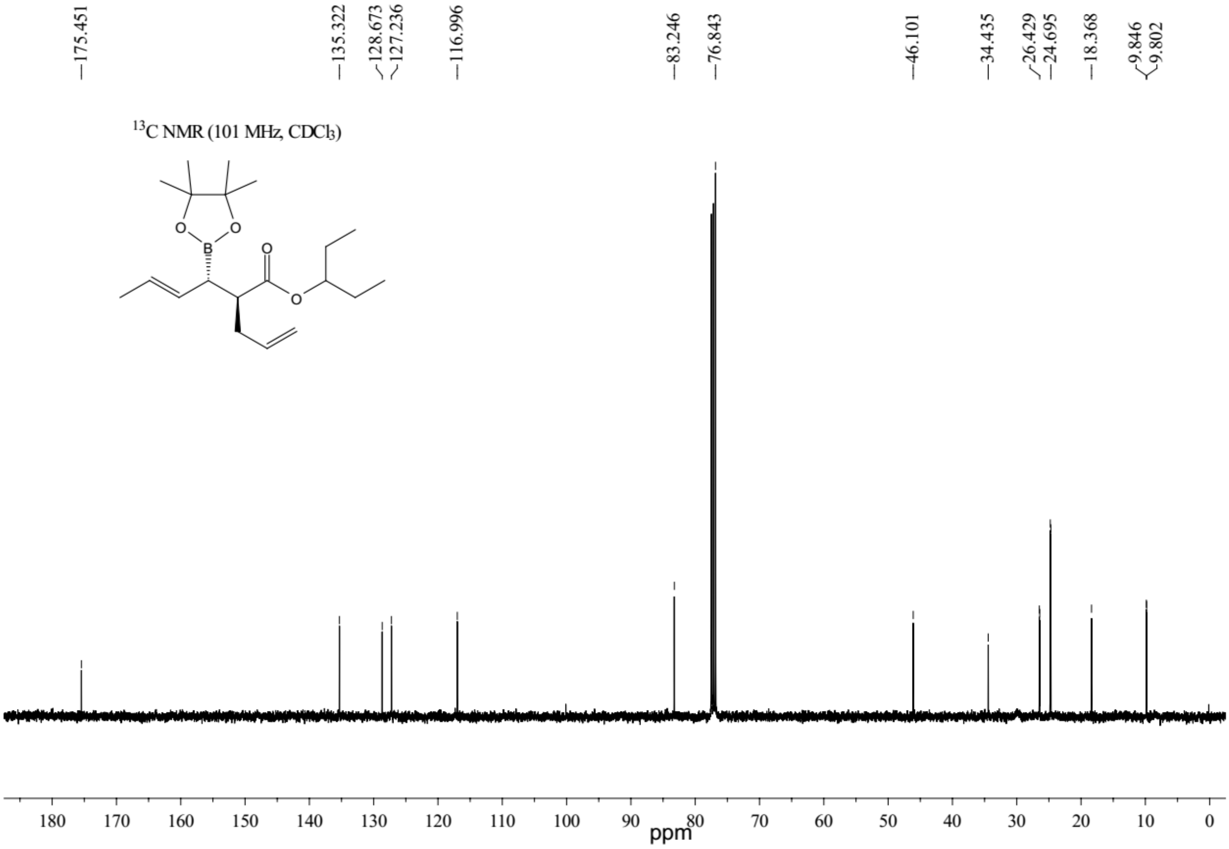

Supplementary Figure 137. ^{1}H NMR spectrum for *syn*-Pentan-3-yl (2*R*,3*S*,*E*)-2-allyl-3-(4,4,5,5-tetramethyl-1,3,2-dioxaborolan-2-yl)hex-4-enoate (8o)

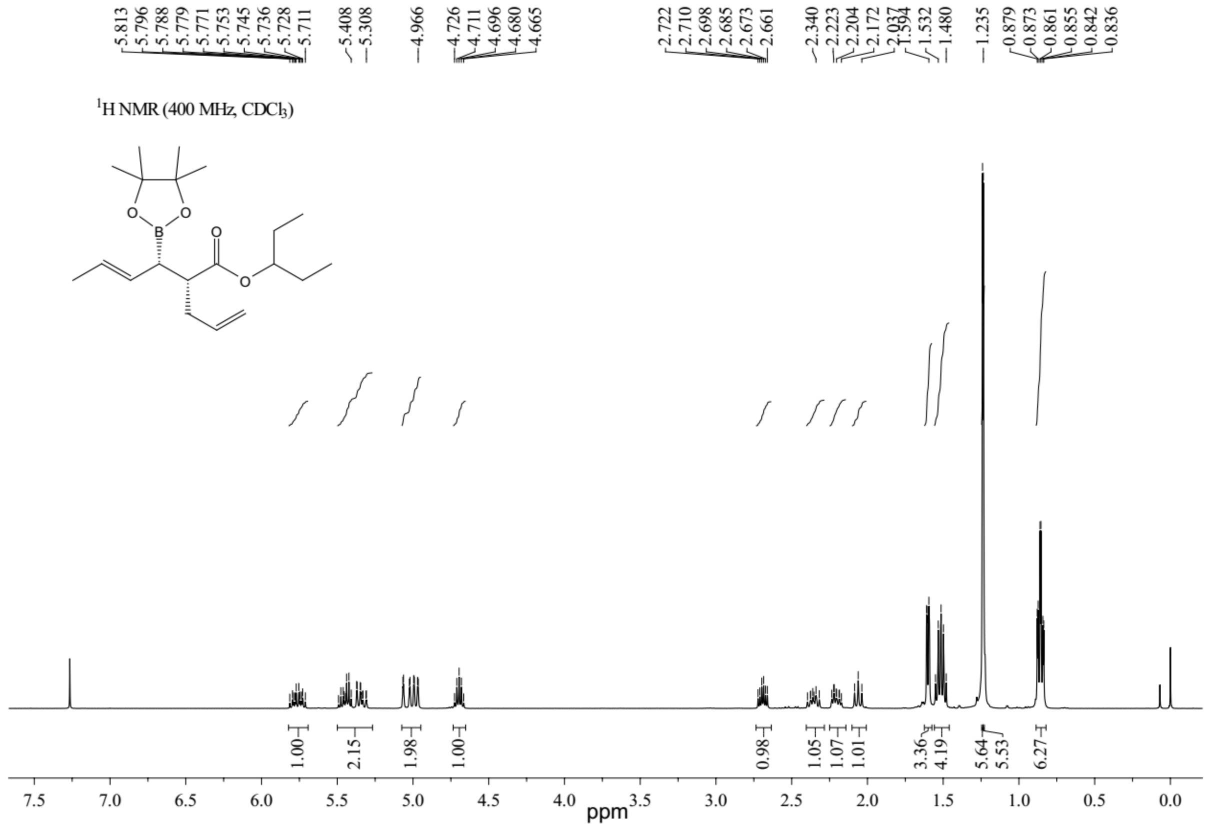

Supplementary Figure 138. ^{13}C NMR spectrum for 8o

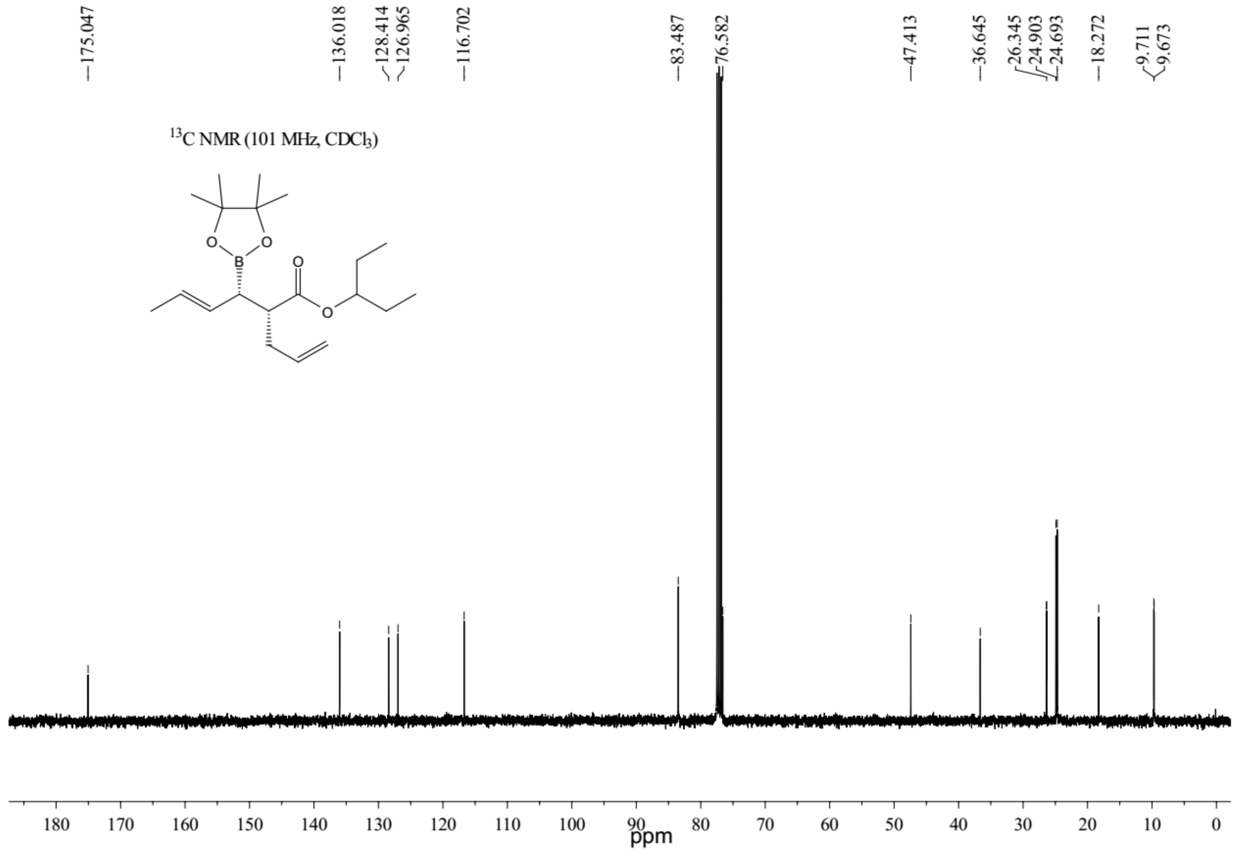

Supplementary Figure 139. ^{1}H NMR spectrum for *anti*-Pentan-3-yl 2-allyl-5-methyl-3-(4,4,5,5-tetramethyl-1,3,2-dioxaborolan-2-yl)hex-4-enoate (7p)

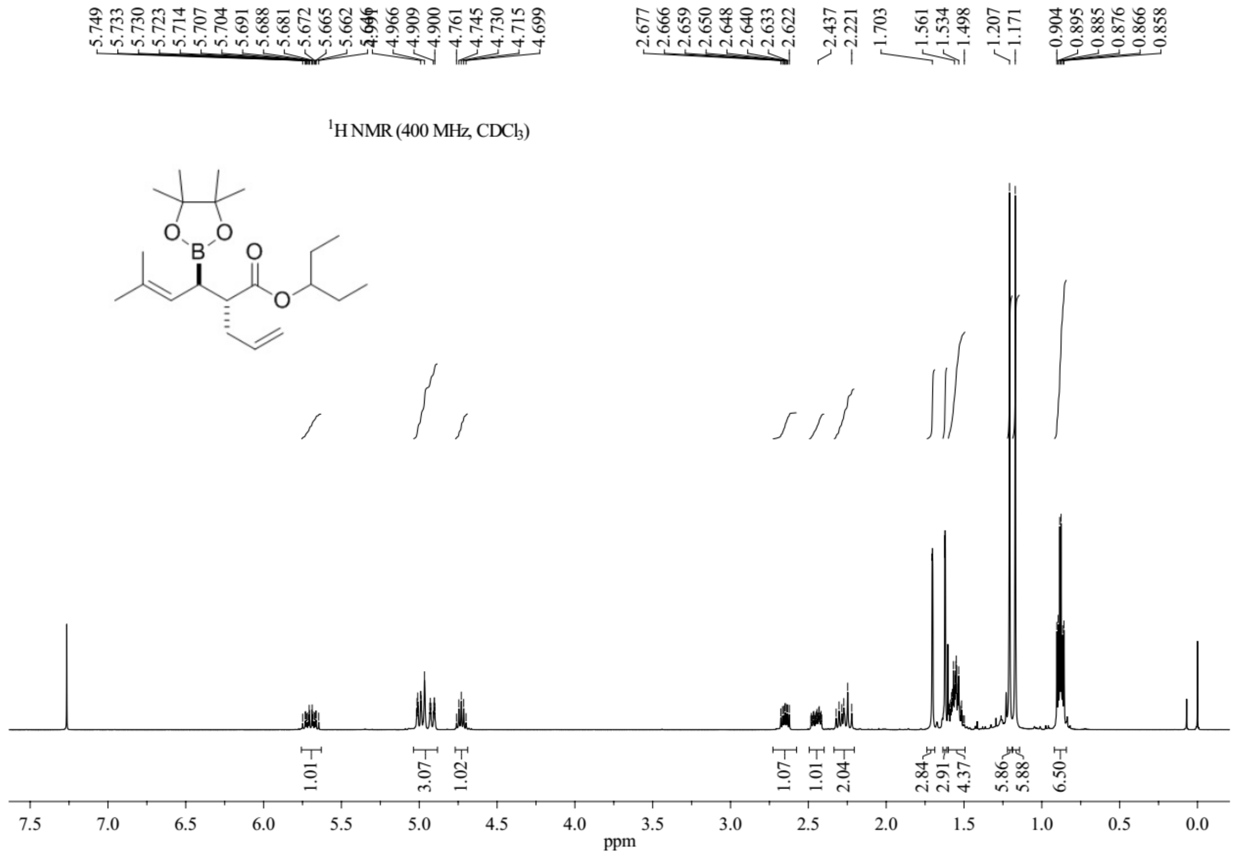

Supplementary Figure 140. ^{13}C NMR spectrum for 7p

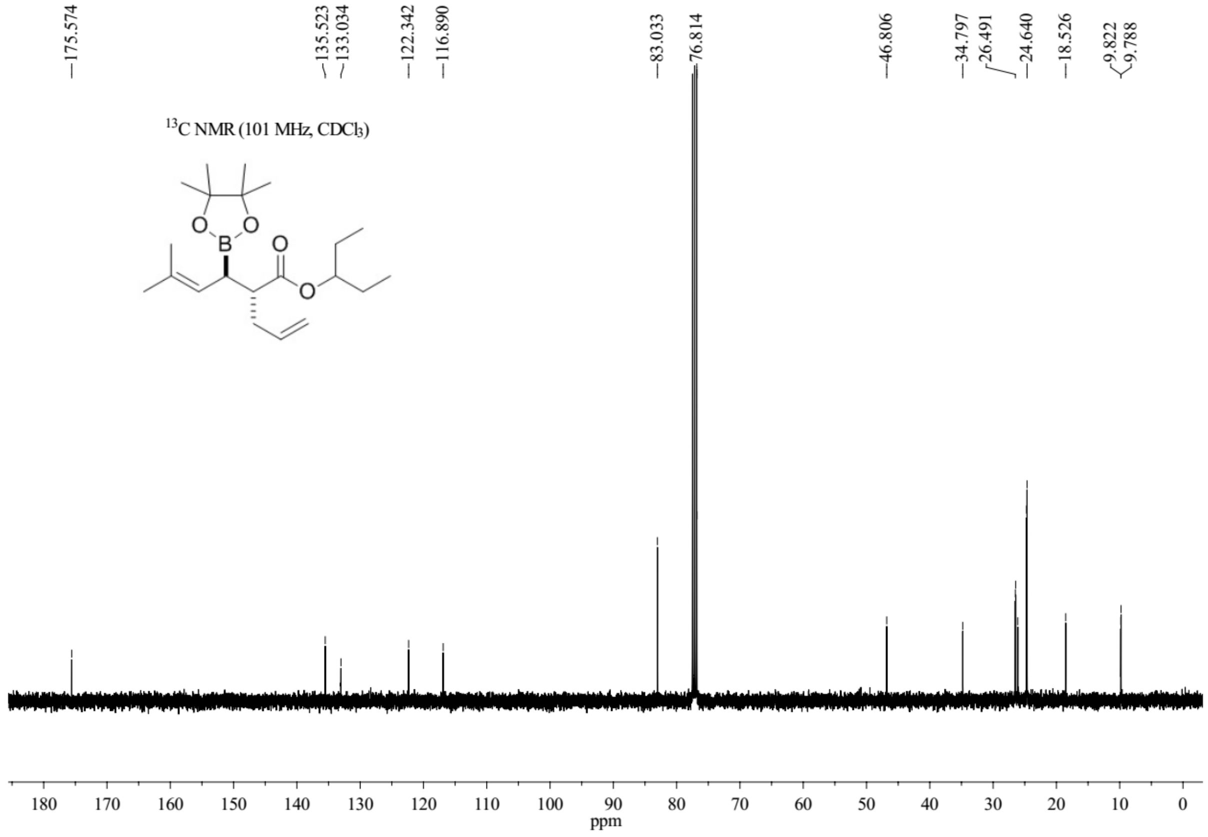

Supplementary Figure 141. ^{1}H NMR spectrum for *syn*-Pentan-3-yl 2-allyl-5-methyl-3-(4,4,5,5-tetramethyl-1,3,2-dioxaborolan-2-yl)hex-4-enoate (8p)

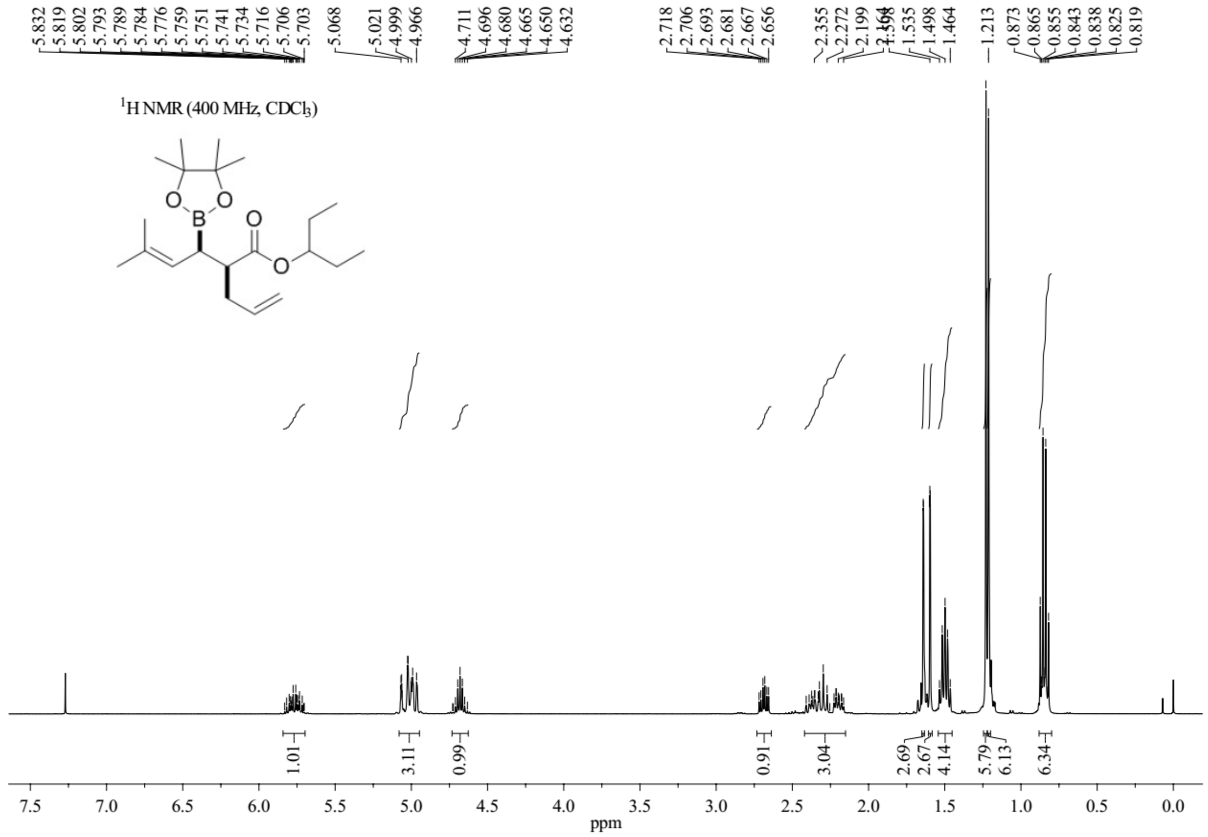

Supplementary Figure 142. ^{13}C NMR spectrum for 8p

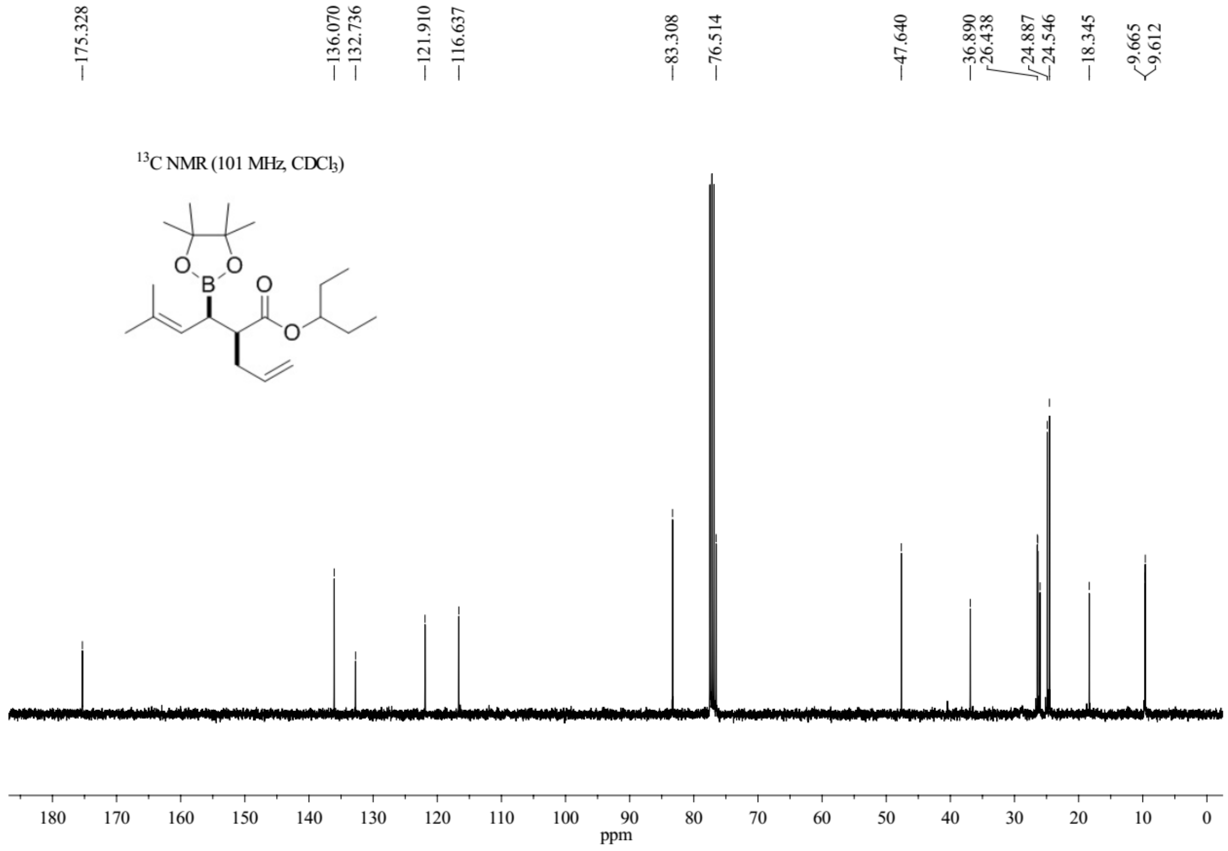

Supplementary Figure 143. ¹H NMR spectrum for *anti-tert*-Butyl-2-methyl-3-phenyl-3-(4,4,5,5-tetramethyl-1,3,2-dioxaborolan-2-yl)propanoate (7q)

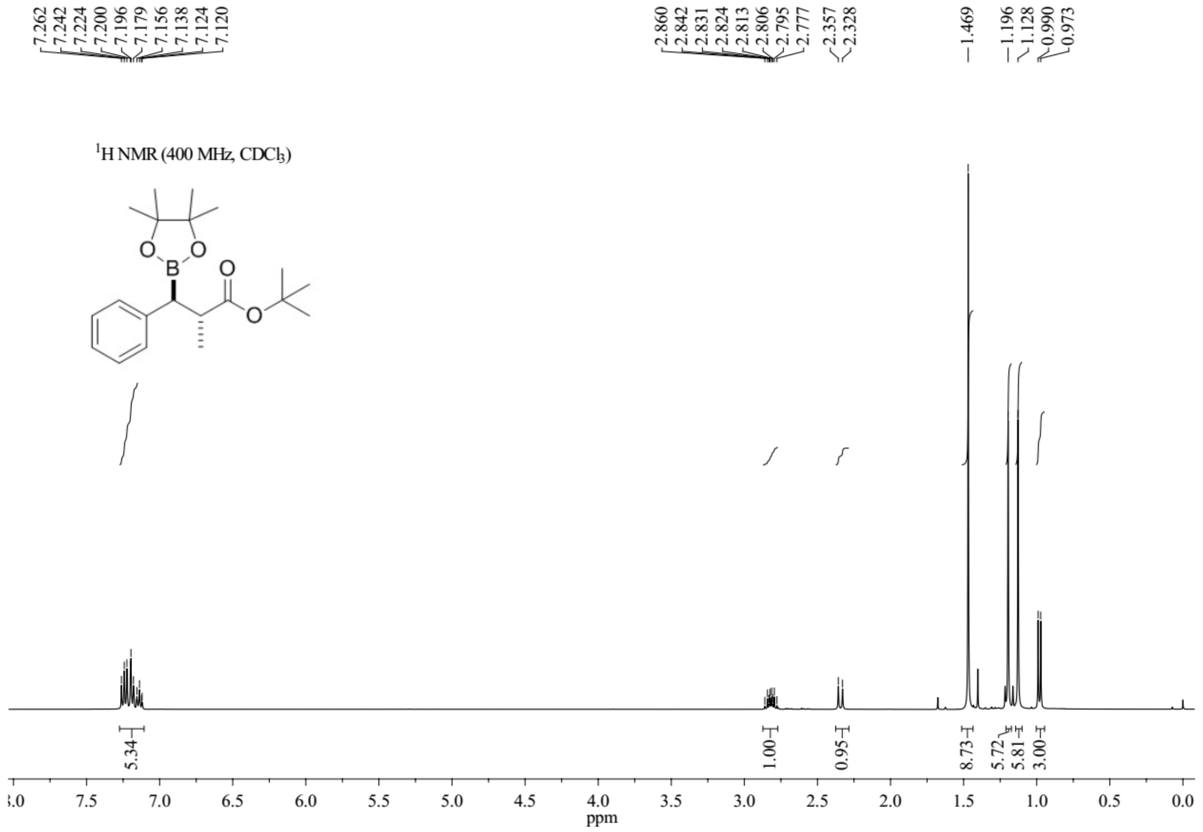

Supplementary Figure 144. ¹³C NMR spectrum for 7q

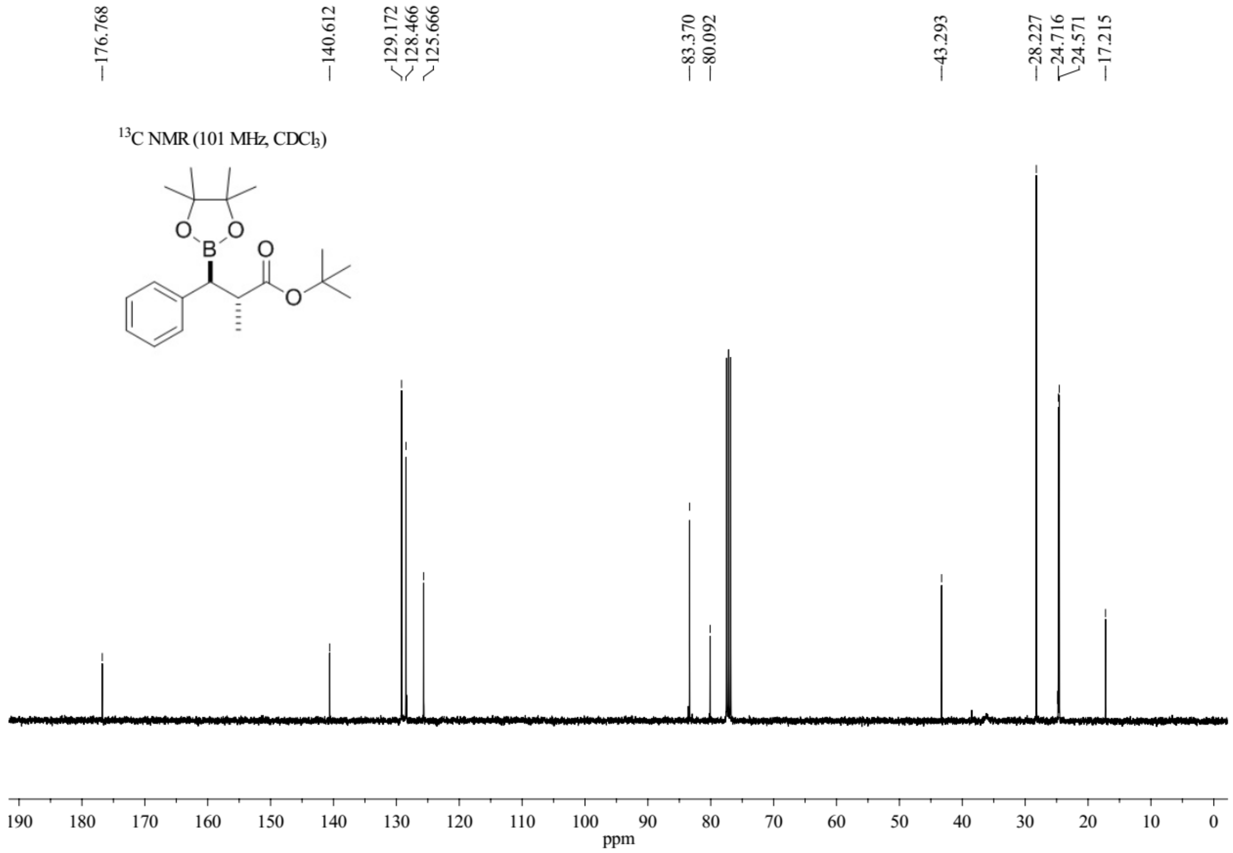

Supplementary Figure 145. ^{1}H NMR spectrum for *syn*-Pentan-3-yl-2-methyl-3-phenyl-3-(4,4,5,5-tetramethyl-1,3,2-dioxaborolan-2-yl)propanoate (8q)

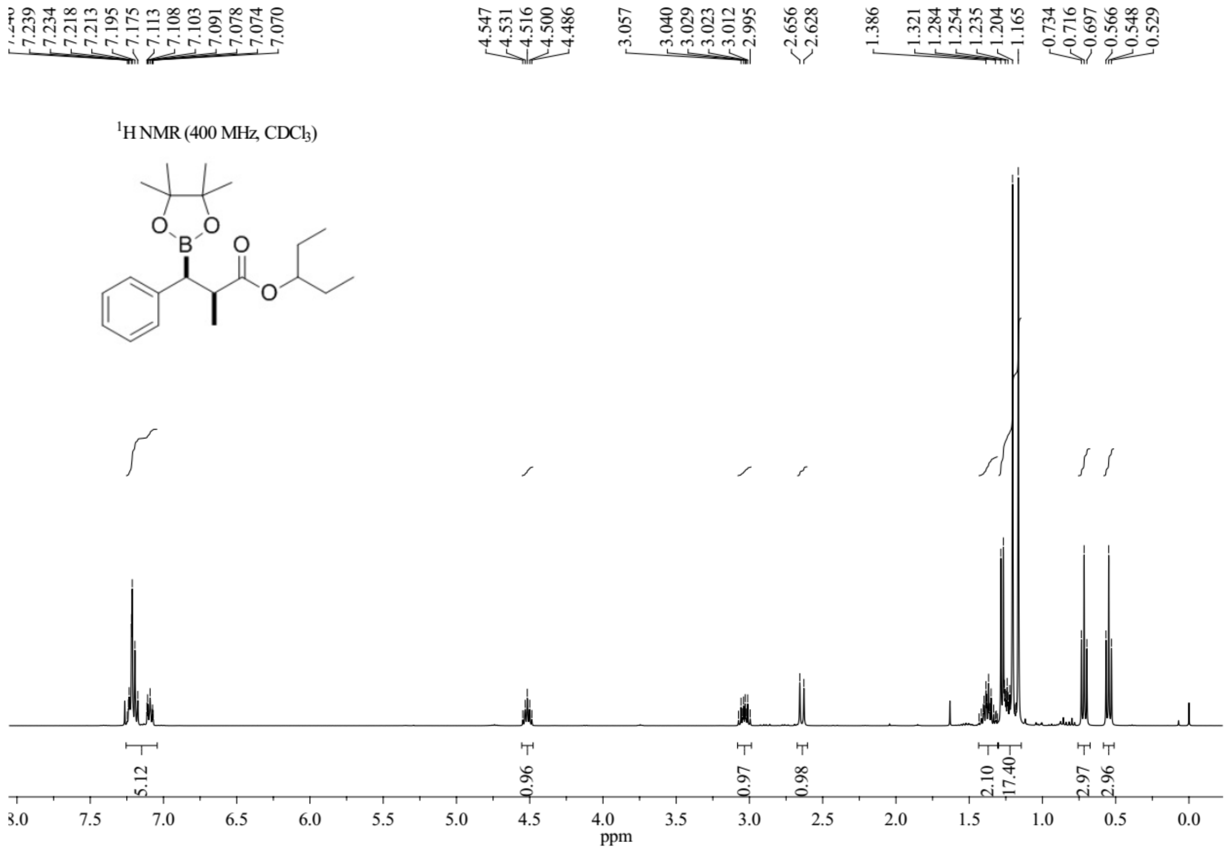

Supplementary Figure 146. ^{13}C NMR spectrum for 8q

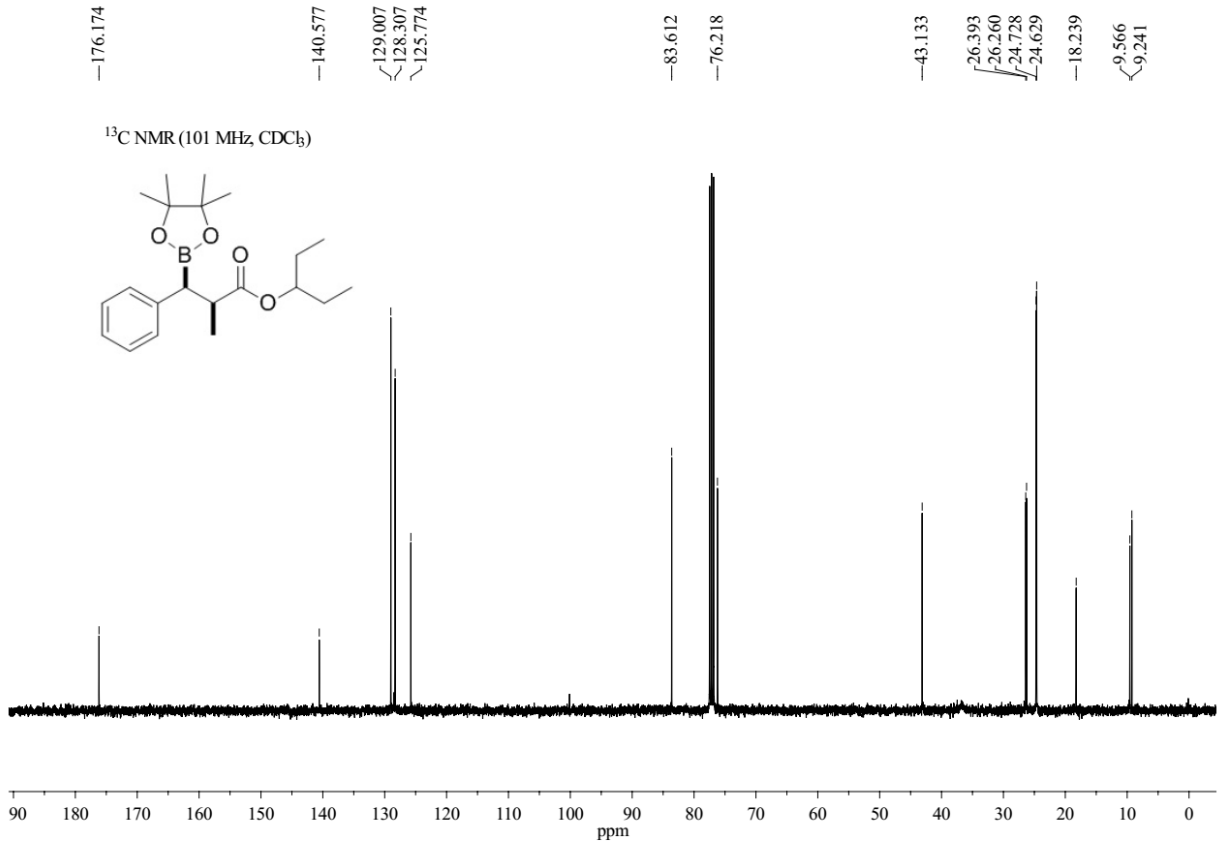

Supplementary Figure 147. ^{1}H NMR spectrum for *anti-tert*-Butyl-2-benzyl-3-phenyl-3-(4,4,5,5-tetramethyl-1,3,2-dioxaborolan-2-yl)propanoate (7r)

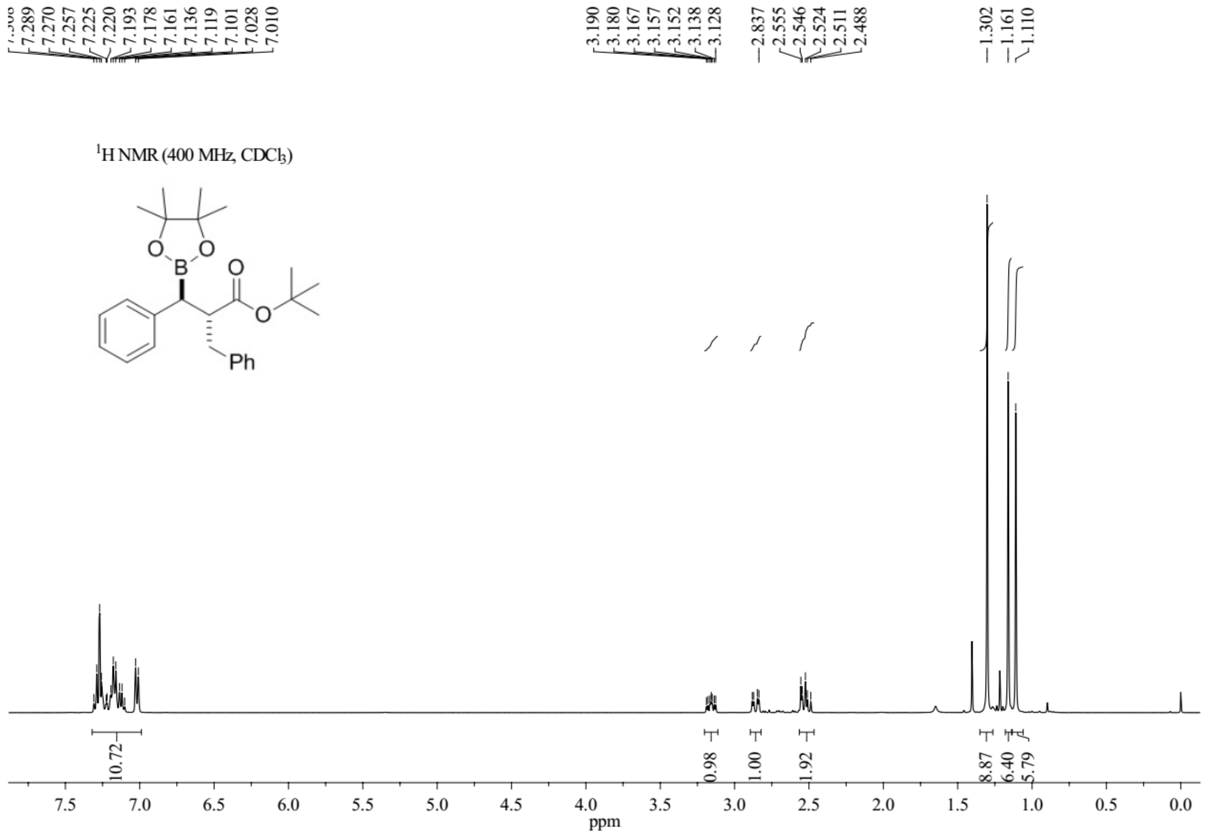

Supplementary Figure 148. ^{13}C NMR spectrum for 7r

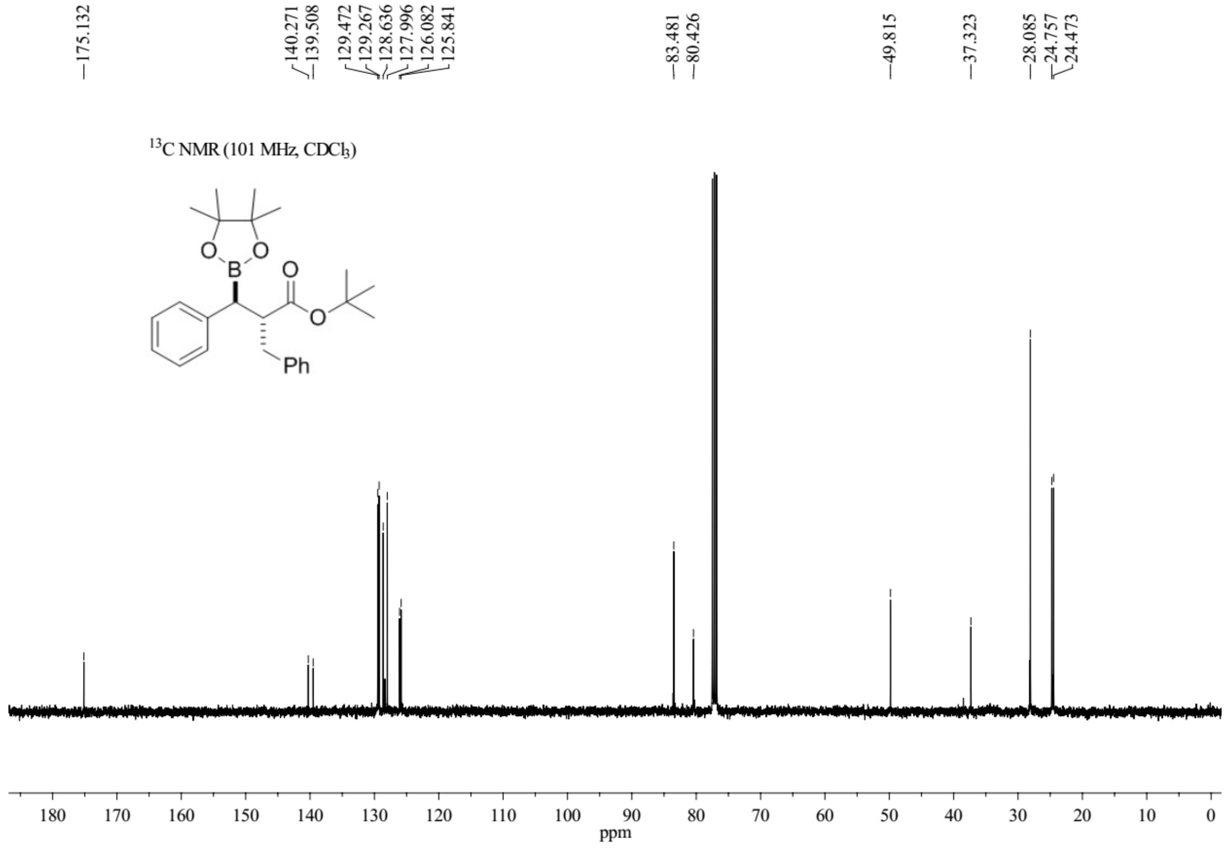

Supplementary Figure 149. ^{1}H NMR spectrum for *syn*-Pentan-3-yl-2-benzyl-3-phenyl-3-(4,4,5,5-tetramethyl-1,3,2-dioxaborolan-2-yl)propanoate (8r)

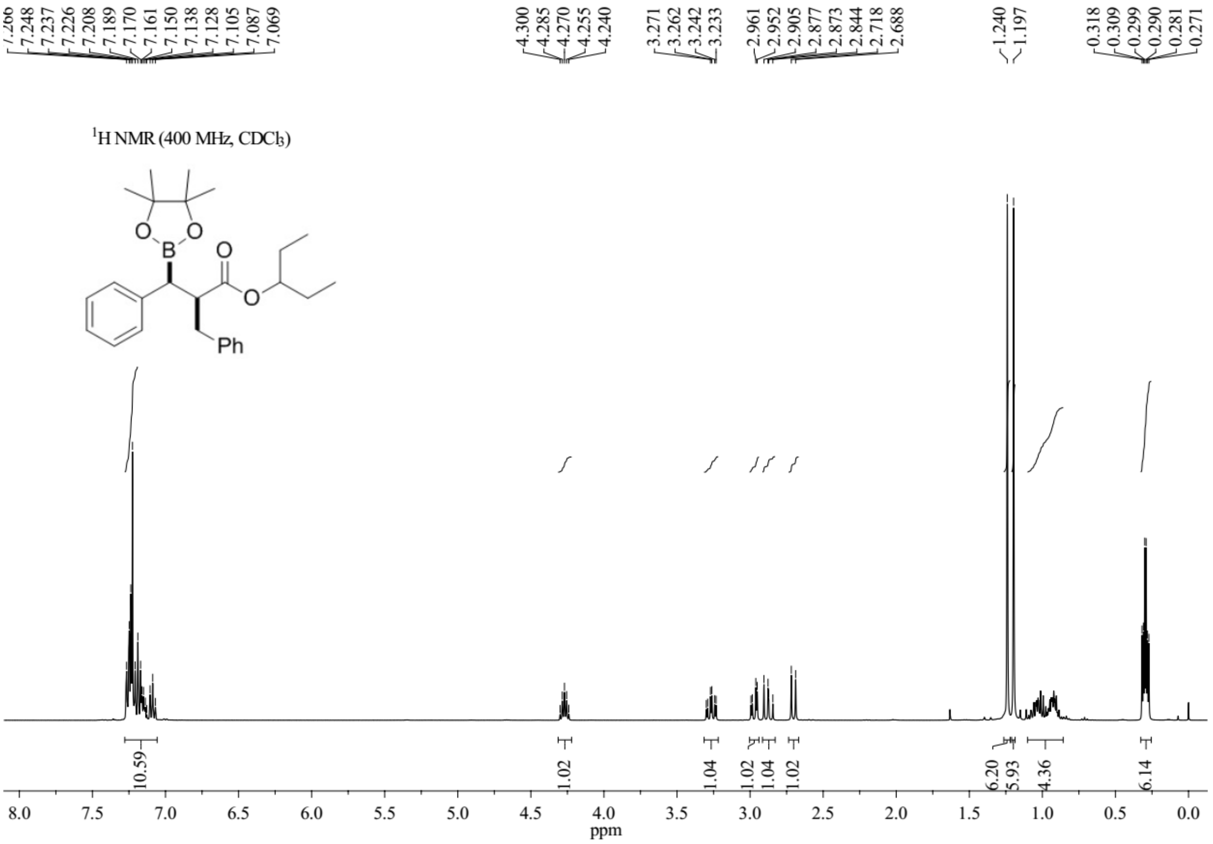

Supplementary Figure 150. ^{13}C NMR spectrum for 8r

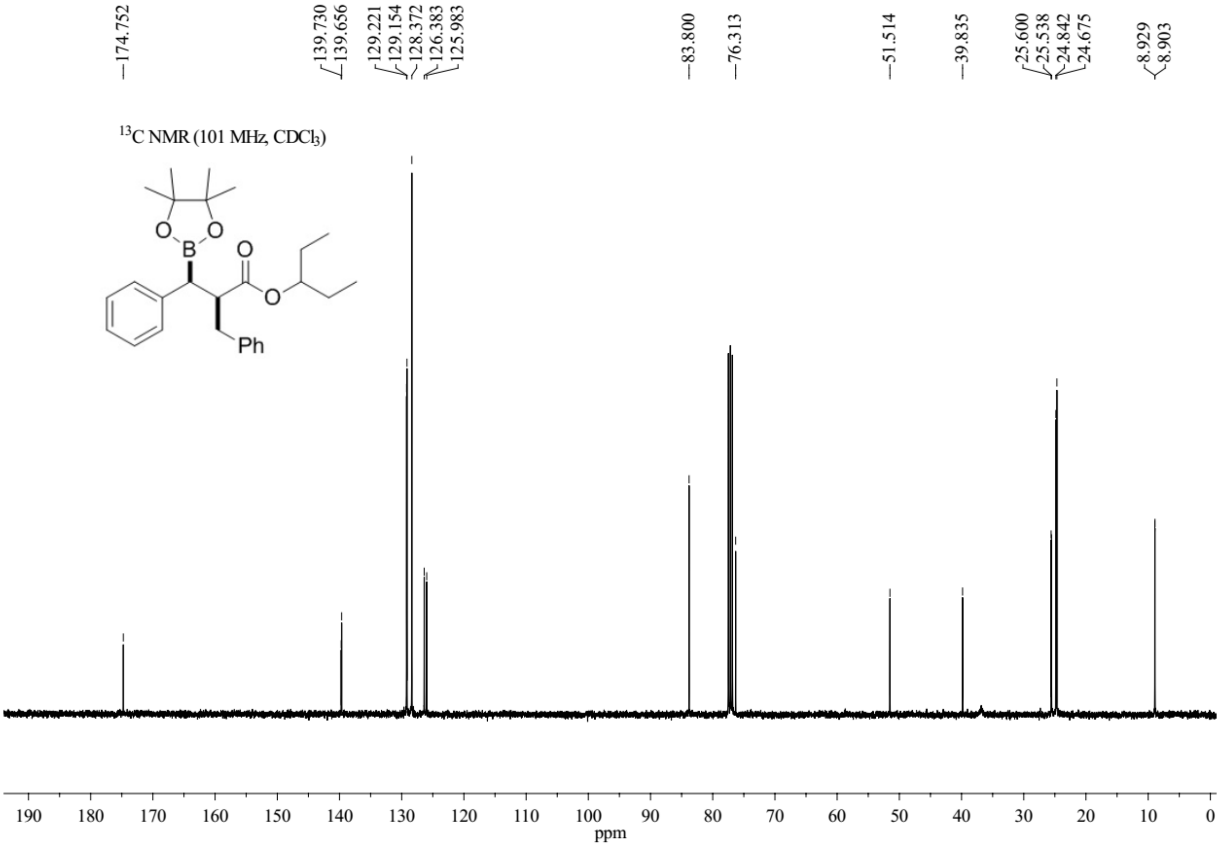

Supplementary Figure 151. ^{1}H NMR spectrum for*anti-tert*-Butyl(*E*)-5-phenyl-2-((S)-phenyl(4,4,5,5-tetramethyl-1,3,2-dioxaborolan-2-yl)methyl)pent -4-enoate (7s)

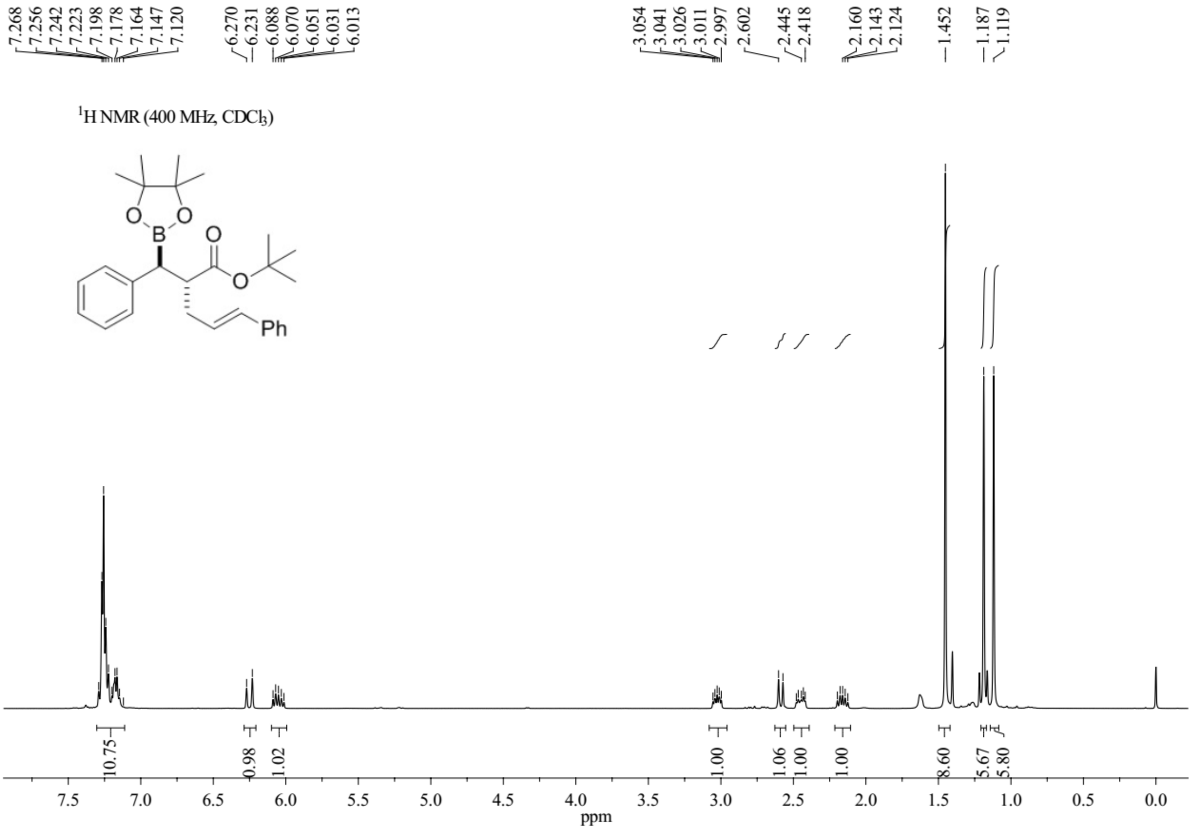

Supplementary Figure 152. ^{13}C NMR spectrum for 7s

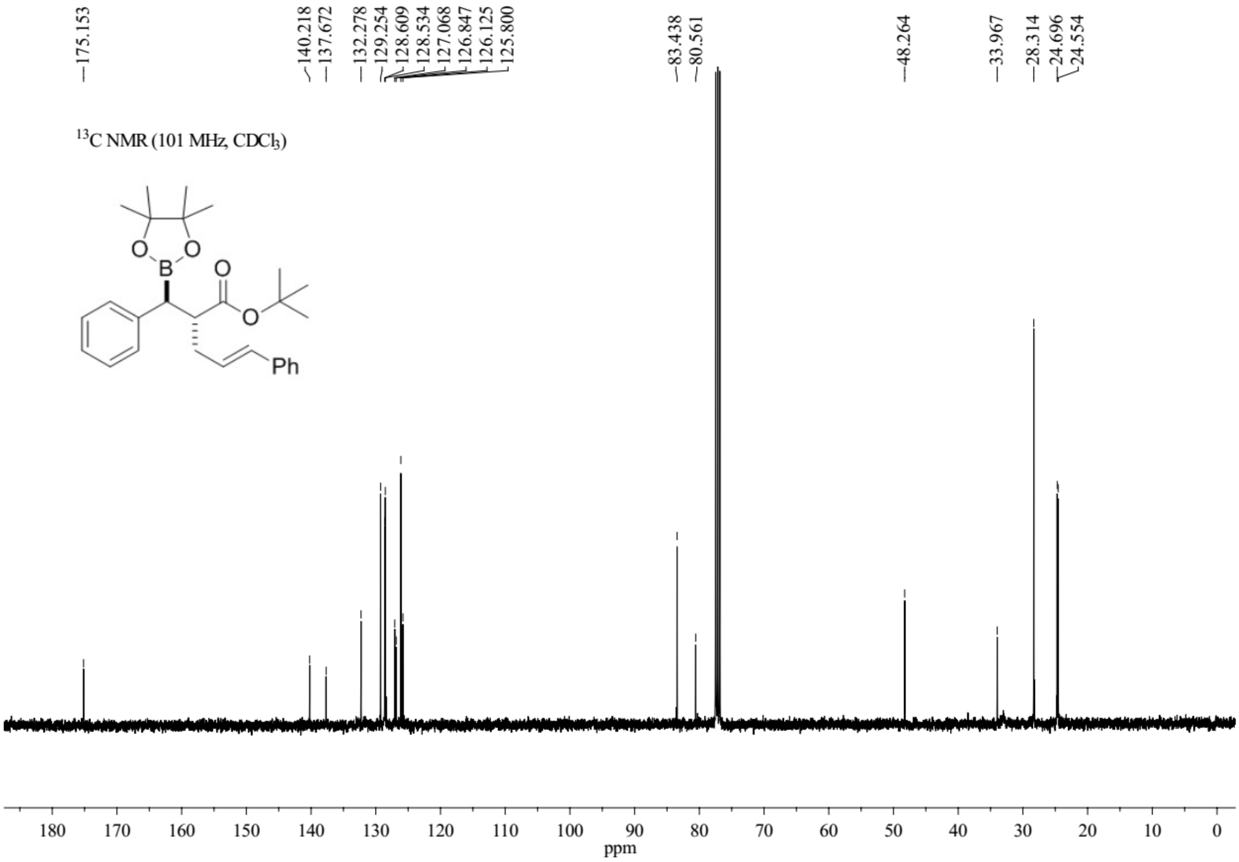

Supplementary Figure 153. ¹H NMR spectrum for *syn*-Pentan-3-yl (*E*)-5-phenyl-2-(phenyl(4,4,5,5-tetramethyl-1,3,2-dioxaborolan-2-yl)methyl)pent-4-enoate (8s)

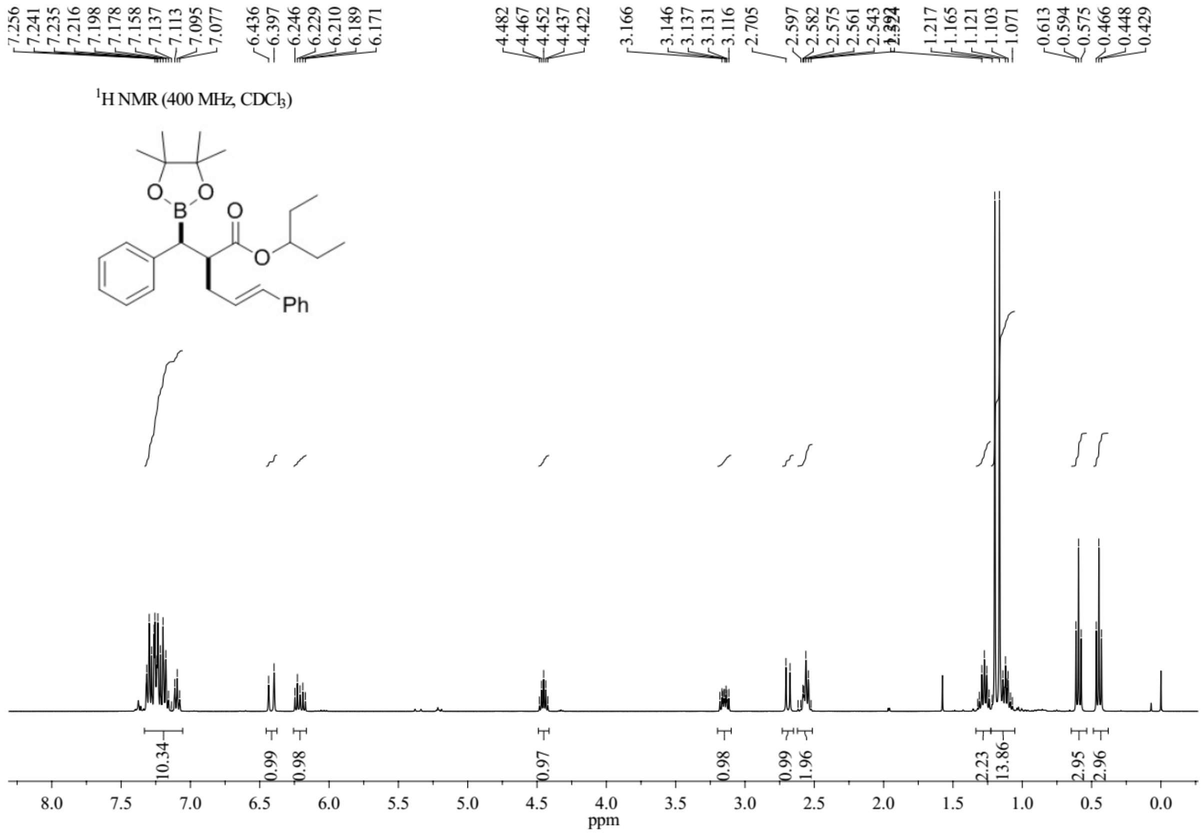

Supplementary Figure 154. ¹³C NMR spectrum for 8s

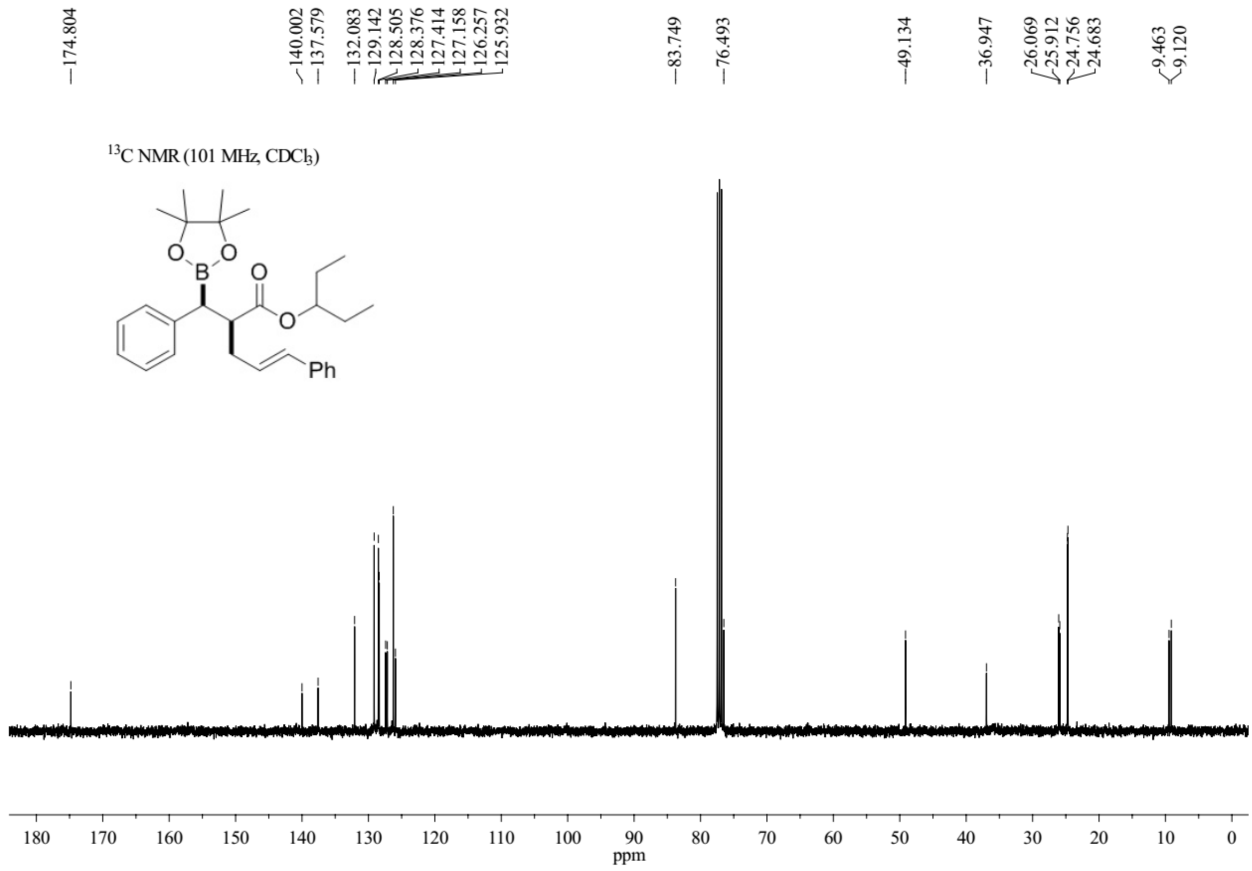

Supplementary Figure 155. ^{1}H NMR spectrum for *anti-tert*-Butyl-2-((benzyloxy)methyl)-3-phenyl-3-(4,4,5,5-tetramethyl-1,3,2-dioxaborolan-2-yl)propanoate (7t)

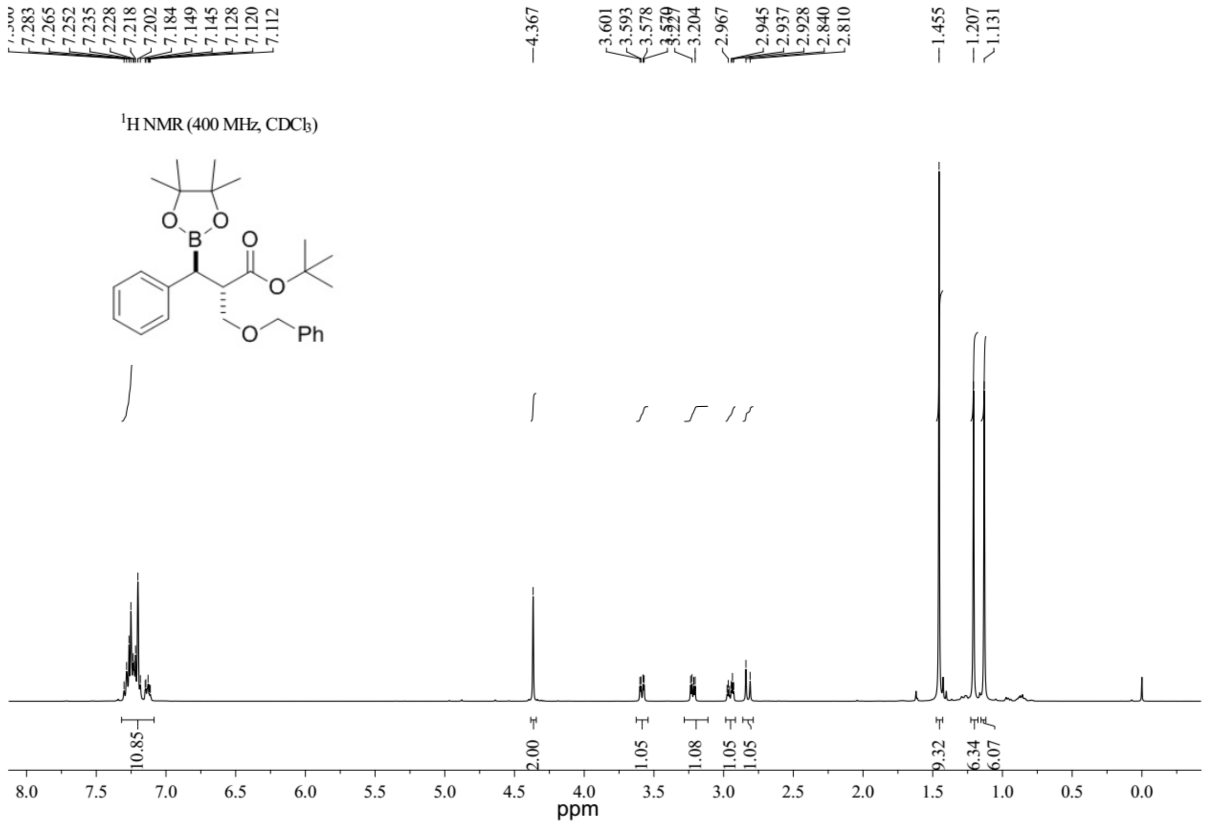

Supplementary Figure 156. ^{13}C NMR spectrum for 7t

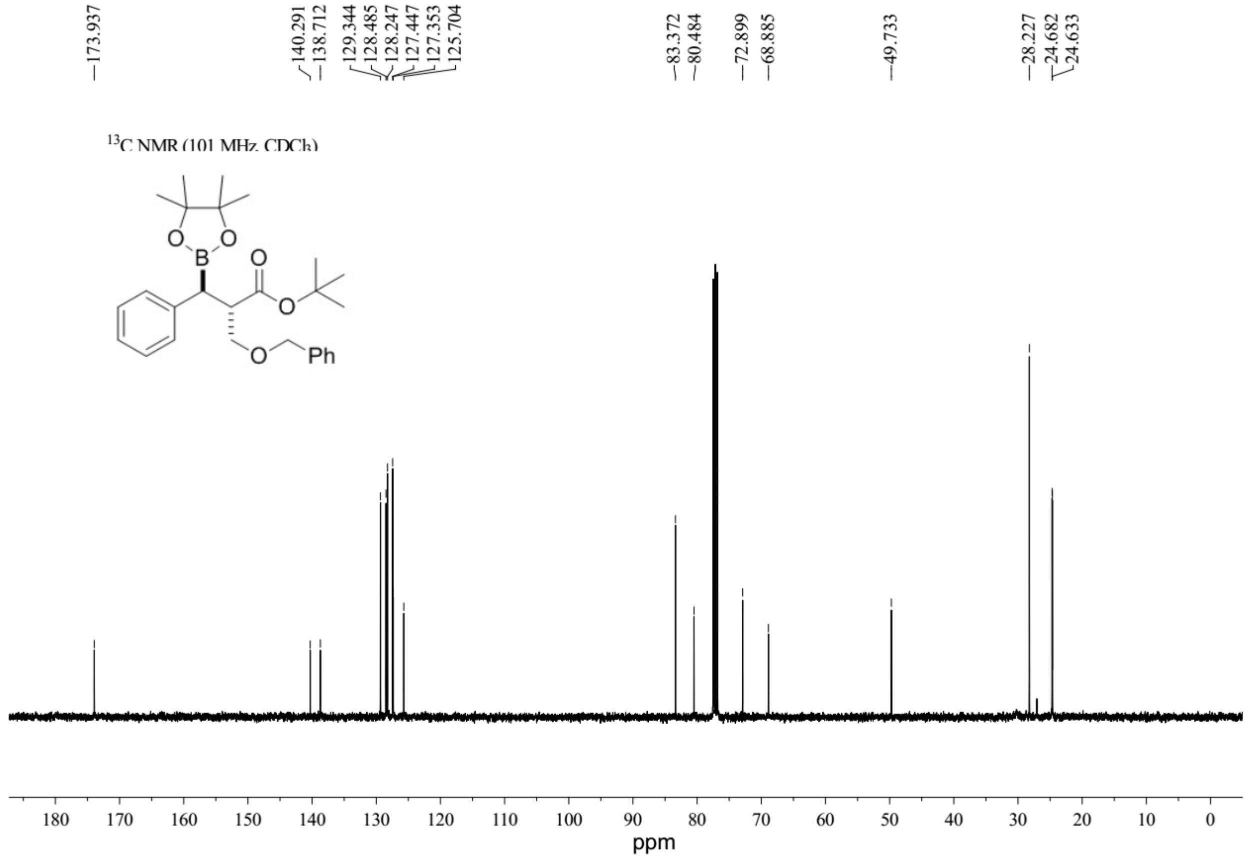

Supplementary Figure 157. ^{1}H NMR spectrum for *syn*-Pentan-3-yl-2-((benzyloxy)methyl)-3-phenyl-3-(4,4,5,5-tetramethyl-1,3,2-dioxaborolan-2-yl)propanoate (8t)

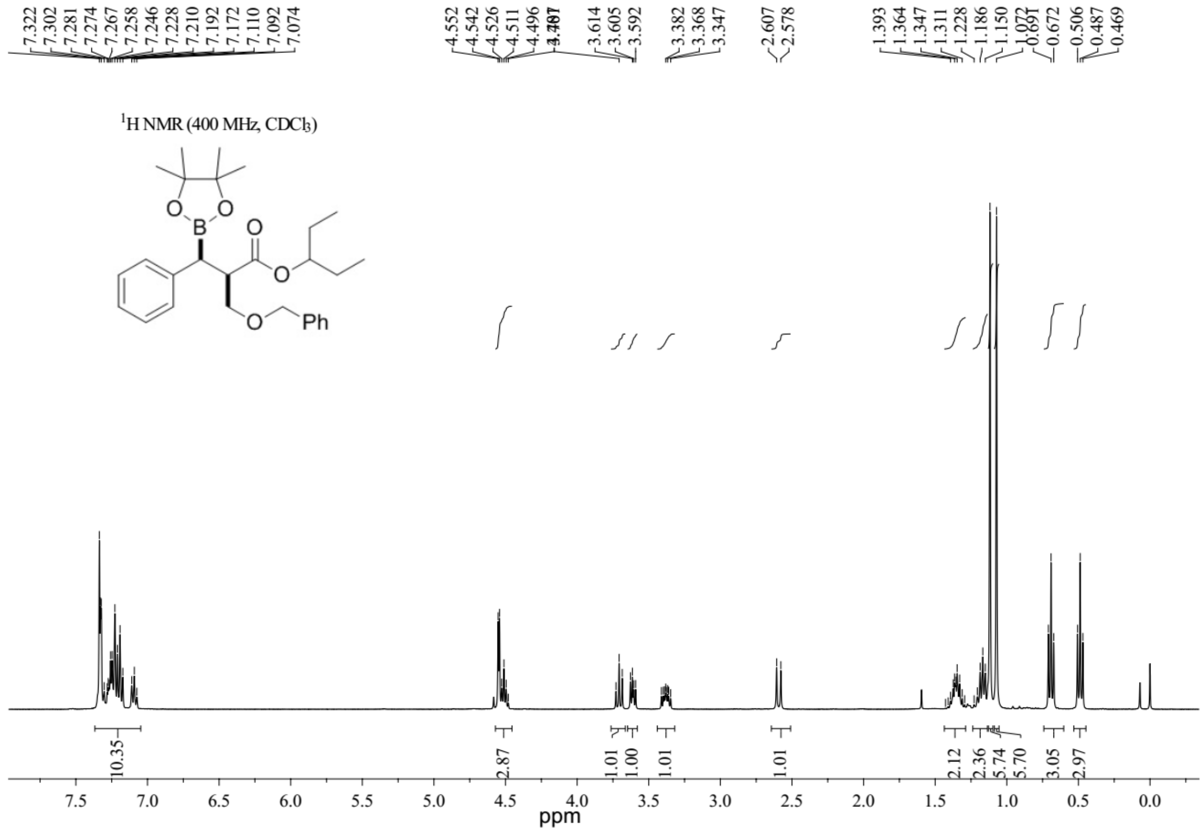

Supplementary Figure 158. ^{13}C NMR spectrum for 8t

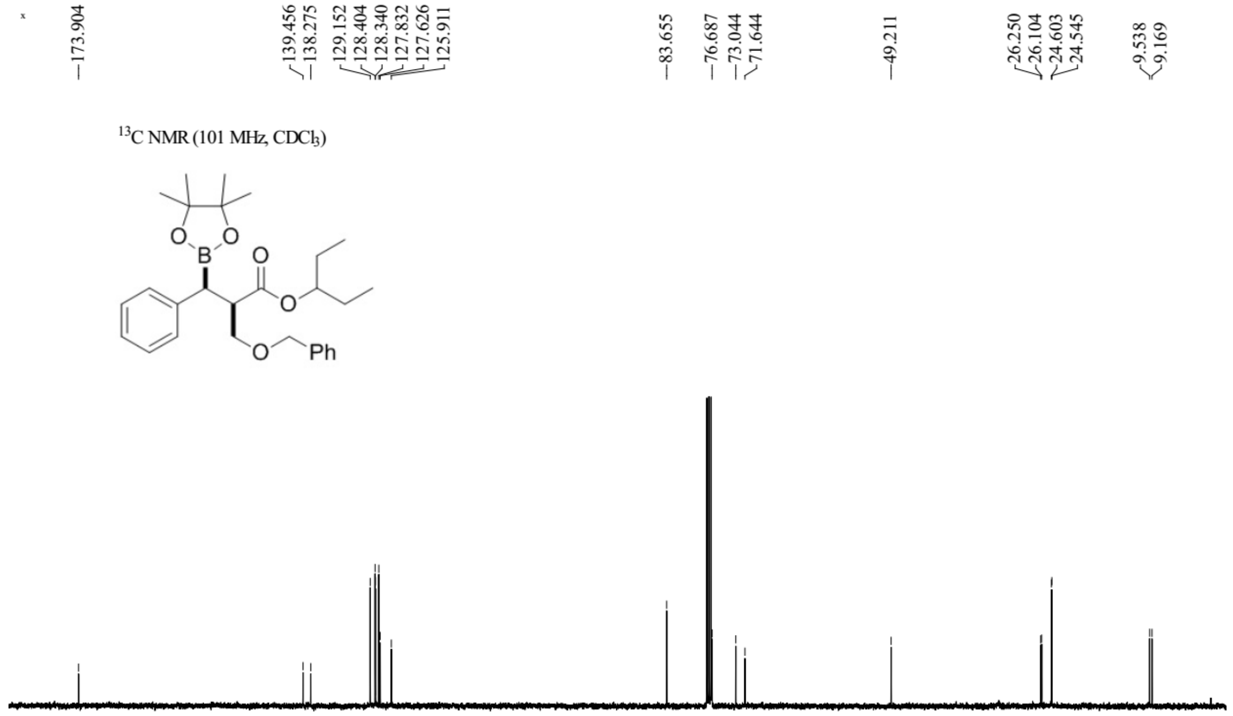

Supplementary Figure 159. ^{1}H NMR spectrum for *anti-tert*-Butyl-2-(phenyl(4,4,5,5-tetramethyl-1,3,2-dioxaborolan-2-yl)methyl)-5-(trimethylsilyl)pe nt-4-ynoate (7u)

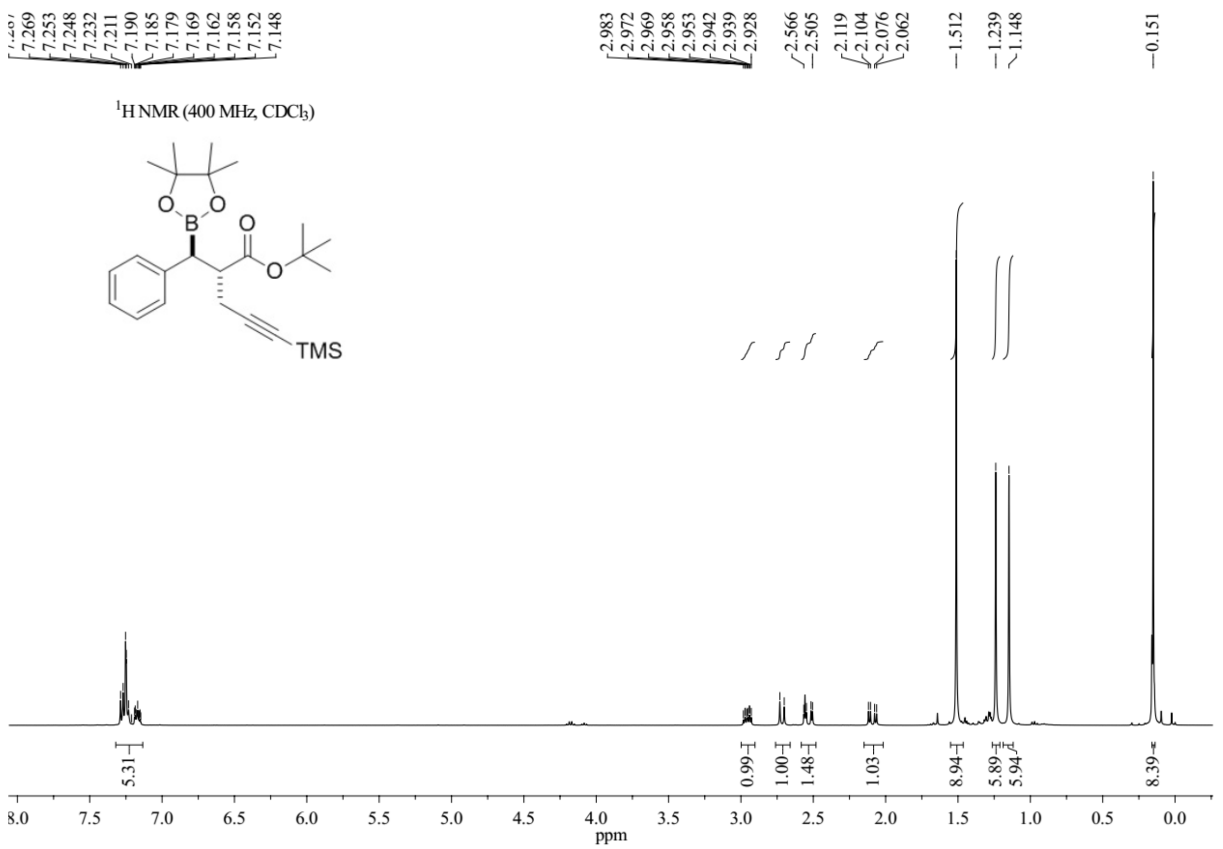

Supplementary Figure 160. ^{13}C NMR spectrum for 7u

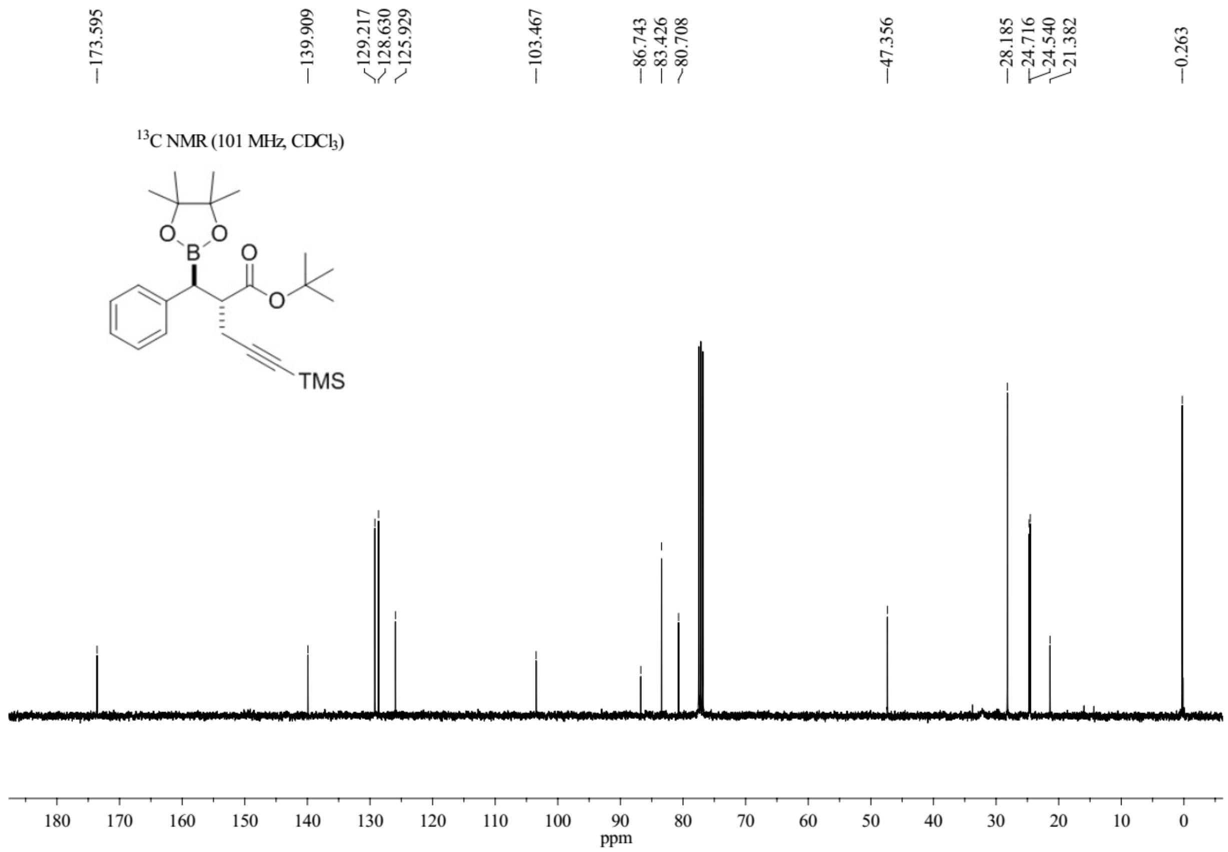

Supplementary Figure 161. ^{1}H NMR spectrum for *syn*-Pentan-3-yl-2-(phenyl(4,4,5,5-tetramethyl-1,3,2-dioxaborolan-2-yl)methyl)-5-(trimethylsilyl)p ent-4-ynoate (8u)

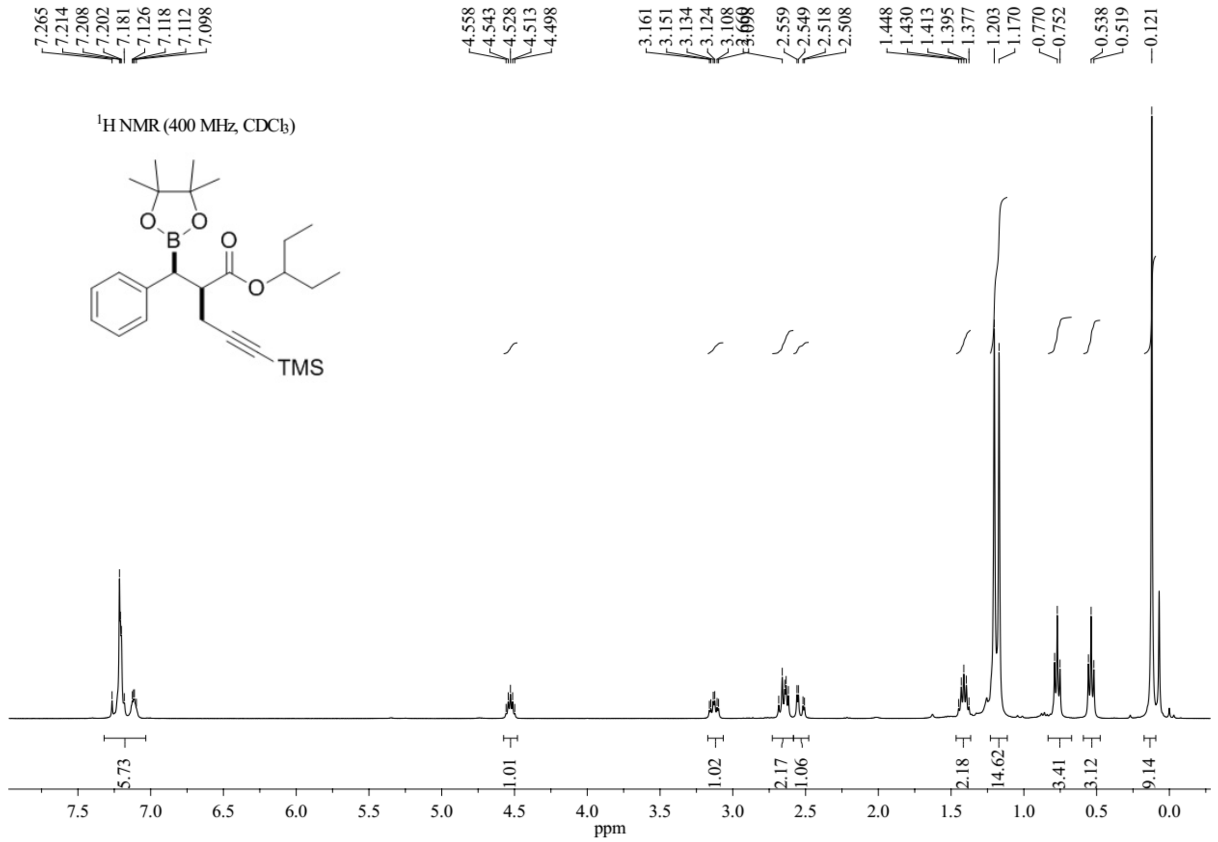

Supplementary Figure 162. ^{13}C NMR spectrum for 8u

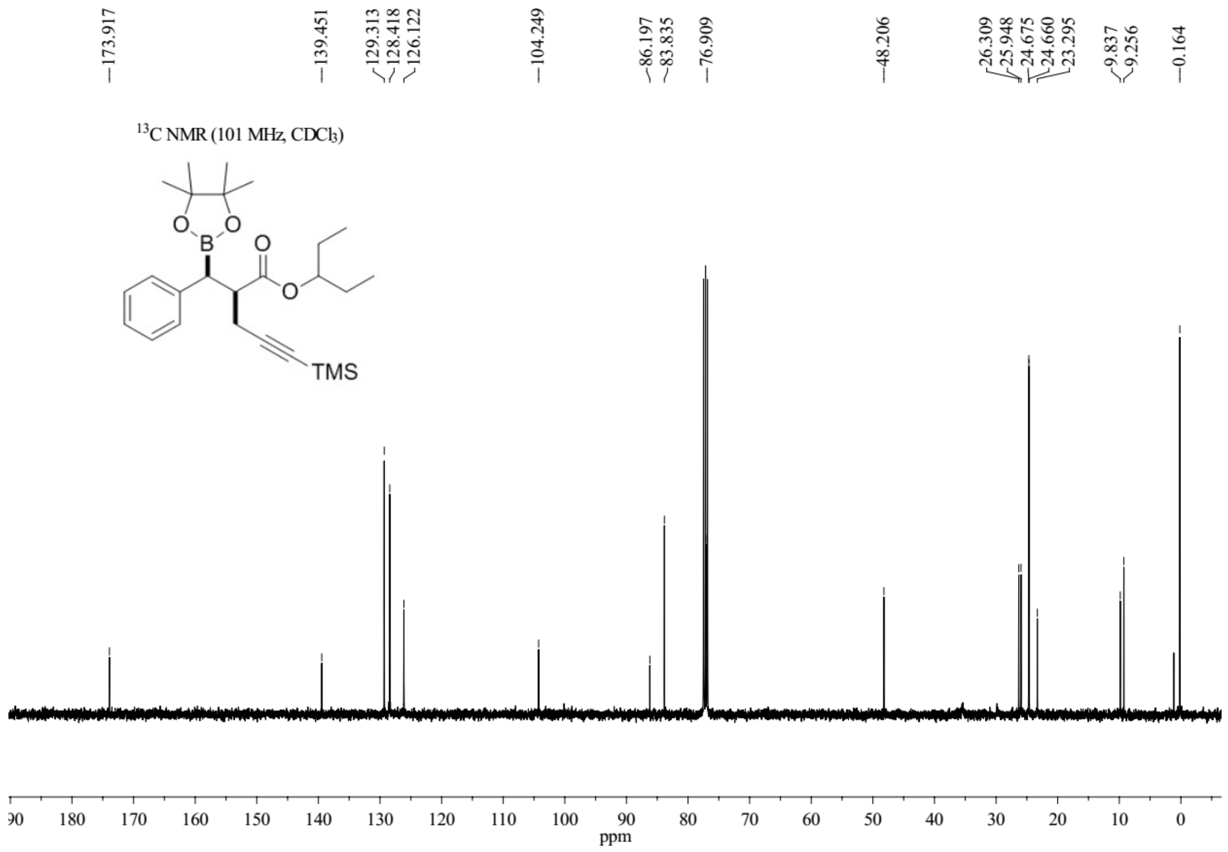

Supplementary Figure 163. **^{1}H NMR spectrum for *anti-tert*-Butyl-3-phenyl-2-(phenylthio)-3-(4,4,5,5-tetramethyl-1,3,2-dioxaborolan-2-yl)propanoate (7v)**

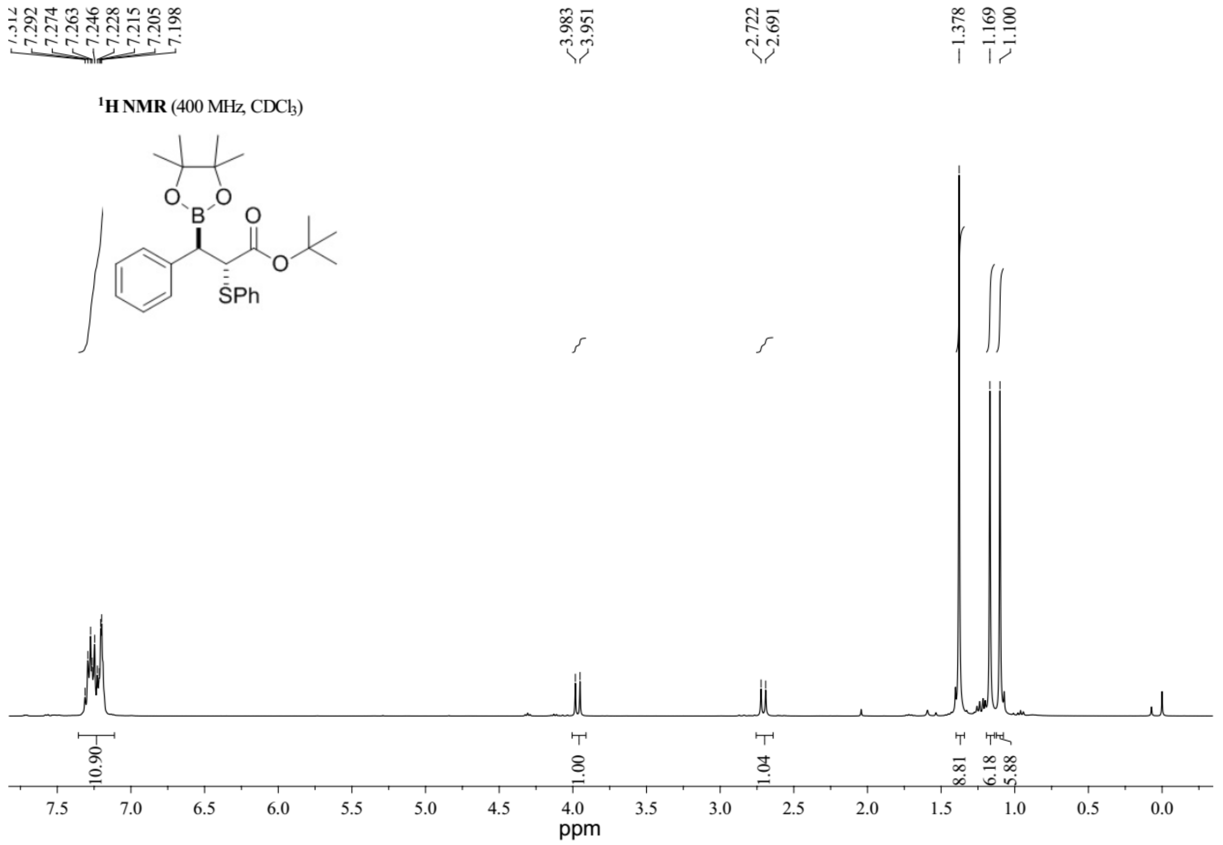

Supplementary Figure 164. **^{13}C NMR spectrum for 7v**

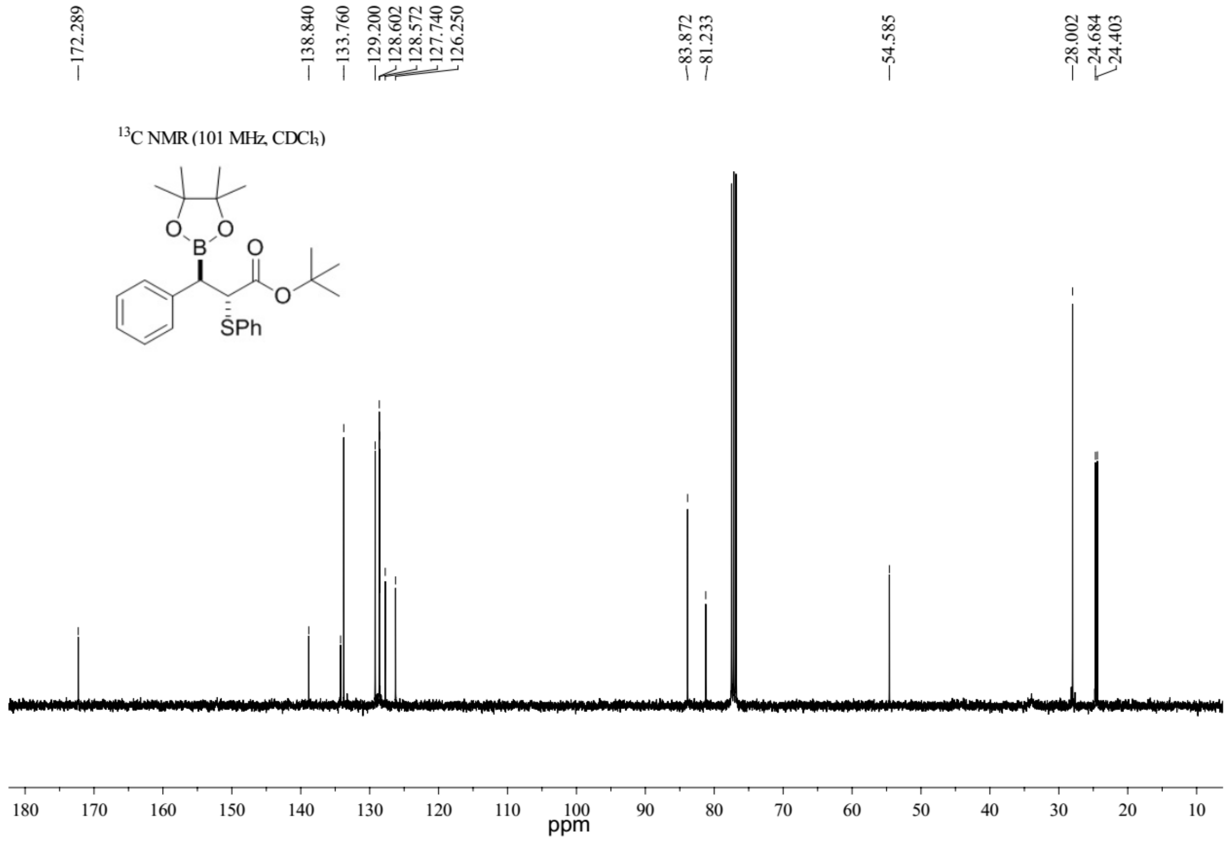

Supplementary Figure 165. ^{1}H NMR spectrum for *syn*-Pentan-3-yl-3-phenyl-2-(phenylthio)-3-(4,4,5,5-tetramethyl-1,3,2-dioxaborolan-2-yl)propanoate (8v)

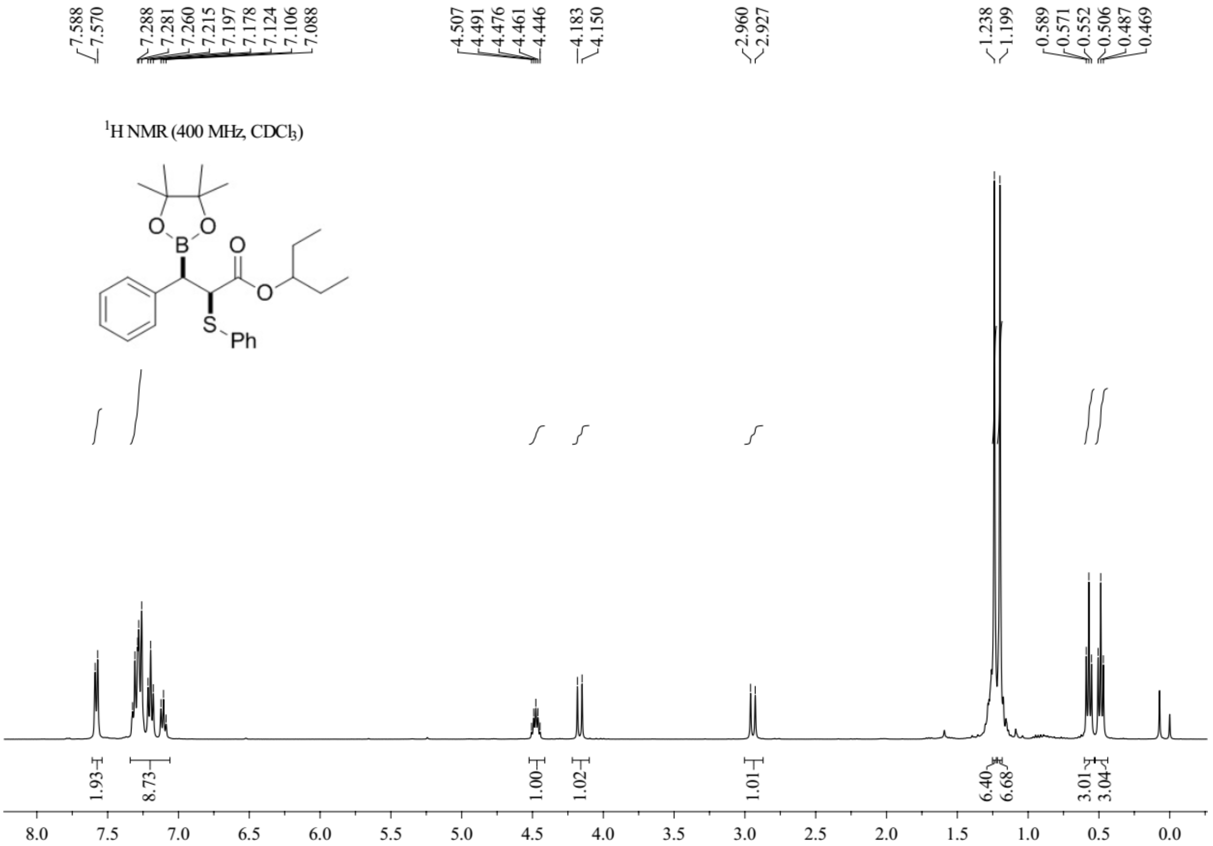

Supplementary Figure 166. ^{13}C NMR spectrum for 8v

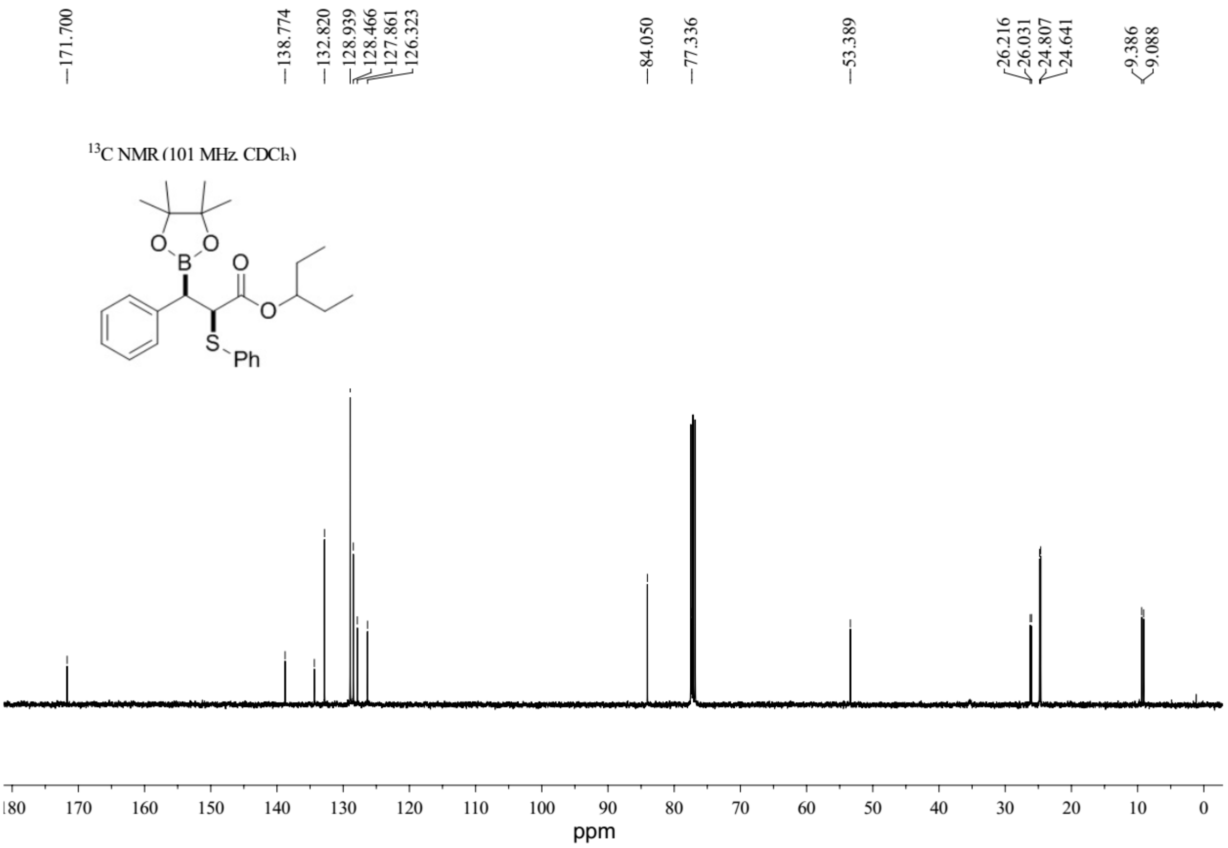

Supplementary Figure 167. ^{1}H NMR spectrum for *anti-N,N*-Diethyl-2-(phenyl(4,4,5,5-tetramethyl-1,3,2-dioxaborolan-2-yl)methyl)pentanamide (7w)

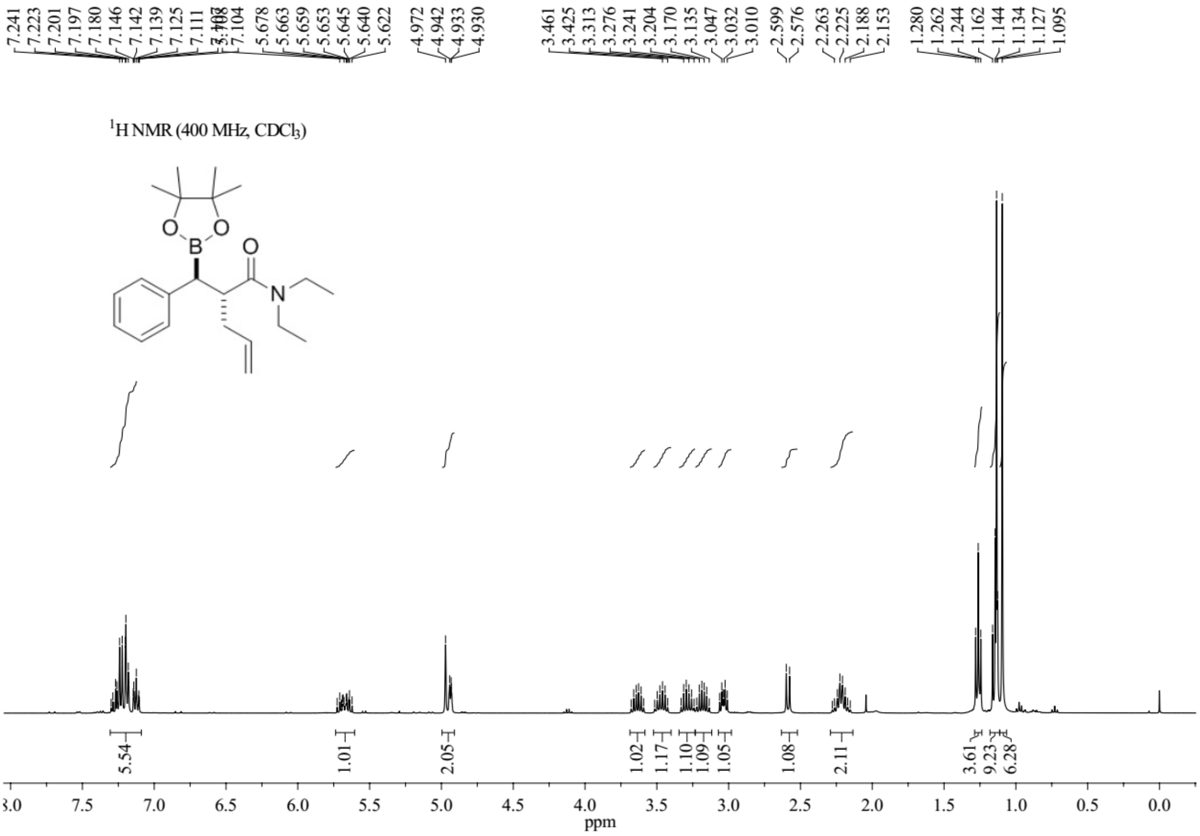

Supplementary Figure 168. ^{13}C NMR spectrum for 7w

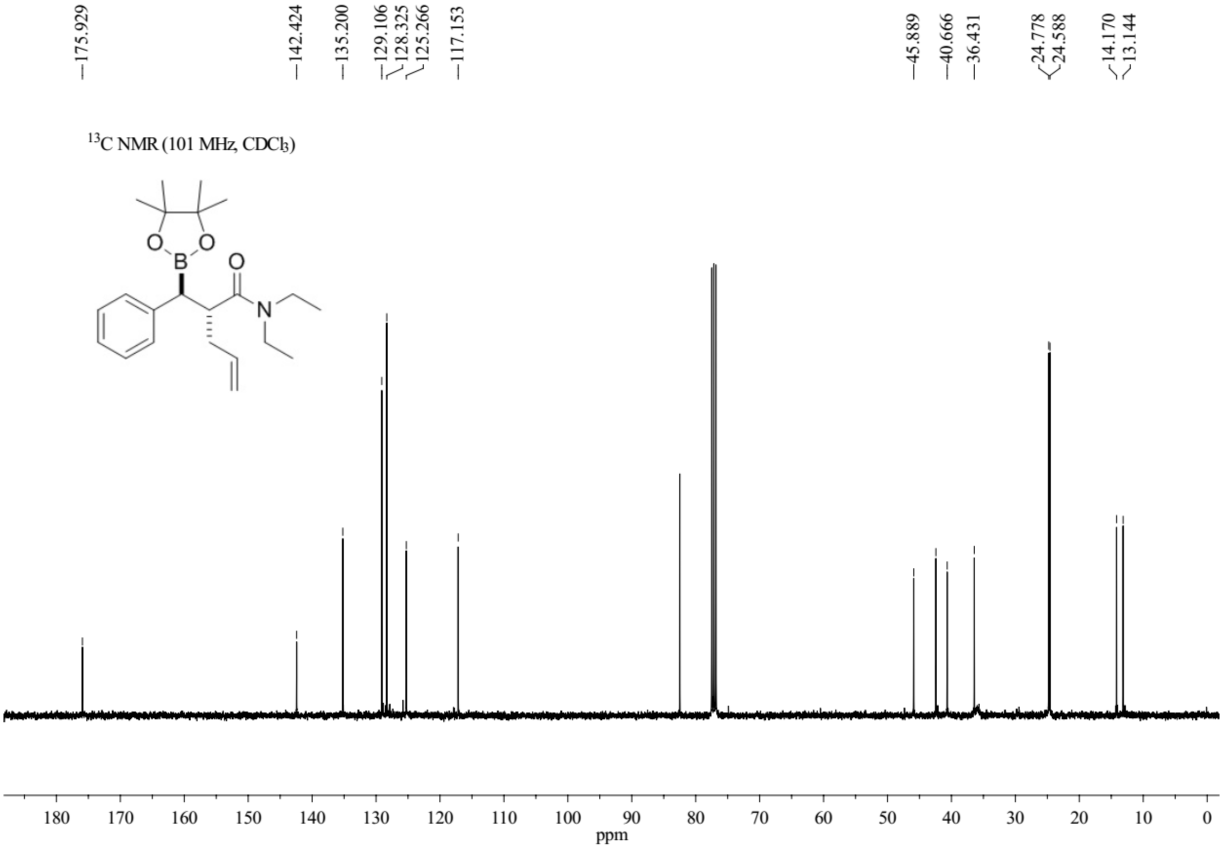

Supplementary Figure 169. ^{1}H NMR spectrum for *anti*-*N*,*N*-Diethyl-2-methyl-3-phenyl-3-(4,4,5,5-tetramethyl-1,3,2-dioxaborolan-2-yl)propanamide (7x)

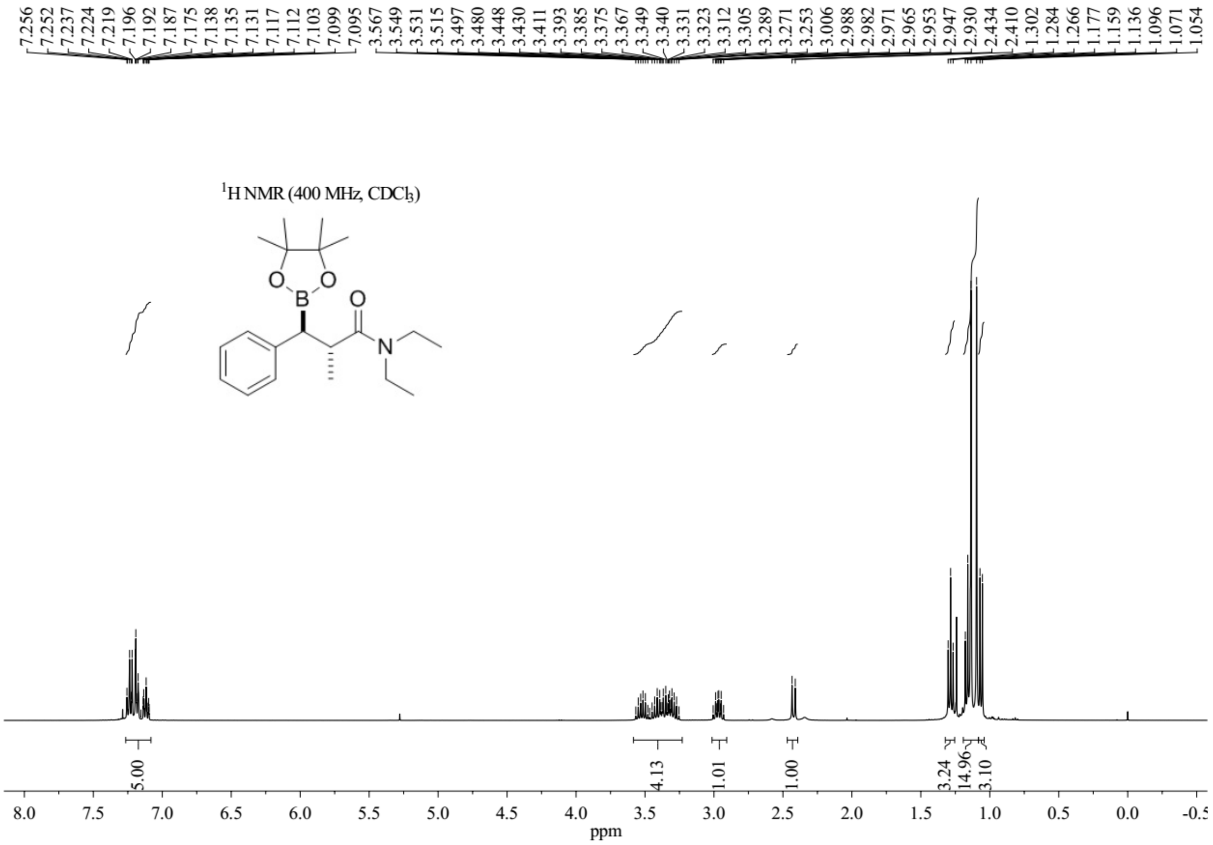

Supplementary Figure 170. ^{13}C NMR spectrum for 7x

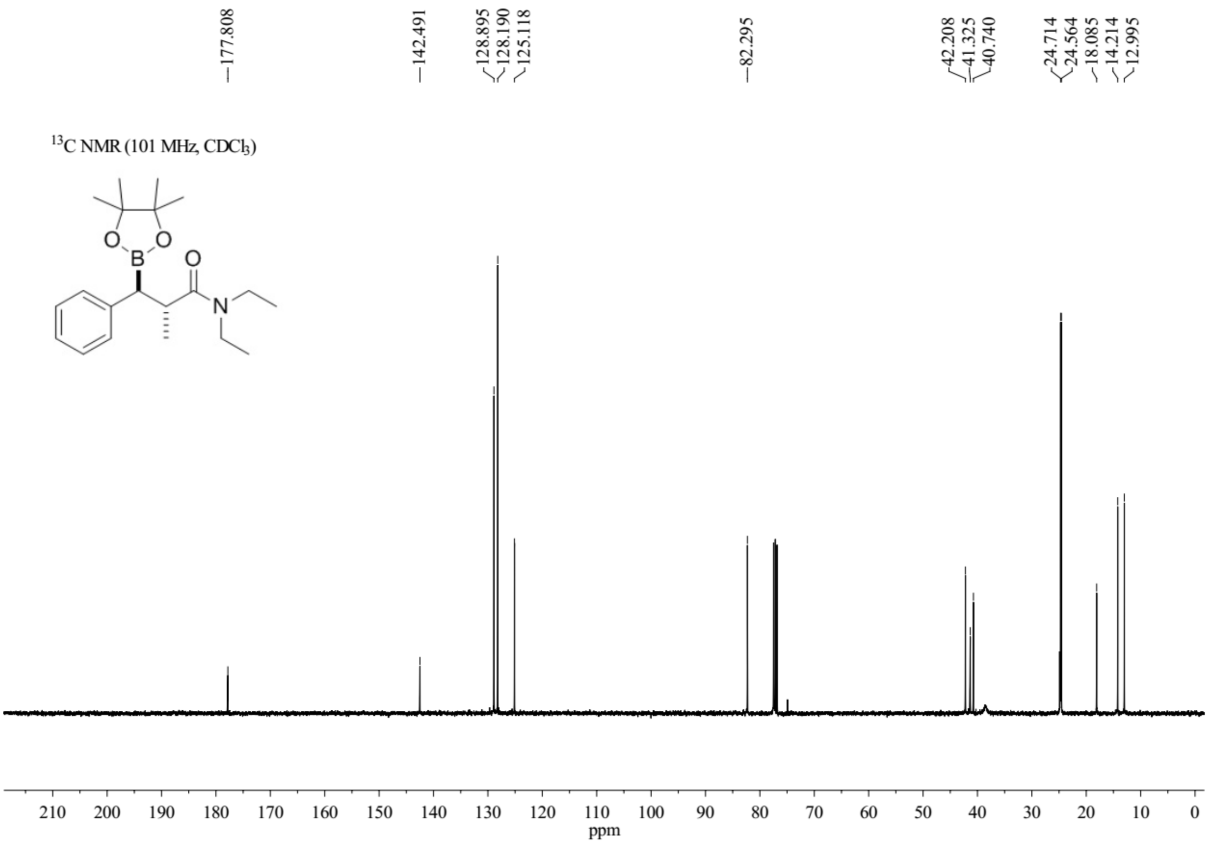

Supplementary Figure 171. ^{1}H NMR spectrum for *anti-tert*-Butyl-2-methyl-2-(phenyl(4,4,5,5-tetramethyl-1,3,2-dioxaborolan-2-yl)methyl)pent-4-enoate (9)

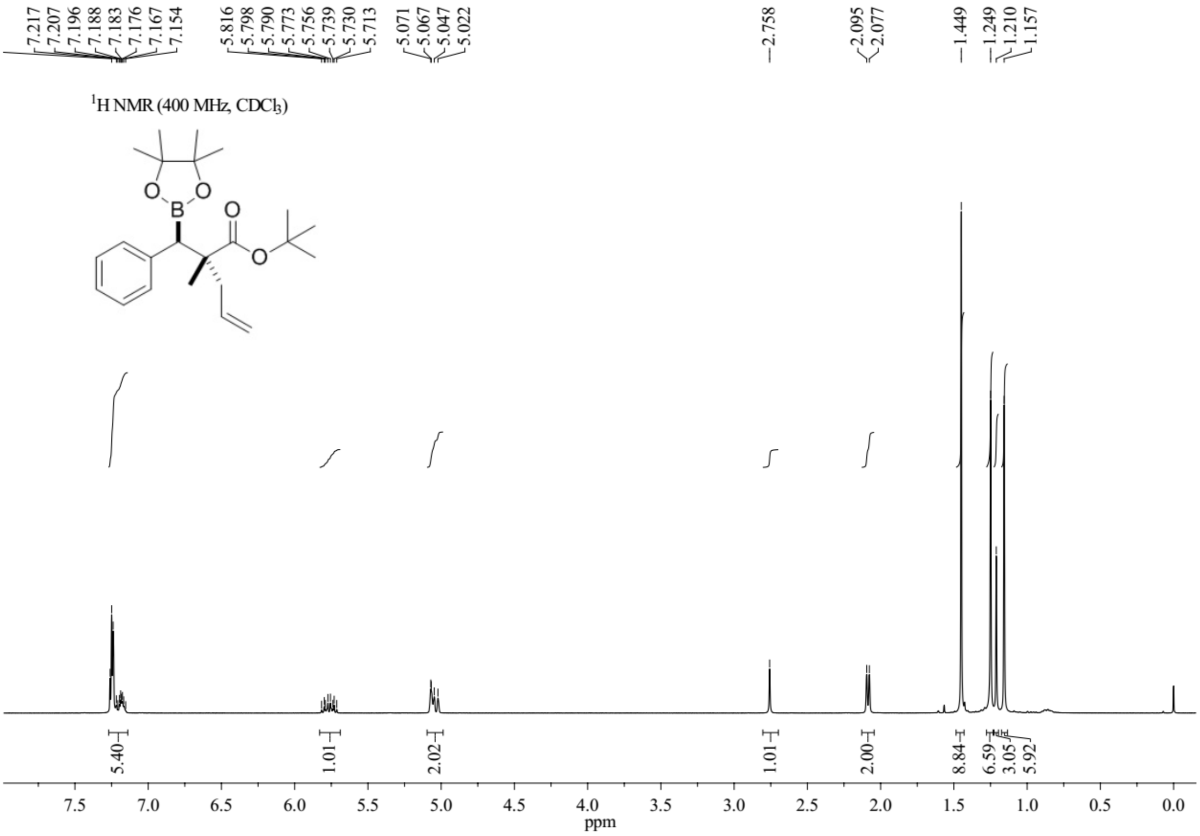

Supplementary Figure 172. ^{13}C NMR spectrum for 9

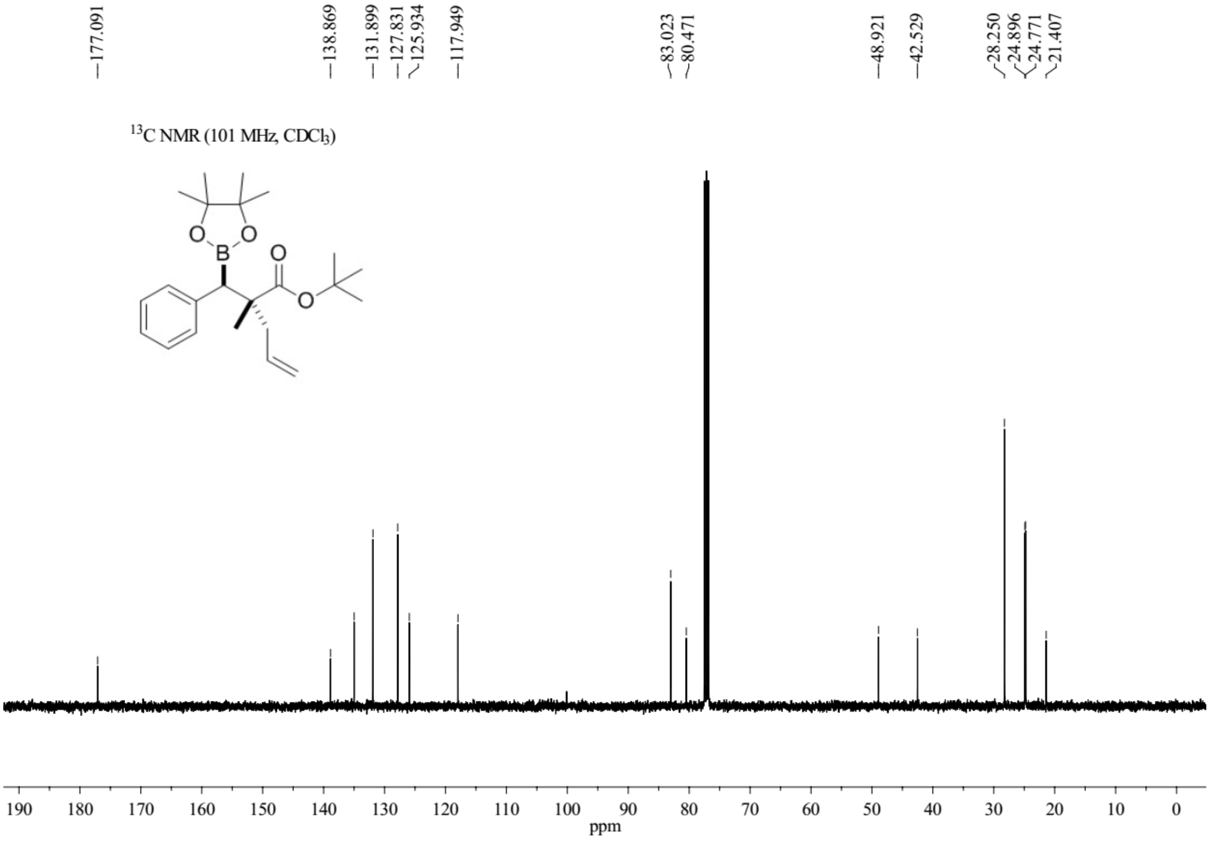

Supplementary Figure 173. ^{1}H NMR spectrum for *anti-tert*-Butyl-2-methyl-2-(phenyl(4,4,5,5-tetramethyl-1,3,2-dioxaborolan-2-yl)methyl)pent-4-enoate (10)

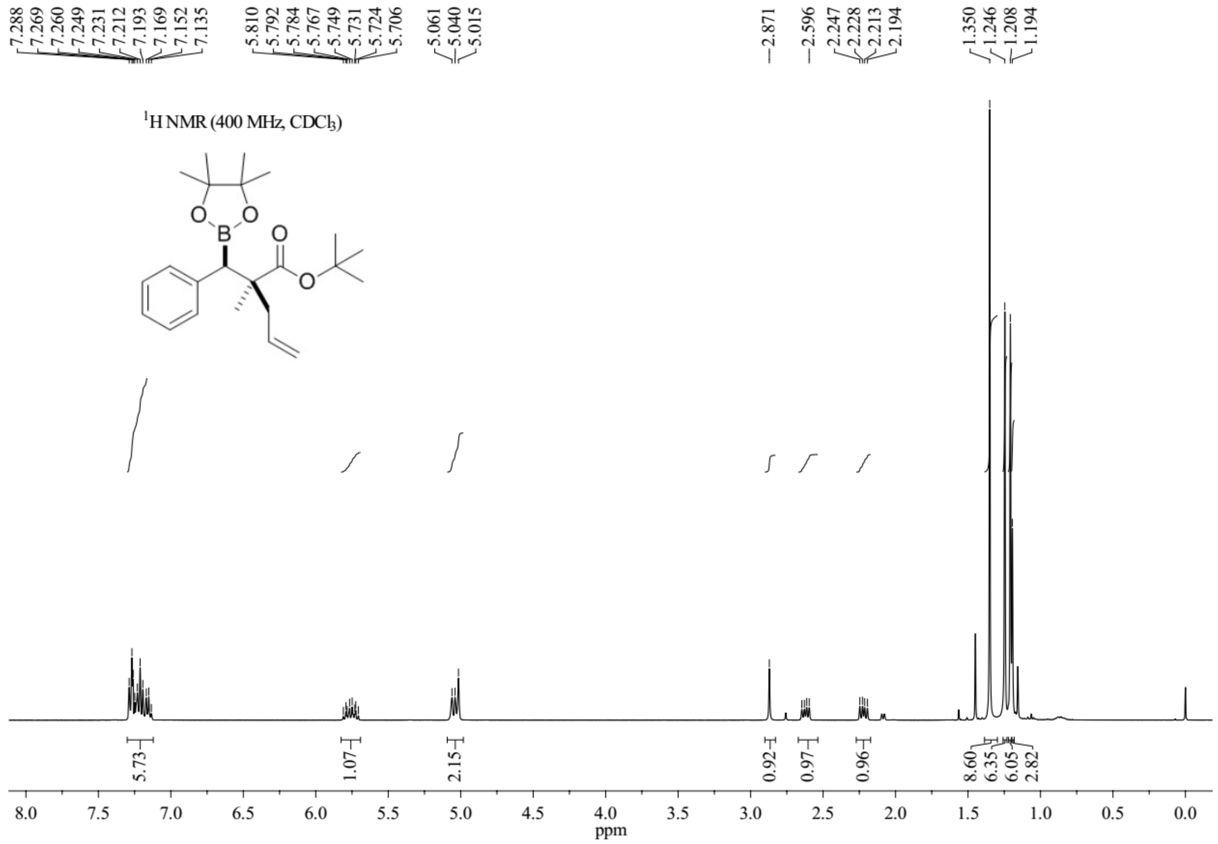

Supplementary Figure 174. ^{13}C NMR spectrum for 10

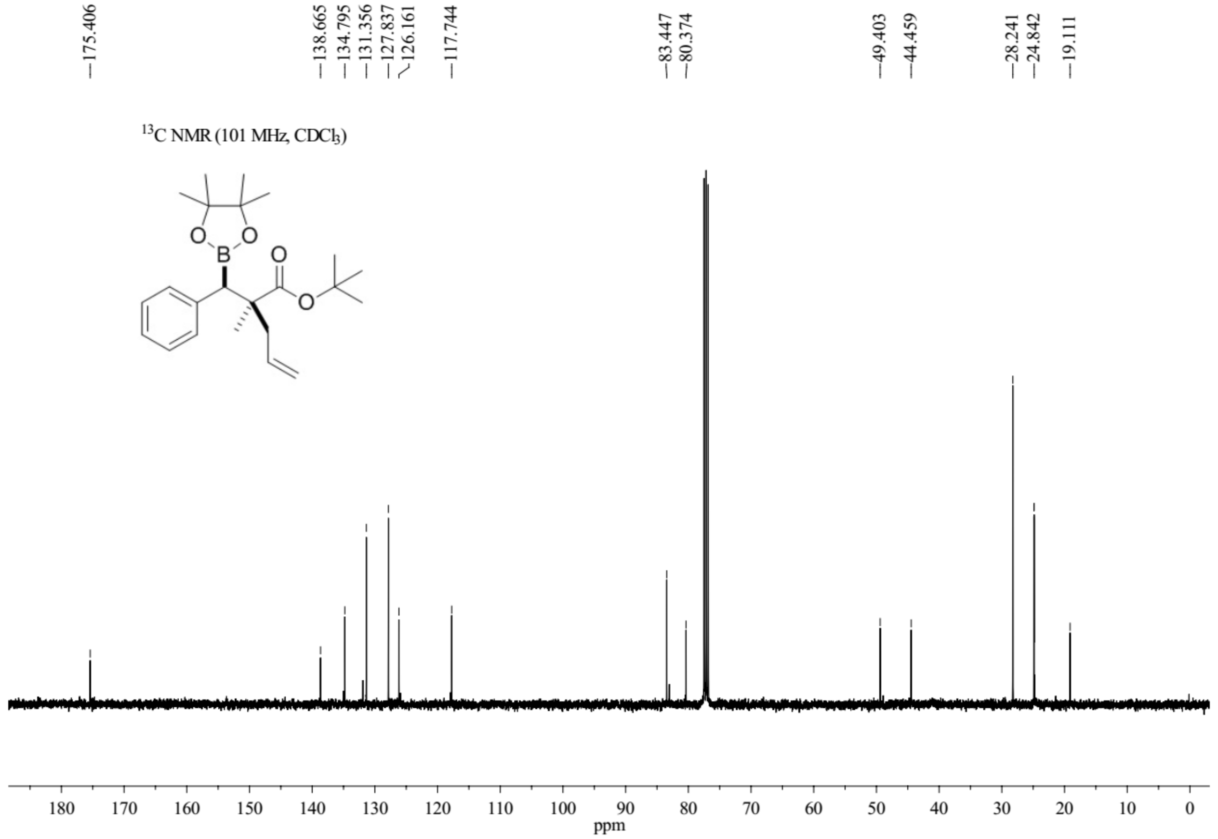

Supplementary Figure 175. ^{1}H NMR spectrum for *anti-tert*-Butyl 2-(hydroxy(phenyl)methyl)pent-4-enoate (3ca)

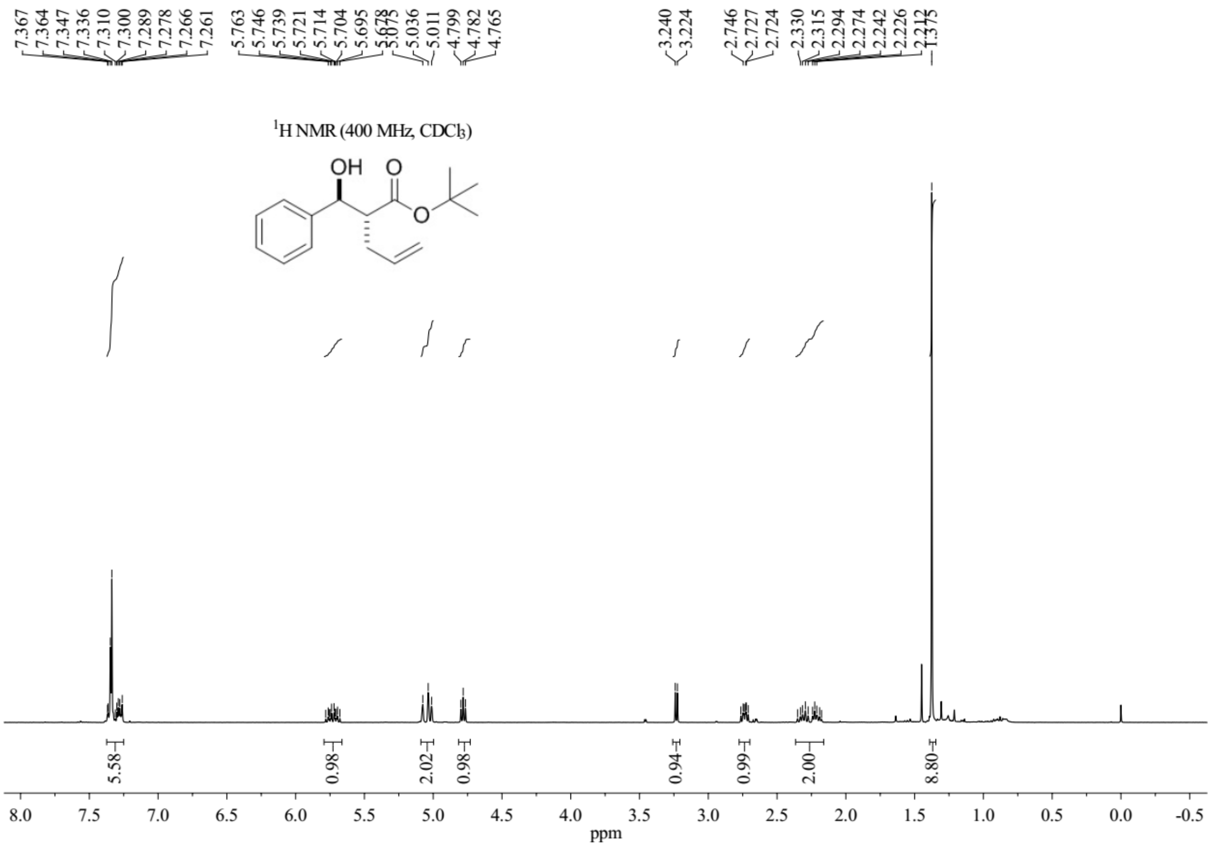

Supplementary Figure 176. ^{13}C NMR spectrum for 3ca

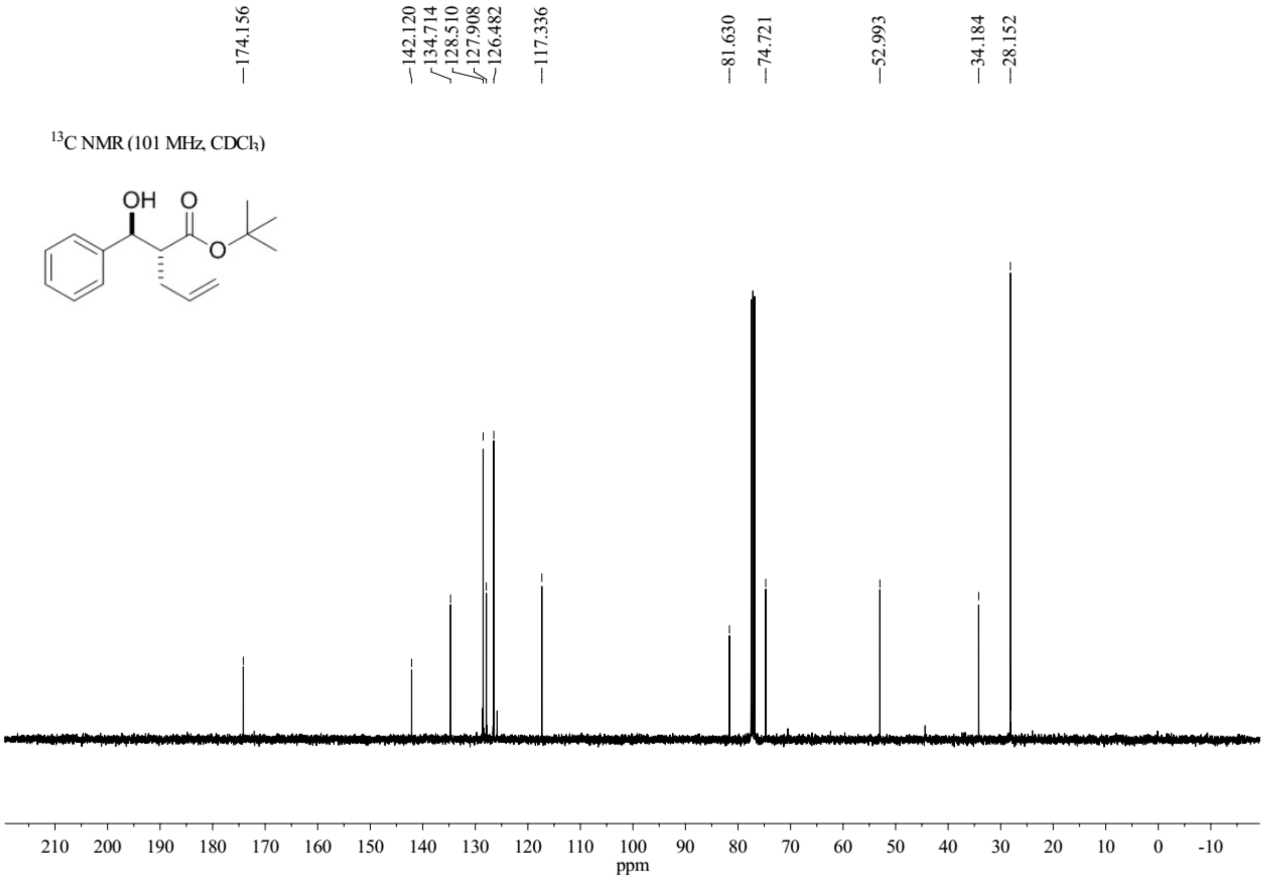

Supplementary Figure 177. ^{1}H NMR spectrum for *anti*-2-allyl-1-phenylpropane-1,3-diol (11)

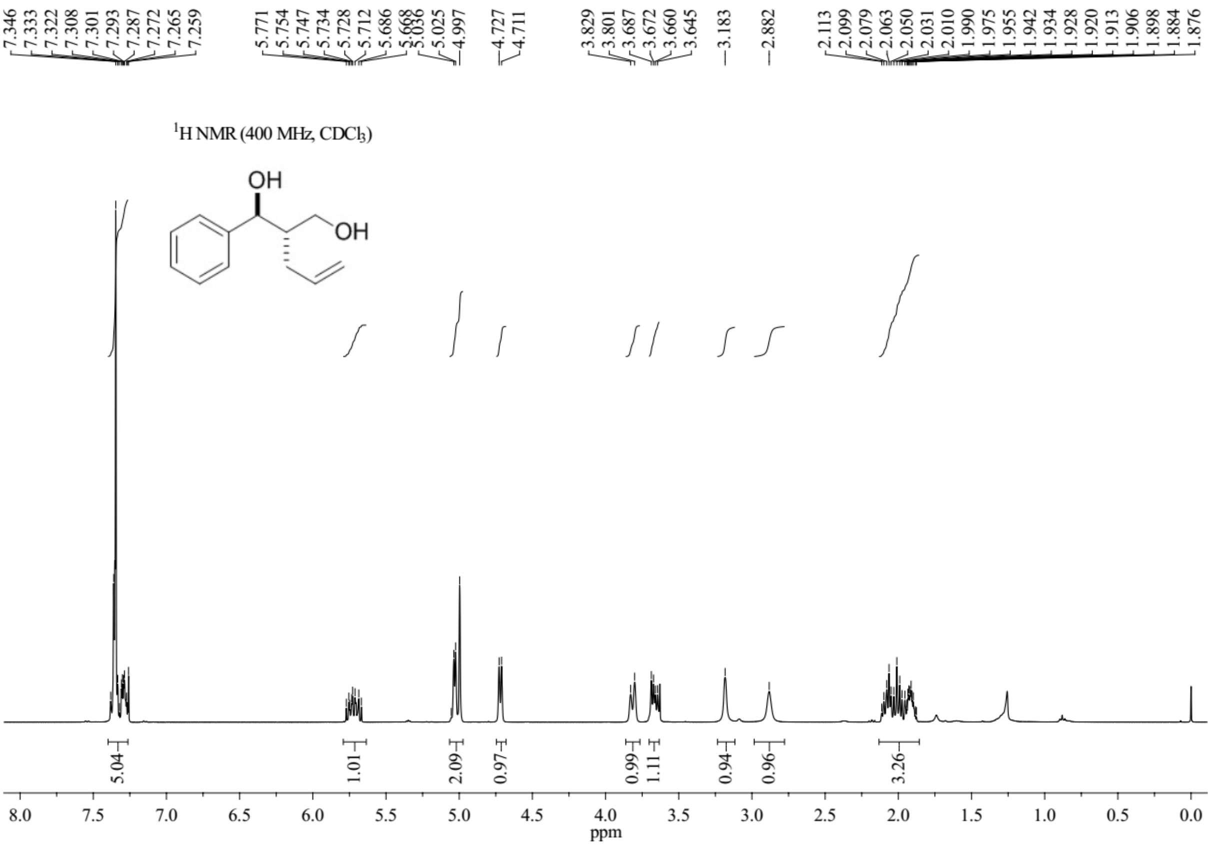

Supplementary Figure 178. ^{13}C NMR spectrum for 11

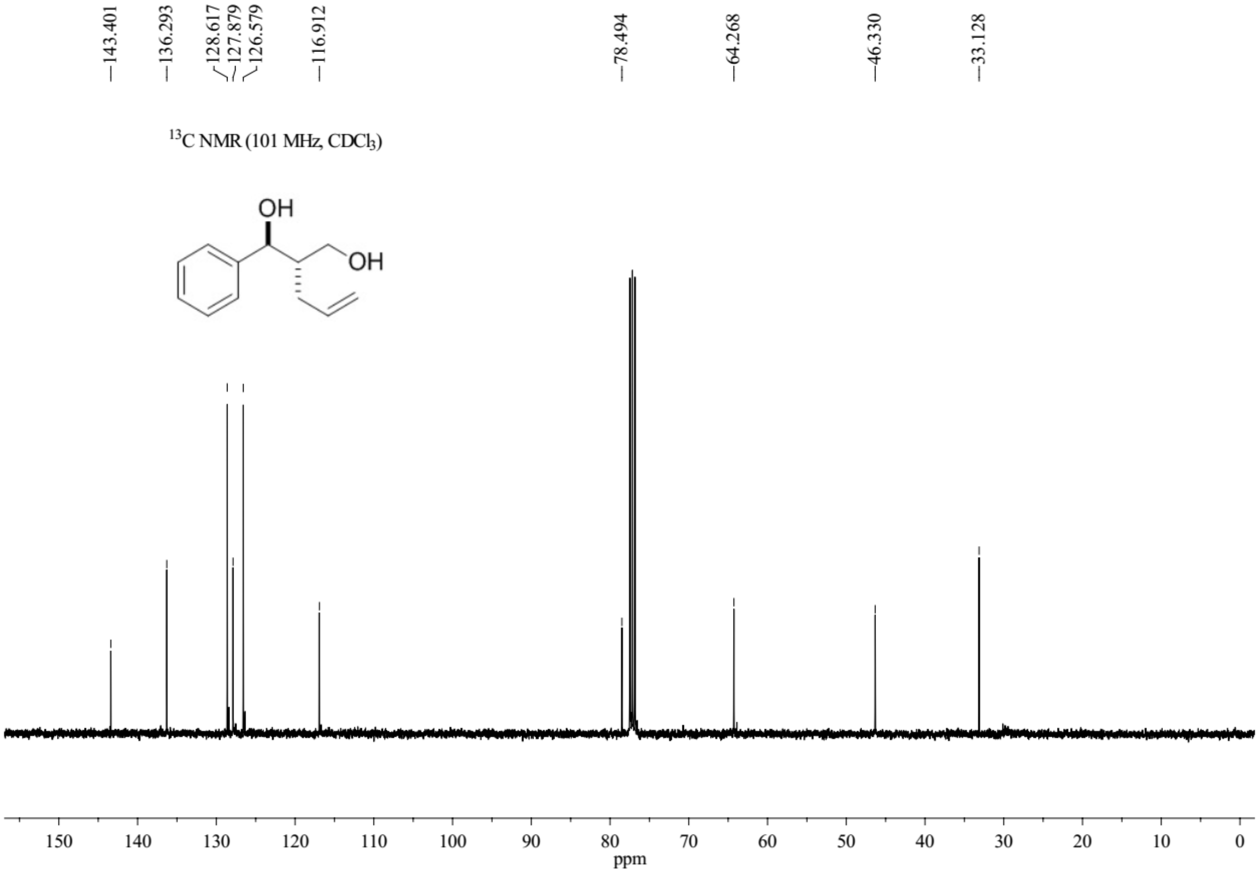

Supplementary Figure 179. ^{1}H NMR spectrum for *syn*-Pentan-3-yl 2-(hydroxy(phenyl)methyl)pent-4-enoate (4ba)

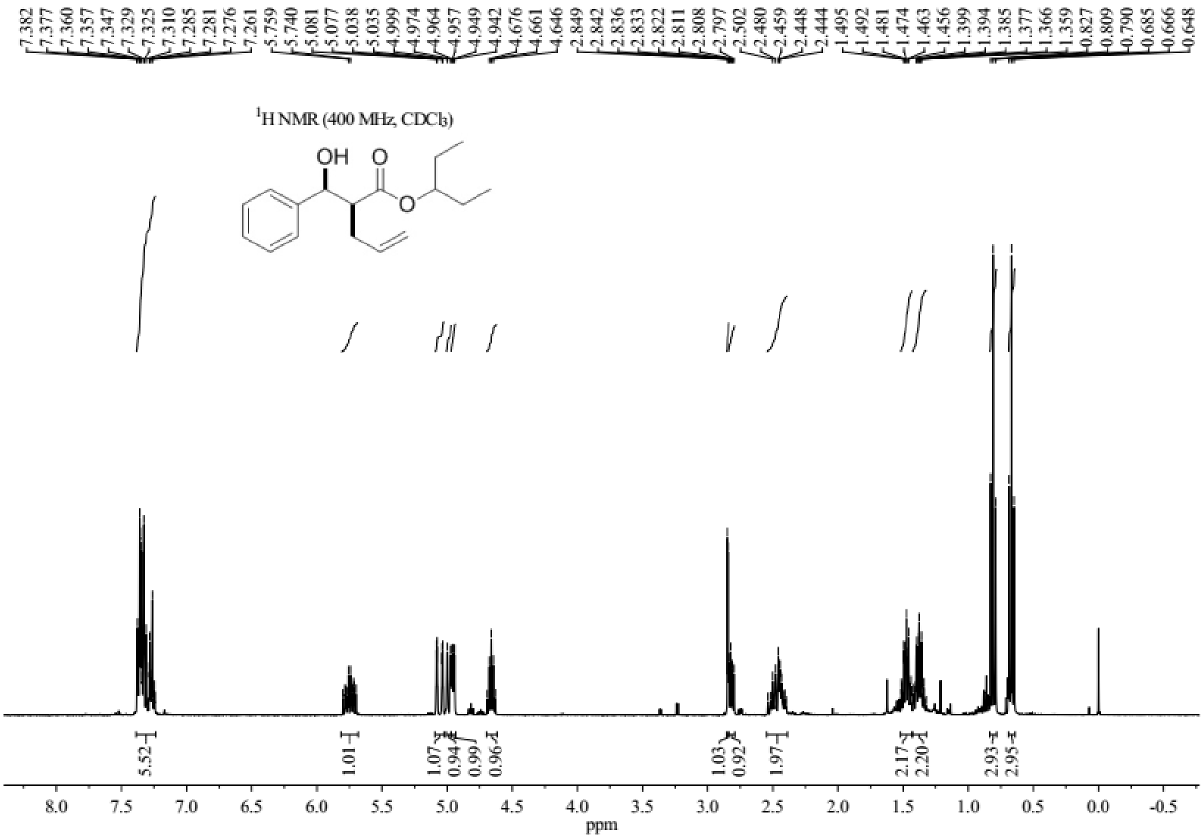

Supplementary Figure 180. ^{13}C NMR spectrum for 4ba

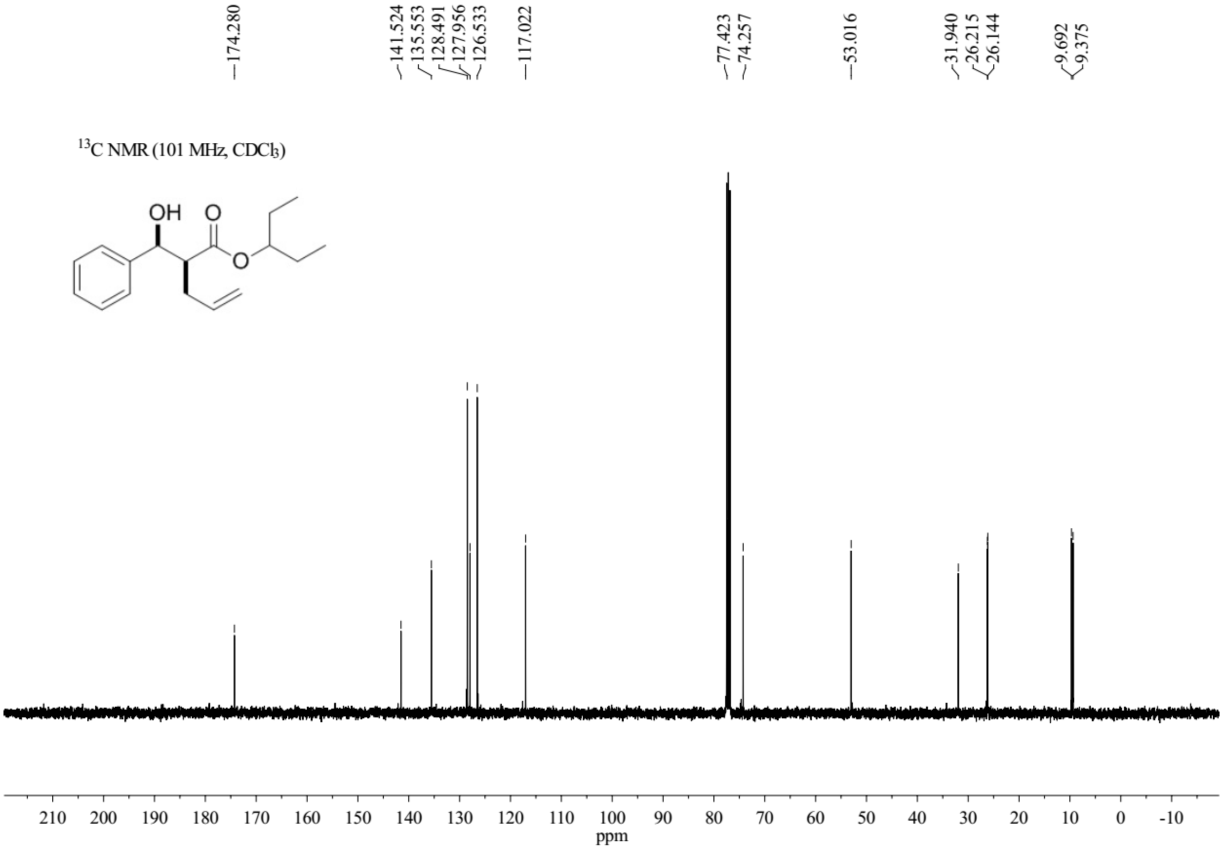

Supplementary Figure 181. ^{1}H NMR spectrum for *syn*-2-allyl-1-phenylpropane-1,3-diol (12)

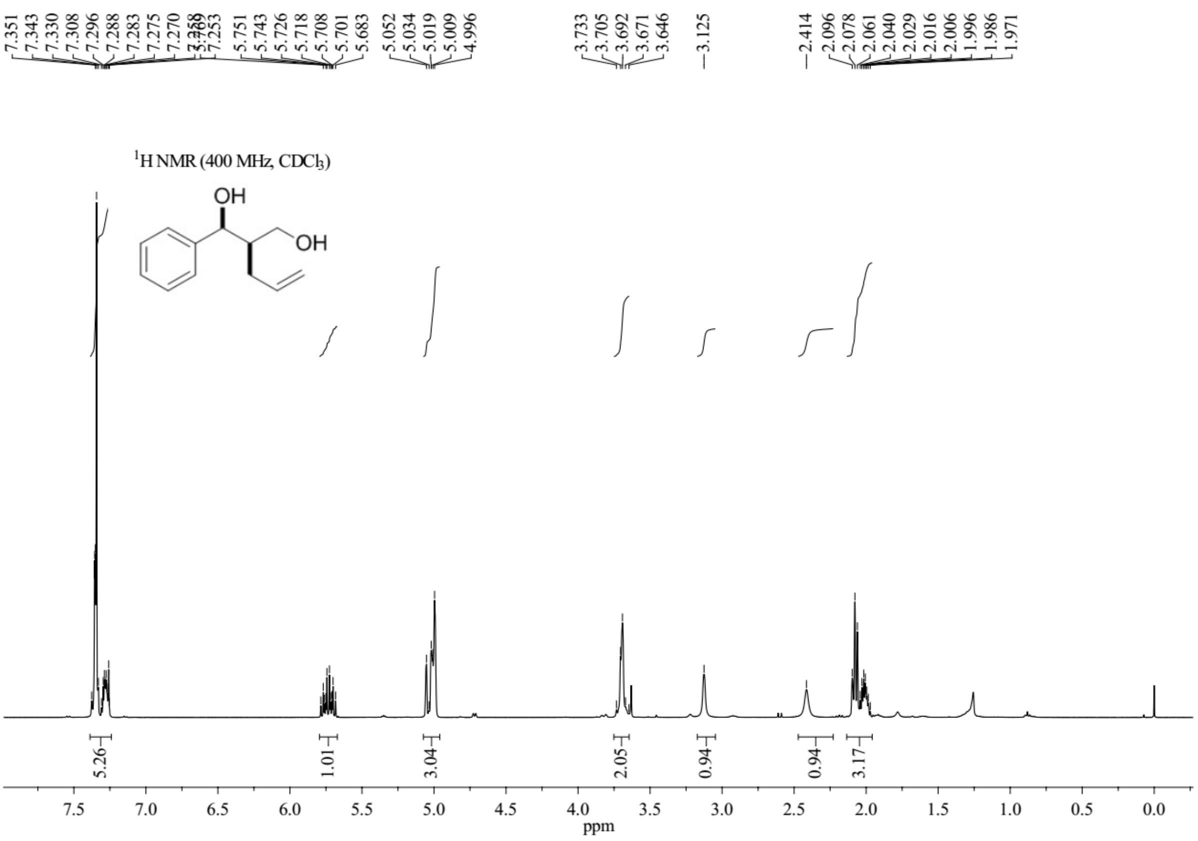

Supplementary Figure 182. ^{13}C NMR spectrum for 12

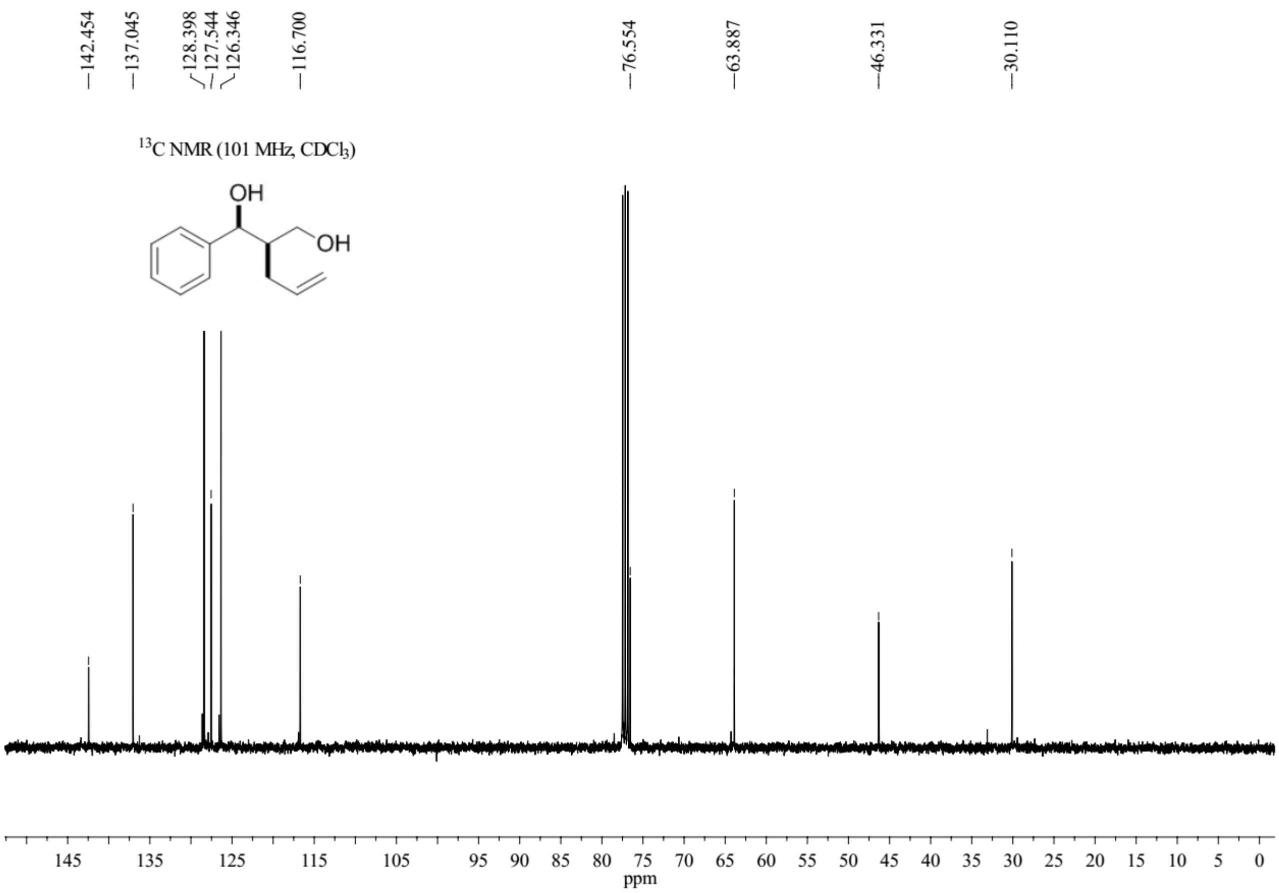

Supplementary Figure 183. ^{1}H NMR spectrum for *syn*-Pentan-3-yl 2-(2-hydroxy-1-phenylethyl)pent-4-enoate (13)

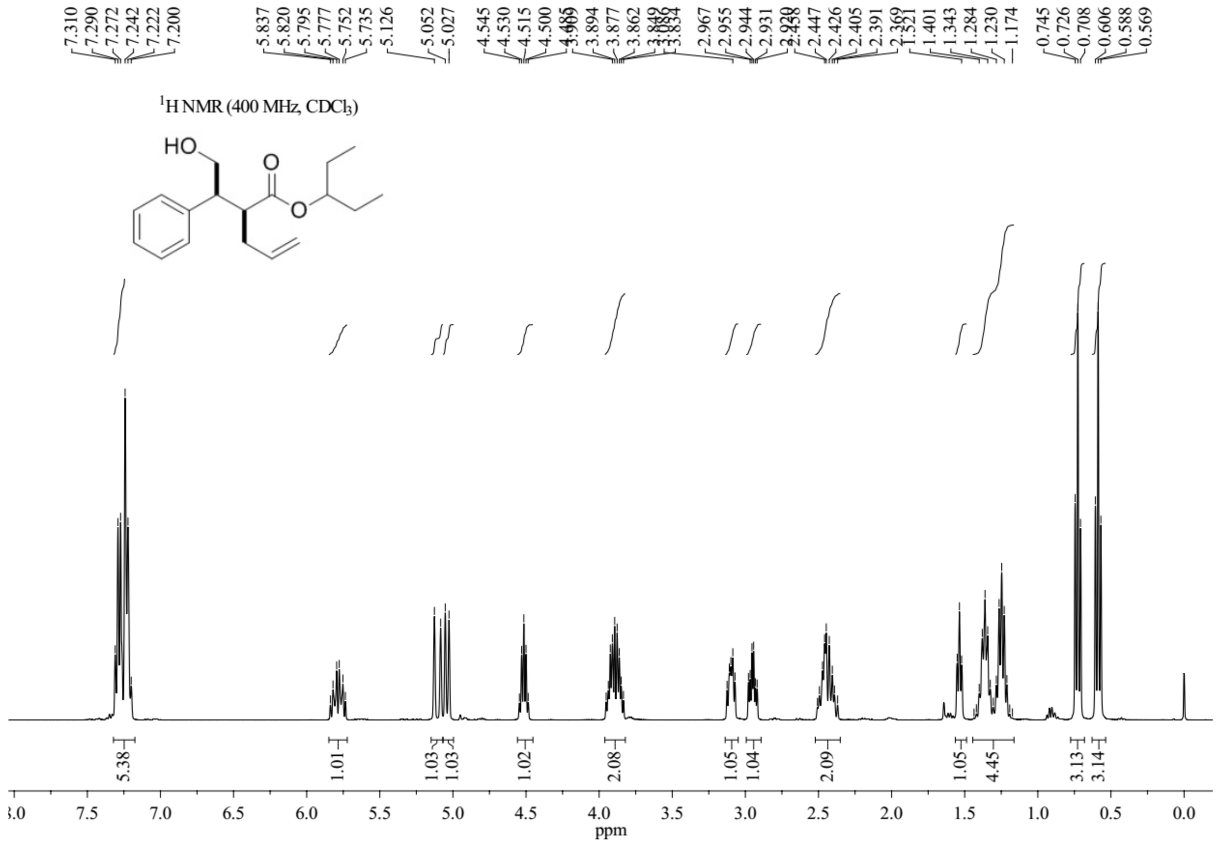

Supplementary Figure 184. ^{13}C NMR spectrum for 13

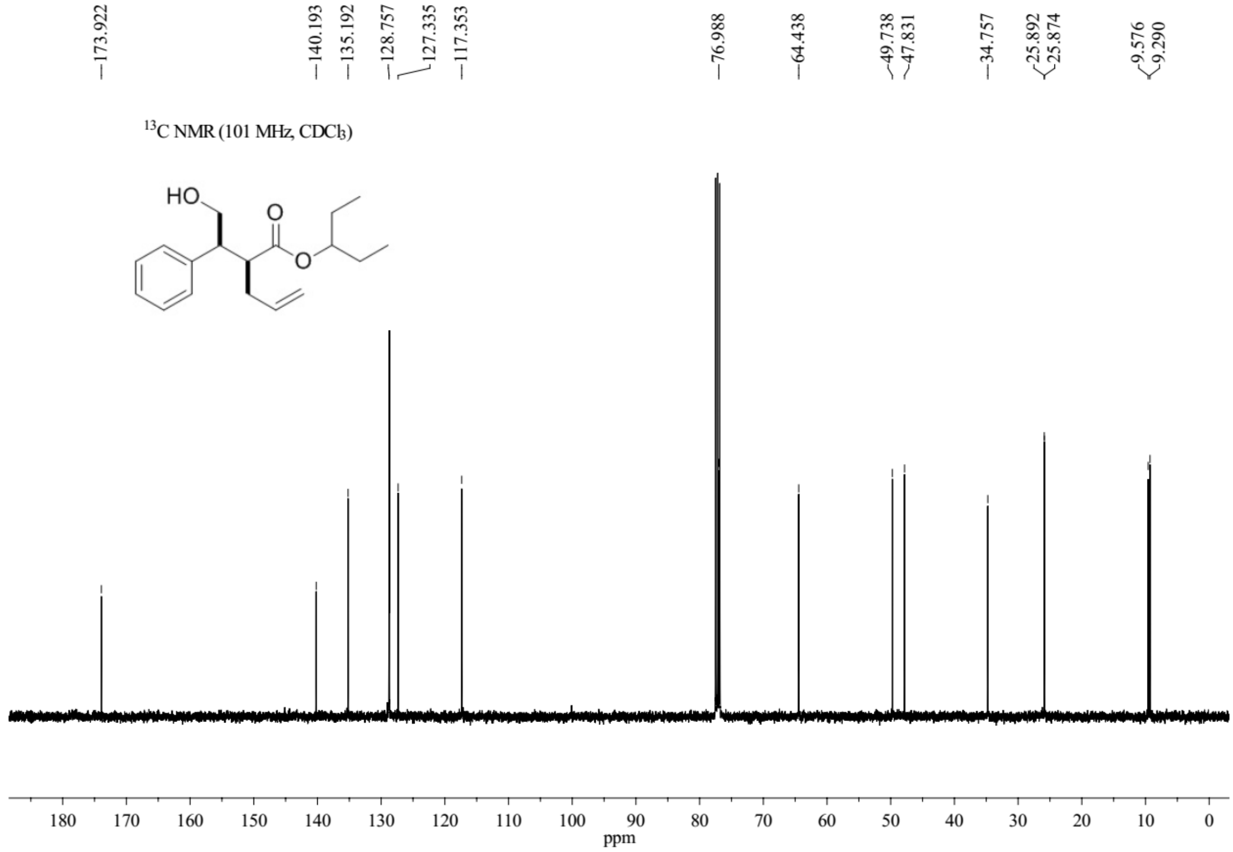

Supplementary Figure 185. ^{1}H NMR spectrum for *syn*-Pentan-3-yl 2-(phenyl(thiophen-2-yl)methyl)pent-4-enoate (14)

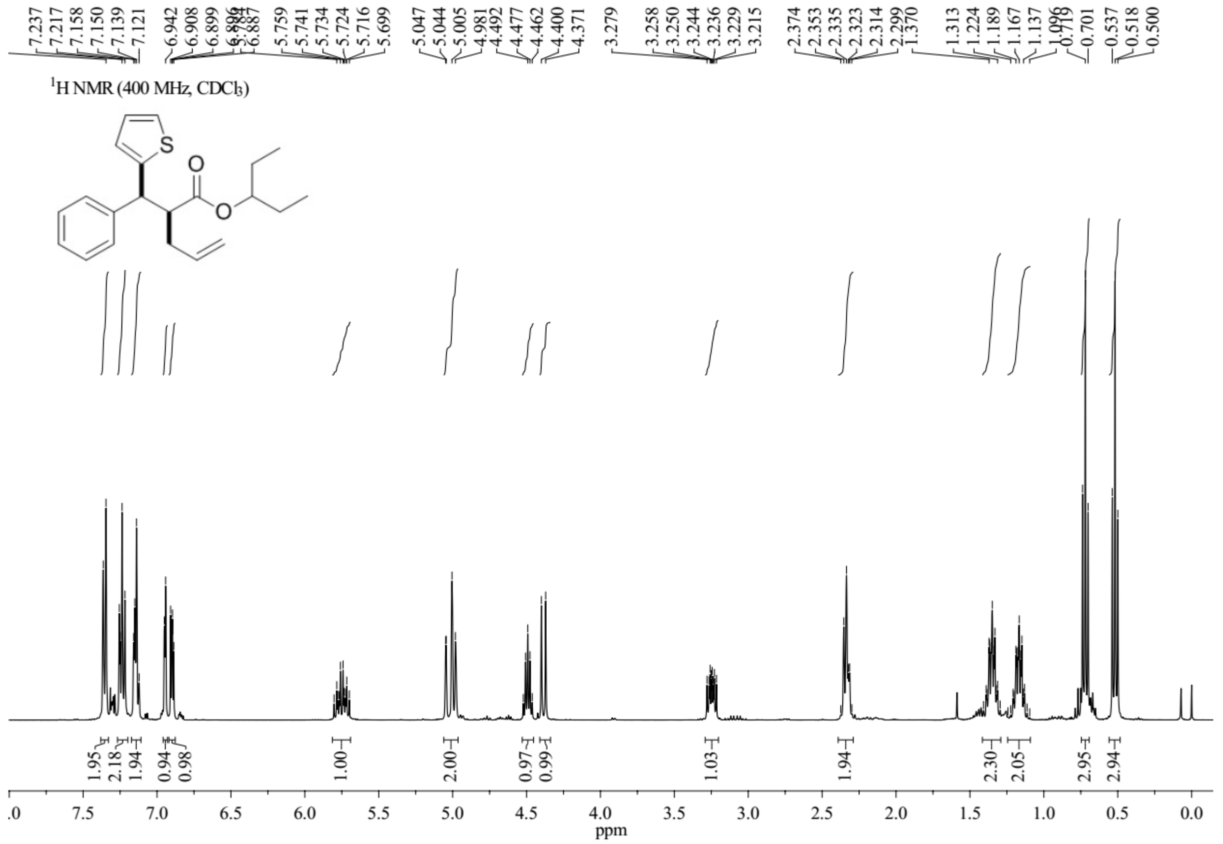

Supplementary Figure 186. ^{13}C NMR spectrum for 14

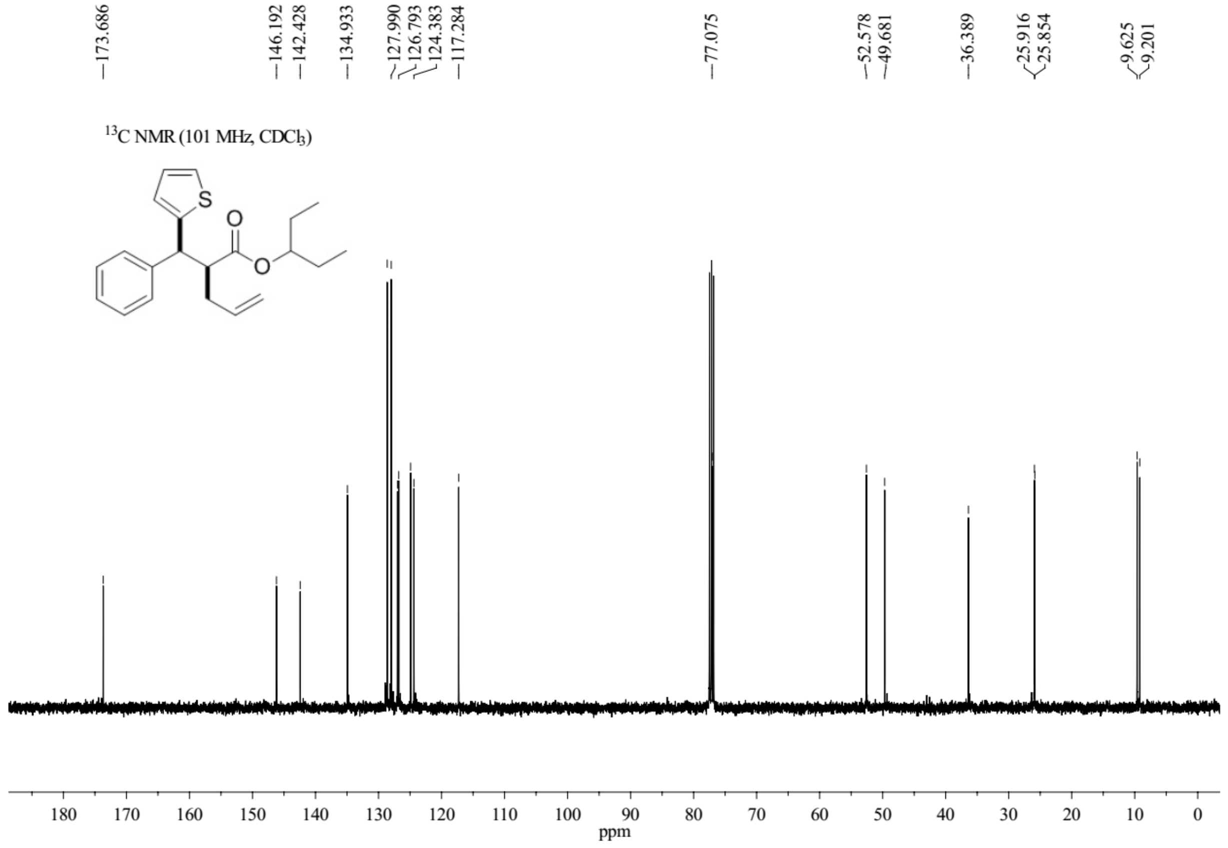

Supplementary Figure 187. ^{1}H NMR spectrum for *syn*-Pentan-3-yl 2-(furan-2-yl(phenyl)methyl)pent-4-enoate (15)

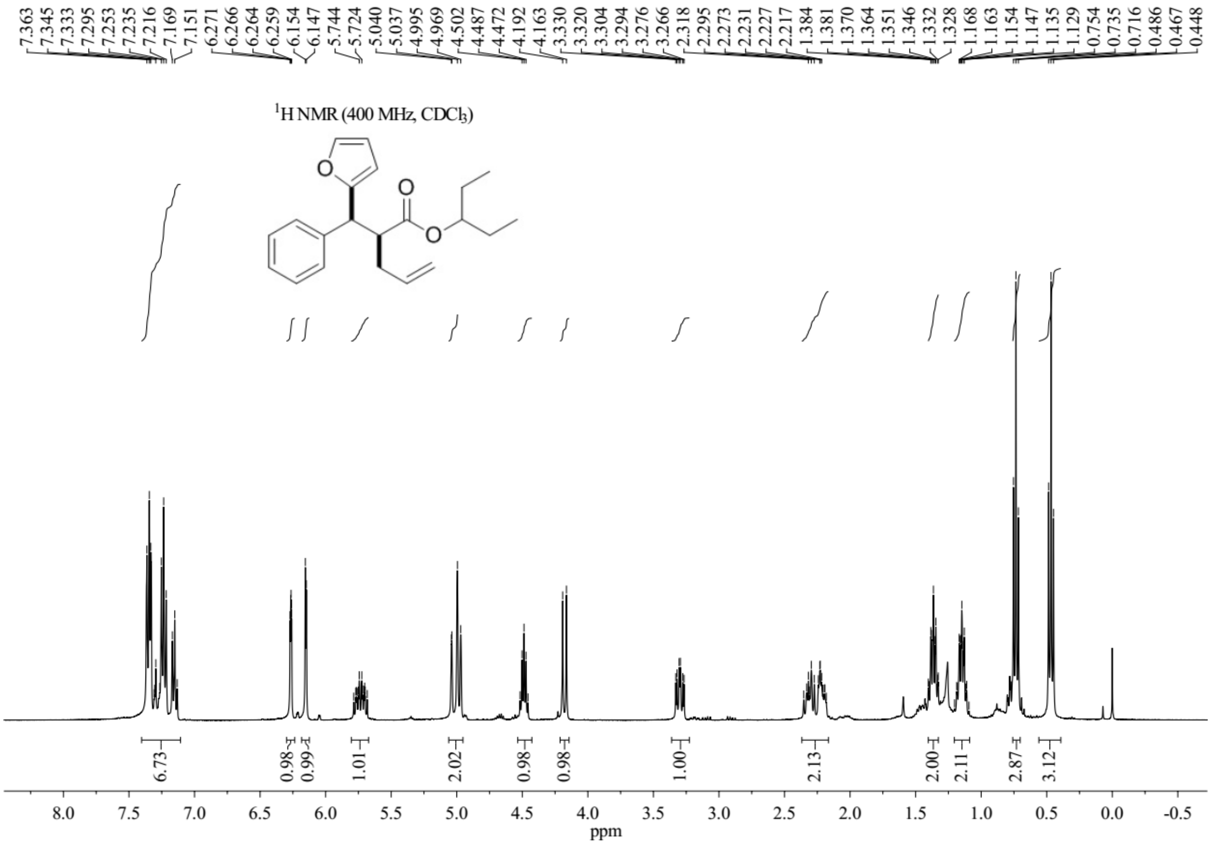

Supplementary Figure 188. ^{13}C NMR spectrum for 15

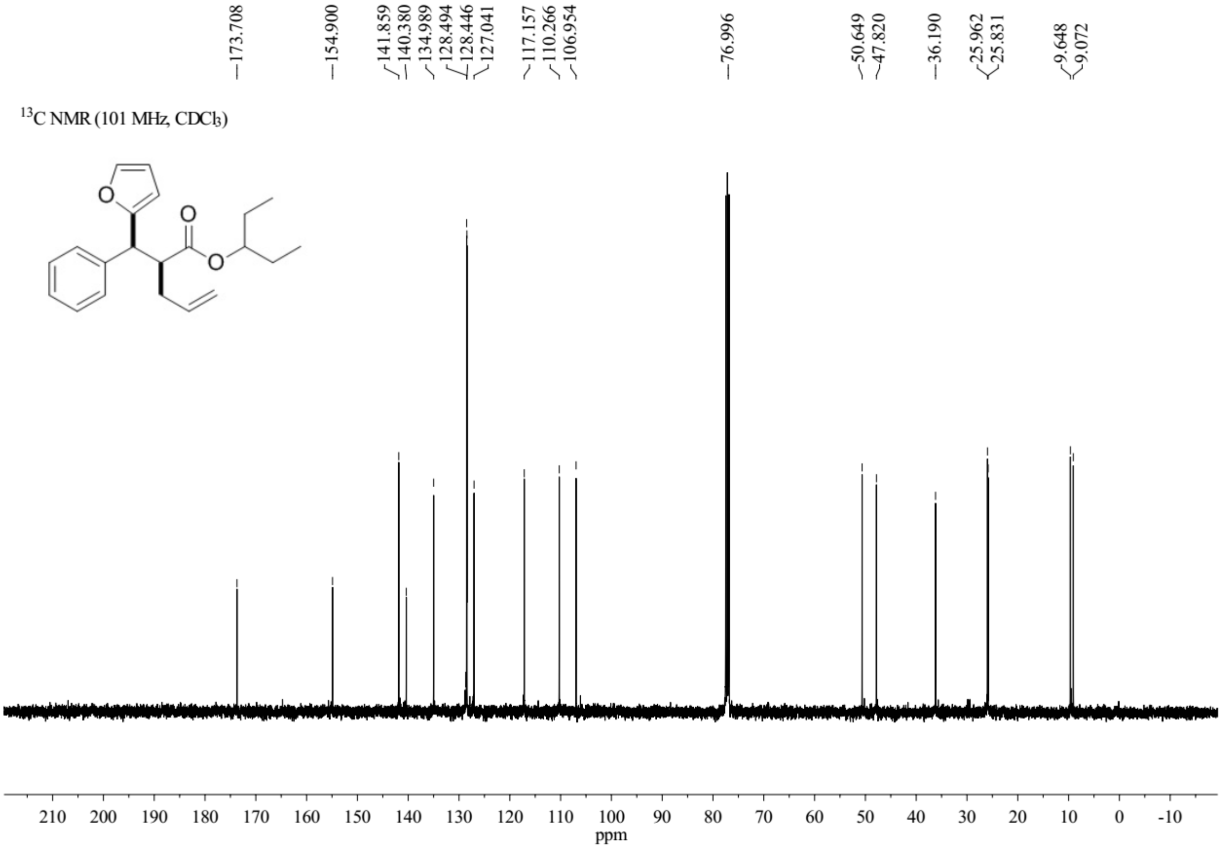

Supplementary Figure 189. ^{1}H NMR spectrum for *syn*-Pentan-3-yl 2-((1-methyl-1H-indol-2-yl)(phenyl)methyl)pent-4-enoate (16)

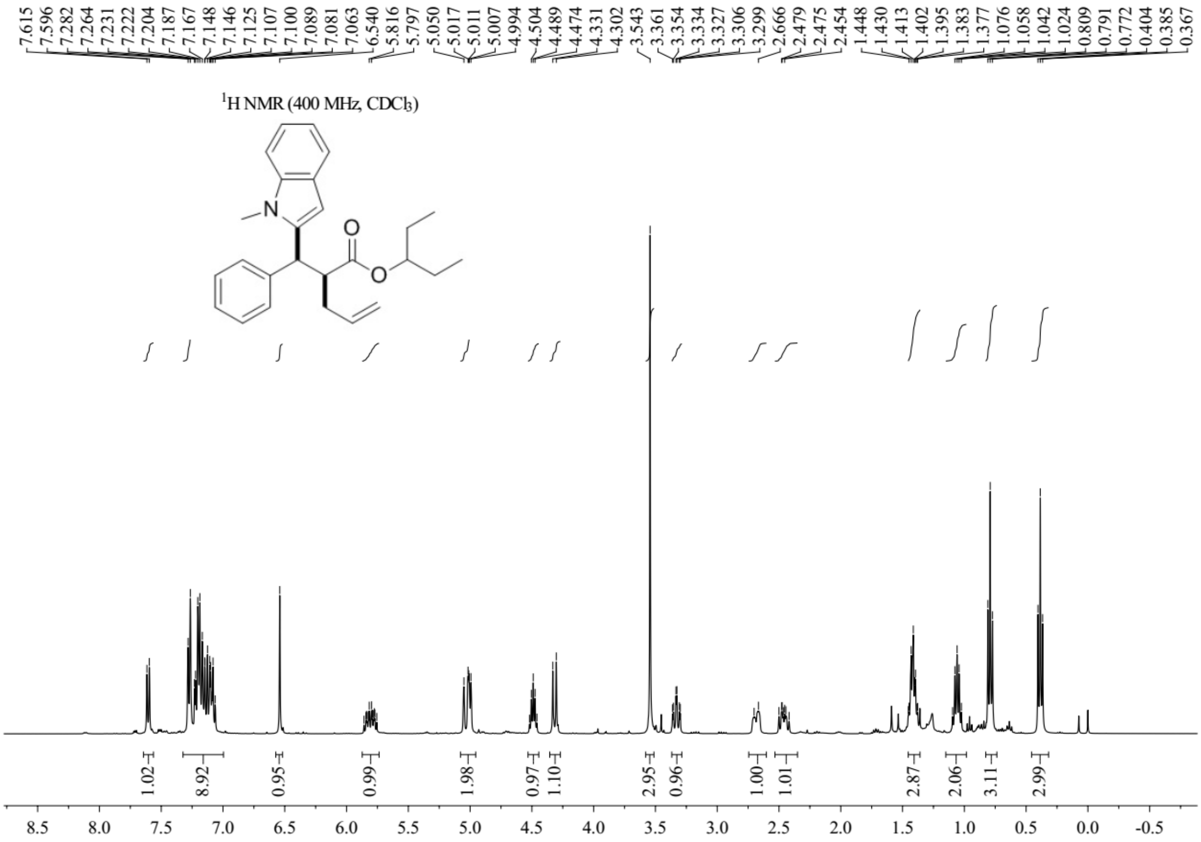

Supplementary Figure 190. ^{13}C NMR spectrum for 16

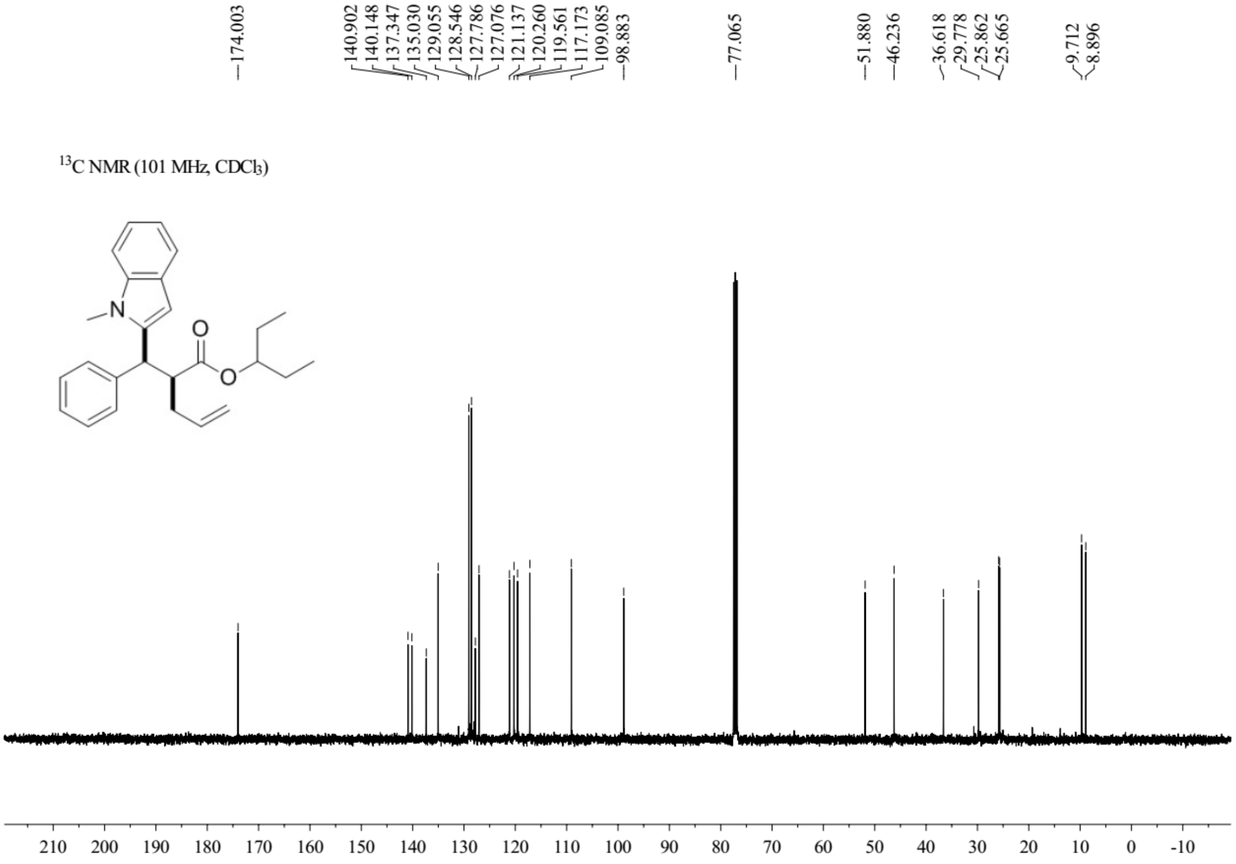

Supplementary Figure 191. ^{1}H NMR spectrum for *syn*-Pentan-3-yl (2*R*,3*S*,*E*)-2-allyl-3-hydroxyhex-4-enoate (17)

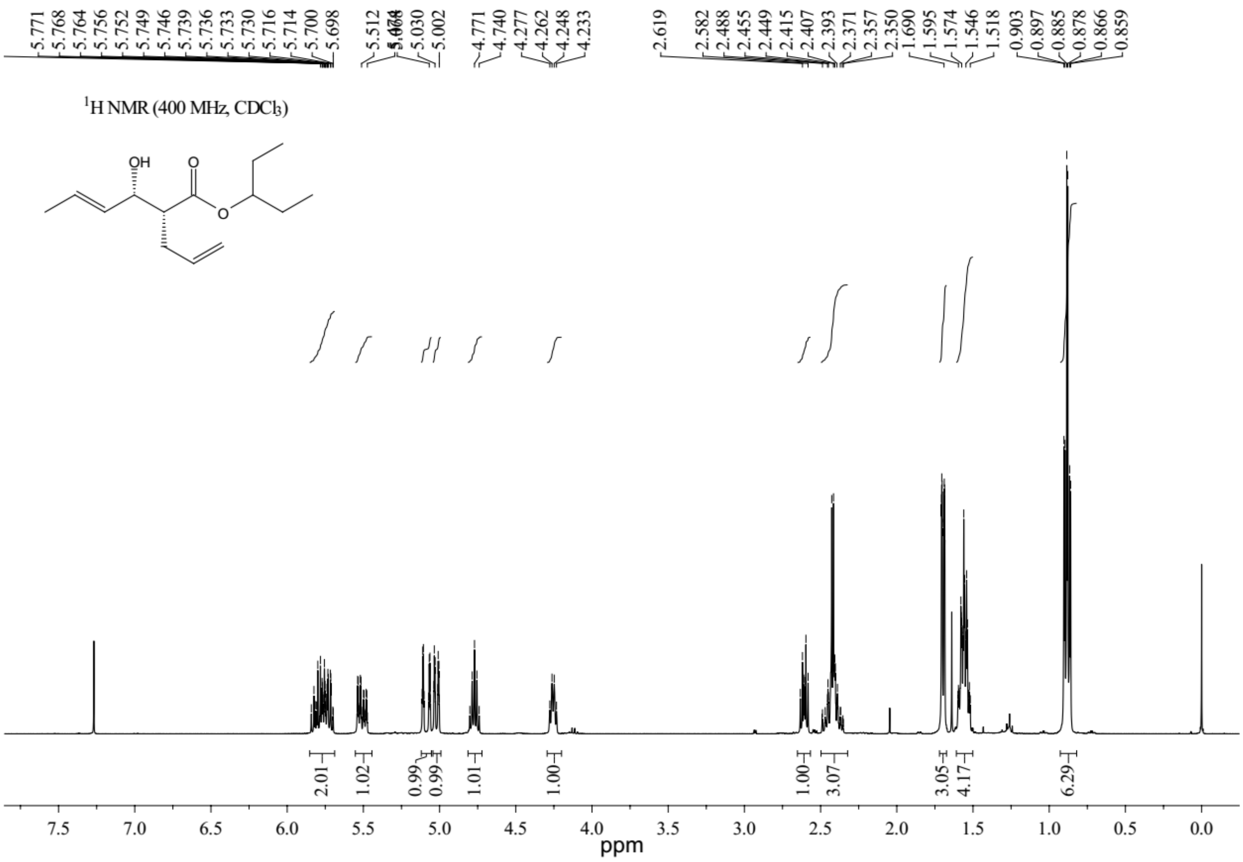

Supplementary Figure 192. ^{13}C NMR spectrum for 17

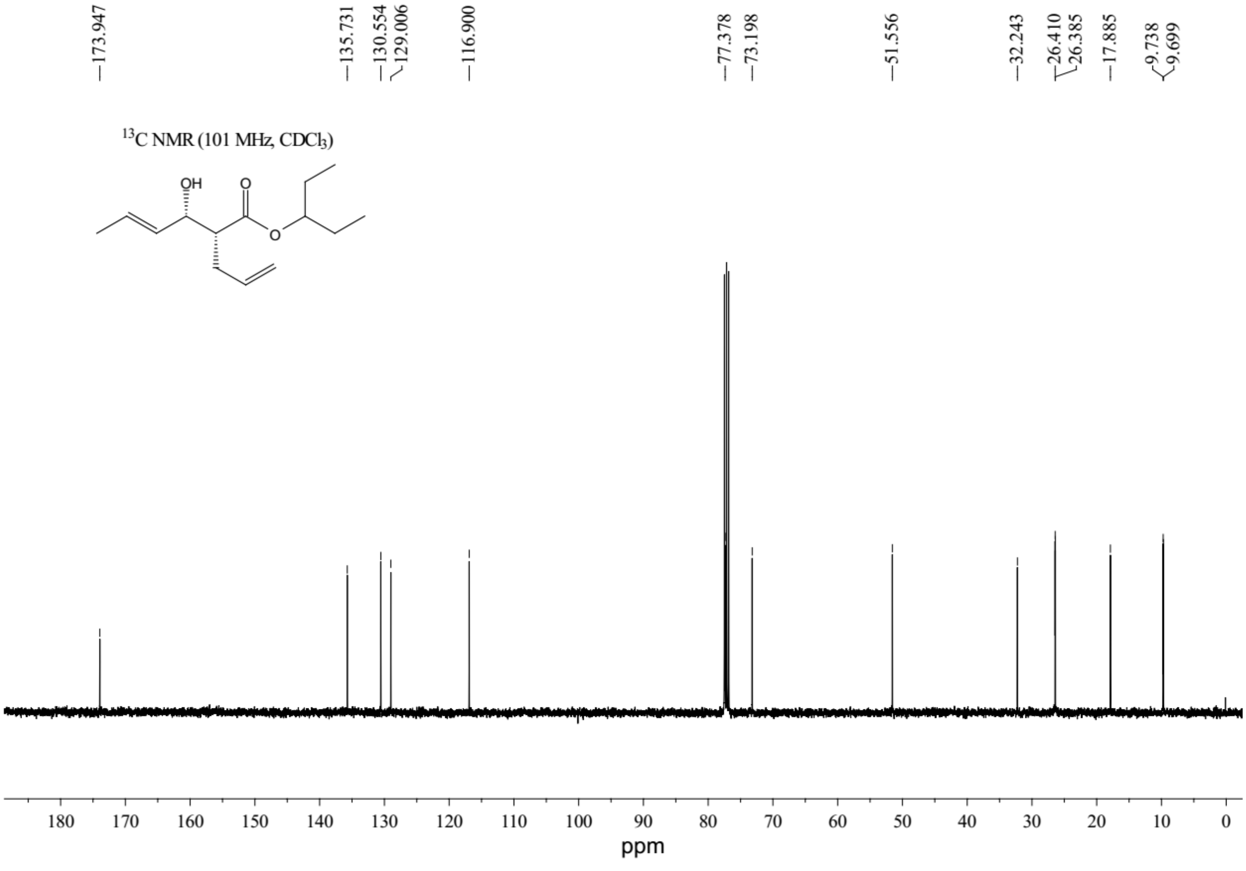

Supplementary Figure 193. ^{1}H NMR spectrum for *syn*-Pentan-3-yl (1*R*,2*S*)-2-hydroxycyclopent-3-ene-1-carboxylate (18)

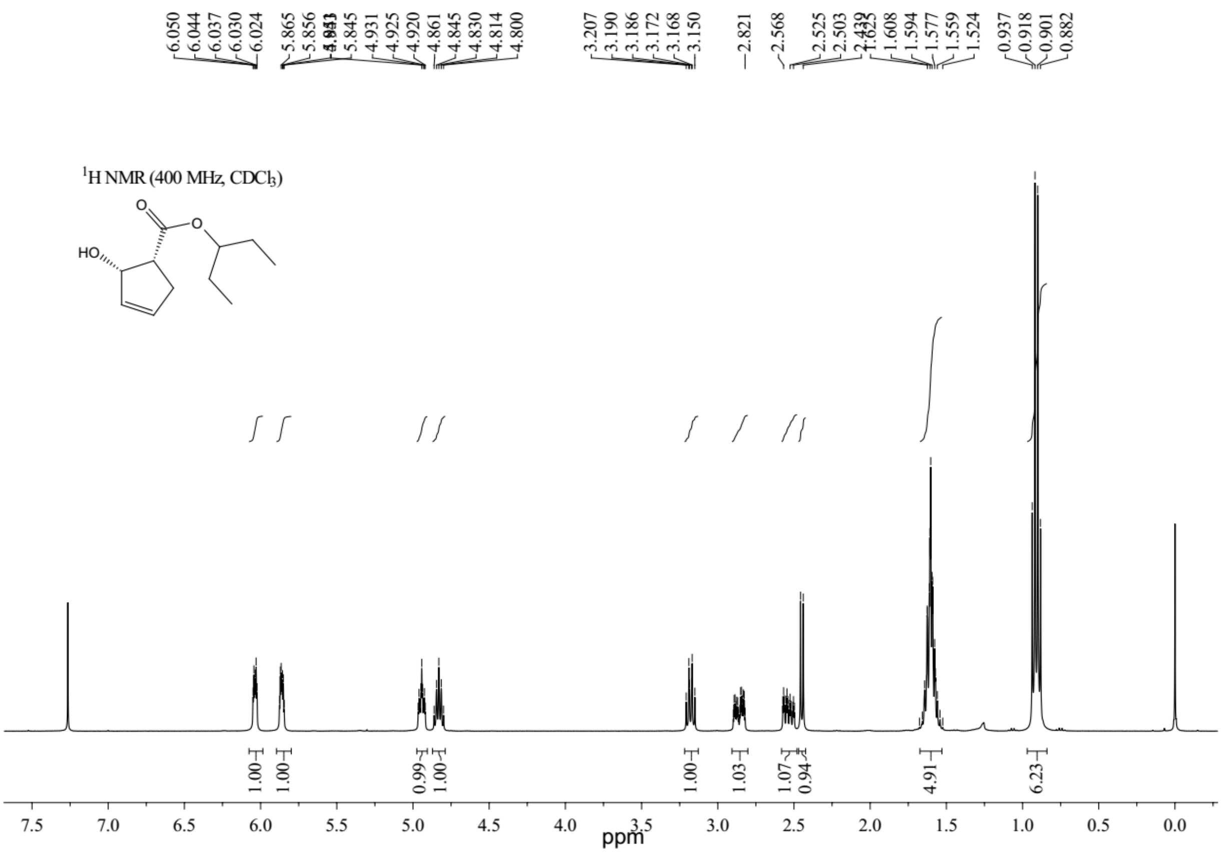

Supplementary Figure 194. ^{13}C NMR spectrum for 18

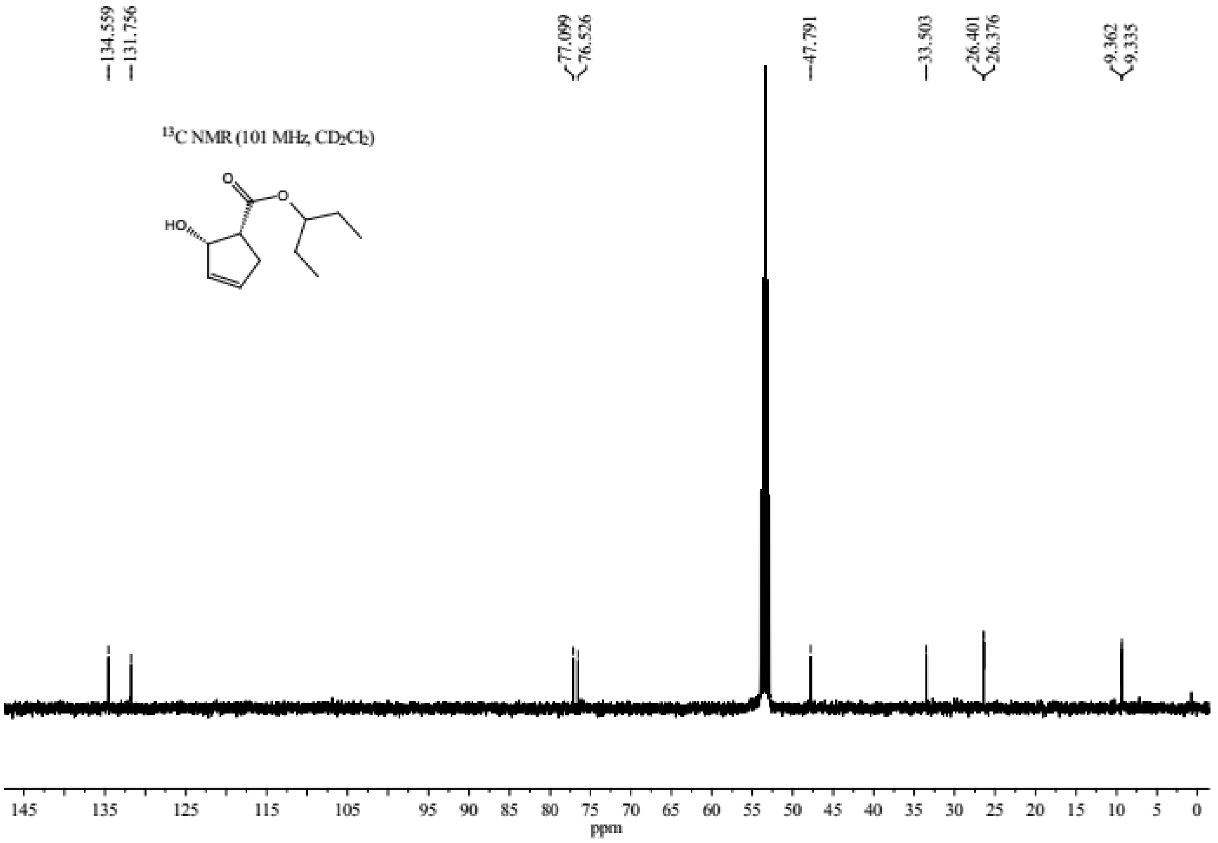

Supplementary Figure 195. ^{1}H NMR spectrum for *syn*-(1*S*,5*S*)-5-(hydroxymethyl)cyclopent-2-en-1-ol (19)

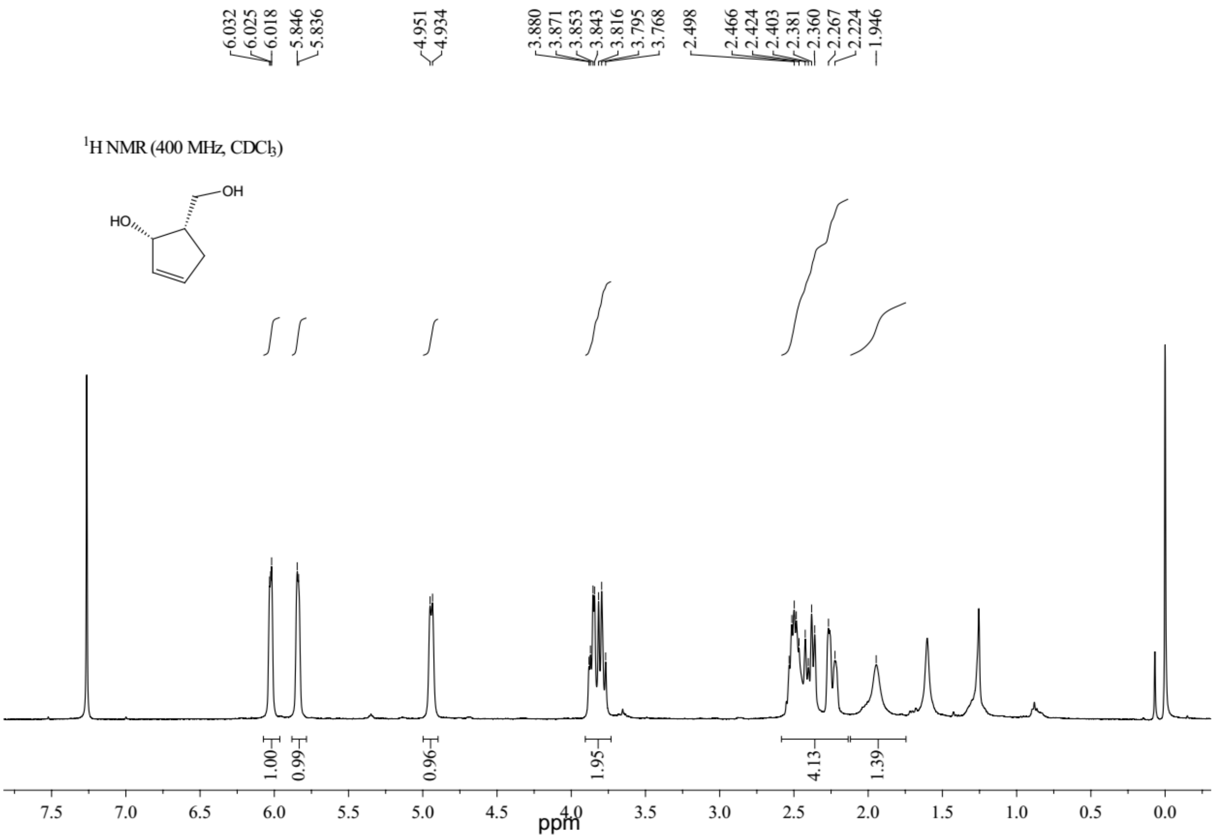

Supplementary Figure 196. ^{13}C NMR spectrum for 19

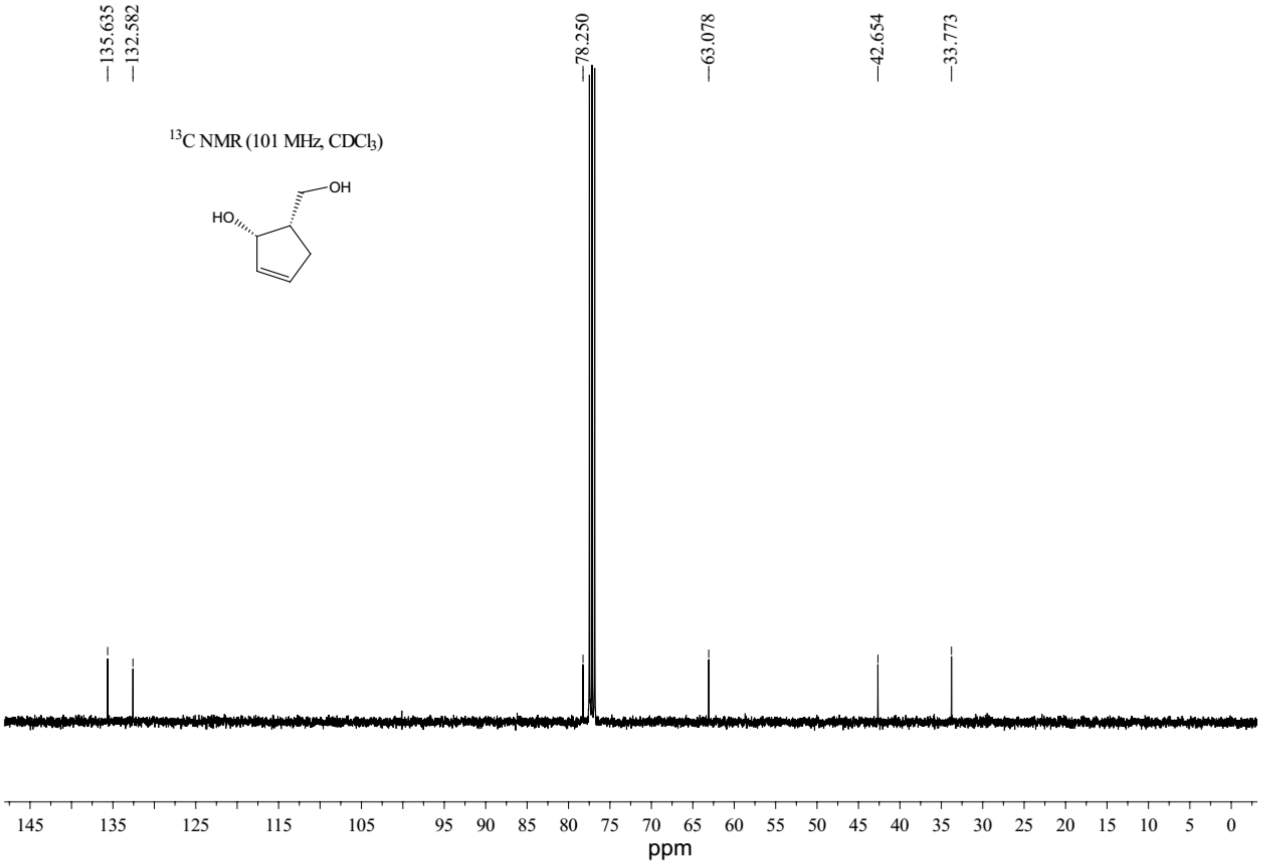

Supplementary Figure 197. ¹H NMR spectrum for *anti*-Pentan-3-yl (2*S*,3*S*,*E*)-2-allyl-3-hydroxyhex-4-enoate (20)

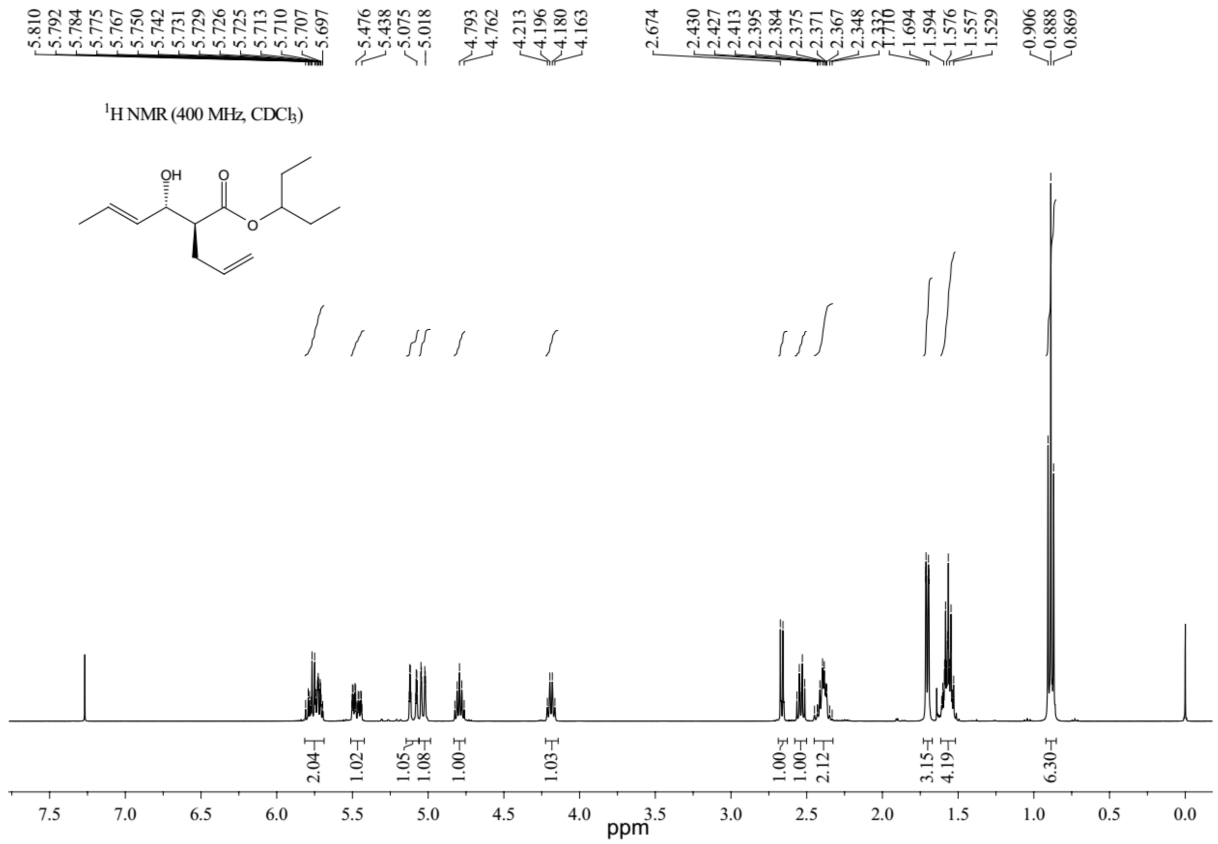

Supplementary Figure 198. ¹³C NMR spectrum for 20

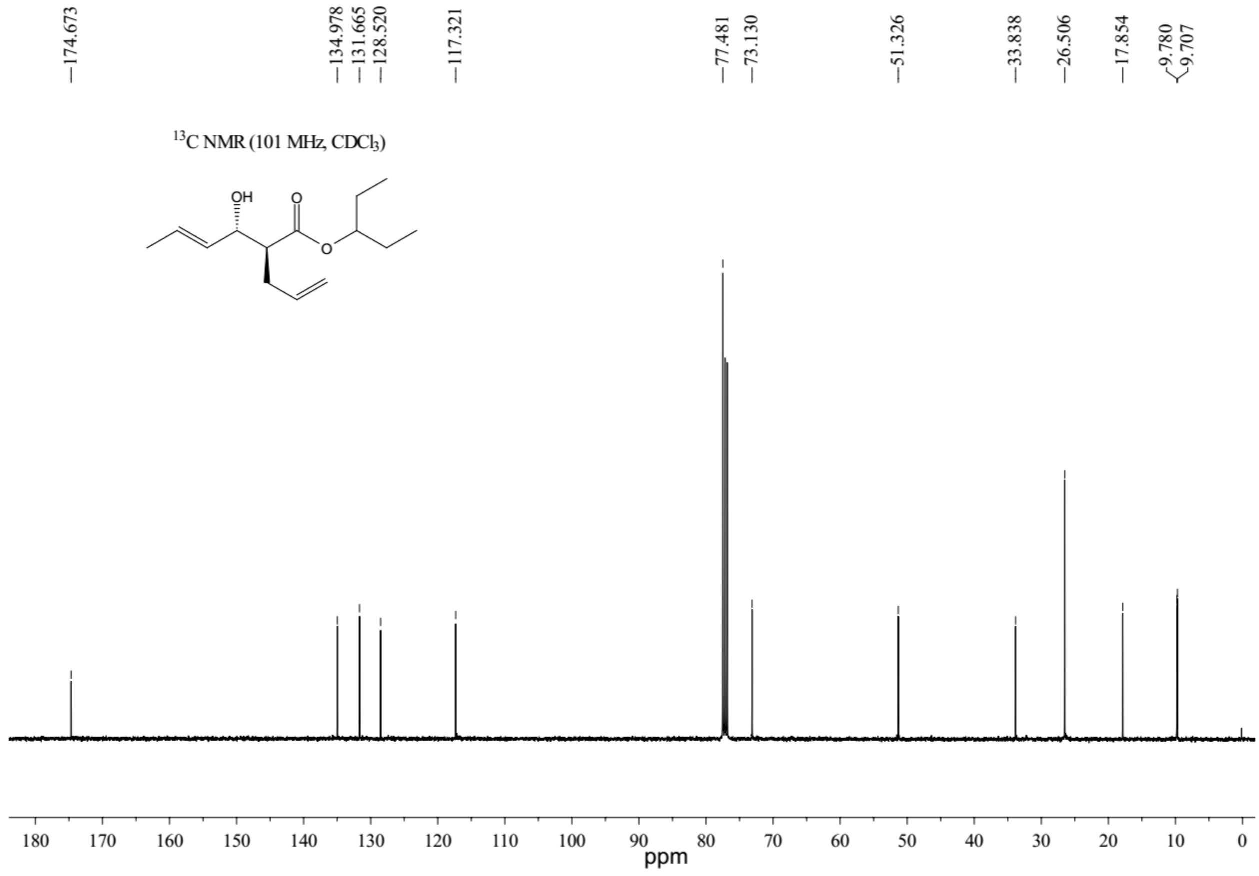

Supplementary Figure 199. ^{1}H NMR spectrum for *anti*-Pentan-3-yl (1*S*,2*S*)-2-hydroxycyclopent-3-ene-1-carboxylate (21)

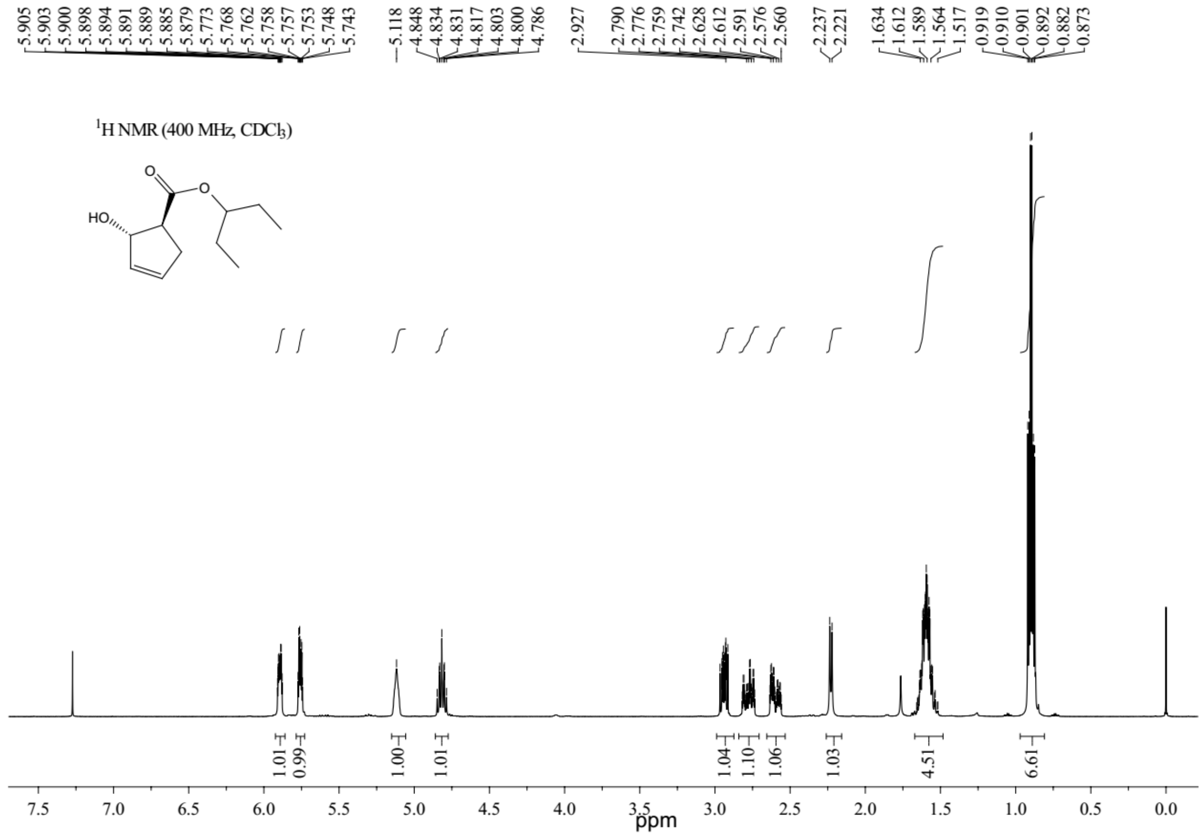

Supplementary Figure 200. ^{13}C NMR spectrum for 21

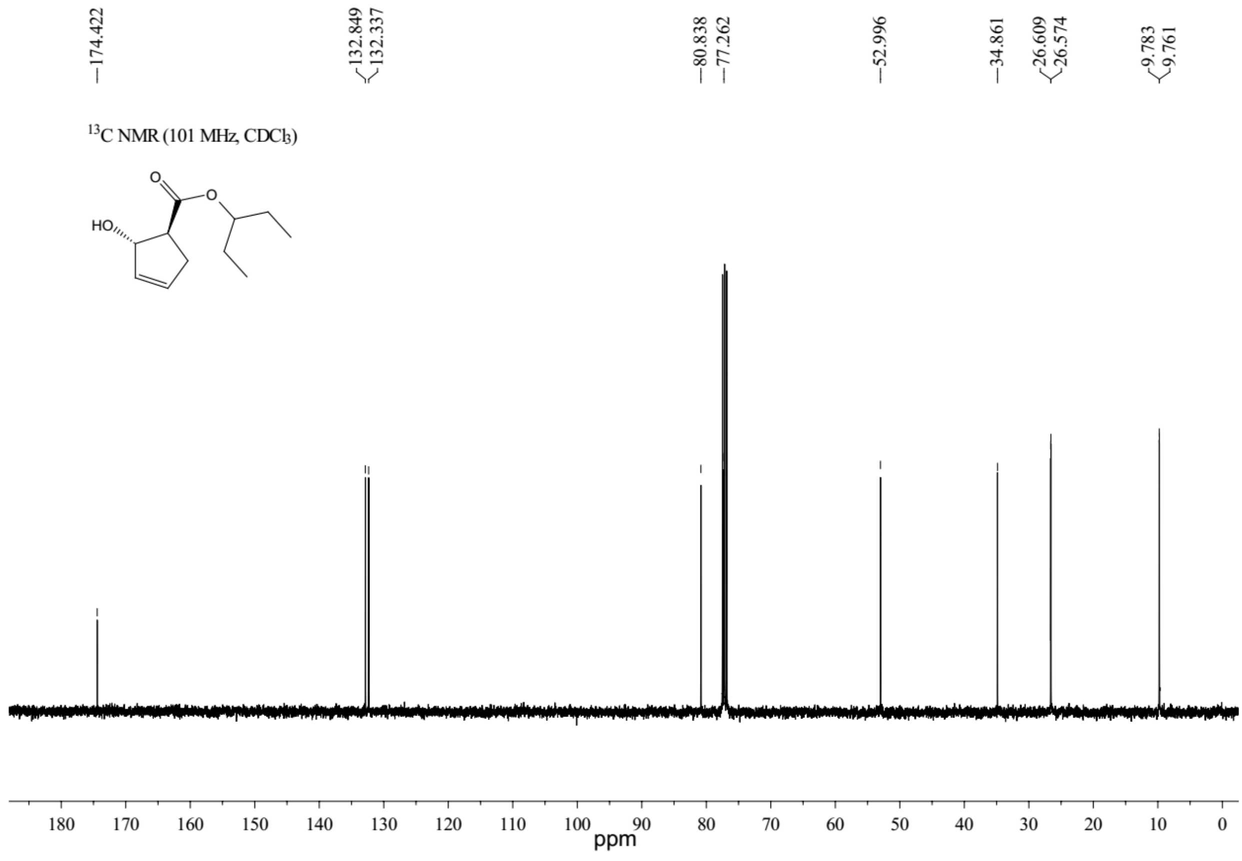

Supplementary Figure 201. ^{1}H NMR spectrum for *anti*-(1*S*,5*R*)-5-(hydroxymethyl)cyclopent-2-en-1-ol (22)

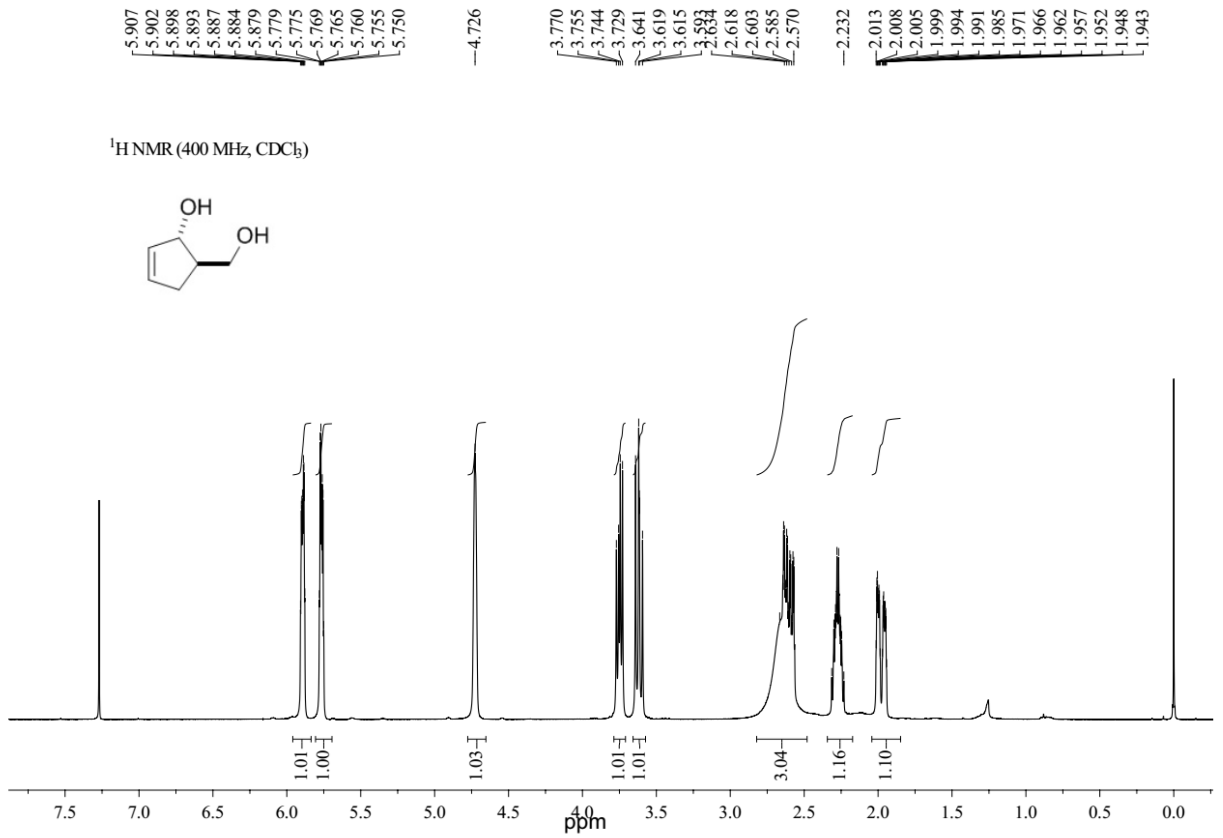

Supplementary Figure 202. ^{13}C NMR spectrum for 22

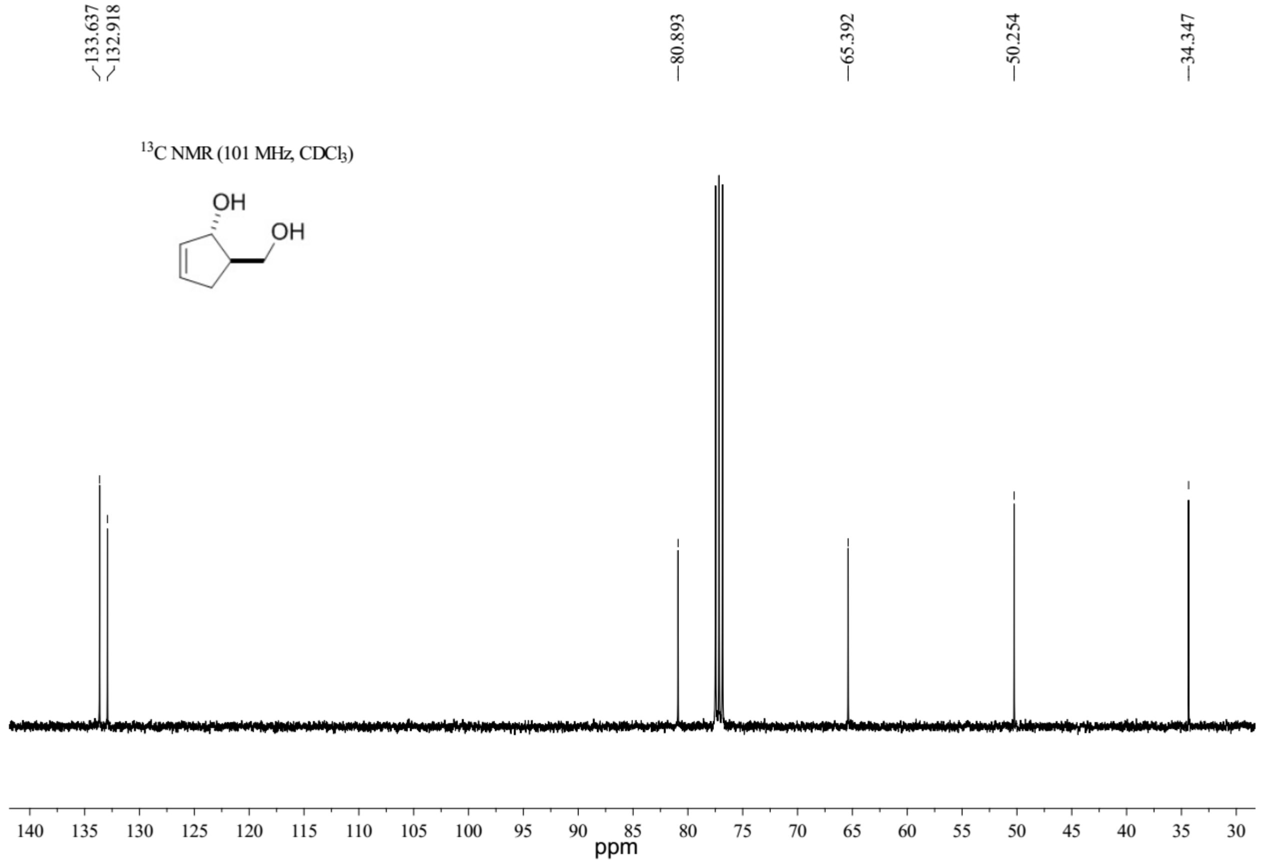

Supplementary Figure 203. ^{1}H NMR spectrum for pentan-3-yl (S)-3-phenyl-3-(4,4,5,5-tetraphenyl-1,3,2-dioxaborolan-2-yl)propanoate (1d)

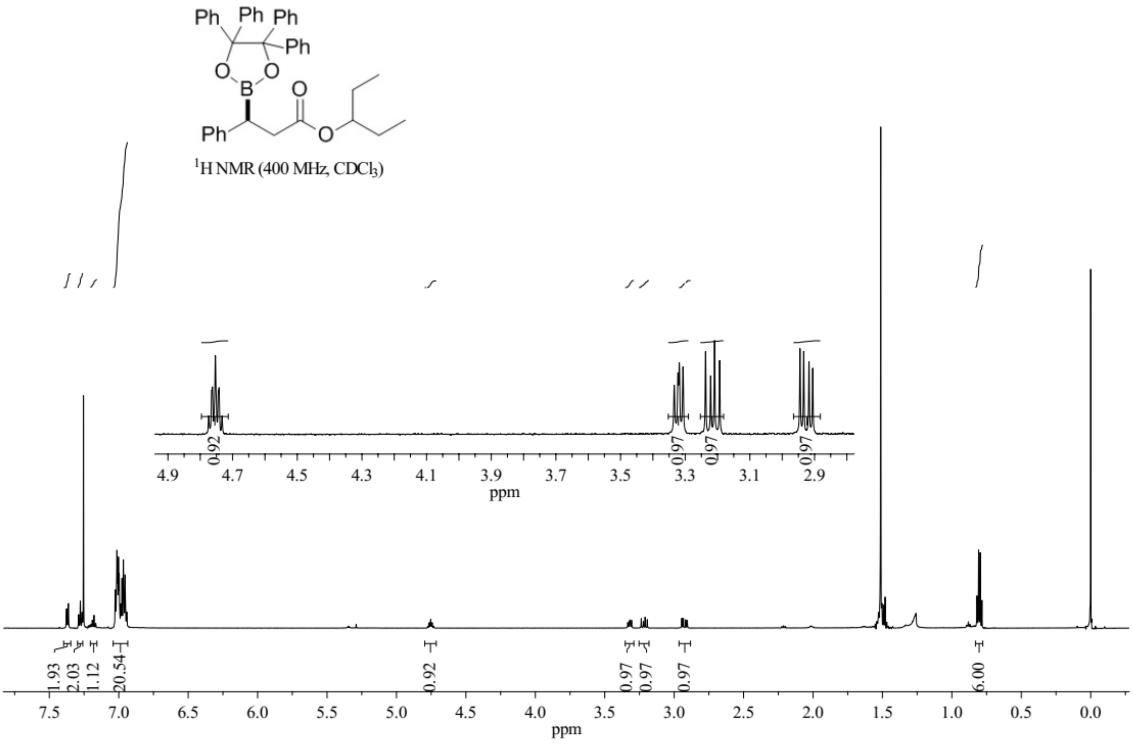

Supplementary Figure 204. ^{1}H NMR spectrum for *anti*-pentan-3-yl 2-(phenyl(4,4,5,5-tetraphenyl-1,3,2-dioxaborolan-2-yl)methyl)pent-4-enoate (3d)

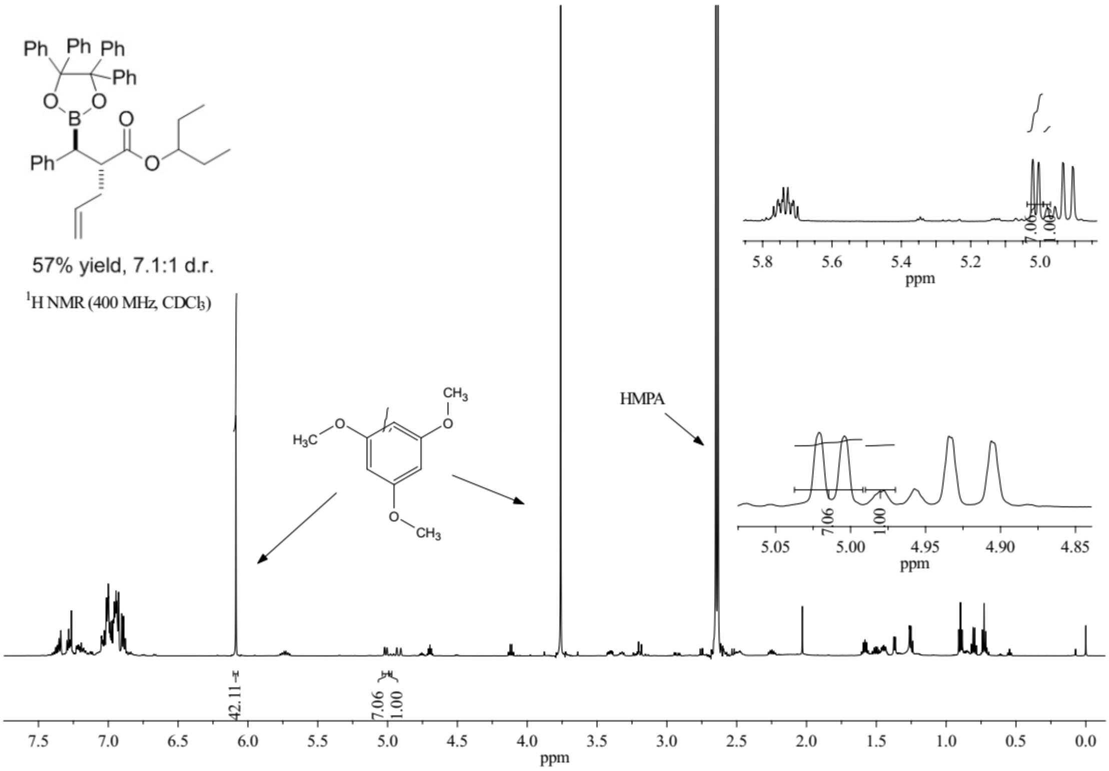

Supplementary Figure 205. **^{1}H NMR spectrum for syn-pentan-3-yl 2-(-phenyl(4,4,5,5-tetraphenyl-1,3,2-dioxaborolan-2-yl)methyl)pent-4-enoate (4d)**

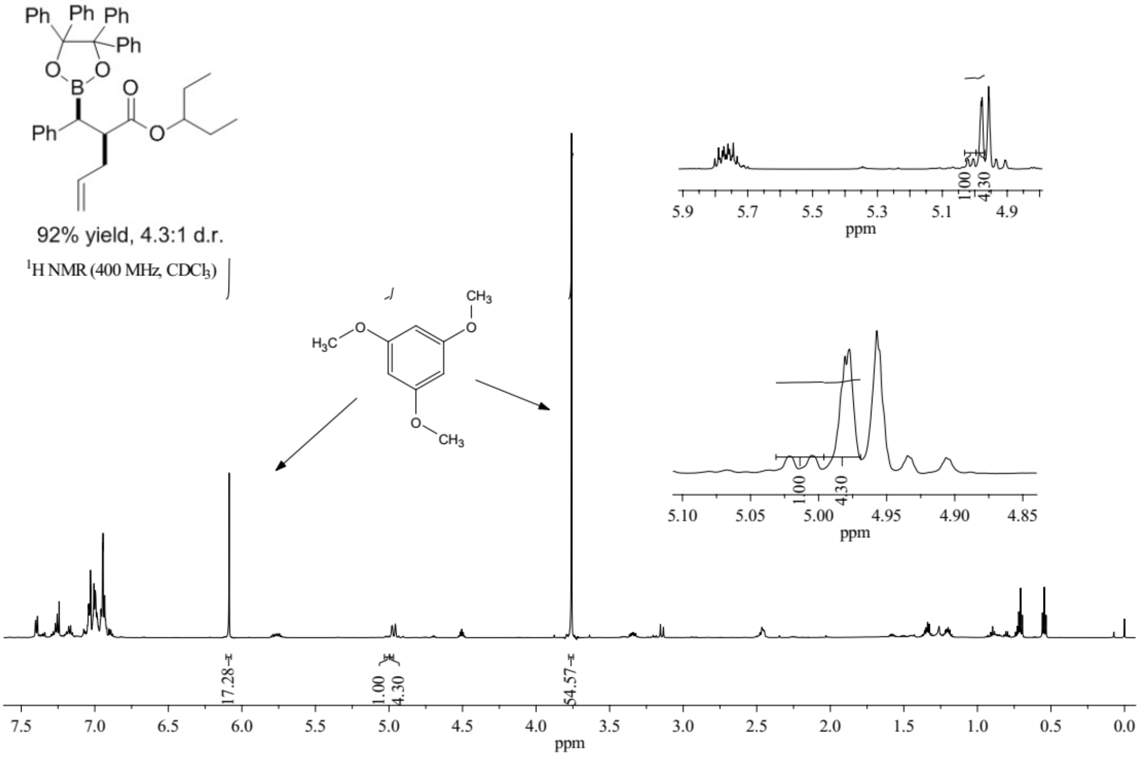

Supplementary Figure 206. **^{1}H NMR spectrum for silyl enol ether of 1b at room temperature**

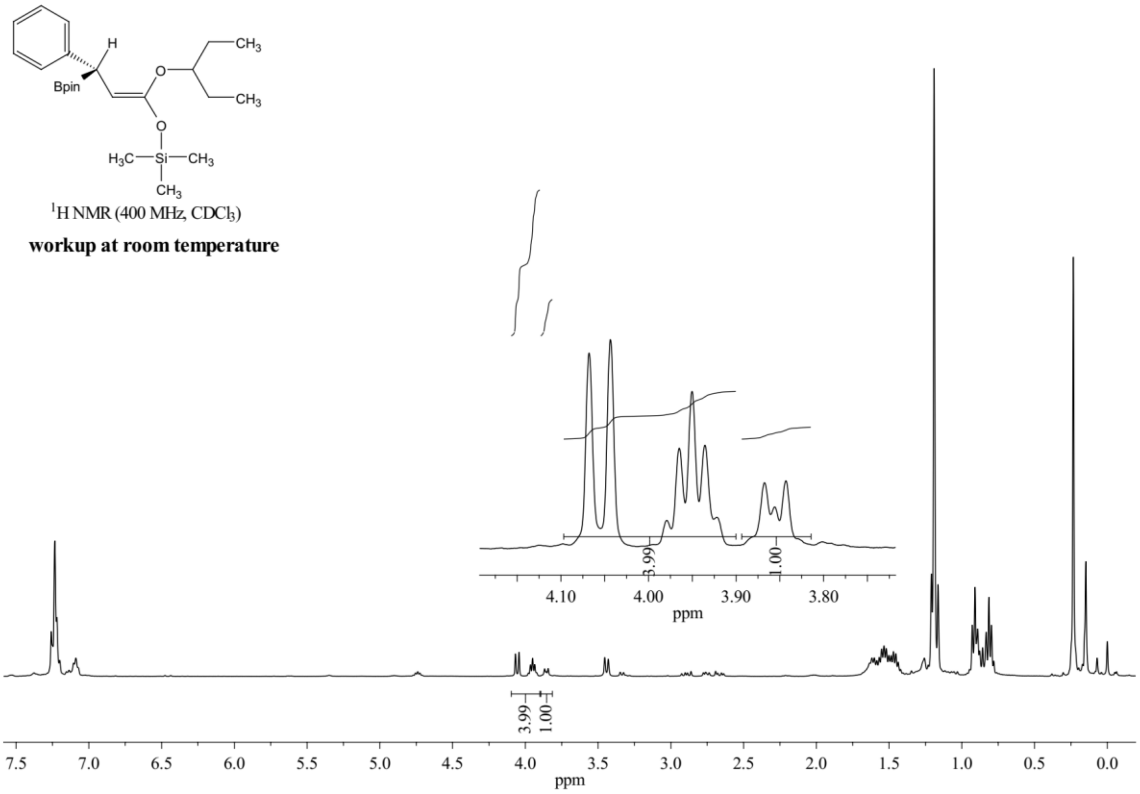

Supplementary Figure 207. ^{1}H NMR spectrum for silyl enol ether of 1b at 0 to 10 ℃

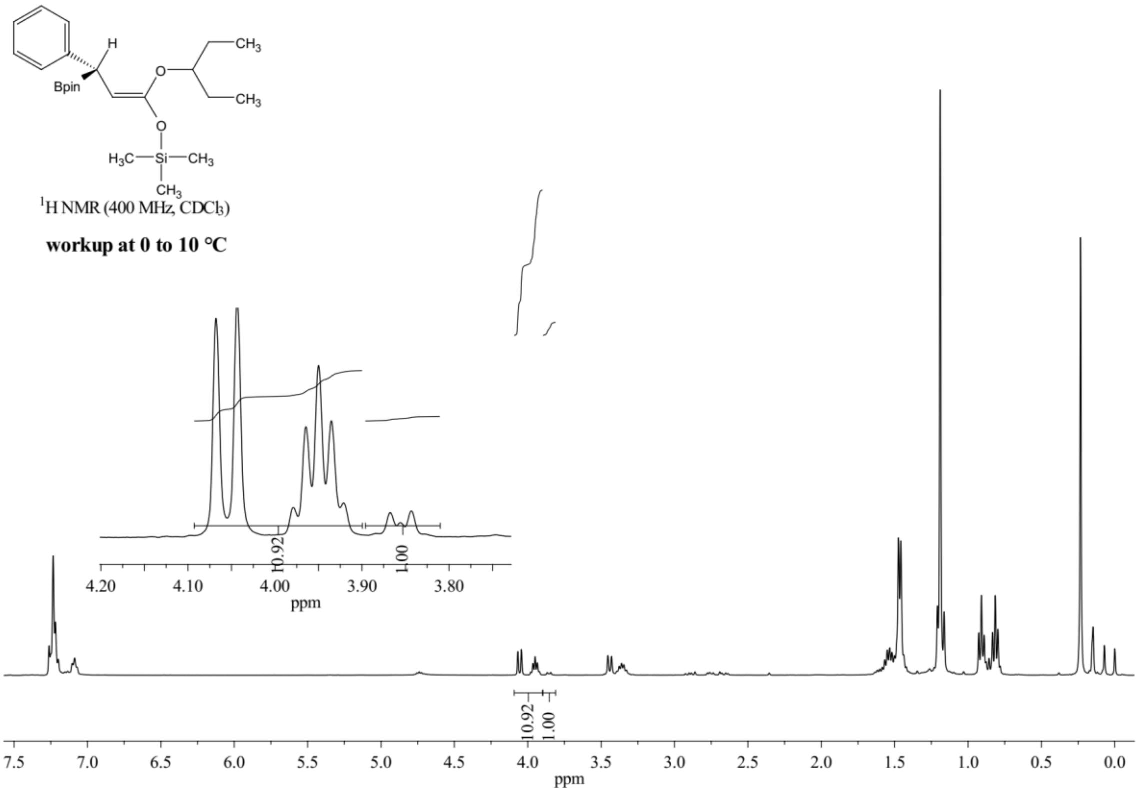

Supplementary Figure 208. ^{1}H NMR spectrum for silyl enol ether of 1b at -20 ℃

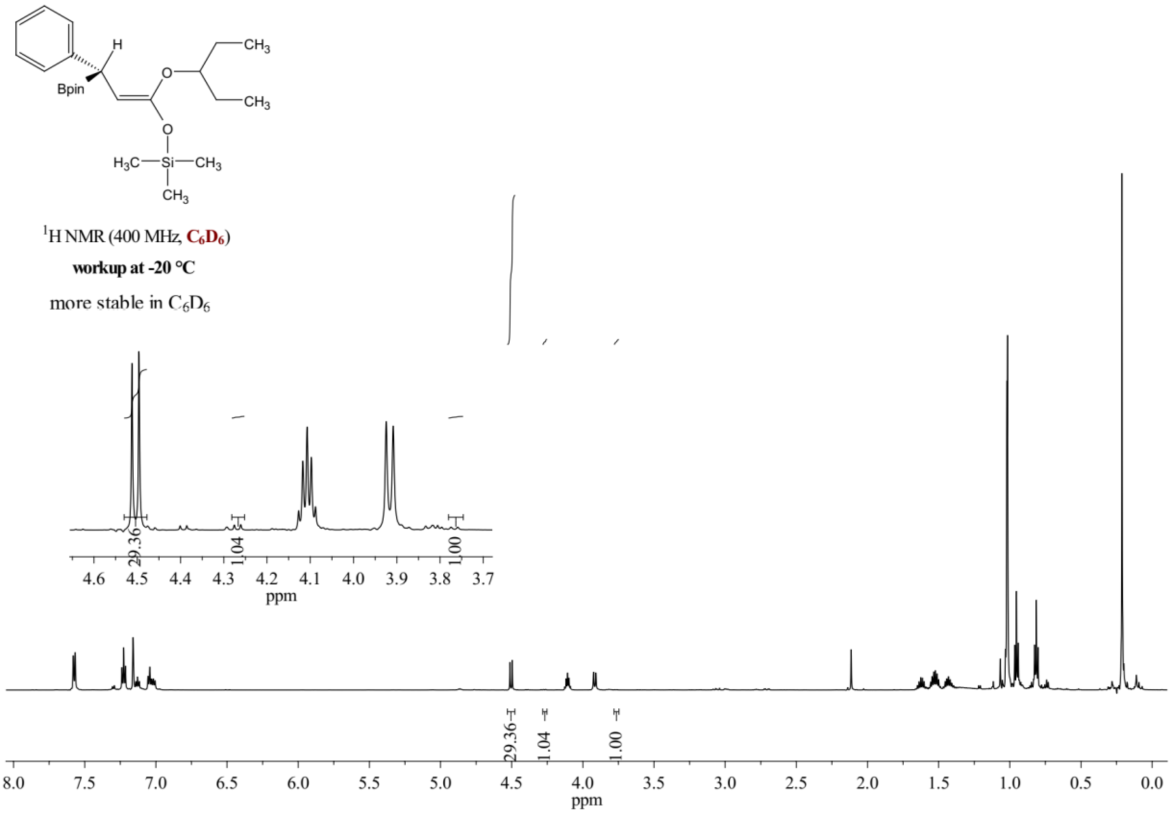

Supplementary Figure 209. ¹H NMR spectrum for silyl enol ether of 5i at room temperature

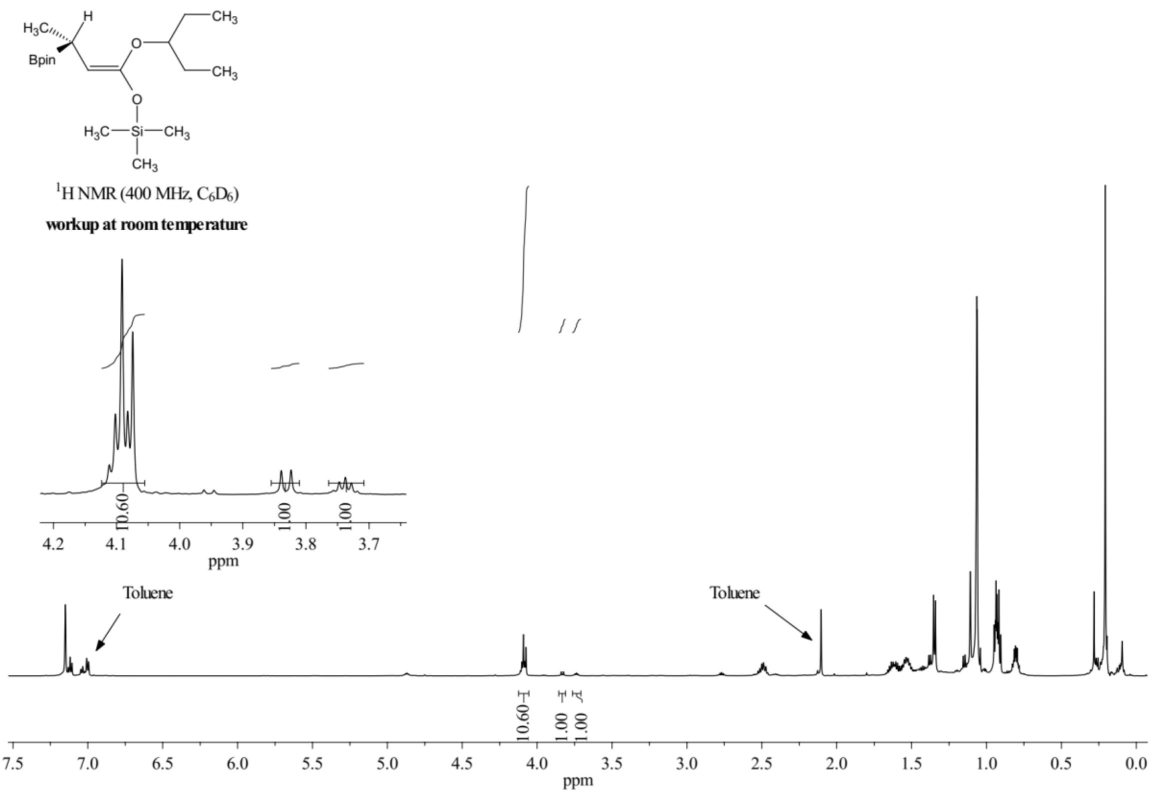

Supplementary Figure 210. ¹H NMR spectrum for silyl enol ether of 1b at -20 to 0 ℃

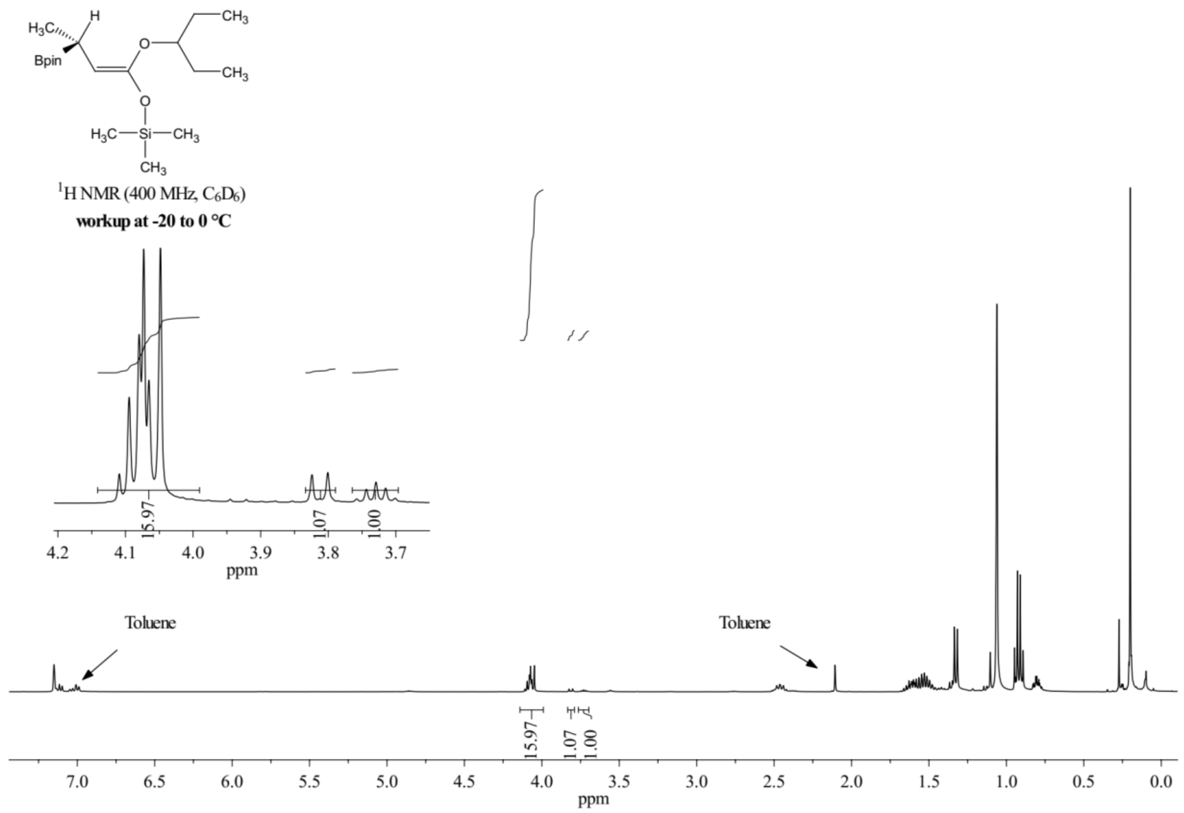

Supplementary References

1. Ma, Y. & Collum, D. B. Lithium Diisopropylamide-Mediated Reactions of Imines, Unsaturated Esters, Epoxides, and Aryl Carbamates: Influence of Hexamethylphosphoramide and Ethereal Cosolvents on Reaction Mechanisms. *J. Am. Chem. Soc.* **129***,* 14818-14825 (2007).

2. Kim, Y. & Chang, S. Borane-Catalyzed Reductive α-Silylation of Conjugated Esters and Amides Leaving Carbonyl Groups Intact. *Angew. Chem., Int. Ed.* **55,** 218-222 (2016).

3. Gaussian 09, Revision D.01, Frisch, M. J. et al. Gaussian, Inc., Wallingford CT, 2013.

4. Becke, A. D. Density‐functional thermochemistry. III. The role of exact exchange. *J. Chem. Phys*. **98**, 5648-5652 (1993).

5. Lee, C., Yang, W. & Parr, R. G. Development of the Colle-Salvetti correlation-energy formula into a functional of the electron density. *Phys. Rev. B* **37**, 785−789 (1988).

6. Marenich, A. V., Cramer, C. J. & Truhlar, D. G. Universal Solvation Model Based on Solute Electron Density and on a Continuum Model of the Solvent Defined by the Bulk Dielectric Constant and Atomic Surface Tensions. *J. Phys. Chem. B* **113**, 6378−6396 (2009).

7. Mun, S., Lee, J.-E. & Yun, J. Copper-Catalyzed β-Boration of α,β-Unsaturated Carbonyl Compounds: Rate Acceleration by Alcohol Additives. *Org. Lett.* **8**, 4887−4889 (2006).

8. Chea, H., Sim, H.-S. & Yun, J. Copper-Catalyzed Conjugate Addition of Diboron Reagents to α,β-Unsaturated Amides: Highly Reactive Copper-1,2- Bis(diphenylphosphino)benzene Catalyst System. *Adv. Synth. Catal.* **351**, 855−858 (2009)**.**

9. Kitanosono, T., Xu, P. & Kobayashi, S. Heterogeneous and homogeneous chiral Cu(II) catalysis in water: enantioselective boron conjugate additions to dienones and dienoesters. *Chem. Comm.* **49,** 8184−8186 (2013).

10. Zhan, M. et al. Silver-Assisted, Iridium-Catalyzed Allylation of Bis[(pinacolato)boryl]methane Allows the Synthesis of Enantioenriched Homoallylic Organoboronic Esters. *ACS Catal.* **6,** 3381−3386 (2016).

11. Wang, Z., Bachman, S., Dudnik, A. S. & Fu, G. C. Nickel-Catalyzed Enantioconvergent Borylation of Racemic Secondary Benzylic Electrophiles. *Angew. Chem., Int. Ed.* **57,** 14529−14532 (2018).

12. Bonet, A., Odachowski, M., Leonori, D., Essafi, S. & Aggarwal, V. K. Enantiospecific sp^2–sp^3 coupling of secondary and tertiary boronic esters. *Nat. Chem.* **6,** 584−589 (2014).
