# Supplementary material for: A unified approach for divergent synthesis of contiguous stereodiads employing a small boronyl group
Source: Nat Commun. 2020 Feb 7;11:792. doi: 10.1038/s41467-020-14592-7 (PMC7005891; doi:10.1038/s41467-020-14592-7)
Supplement: Supplementary file 2 — Description of Additional Supplementary Files [file 41467_2020_14592_MOESM2_ESM.pdf]

Description of Additional Supplementary Files

File Name: Supplementary Data 1

Description: Calculated Energies and Cartesian Coordinates
