# Supplementary material for: A unified approach for divergent synthesis of contiguous stereodiads employing a small boronyl group
Source: Nat Commun. 2020 Feb 7;11:792. doi: 10.1038/s41467-020-14592-7 (PMC7005891; doi:10.1038/s41467-020-14592-7)
Supplement: Supplementary file 3 — Supplementary Data 1 [file 41467_2020_14592_MOESM3_ESM.pdf]

Absolute Calculation Energies, Enthalpies, and Free Energies

Geometry	$E_{(elec\text{-}B3LYP)}$[1]	$G_{(corr\text{-}B3LYP)}$[2]	$H_{(corr\text{-}B3LYP)}$[3]	$E_{(solv,\ M06\text{-}2X)}$[4]	IF[5]
**1**	-1106.674134	0.428444	0.515577	-1106.545525	-
**2**	-2688.707406	0.042158	0.077215	-2691.452608	
**THF**	-232.445608	0.088794	0.123318	-232.413273	-
**C-3**	-764.296783	0.372805	0.457117	-764.170483	-
**C-4**	-1638.522861	0.716855	0.849141	-1638.30072	-
**5-ts**	-1638.514491	0.715009	0.844578	-1638.290446	-1323.48
**C-6**	-1638.543282	0.718511	0.851285	-1638.324075	-
**C-6-Me**	-1446.808482	0.670472	0.794945	-	-
**C-6-Cy**	-1642.167218	0.788831	0.922885	-	-
**C-6-T**	-1959.301197	0.686938	0.817394	-	-
**7-ts**	-1638.511435	0.715210	0.844811	-1638.288559	-1376.34
**C-8**	-1638.538081	0.723252	0.850821	-1638.332732	-
**9-ts**	-4327.248698	0.776740	0.929243	-4329.775208	-258.14
**C-10**	-1755.724595	0.790814	0.929935	-1755.514066	-
**11-ts**	-4327.24961	0.779432	0.929093	-4329.774803	-246.30
**C-12**	-1755.725242	0.789214	0.930815	-1755.516267	-
**5-ts-Me**	-1446.780322	0.668374	0.788569	-1446.573575	-1330.33
**7-ts-Me**	-1446.776829	0.667698	0.788593	-1446.569691	-1376.20
**9-ts-Me**	-4135.514078	0.732106	0.873203	-4138.058145	-258.18
**11-ts-Me**	-4135.516333	0.732200	0.873072	-4138.057688	-246.73
**Br^-**	-2571.464366	-0.016176	0.002360	-2574.333611	-

[1]The electronic energy calculated by B3LYP in gas phase. [2]The thermal correction to Gibbs free energy calculated by B3LYP in gas phase. [3]The thermal correction to enthalpy calculated by B3LYP in gas phase. [4]The electronic energy calculated by M06-2X in THF solvent. [5]The B3LYP calculated imaginary frequencies for the transition states.

B3LYP Geometries for All the Optimized Compounds and Transition States

1

C 0.83708700 -1.15307800 0.26185000

H 0.50101700 -1.48240700 1.25293200

C -0.01553300 -1.88156700 -0.81143400

H	0.28753100	-2.92757100	-0.91014500
C	-1.50739900	-1.89744400	-0.52787400
O	-2.21342500	-2.87475700	-0.66756200
O	-1.94676400	-0.68656500	-0.11265200
C	-3.35833800	-0.48661600	0.22737200
C	-4.27379100	-0.66437200	-0.98988800
C	-3.74393300	-1.31677000	1.45943400
H	-4.29316000	-1.72014200	-1.27392500
H	-5.28938000	-0.39867900	-0.66918700
H	-2.92337600	-1.23037300	2.18320900
H	-3.81526400	-2.37171100	1.18020800
C	1.00065800	2.58695600	0.87081100
C	0.52200000	2.54214600	-0.63427000
B	0.71378000	0.42397600	0.20551100
O	0.69352700	1.13502500	-0.97051200
O	0.74669800	1.22410600	1.31893100
C	2.31160300	-1.53102900	0.13407300
C	2.94857000	-2.27286300	1.13890800
C	3.06511500	-1.15505300	-0.99056500
C	4.29268800	-2.63327300	1.02684900
H	2.38305400	-2.57202700	2.01851000
C	4.40767000	-1.51774300	-1.10716600
H	2.59669300	-0.56574200	-1.77438100
C	5.02793600	-2.25864700	-0.09878900
H	4.76369600	-3.20881000	1.81978600
H	4.97119700	-1.21719500	-1.98701600
H	6.07382200	-2.53976600	-0.18913400
C	-0.96744700	2.86330500	-0.80955200
H	-1.26620100	2.62082100	-1.83408900
H	-1.58256500	2.26634500	-0.13045800
H	-1.17382900	3.92395900	-0.63082700
C	1.35501900	3.37435200	-1.60821200
H	0.96959700	3.24276900	-2.62458800
H	1.29654700	4.44056500	-1.36041000
H	2.40501300	3.07285700	-1.60409700
C	0.22576900	3.54477200	1.77400800
H	0.60680800	3.47123100	2.79772000
H	0.34833400	4.58191700	1.44102000
H	-0.84067000	3.30865900	1.79417900
C	2.50970800	2.82108000	1.02296200
H	2.78924500	3.84630200	0.75806600
H	2.79264600	2.64510800	2.06543400
H	3.08413200	2.12939400	0.39875300
H	0.13382500	-1.40535800	-1.78919300
H	-3.35975900	0.57300200	0.51100800
C	-3.88309600	0.20409600	-2.19070700
H	-3.88570300	1.27029200	-1.93156100
H	-2.88186000	-0.04488000	-2.55690700
H	-4.58750800	0.05965300	-3.01718300
C	-5.04765900	-0.84821600	2.11868200
H	-5.91501000	-0.99330000	1.46529800
H	-5.23403700	-1.41277400	3.03862600
H	-5.00378800	0.21529400	2.38620000

2

C	2.76143400	-0.34480200	-0.28574400
C	1.71353600	-0.01614300	0.47301500
H	2.85331100	-0.00347100	-1.31461700
H	3.56702500	-0.96571200	0.09545300
H	1.64035000	-0.38018500	1.49625100
C	0.60633400	0.86159600	0.00809800
H	0.41175100	1.69539700	0.68373100
H	0.75612300	1.22863900	-1.00699300
Br	-1.13475400	-0.13081600	-0.03217300

THF

C	1.16477900	-0.43149800	0.13268200
O	-0.00044500	-1.25109300	-0.00109900
C	-1.16550100	-0.43050800	-0.13131700
C	-0.73310600	0.99734400	0.22678100
C	0.73432700	0.99628000	-0.22739600
H	1.53294200	-0.48334500	1.16931800
H	1.94926700	-0.82497100	-0.52447100
H	-1.53686000	-0.48294200	-1.16671500
H	-1.94830200	-0.82282000	0.52858900
H	-1.34303200	1.76207100	-0.26427700
H	-0.79578100	1.15637400	1.31011600
H	0.79730100	1.15363700	-1.31095300
H	1.34503100	1.76103400	0.26268200

C-3

Li	0.01356700	0.25066400	-0.06683600
C	-1.74128200	2.69024500	0.12688200
O	-1.31273700	1.57214300	-0.66780800
C	-2.48111600	0.97083700	-1.29378900
C	-3.71262400	1.52020100	-0.53945100
C	-3.10645200	2.26829100	0.66568000
H	-0.97954200	2.85803700	0.89283200
H	-1.81649600	3.58779800	-0.50499000
H	-2.48937000	1.24539400	-2.35484400
H	-2.34926500	-0.11008700	-1.19740000

H	-4.27828400	2.21038100	-1.17465000
H	-4.39166900	0.72047000	-0.23096700
H	-3.71482500	3.11672600	0.99362500
H	-2.97499600	1.58950000	1.51543800
N	-0.16255900	-1.60658400	0.00442000
C	-0.80875200	-2.32259100	1.08773300
C	-2.23305600	-2.83041300	0.74472500
C	-0.88723500	-1.42888400	2.33603900
H	-0.23315400	-3.22286800	1.39356500
H	-2.22852100	-3.43143500	-0.17167900
H	-2.65522700	-3.45295500	1.54729700
H	-2.90920000	-1.97911500	0.58132700
H	0.11533400	-1.14336400	2.67764600
H	-1.44387800	-0.50529000	2.10873500
H	-1.40508200	-1.92330400	3.16791400
C	0.31707800	-2.47033800	-1.05750700
C	1.60032300	-3.26095000	-0.69518600
C	0.58583900	-1.64272400	-2.32515700
H	-0.43681500	-3.23421100	-1.34718300
H	1.46615000	-3.83256800	0.23013500
H	1.88427700	-3.97193400	-1.48488300
H	2.43999100	-2.56881200	-0.53945500
H	-0.33241900	-1.15599400	-2.67705700
H	1.32961300	-0.85633700	-2.11683100
H	0.98427100	-2.25564800	-3.14378500
C	2.24524400	2.26914200	-0.36831200
O	1.58012200	1.30620800	0.48378900
C	2.56221400	0.50383600	1.19518000
C	3.85819200	0.67794000	0.40839500
C	3.74496600	2.13307300	-0.07561500
H	2.00995100	2.02510300	-1.41160900
H	1.85005600	3.26569800	-0.14613500
H	2.65403900	0.89015000	2.21910600
H	2.17315300	-0.51772100	1.21482400
H	4.74703900	0.49588200	1.01972900
H	3.87977900	-0.01291500	-0.44252000
H	4.04441000	2.82357800	0.72149500
H	4.35877900	2.34712400	-0.95566600

C-4

C	-1.55612700	0.86858700	0.45151800
H	-1.33602900	0.70537000	1.51383500
C	-0.33537300	0.38542900	-0.37526000
H	0.52414200	1.05987200	-0.26081700
C	0.16778900	-0.99546200	-0.02361500
O	1.35813100	-1.30602700	-0.02148900
O	-0.80731300	-1.85473200	0.27807100
C	-0.52322400	-3.23796300	0.69649100
C	0.15802400	-4.05351600	-0.40754700
C	0.18698200	-3.24982000	2.05616300
H	1.19042000	-3.71338100	-0.52689500
H	0.20242700	-5.08980300	-0.04862200
H	-0.30580400	-2.50450800	2.69277700
H	1.22583800	-2.92932800	1.93253700
C	-5.09882000	-0.47916500	0.36678200
C	-4.58819200	-0.88006600	-1.07435200
B	-2.91210900	0.12596700	0.11781700
O	-3.31670200	-0.17464200	-1.16139500
O	-3.85814700	-0.17429800	1.06486000
C	-1.76391900	2.37116000	0.25841800
C	-1.50546200	3.26648400	1.30562800
C	-2.20454000	2.89314300	-0.96902000
C	-1.67825500	4.64128900	1.13558300
H	-1.15730500	2.88243000	2.26134500
C	-2.37498000	4.26758400	-1.14193300
H	-2.42135400	2.21699600	-1.79179100
C	-2.11360100	5.14765200	-0.08991600
H	-1.46673000	5.31595100	1.96109600
H	-2.71348000	4.65067700	-2.10142700
H	-2.24495400	6.21793300	-0.22513600
C	-4.27621500	-2.37512000	-1.21814700
H	-3.74897700	-2.53883200	-2.16312900
H	-3.63003900	-2.72367400	-0.40712900
H	-5.18968600	-2.97926800	-1.22316900
C	-5.47344400	-0.41793000	-2.23109800
H	-5.01442900	-0.70743000	-3.18208000
H	-6.46357700	-0.88520200	-2.17752700
H	-5.60000500	0.66694500	-2.23499700
C	-5.82442100	-1.58469000	1.13222200
H	-6.10053100	-1.21980400	2.12671600
H	-6.74314700	-1.88222100	0.61369400
H	-5.19505600	-2.46830000	1.26138800
C	-5.93439700	0.80846000	0.38360200
H	-6.91704400	0.66024600	-0.07670100
H	-6.08394300	1.11901200	1.42226900
H	-5.42270600	1.62340100	-0.13796100
Li	2.92709900	-0.12085600	0.04918500
C	4.58993200	-2.50689300	-0.85434300
O	4.44452600	-1.36841900	0.02471200

C	5.74051200	-0.77828900	0.29186100
C	6.60256100	-1.19154500	-0.89682700
C	6.08981900	-2.61456300	-1.17783200
H	4.19601500	-3.39688100	-0.35179000
H	3.98727500	-2.31959600	-1.75087100
H	5.57666900	0.29680800	0.39466000
H	6.13126800	-1.18654100	1.23463800
H	6.40756900	-0.53118500	-1.74981500
H	7.67322700	-1.15735800	-0.67410500
H	6.26828300	-2.94432400	-2.20569900
H	6.57590300	-3.33155100	-0.50611400
H	-0.59028400	0.36915000	-1.44396900
N	3.06070400	1.74810000	0.12805300
C	2.78075900	2.45569500	1.36221000
C	3.93688000	3.31466300	1.93629200
C	2.34596400	1.45306100	2.44532400
H	1.92953700	3.16545900	1.23187900
H	4.22741500	4.10790200	1.23966500
H	3.64827200	3.79838500	2.88121400
H	4.82120500	2.68956600	2.12317300
H	1.47057200	0.87388300	2.12293700
H	3.16067400	0.74508700	2.66185700
H	2.08758800	1.95324700	3.38718200
C	3.32178900	2.61990800	-1.00554100
C	2.66100200	2.06504700	-2.28442500
C	4.82636200	2.85137700	-1.30769400
H	2.87666700	3.62215300	-0.83563800
H	1.57124900	2.03212000	-2.18028700
H	2.90293600	2.66341000	-3.17362300
H	3.01353400	1.03902000	-2.47702900
H	5.35984400	3.22306800	-0.42827800
H	5.29756000	1.90246700	-1.60301100
H	4.97909800	3.56846000	-2.12868000
H	-1.54107900	-3.62047800	0.83379900
C	-0.57369300	-4.02072000	-1.75373700
H	-1.60268000	-4.38823000	-1.65925800
H	-0.62098200	-3.00661700	-2.16366600
H	-0.05962800	-4.65323900	-2.48550500
C	0.13222500	-4.61787600	2.74891600
H	0.68638600	-5.38347700	2.19493800
H	0.57357500	-4.55372300	3.74896600
H	-0.90095100	-4.96863300	2.86474800

5-ts

C	-1.50968700	0.83429500	0.22598900
H	-1.30183100	1.04789700	1.28092600
C	-0.22714800	0.28050400	-0.42110500
H	0.19987600	-0.96623800	-0.04841200
C	0.99660800	1.03531400	-0.25751400
O	2.09156900	0.69603300	-0.77857500
O	0.94628000	2.07402400	0.60797500
C	2.13852600	2.86972600	0.83368000
C	2.28218900	3.87575500	-0.31772800
C	1.96432900	3.52299100	2.20577000
H	1.41271100	4.54545000	-0.30628600
H	2.23758400	3.31405400	-1.25658400
H	2.80120700	4.21536500	2.36033500
H	1.05178000	4.13339600	2.18341900
C	-4.40704800	-1.53346900	0.96281300
C	-4.39161500	-1.51030000	-0.61659700
B	-2.69936700	-0.20561400	0.20788800
O	-3.09299600	-0.91795900	-0.90397100
O	-3.49759100	-0.44962400	1.29917400
C	-2.01538200	2.13718300	-0.40217700
C	-2.18207300	3.29413200	0.37267700
C	-2.34387700	2.20699800	-1.76623600
C	-2.64820300	4.48320000	-0.19215300
H	-1.93562400	3.26019600	1.43128700
C	-2.80784300	3.39357900	-2.33540000
H	-2.23988800	1.31909900	-2.38404600
C	-2.96170300	4.53945500	-1.55128000
H	-2.76446000	5.36686000	0.43123800
H	-3.05377800	3.42196700	-3.39445100
H	-3.32309300	5.46401400	-1.99436900
C	-5.44366600	-0.57201400	-1.22442400
H	-5.24701000	-0.46567500	-2.29582100
H	-5.39628400	0.42495800	-0.77557100
H	-6.45802200	-0.96520800	-1.09733500
C	-4.46094900	-2.88018800	-1.28919600
H	-4.40136100	-2.75820700	-2.37565000
H	-5.40758800	-3.38206100	-1.05684300
H	-3.63735400	-3.52683400	-0.97846300
C	-5.76284200	-1.23893200	1.60382400
H	-5.65948400	-1.23822900	2.69381700
H	-6.49947000	-2.00467500	1.33400900
H	-6.14774700	-0.26217900	1.30230800
C	-3.80777200	-2.81267900	1.56257900
H	-4.46071200	-3.67843600	1.40760400
H	-3.67512900	-2.66984200	2.63965300

H	-2.82678500	-3.03308900	1.13005100
Li	2.45770900	-1.14165200	-0.52751400
C	5.09393500	-0.34446100	-1.51239700
O	4.31841300	-1.42512500	-0.93765900
C	5.19901000	-2.42160500	-0.36062000
C	6.62818800	-1.93573700	-0.64034100
C	6.44341600	-0.41749500	-0.80334700
H	4.53171700	0.57649900	-1.34933500
H	5.19909600	-0.51741300	-2.59201700
H	4.97318300	-3.39174500	-0.81259200
H	4.98499800	-2.47774200	0.71276700
H	7.00686500	-2.37261400	-1.57148700
H	7.32010200	-2.20434900	0.16310000
H	7.24607600	0.05470600	-1.37749800
H	6.38680700	0.07324900	0.17559500
H	-0.35374900	0.02552000	-1.47589600
N	0.93040900	-2.18710700	0.16604700
C	0.74720700	-2.49306800	1.59240200
C	1.48010800	-3.74995600	2.10419900
C	1.18181200	-1.28966100	2.44194000
H	-0.32966900	-2.66548800	1.80186300
H	1.13314600	-4.66261600	1.60986800
H	1.31081700	-3.87753600	3.18174300
H	2.56291200	-3.66371800	1.93898200
H	0.64545100	-0.37912000	2.16259400
H	2.25861400	-1.09993100	2.32186800
H	0.99726200	-1.47598000	3.50667600
C	0.47970300	-3.27254500	-0.72670900
C	-0.20139800	-2.72694800	-1.99471900
C	1.63080900	-4.20730800	-1.16776600
H	-0.26998900	-3.89774200	-0.20573800
H	-1.11720800	-2.17708800	-1.76555600
H	-0.45746100	-3.54909800	-2.67491500
H	0.47386000	-2.05254200	-2.54073000
H	2.17846700	-4.61997700	-0.31679300
H	2.34763800	-3.65106200	-1.78910600
H	1.25509000	-5.04565400	-1.77085300
C	3.58282200	4.68735500	-0.28026000
H	3.66410900	5.32187300	-1.16968400
H	3.63979300	5.34350200	0.59522800
H	4.46203500	4.03044200	-0.26238400
C	1.90450900	2.53108300	3.37112400
H	2.81832100	1.92586200	3.42441100
H	1.79810400	3.05740400	4.32644900
H	1.05733900	1.84746300	3.26523300
H	3.00238800	2.19507300	0.84045000

C-6

C	-1.87154100	0.59812400	0.70845600
H	-1.79690300	0.04664900	1.64994400
C	-0.60562900	0.29839400	-0.09857000
H	1.34364600	1.86358400	0.03851300
C	0.29729800	-0.69246000	0.18476000
O	1.34300100	-1.00598000	-0.51395800
O	0.09692900	-1.38184200	1.37803400
C	0.55444000	-2.74234500	1.45909500
C	1.70334100	-2.80597000	2.47521700
C	-0.66348100	-3.62342900	1.78331500
H	1.35571500	-2.41687700	3.44042400
H	2.47819200	-2.11308800	2.12434700
H	-0.36672500	-4.67916700	1.70904600
H	-1.40594100	-3.44701700	0.99562500
C	-4.54403400	-1.63475800	-0.82978700
C	-4.24540100	-0.61552400	-1.99734700
B	-3.00326600	-0.07872400	-0.14992300
O	-3.55728600	0.45512900	-1.29892400
O	-3.48082100	-1.34741200	0.11373000
C	-2.01400100	2.07760700	1.03173700
C	-1.70957400	2.54803700	2.31913600
C	-2.40796100	3.02160600	0.06755200
C	-1.78866400	3.90579500	2.63666600
H	-1.40930700	1.83438500	3.08382300
C	-2.48515200	4.38095400	0.38032600
H	-2.67405000	2.68060600	-0.92836000
C	-2.17517000	4.83164400	1.66579000
H	-1.55245300	4.23902400	3.64490200
H	-2.79890900	5.09018000	-0.38259800
H	-2.24074800	5.88928000	1.90892700
C	-3.26452000	-1.16161600	-3.04485200
H	-2.96586500	-0.34439800	-3.70943500
H	-2.36004800	-1.55469000	-2.57012800
H	-3.71756100	-1.95235000	-3.65266200
C	-5.47917100	-0.03242500	-2.68513900
H	-5.16754900	0.68984100	-3.44708600
H	-6.05984800	-0.81875200	-3.18172200
H	-6.12899800	0.48639300	-1.97646200
C	-4.46231400	-3.11064700	-1.21911800
H	-4.64462200	-3.73287600	-0.33661300
H	-5.21893200	-3.36139400	-1.97214200

H	-3.47696700	-3.36827100	-1.61461400
C	-5.86759100	-1.35518800	-0.10319800
H	-6.73476500	-1.60953500	-0.72257500
H	-5.90504900	-1.96001300	0.80836500
H	-5.94368000	-0.30267500	0.18764800
Li	2.68128300	0.15550700	-0.73711000
C	3.83589700	-2.00256100	-2.17924800
O	4.16561000	-0.68982100	-1.64462400
C	5.57729600	-0.61810000	-1.33327000
C	6.17780000	-1.96317400	-1.76497000
C	4.96596100	-2.90788900	-1.69954900
H	2.84393400	-2.25615600	-1.80141900
H	3.81093500	-1.93606300	-3.27506800
H	6.01032200	0.23847700	-1.85946400
H	5.67998800	-0.45379700	-0.25338800
H	6.55552000	-1.90138100	-2.79207500
H	7.00527700	-2.27222300	-1.11974300
H	5.08252200	-3.79928100	-2.32293900
H	4.78114700	-3.23133400	-0.66851200
H	-0.48244400	0.82089800	-1.04270400
N	2.33881200	2.09929100	-0.07419900
C	2.81675200	2.47055500	1.28778700
C	4.32456200	2.73758100	1.31997000
C	2.43492800	1.36354600	2.27406800
H	2.30502300	3.39715700	1.59947400
H	4.61185100	3.58859200	0.69435300
H	4.63971600	2.96232300	2.34517800
H	4.88479000	1.85701700	0.97944100
H	1.36499600	1.13879100	2.23282600
H	2.97904600	0.43504900	2.05887900
H	2.68446300	1.66745400	3.29678700
C	2.43711000	3.19517100	-1.08566400
C	1.14392200	3.28326100	-1.90346000
C	3.64517700	2.99569400	-2.01216500
H	2.56233200	4.15064200	-0.55250500
H	0.27692300	3.48812800	-1.26675200
H	1.21922200	4.08432300	-2.64727800
H	0.95636400	2.34505000	-2.44083800
H	4.58019300	2.90203700	-1.45220200
H	3.52168000	2.08800200	-2.61661700
H	3.74607900	3.84464600	-2.69892000
C	-1.31349500	-3.35836600	3.14604700
H	-2.21747000	-3.96626700	3.26711600
H	-0.64113500	-3.59931600	3.97843400
H	-1.60151400	-2.30651800	3.23624100
C	2.30681400	-4.20370600	2.65658000
H	2.63412300	-4.62432800	1.69658600
H	3.17991900	-4.17144500	3.31886100
H	1.58843900	-4.90542900	3.09497000
H	0.94886000	-3.03130100	0.47942200

7-ts

C	1.04469600	0.19692700	-0.52104100
H	0.71008800	-0.71737100	-1.02858200
C	0.20227900	0.43657000	0.74787300
H	-0.10748900	-0.84018100	1.19910300
C	-1.14411000	0.90345700	0.52076800
O	-1.94044900	0.37406300	-0.29445400
O	-1.56480500	1.88571600	1.35568700
C	-2.94835400	2.32326700	1.28909000
C	-3.06806500	3.40930600	0.21157500
C	-3.33752200	2.74765200	2.71037400
H	-2.40498100	4.24551700	0.46413400
H	-2.69121500	2.98433500	-0.72488300
H	-4.40912300	2.98633100	2.71336500
H	-3.21579200	1.87289700	3.36112600
C	4.75442000	-0.64444500	-0.45511200
C	4.62372800	0.29797400	0.80929300
B	2.57642000	0.00093800	-0.17651200
O	3.27478100	0.82243700	0.67301400
O	3.36613200	-0.96422300	-0.75574800
C	1.00552300	1.31413800	-1.57024400
C	1.00921500	0.99898400	-2.93793100
C	1.01957600	2.67049500	-1.20801100
C	1.00980300	1.99922700	-3.91214700
H	1.01093900	-0.04663400	-3.23987300
C	1.02091400	3.67303800	-2.17919100
H	1.02252800	2.93903100	-0.15568000
C	1.01484000	3.34375000	-3.53670600
H	1.00393300	1.72650900	-4.96499100
H	1.02821000	4.71653400	-1.87291900
H	1.01252700	4.12552200	-4.29197400
C	4.67452400	-0.45136300	2.14747900
H	4.38560100	0.23655300	2.94824500
H	3.97774900	-1.29454600	2.16386800
H	5.68051800	-0.82749300	2.36260700
C	5.59423800	1.47903300	0.84105700
H	5.39501500	2.08984500	1.72747700
H	6.63287500	1.13240300	0.89502000

H	5.48324200	2.11705400	-0.03846500
C	5.50979100	-1.95110100	-0.21709200
H	5.51136200	-2.54584000	-1.13636700
H	6.55181900	-1.75527400	0.06116800
H	5.04478700	-2.54964900	0.56956100
C	5.31206400	0.06651800	-1.69626900
H	6.37495200	0.30583400	-1.58360900
H	5.19762400	-0.59302500	-2.56227300
H	4.76578800	0.99136000	-1.90626500
Li	-1.80903400	-1.51635800	-0.18537500
C	-3.49352300	-1.42068100	-2.56482600
O	-2.86606300	-2.29143000	-1.58863900
C	-3.46463900	-3.60997800	-1.63318000
C	-4.48577600	-3.57077600	-2.77776900
C	-4.83991000	-2.07646400	-2.85952400
H	-3.54867900	-0.42451800	-2.12281400
H	-2.86038100	-1.37965500	-3.46074900
H	-2.67146200	-4.34867100	-1.78314300
H	-3.94074300	-3.79828300	-0.66384600
H	-4.02450400	-3.90242900	-3.71512000
H	-5.35020300	-4.21180300	-2.58211500
H	-5.24521800	-1.78235500	-3.83214200
H	-5.57147500	-1.80967900	-2.08760200
H	0.71811400	1.02129800	1.50986100
N	-0.74208900	-2.10335200	1.38513000
C	-1.51066400	-2.08252000	2.65046800
C	-3.01835700	-1.90623200	2.37925500
C	-1.03837200	-1.02051300	3.66650900
H	-1.40091300	-3.06226900	3.14454900
H	-3.39583600	-2.72106100	1.74681900
H	-3.60403900	-1.90397700	3.30790500
H	-3.21568900	-0.95416100	1.86751400
H	0.03050400	-1.12609000	3.87746900
H	-1.20266800	-0.00501400	3.28995100
H	-1.58459200	-1.11815500	4.61437700
C	0.12441100	-3.28713700	1.29356700
C	1.31195900	-3.28729400	2.28083000
C	0.64731000	-3.49955500	-0.13475200
H	-0.48470600	-4.18009100	1.53768700
H	0.97175300	-3.21354700	3.31934500
H	1.90622400	-4.20650600	2.19037900
H	1.97047800	-2.43324000	2.08150800
H	-0.17455300	-3.49528600	-0.86384100
H	1.36939700	-2.72921400	-0.42414500
H	1.15639300	-4.46795600	-0.21665100
H	-3.55834900	1.46483600	0.99013400
C	-4.50122500	3.91392100	0.00531000
H	-4.88672600	4.43314900	0.89009800
H	-4.54558000	4.61692600	-0.83382800
H	-5.18645800	3.08677600	-0.21990800
C	-2.53712800	3.92317900	3.28313700
H	-2.82559300	4.11320000	4.32304100
H	-1.46400000	3.70943200	3.26260400
H	-2.70847700	4.84770400	2.72003300

C-8

C	2.02450900	0.44236100	-1.00043300
H	1.91883500	0.25774500	-2.08166900
C	1.34380700	1.75519200	-0.65397500
H	-1.59893900	-1.04205600	-1.40627100
C	0.24669200	1.52655100	0.08285000
O	-0.04095800	0.24672900	0.39775200
O	-0.77411200	2.31488100	0.56568300
C	-0.68721700	3.76407600	0.60712100
C	0.51756300	4.24423500	1.42531800
C	-0.80781200	4.37403600	-0.79818400
H	1.44531300	4.01406400	0.89184700
H	0.45214500	5.33768400	1.48764700
H	-1.61106000	3.83802000	-1.31916700
H	0.10862500	4.19488200	-1.36965600
C	0.55438600	-2.98796700	-0.47082600
C	1.75673900	-2.80521500	0.53814200
B	1.08120600	-0.69039900	-0.19911200
O	1.64965100	-1.43266100	0.90293200
O	0.44513000	-1.69128800	-1.07458300
C	3.50597600	0.43511700	-0.69050000
C	4.45257000	0.14829800	-1.68563800
C	3.98712500	0.71866900	0.59958900
C	5.82266100	0.13533800	-1.41042000
H	4.10663100	-0.06681300	-2.69528900
C	5.35250000	0.71249000	0.88008800
H	3.27405300	0.93815900	1.38911500
C	6.28103100	0.41946700	-0.12393600
H	6.53066200	-0.09077400	-2.20487700
H	5.69591400	0.93326700	1.88867400
H	7.34595400	0.41473900	0.09550500
C	1.66644300	-3.65254900	1.81340100
H	2.53236000	-3.43964500	2.44956600
H	0.76645600	-3.42005400	2.38968700

H	1.67013000	-4.72602300	1.58690900
C	3.12606400	-3.05301900	-0.12294700
H	3.91150900	-2.70878300	0.55654500
H	3.29198800	-4.11583900	-0.33655300
H	3.22941400	-2.48888400	-1.05344100
C	-0.76692700	-3.32028400	0.24916100
H	-1.58794200	-3.30282000	-0.47741700
H	-0.75244400	-4.31819300	0.70263800
H	-0.98103000	-2.58659800	1.03334900
C	0.78834400	-4.02191500	-1.57776200
H	0.94696100	-5.02531500	-1.16359400
H	-0.08735600	-4.06605100	-2.23659200
H	1.65275000	-3.75677200	-2.19040200
C	-1.55183000	-0.20773100	3.20241500
O	-2.57173600	-0.23290100	2.15125900
C	-3.69445700	-1.03763400	2.57022100
C	-3.16091000	-1.91406200	3.70144500
C	-2.16043500	-0.96798700	4.38467100
H	-0.65316800	-0.68300300	2.80047100
H	-1.32558900	0.83787600	3.42511200
H	-4.50088900	-0.37740300	2.91775300
H	-4.04969500	-1.60068000	1.70279700
H	-3.95356900	-2.26445200	4.36914000
H	-2.64407300	-2.78866000	3.29037900
H	-2.68680200	-0.28229500	5.05907100
H	-1.39925800	-1.49583100	4.96601300
Li	-1.91636100	0.28805400	0.41311100
H	1.68771300	2.72683600	-0.98443400
N	-2.41471800	-0.41964300	-1.48377500
C	-3.63116600	-1.27727700	-1.56287500
C	-3.83515600	-2.07509100	-2.86319600
C	-4.91569700	-0.52820600	-1.17294900
H	-3.45800900	-2.01946700	-0.77082600
H	-2.93071300	-2.62604800	-3.14066500
H	-4.64421700	-2.80318300	-2.72632000
H	-4.11474600	-1.42954200	-3.70219300
H	-4.76966100	0.05945200	-0.25910000
H	-5.25895200	0.15073700	-1.95776900
H	-5.72228100	-1.24758100	-0.98761300
C	-2.03449000	0.54355500	-2.56906900
C	-3.14398300	1.53123400	-2.93978400
C	-1.41342700	-0.11237800	-3.81650400
H	-1.22675300	1.12873700	-2.11314500
H	-3.58215700	2.00254300	-2.05293100
H	-2.72392800	2.32378200	-3.56937000
H	-3.94964100	1.05840200	-3.51217700
H	-0.63886000	-0.82812500	-3.52188500
H	-2.15192500	-0.62603900	-4.43938100
H	-0.93759100	0.65866100	-4.43477800
H	-1.59818400	4.02149300	1.16280400
C	-1.12797400	5.87453400	-0.78413800
H	-0.30984800	6.46637500	-0.35958100
H	-1.30054600	6.23930900	-1.80244300
H	-2.03222900	6.08617900	-0.19919200
C	0.57496300	3.65436400	2.83806700
H	-0.35022000	3.85696600	3.39337500
H	0.72223100	2.57040000	2.80756900
H	1.40557400	4.08866100	3.40496300

9-ts

C	2.34717400	0.34187600	-0.45803100
C	0.92068200	0.23149700	0.02488200
H	-3.57421100	-1.46068800	-0.46145600
C	-0.09332500	1.10992200	-0.35746800
O	-1.27866200	1.12728100	0.11394500
O	0.23825800	1.97337500	-1.36969500
C	-0.75402400	2.87773200	-1.89835200
C	-1.27843800	2.29749700	-3.21968300
C	-0.10003500	4.26343600	-1.99773900
H	-0.43630500	2.14373200	-3.90518000
H	-1.68849100	1.30318500	-3.00321600
H	-0.87258100	4.98571600	-2.29434000
H	0.21751000	4.54742700	-0.98662400
C	5.17400800	-2.05368800	0.36323000
C	4.09453000	-2.47618800	1.43926000
B	3.26319900	-0.83344900	0.07160900
O	3.08624600	-1.43877300	1.29488000
O	4.38790100	-1.27913800	-0.58225700
C	4.58311000	-2.46942600	2.88802400
H	3.75537500	-2.73336300	3.55467200
H	4.95318300	-1.48527300	3.18443500
H	5.38366200	-3.20357600	3.03588900
C	3.40798600	-3.81253400	1.12757400
H	2.54581700	-3.93220400	1.79148100
H	4.08261900	-4.66117500	1.28351700
H	3.04667900	-3.84249300	0.09557700
C	6.25029200	-1.10832500	0.91600300
H	6.84162700	-0.71970900	0.08111300
H	6.92580200	-1.62284900	1.60792000

H	5.80311300	-0.25357900	1.43281300
C	5.83213500	-3.21051500	-0.38814000
H	6.40451000	-3.84835600	0.29557400
H	6.52411400	-2.81198100	-1.13711100
H	5.09628000	-3.82911900	-0.90753900
Li	-2.69562700	0.41602700	0.92874400
C	-3.79549700	1.07584200	3.77313600
O	-2.78111100	1.12572300	2.74186600
C	-1.67882400	1.97387700	3.16563500
C	-2.26150400	2.84397900	4.27482500
C	-3.23507700	1.87079000	4.95922100
H	-3.99978300	0.02752600	4.01326500
H	-4.71081500	1.52829700	3.37424100
H	-1.32774800	2.51233600	2.28508900
H	-0.86692900	1.33538700	3.53650500
H	-2.80309800	3.69669900	3.84846100
H	-1.49248600	3.23040200	4.94997700
H	-4.02253200	2.37150700	5.52989100
H	-2.69125200	1.20828900	5.64238800
H	0.72416600	-0.34854200	0.92023900
N	-4.10402700	-0.96474600	0.27007400
C	-5.25840500	-0.32268800	-0.41803100
C	-6.02015900	0.61372400	0.52556400
C	-4.73557800	0.42831000	-1.64698700
H	-5.95933400	-1.09182800	-0.77371200
H	-6.42712100	0.08670000	1.39530500
H	-6.86130000	1.07762500	-0.00147000
H	-5.36713100	1.41904600	0.88811300
H	-4.20582200	-0.25201900	-2.32240000
H	-4.04090900	1.22728000	-1.35733200
H	-5.56646500	0.88366800	-2.19734700
C	-4.43653000	-1.99639300	1.29596700
C	-5.26023400	-3.17459000	0.75014900
C	-3.12362500	-2.50235900	1.90894100
H	-5.01480200	-1.49533900	2.08322700
H	-6.24496700	-2.86225200	0.38770600
H	-5.42407500	-3.91883500	1.53824100
H	-4.72712800	-3.65958800	-0.07576600
H	-2.55272400	-1.68728800	2.37153200
H	-2.50134000	-2.97296200	1.13842700
H	-3.32256800	-3.25054500	2.68427400
C	1.56571500	-2.31714600	-2.68642600
C	0.75843200	-2.43102300	-1.61785300
H	1.40443700	-1.55128800	-3.44097600
H	2.40750800	-2.98510200	-2.83929400
H	0.93425300	-3.21975600	-0.88969300
C	-0.36830000	-1.55663700	-1.35811400
H	-0.57836600	-0.74358900	-2.03476300
H	-0.82995300	-1.52903000	-0.38959400
Br	-2.27562300	-2.86861300	-2.14830300
C	3.08141800	1.62410700	-0.03101900
C	3.94536200	2.28324900	-0.91765300
C	2.96249600	2.13119800	1.27201500
C	4.65064000	3.42293900	-0.52578300
H	4.06235600	1.89656000	-1.92757500
C	3.66821300	3.26803700	1.66907600
H	2.30315600	1.62684800	1.97344900
C	4.51488300	3.92236600	0.77101600
H	5.30594100	3.92165000	-1.23610600
H	3.55771900	3.64368400	2.68402600
H	5.06153000	4.81039100	1.07801500
H	2.37858800	0.31282900	-1.55613600
C	-2.35799200	3.15291800	-3.89357600
H	-1.96721300	4.12348200	-4.21947300
H	-2.75852800	2.64467300	-4.77763000
H	-3.19799700	3.34387000	-3.21346400
C	1.09908200	4.35941700	-2.94739500
H	1.53010900	5.36674800	-2.91863200
H	1.88030300	3.65166200	-2.65557200
H	0.81759800	4.15247800	-3.98661000
H	-1.58083000	2.93153700	-1.18404700

C-10

C	1.99984700	0.25849500	0.51546000
H	2.18753400	1.03654300	1.26228500
C	0.94005500	0.79558700	-0.49576500
H	-1.86806900	-2.39422200	0.69475900
C	-0.32190700	1.28007800	0.19783400
O	-1.44892900	0.83976900	-0.06271100
O	-0.11330000	2.24537100	1.07114200
C	-1.25054900	2.95121300	1.69393400
C	-1.76398500	3.99776800	0.69789000
C	-0.71990000	3.53664700	2.99960900
H	-0.97242700	4.73718700	0.52708200
H	-1.94089000	3.49953200	-0.26256900
H	-1.50964700	4.17075000	3.41887300
H	0.12186900	4.20008600	2.76440100
C	5.63514400	-0.03530400	-0.61534700
C	4.91848200	-1.10134800	-1.53748200

B	3.39118500	-0.07171300	-0.18401900
O	3.50014200	-0.76514500	-1.36103300
O	4.58783500	0.29716200	0.35482700
C	1.50998400	-0.98930800	1.24953800
C	1.34240000	-0.98434000	2.64170300
C	1.25556700	-2.18697700	0.55694400
C	0.92021300	-2.12859600	3.32438300
H	1.56251600	-0.07907400	3.20083200
C	0.83496000	-3.33309500	1.23626500
H	1.42961700	-2.23111200	-0.51503000
C	0.66092100	-3.30805300	2.62388500
H	0.80936900	-2.10028000	4.40495100
H	0.67670500	-4.25698200	0.68516600
H	0.34981800	-4.20380300	3.15410000
C	5.25272800	-1.00489700	-3.02379100
H	4.67807600	-1.75237500	-3.57987400
H	5.01362400	-0.02057300	-3.43249400
H	6.31641200	-1.20315400	-3.19511900
C	5.08414500	-2.54547300	-1.04921800
H	4.42046200	-3.19635300	-1.62697700
H	6.11108400	-2.89849600	-1.18539600
H	4.82101600	-2.64632800	0.00843800
C	5.98516000	1.27194100	-1.33682700
H	6.29513800	2.01424700	-0.59515400
H	6.80815700	1.13264600	-2.04487800
H	5.12624900	1.67781000	-1.88099800
C	6.84984100	-0.55309900	0.15155100
H	7.63956300	-0.86829600	-0.53944800
H	7.25291200	0.24513300	0.78235600
H	6.59440800	-1.39592100	0.79743200
Li	-2.74694000	-0.40268300	-0.50899800
C	-5.16118700	-0.41439300	-2.35918100
O	-3.97045300	0.23871900	-1.83418400
C	-3.78765300	1.53235100	-2.48246000
C	-5.08916700	1.79974400	-3.23666400
C	-5.54848700	0.38038900	-3.60484200
H	-4.91274100	-1.45930700	-2.56293900
H	-5.94258600	-0.37592500	-1.59152600
H	-3.56424300	2.26869100	-1.70675600
H	-2.93011900	1.45598900	-3.16132500
H	-5.82193900	2.28338000	-2.58094100
H	-4.93636900	2.44234900	-4.10783400
H	-6.61835900	0.31529500	-3.81975000
H	-5.00138600	0.01375700	-4.48063100
H	0.61931800	-0.03485300	-1.13132700
N	-2.83602200	-2.26407200	0.38266000
C	-3.68167800	-2.28878600	1.61567800
C	-5.13864200	-1.94003800	1.29846700
C	-3.08825700	-1.31327100	2.63658300
H	-3.65890300	-3.29262400	2.06204800
H	-5.60074100	-2.65348200	0.60864600
H	-5.73170000	-1.94695000	2.21868100
H	-5.21718600	-0.93645300	0.85938100
H	-2.04587500	-1.55935600	2.86903900
H	-3.12065500	-0.28029400	2.26576800
H	-3.65881300	-1.35040600	3.57022200
C	-3.09899200	-3.34181700	-0.62127600
C	-2.90990400	-4.76481300	-0.07598700
C	-2.19777500	-3.09938600	-1.83873500
H	-4.14177800	-3.22580700	-0.93838400
H	-3.59754700	-4.99328800	0.74357800
H	-3.09109000	-5.49978800	-0.86749900
H	-1.88517800	-4.90934400	0.28797500
H	-2.39046500	-2.12312100	-2.30224900
H	-1.13830100	-3.14084500	-1.55711900
H	-2.36749900	-3.86424500	-2.60329400
C	1.82771900	4.36630900	-1.11380400
C	2.13208200	3.10059700	-0.82151700
H	1.04440000	4.61821200	-1.82661300
H	2.36115500	5.19999000	-0.66596000
H	2.93780100	2.89936900	-0.11618000
C	1.48241100	1.90073400	-1.45805500
H	2.21344400	1.38958200	-2.09844000
H	0.67115700	2.22798300	-2.12158100
H	-2.02495200	2.20265400	1.89107600
C	-3.05768900	4.68845700	1.14840300
H	-2.91481900	5.28344500	2.05571700
H	-3.41608800	5.36754400	0.36805600
H	-3.85369900	3.95982600	1.34749200
C	-0.30137000	2.49446500	4.03989300
H	-1.13662500	1.83388200	4.30305200
H	0.51823300	1.87166900	3.66993500
H	0.03822300	2.98292600	4.95867900

11-ts

C	2.14749300	1.24109300	0.09671100
C	0.77431500	0.66538500	0.35585500
H	-4.03949500	0.76941300	-0.35946700
C	0.18570700	-0.34467900	-0.39975900

O	-0.93882100	-0.89719600	-0.16403400
O	0.89202700	-0.71123800	-1.52514400
C	0.29672500	-1.61474100	-2.48143300
C	0.41165200	-0.93644500	-3.85265100
C	0.99722300	-2.97716200	-2.40579100
H	1.47438900	-0.79035900	-4.08790600
H	-0.02536800	0.06600500	-3.76355700
H	2.03891400	-2.85868000	-2.73490200
H	0.51789000	-3.64534400	-3.13321100
C	4.81689900	-1.45973100	0.35556800
C	5.27167900	-0.68055100	-0.94301800
B	3.30910000	0.18650500	-0.13909300
O	4.38917600	0.47100800	-0.94034200
O	3.44229000	-1.01296800	0.52634700
C	6.71615300	-0.17921900	-0.92648400
H	6.91900100	0.37898200	-1.84630800
H	6.90273900	0.48774000	-0.08175900
H	7.42226400	-1.01637500	-0.87577300
C	4.99933100	-1.44422600	-2.24580200
H	5.16503100	-0.77022200	-3.09222600
H	5.66296900	-2.30851500	-2.35759900
H	3.96235900	-1.78888400	-2.28868800
C	5.57541700	-1.03316700	1.62074300
H	5.08517000	-1.47706600	2.49312000
H	6.61722900	-1.37108800	1.60248900
H	5.56147500	0.05347700	1.74759000
C	4.82624200	-2.98283700	0.23416100
H	5.84345900	-3.35362100	0.06129800
H	4.45899200	-3.42767500	1.16503400
H	4.18623600	-3.32944000	-0.58005600
Li	-2.55280200	-0.99147900	0.59878400
C	-3.45929500	-2.93448200	2.84882600
O	-2.45264900	-2.14693700	2.16813800
C	-1.13158700	-2.50591900	2.65172700
C	-1.29641500	-3.91796800	3.20310800
C	-2.70208000	-3.84983100	3.82341900
H	-4.15735000	-2.25753400	3.35229500
H	-4.00894400	-3.50439700	2.09162700
H	-0.44613600	-2.40944400	1.80959200
H	-0.83793600	-1.79913900	3.43956800
H	-1.26582800	-4.65024000	2.38782700
H	-0.51928100	-4.17871900	3.92743200
H	-3.17953800	-4.82876500	3.92412800
H	-2.65499000	-3.39451700	4.81916400

H	0.31743700	0.85433400	1.32076600
N	-4.36194500	-0.11927100	0.05106400
C	-5.04725500	-0.86252000	-1.04222300
C	-5.34491600	-2.30814300	-0.62970200
C	-4.16852200	-0.81034900	-2.29663900
H	-6.00197400	-0.37575300	-1.28831400
H	-5.98437400	-2.36399600	0.25812500
H	-5.86118100	-2.83447500	-1.44034800
H	-4.41512500	-2.85356300	-0.41712700
H	-3.97478500	0.22643800	-2.59243100
H	-3.20204000	-1.29968100	-2.12133400
H	-4.66355000	-1.32170300	-3.12985500
C	-5.21161000	0.26303400	1.21820600
C	-6.40029000	1.16752800	0.85247400
C	-4.31758500	0.95684800	2.25499500
H	-5.59640200	-0.66712900	1.65702500
H	-7.10621900	0.67697000	0.17442400
H	-6.95536000	1.44427800	1.75639700
H	-6.04256600	2.08592200	0.37331600
H	-3.50368500	0.30148100	2.58884200
H	-3.88343100	1.86953500	1.82960700
H	-4.90041300	1.24133000	3.13827100
C	0.48550000	3.69359700	-1.86316500
C	-0.23630600	3.40917300	-0.76343200
H	0.45871800	3.05398700	-2.74198400
H	1.10444600	4.58373700	-1.92294100
H	-0.19546300	4.07624100	0.09460300
C	-1.06814900	2.23580200	-0.63557900
H	-1.12393800	1.52374600	-1.44251600
H	-1.45288700	1.93304700	0.32047000
Br	-3.34251600	3.08068500	-1.15541600
C	2.63403700	2.11317000	1.25350900
C	3.12367400	3.40820000	1.02819200
C	2.64479600	1.62680200	2.57201100
C	3.59462000	4.19598000	2.08039800
H	3.12836700	3.80199700	0.01533500
C	3.11220600	2.41231700	3.62659600
H	2.29052000	0.61737200	2.76432300
C	3.58860100	3.70309500	3.38646800
H	3.96282600	5.19897500	1.87823500
H	3.10784800	2.01321200	4.63830200
H	3.95086500	4.31676600	4.20721200
H	2.12362500	1.88128000	-0.80113600
C	-0.28313100	-1.69203400	-4.99185600

H 0.18836900 -2.66009600 -5.19411800
H -0.24549500 -1.10936500 -5.91885100
H -1.33998600 -1.87620500 -4.76100600
C 0.96445100 -3.62378200 -1.01730100
H 1.43837800 -4.61244400 -1.03886500
H -0.06636500 -3.75082000 -0.66597100
H 1.49872600 -3.00622700 -0.28880300
H -0.75723200 -1.73873000 -2.21664500

C-12

C 2.21640900 -0.85430100 0.74534000
H 2.73161300 -0.54460600 1.66187200
C 0.71007400 -1.06193000 1.09051300
H -2.90983500 -2.15557000 0.51575000
C -0.00028000 0.24088000 1.40610100
O -1.07773400 0.57872300 0.90317200
O 0.63167500 0.96863900 2.30920100
C 0.06605800 2.25494800 2.76502400
C -0.91408400 1.97330100 3.90724100
C 1.26810200 3.13911700 3.10524100
H -0.38813300 1.45196000 4.71506900
H -1.68284300 1.28654900 3.53305300
H 0.88715100 4.14855400 3.30362900
H 1.88784700 3.21920000 2.20364800
C 3.59187400 1.89552900 -1.50891000
C 2.35210000 1.37714500 -2.34152900
B 2.50087200 0.24808300 -0.35886900
O 1.60508900 0.60329100 -1.34234700
O 3.69767500 0.89550500 -0.45214000
C 2.86213000 -2.15450700 0.26412800
C 3.90419900 -2.74890100 0.98941900
C 2.44103700 -2.77251200 -0.92525700
C 4.50419000 -3.92918000 0.54609300
H 4.25079600 -2.28116400 1.90779000
C 3.03639800 -3.95479000 -1.36788700
H 1.64918500 -2.31794300 -1.51647500
C 4.07076800 -4.53792100 -0.63285100
H 5.31076800 -4.37205500 1.12376800
H 2.69701700 -4.41692200 -2.29111300
H 4.53709600 -5.45619300 -0.97792500
C 1.44046100 2.47068300 -2.89169900
H 0.59641200 2.01419400 -3.42028700
H 1.04518700 3.10816400 -2.09690800
H 1.97994300 3.10269800 -3.60569500
C 2.73151000 0.39390900 -3.45538500
H 1.81996900 -0.05132900 -3.86750800
H 3.26379900 0.89511300 -4.26982400
H 3.36145400 -0.41627100 -3.07607000
C 3.34754900 3.24125300 -0.81401400
H 4.16147900 3.42711200 -0.10696700
H 3.32195800 4.06821400 -1.53093000
H 2.40666700 3.23883200 -0.25321500
C 4.91570600 1.93241700 -2.26979000
H 4.86388100 2.63327300 -3.11049100
H 5.71257400 2.26832800 -1.59927700
H 5.19196600 0.94716900 -2.65097700
Li -2.37359700 0.09581900 -0.35431200
C -3.21666000 1.28792100 -3.00821800
O -2.78701800 1.45801800 -1.62777800
C -2.53980700 2.86754900 -1.35202700
C -3.14404100 3.62402800 -2.53394300
C -2.94855700 2.62946200 -3.68908700
H -2.65343200 0.45514100 -3.43903200
H -4.28331100 1.03605200 -3.00804800
H -3.00145100 3.10857700 -0.39050500
H -1.45699700 3.01628000 -1.27779800
H -4.20997200 3.81359700 -2.36537200
H -2.65101700 4.58467100 -2.70541300
H -3.62349800 2.80710800 -4.53051100
H -1.91907900 2.67185600 -4.06204200
H 0.19562800 -1.47556000 0.21839900
N -3.46011800 -1.65418200 -0.18982100
C -4.80912700 -1.42012800 0.41258100
C -5.71441500 -0.62569900 -0.53267000
C -4.63004000 -0.68914500 1.74734600
H -5.29492700 -2.38283800 0.62561500
H -5.93240600 -1.16830600 -1.45797000
H -6.67223700 -0.42127400 -0.04337600
H -5.25641600 0.33601800 -0.79546800
H -3.97392600 -1.24560300 2.42632000
H -4.20256700 0.31255500 1.60516000
H -5.59712600 -0.56731100 2.24550100
C -3.42555100 -2.49350700 -1.42685700
C -4.04353200 -3.88844900 -1.25463500
C -1.97183400 -2.59769600 -1.90307700
H -3.99517800 -1.94966700 -2.18912000
H -5.10592500 -3.84478100 -0.99738200
H -3.95698300 -4.45729200 -2.18649300
H -3.52382000 -4.45213900 -0.46957100

H	-1.53028400	-1.61071000	-2.09484900
H	-1.34845700	-3.11162000	-1.16072100
H	-1.91335700	-3.16911500	-2.83501300
C	-1.47481900	-3.54936300	2.51307100
C	-0.88319400	-2.35983800	2.66323400
H	-0.94787000	-4.39930900	2.08372700
H	-2.49133000	-3.73089000	2.85353000
H	-1.44531800	-1.54919100	3.13032500
C	0.54048400	-2.05635200	2.27880100
H	1.05458300	-2.98134600	2.00035300
H	1.07124600	-1.64056300	3.14596300
H	-0.47450300	2.67893700	1.91369900
C	2.12849700	2.66138400	4.28058600
H	2.50138600	1.64572500	4.11363100
H	2.99476800	3.31889400	4.40373400
H	1.57479700	2.66915500	5.22549400
C	-1.58657300	3.24223200	4.44689000
H	-2.33663900	2.98306600	5.20054900
H	-0.86547600	3.91750400	4.91873900
H	-2.09529300	3.79783800	3.64954200

5-ts-Me

C	-1.53750400	1.68267800	-0.36199400
H	-1.32418000	2.10532700	0.62821000
C	-0.36550000	0.77110800	-0.76122500
H	-0.23215600	-0.43503400	-0.11890700
C	0.98448700	1.27014200	-0.62871600
O	2.00366600	0.61163100	-0.96903000
O	1.13045000	2.45462300	0.01770400
C	2.46585000	2.96187100	0.27052000
C	3.00586100	3.61983000	-1.00796400
C	2.34081600	3.92730600	1.45090300
H	2.38580900	4.49547900	-1.24245600
H	2.87671000	2.90735800	-1.82869300
H	3.31797300	4.40185100	1.60495600
H	1.64421200	4.72826300	1.16919000
C	-4.76990300	-0.07002800	0.67074000
C	-4.84557400	-0.11424300	-0.90641000
B	-2.89090700	0.88357400	-0.24024700
O	-3.48453200	0.21773800	-1.29353400
O	-3.65279200	0.83017700	0.90447000
C	-5.74713100	0.97542700	-1.50514300
H	-5.59643400	1.00374600	-2.58887900
H	-5.49653400	1.96308600	-1.10514500
H	-6.80673600	0.77953500	-1.30848500
C	-5.20621000	-1.47326500	-1.50481600
H	-5.18473600	-1.40875400	-2.59762900
H	-6.21530800	-1.77850600	-1.20363800
H	-4.50132900	-2.25005000	-1.20063500
C	-6.00406900	0.50598600	1.36544900
H	-5.83798500	0.52705600	2.44745800
H	-6.88830200	-0.11208600	1.17056300
H	-6.21327300	1.52725200	1.03858400
C	-4.39683400	-1.41654700	1.30539700
H	-5.21566300	-2.14048200	1.23010200
H	-4.17385000	-1.25816000	2.36529800
H	-3.50637900	-1.84732300	0.83759100
Li	1.94101800	-1.17362900	-0.36242700
C	4.69979500	-1.17969600	-1.32854300
O	3.69308400	-1.93194600	-0.60757800
C	4.31121000	-3.00890900	0.13990300
C	5.80967200	-2.94368300	-0.18682800
C	6.00337300	-1.47224200	-0.59065300
H	4.38542200	-0.13476900	-1.32332800
H	4.73737900	-1.53898400	-2.36587200
H	3.84236800	-3.95245800	-0.15387200
H	4.11322500	-2.83701500	1.20406900
H	6.04771100	-3.60185200	-1.03034100
H	6.43053500	-3.24602000	0.66143500
H	6.88606500	-1.31001000	-1.21629800
H	6.08993600	-0.83502800	0.29749600
H	-0.48910000	0.33615200	-1.75778200
N	0.19510800	-1.71056500	0.39050100
C	-0.09040500	-1.65878400	1.83276200
C	0.30947900	-2.91300600	2.63639200
C	0.59905000	-0.43533500	2.45350000
H	-1.18055900	-1.52982400	1.99237000
H	-0.23054100	-3.80631200	2.30734900
H	0.08517600	-2.76964800	3.70184900
H	1.38601400	-3.11118300	2.54031300
H	0.29916400	0.49299900	1.96112200
H	1.69274400	-0.52574800	2.37571500
H	0.35151900	-0.34558800	3.51799500
C	-0.46965500	-2.83358500	-0.29727800
C	-0.97502000	-2.43292800	-1.69519100
C	0.44681400	-4.06944600	-0.45647800
H	-1.35552100	-3.15587900	0.28221400
H	-1.74544000	-1.65913200	-1.65078400
H	-1.39501500	-3.30358400	-2.21454500

H	-0.14992000	-2.05233300	-2.31382900
H	0.86628100	-4.39794000	0.49783100
H	1.28565900	-3.82942900	-1.12599100
H	-0.09629900	-4.91550400	-0.90028400
C	4.48258200	4.02562000	-0.92495200
H	4.82709900	4.41947200	-1.88757200
H	4.66108100	4.80128000	-0.17208200
H	5.11689400	3.16570100	-0.67434500
C	1.88098100	3.27223800	2.75648200
H	2.57138800	2.47848400	3.06840300
H	1.83343700	4.00933400	3.56606000
H	0.88843700	2.82713400	2.64503300
H	3.10459700	2.11243500	0.53937600
C	-1.73233700	2.86482100	-1.34756200
H	-0.82102900	3.46862600	-1.41382300
H	-1.97261200	2.49662300	-2.35224900
H	-2.55019800	3.52866900	-1.03412900

7-ts-Me

C	1.14453700	0.24140500	-1.24955000
H	0.80040500	-0.78939500	-1.41408000
C	0.34280700	0.87536100	-0.08903800
H	0.15386900	-0.14572700	0.83603400
C	-1.04679600	1.15123400	-0.34915300
O	-1.85259800	0.32047200	-0.84612900
O	-1.49640600	2.34335900	0.12119600
C	-2.91339100	2.64674300	0.04800900
C	-3.22319800	3.23090900	-1.33715000
C	-3.21868700	3.56355100	1.23914500
H	-2.60708900	4.12363100	-1.49843700
H	-2.90595400	2.49141800	-2.08043600
H	-4.30354800	3.72782000	1.27484200
H	-2.96123400	3.01376500	2.15283700
C	4.83849000	-0.55783200	-0.72634700
C	4.74993600	0.94351800	-0.24005100
B	2.68003900	0.19949500	-0.89111700
O	3.42649100	1.33800300	-0.68900900
O	3.43759300	-0.94689900	-0.79718800
C	4.76601100	1.09927500	1.28720300
H	4.51079500	2.13347900	1.53880900
H	4.02915300	0.44748500	1.76636500
H	5.75302800	0.87573200	1.70644900
C	5.77288100	1.89103100	-0.86756500
H	5.60011200	2.90826100	-0.50141100
H	6.79488400	1.60231800	-0.59539000
H	5.69333800	1.90931100	-1.95692600
C	5.56104600	-1.51008800	0.22530200
H	5.53288700	-2.52635300	-0.18126400
H	6.61211200	-1.22121900	0.34259900
H	5.09215100	-1.53037000	1.21138000
C	5.40112900	-0.70970200	-2.14671100
H	6.47501800	-0.49741800	-2.18424200
H	5.24184200	-1.73949100	-2.48164500
H	4.89061500	-0.04425600	-2.85014900
Li	-1.63025500	-1.35087800	0.02451400
C	-3.59143900	-2.21049100	-1.95683900
O	-2.83712200	-2.61383000	-0.78744900
C	-3.42345100	-3.80117600	-0.19987900
C	-4.63228000	-4.15841700	-1.07854800
C	-4.97607500	-2.81619000	-1.74705200
H	-3.56189700	-1.12059100	-1.99886000
H	-3.10583700	-2.61924100	-2.85330700
H	-2.66494100	-4.58955100	-0.16917300
H	-3.71593200	-3.55613300	0.82725600
H	-4.35033000	-4.89760300	-1.83700800
H	-5.45793900	-4.57476300	-0.49434900
H	-5.53116900	-2.93208400	-2.68265200
H	-5.56769000	-2.18683900	-1.07174400
H	0.84744800	1.73876500	0.34870500
N	-0.38277900	-1.24849700	1.56379900
C	-0.97351600	-0.74924300	2.82562600
C	-2.51284500	-0.71586700	2.73887300
C	-0.44833700	0.63183700	3.27310400
H	-0.72676700	-1.45635100	3.63506600
H	-2.90907000	-1.71735900	2.52429100
H	-2.97062300	-0.36691300	3.67408500
H	-2.84496000	-0.03812300	1.94006500
H	0.64325300	0.62928000	3.35903800
H	-0.72277800	1.41548600	2.55863500
H	-0.86598300	0.90787400	4.25066000
C	0.51675100	-2.38913900	1.79477800
C	1.84333600	-2.02384700	2.49462400
C	0.81421000	-3.14474200	0.49111000
H	-0.00234300	-3.10699000	2.46026500
H	1.66415600	-1.50354500	3.44162600
H	2.43777500	-2.92115900	2.71444700
H	2.44247500	-1.37101700	1.84979100
H	-0.11349800	-3.44947000	-0.01285800
H	1.40403600	-2.53587500	-0.20111600

H	1.39153600	-4.05486400	0.69648800
H	-3.46761400	1.70934400	0.16389800
C	-4.70463400	3.56436400	-1.54957800
H	-5.04426500	4.37107200	-0.89014000
H	-4.88362900	3.88658500	-2.58139700
H	-5.34142700	2.69043800	-1.36135700
C	-2.48430800	4.90926000	1.23067700
H	-2.70007900	5.46976200	2.14724200
H	-1.40170500	4.76103200	1.17074000
H	-2.78695400	5.53548700	0.38356900
C	0.97469200	1.00602800	-2.58844700
H	1.32404300	2.04041600	-2.49320800
H	1.54369500	0.53443700	-3.40209300
H	-0.07822100	1.02858300	-2.89312100

9-ts-Me

C	2.42451100	1.21470700	0.61827300
C	1.03974200	0.61539000	0.65695700
H	-3.02136000	-0.90992900	-1.37198400
C	-0.12602500	1.34545000	0.89057200
O	-1.29377600	0.86027900	1.05951300
O	0.02732700	2.71256800	0.86961900
C	-1.13089700	3.55948600	1.02321700
C	-1.68064600	3.91092200	-0.36678400
C	-0.69048100	4.75488000	1.87950100
H	-0.89137600	4.39346100	-0.95640800
H	-1.91792800	2.96818800	-0.87360000
H	-1.57815800	5.35697400	2.11448100
H	-0.32835800	4.35552300	2.83548700
C	5.67075400	-0.55938300	-0.20473900
C	4.65411300	-1.76879500	-0.25413700
B	3.53920100	0.19546700	0.16775100
O	3.43759700	-1.17553300	0.27503400
O	4.78979400	0.59224300	-0.25567500
C	5.02362500	-2.96656500	0.62161600
H	4.23961000	-3.72778900	0.55010000
H	5.12320700	-2.68425300	1.67225900
H	5.96544700	-3.41914500	0.29039800
C	4.34227100	-2.24572200	-1.67881700
H	3.49766500	-2.94155500	-1.64474400
H	5.19647100	-2.76411000	-2.12743100
H	4.06559400	-1.40886000	-2.32615200
C	6.44372000	-0.46473500	1.11942200
H	6.97092000	0.49382900	1.15292900
H	7.18179600	-1.26801400	1.21890200
H	5.76743000	-0.50535400	1.97920900
C	6.64584600	-0.47616000	-1.37926300
H	7.30537800	-1.35147200	-1.40680800
H	7.27178900	0.41557800	-1.27080000
H	6.12252400	-0.40296400	-2.33530900
Li	-2.32181500	-0.59971300	1.00997100
C	-2.61544100	-2.78933400	3.20829700
O	-2.13415500	-1.52748400	2.71011200
C	-1.14709800	-0.97978900	3.63542300
C	-0.92681300	-2.05892300	4.70953300
C	-1.46270600	-3.33849500	4.04385600
H	-2.88278900	-3.40639200	2.34691700
H	-3.51377200	-2.62670600	3.82109100
H	-1.53662500	-0.04315100	4.04421700
H	-0.24881700	-0.75656800	3.05540200
H	-1.50682800	-1.83140900	5.61111500
H	0.12445000	-2.13793700	5.00014500
H	-1.78470900	-4.09686900	4.76364100
H	-0.70392700	-3.78359100	3.38994200
H	0.95394400	-0.45701500	0.80802700
N	-3.50470700	-1.30149800	-0.55096600
C	-4.84730100	-0.65866200	-0.49591500
C	-5.54808500	-0.95770100	0.83423800
C	-4.67630500	0.84893000	-0.71249800
H	-5.48400900	-1.03839600	-1.30828600
H	-5.69392300	-2.03162700	0.99388100
H	-6.53625800	-0.48488000	0.85510600
H	-4.97064200	-0.56313500	1.68123100
H	-4.19501400	1.05156900	-1.67548700
H	-4.05600100	1.29727200	0.07384800
H	-5.65191200	1.34761500	-0.70298300
C	-3.49051800	-2.78144200	-0.73839600
C	-4.15256600	-3.24977100	-2.04503600
C	-2.03365400	-3.26063400	-0.68336900
H	-4.03389200	-3.21898600	0.10968400
H	-5.21707000	-2.99844500	-2.08735200
H	-4.07081700	-4.33887700	-2.14085600
H	-3.65372600	-2.79047300	-2.90608600
H	-1.55132700	-2.98240600	0.26227600
H	-1.46007600	-2.82248900	-1.50847300
H	-1.98162700	-4.35118000	-0.77751200
C	2.03720400	1.10816400	-2.96201500
C	1.31726600	0.10115200	-2.43776100
H	1.70139900	2.14006300	-2.89789900

H 2.97764400 0.93095200 -3.47495500
H 1.67292500 -0.92207600 -2.52953300
C 0.06032800 0.28485100 -1.74080000
H -0.33861200 1.27806300 -1.61308100
H -0.34747300 -0.49729800 -1.12972600
Br -1.60721600 -0.29518800 -3.44043300
H 2.44271200 2.05735000 -0.08929200
C -2.93248300 4.79599700 -0.33783100
H -2.72249900 5.79126600 0.07021600
H -3.33278500 4.93195500 -1.34851100
H -3.72497200 4.34610600 0.27375300
C 0.39270400 5.64406100 1.25715100
H 0.70132500 6.42538600 1.96112600
H 1.27744800 5.05555700 0.99493300
H 0.03857100 6.14337100 0.34793400
H -1.89441000 2.99746800 1.56997700
C 2.86736000 1.79488400 1.99841500
H 2.90561500 1.00711000 2.76216500
H 3.86177200 2.25473600 1.93634400
H 2.15616100 2.55465400 2.33630400

11-ts-Me

C 2.05455400 -1.51465300 -1.26691400
C 0.80277600 -0.73598100 -0.94780300
H -3.84558000 -0.51413700 -0.15284900
C 0.48557100 -0.23480100 0.30964200
O -0.53757100 0.47710000 0.59642000
O 1.35282700 -0.60105600 1.31556100
C 1.04114500 -0.27581200 2.68586100
C 1.05917700 -1.59262800 3.47364900
C 2.05287400 0.75939300 3.19166700
H 2.06569000 -2.02786600 3.41564300
H 0.39070400 -2.29373600 2.95922200
H 3.05393400 0.30587300 3.18394800
H 1.82107300 0.98361100 4.24091300
C 5.02799300 0.85058200 -1.03076800
C 5.60863400 -0.46252100 -0.37549800
B 3.40705900 -0.76435900 -0.94231400
O 4.57926500 -1.43876900 -0.67911700
O 3.59604500 0.60172700 -1.01307400
C 6.93107200 -0.95544800 -0.96361000
H 7.22420100 -1.88798700 -0.47054600
H 6.84857400 -1.15462100 -2.03462000
H 7.73076100 -0.22245500 -0.80425400
C 5.71245700 -0.38780500 1.15398400
H 5.93233800 -1.38705400 1.54277100
H 6.51022900 0.29080500 1.47505900
H 4.76887100 -0.05540600 1.59649900
C 5.42320700 1.02201700 -2.50531400
H 4.82639200 1.83038400 -2.93975500
H 6.48252700 1.27749700 -2.61688000
H 5.22384000 0.11118000 -3.07870400
C 5.31296700 2.14222900 -0.26523700
H 6.39054700 2.33920000 -0.21861200
H 4.83864800 2.98531000 -0.77885900
H 4.92100200 2.10526000 0.75344400
Li -2.05432400 1.26184200 0.05789500
C -2.50468300 4.25568400 -0.62732600
O -1.69363900 3.05648900 -0.59951700
C -0.32031500 3.37868700 -0.94224800
C -0.17728800 4.85505500 -0.58894600
C -1.55508500 5.40793400 -0.99167700
H -3.31222300 4.12025400 -1.35454000
H -2.94694700 4.38400800 0.36685400
H 0.31921100 2.69974300 -0.37804200
H -0.17199700 3.20655900 -2.01678200
H -0.00967500 4.97260100 0.48802500
H 0.64990600 5.33550000 -1.11945600
H -1.81749500 6.33632600 -0.47629600
H -1.58412900 5.60217100 -2.06980500
H 0.25173700 -0.29522000 -1.77410600
N -3.98801900 0.48225500 0.06577700
C -4.56345300 0.53452900 1.43899100
C -4.55187800 1.96556800 1.98808100
C -3.76790400 -0.41748900 2.33886800
H -5.60573500 0.18485500 1.42605100
H -5.11789600 2.65734500 1.35437000
H -5.00133400 1.99133500 2.98706000
H -3.52436300 2.34481900 2.07752300
H -3.82177700 -1.44399500 1.96008900
H -2.71036400 -0.12802500 2.38253500
H -4.17025900 -0.40400900 3.35797200
C -4.85007600 1.01128400 -1.03255200
C -6.19155600 0.27392100 -1.17959000
C -4.05465600 0.93416600 -2.34291000
H -5.04875000 2.06749900 -0.80689200
H -6.82319800 0.37255400 -0.29085100
H -6.75512800 0.68206400 -2.02669300
H -6.01857000 -0.79268500 -1.36296200

H	-3.13080100	1.52266700	-2.28721200
H	-3.79411800	-0.10711000	-2.56676900
H	-4.64892400	1.32104100	-3.17836600
C	0.05122700	-4.40955800	-0.63974900
C	-0.62998400	-3.38773500	-0.09276800
H	0.15522800	-4.51338300	-1.71686100
H	0.51267500	-5.17771300	-0.02668200
H	-0.72769000	-3.32914700	0.98920800
C	-1.25332200	-2.32240100	-0.85040300
H	-1.55787800	-1.41720500	-0.36064100
H	-1.16519600	-2.31003100	-1.92646600
Br	-3.61420900	-2.95843200	-0.93242500
H	2.05915900	-2.45203300	-0.68607000
C	0.62454100	-1.45758300	4.93809200
H	1.32579800	-0.85207900	5.52291600
H	0.56544300	-2.44208800	5.41527700
H	-0.36579000	-0.99168200	5.01806000
C	2.06594600	2.06023300	2.38206500
H	2.78066500	2.77622800	2.80536200
H	1.07636600	2.53339700	2.38195500
H	2.34988300	1.86877700	1.34247300
H	0.03667200	0.15691600	2.71251800
C	2.07978300	-1.91174600	-2.76426900
H	2.95437100	-2.52843300	-3.00045300
H	1.18327200	-2.48319300	-3.03553000
H	2.10944900	-1.02352600	-3.40895000

Br-

Br	0.00000000	0.00000000	0.00000000

C-6-Me

C	-1.93010800	-0.95456300	-1.47399400
H	-1.91989200	-0.11777700	-2.17967600
C	-0.71439300	-0.82712800	-0.56306400
H	1.49625700	-1.84779700	-1.07966600
C	0.00507100	0.32050800	-0.34693600
O	0.96345100	0.48599200	0.51125000
O	-0.28823300	1.39163100	-1.18619200
C	-0.08719300	2.72282900	-0.68264300
C	1.08169000	3.36134900	-1.44578700
C	-1.43075000	3.46304600	-0.79257000
H	0.86499000	3.34296600	-2.52119700
H	1.95306100	2.71226800	-1.29487900
H	-1.34527100	4.43268700	-0.28178900
H	-2.16670500	2.87241700	-0.23443000
C	-4.84221200	0.16670700	0.72114100
C	-4.47934300	-1.25101400	1.31177500
B	-3.13590900	-0.78392400	-0.48221200
O	-3.67186000	-1.82574200	0.25376400
O	-3.72871800	0.42645300	-0.16955300
C	-3.58524800	-1.17790400	2.55870700
H	-3.22036700	-2.18396900	2.79003900
H	-2.71502700	-0.53824000	2.38052400
H	-4.12922800	-0.79945400	3.43116800
C	-5.66906000	-2.17480200	1.57136400
H	-5.30867400	-3.14079000	1.94057500
H	-6.33935600	-1.75150300	2.32893600
H	-6.24356900	-2.35705400	0.66012500
C	-4.91945100	1.29836000	1.74608200
H	-5.14393400	2.24083100	1.23544700
H	-5.71380400	1.11327600	2.47914800
H	-3.97381200	1.42190500	2.27897700
C	-6.10965600	0.15623800	-0.14629600
H	-7.01683200	0.03402400	0.45581000
H	-6.17766300	1.10704700	-0.68447900
H	-6.07265700	-0.64842500	-0.88754600
Li	2.47871000	-0.44526100	0.44269900
C	3.09916700	1.02667700	2.78505200
O	3.71635800	0.14431400	1.80444300
C	5.12744700	0.44107100	1.68270700
C	5.42898300	1.51035800	2.74021900
C	4.06548800	2.20045800	2.91336600
H	2.10830300	1.27943400	2.40330300
H	3.00333700	0.47891700	3.73181500
H	5.69370600	-0.48437700	1.82706000
H	5.31398100	0.81154100	0.66665400
H	5.73959100	1.04117700	3.68092300
H	6.22435200	2.19157200	2.42409400
H	3.96603800	2.72109200	3.87036100
H	3.89303700	2.92536000	2.10919200
H	-0.52312400	-1.64882400	0.12364700
N	2.51016100	-1.98294500	-0.95792800
C	3.11597300	-1.69905400	-2.28851900
C	4.64661700	-1.72680700	-2.24761100
C	2.60800700	-0.34580000	-2.79510000
H	2.78677800	-2.47681700	-2.99998400
H	5.04041900	-2.71391900	-1.98579600
H	5.04896200	-1.46308000	-3.23224400
H	5.02963900	-0.99979200	-1.51964200
H	1.51538100	-0.28826700	-2.77185200

H	2.99612300	0.47707000	-2.18155200
H	2.94247500	-0.17977300	-3.82514500
C	2.73340900	-3.37162900	-0.45469600
C	1.43298700	-3.94560100	0.11896400
C	3.84628300	-3.41462900	0.60251900
H	3.03585200	-4.00623300	-1.30292900
H	0.63917600	-3.97764700	-0.63461100
H	1.59704800	-4.96402700	0.48788300
H	1.07406200	-3.33787900	0.95855800
H	4.78582800	-2.99878000	0.22740800
H	3.55434500	-2.84119000	1.49147900
H	4.03650200	-4.44789900	0.91685900
C	-1.94072700	3.66952800	-2.22349400
H	-2.94318100	4.11294000	-2.21608700
H	-1.29058800	4.33703500	-2.80220700
H	-1.99976400	2.71266300	-2.75114800
C	1.41862700	4.78928300	-1.00038500
H	1.61034200	4.83506800	0.07976500
H	2.31496800	5.15934100	-1.51196100
H	0.60356900	5.48840900	-1.21919700
H	0.19286000	2.65339900	0.37373000
C	-1.94183700	-2.28038900	-2.25591400
H	-2.81566900	-2.35730600	-2.91666000
H	-1.03957900	-2.37974500	-2.87501900
H	-1.97659600	-3.13959800	-1.57434400

C-6-Cy

C	-1.86113600	0.42776600	0.66499500
H	-1.73192500	-0.11491700	1.60803700
C	-0.52856500	0.35606600	-0.07498500
H	1.44501300	2.09380400	-0.02074900
C	0.48169900	-0.52459200	0.21876400
O	1.56224800	-0.72769400	-0.46945900
O	0.35638700	-1.23017900	1.41395100
C	0.92275300	-2.54889000	1.48589800
C	2.09290800	-2.52220200	2.47965200
C	-0.21448200	-3.52371800	1.83476200
H	1.73420000	-2.16021100	3.45125800
H	2.80599200	-1.77195700	2.11512600
H	0.16360800	-4.55294200	1.75613200
H	-0.98351200	-3.40545600	1.06277200
C	-3.96190100	-2.28321700	-1.00061400
C	-3.94834100	-1.15216800	-2.10084200
B	-2.81195000	-0.44476600	-0.23661200
O	-3.50738500	0.00530100	-1.34627600
O	-2.97578900	-1.80782300	-0.05278800
C	-2.90248800	-1.38595400	-3.20101000
H	-2.82459400	-0.48052900	-3.81155600
H	-1.91605900	-1.58593600	-2.77078400
H	-3.17777700	-2.22040600	-3.85536800
C	-5.30606700	-0.83559300	-2.72702900
H	-5.19758800	-0.01385200	-3.44279400
H	-5.70205700	-1.70411200	-3.26659900
H	-6.03686100	-0.53020800	-1.97430200
C	-3.53983500	-3.66913900	-1.48990400
H	-3.54696800	-4.37209100	-0.65012400
H	-4.23217700	-4.04601000	-2.25230500
H	-2.53116000	-3.65738600	-1.90961400
C	-5.29377500	-2.38081300	-0.24170200
H	-6.09209500	-2.79636500	-0.86651200
H	-5.15797500	-3.03522200	0.62524600
H	-5.61270600	-1.39994200	0.12473900
Li	2.90183500	0.42260800	-0.62258700
C	4.17138100	-1.73160400	-1.95216900
O	4.45386300	-0.40404800	-1.42666700
C	5.84266800	-0.31144700	-1.02830500
C	6.47687900	-1.66752800	-1.36989700
C	5.26646000	-2.61654500	-1.36592400
H	3.15508700	-1.98159200	-1.64221200
H	4.22847000	-1.69221900	-3.04812800
H	6.30552100	0.52765500	-1.55749200
H	5.87462300	-0.10615600	0.04888400
H	6.93196700	-1.63705100	-2.36649500
H	7.25349700	-1.95157900	-0.65390500
H	5.43158700	-3.52456500	-1.95329600
H	5.00723200	-2.91165200	-0.34245200
H	-0.44324900	0.87027500	-1.02895000
N	2.43479800	2.36788000	-0.06966600
C	2.78855100	2.83107600	1.30155400
C	4.28057000	3.14617800	1.44131100
C	2.35351200	1.77037500	2.31652100
H	2.23039800	3.75963100	1.51493300
H	4.59619400	3.97215600	0.79622500
H	4.50253300	3.43361400	2.47513400
H	4.89005400	2.26649100	1.19683400
H	1.29702300	1.50994900	2.19870100
H	2.93538700	0.84761900	2.19991300
H	2.50878000	2.13841700	3.33672000
C	2.57703500	3.40212200	-1.13786600

C	1.36549700	3.36182700	-2.07532300
C	3.87441300	3.21150300	-1.93757000
H	2.60508100	4.39568400	-0.66269800
H	0.43613300	3.58302500	-1.54029800
H	1.47853500	4.09799800	-2.87860300
H	1.26141100	2.37236500	-2.53795400
H	4.75682000	3.19903900	-1.29163100
H	3.85004600	2.26568600	-2.49354100
H	3.99857900	4.02405100	-2.66337100
C	-0.85402600	-3.30590300	3.21076700
H	-1.71246700	-3.97371000	3.34735600
H	-0.15182700	-3.50192000	4.03035200
H	-1.21017200	-2.27581500	3.30912000
C	2.80544700	-3.86911900	2.65011100
H	3.14630800	-4.26531900	1.68419800
H	3.68537500	-3.76915500	3.29655500
H	2.15110500	-4.62354100	3.10121800
H	1.31954000	-2.80881900	0.49879400
C	-2.33301400	1.85838700	1.03632300
C	-2.50462900	2.81587500	-0.15978800
C	-3.63690200	1.82170900	1.85940100
H	-1.54813900	2.29071700	1.68222200
C	-2.93873600	4.22148300	0.28696700
H	-3.24995100	2.40265700	-0.85143000
H	-1.56427300	2.88016500	-0.72137000
C	-4.08975200	3.21977200	2.30907800
H	-4.43081200	1.36736000	1.24632600
H	-3.50650000	1.16862000	2.73327000
C	-4.23132400	4.17631000	1.11501300
H	-3.07211200	4.87332700	-0.58732900
H	-2.13975500	4.67465600	0.89447800
H	-5.03804100	3.15443000	2.85962500
H	-3.34934300	3.62990200	3.01271000
H	-4.50217700	5.18307200	1.46090500
H	-5.05655400	3.83256900	0.47333100

C-6-T

C	-1.82927200	0.70485300	0.69515700
H	-1.80718600	0.19756700	1.66489000
C	-0.57975600	0.28014100	-0.07669400
H	1.44814000	1.77865000	0.12469500
C	0.27535100	-0.72823500	0.29217200
O	1.32006600	-1.12243600	-0.36130600
O	0.00534600	-1.33452800	1.51317100
C	0.70865200	-2.53561700	1.86051500
C	0.69793600	-2.58915300	3.39325500
C	0.07567600	-3.75065000	1.15853600
H	-0.33944600	-2.60463300	3.74896400
H	1.12991800	-1.64815000	3.75628800
H	0.67693100	-4.64057400	1.39302800
H	0.17957400	-3.58642100	0.07960900
C	-4.63708600	-1.40275800	-0.77799800
C	-4.30785600	-0.41046700	-1.96118100
B	-3.00575700	0.07858900	-0.14209600
O	-3.54585900	0.62779900	-1.28952300
O	-3.54829300	-1.15616800	0.14844000
C	-3.38121400	-1.01623800	-3.02512500
H	-3.05653500	-0.22143800	-3.70447700
H	-2.48740100	-1.45211000	-2.56779000
H	-3.88820300	-1.78794900	-3.61454200
C	-5.52261600	0.23320500	-2.62833600
H	-5.18834400	0.93423700	-3.40042400
H	-6.15237800	-0.52446200	-3.10930300
H	-6.13171900	0.78841300	-1.91105000
C	-4.62999200	-2.88459700	-1.15272000
H	-4.82789800	-3.48867200	-0.26123900
H	-5.40828800	-3.10648600	-1.89256900
H	-3.66345000	-3.19236600	-1.55808700
C	-5.93464000	-1.05485400	-0.03430900
H	-6.82191200	-1.27122200	-0.63940800
H	-5.98748700	-1.65174700	0.88160200
H	-5.95630400	0.00170700	0.25110600
Li	2.63177900	0.01784500	-0.79132700
C	3.61182300	-2.25606100	-2.18758000
O	3.98732400	-0.90035800	-1.81785800
C	5.42895900	-0.76900800	-1.81490900
C	5.98655400	-2.14781800	-2.20212700
C	4.83344700	-3.09684100	-1.83340200
H	2.70148400	-2.49114100	-1.63387200
H	3.40559100	-2.28151300	-3.26610000
H	5.71396700	0.02237600	-2.51564300
H	5.73829200	-0.46956900	-0.80642600
H	6.18362200	-2.19214600	-3.27930700
H	6.92049900	-2.37618700	-1.68043800
H	4.86536600	-4.04354000	-2.38071300
H	4.84136400	-3.32016600	-0.76004800
H	-0.43121200	0.71949900	-1.05844600
N	2.44119900	1.96040700	-0.07359800
C	3.07773500	2.22793200	1.24699800

C	4.59384600	2.40949600	1.12874600
C	2.73820000	1.08934700	2.21314200
H	2.65708300	3.16092400	1.65888800
H	4.86356500	3.26722000	0.50467800
H	5.02686900	2.57507300	2.12165400
H	5.06242800	1.51344400	0.70101200
H	1.65788400	0.93671600	2.29305600
H	3.18696100	0.14285500	1.88463200
H	3.12695000	1.31607000	3.21217600
C	2.51135700	3.10156300	-1.03664200
C	1.14400400	3.34571700	-1.68479200
C	3.58048500	2.85700500	-2.11111200
H	2.78298300	4.01074700	-0.47802500
H	0.37636400	3.58104200	-0.94041300
H	1.20488000	4.18258700	-2.38968900
H	0.81165600	2.46242000	-2.24376900
H	4.56233700	2.65405300	-1.67279600
H	3.30597400	1.99893600	-2.73838100
H	3.67596200	3.73205300	-2.76505500
C	-1.39723500	-4.00497800	1.49973700
H	-1.78423700	-4.85025600	0.91775600
H	-1.53540500	-4.24997900	2.55983900
H	-2.01016600	-3.12771900	1.27119700
C	1.47369400	-3.77253900	3.98221700
H	2.50849700	-3.79430800	3.61619400
H	1.51151900	-3.70683700	5.07556800
H	1.01250200	-4.73324900	3.72680500
H	1.74440300	-2.44708500	1.51236700
C	-1.89941800	2.19799000	0.91513200
C	-2.55006100	3.17083300	0.19948500
S	-0.97462800	2.94069600	2.21549000
C	-2.31796300	4.49999300	0.67969400
H	-3.17647200	2.93299600	-0.65215600
C	-1.48641800	4.53977500	1.76301400
H	-2.76175200	5.38573600	0.23561700
H	-1.16247300	5.40148200	2.33172800
